# Arene difunctionalization through an acyl-inserting Smiles rearrangement enabled by *N*-heterocyclic carbene catalysis

**DOI:** 10.1038/s41467-026-74546-3

**Published:** 2026-06-15

**Authors:** Qing-Zhu Li, Yan-Qing Liu, Long-Hai Hong, Ting Qi, Mei-Hao He, Peng-Tao Wang, Xin-Xin Kou, Jun-Long Li

**Affiliations:** 1 Anti-infective Agent Creation Engineering Research Centre of Sichuan Province, Sichuan Industrial Institute of Antibiotics, School of Pharmacy, Chengdu University, Chengdu, China; 2 Department of Pharmacy, the Thirteenth People’s Hospital of Chongqing, Chongqing Geriatrics Hospital, Chongqing, China

**Keywords:** Organocatalysis, Synthetic chemistry methodology

## Abstract

Arene difunctionalization offers a powerful strategy for the simultaneous installation of two functional groups in a single step. Despite recent advances, *ipso*/*para*-selective arene transformations remain underdeveloped. Herein, we report an *N*-heterocyclic carbene (NHC)-catalyzed radical protocol that addresses this challenge. The process features a unique generation of acyl-inserting Smiles rearrangement, wherein radical Meisenheimer intermediates are intercepted by NHC-bound radicals prior to rearomatization. Subsequent ketone deprotonation regenerates ionic Meisenheimer intermediates, thereby completing the rearrangement and affording 1,4-difunctionalized arenes. This organocatalytic protocol exhibits broad substrate scope, tolerates diverse functional groups, and delivers acylated aniline derivatives in excellent yields (96 examples, up to 98% yield). The synthetic potential is further showcased by a ring-expansion strategy to benzo[*b*]azepines and by late-stage functionalization of drug-like molecules. Mechanistic insights from combined experimental and computational studies shed light on the unique reactivity and the observed excellent site-selectivity.

## Abstract (web summary)

The authors report an N-heterocyclic carbene-catalyzed radical Smiles-type rearrangement which selectively produces 1,4-difunctionalized arenes.

## Introduction

**Fig. 1:**
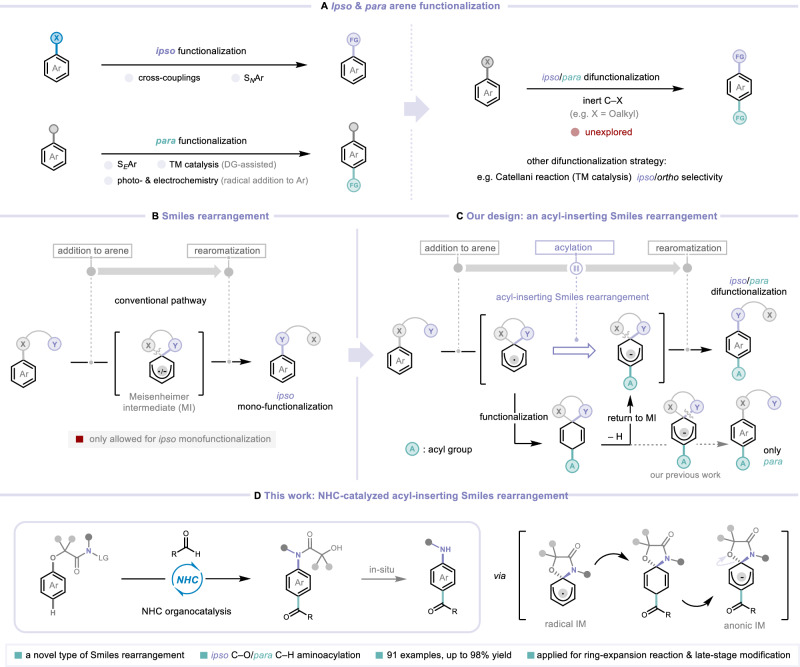
Background and research motivation for catalytic arene difunctionalization. **A** *Ipso* & *para* arene functionalization. **B** Smiles rearrangement. **C** Our design: an acyl-inserting Smiles rearrangement. **D** This work: NHC-catalyzed acyl-inserting Smiles rearrangement.

Aromatic rings, ubiquitous in pharmaceuticals and agrochemicals, are central targets for functionalization^1–3^. Over time, methods have advanced from classical electrophilic/nucleophilic substitution (S_*E*_Ar & S_*N*_Ar)^4–7^, to transition-metal-catalyzed cross-coupling^8–11^ and C–H activation^12–16^ and more recently, to radical functionalization driven by photo- or electrochemistry^17,18^, offering powerful strategies for *ipso*- or *para*-selective arene functionalization. Despite these progresses, current methods excel primarily at single-site modification, while multi-site installation typically relies on lengthy, waste-generating sequences. Consequently, strategies that enable simultaneous, site-selective introduction of multiple functionalities across an aromatic framework represent a critical challenge in synthetic chemistry^19–22^. As a landmark advance in arene difunctionalization, the Catellani reaction^23–27^ enables aryl halides to undergo *ortho* C–H activation followed by *ipso* C–X bond coupling, thus furnishing *ipso*/*ortho*-functionalized arenes within a single catalytic cycle^28–35^. However, the reaction flexibility was still limited, which in turn impeded the realization of diverse site-selectivities in arene difunctionalization. Accordingly, the development of a general and modular strategy to achieve other site-selective patterns, such as *ipso*/*para*-selectivity, remains a conspicuous unmet goal in modern synthesis. Building on the developing trajectory from the single C–X bond transformations to integrate C–X/C–H difunctionalization, we therefore postulated that the inert bond activation (e.g., C–O activation^36,37^ or Smiles-type *ipso*-substitution) might be repurposed to unlock a paradigm of C–O/C–H difunctionalizations featuring distinct arene site-selectivity (Fig. 1A).

The Smiles rearrangement, a venerable transformation first reported nearly a century ago, enables intramolecular aryl migration by converting an aryl C–X bond to C–Y bond^38–42^. While numerous mechanistic variants have been developed, the archetypal Smiles pathway—dearomative addition to generate anionic^43–47^ or radical^48–54^ Meisenheimer intermediates^55,56^ (MIs), followed by rapid rearomatization and aryl migration—has remained essentially unchanged. (Fig. 1B) This remarkable robustness reflects the difficulty of interrupting and redesigning the rearrangement sequence. Specifically, once the fleeting MI is generated, pausing the process to incorporate an additional functionalization event and then reinitiating the rearomatization represents a formidable challenge. Although functionalization of MI has been demonstrated to afford dearomatized cyclohexadienes, no strategy to date has enabled their subsequent rearomatization to ultimately complete the overall Smiles rearrangement^57–59^.

Recently, we disclosed a remote *para*-selective C–H functionalization^60^ of arenes by trapping the transient radical MI through *N*-heterocyclic carbene (NHC) catalysis^61–64^. Our previous finding revealed that incorporation of an electron-withdrawing acyl group onto the dearomatized cyclohexadiene species facilitates in-situ deprotonation, which in turn promotes the desired rearomatization via C–X bond cleavage and achieves the mono-functionalization. Inspired by this principle, we conceived a unique generation of acyl-inserting Smiles rearrangement in which the radical MI first undergoes *para*-selective acylation, and the newly introduced acyl moiety then triggers deprotonation to regenerate an anionic MI. Subsequent rearomatization via alternative C–Y bond cleavage accomplishes *ipso*/*para* difunctionalization of arenes in a single catalytic step (Fig. 1C).

As part of our continuing pursuit of NHC organocatalysis^65–71^, we herein establish an NHC-catalyzed radical reaction platform that unlocks a previously elusive arene difunctionalization. In contrast to conventional arene acylation strategies^4–7^, this method achieves precise *para*-selective acylation of readily available aryl ether substrates while simultaneously converting the *ipso* C–O bond into a C–N bond, thereby delivering diverse 4-acylated anilines in excellent yields (up to 98%). The protocol demonstrates broad generality and practical value, as exemplified by ring-expansion to furnish medicinally relevant benzo[*b*]azepines and late-stage diversification of drug-like scaffolds. Preliminary mechanistic investigations, supported by both experimental and DFT studies, not only corroborate the proposed NHC-radical catalytic cycle but also illuminate the origins of its remarkable site-selectivity (Fig. 1D).

## Results

### Reaction development

**Fig. 2:**
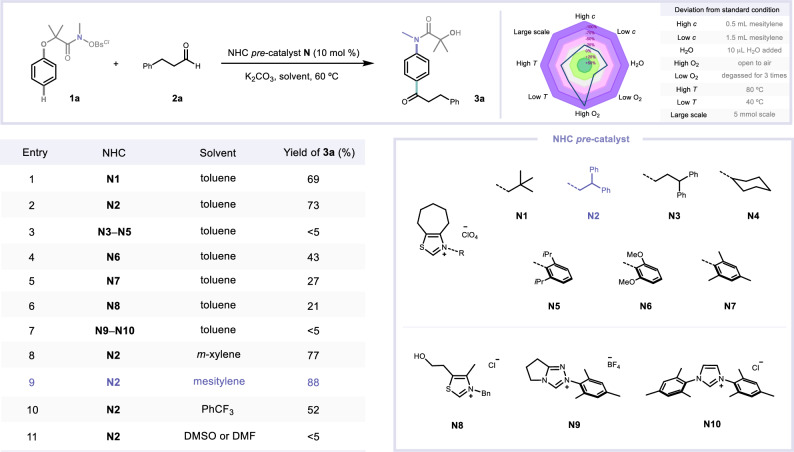
Reaction development. The reactions were conducted with **1a** (0.1 mmol), **2a** (0.25 mmol), NHC *pre*-catalyst (10 mol %) and K_2_CO_3_ (0.20 mmol) in 1 mL of solvent at 60 °C for 12 hours; isolated yield; Bs^*Cl*^: 4-chlorobenzenesulfonyl.

We commenced the investigation by selecting the aryl ether substrate **1a** and aldehyde **2a** as the model substrates to evaluate the proposed arene difunctionalization strategy (Fig. 2). To our gratification, the reaction proceeded smoothly when a *t*-amyl-substituted thiazolium salt (**N1**) was employed as the NHC *pre*-catalyst in combination with K_2_CO_3_ as the base in toluene at 60 °C, affording the desired 4-acylated aniline derivative **3a** in 69% yield (entry 1). Encouraged by this preliminary result, we systematically screened a variety of NHC catalysts. Replacing **N1** with **N2**, which features a 2,2-diphenylethyl substituent, led to an increased yield (entry 2). However, further extending the *N*-alkyl chain (**N3**) resulted in a complete loss of catalytic reactivity. No reaction occurred when the cyclohexyl-substituted analog **N4** was used. The *N*-aryl-substituted catalyst **N5** was also evaluated and proved entirely ineffective (entry 3). Thiazolium salts **N6** and **N7** exhibited unsatisfactory catalytic efficiency, yielding **3a** in low yields (entries 4–5). The commercially available catalyst **N8** also showed inferior catalytic reactivity (entry 6). Neither triazolium-nor imidazolium-based NHC precursors (**N9** and **N10**) promoted the target transformation (entry 7). Then, solvent screening revealed mesitylene to be optimal, which could increase the isolated yield of **3a** to 88% (entries 8–11). Next, a sensitivity analysis was undertaken to assess the robustness and reproducibility of the catalytic protocol under varied conditions^72,73^. As depicted in the radar chart, the transformation tolerated substantial deviations in substrate concentration (±50%), reaction temperature (±20 °C), residual moisture (≤15 wt %), and reaction scale (up to 5 mmol), while maintaining high conversion and isolated yield. In contrast, the reaction was substantially inhibited by high oxygen concentrations, indicating the requirement for anaerobic reaction conditions.

### Substrate scope

**Fig. 3:**
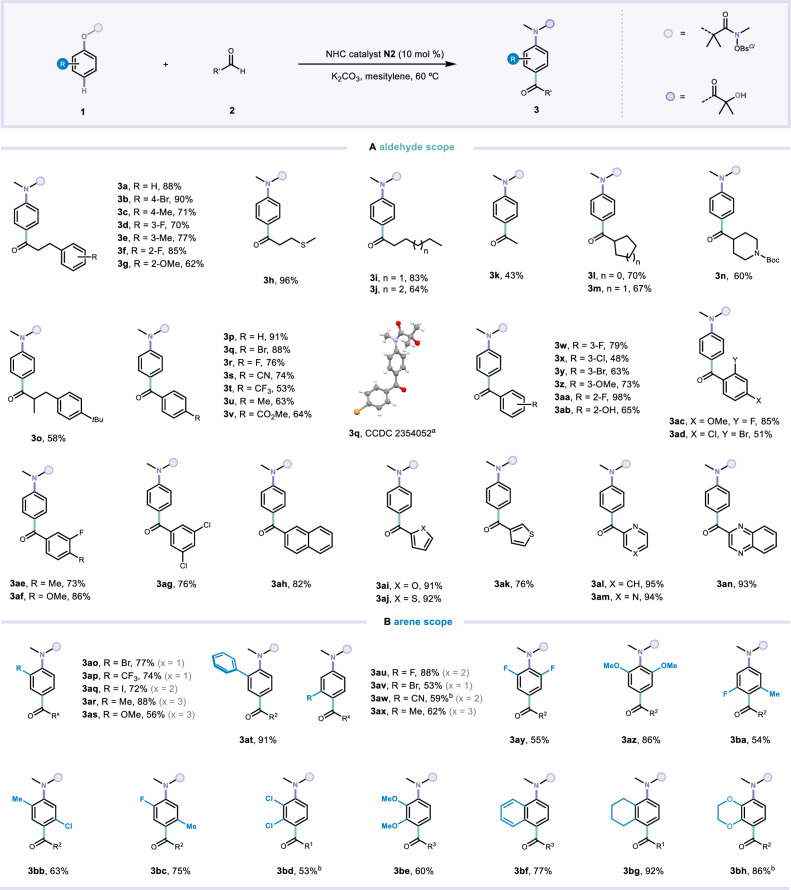
Scope of NHC-catalyzed arene difunctionalization. **A** Aldehyde scope. **B** Arene scope. Unless otherwise noted, reactions were carried out with **1** (0.10 mmol), **2** (0.25 mmol), NHC **N2** (10 mol %) and K_2_CO_3_ (0.20 mmol) in 1 mL of mesitylene at 60 °C for 12 h; isolated yields are provided; R^1^: 2-quinoxalinyl; R^2^: pyrazinyl; R^3^: phenylethyl. ^a^The structure of **3q** was determined by X-ray diffraction analysis, and other products were assigned by analogy. ^b^KHCO_3_ was used as the base.

With the optimized conditions established, we first evaluated the generality and limitations of the organocatalytic difunctionalization by examining a range of aldehydes in combination with **1a** as the constant arene component. As summarized in Fig. 3A, diverse arylethyl-substituted aldehydes proved compatible with this transformation, affording products **3a**–**3g** in 62%–90% yields. An aldehyde featuring a sulfur-containing alkyl chain provided 96% yield (**3h**). Linear aliphatic aldehydes also reacted smoothly to furnish **3i** and **3j**. Notably, acetaldehyde, which is widely recognized as a challenging substrate in NHC catalysis, was also productive under our catalytic conditions, providing **3k** in moderate yield. Cyclic alkyl aldehydes worked smoothly, delivering **3l–3n** in 60%–70% yields, and a branched secondary alkylaldehyde was also well tolerated (**3o**). Moreover, the catalytic system exhibited excellent generality with regard to aromatic aldehydes. A wide range of substituted arylaldehydes bearing either electron-donating or electron-withdrawing groups at *para*, *meta*, or *ortho* positions could smoothly undergo the transformation to afford products **3p**–**3ab** in 48%−98% yields. Disubstituted arylaldehydes were also suitable substrates, allowing efficient synthesis of **3ac**–**3ag**. Furthermore, a naphthalene-derived aldehyde participated in the reaction efficiently to give **3ah**. Heteroaromatic variants, including furyl-, thienyl-, pyridyl-, pyrazinyl- and 2-quinoxalinyl-substituted aldehydes, all proved to be well compatible, affording products **3ai**–**3an** in outstanding yields.

Next, we investigated the scope of arene substrates (Fig. 3B). To our gratification, arenes bearing either electron-donating or electron-withdrawing substituents at the *ortho* position underwent smooth difunctionalization, yielding products **3ao**–**3as** in 56%–88% yields. Notably, high site-selectivity was maintained in the reaction even using a biaryl substrate (**3at**). *Meta*-substituted arenes were also well tolerated, providing **3au**–**3ax** efficiently. In addition, arenes containing two substituents are suitable substrates (**3ay**–**3be**). It is worthy to note that even the presence of strongly directing groups (e.g. OMe) did not disturb the site-selectivity (**3az** and **3be**). Bicyclic arene substrates were also compatible, which can successfully deliver the corresponding products **3bf**–**3bh**.

**Fig. 4:**
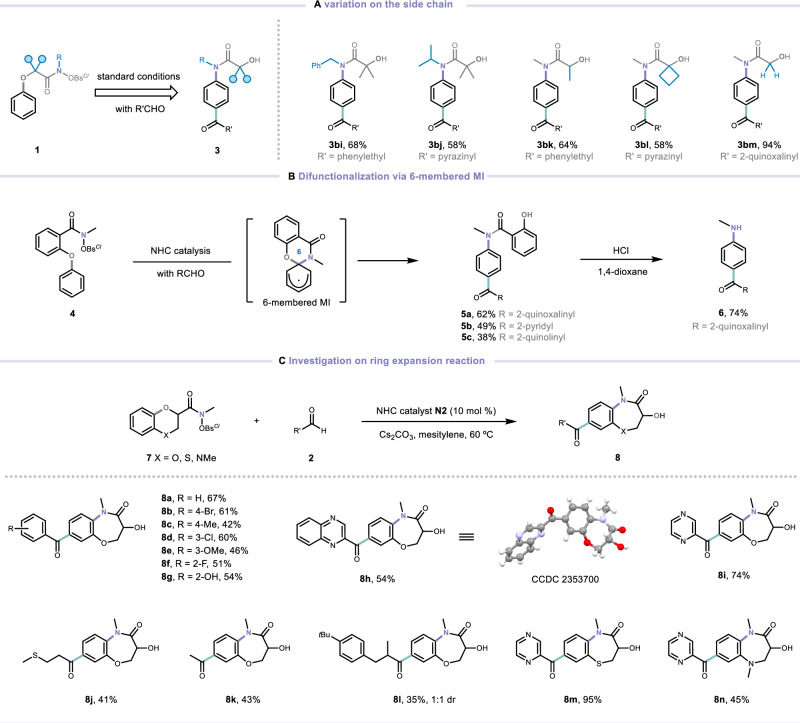
Further exploration of the substrate scope. **A** Variation on the side chain. **B** Difunctionalization via 6-membered MI. **C** Investigation on ring expansion reaction. Isolated yields are provided.

Subsequently, the O-tethered side chains were also diversified. As illustrated in Fig. 4A, both *N*-benzyl and isopropyl substrates participated smoothly to give **3bi** and **3bj**. Removing one methyl group from *gem*-dimethyl motif or replacing the linker with a cyclic framework could also successfully provide the products **3bk** and **3bl**, respectively. The unsubstituted substrate also performed exceptionally well, furnishing product **3bm** in 94% yield. When the linker was modified to a phenyl ring (**4**), the reaction was expected to undergo a six-membered intermediate. Under standard conditions, this transformation proceeded smoothly, delivering the difunctionalized products **5a**–**5c** in 38%–62% yields. Acidic hydrolysis of **5a** cleanly yielded *para*-acyl aniline **6** in 74% yield, further confirming the C–N bond formation (Fig. 4B). To our satisfaction, we found the employment of bicyclic arenes **7** in this catalytic system would facilitate a ring expansion to produce benzo[*b*]azepines **8**, which represent a privileged medium-sized *N*-heterocycles renowned for broad bioactivity^74–76^. Importantly, the ring-expansion reaction also exhibited broad tolerance toward diverse (hetero)aryl aldehydes, affording a series of acylated benzo[*b*]azepine derivatives (**8a**–**8i**). As shown in Fig. 4B, the reaction using different alkyl aldehydes, particularly for acetaldehyde, proceeded smoothly (**8j**–**8l**). Switching the linker atom from O to S could accelerate the reaction process, delivering thioether-tethered **8m** in 95% yield. Moreover, *N*-linker (**8n**) was also compatible for this reaction.

### Synthetic applications

**Fig. 5:**
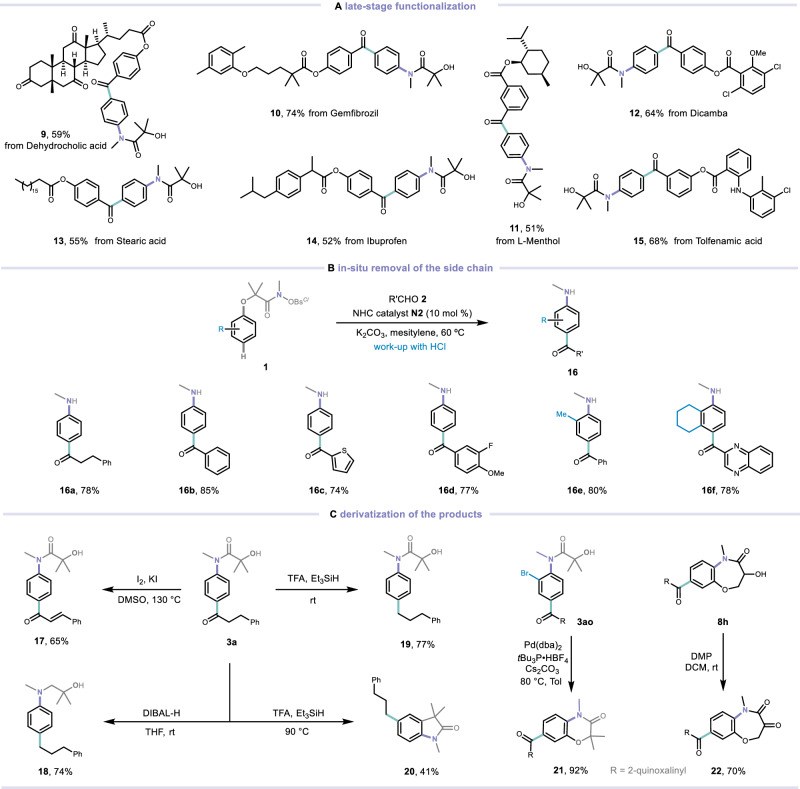
Further synthetic applications based on this method. **A** Late-stage functionalization. **B** In-situ removal of the side chain. **C** Derivatization of the products. Isolated yields are provided.

To further showcase the generality of this protocol, we applied this established NHC organocatalytic protocol to the late-stage diversification of structurally complex pharmaceutical scaffolds. As shown in Fig. 5A, aldehydes derived from dehydrocholic acid (a bile-acid modulator), gemfibrozil (a PPAR-α agonist), and L-menthol (a transient-receptor modulator) engaged efficiently under the standard conditions, furnishing drug-like scaffolds **9** (59%), **10** (74%), and **11** (51%), respectively. The aldehyde derived from dicamba underwent smoothly to give **12** (64%), while the long-chain stearic-acid-derived aldehyde delivered **13** in 55% yield. Aldehyde derivatives of ibuprofen and tolfenamic acid were well tolerated under the catalytic conditions, yielding the corresponding **14** and **15** in 52%–68% yields. To streamline access to aniline, we evaluated a one-pot deprotection sequence (Fig. 5B). Upon completion of the NHC-catalyzed difunctionalization, simple work-up with 4 M HCl in 1,4-dioxane promoted in-situ cleavage of the *N*-tethered side chain to furnish anilines **16a**–**16f** in 74%–85% overall yield.

Then, functional group diversification of the obtained products was further investigated to demonstrate the synthetic potential of this method (Fig. 5C). The α,β-desaturation of ketone **3a** was readily accomplished with I_2_/KI in DMSO at 130 °C, furnishing enone **17** in 65% yield. Reduction of both the amidyl and keto carbonyls in **3a** was achieved using DIBAL-H to deliver **18**. By contrast, chemoselective reduction of the ketone was realized under TFA/Et_3_SiH, offering **19** in 77% yield. Raising the reaction temperature to 90 °C in the same medium triggered an unexpected Brønsted-acid-promoted cyclization, and an oxindole **20** was obtained. Moreover, the *o*-Br-substituted **3ao** could undergo the Pd-catalyzed intramolecular cross-coupling reaction to form a C−O bond, affording the bicyclic **21** in 92% yield. In addition, the benzo[*b*]azepine product **8h** could be smoothly oxidized using Dess−Martin periodinane (DMP) to deliver a 1,2-dicarbonyl compound **22** in 70% yield.

### Mechanistic investigations

**Fig. 6:**
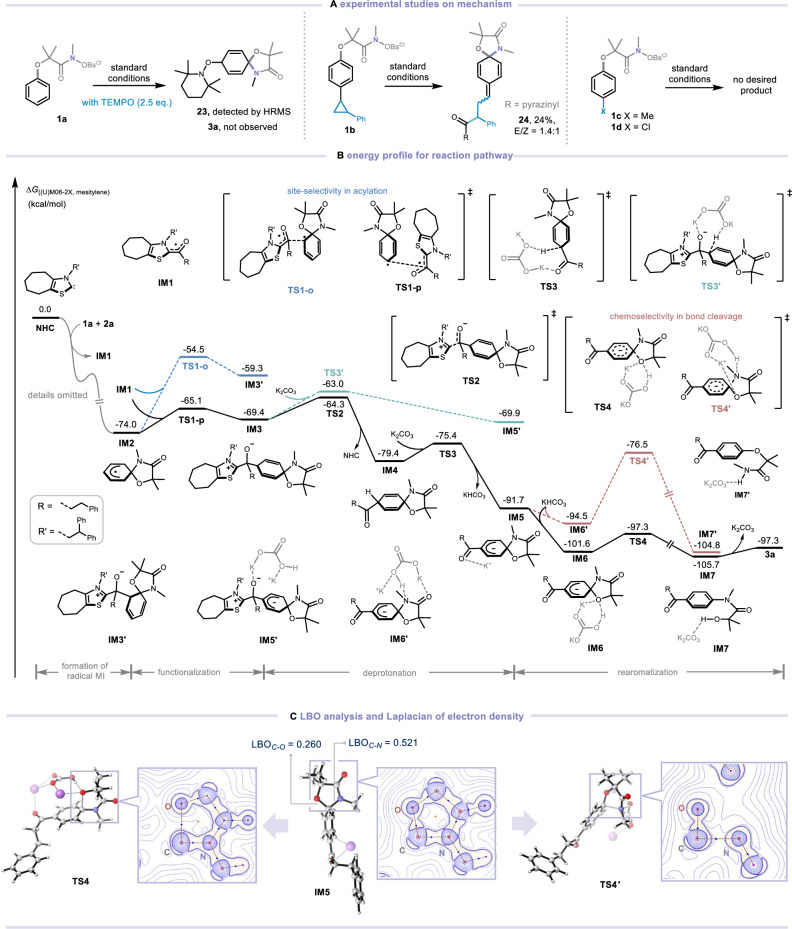
Mechanistic investigations. **A** Experimental studies on mechanism. **B** Energy profile for reaction pathway. **C** LBO analysis and Laplacian of electron density.

To investigate the mechanism of the NHC-catalyzed radical reaction, several control experiments were conducted. As shown in Fig. 6A, the catalytic conversion of **1a** was completely suppressed in the presence of 2.5 equiv of 2,2,6,6-tetramethylpiperidinyloxy (TEMPO). HRMS analysis of this reaction mixture detected the TEMPO-adducted dearomatic intermediate **23**. Moreover, a radical-clock experiment employing cyclopropane-substituted substrate **1b** led to the ring-opening product **24**. These results not only support the involvement of a radical pathway, but also provide strong evidence for the generation of Meisenheimer intermediate during the reaction process. In addition, installing a methyl or chlorine substituent at the *para* position (**1c** and **1d**) abolished reactivity, indicating that homolysis of the C−O bond is disfavored and radical capture of the Meisenheimer intermediate is essential for productive rearrangement.

We further explored the reaction mechanism by DFT calculations. Due to the heterogeneous nature of K_2_CO_3_ in mesitylene, a simplified calculation model that assumes a freely solvated carbonate anion was used (see Fig. S7 for discussion). As shown in Fig. 6B, the reaction starts with the formation of an NHC-bound radical intermediate **IM1** and a radical Meisenheimer intermediate **IM2** under NHC catalysis. The *para*-selective radical cross-coupling between **IM1** and **IM2** occurs through transition state **TS1-p**, with an energy barrier of 8.9 kcal/mol, which is significantly lower than that of the *ortho*-coupling pathway via **TS1-o** (Δ*G*^‡^ = 19.5 kcal/mol). This energy difference rationalizes the observed *para*-selectivity in the coupling step. In addition, another radical coupling pathway involving a 1,2-carbon migration process is also possible (for detailed discussion, see Fig. S3 in Supplementary Information). Following the radical acylation event, release of the NHC catalyst from **IM3** yields intermediate **IM4** (Δ*G* = –79.4 kcal/mol), which readily undergoes deprotonation via **TS3** (Δ*G*^‡^ = 4.0 kcal/mol) to form the anionic Meisenheimer intermediate **IM5**. A competing pathway involving direct deprotonation of **IM3** prior to catalyst dissociation was also considered. Its corresponding transition state (**TS3′**) lies 1.3 kcal/mol higher in free energy than **TS2**, rendering this route less favorable. Subsequent rearomatization proceeds via a concerted process involving bond cleavage and protonation. Moreover, we found that the C–O bond scission pathway (**TS4**, Δ*G* = –97.3 kcal/mol) is kinetically favored over C–N bond cleavage (**TS4′**, Δ*G* = –76.5 kcal/mol). To gain deeper insight into the chemoselectivity of C–O *v.s*. C–N bond cleavage, analysis of the Laplacian bond order (LBO) was performed (Fig. 6C). The LBO of the C–O bond in intermediate **IM5** is significantly lower than that of the C–N bond (0.260 vs. 0.521), which indicates a weaker C–O bond, thus suggesting that C–O bond cleavage is more favorable. Furthermore, we plotted the Laplacian of electron density of **IM5,** **TS4** and **TS4′** on the O–C–N plane within the spiro five-membered ring. In **TS4** (C–O bond cleavage), the 5-membered ring adopts a nearly planar topology, similar to the precursor **IM5**. In contrast, **TS4′** exhibits pronounced ring distortion. This structural difference is quantified using root mean square displacement/deviation (RMSD) of atomic positions between **IM5** and each transition state (for detailed discussion, see Fig. S5 in SI). Thus, we propose that this ring distortion in **TS4′** renders the C–N bond cleavage pathway less favorable.

## Discussion

In summary, we have developed an acyl-inserting Smiles rearrangement that enables the *ipso*/*para*-selective arene difunctionalization under NHC organocatalysis. This strategy captures the radical Meisenheimer intermediate prior to rearomatization, merging *ipso* C–O amination with *para*-selective acylation to convert aryl ethers into 4-acylated aniline derivatives. The method demonstrates broad functional group tolerance, and its synthetic utility is further underscored by a ring-expansion reaction that expedites access to biologically relevant benzo[*b*]azepines and by late-stage diversification of pharmaceutically important scaffolds. Preliminary mechanistic studies, combining experimental evidence with DFT calculations, support the proposed NHC-catalyzed radical cycle and clarify the origin of the observed site-selectivities. This work broadens the synthetic scope of NHC-radical catalysis and provides a foundation for future exploration of divergent Smiles-type rearrangements.

## Methods

### General procedure for the NHC-catalyzed difunctionalization of arenes

To an oven-dried Schlenck tube was added substrate **1** (0.10 mmol), NHC *pre*-catalyst **N2** (10 mol %), and K_2_CO_3_ (0.20 mmol). The Schlenck tube was subjected to three cycles of pressurization/depressurization using dry Ar. After that, under the protection of an Ar atmosphere, a solution of aldehydes **2** (0.25 mmol) in dry mesitylene (1 mL) was added, and the reaction mixture was stirred at 60 °C for 12 hours. Then the mixture was purified by column chromatography on silica gel to afford the corresponding difunctionalized products, which were dried under vacuum and further analyzed by ^1^H NMR, ^13^C NMR, HRMS, etc.

### General procedure for in-situ removal of the side chain

To an oven-dried Schlenck tube was added substrate **1** (0.10 mmol), NHC **N2** (10 mol %), and K_2_CO_3_ (0.20 mmol). The Schlenck tube was subjected to three cycles of pressurization/depressurization using dry Ar. After that, under the protection of an Ar atmosphere, a solution of aldehydes **2** (0.25 mmol) in dry mesitylene (1 mL) was added, and the reaction mixture was stirred at 60 °C for 12 hours. Then, to the reaction mixture was added 1.5 mL HCl solution (in 1,4-dioxane, 4 M) slowly, and further stirred at 60 °C for 5 h. The resulting mixture was diluted with water (5.0 mL) and extracted with ethyl acetate (3 × 10 mL), washed with 1 M NaHCO_3_ aq. (2 × 20 mL), and the aqueous layer was extracted with ethyl acetate (3 × 10 mL). The organic layer was combined, dried over sodium sulfate, and filtered. The filtrate was evaporated in vacuo to give a residue, which was purified by column chromatography on silica gel to afford the compound **16**. The product was finally dried under vacuum and further analyzed by ^1^H NMR, ^13^C NMR, HRMS, etc.

## Supplementary information

Supplementary Information

Transparent Peer Review file

## Source data

Source Data

## Data Availability

The data supporting the findings of this study are available within this Article and its Supplementary Information. The Cartesian coordinates are provided in the Source Data file. Crystallographic data for the structures reported in this Article have been deposited at the Cambridge Crystallographic Data Centre, under deposition numbers CCDC 2354052 (**3q**) and 2353700 (**8h**). Copies of the data can be obtained free of charge via https://www.ccdc.cam.ac.uk/structures/. Data are available from the corresponding author upon request. Source data are provided with this paper.
