# Supplementary material for: Arene difunctionalization through an acyl-inserting Smiles rearrangement enabled by N-heterocyclic carbene catalysis
Source: Nat Commun. 2026 Jun 15;17:7539. doi: 10.1038/s41467-026-74546-3 (PMC13408099; doi:10.1038/s41467-026-74546-3)
Supplement: Supplementary file 1 — Supplementary Information [file 41467_2026_74546_MOESM1_ESM.pdf]

Arene difunctionalization through an acyl-inserting Smiles rearrangement enabled by *N*-heterocyclic carbene catalysis

Qing-Zhu Li[†,#], Yan-Qing Liu[‡,#], Long-Hai Hong[†], Ting Qi[†], Mei-Hao He[†], Peng-Tao Wang[†], Xin-Xin Kou[†], and Jun-Long Li[†,*]

[†] *Anti-infective Agent Creation Engineering Research Centre of Sichuan Province, Sichuan Industrial Institute of Antibiotics, School of Pharmacy, Chengdu University, Chengdu 610106, China*

[‡] *Department of Pharmacy, the Thirteenth People's Hospital of Chongqing, Chongqing Geriatrics Hospital, Chongqing 400053, China*

[#] *These authors contributed equally to this work*

[*] *Correspondence: lijunlong709@hotmail.com (Jun-Long Li)*

Supplementary Information

Table of Contents

1. **General Information** 2
2. **Optimization Studies** 4
3. **The Preperation of Arene Substrates and NHC *pre*-Catalysts** 10
4. **The NHC-Catalyzed Difunctionalization** 41
5. **Investigations on Ring Expansion Reaction** 86
6. **Late-stage Functionalization of Complex Bioactive Molecules** 95
7. **Synthetic Applications** 100
8. **Mechanism Studies** 110
9. **Crystal Data and Structure Refinement** 115
10. **DFT computational calculation studies** 118
11. **Copies of ^{1}H, ^{13}C NMR Spectra** 137
12. **References and Notes** 291

1. General Information

General Procedures. All reactions were performed in oven-dried or flame-dried reaction vessels, modified Schlenk flasks, or round-bottom flasks. The flasks were fitted with Teflon screw caps and reactions were conducted under an atmosphere of argon if needed. Gas-tight syringes with stainless steel needles were used to transfer air- and moisture-sensitive liquids. All moisture and/or air sensitive solid compounds were manipulated inside normal desiccators. Flash column chromatography was performed over silica gel (40 – 45 μm, 300 – 400 mesh).

Analytical thin layer chromatography (TLC) was performed on silica gel $HSGF_{254}$ glass plates (purchased from Jiangyou silica gel development Co., Ltd, Yantai, China) containing a 254 nm fluorescent indicator. TLC plates were visualized by exposure to short wave ultraviolet light (254 nm) or I_2 and to a solution of $KMnO_4$ (1 g of $KMnO_4$, 6 g of K_2CO_3 and 0.1 g of KOH in 100 mL of H_2O) or vanillin (2 g of vanillin and 4 mL of concentrated H_2SO_4 in 100 mL of EtOH) followed by heating.

Organic solutions were concentrated at 30 – 40 °C on rotary evaporators at ~80 mbar followed by drying on vacuum pump below 1 mbar. Reaction temperatures are reported as the temperature of the bath surrounding the vessel unless otherwise stated.

Materials. Commercial reagents and solvents were obtained from Adamas-beta, Aldrich Chemical Co., Alfa Aesar, Leyan, Macklin and Energy Chemical and used as received. All solvents were dried and/or distilled by standard methods[1-3]. Pharmaceutical skeleton-derived aldehydes were synthesized according to the reported literature procedures[4]. NHC *pre*-catalysts **N1**[5,6], **N2**[5,6], **N3**[7], **N4**[7], **N5**[7], **N6**[8], **N7**[5,6], **N9**[7], **N11**[8], **N12**[5,6] were synthesized according to the reported literature procedures. NHC *pre*-catalysts **N8** and **N10** are commercially available.

Instrumentation.

- Proton nuclear magnetic resonance (^1H NMR) spectra were measured on a JEOL JNM-ECZ600R/S1 spectrometer at ambient temperature for ^1H at 600 MHz. Proton chemical shifts are reported in parts per million (δ scale), and are referenced using tetramethylsilane (TMS) as an internal standard or residual protium in the NMR solvent ($CDCl_3$: δ 7.26 or DMSO-d_6: δ 2.50). Data are reported as follows: chemical shift [multiplicity (s = singlet, d

= doublet, t = triplet, q = quartet, m = multiplet, dd = doublet of doublets, td = triplet of doublets, brs = broad singlet), coupling constant(s) (Hz), integration].

- Carbon-13 nuclear magnetic resonance (^{13}C NMR) spectra measured on a JEOL JNM-ECZ600R/S1 spectrometer at ambient temperature for ^{13}C at 151 MHz.. Carbon chemical shifts are reported in parts per million (δ scale), and are referenced using the carbon resonances of the solvent (δ 77.00 ($CDCl_3$) or δ 39.52 (DMSO-d_6)).

- High resolution mass spectra (HRMS) were performed on an Agilent 6230 time-of-flight (TOF) LC/MS instrument or a Waters SYNAPT G2 mass spectrometer by using an electrospray ionization (ESI) ionization source analyzed by quadrupole time-of-flight (Q-TOF). Melting points were determined on a SGW X-4 digital melting point apparatus and temperatures were not corrected.

2. Optimization Studies

Table S1. Optimization of bases[a]

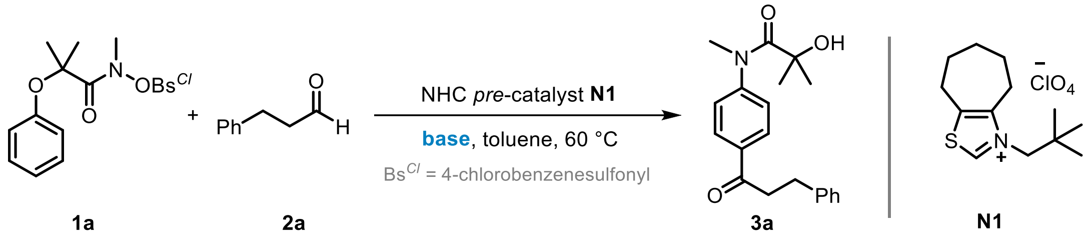

Entry	Base	Yield of **3a** (%)[b]	Entry	Base	Yield of **3a** (%)[b]
1	$KHCO_3$	64	4	K_3PO_4	62
2	Cs_2CO_3	53	5	DBU	< 5
3	K_2CO_3	69	6	DMAP	22

[a] The reactions were carried out with **1a** (0.10 mmol), **2a** (0.25 mmol), NHC **N1** (10 mol %) and base (0.2 mmol) in toluene (1.0 mL) at 60 °C for 12 hours. [b] Isolated yield of **3a**.

Table S2. Optimization of catalysts[a]

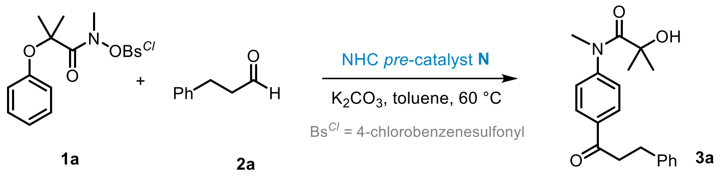

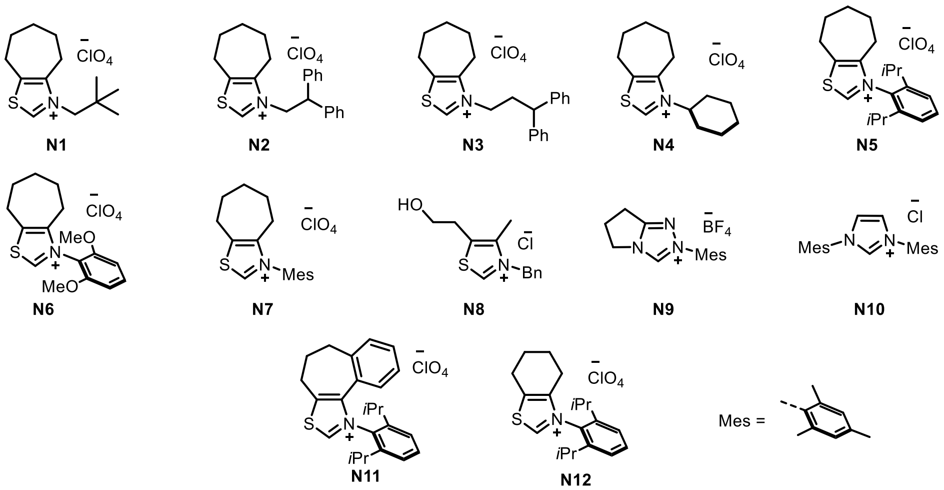

Entry	NHC	Yield of **3a** (%)[b]	Entry	NHC	Yield of **3a** (%)[b]
1	**N1**	69	7	**N7**	27
2	**N2**	73	8	**N8**	21
3	**N3**	< 5	9	**N9**	< 5
4	**N4**	< 5	10	**N10**	< 5
5	**N5**	<5	11	**N11**	< 5
6	**N6**	43	12	**N12**	< 5

[a] The reactions were carried out with **1a** (0.10 mmol), **2a** (0.25 mmol), NHC (10 mol %) and K_2CO_3 (0.2 mmol) in toluene (1.0 mL) at 60 °C for 12 hours. [b] Isolated yield of **3a**.

Table S3. Optimization of solvents[a]

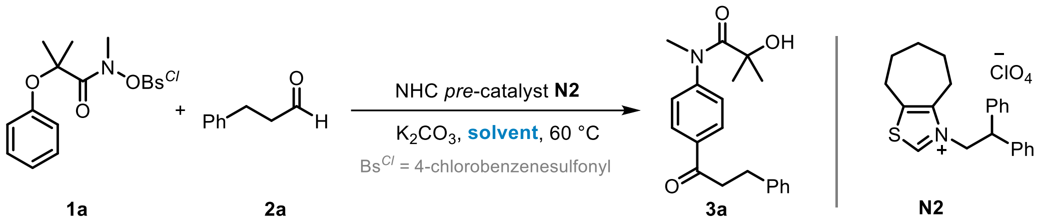

Entry	Solvent	Yield of **3a** (%)[b]
1	toluene	73
2	$PhCF_3$	52
3	*m*-xylene	77
4	mesitylene	88
5	MeCN	18
6	DCM	39
7	DMSO	< 5
8	DMF	< 5
9	MTBE	60
10	1,4-dioxane	56

[a] The reactions were carried out with **1a** (0.10 mmol), **2a** (0.25 mmol), NHC **N2** (10 mol %) and K_2CO_3 (0.2 mmol) in solvent (1.0 mL) at 60 °C for 12 hours. [b] Isolated yield of **3a**.

Table S4. Optimization of reaction temperature[a]

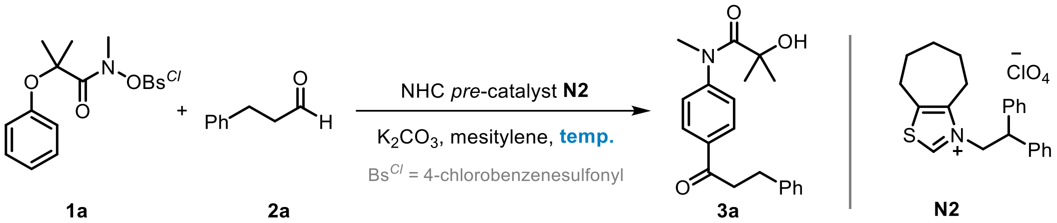

Entry	Temperature (°C)	Yield of **3a** (%)[b]
1	25	55
2	40	61
3	60	88
4	80	58

[a] The reactions were carried out with **1a** (0.10 mmol), **2a** (0.25 mmol), NHC **N2** (10 mol%) and base (0.2 mmol) in mesitylene (1.0 mL) for 12 hours. [b] Isolated yield of **3a**.

Table S5. Optimization of the *N*-leaving group[a]

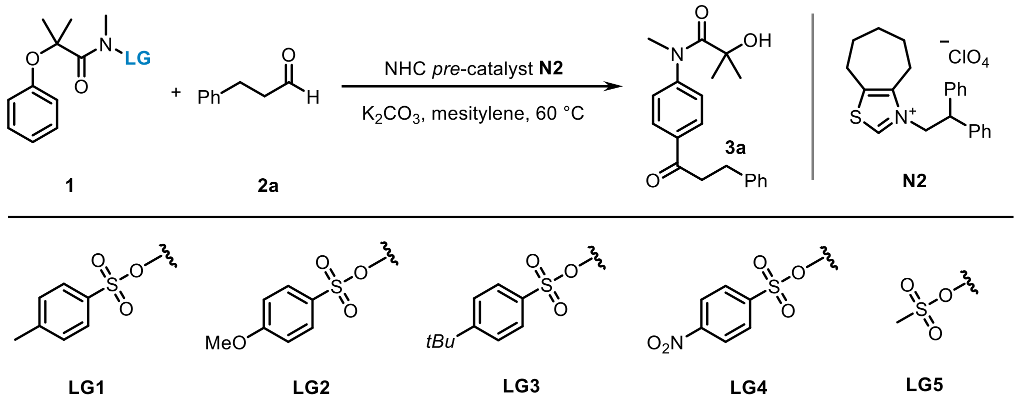

Entry	Leaving group	Yield of **3a** (%)[b]
1	**LG1**	31
2	**LG2**	30
3	**LG3**	37
4	**LG4**	< 5
5	**LG5**	38

[a] The reactions were carried out with **1** (0.10 mmol), **2a** (0.25 mmol), NHC **N2** (10 mol%) and K_2CO_3 (0.2 mmol) in solvent (1.0 mL) at 60 °C for 12 hours. [b] Isolated yield of **3a**.

Table S6. Further optimization of the *N*-leaving group[a]

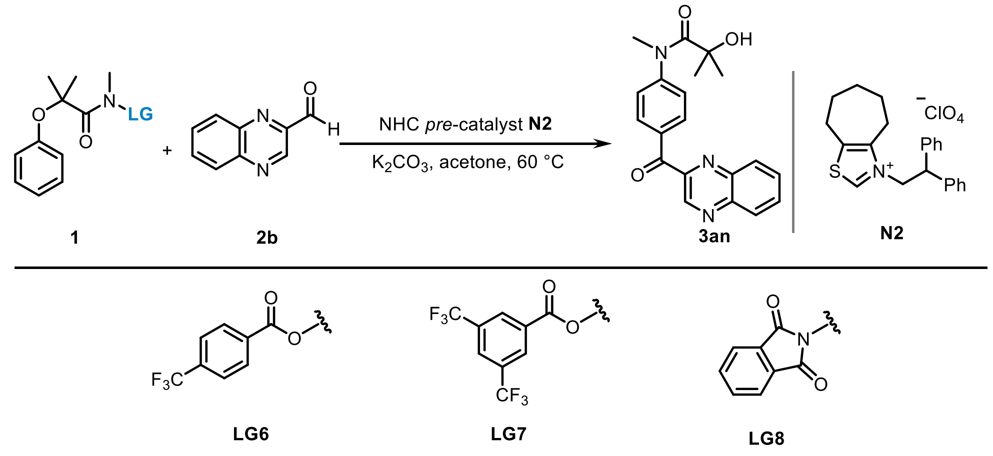

Entry	Leaving group	Yield of **3an** (%)[b]
6	**LG6**	27
7	**LG7**	37
8	**LG8**	< 5

[a] The reactions were carried out with **1** (0.10 mmol), **2b** (0.25 mmol), NHC **N2** (10 mol%) and K_2CO_3 (0.2 mmol) in solvent (1.0 mL) at 60 °C for 12 hours. [b] Isolated yield of **3an**.

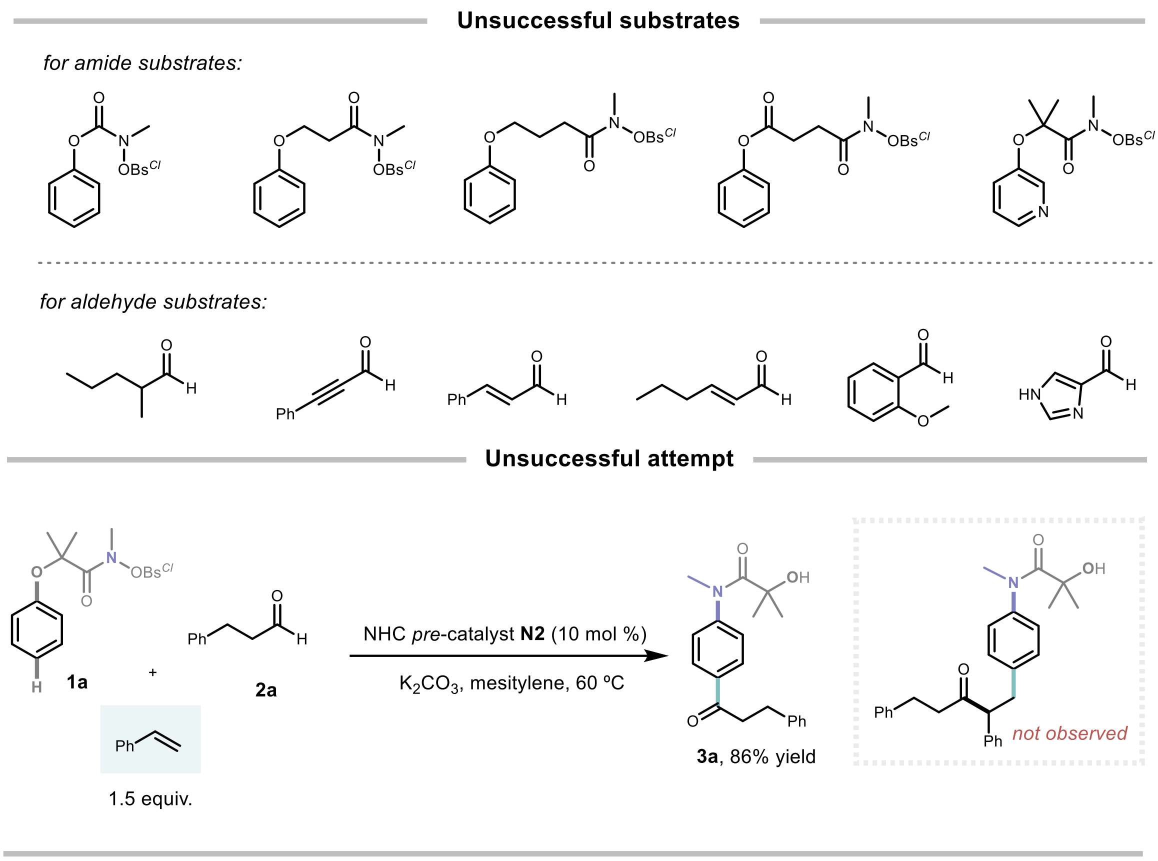

Further investigation of base solubility on reaction efficiency

To probe the effect of base solubility on reaction selectivity, we carried out a series of additional experiments. Using *n*Bu$_4$OAc in mesitylene provided a homogeneous system, in which C–O bond cleavage proceeded efficiently with high chemoselectivity (condition A). We next investigated partially heterogeneous or heterogeneous systems: potassium 2-ethylhexanoate (RCO_2K), an organic base with some solubility in THF or in a mesitylene/THF (1:1) mixture, exists as a partially dissolved suspension rather than a fully homogeneous solution. Under these conditions, exclusive formation of the C–O bond cleavage product **3a** was still observed (conditions B and C). Similarly, despite the poor solubility of Cs_2CO_3 or RCO_2K in mesitylene, the reactions consistently afforded **3a** without any detectable C–N cleavage products (conditions D and E). These experimental results collectively suggest that the solubility of the base does not play a determining role in bond cleavage selectivity. For DFT calculations on these experiments, see Figure S7 in section 10.

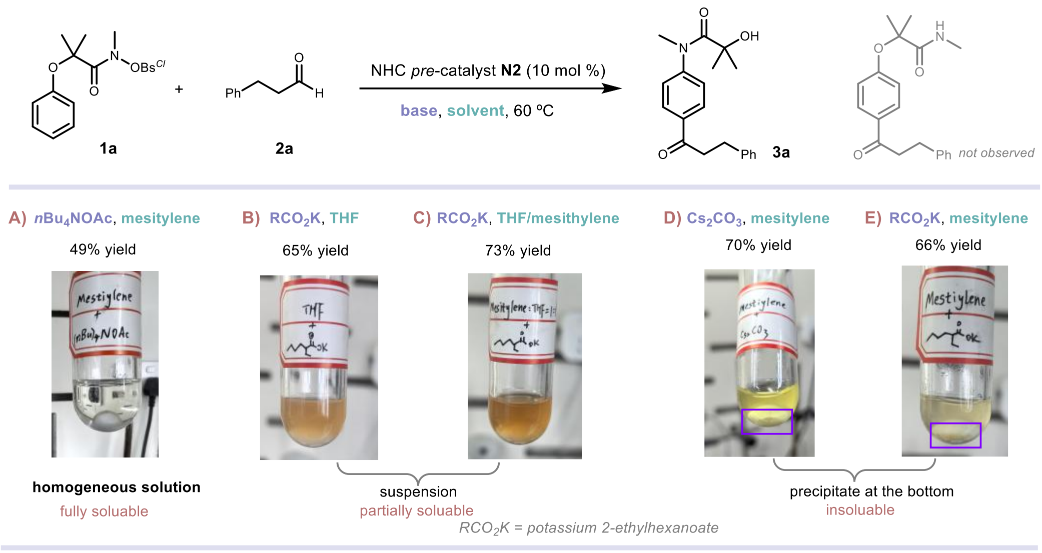

Condition-based sensitivity screening

A condition-based sensitivity screening approach was undertaken according to the report of Glorius and co-workers,[9,10] as detailed below.

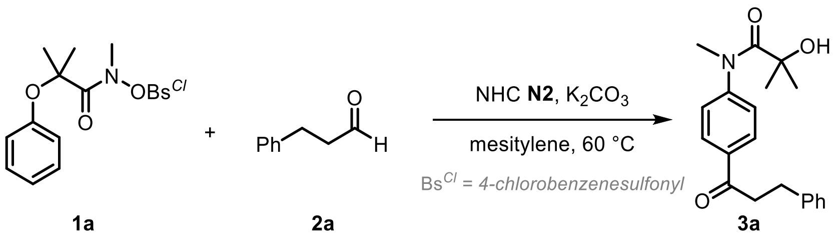

Standard conditions: n = 0.1 mmol, c = 0.1 M, V = 1 mL, inert atmosphere, T = 60 °C, **1a**: 0.1 mmol, **2a**: 0.25 mmol, NHC **N2**: 0.01 mmol, K_2CO_3: 0.2 mmol, mesitylene: 1 mL.

Stock solution ‘large scale’: n = 5.0 mmol, c = 0.1 M, V = 50.0 mL, **1a**: 5.0 mmol, **2a**: 12.5 mmol, NHC **N2**: 0.5 mmol, K_2CO_3: 10 mmol, mesitylene: 50.0 mL.

Table S7. Results of sensitivity assessment

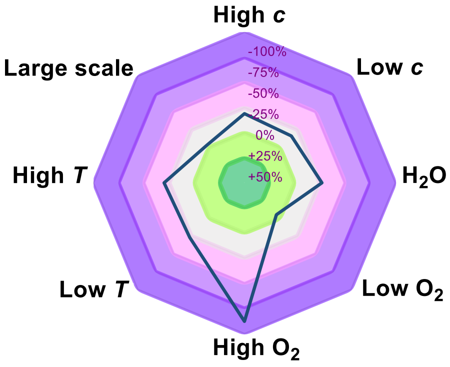

Entry	Experiment	Deviation from standard condition	Yield (%)	Deviation
1	High *c*	0.5 mL mesitylene	69	–19
2	Low *c*	1.5 mL mesitylene	72	–16
3	High H_2O	10 uL H_2O	61	–27
4[a]	Low O_2	Degassed for 3 times	93	+5
5	High O_2	air	0	–88
6	Low *T*	T = 40 °C	61	–27
7	High *T*	T = 80 °C	58	–30
8	Standard	1.0 mL mesitylene	88	0
9	Large scale	50 mL stock solution 'large scale'	85	–3

[a] Under a fully argon atmosphere.

3. The Preperation of Arene Substrates and NHC *pre*-Catalysts

The direct synthesis of ***1a*** *from phenol*

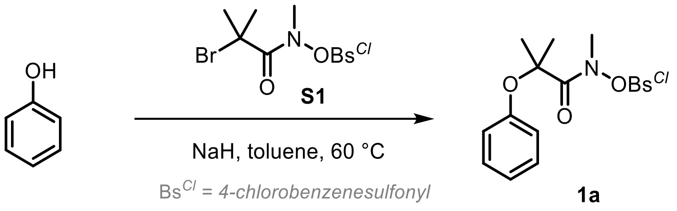

To an oven-dried Schlenck tube was added phenol (1.0 equiv.), then the Schlenck tube was subjected to three cycles of pressurization/depressurization using dry Ar. After that, under the protection of Ar atmosphere, toluene (0.2 M) and NaH (1.5 equiv.) were added successively, then the mixture was stirred for 5 minutes. Subsequently, bromide **S1** was added, and the reaction mixture was stirred for 10 h. After the reaction finished, the reaction mixture was diluted with water and extracted with dichloromethane. The combined organic phases were washed with brine, dried over anhydrous Na_2SO_4, filtered and concentrated in vacuum. The crude residue was then purified by column chromatography on silica gel eluting with petroleum ether/ethyl acetate (5:1 to 1:1) to provide the substrate **1a** in 23% yield.

General Procedure A: *the preperation of arene substrates*

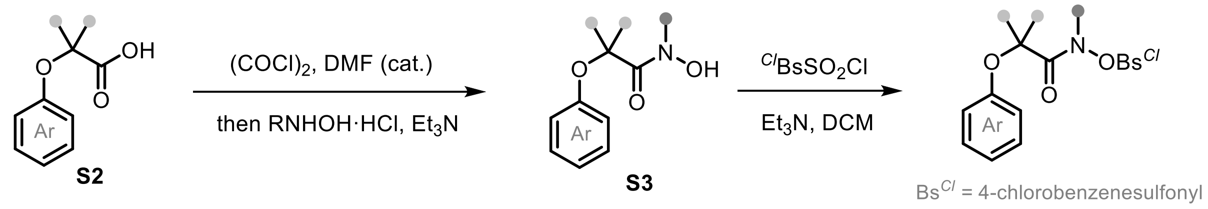

To a solution of acid **S2** (1.0 equiv.) and DMF (0.2 equiv.) in DCM (1 M) was added oxalyl chloride (1.5 equiv.) at 0 °C slowly. The reaction mixture was allowed to stir at room temperature for 2–4 h, then it was concentrated to afford the acyl chloride that was directly used in the next reaction. RNHOH·HCl (1.0 equiv.) was added to a solution of Et_3N (2.4 equiv.) in DCM (1 M) at 0 °C, followed by dropwise addition of a DCM solution of the acyl chloride. Then, the reaction was warmed to room temperature for stirring additional 2–4 h. After completion, the reaction mixture was diluted with water and extracted by DCM. The combined

organic phases were washed with brine, dried over anhydrous Na_2SO_4, filtered and concentrated in vacuum to afford the **S3** that was directly used without further purification.

To a solution of the **S3** (1.0 equiv.) and triethylamine (1.2 equiv.) in DCM (1 M) was dropwise added 4-chlorobenzenesulfonyl chloride (1.1 equiv.) over 5 min at 0 °C. Then the resulting mixture was stirred for 3−12 h at room temperature. After the reaction finished, the reaction mixture was diluted with water and extracted by dichloromethane. The combined organic phases were washed with brine, dried over anhydrous Na_2SO_4, filtered and concentrated in vacuum. The crude residue was then purified by column chromatography on silica gel eluting from petroleum ether/ethyl acetate to provide the substrates **1**,**4** or **7**, which were dried under vacuum and further analyzed by ^{1}H NMR, ^{13}C NMR, HRMS, etc.

Characterization of representative examples of arene substrates:

***N*-(((4-chlorophenyl)sulfonyl)oxy)-*N*,2-dimethyl-2-phenoxypropanamide 1a**

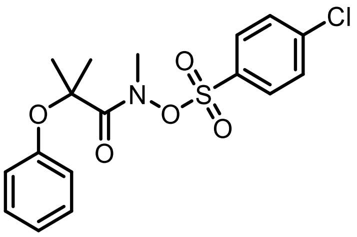

Prepared according to the ***General Procedure A*** by using corresponding **S2** (3.42 g) to afford 2.8 g of **1a** in 38% yield as white solid, m.p. = 102.1 – 105.1 ºC.

*NMR and HRMS data for the substrate **1a***:

^{1}H NMR (600 MHz, $CDCl_3$) δ (ppm): 7.89 (d, J = 9.0 Hz, 2H), 7.50 (d, J = 9.0 Hz, 2H), 7.27 (t, J = 7.8 Hz, 2H), 7.04 (t, J = 7.8 Hz, 1H), 6.81 (d, J = 7.8 Hz, 2H), 3.60 (s, 3H), 1.52 (s, 6H).

^{13}C NMR (151 MHz, $CDCl_3$) δ (ppm): 173.3, 154.4, 141.6, 133.2, 130.7, 129.5, 129.3, 122.6, 118.5, 80.0, 41.9, 25.5.

HRMS (ESI-TOF) *m/z*: $[M + Na]^+$ Calcd for $C_{17}H_{18}{}^{35}ClNO_5SNa^+$ 406.0487, $C_{17}H_{18}{}^{37}ClNO_5SNa^+$ 408.0457; Found 406.0490, 408.0459.

***N*-(((4-chlorophenyl)sulfonyl)oxy)-*N*,2-dimethyl-2-(4-(2-phenylcyclopropyl)phenoxy)propanamide 1b**

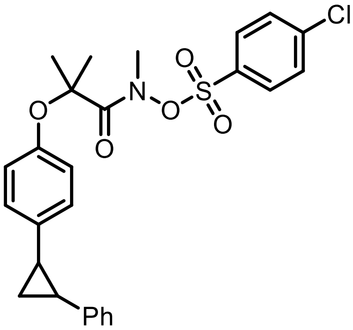

Prepared according to the ***General Procedure A*** by using corresponding **S2** (1.48 g) to afford 0.50 g of **1b** in 50% yield as white solid, m.p. = 99.1 – 102.8 ºC.

*NMR and HRMS data for the substrate **1b***:

^{1}H NMR (600 MHz, $CDCl_3$) δ (ppm): 7.91 (d, *J* = 9.0 Hz, 2H), 7.50 (d, *J* = 8.4 Hz, 2H), 7.29 (t, *J* = 7.8 Hz, 2H), 7.18 (t, *J* = 7.8 Hz, 1H), 7.13 (d, *J* = 8.4 Hz, 2H), 7.03 (d, *J* = 8.4 Hz, 2H), 6.74 (d, *J* = 8.4 Hz, 2H), 3.63 (s, 3H), 2.14 – 2.09 (m, 2H), 1.49 (s, 6H), 1.44 – 1.41 (m, 1H), 1.40 – 1.37 (m, 1H).

^{13}C NMR (151 MHz, $CDCl_3$) δ (ppm): 173.4, 152.5, 142.4, 141.6, 136.9, 133.2, 130.7, 129.3, 128.4, 126.8, 125.8, 125.7, 118.7, 80.1, 41.9, 27.8, 27.3, 25.5, 18.1.

HRMS (ESI-TOF) *m/z*: [M + Na]$^+$ Calcd for $C_{26}H_{26}{}^{35}ClNO_5SNa^+$ 522.1112, $C_{26}H_{26}{}^{37}ClNO_5SNa^+$ 524.1083; Found 522.1116, 524.1090.

***N*-(((4-chlorophenyl)sulfonyl)oxy)-*N*,2-dimethyl-2-(p-tolyloxy)propanamide 1c**

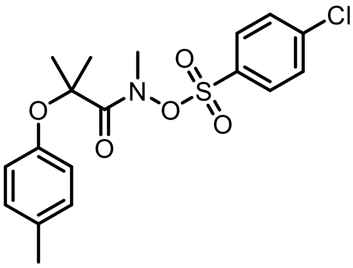

Prepared according to the ***General Procedure A*** by using corresponding **S2** (2.25 g) to afford 0.77 g of **1c** in 17% yield as white solid, m.p. = 102.3 – 103.5 ºC.

*NMR and HRMS data for the substrate **1c***:

^{1}H NMR (600 MHz, CDCl$_3$) δ (ppm): 7.91 (d, J = 8.4 Hz, 2H), 7.50 (d, J = 8.4 Hz, 2H), 7.05 (d, J = 9.0 Hz, 2H), 6.70 (d, J = 9.0 Hz, 2H), 3.63 (s, 3H), 2.30 (s, 3H), 1.48 (s, 6H).

^{13}C NMR (151 MHz, CDCl$_3$) δ (ppm): 173.4, 152.2, 141.5, 133.2, 132.2, 130.7, 130.0, 129.3, 118.6, 80.0, 41.9, 25.5, 20.5.

HRMS (ESI-TOF) *m/z*: [M + Na]$^+$ Calcd for $C_{18}H_{20}{}^{35}ClNO_5SNa^+$ 420.0643, $C_{18}H_{20}{}^{37}ClNO_5SNa^+$ 422.0614; Found 420.0641, 422.0616.

2-(4-chlorophenoxy)-*N*-(((4-chlorophenyl)sulfonyl)oxy)-*N*,2-dimethylpropanamide 1d

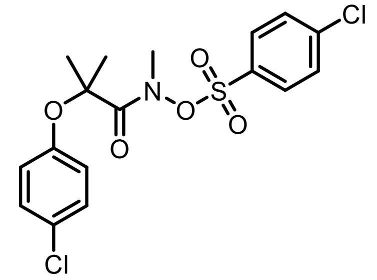

Prepared according to the ***General Procedure A*** by using corresponding **S2** (2.32 g) to afford 0.92 g of **1d** in 20% yield as white solid, m.p. = 106.8 – 108.6 ºC.

*NMR and HRMS data for the substrate **1d***:

^{1}H NMR (600 MHz, CDCl$_3$) δ (ppm): 7.90 (d, J = 7.8 Hz, 2H), 7.51 (d, J = 8.4 Hz, 2H), 7.22 (d, J = 9.0 Hz, 2H), 6.76 (d, J = 9.0 Hz, 2H), 3.58 (s, 3H), 1.51 (s, 6H).

^{13}C NMR (151 MHz, CDCl$_3$) δ (ppm): 172.9, 153.0, 141.7, 133.2, 130.6, 129.5, 129.4, 127.8, 119.8, 80.4, 41.8, 25.4.

HRMS (ESI-TOF) *m/z*: [M + Na]$^+$ Calcd for $C_{17}H_{17}{}^{35}Cl{}^{35}ClNO_5SNa^+$ 440.0097, $C_{17}H_{17}{}^{37}Cl{}^{35}ClNO_5SNa^+$ 442.0068; Found 440.0096, 442.0069.

***N*-(((4-chlorophenyl)sulfonyl)oxy)-*N*,2-dimethyl-2-(o-tolyloxy)propanamide 1e**

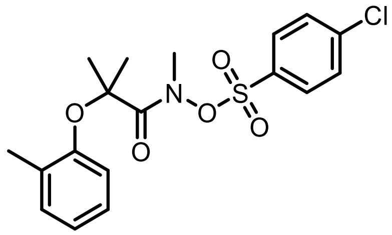

Prepared according to the ***General Procedure A*** by using corresponding **S2** (2.02 g) to afford 1.71 g of **1e** in 42% yield as white solid, m.p. = 84.7 – 87.4 ºC.

*NMR and HRMS data for the substrate **1e***:

^{1}H NMR (600 MHz, $CDCl_3$) δ (ppm): 7.92 (d, *J* = 8.4 Hz, 2H), 7.51 (d, *J* = 8.4 Hz, 2H), 7.16 (d, *J* = 7.2 Hz, 1H), 7.08 (t, *J* = 7.8 Hz, 1H), 6.93 (t, *J* = 7.2 Hz, 1H), 6.65 (d, *J* = 8.4 Hz, 1H), 3.60 (s, 3H), 2.18 (s, 3H), 1.52 (s, 6H).

^{13}C NMR (151 MHz, $CDCl_3$) δ (ppm): 173.5, 152.7, 141.6, 133.3, 131.2, 130.7, 129.3, 128.9, 126.7, 122.3, 116.1, 79.9, 41.8, 25.6, 16.6.

HRMS (ESI-TOF) *m/z*: $[M + Na]^+$ Calcd for $C_{18}H_{20}{}^{35}ClNO_5SNa^+$ 420.0643, $C_{18}H_{20}{}^{37}ClNO_5SNa^+$ 422.0614; Found 420.0637, 422.0614.

***N*-(((4-chlorophenyl)sulfonyl)oxy)-2-(2-methoxyphenoxy)-*N*,2-dimethylpropanamide 1f**

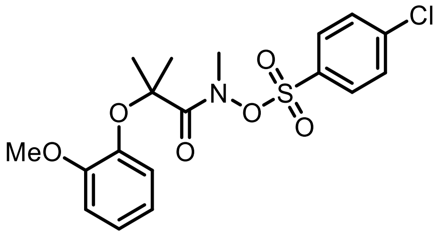

Prepared according to the ***General Procedure A*** by using corresponding **S2** (2.25 g) to afford 2.21 g of **1f** in 43% yield as white solid, m.p. = 66.2 – 68.2 ºC.

*NMR and HRMS data for the substrate **1f***:

^{1}H NMR (600 MHz, $CDCl_3$) δ (ppm): 7.95 (d, *J* = 9.0 Hz, 2H), 7.50 (d, *J* = 9.0 Hz, 2H), 7.04 (t, *J* = 8.4 Hz, 1H), 6.86 – 6.82 (m, 3H), 3.75 (s, 3H), 3.73 (s, 3H), 1.34 (s, 6H).

^{13}C NMR (151 MHz, $CDCl_3$) δ (ppm): 173.3, 152.0, 143.2, 141.4, 133.0, 130.9, 129.1, 124.5, 122.3, 120.7, 111.9, 81.3, 55.2, 42.7, 25.6.

HRMS (ESI-TOF) *m/z*: $[M + Na]^+$ Calcd for $C_{18}H_{20}{}^{35}ClNO_6SNa^+$ 436.0593, $C_{18}H_{20}{}^{37}ClNO_6SNa^+$ 438.0563; Found 436.0596, 438.0561.

2-([1,1'-biphenyl]-2-yloxy)-*N*-(((4-chlorophenyl)sulfonyl)oxy)-*N*,2-dimethylpropanamide 1g

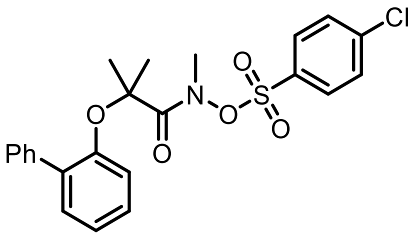

Prepared according to the ***General Procedure A*** by using corresponding **S2** (1.26 g) to afford 0.34 g of **1g** in 15% yield as white solid, m.p. = 100.1 – 103.2 ºC.

*NMR and HRMS data for the substrate **1g***:

^{1}H NMR (600 MHz, CDCl$_3$) δ (ppm): 7.90 (d, *J* = 8.4 Hz, 2H), 7.50 (d, *J* = 8.4 Hz, 2H), 7.43 (d, *J* = 7.8 Hz, 2H), 7.40 (t, *J* = 7.2 Hz, 2H), 7.36 – 7.31 (m, 2H), 7.25 (t, *J* = 7.8 Hz, 1H), 7.13 (t, *J* = 7.8 Hz, 1H), 6.84 (d, *J* = 7.8 Hz, 1H), 3.62 (s, 3H), 1.36 (s, 6H).

^{13}C NMR (151 MHz, CDCl$_3$) δ (ppm): 173.4, 151.3, 141.6, 138.4, 134.0, 133.2, 131.3, 130.7, 129.6, 129.3, 128.4, 127.9, 127.0, 123.1, 117.9, 80.7, 42.0, 25.3.

HRMS (ESI-TOF) *m/z*: [M + H]$^+$ Calcd for $C_{23}H_{23}{}^{35}ClNO_5S^+$ 460.0980, $C_{23}H_{23}{}^{37}ClNO_5S^+$ 462.0951; Found 460.0982, 462.0957.

2-(2-bromophenoxy)-*N*-(((4-chlorophenyl)sulfonyl)oxy)-*N*,2-dimethylpropanamide 1h

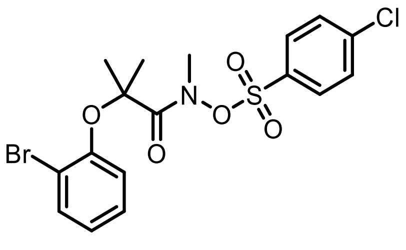

Prepared according to the ***General Procedure A*** by using corresponding **S2** (2.40 g) to afford 1.1 g of **1h** in 26% yield as white solid, m.p. = 82.1 – 83.4 ºC.

*NMR and HRMS data for the substrate **1h***:

^{1}H NMR (600 MHz, CDCl$_3$) δ (ppm): 7.91 (d, *J* = 8.4 Hz, 2H), 7.56 (d, *J* = 7.8 Hz, 1H), 7.51 (d, *J* = 8.4 Hz, 2H), 7.21 (t, *J* = 7.8 Hz, 1H), 6.93 (t, *J* = 7.8 Hz, 1H), 6.84 (d, *J* = 8.4 Hz, 1H), 3.63 (s, 3H), 1.56 (s, 6H).

^{13}C NMR (151 MHz, CDCl$_3$) δ (ppm): 172.9, 151.3, 141.7, 133.7, 133.2, 130.7, 129.4, 128.4, 124.0, 118.6, 115.4, 81.5, 42.0, 25.3.

HRMS (ESI-TOF) *m/z*: [M + Na]$^+$ Calcd for $C_{17}H_{17}{}^{79}Br^{35}ClNO_5SNa^+$ 483.9592, $C_{17}H_{17}{}^{81}Br^{35}ClNO_5SNa^+$ 485.9572, $C_{17}H_{17}{}^{81}Br^{37}ClNO_5SNa^+$ 487.9542; Found 483.9595, 485.9576, 487.9546.

***N*-(((4-chlorophenyl)sulfonyl)oxy)-2-(2-iodophenoxy)-*N*,2-dimethylpropanamide 1i**

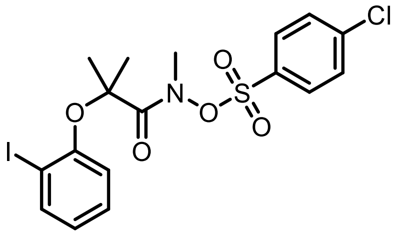

Prepared according to the ***General Procedure A*** by using corresponding **S2** (2.29 g) to afford 2.0 g of **1i** in 52% yield as white solid, m.p. = 99.2 – 102.3 °C.

*NMR and HRMS data for the substrate **1i***:

^{1}H NMR (600 MHz, CDCl$_3$) δ (ppm): 7.91 (d, J = 9.0 Hz, 2H), 7.79 (d, J = 7.2 Hz, 1H), 7.51 (d, J = 8.4 Hz, 2H), 7.24 (t, J = 7.2 Hz, 1H), 6.79 (t, J = 7.8 Hz, 1H), 6.76 (d, J = 8.4 Hz, 1H), 3.61 (s, 3H), 1.59 (s, 6H).

^{13}C NMR (151 MHz, CDCl$_3$) δ (ppm): 172.9, 153.7, 141.7, 139.8, 133.2, 130.7, 129.4, 124.5, 117.1, 90.2, 81.5, 42.0, 25.4.

HRMS (ESI-TOF) *m/z*: [M + Na]$^+$ Calcd for $C_{17}H_{17}I^{35}ClNO_5SNa^+$ 531.9453, $C_{17}H_{17}I^{37}ClNO_5SNa^+$ 533.9424; Found 531.9461, 533.9428.

***N*-(((4-chlorophenyl)sulfonyl)oxy)-*N*,2-dimethyl-2-(2-(trifluoromethyl)phenoxy)propanamide 1j**

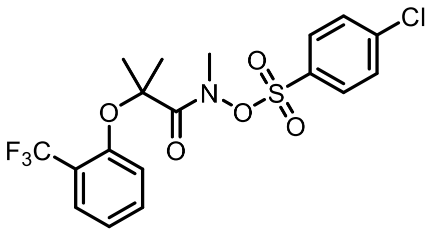

Prepared according to the ***General Procedure A*** by using corresponding **S2** (0.48 g) to afford 0.42 g of **1j** in 52% yield as white solid, m.p. = 83.5 – 85.7 °C.

NMR and HRMS data for the substrate ***1j***:

^{1}H NMR (600 MHz, CDCl3) δ (ppm): 7.91 (d, J = 7.8 Hz, 2H), 7.60 (d, J = 7.2 Hz, 1H), 7.52 (d, J = 8.4 Hz, 2H), 7.44 (t, J = 7.8 Hz, 1H), 7.10 (t, J = 7.8 Hz, 1H), 6.86 (d, J = 8.4 Hz, 1H), 3.57 (s, 3H), 1.58 (s, 6H).

^{13}C NMR (151 MHz, CDCl3) δ (ppm): 172.8, 152.4, 141.8, 133.1, 133.0, 130.6, 129.4, 127.4 (q, J = 4.8 Hz), 123.4 (q, J = 273.3 Hz), 121.8, 121.2 (q, J = 30.4 Hz), 117.2, 80.7, 41.7, 25.1.

^{19}F NMR (564 MHz, CDCl3) δ (ppm): -61.97 (s, 1F).

HRMS (ESI-TOF) *m/z*: $[M + Na]^+$ Calcd for $C_{18}H_{17}{}^{35}ClF_3NO_5SNa^+$ 474.0360, $C_{18}H_{17}{}^{37}ClF_3NO_5SNa^+$ 476.0331; Found 474.0363, 476.0339.

***N*-(((4-chlorophenyl)sulfonyl)oxy)-*N*,2-dimethyl-2-(m-tolyloxy)propanamide 1k**

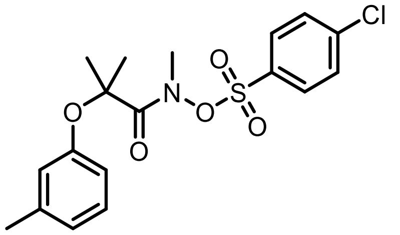

Prepared according to the ***General Procedure A*** by using corresponding **S2** (2.31 g) to afford 2.0 g of **1k** in 42% yield as white solid, m.p. = 95.9 – 96.8 °C.

NMR and HRMS data for the substrate ***1k***:

^{1}H NMR (600 MHz, CDCl3) δ (ppm): 7.88 (d, J = 9.0 Hz, 2H), 7.49 (d, J = 8.4 Hz, 2H), 7.14 (t, J = 7.8 Hz, 1H), 6.86 (d, J = 7.8 Hz, 1H), 6.70 (s, 1H), 6.60 (d, J = 8.4 Hz, 1H), 3.58 (s, 3H), 2.32 (s, 3H), 1.51 (s, 6H).

^{13}C NMR (151 MHz, CDCl3) δ (ppm): 173.4, 154.4, 141.6, 139.8, 133.3, 130.7, 129.3, 129.2, 123.4, 119.3, 115.2, 79.9, 41.9, 25.6, 21.4.

HRMS (ESI-TOF) *m/z*: $[M + Na]^+$ Calcd for $C_{18}H_{20}{}^{35}ClNO_5SNa^+$ 420.0643, $C_{18}H_{20}{}^{37}ClNO_5SNa^+$ 422.0614; Found 420.0647, 422.0619.

N-(((4-chlorophenyl)sulfonyl)oxy)-2-(3-fluorophenoxy)-N,2-dimethylpropanamide 1l

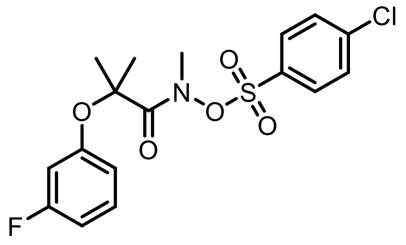

Prepared according to the ***General Procedure A*** by using corresponding **S2** (2.57 g) to afford 1.70 g of **1l** in 33% yield as white solid, m.p. = 112.5 – 114.8 ºC.

*NMR and HRMS data for the substrate **1l***:

^{1}H NMR (600 MHz, CDCl$_3$) δ (ppm): 7.91 (d, J = 9.0 Hz, 2H), 7.52 (d, J = 8.4 Hz, 2H), 7.21 (td, J = 8.4, 6.6 Hz, 1H), 6.76 (td, J = 8.4, 3.0 Hz, 1H), 6.60 (dd, J = 8.4, 3.0 Hz, 1H), 6.56 (dt, J = 10.2, 2.4 Hz, 1H), 3.59 (s, 3H), 1.53 (s, 6H).

^{13}C NMR (151 MHz, CDCl$_3$) δ (ppm): 172.7, 163.3 (d, J = 247.3 Hz), 155.7 (d, J = 10.1 Hz), 141.7, 133.2, 130.7, 130.3 (d, J = 10.1 Hz), 129.4, 113.9, 109.6 (d, J = 21.7 Hz), 106.4 (d, J = 24.6 Hz), 80.5, 41.8, 25.4.

^{19}F NMR (564 MHz, CDCl$_3$) δ (ppm): -110.73 – -110.78 (m, 1F).

HRMS (ESI-TOF) *m/z*: $[M + Na]^+$ Calcd for $C_{17}H_{17}{}^{35}ClFNO_5SNa^+$ 424.0393, $C_{17}H_{17}{}^{37}ClFNO_5SNa^+$ 426.0363; Found 424.0388, 426.0361.

2-(3-bromophenoxy)-N-(((4-chlorophenyl)sulfonyl)oxy)-N,2-dimethylpropanamide 1m

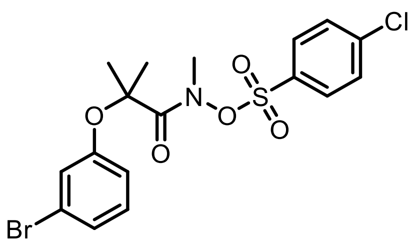

Prepared according to the ***General Procedure A*** by using corresponding **S2** (2.10 g) to afford 2.24 g of **1m** in 60% yield as white solid, m.p. = 112.5 – 114.9 ºC.

*NMR and HRMS data for the substrate **1m***:

^{1}H NMR (600 MHz, CDCl$_3$) δ (ppm): 7.91 (d, J = 9.0 Hz, 2H), 7.51 (d, J = 7.8 Hz, 2H), 7.19 (d, J = 9.0 Hz, 1H), 7.13 (t, J = 7.8 Hz, 1H), 7.06 (s, 1H), 6.75 (d, J = 8.4 Hz, 1H), 3.57 (s, 3H), 1.52 (s, 6H).

^{13}C NMR (151 MHz, CDCl$_3$) δ (ppm): 172.8, 155.2, 141.7, 133.3, 130.63, 130.58, 129.4, 125.9, 122.8, 122.3, 116.9, 80.6, 41.9, 25.5.

HRMS (ESI-TOF) *m/z*: [M + H]$^+$ Calcd for $C_{17}H_{18}{}^{79}Br^{35}ClFNO_5S^+$ 461.9772, $C_{17}H_{18}{}^{81}Br^{35}ClFNO_5S^+$ 463.9752, $C_{17}H_{18}{}^{81}Br^{37}ClFNO_5S^+$ 465.9722; Found 461.9768, 463.9748, 465.9723.

<u>*N*-(((4-chlorophenyl)sulfonyl)oxy)-2-(3-cyanophenoxy)-*N*,2-dimethylpropanamide 1n</u>

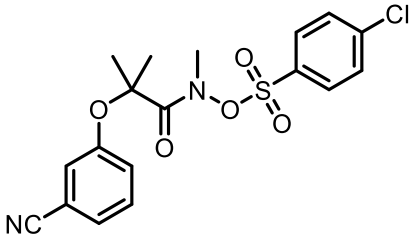

Prepared according to the ***General Procedure A*** by using corresponding **S2** (0.93 g) to afford 0.24 g of **1n** in 13% yield as white solid, m.p. = 102.1 – 103.3 °C.

*NMR and HRMS data for the substrate **1n***:

^{1}H NMR (600 MHz, CDCl$_3$) δ (ppm): 7.92 (d, J = 8.4 Hz, 2H), 7.54 (d, J = 8.4 Hz, 2H), 7.38 (t, J = 7.8 Hz, 1H), 7.34 (d, J = 7.2 Hz, 1H), 7.14 (s, 1H), 7.08 (d, J = 8.4 Hz, 1H), 3.58 (s, 3H), 1.56 (s, 6H).

^{13}C NMR (151 MHz, CDCl$_3$) δ (ppm): 172.2, 154.7, 141.8, 133.2, 130.5, 129.5, 126.3, 122.8, 122.1, 118.1, 113.6, 80.8, 41.8, 25.3.

HRMS (ESI-TOF) *m/z*: [M + Na]$^+$ Calcd for $C_{18}H_{17}{}^{35}ClN_2O_5SNa^+$ 431.0439, $C_{18}H_{17}{}^{37}ClN_2O_5SNa^+$ 433.0410; Found 431.0438, 433.0403.

2-(5-chloro-2-methylphenoxy)-*N*-(((4-chlorophenyl)sulfonyl)oxy)-*N*,2-dimethylpropanamide 1o

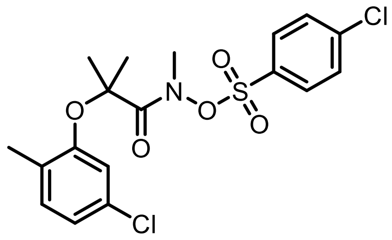

Prepared according to the ***General Procedure A*** by using corresponding **S2** (0.91 g) to afford 0.45 g of **1o** in 26% yield as white solid, m.p. = 138.1 – 140.3 ºC.

*NMR and HRMS data for the substrate **1o***:

^{1}H NMR (600 MHz, CDCl$_3$) δ (ppm): 7.96 (d, *J* = 8.4 Hz, 2H), 7.52 (d, *J* = 7.8 Hz, 2H), 7.08 (d, *J* = 8.4 Hz, 1H), 6.95 (d, *J* = 8.4 Hz, 1H), 6.79 (s, 1H), 3.58 (s, 3H), 2.14 (s, 3H), 1.52 (s, 6H).

^{13}C NMR (151 MHz, CDCl$_3$) δ (ppm): 173.0, 153.1, 141.6, 133.4, 131.74, 131.69, 130.6, 129.5, 127.8, 122.7, 117.0, 80.6, 41.9, 25.5, 16.3.

HRMS (ESI-TOF) *m/z*: [M + Na]$^+$ Calcd for $C_{18}H_{19}{}^{35}Cl^{35}ClNO_5SNa^+$ 454.0253, $C_{18}H_{19}{}^{37}Cl^{35}ClNO_5SNa^+$ 454.0253, $C_{18}H_{19}{}^{37}Cl^{37}ClNO_5SNa^+$ 458.0194; Found 454.0250, 456.0222, 458.0200.

***N*-(((4-chlorophenyl)sulfonyl)oxy)-2-(3-fluoro-5-methylphenoxy)-*N*,2-dimethylpropanamide 1p**

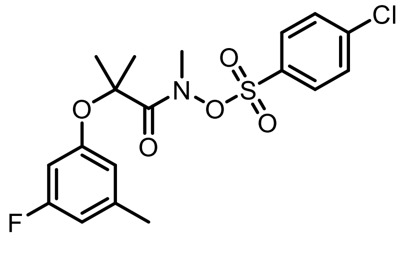

Prepared according to the ***General Procedure A*** by using corresponding **S2** (0.91 g) to afford 0.40 g of **1p** in 22% yield as white solid, m.p. = 102.1 – 104.2 ºC.

*NMR and HRMS data for the substrate **1p***:

^{1}H NMR (600 MHz, CDCl$_3$) δ (ppm): 7.89 (d, J = 8.4 Hz, 2H), 7.51 (d, J = 8.4 Hz, 2H), 6.58 (d, J = 9.0 Hz, 1H), 6.49 (s, 1H), 6.35 (d, J = 9.6 Hz, 1H), 3.57 (s, 3H), 2.31 (s, 3H), 1.52 (s, 6H).

^{13}C NMR (151 MHz, CDCl$_3$) δ (ppm): 172.8, 163.1 (d, J = 245.8 Hz), 155.3 (d, J = 11.5 Hz), 141.6, 141.2 (d, J = 8.6 Hz), 133.2, 130.6, 129.4, 114.7, 110.3 (d, J = 21.6 Hz), 103.2 (d, J = 24.6 Hz), 80.3, 41.8, 25.5, 21.5.

^{19}F NMR (564 MHz, CDCl$_3$) δ (ppm): -112.05 (t, J = 10.7 Hz, 1F).

HRMS (ESI-TOF) *m/z*: [M + Na]$^+$ Calcd for $C_{18}H_{19}{}^{35}ClFNO_5SNa^+$ 438.0549, $C_{18}H_{19}{}^{37}ClFNO_5SNa^+$ 440.0520; Found 438.0550, 440.0525.

***N*-(((4-chlorophenyl)sulfonyl)oxy)-2-(2,3-dichlorophenoxy)-*N*,2-dimethylpropanamide 1q**

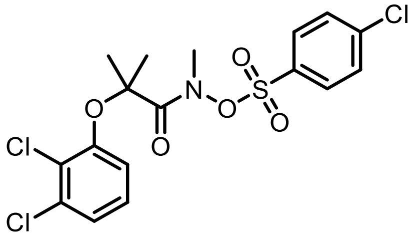

Prepared according to the ***General Procedure A*** by using corresponding **S2** (0.88 g) to afford 0.60 g of **1q** in 37% yield as white solid, m.p. = 100.1 – 103.3 °C.

*NMR and HRMS data for the substrate **1q***:

^{1}H NMR (600 MHz, CDCl$_3$) δ (ppm): 7.90 (d, J = 8.4 Hz, 2H), 7.52 (d, J = 7.8 Hz, 2H), 7.18 (d, J = 7.2 Hz, 1H), 7.10 (t, J = 8.4 Hz, 1H), 6.80 (d, J = 8.4 Hz, 1H), 3.61 (s, 3H), 1.57 (s, 6H).

^{13}C NMR (151 MHz, CDCl$_3$) δ (ppm): 172.6, 151.7, 141.8, 134.2, 133.1, 130.6, 129.4, 127.2, 124.9, 124.5, 116.7, 81.9, 41.9, 25.2.

HRMS (ESI-TOF) *m/z*: [M + Na]$^+$ Calcd for $C_{17}H_{16}{}^{35}Cl^{35}Cl^{35}ClNO_5SNa^+$ 473.9707, $C_{17}H_{16}{}^{37}Cl^{35}Cl^{35}ClNO_5SNa^+$ 475.9678, $C_{17}H_{16}{}^{37}Cl^{37}Cl^{35}ClNO_5SNa^+$ 477.9648; Found 473.9706, 475.9680, 477.9647.

_N_-(((4-chlorophenyl)sulfonyl)oxy)-2-(2,3-dimethoxyphenoxy)-_N_,2-dimethylpropanamide 1r

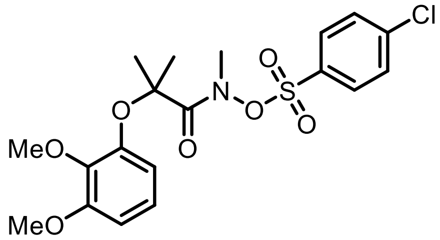

Prepared according to the ***General Procedure A*** by using corresponding **S2** (3.12 g) to afford 2.33 g of **1r** in 40% yield as white solid, m.p. = 90.1 – 92.1 ºC.

*NMR and HRMS data for the substrate **1r***:

^{1}H NMR (600 MHz, $CDCl_3$) δ (ppm): 7.94 (d, J = 9.0 Hz, 2H), 7.51 (d, J = 8.4 Hz, 2H), 6.91 (t, J = 8.4 Hz, 1H), 6.66 (d, J = 8.4 Hz, 1H), 6.45 (d, J = 8.4 Hz, 1H), 3.85 (s, 3H), 3.80 (s, 3H), 3.70 (s, 3H), 1.47 (s, 6H).

^{13}C NMR (151 MHz, $CDCl_3$) δ (ppm): 173.3, 153.8, 147.9, 141.5, 141.1, 133.1, 130.8, 129.3, 123.3, 112.6, 107.4, 81.3, 60.8, 56.0, 42.2, 25.4.

HRMS (ESI-TOF) *m/z*: [M + Na]$^+$ Calcd for $C_{19}H_{22}{}^{35}ClNO_7SNa^+$ 466.0698, $C_{19}H_{22}{}^{37}ClNO_7SNa^+$ 468.0669; Found 466.0702, 468.0676.

_N_-(((4-chlorophenyl)sulfonyl)oxy)-2-(2,6-difluorophenoxy)-_N_,2-dimethylpropanamide 1s

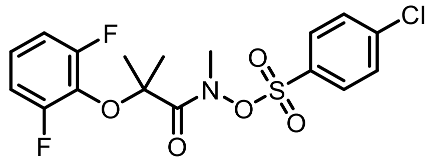

Prepared according to the ***General Procedure A*** by using corresponding **S2** (0.58 g) to afford 0.20 g of **1s** in 19% yield as white solid, m.p. = 76.9 – 79.2 ºC.

*NMR and HRMS data for the substrate **1s***:

^{1}H NMR (600 MHz, $CDCl_3$) δ (ppm): 7.97 (d, J = 7.8 Hz, 2H), 7.51 (d, J = 9.0 Hz, 2H), 7.08 – 7.03 (m, 1H), 6.91 (t, J = 8.4 Hz, 2H), 3.81 (s, 3H), 1.35 (s, 6H).

^{13}C NMR (151 MHz, CDCl$_3$) δ (ppm): 172.5, 157.3 (dd, J = 250.1, 5.7 Hz), 141.4, 132.7, 131.0, 130.0 (t, J = 15.2 Hz), 129.2, 125.0 (t, J = 9.4 Hz), 112.2 (dd, J = 18.7, 4.4 Hz), 84.4, 42.8, 24.7.

^{19}F NMR (564 MHz, CDCl$_3$) δ (ppm): -124.28 (t, J = 6.5 Hz, 1F).

HRMS (ESI-TOF) *m/z*: [M + Na]$^+$ Calcd for $C_{17}H_{16}{}^{35}ClF_2NO_5SNa^+$ 442.0298, $C_{17}H_{16}{}^{37}ClF_2NO_5SNa^+$ 444.0269; Found 442.0298, 444.0260.

<u>*N*-(((4-chlorophenyl)sulfonyl)oxy)-2-(2,6-dimethoxyphenoxy)-*N*,2-dimethylpropanamide 1t</u>

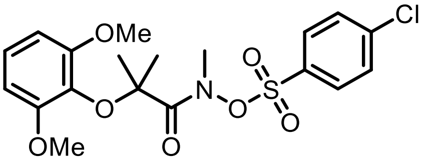

Prepared according to the ***General Procedure A*** by using corresponding **S2** (2.26 g) to afford 1.6 g of **1t** in 38% yield as white solid, m.p. = 129.5 – 130.6 ºC.

*NMR and HRMS data for the substrate **1t***:

^{1}H NMR (600 MHz, CDCl$_3$) δ (ppm): 7.95 (d, J = 9.0 Hz, 2H), 7.49 (d, J = 9.0 Hz, 2H), 6.97 (t, J = 8.4 Hz, 1H), 6.49 (d, J = 8.4 Hz, 2H), 3.78 (s, 3H), 3.73 (s, 6H), 1.23 (s, 6H).

^{13}C NMR (151 MHz, CDCl$_3$) δ (ppm): 172.9, 153.9, 141.2, 132.9, 132.1, 131.0, 129.0, 124.5, 104.4, 81.3, 55.6, 43.3, 25.2.

HRMS (ESI-TOF) *m/z*: [M + Na]$^+$ Calcd for $C_{19}H_{22}{}^{35}ClNO_7SNa^+$ 466.0698, $C_{19}H_{22}{}^{37}ClNO_7SNa^+$ 468.0669; Found 466.0698, 468.0669.

<u>*N*-(((4-chlorophenyl)sulfonyl)oxy)-2-(2-fluoro-5-methylphenoxy)-*N*,2-dimethylpropanamide 1u</u>

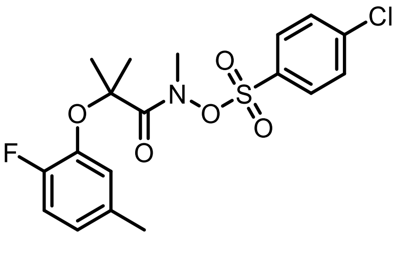

Prepared according to the ***General Procedure A*** by using corresponding **S2** (1.59 g) to afford 0.66 g of **1u** in 21% yield as white solid, m.p. = 94.7 – 96.4 ºC.

*NMR and HRMS data for the substrate **1u***:

^{1}H NMR (600 MHz, CDCl$_3$) δ (ppm): 7.91 (d, *J* = 9.0 Hz, 2H), 7.50 (d, *J* = 7.8 Hz, 2H), 6.96 (dd, *J* = 10.8, 8.4 Hz, 1H), 6.83 (t, *J* = 7.2 Hz, 2H), 3.70 (s, 3H), 2.30 (s, 3H), 1.47 (s, 6H).

^{13}C NMR (151 MHz, CDCl$_3$) δ (ppm): 173.1, 153.0 (d, *J* = 244.3 Hz), 141.5, 141.2 (d, *J* = 11.5 Hz), 134.3 (d, *J* = 2.9 Hz), 133.1, 130.7, 129.3, 124.7 (d, *J* = 7.2 Hz), 122.7, 116.1 (d, *J* = 20.2 Hz), 82.0, 42.3, 25.1, 20.8.

^{19}F NMR (564 MHz, CDCl$_3$) δ (ppm): -134.92 (s, 1F).

HRMS (ESI-TOF) *m/z*: [M + Na]$^+$ Calcd for $C_{18}H_{19}{}^{35}ClFNO_5SNa^+$ 438.0549, $C_{18}H_{19}{}^{37}ClFNO_5SNa^+$ 440.0520; Found 438.0555, 440.0528.

<u>*N*-(((4-chlorophenyl)sulfonyl)oxy)-*N*-isopropyl-2-methyl-2-phenoxypropanamide 1v</u>

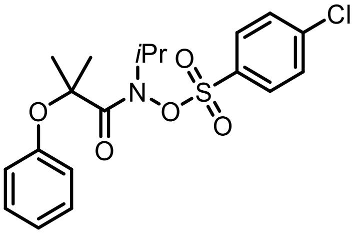

Prepared according to the ***General Procedure A*** by using corresponding **S2** (1.60 g) to afford 0.98 g of **1v** in 27% yield as white solid, m.p. = 122.7 – 123.9 ºC.

*NMR and HRMS data for the substrate **1v***:

^{1}H NMR (600 MHz, CDCl$_3$) δ (ppm): 8.04 (d, *J* = 8.4 Hz, 2H), 7.54 (d, *J* = 9.0 Hz, 2H), 7.28 (t, *J* = 7.8 Hz, 2H), 7.02 (t, *J* = 7.8 Hz, 1H), 6.98 (d, *J* = 8.4 Hz, 2H), 5.14 – 5.08 (m, 1H), 1.60 (s, 6H), 1.16 (d, *J* = 6.0 Hz, 6H).

^{13}C NMR (151 MHz, CDCl$_3$) δ (ppm): 172.7, 154.7, 141.2, 134.1, 130.7, 129.4, 129.2, 122.6, 119.0, 80.4, 55.4, 26.2, 20.2.

HRMS (ESI-TOF) *m/z*: [M + Na]$^+$ Calcd for $C_{19}H_{22}{}^{35}ClNO_5SNa^+$ 434.0799, $C_{19}H_{22}{}^{37}ClNO_5SNa^+$ 436.0770; Found 434.0793, 436.0767.

***N*-benzyl-*N*-(((4-chlorophenyl)sulfonyl)oxy)-2-methyl-2-phenoxypropanamide 1w**

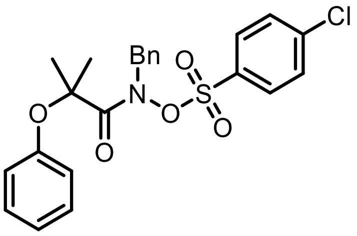

Prepared according to the ***General Procedure A*** by using corresponding **S2** (1.96 g) to afford 2.80 g of **1w** in 56% yield as white solid, m.p. = 89.8 – 92.7 ºC.

*NMR and HRMS data for the substrate **1w***:

^{1}H NMR (600 MHz, CDCl$_3$) δ (ppm): 7.78 (d, *J* = 8.4 Hz, 2H), 7.40 (d, *J* = 7.8 Hz, 2H), 7.30 (t, *J* = 7.8 Hz, 2H), 7.26 – 7.22 (m, 3H), 7.01 – 7.05 (m, 3H), 6.95 (d, *J* = 8.4 Hz, 2H), 5.23 (s, 2H), 1.56 (s, 6H).

^{13}C NMR (151 MHz, CDCl$_3$) δ (ppm): 174.1, 154.2, 141.3, 134.4, 133.6, 130.4, 129.4, 129.3, 128.5, 128.1, 127.9, 122.9, 119.8, 80.9, 57.6, 26.0.

HRMS (ESI-TOF) *m/z*: [M + Na]$^+$ Calcd for $C_{23}H_{22}{}^{35}ClNO_5SNa^+$ 482.0799, $C_{23}H_{22}{}^{37}ClNO_5SNa^+$ 484.0770; Found 482.0794, 484.0762.

***N*-(((4-chlorophenyl)sulfonyl)oxy)-*N*-methyl-2-phenoxypropanamide 1x**

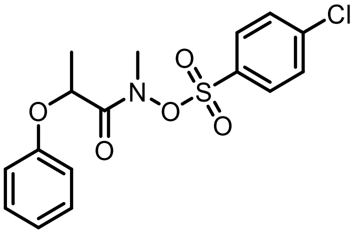

Prepared according to the ***General Procedure A*** by using corresponding **S2** (1.52 g) to afford 0.60 g of **1x** in 18% yield as white solid, m.p. = 97.2 – 99.2 ºC.

*NMR and HRMS data for the substrate **1x***:

^{1}H NMR (600 MHz, CDCl$_3$) δ (ppm): 7.89 (d, *J* = 8.4 Hz, 2H), 7.55 (d, *J* = 9.0 Hz, 2H), 7.26 (t, *J* = 7.8 Hz, 2H), 6.99 (t, *J* = 7.2 Hz, 1H), 6.79 (d, *J* = 7.2 Hz, 2H), 5.05 (q, *J* = 6.6 Hz, 1H), 3.24 (s, 3H), 1.53 (d, *J* = 6.0 Hz, 3H).

^{13}C NMR (151 MHz, CDCl$_3$) δ (ppm): 175.1, 157.0, 142.5, 132.0, 130.7, 130.0, 129.6, 121.8, 115.1, 71.4, 39.2, 17.2.

HRMS (ESI-TOF) *m/z*: [M + H]$^+$ Calcd for $C_{16}H_{17}{}^{35}ClNO_5S^+$ 370.0510, $C_{16}H_{17}{}^{37}ClNO_5S^+$ 372.0481; Found 370.0510, 372.0486.

***N*-(((4-chlorophenyl)sulfonyl)oxy)-*N*-methyl-1-phenoxycyclobutane-1-carboxamide 1y**

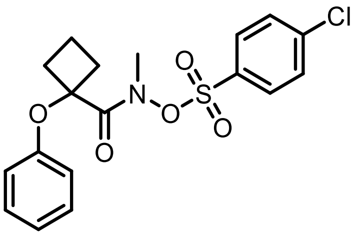

Prepared according to the ***General Procedure A*** by using corresponding **S2** (3.44 g) to afford 1.05 g of **1y** in 15% yield as white solid, m.p. = 103.2 – 105.5 ºC.

*NMR and HRMS data for the substrate **1y***:

^{1}H NMR (600 MHz, CDCl$_3$) δ (ppm): 7.48 (d, *J* = 9.0 Hz, 2H), 7.35 (d, *J* = 9.0 Hz, 2H), 7.27 – 7.24 (m, 2H), 7.03 (t, *J* = 7.2 Hz, 1H), 6.59 (d, *J* = 7.8 Hz, 2H), 3.30 (s, 3H), 2.85 – 2.71 (m, 2H), 2.39 (dd, *J* = 22.2, 9.6 Hz, 2H), 1.96 – 1.89 (m, 1H), 1.84 – 1.76 (m, 1H).

^{13}C NMR (151 MHz, CDCl$_3$) δ (ppm): 171.9, 154.5, 141.3, 132.7, 130.6, 129.7, 129.4, 121.7, 115.6, 80.1, 40.9, 31.5, 13.1.

HRMS (ESI-TOF) *m/z*: [M + Na]$^+$ Calcd for $C_{18}H_{18}{}^{35}ClNO_5SNa^+$ 418.0487, $C_{18}H_{18}{}^{37}ClNO_5SNa^+$ 420.0457; Found 418.0479, 420.0464.

***N*-(((4-chlorophenyl)sulfonyl)oxy)-*N*-methyl-2-phenoxyacetamide 1z**

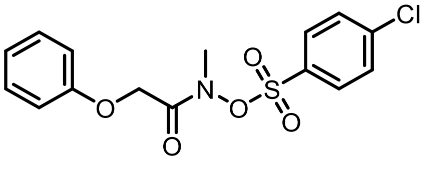

Prepared according to the ***General Procedure A*** by using corresponding **S2** (1.52 g) to afford 1.2 g of **1z** in 34% yield as white solid, m.p. = 103.2 – 105.5 ºC.

*NMR and HRMS data for the substrate **1z***:

^{1}**H NMR (600 MHz, $CDCl_3$) δ (ppm):** 7.94 (d, *J* = 8.4 Hz, 2H), 7.59 (d, *J* = 7.8 Hz, 2H), 7.25 (t, *J* = 7.8 Hz, 2H), 6.97 (t, *J* = 7.8 Hz, 1H), 6.75 (d, *J* = 8.4 Hz, 2H), 4.71 (s, 2H), 3.17 (s, 3H).

^{13}**C NMR (151 MHz, $CDCl_3$) δ (ppm):** 173.2, 157.5, 142.7, 131.6, 130.7, 130.2, 129.5, 121.7, 114.5, 65.5, 38.6.

HRMS (ESI-TOF) *m/z*: $[M + H]^+$ Calcd for $C_{15}H_{15}{}^{35}ClNO_5SH^+$ 356.0354, $C_{15}H_{15}{}^{37}ClNO_5SH^+$ 358.0324; Found 356.0350, 358.0323.

***N*-(((4-chlorophenyl)sulfonyl)oxy)-*N*,2-dimethyl-2-(naphthalen-1-yloxy)propanamide 1aa**

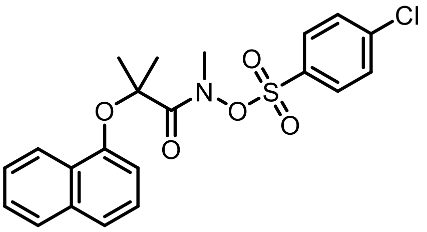

Prepared according to the ***General Procedure A*** by using corresponding **S2** (1.59 g) to afford 0.90 g of **1aa** in 30% yield as white solid, m.p. = 115.6 – 118.2 ºC.

*NMR and HRMS data for the substrate **1aa***:

^{1}**H NMR (600 MHz, $CDCl_3$) δ (ppm):** 8.15 (d, *J* = 8.4 Hz, 1H), 7.90 (d, *J* = 9.0 Hz, 2H), 7.83 (d, *J* = 6.6 Hz, 1H), 7.53 – 7.46 (m, 5H), 7.32 (t, *J* = 7.8 Hz, 1H), 6.74 (d, *J* = 7.2 Hz, 1H), 3.56 (s, 3H), 1.67 (s, 6H).

^{13}**C NMR (151 MHz, $CDCl_3$) δ (ppm):** 173.3, 149.9, 141.6, 134.8, 133.2, 130.7, 129.3, 127.8, 126.9, 126.5, 125.7, 125.5, 122.1, 121.8, 110.2, 80.4, 41.7, 25.4.

HRMS (ESI-TOF) *m/z*: $[M + Na]^+$ Calcd for $C_{21}H_{20}{}^{35}ClNO_5SNa^+$ 456.0643, $C_{21}H_{20}{}^{37}ClNO_5SNa^+$ 458.0614; Found 456.0640, 458.0614.

N-(((4-chlorophenyl)sulfonyl)oxy)-N,2-dimethyl-2-((5,6,7,8-tetrahydronaphthalen-1-yl)oxy)propanamide 1ab

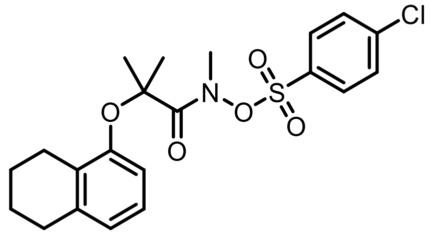

Prepared according to the ***General Procedure A*** by using corresponding **S2** (0.61 g) to afford 0.46 g of **1ab** in 40% yield as white solid, m.p. = 100.7 – 104.6 ºC.

*NMR and HRMS data for the substrate **1ab***:

^{1}H NMR (600 MHz, CDCl$_3$) δ (ppm): 7.92 (d, *J* = 7.8 Hz, 2H), 7.51 (d, *J* = 8.4 Hz, 2H), 6.96 (t, *J* = 7.8 Hz, 1H), 6.76 (d, *J* = 7.8 Hz, 1H), 6.43 (d, *J* = 7.8 Hz, 1H), 3.59 (s, 3H), 2.75 (t, *J* = 5.4 Hz, 2H), 2.58 (t, *J* = 6.0 Hz, 2H), 1.77 – 1.74 (m, 4H), 1.51 (s, 6H).

^{13}C NMR (151 MHz, CDCl$_3$) δ (ppm): 173.6, 152.2, 141.6, 139.2, 133.2, 130.8, 129.3, 128.1, 125.5, 123.2, 112.7, 79.6, 41.8, 29.6, 25.6, 23.6, 22.8, 22.7.

HRMS (ESI-TOF) *m/z*: [M + Na]$^+$ Calcd for $C_{21}H_{24}{}^{35}ClNO_5SNa^+$ 460.0956, $C_{21}H_{24}{}^{37}ClNO_5SNa^+$ 462.0927; Found 460.0956, 462.0934.

N-(((4-chlorophenyl)sulfonyl)oxy)-2-((2,3-dihydrobenzo[b][1,4]dioxin-5-yl)oxy)-N,2-dimethylpropanamide 1ac

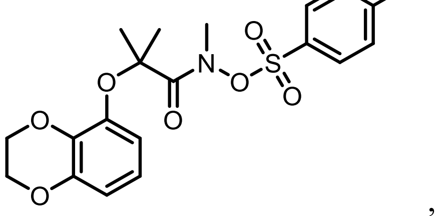

,

Prepared according to the ***General Procedure A*** by using corresponding **S2** (1.11 g) to afford 0.54 g of **1ac** in 26% yield as white solid, m.p. = 122.7 – 123.9 ºC.

*NMR and HRMS data for the substrate **1ac***:

^{1}H NMR (600 MHz, CDCl$_3$) δ (ppm): 7.93 (d, *J* = 8.4 Hz, 2H), 7.51 (d, *J* = 7.8 Hz, 2H), 6.70 (t, *J* = 7.8 Hz, 1H), 6.62 (d, *J* = 7.8 Hz, 1H), 6.43 (d, *J* = 7.2 Hz, 1H), 4.22 (q, *J* = 4.8 Hz, 4H), 3.72 (s, 3H), 1.41 (s, 6H).

^{13}C NMR (151 MHz, CDCl$_3$) δ (ppm): 173.2, 144.6, 143.2, 141.4, 136.4, 133.0, 130.9, 129.2, 120.2, 113.7, 112.8, 81.4, 64.11, 64.08, 42.5, 25.4.

HRMS (ESI-TOF) *m/z*: [M + Na]$^+$ Calcd for $C_{19}H_{20}{}^{35}ClNO_7SNa^+$ 464.0541, $C_{19}H_{20}{}^{37}ClNO_7SNa^+$ 466.0512; Found 464.0534, 466.0513.

<u>*N*-(((4-methoxyphenyl)sulfonyl)oxy)-*N*,2-dimethyl-2-phenoxypropanamide 1ad</u>

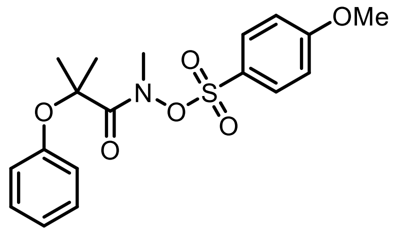

Prepared according to the ***General Procedure A*** by using corresponding **S2** (0.86 g) to afford 1.05 g of **1ad** in 56% yield as white solid, m.p. = 89.5 – 91.1 °C.

*NMR and HRMS data for the substrate **1ad***:

^{1}H NMR (600 MHz, CDCl$_3$) δ (ppm): 7.88 (d, *J* = 9.0 Hz, 2H), 7.26 (t, *J* = 8.4 Hz, 2H), 7.03 (t, *J* = 7.8 Hz, 1H), 6.97 (d, *J* = 9.0 Hz, 2H), 6.83 (d, *J* = 9.0 Hz, 2H), 3.88 (s, 3H), 3.56 (s, 3H), 1.52 (s, 6H).

^{13}C NMR (151 MHz, CDCl$_3$) δ (ppm): 173.2, 164.6, 154.5, 131.7, 129.5, 125.8, 122.5, 118.4, 114.2, 80.0, 55.7, 41.6, 25.5.

HRMS (ESI-TOF) *m/z*: [M + Na]$^+$ Calcd for $C_{18}H_{21}NO_6SNa^+$ 402.0982; Found 402.0991.

<u>*N*,2-dimethyl-*N*-((methylsulfonyl)oxy)-2-phenoxypropanamide 1ae</u>

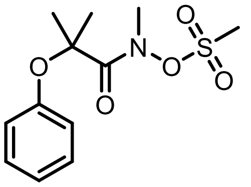

Prepared according to the ***General Procedure A*** by using corresponding **S2** (0.86 g) to afford 1.05 g of **1ae** in 76% yield as colorless oil.

*NMR and HRMS data for the substrate **1ae***:

^{1}H NMR (600 MHz, $CDCl_3$) δ (ppm): 7.28 (t, J = 7.8 Hz, 2H), 7.04 (t, J = 7.8 Hz, 1H), 6.89 (d, J = 7.8 Hz, 2H), 3.75 (s, 3H), 3.28 (s, 3H), 1.64 (s, 6H).

^{13}C NMR (151 MHz, $CDCl_3$) δ (ppm): 172.5, 154.5, 129.7, 122.9, 118.6, 80.1, 42.3, 39.5, 25.5.

HRMS (ESI-TOF) *m/z*: $[M + Na]^+$ Calcd for $C_{12}H_{17}NO_5SNa^+$ 310.0720; Found 310.0722.

<u>*N*,2-dimethyl-2-phenoxy-*N*-(tosyloxy)propanamide 1af</u>

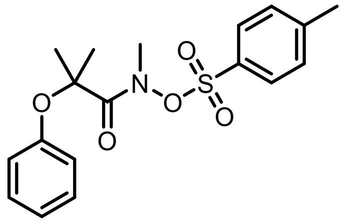

Prepared according to the ***General Procedure A*** by using corresponding **S2** (0.86 g) to afford 0.80 g of **1af** in 46% yield as white solid, m.p. = 94.0 – 95.5 ºC.

*NMR and HRMS data for the substrate **1af**:*

^{1}H NMR (600 MHz, $CDCl_3$) δ (ppm): 7.83 (d, J = 8.4 Hz, 2H), 7.32 (d, J = 8.4 Hz, 2H), 7.26 (t, J = 7.8 Hz, 2H), 7.03 (t, J = 7.2 Hz, 1H), 6.83 (d, J = 7.2 Hz, 2H), 3.55 (s, 3H), 2.44 (s, 3H), 1.51 (s, 6H).

^{13}C NMR (151 MHz, $CDCl_3$) δ (ppm): 173.2, 154.5, 146.0, 131.7, 129.6, 129.5, 129.3, 122.5, 118.4, 80.0, 41.6, 25.5, 21.8.

HRMS (ESI-TOF) *m/z*: $[M + Na]^+$ Calcd for $C_{18}H_{21}NO_5SNa^+$ 386.1033; Found 386.1035.

<u>*N*-(((4-(*tert*-butyl)phenyl)sulfonyl)oxy)-*N*,2-dimethyl-2-phenoxypropanamide 1ag</u>

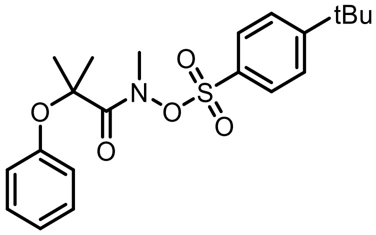

Prepared according to the ***General Procedure A*** by using corresponding **S2** (0.86 g) to afford 1.17 g of **1ag** in 60% yield as white solid, m.p. = 82.6 – 84.0 °C.

*NMR and HRMS data for the substrate **1ag***:

^{1}H NMR (600 MHz, CDCl$_3$) δ (ppm): 7.90 (d, J = 9.0 Hz, 2H), 7.53 (d, J = 8.4 Hz, 2H), 7.26 (t, J = 7.8 Hz, 2H), 7.03 (t, J = 7.8 Hz, 1H), 6.83 (d, J = 9.0 Hz, 2H), 3.57 (s, 3H), 1.49 (s, 6H), 1.34 (s, 9H).

^{13}C NMR (151 MHz, CDCl$_3$) δ (ppm): 173.2, 159.0, 154.5, 131.4, 129.5, 129.3, 126.0, 122.5, 118.6, 80.1, 41.6, 35.4, 31.0, 25.5.

HRMS (ESI-TOF) *m/z*: $[M + Na]^+$ Calcd for $C_{21}H_{27}NO_5SNa^+$ 428.1503; Found 428.1498.

<u>*N*,2-dimethyl-2-phenoxy-*N*-((4-(trifluoromethyl)benzoyl)oxy)propanamide 1ah</u>

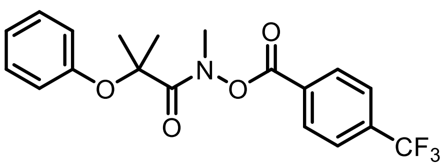

Prepared according to the ***General Procedure A*** by using corresponding **S2** (1.8g) to afford 1.2 g of **1ah** in 32% yield as colorless oil.

*NMR and HRMS data for the substrate **1ah***:

^{1}H NMR (600 MHz, CDCl$_3$) δ (ppm): 8.14 (s, 2H), 7.72 (d, J = 8.4 Hz, 2H), 7.26-7.25 (m, 2H), 7.01-6.89 (m, 3H), 3.63 (s, 3H), 1.65 (s, 6H).

^{13}C NMR (151 MHz, CDCl$_3$) δ (ppm): 162.9, 155.0, 135.4 (C-F, $^2J_{C\text{-}F}$ = 31.8 Hz), 130.7, 130.4, 129.3, 125.7, 125.6, 123.4, (C-F, $^1J_{C\text{-}F}$ = 273.3 Hz), 122.3, 118.7, 80.1, 38.9, 25.4.

^{19}F NMR (564 MHz, CDCl$_3$) δ (ppm): -63.25 (s, 1F).

HRMS (ESI-TOF) *m/z*: $[M + H]^+$ Calcd for $C_{19}H_{18}F_3NO_4H^+$ 382.1261, Found 382.1258.

<u>*N*-((3,5-bis(trifluoromethyl)benzoyl)oxy)-*N*,2-dimethyl-2-phenoxypropanamide 1ai</u>

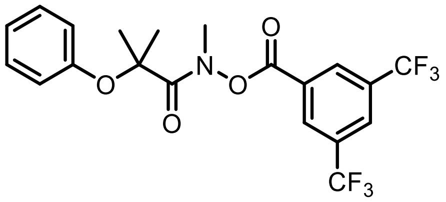

Prepared according to the ***General Procedure A*** by using corresponding **S2** (1.6g) to afford 1.3 g of **1ai** in 32% yield as white solid.

*NMR and HRMS data for the substrate **1ai***:

^{1}H NMR (600 MHz, $CDCl_3$) δ (ppm): 8.48 (s, 2H), 8.13 (s, 1H), 7.28 (t, J = 7.8 Hz, 2H), 7.03 (t, J = 7.2 Hz, 1H), 6.91 (s, 2H), 3.68 (s, 3H), 1.67 (s, 6H).

^{13}C NMR (151 MHz, $CDCl_3$) δ (ppm): 161.7, 154.9, 132.6(C-F, $^2J_{C\text{-}F}$ = 34.7 Hz), 130.1(C-F, $^4J_{C\text{-}F}$ = 2.9 Hz), 129.7, 129.5, 127.3(C-F, $^3J_{C\text{-}F}$ = 7.2 Hz), 127.2, 122.7(C-F, $^1J_{C\text{-}F}$ = 273.3 Hz), 122.5, 118.6, 80.2, 38.9, 25.4.

^{19}F NMR (564 MHz, $CDCl_3$) δ (ppm): -62.91 (s, 1F).

HRMS (ESI-TOF) *m/z*: $[M + H]^+$ Calcd for $C_{20}H_{17}F_6NO_4H^+$ 450.1135, Found 450.1141.

2-(anthracen-9-yloxy)-*N*-(((4-chlorophenyl)sulfonyl)oxy)-*N*,2-dimethylpropanamide 1aj

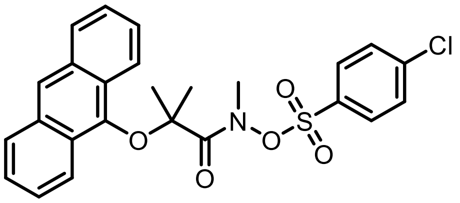

Prepared according to the ***General Procedure A*** by using corresponding **S2** (1.2 g) to afford 1.02 g of **1aj** in 36% yield as white solid, m.p. = 70.0 – 71.3 °C.

*NMR and HRMS data for the substrate **1aj***:

^{1}H NMR (600 MHz, $CDCl_3$) δ (ppm): 8.24 (s, 1H), 8.11 (d, J = 8.4 Hz, 2H), 8.01-7.97 (m, 4H), 7.50-7.44 (m, 4H), 7.34 (d, J = 7.8 Hz, 2H), 4.05 (s, 3H), 1.46 (s, 6H).

^{13}C NMR (151 MHz, $CDCl_3$) δ (ppm): 174.0, 146.5, 141.4, 133.6, 132.1, 130.7, 129.3, 128.4, 127.1, 125.8, 125.4, 123.4, 122.9, 84.9, 43.0, 25.9.

HRMS (ESI-TOF) *m/z*: $[M + Na]^+$ Calcd for $C_{25}H_{22}{}^{35}ClNO_5SNa^+$ 506.0799, $C_{25}H_{22}{}^{37}ClNO_5SNa^+$ 508.0770; Found 506.0800, 508.0769.

***N*-(((4-chlorophenyl)sulfonyl)oxy)-*N*,2-dimethyl-2-(pyridin-3-yloxy)propanamide 1ak**

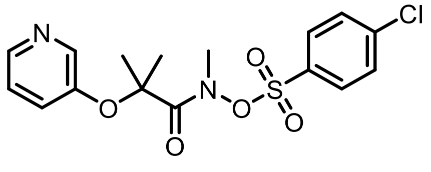

Prepared according to the ***General Procedure A*** by using corresponding **S2** (1.52 g) to afford 62mg of **1ak** in as white solid.

*NMR and HRMS data for the substrate **1ak***:

^{1}H NMR (600 MHz, CDCl3) δ (ppm): 8.27 (d, J = 4.2 Hz, 1H), 8.19 (s, 1H), 7.84 (d, J = 7.8 Hz, 2H), 7.48 (d, J = 7.8 Hz, 2H), 7.20-7.17 (m, 1H), 7.14-7.12 (m, 1H), 3.53 (s, 3H), 1.51 (s, 6H).

^{13}C NMR (151 MHz, CDCl3) δ (ppm): 172.5, 150.8, 143.7, 141.6, 133.0, 130.5, 129.4, 125.0, 123.7, 80.7, 41.7, 25.2.

HRMS (ESI-TOF) *m/z*: [M + Na]$^+$ Calcd for $C_{16}H_{17}{}^{35}ClN_2O_5SNa^+$ 407.0439, $C_{16}H_{17}{}^{37}ClN_2O_5SNa^+$ 409.0409; Found 407.0446, 409.0410.

<u>*N*-(((4-chlorophenyl)sulfonyl)oxy)-*N*-methyl-2-phenoxybenzamide 4</u>

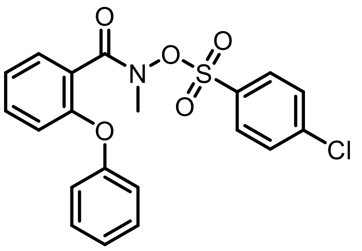

Prepared according to the ***General Procedure A*** by using corresponding **S2** (2.1 g) to afford 1.5 g of **4** in 36% yield as white solid, m.p. = 95.2 – 98.0 ºC.

*NMR and HRMS data for the substrate **4***:

^{1}H NMR (600 MHz, CDCl3) δ (ppm): 7.65 (d, J = 8.4 Hz, 2H), 7.38 (t, J = 7.8 Hz, 2H), 7.31-7.26 (m, 3H), 7.19 (t, J = 7.8 Hz, 1H), 7.04-6.98 (m, 4H), 6.75 (d, J = 7.8 Hz, 1H), 3.50 (s, 3H).

^{13}C NMR (151 MHz, CDCl3) δ (ppm): 169.5, 155.8, 154.0, 141.5, 131.8, 130.6, 130.0, 129.3, 129.1, 124.5, 124.3, 122.8, 119.5, 117.3, 40.0.

HRMS (ESI-TOF) *m/z*: [M + H]$^+$ Calcd for $C_{20}H_{17}{}^{35}ClNO_5SNa^+$ 418.0510, $C_{20}H_{17}{}^{35}ClNO_5SNa^+$ 420.0481; Found 418.0505, 420.0480.

***N*-(((4-chlorophenyl)sulfonyl)oxy)-*N*-methyl-2,3-dihydrobenzo[b][1,4]dioxine-2-carboxamide 7a**

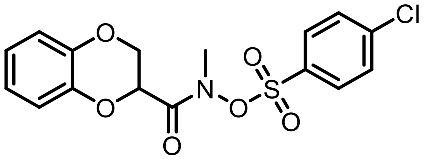

Prepared according to the ***General Procedure A*** by using corresponding **S2** (1.10 g) to afford 0.42 g of **7a** in 18% yield as white solid, m.p. = 89.6 – 91.2 ºC.

*NMR and HRMS data for the substrate 7**a***:

^{1}H NMR (600 MHz, CDCl3) δ (ppm): 7.87 (d, *J* = 8.4 Hz, 2H), 7.52 (d, *J* = 7.8 Hz, 2H), 6.93 – 6.85 (m, 4H), 4.90 (t, *J* = 4.2 Hz, 1H), 4.27 (d, *J* = 4.8 Hz, 2H), 3.35 (s, 3H).

^{13}C NMR (151 MHz, CDCl3) δ (ppm): 169.9, 142.7, 142.6, 142.1, 131.5, 130.6, 130.0, 122.04, 122.01, 117.3, 117.2, 70.2, 63.8, 39.2.

HRMS (ESI-TOF) *m/z*: [M + H]$^+$ Calcd for $C_{16}H_{15}{}^{35}ClNO_6S^+$ 384.0303, $C_{16}H_{15}{}^{37}ClNO_6S^+$ 386.0274; Found 384.0304, 386.0266.

The preperation of thiazolium NHC pre-catalysts

NHC *pre*-catalysts **N1**[5,6], **N2**[5,6], **N3**[7], **N4**[7], **N5**[7], **N6**[8], **N7**[5,6], **N9**[7], **N11**[8], **N12**[5,6] were synthesized according to the reported literature procedures.

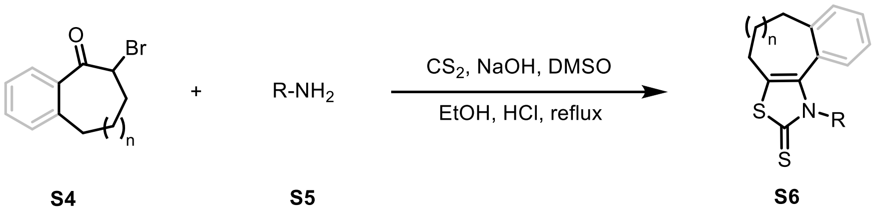

Synthesis of thiazole-2-thiones **S6**: To a solution of the amine **S5** (1.0 eq.) in DMSO (0.5 mmol/mL) was treated with 20 N aq. NaOH solution (2.0 eq.) at 0 °C, CS_2 was was added dropwise and stirred for 1 h at room temperature, and the colour of resulting mixture getted red from light yellow. Then, **S4** (1 eq.) was added, the mixture was stirred for 12 h at room temperature, and the colour of resulting mixture getted back to light yellow. After the reaction finished, the reaction mixture diluted with water and extracted by EA three times. The combined organic phases were washed with brine, dried over anhydrous Na_2SO_4, filtered and concentrated

in vacuum. The crude product did not need to be futher purified and was suspended in EtOH (1 mL/mmol), concd. HCl (0.05 mL/mmol) was added and the mixture was heated to reflux for 1 h. Also, the same extraction operation was used and the crude residue was then purified by column chromatography on silica gel eluting from petroleum ether/ethyl acetate (20:1 to 10:1) to provide the thione **S6**.

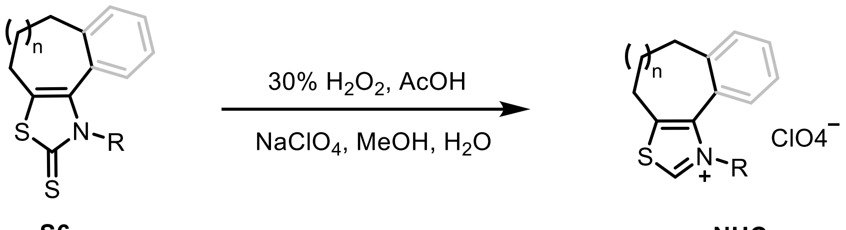

Synthesis of thiazolium *pre*-NHCs: To a solution of the thione **S6** (1.0 eq.) in AcOH (5.0 mmol/mL) was was slowly added 30% H_2O_2 (4.0 eq.) at 0 °C, and the mixture was stirred for 1 h at room temperature. After that, the solution was concentrated in vacuum to remove all AcOH. Then, the residue was dissolved in MeOH (1.0 mL/mmol) and a mixture of sodium perchlorate monohydrate (4.0 eq) in a mixture of MeOH/H_2O = 2/1 (3.0 mL/mmol) was added at 0 °C. After the reaction finished, the reaction mixture was extracted by ethyl acetate three times and the crude residue was then purified by column chromatography on silica gel eluting from DCM/MeOH (50:1 to 10:1) to provide the thiazolium *pre*-NHCs (**N1**–**N7**, **N11** and **N12**).

3-neopentyl-5,6,7,8-tetrahydro-4H-cyclohepta[d]thiazol-3-ium perchlorate N1

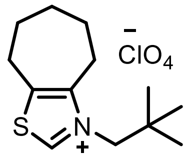

*NMR and HRMS data for the product **N1***:

^{1}H NMR (600 MHz, CDCl$_3$) δ (ppm): 9.59 (s, 1H), 4.36 (s, 2H), 3.01-2.98 (m, 4H), 1.96-1.94 (m, 2H), 1.82-1.78 (m, 4H), 1.02 (s, 9H).

^{13}C NMR (151 MHz, CDCl$_3$) δ (ppm): 154.5, 148.8, 139.1, 63.6, 33.6, 30.9, 28.1, 27.9, 27.3, 26.3, 25.1.

HRMS (ESI-TOF) *m/z*: $[M + H]^+$ Calcd for $C_{13}H_{22}NS^+$ 224.1467; Found 224.1475.

3-(2,2-diphenylethyl)-5,6,7,8-tetrahydro-4H-cyclohepta[d]thiazol-3-ium perchlorate N2

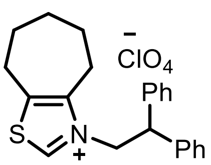

*NMR and HRMS data for the product **N2***:

^{1}H NMR (600 MHz, $CDCl_3$) δ (ppm): 9.15 (s, 1H), 7.30 (t, *J* = 7.2 Hz, 5H), 7.23 (t, *J* = 7.8 Hz, 5H), 5.11 (d, *J* = 8.4 Hz, 2H), 4.32 (t, *J* = 8.4 Hz, 1H), 2.87 (t, *J* = 5.4 Hz, 4H), 1.91-1.90 (m, 2H), 1.75-1.70 (m, 4H).

^{13}C NMR (151 MHz, $CDCl_3$) δ (ppm): 153.8, 147.6, 139.1, 138.6, 129.3, 127.9, 127.8, 57.9, 51.7, 30.5, 27.8, 27.0, 26.3, 24.9.

HRMS (ESI-TOF) *m/z*: $[M + H]^+$ Calcd for $C_{22}H_{24}NS^+$ 334.1624; Found 334.1628.

3-(3,3-diphenylpropyl)-5,6,7,8-tetrahydro-4H-cyclohepta[d]thiazol-3-ium perchlorate N 3

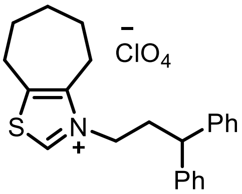

*NMR and HRMS data for the product **N3***:

^{1}H NMR (600 MHz, $CDCl_3$) δ (ppm): 9.46 (s, 1H), 7.26 (d, *J* = 4.8 Hz, 8H), 7.17-7.14 (m, 2H), 4.40 (t, *J* = 7.8 Hz, 2H), 4.14 (t, *J* = 7.8 Hz, 1H), 2.82 (t, *J* = 6.0 Hz, 2H), 2.71 (t, *J* = 5.4 Hz, 2H), 2.60-2.56 (m, 2H), 1.88-1.87 (m, 2H), 1.75-1.68 (m, 4H).

^{13}C NMR (151 MHz, $CDCl_3$) δ (ppm): 153.3, 147.3, 142.8, 139.8, 128.9, 127.6, 126.8, 53.2, 48.3, 35.2, 30.5, 27.7, 26.7, 26.2, 24.8.

HRMS (ESI-TOF) *m/z*: $[M + H]^+$ Calcd for $C_{23}H_{26}NS^+$ 348.1780; Found 348.1784.

3-cyclohexyl-5,6,7,8-tetrahydro-4H-cyclohepta[d]thiazol-3-ium perchlorate N4

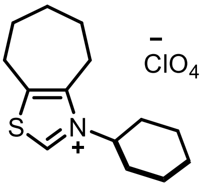

*NMR and HRMS data for the product **N4***:

^{1}H NMR (600 MHz, CDCl$_3$) δ (ppm): 9.56 (s, 1H), 4.37 (t, *J* = 12.0 Hz, 1H), 3.00-2.96 (m, 4H), 2.15-2.13 (m, 2H), 1.97-1.95 (m, 4H), 1.84-1.74 (m, 7H), 1.54-1.47 (m, 2H), 1.35-1.30 (m, 1H).

^{13}C NMR (151 MHz, CDCl$_3$) δ (ppm): 150.8, 147.8, 139.8, 63.7, 33.1, 30.6, 27.6, 26.7, 26.1, 25.2, 25.0, 24.5.

HRMS (ESI-TOF) *m/z*: $[M + H]^+$ Calcd for $C_{14}H_{22}NS^+$ 236.1467; Found 236.1471.

<u>3-(2,6-diisopropylphenyl)-5,6,7,8-tetrahydro-4H-cyclohepta[d]thiazol-3-ium perchlorate N5</u>

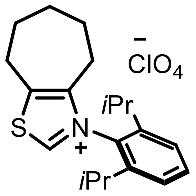

*NMR and HRMS data for the product **N5***:

^{1}H NMR (600 MHz, CDCl$_3$) δ (ppm): 9.68 (s, 1H), 7.60 (t, *J* = 7.2 Hz, 1H), 7.36 (d, *J* = 7.8 Hz, 2H), 3.18 (t, *J* = 5.4 Hz, 2H), 2.55 (t, *J* = 6.0 Hz, 2H), 2.08-2.04 (m, 2H), 1.96-1.95 (m, 2H), 1.92-1.91 (m, 2H), 1.64-1.62 (m, 2H), 1.17 (t, *J* = 6.0 Hz, 12H)

^{13}C NMR (151 MHz, CDCl$_3$) δ (ppm): 155.6, 148.5, 144.9, 141.0, 132.5, 132.1, 125.2, 30.8, 28.8, 28.2, 27.3, 26.6, 25.5, 24.8, 23.2.

HRMS (ESI-TOF) *m/z*: $[M + H]^+$ Calcd for $C_{20}H_{28}NS^+$ 314.1937; Found 314.1940.

<u>3-(2,6-dimethoxyphenyl)-5,6,7,8-tetrahydro-4H-cyclohepta[d]thiazol-3-ium perchlorate N6</u>

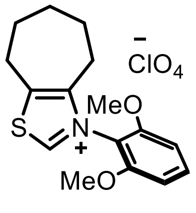

*NMR and HRMS data for the product **N6***:

^{1}**H NMR (600 MHz, CDCl$_3$) δ (ppm):** 9.55 (s, 1H), 7.53 (t, *J* = 8.4 Hz, 1H), 6.74 (d, *J* = 8.4 Hz, 2H), 3.82 (s, 6H), 3.05 (t, *J* = 5.4 Hz, 2H), 2.56 (t, *J* = 5.4 Hz, 2H), 1.94-1.94 (m, 2H), 1.88-1.87 (m, 2H), 1.66-1.64 (m, 2H).

^{13}**C NMR (151 MHz, CDCl$_3$) δ (ppm):** 157.2, 154.7, 148.9, 138.1, 133.6, 113.6, 104.6, 56.5, 30.9, 28.0, 27.1, 26.6, 25.1.

HRMS (ESI-TOF) *m/z*: $[M + H]^+$ Calcd for $C_{16}H_{20}NS^+$ 290.1209; Found 290.1203.

3-mesityl-5,6,7,8-tetrahydro-4H-cyclohepta[d]thiazol-3-ium perchlorate N7

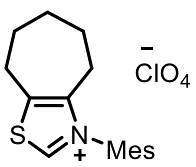

NMR and HRMS data for the product ***N7***:

^{1}**H NMR (600 MHz, CDCl$_3$) δ (ppm):** 10.25 (s, 1H), 7.02 (s, 2H), 3.06 (t, *J* = 5.4 Hz, 2H), 2.50 (t, *J* = 5.4 Hz, 2H), 2.33 (s, 3H), 1.93 (m, 8H), 1.84-1.83 (m, 2H), 1.62-1.61 (m, 2H).

^{13}**C NMR (151 MHz, CDCl$_3$) δ (ppm):** 158.2, 147.4, 141.9, 140.4, 133.8, 132.7, 130.0, 30.7, 28.0, 26.7, 26.6, 25.5, 21.0, 17.3.

HRMS (ESI-TOF) *m/z*: $[M + H]^+$ Calcd for $C_{17}H_{22}NS^+$ 272.1467; Found 272.1476.

1-(2,6-diisopropylphenyl)-5,6-dihydro-4H-benzo[6,7]cyclohepta[1,2-d]thiazol-1-ium perchlorate N11

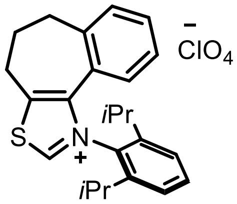

NMR and HRMS data for the product ***N11***:

^{1}**H NMR (600 MHz, CDCl$_3$) δ (ppm):** 9.99 (s, 1H), 7.55 (t, *J* = 7.8 Hz, 1H), 7.38 (d, *J* = 7.2 Hz, 1H), 7.33 (t, *J* = 7.8 Hz, 1H), 7.28 (d, *J* = 7.2 Hz, 2H), 7.04 (t, *J* = 7.8 Hz, 1H), 6.58 (d, *J* = 8.4 Hz, 1H), 2.99-2.99 (m, 2H), 2.65-2.62 (m, 2H), 2.57-2.54 (m, 2H), 2.16-2.16 (m, 2H), 1.21 (d, *J* = 6.6 Hz, 6H), 0.88-0.88 (m, 6H)

^{13}**C NMR (151 MHz, CDCl$_3$) δ (ppm):** 157.8, 144.7, 144.4, 141.7, 140.0, 132.4, 132.2, 131.1, 130.5, 127.6, 126.8, 126.0, 125.2, 34.1, 31.4, 29.0, 25.4, 23.9, 22.4.

HRMS (ESI-TOF) *m/z*: [M + H]$^+$ Calcd for $C_{24}H_{28}NS^+$ 362.1937; Found 362.1942.

3-(2,6-diisopropylphenyl)-4,5,6,7-tetrahydrobenzo[d]thiazol-3-ium perchlorate N12

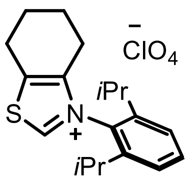

*NMR and HRMS data for the product **N12***:

^{1}H NMR (600 MHz, CDCl$_3$) δ (ppm): 9.81 (s, 1H), 7.58 (t, *J* = 7.8 Hz, 1H), 7.35 (d, *J* = 7.8 Hz, 2H), 3.10 (t, *J* = 5.4 Hz, 2H), 2.28-2.26 (m, 2H), 2.07-2.02 (m, 2H), 1.99-1.95 (m, 2H), 1.91-1.88 (m, 2H), 1.18 (d, *J* = 6.6 Hz, 6H), 1.14 (d, *J* = 6.6 Hz, 6H)

^{13}C NMR (151 MHz, CDCl$_3$) δ (ppm): 157.1, 144.6, 144.2, 137.5, 132.4, 131.1, 125.2, 28.7, 24.9, 23.7, 23.4, 23.0, 21.6, 20.7.

HRMS (ESI-TOF) *m/z*: [M + H]$^+$ Calcd for $C_{19}H_{26}NS^+$ 300.1780; Found 300.1786.

*The preperation of triazolium NHC **N9***

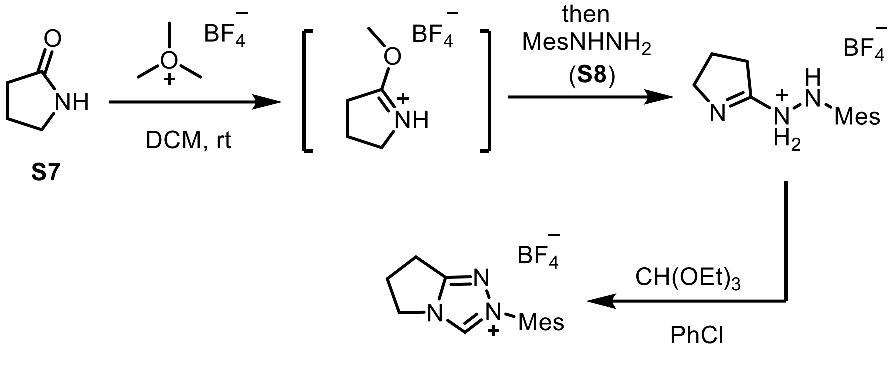

A flame-dried round-bottom flask was charged with **S7** (1 eq.) and DCM (5.0 mL/mmol). Trimethyloxonium tetrafluoroborate (1 eq.) was added and the reaction mixture stirred for 12 h at room temperature. **S8** (1.1 eq.) was then added and allowed to stir for 2 h at room temperature. The solvent was removed in vacuo, and chlorobenzene (10 mL/mmol) was added, followed by triethyl orthoformate (2.5 eq.). The resulting solution was stirred at 110 °C for 12 h. At this time, additional triethyl orthoformate (2.5 eq.) was added, and heating at 110 °C was continued for 12 h. Upon cooling, ethyl acetate (10 mL/mmol) was added, and the light tan solid product was collected by filtration.

2-mesityl-6,7-dihydro-5H-pyrrolo[2,1-c][1,2,4]triazol-2-ium tetrafluoroborate fluoroborate N9

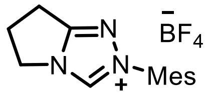

NMR and HRMS data for the product ***N9***:

^{1}H NMR (600 MHz, $CDCl_3$) δ (ppm): 9.38 (s, 1H), 6.96 (s, 2H), 4.61 (t, J = 7.2 Hz, 2H), 3.20 (t, J = 7.8 Hz, 2H), 2.86-2.83 (m, 2H), 2.34 (s, 3H), 2.04 (s, 6H)

^{13}C NMR (151 MHz, $CDCl_3$) δ (ppm): 162.9, 141.7, 141.1, 135.2, 131.8, 129.5, 47.7, 26.5, 21.8, 21.1, 17.1.

HRMS (ESI-TOF) *m/z*: $[M + H]^+$ Calcd for $C_{14}H_{18}NS^+$ 228.1495; Found 228.1500.

4. The NHC-Catalyzed Difunctionalization

General Procedure B: *the NHC-catalyzed difunctionalization of arenes*

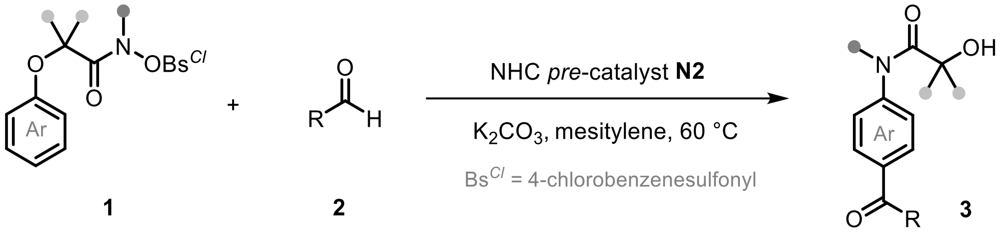

To an oven-dried Schlenck tube was added substrates **1** (0.10 mmol), NHC **N2** (10 mol %) and K_2CO_3 (0.20 mmol). The Schlenck tube was subjected to three cycles of pressurization/depressurization using dry Ar. After that, under the protection of Ar atmosphere, a solution of aldehydes **2** (0.25 mmol) in dry mesitylene (1 mL) was added, and the reaction mixture was stirred at 60 °C for 12 hours. Then the mixture was purified by column chromatography on silica gel to afford the corresponding products **3**, which were dried under vacuum and further analyzed by ^{1}H NMR, ^{13}C NMR, HRMS, etc.

2-hydroxy-*N*,2-dimethyl-*N*-(4-(3-phenylpropanoyl)phenyl)propanamide 3a

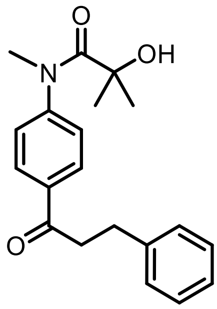

Prepared according to the ***General Procedure B*** to afford **3a** (28.6 mg) in 88% yield as colorless oil.

*NMR and HRMS data for the product **3a***:

^{1}H NMR (600 MHz, $CDCl_3$) δ (ppm): 8.00 (d, *J* = 9.0 Hz, 2H), 7.33 (d, *J* = 9.0 Hz, 2H), 7.30 (t, *J* = 7.8 Hz, 2H), 7.25 (d, *J* = 7.2 Hz, 2H), 7.21 (t, *J* = 7.2 Hz, 1H), 4.08 (brs, 1H), 3.33 (s, 3H), 3.31 (t, *J* = 7.8 Hz, 2H), 3.08 (t, *J* = 7.8 Hz, 2H), 1.23 (s, 6H).

^{13}C NMR (151 MHz, $CDCl_3$) δ (ppm): 197.9, 176.6, 148.1, 140.9, 136.4, 129.3, 128.6, 128.5, 128.4, 126.2, 73.1, 40.7, 40.5, 30.0, 28.6.

HRMS (ESI-TOF) *m/z*: $[M + Na]^+$ Calcd for $C_{20}H_{23}NO_3Na^+$ 348.1571; Found 348.1579.

***N*-(4-(3-(4-bromophenyl)propanoyl)phenyl)-2-hydroxy-*N*,2-dimethylpropanamide 3b**

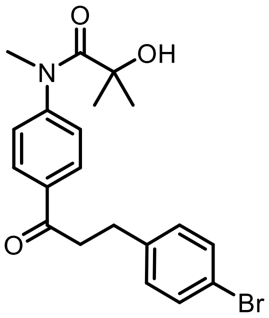

Prepared according to the ***General Procedure B*** to afford **3b** (36.4 mg) in 90% yield as colorless oil.

*NMR and HRMS data for the product **3b***:

^{1}H NMR (600 MHz, $CDCl_3$) δ (ppm): 7.99 (d, *J* = 9.0 Hz, 2H), 7.41 (d, *J* = 8.4 Hz, 2H), 7.34 (d, *J* = 8.4 Hz, 2H), 7.13 (d, *J* = 8.4 Hz, 2H), 4.06 (s, 1H), 3.33 (s, 3H), 3.29 (t, *J* = 7.2 Hz, 2H), 3.04 (t, *J* = 7.2 Hz, 2H), 1.23 (s, 6H).

^{13}C NMR (151 MHz, $CDCl_3$) δ (ppm): 197.5, 176.6, 148.3, 139.9, 136.3, 131.6, 130.2, 129.3, 128.6, 120.0, 73.1, 40.7, 40.2, 29.3, 28.7.

HRMS (ESI-TOF) *m/z*: $[M + Na]^+$ Calcd for $C_{20}H_{22}{}^{79}BrNO_3Na^+$ 426.0676, $C_{20}H_{22}{}^{81}BrNO_3Na^+$ 428.0655; Found 426.0669, 428.0645.

2-hydroxy-*N*,2-dimethyl-*N*-(4-(3-(*p*-tolyl)propanoyl)phenyl)propanamide 3c

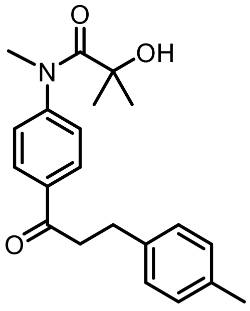

Prepared according to the ***General Procedure B*** to afford **3c** (24.1 mg) in 71% yield as white solid, m.p. = 91.1 – 93.2 ºC.

*NMR and HRMS data for the product **3c***:

^{1}H NMR (600 MHz, $CDCl_3$) δ (ppm): 8.00 (d, *J* = 8.4 Hz, 2H), 7.33 (d, *J* = 9.0 Hz, 2H), 7.14 (d, *J* = 7.8 Hz, 2H), 7.11 (d, *J* = 7.8 Hz, 2H), 4.11 (s, 1H), 3.33 (s, 3H), 3.29 (t, *J* = 7.8 Hz, 2H), 3.04 (t, *J* = 7.8 Hz, 2H), 2.32 (s, 3H), 1.22 (s, 6H).

^{13}C NMR (151 MHz, CDCl$_3$) δ (ppm): 198.0, 176.6, 148.1, 137.8, 136.4, 135.7, 129.3, 129.2, 128.6, 128.3, 73.0, 40.7, 40.7, 29.6, 28.6, 21.0.

HRMS (ESI-TOF) *m/z*: [M + Na]$^+$ Calcd for $C_{21}H_{25}NO_3Na^+$ 362.1727; Found 362.1735.

<u>*N*-(4-(3-(3-fluorophenyl)propanoyl)phenyl)-2-hydroxy-*N*,2-dimethylpropanamide 3d</u>

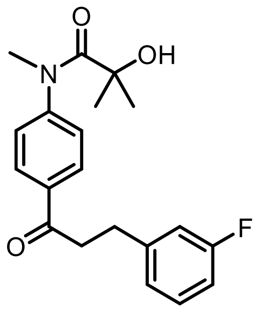

Prepared according to the ***General Procedure B*** to afford **3d** (24.0 mg) in 70% yield as colorless oil.

*NMR and HRMS data for the product **3d***:

^{1}H NMR (600 MHz, CDCl$_3$) δ (ppm): 8.00 (d, *J* = 7.8 Hz, 2H), 7.34 (d, *J* = 8.4 Hz, 2H), 7.27 – 7.23 (m, 1H), 7.02 (d, *J* = 7.2 Hz, 1H), 6.95 (d, *J* = 9.6 Hz, 1H), 6.90 (td, *J* = 8.4, 2.4 Hz, 1H), 4.08 (s, 1H), 3.33 (s, 3H), 3.31 (t, *J* = 7.8 Hz, 2H), 3.08 (t, *J* = 7.8 Hz, 2H), 1.23 (s, 6H).

^{13}C NMR (151 MHz, CDCl$_3$) δ (ppm): 197.5, 176.6, 162.9 (C-F, $^1J_{C\text{-}F}$ = 245.8 Hz), 148.2, 143.5 (C-F, $^3J_{C\text{-}F}$ = 7.2 Hz), 136.2, 130.0 (C-F, $^3J_{C\text{-}F}$ = 8.8 Hz), 129.3, 128.6, 124.1, 115.3 (C-F, $^2J_{C\text{-}F}$ = 21.4 Hz), 113.1 (C-F, $^2J_{C\text{-}F}$ = 21.6 Hz), 73.1, 40.7, 40.1, 29.6, 28.6.

^{19}F NMR (564 MHz, CDCl$_3$) δ (ppm): -113.19 – -113.24 (m, 1F).

HRMS (ESI-TOF) *m/z*: [M + Na]$^+$ Calcd for $C_{20}H_{22}FNO_3Na^+$ 366.1476; Found 366.1485.

<u>2-hydroxy-*N*,2-dimethyl-*N*-(4-(3-(*m*-tolyl)propanoyl)phenyl)propanamide 3e</u>

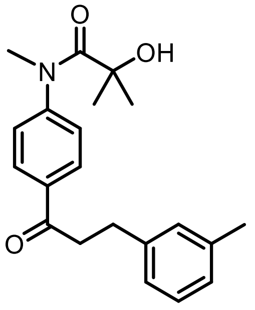

Prepared according to the ***General Procedure B*** to afford **3e** (26.1 mg) in 77% yield as colorless oil.

*NMR and HRMS data for the product **3e***:

^{1}H NMR (600 MHz, $CDCl_3$) δ (ppm): 8.01 (d, J = 8.4 Hz, 2H), 7.33 (d, J = 7.8 Hz, 2H), 7.19 (t, J = 7.8 Hz, 1H), 7.07 (s, 1H), 7.05 (d, J = 7.2 Hz, 1H), 7.03 (d, J = 7.8 Hz, 1H), 4.09 (brs, 1H), 3.33 (s, 3H), 3.30 (t, J = 7.8 Hz, 2H), 3.04 (t, J = 7.8 Hz, 2H), 2.33 (s, 3H), 1.23 (s, 6H).

^{13}C NMR (151 MHz, $CDCl_3$) δ (ppm): 198.0, 176.6, 148.1, 140.9, 138.1, 136.4, 129.3, 129.2, 128.6, 128.4, 127.0, 125.3, 73.1, 40.7, 40.6, 29.9, 28.7, 21.4.

HRMS (ESI-TOF) *m/z*: $[M + Na]^+$ Calcd for $C_{21}H_{25}NO_3Na^+$ 362.1727; Found 362.1732.

***N*-(4-(3-(2-fluorophenyl)propanoyl)phenyl)-2-hydroxy-*N*,2-dimethylpropanamide 3f**

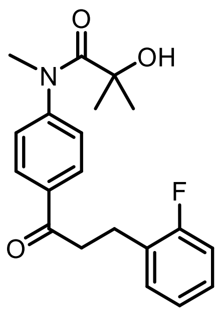

Prepared according to the ***General Procedure B*** to afford **3f** (29.2 mg) in 85% yield as colorless oil.

*NMR and HRMS data for the product **3f***:

^{1}H NMR (600 MHz, $CDCl_3$) δ (ppm): 8.01 (d, J = 9.0 Hz, 2H), 7.33 (d, J = 9.0 Hz, 2H), 7.29 – 7.24 (m, 1H), 7.22 – 7.18 (m, 1H), 7.07 (t, J = 7.8 Hz, 1H), 7.02 (t, J = 9.0 Hz, 1H), 4.10 (s, 1H), 3.32 (s, 3H), 3.31 (t, J = 7.2 Hz, 5H), 3.10 (t, J = 7.2 Hz, 2H), 1.22 (s, 6H).

^{13}C NMR (151 MHz, $CDCl_3$) δ (ppm): 197.7, 176.6, 161.2 (C-F, $^1J_{C-F}$ = 245.8 Hz), 148.2, 136.3, 130.9 (C-F, $^3J_{C-F}$ = 4.4 Hz), 129.3, 128.59, 128.55, 128.1 (C-F, $^2J_{C-F}$ = 7.2 Hz), 124.1 (C-F, $^3J_{C-F}$ = 2.9 Hz), 115.3 (C-F, $^2J_{C-F}$ = 21.6 Hz), 73.1, 40.7, 38.9, 28.7, 23.9.

^{19}F NMR (564 MHz, $CDCl_3$) δ (ppm): -118.30 – -118.35 (m, 1F).

HRMS (ESI-TOF) *m/z*: $[M + Na]^+$ Calcd for $C_{20}H_{22}FNO_3Na^+$ 366.1476; Found 366.1468.

2-hydroxy-*N*-(4-(3-(2-methoxyphenyl)propanoyl)phenyl)-*N*,2-dimethylpropanamide 3g

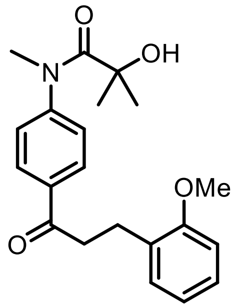

Prepared according to the ***General Procedure B*** to afford **3g** (22.0 mg) in 62% yield as colorless oil.

*NMR and HRMS data for the product **3g***:

^{1}H NMR (600 MHz, $CDCl_3$) δ (ppm): 8.02 (d, J = 8.4 Hz, 2H), 7.33 (d, J = 8.4 Hz, 2H), 7.23 – 7.19 (m, 2H), 6.89 (t, J = 7.8 Hz, 1H), 6.86 (d, J = 7.8 Hz, 1H), 4.12 (brs, 1H), 3.84 (s, 3H), 3.33 (s, 3H), 3.27 (t, J = 7.8 Hz, 2H), 3.05 (t, J = 7.8 Hz, 2H), 1.22 (s, 6H).

^{13}C NMR (151 MHz, $CDCl_3$) δ (ppm): 198.7, 176.7, 157.5, 147.9, 136.6, 130.2, 129.4, 129.1, 128.6, 127.6, 120.6, 110.2, 73.1, 55.2, 40.8, 39.0, 28.7, 25.7.

HRMS (ESI-TOF) *m/z*: $[M + Na]^+$ Calcd for $C_{21}H_{25}NO_4Na^+$ 378.1676; Found 378.1684.

2-hydroxy-*N*,2-dimethyl-*N*-(4-(3-(methylthio)propanoyl)phenyl)propanamide 3h

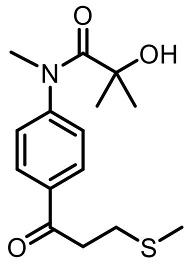

Prepared according to the ***General Procedure B*** to afford **3h** (28.4 mg) in 96% yield as white solid, m.p. = 103.7 – 105.2 °C.

*NMR and HRMS data for the product **3h***:

^{1}H NMR (600 MHz, $CDCl_3$) δ (ppm): 8.01 (d, J = 8.4 Hz, 2H), 7.35 (d, J = 8.4 Hz, 2H), 4.04 (s, 1H), 3.33 (s, 3H), 3.29 (t, J = 7.2 Hz, 2H), 2.91 (t, J = 7.2 Hz, 2H), 2.16 (s, 3H), 1.23 (s, 6H).

^{13}C NMR (151 MHz, $CDCl_3$) δ (ppm): 197.1, 176.6, 148.4, 136.1, 129.3, 128.6, 73.1, 40.7, 38.7, 28.7, 28.3, 16.0.

HRMS (ESI-TOF) *m/z*: $[M + Na]^+$ Calcd for $C_{15}H_{21}NO_3SNa^+$ 318.1134; Found 318.1132.

***N*-(4-hexanoylphenyl)-2-hydroxy-*N*,2-dimethylpropanamide 3i**

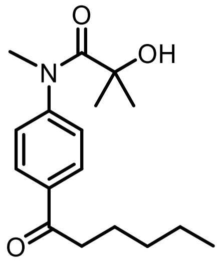

Prepared according to the ***General Procedure B*** to afford **3i** (24.2 mg) in 83% yield as colorless oil.

*NMR and HRMS data for the product **3i***:

^{1}H NMR (600 MHz, CDCl$_3$) δ (ppm): 8.01 (d, *J* = 8.4 Hz, 2H), 7.34 (d, *J* = 8.4 Hz, 2H), 4.11 (s, 1H), 3.33 (s, 3H), 2.97 (t, *J* = 7.8 Hz, 2H), 1.77 – 1.73 (m, 2H), 1.40 – 1.34 (m, 4H), 1.23 (s, 6H), 0.92 (t, *J* = 7.2 Hz, 3H).

^{13}C NMR (151 MHz, CDCl$_3$) δ (ppm): 199.2, 176.7, 147.9, 136.7, 129.3, 128.6, 73.1, 40.8, 38.7, 31.5, 28.7, 23.9, 22.5, 13.9.

HRMS (ESI-TOF) *m/z*: $[M + Na]^+$ Calcd for $C_{17}H_{25}NO_3Na^+$ 314.1727; Found 314.1737.

***N*-(4-heptanoylphenyl)-2-hydroxy-*N*,2-dimethylpropanamide 3j**

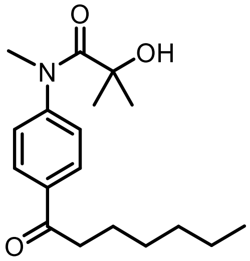

Prepared according to the ***General Procedure B*** to afford **3j** (19.5 mg) in 64% yield as white solid, m.p. = 61.4 – 63.3 ºC.

*NMR and HRMS data for the product **3j***:

^{1}H NMR (600 MHz, CDCl$_3$) δ (ppm): 8.01 (d, *J* = 8.4 Hz, 2H), 7.34 (d, *J* = 8.4 Hz, 2H), 4.11 (s, 1H), 3.33 (s, 3H), 2.96 (t, *J* = 7.8 Hz, 2H), 1.76 – 1.71 (m, 2H), 1.41 – 1.36 (m, 2H), 1.33 – 1.30 (m, 4H), 1.22 (s, 6H), 0.89 (t, *J* = 7.2 Hz, 3H).

^{13}C NMR (151 MHz, CDCl$_3$) δ (ppm): 199.2, 176.7, 147.9, 136.7, 129.3, 128.6, 73.1, 40.7, 38.7, 31.6, 29.0, 28.7, 24.1, 22.5, 14.0.

HRMS (ESI-TOF) *m/z*: $[M + Na]^+$ Calcd for $C_{18}H_{27}NO_3Na^+$ 328.1884; Found 328.1889.

<u>*N*-(4-acetylphenyl)-2-hydroxy-*N*,2-dimethylpropanamide 3k</u>

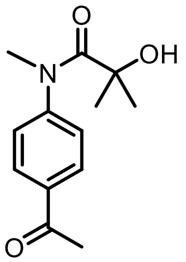

Prepared according to the ***General Procedure B*** to afford **3k** (10.1 mg) in 43% yield as white solid, m.p. = 92.1 – 94.9 ºC.

*NMR and HRMS data for the product **3k***:

^{1}H NMR (600 MHz, CDCl$_3$) δ (ppm): 8.01 (d, J = 9.0 Hz, 2H), 7.35 (d, J = 8.4 Hz, 2H), 4.11 (s, 1H), 3.33 (s, 3H), 2.63 (s, 3H), 1.23 (s, 6H).

^{13}C NMR (151 MHz, CDCl$_3$) δ (ppm): 196.8, 176.6, 148.2, 136.6, 129.6, 128.6, 73.1, 40.7, 28.6, 26.7.

HRMS (ESI-TOF) *m/z*: $[M + Na]^+$ Calcd for $C_{13}H_{17}NO_3Na^+$ 258.1101; Found 258.1105.

<u>*N*-(4-(cyclobutanecarbonyl)phenyl)-2-hydroxy-*N*,2-dimethylpropanamide 3l</u>

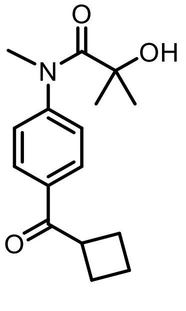

Prepared according to the ***General Procedure B*** to afford **3l** (19.3 mg) in 70% yield as white solid, m.p. = 90.5 – 94.9 ºC.

*NMR and HRMS data for the product **3l***:

^{1}H NMR (600 MHz, $CDCl_3$) δ (ppm): 7.94 (d, J = 8.4 Hz, 2H), 7.32 (d, J = 8.4 Hz, 2H), 4.11 (s, 1H), 4.01 – 3.95 (m, 1H), 3.32 (s, 3H), 2.46 – 2.39 (m, 2H), 2.34 – 2.28 (m, 2H), 2.14 – 2.06 (m, 1H), 1.96 – 1.90 (m, 1H), 1.22 (s, 6H).

^{13}C NMR (151 MHz, $CDCl_3$) δ (ppm): 199.7, 176.7, 147.9, 135.2, 129.5, 128.6, 73.0, 42.2, 40.7, 28.6, 25.0, 18.1.

HRMS (ESI-TOF) *m/z*: $[M + Na]^+$ Calcd for $C_{16}H_{21}NO_3Na^+$ 298.1414; Found 298.1423.

***N*-(4-(cyclopentanecarbonyl)phenyl)-2-hydroxy-*N*,2-dimethylpropanamide 3m**

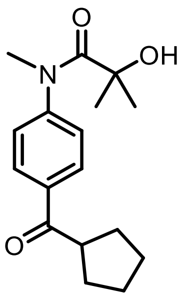

Prepared according to the ***General Procedure B*** to afford **3m** (19.4 mg) in 67% yield as white solid, m.p. = 100.3 – 103.2 ºC.

*NMR and HRMS data for the product **3m***:

^{1}H NMR (600 MHz, $CDCl_3$) δ (ppm): 8.02 (d, J = 8.4 Hz, 2H), 7.34 (d, J = 8.4 Hz, 2H), 4.13 (s, 1H), 3.72 – 3.66 (m, 1H), 3.33 (s, 3H), 1.97 – 1.88 (m, 4H), 1.77 – 1.63 (m, 4H), 1.22 (s, 6H).

^{13}C NMR (151 MHz, $CDCl_3$) δ (ppm): 201.5, 176.7, 147.8, 136.5, 129.7, 128.5, 73.0, 46.5, 40.7, 29.9, 28.7, 26.3.

HRMS (ESI-TOF) *m/z*: $[M + H]^+$ Calcd for $C_{17}H_{24}NO_3^+$ 290.1751; Found 290.1760.

<u>*tert*-butyl 4-(4-(2-hydroxy-*N*,2-dimethylpropanamido)benzoyl)piperidine-1-carboxylate 3n</u>

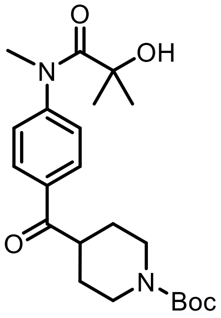

Prepared according to the ***General Procedure B*** to afford **3n** (24.3 mg) in 60% yield as white solid, m.p. = 129.1 – 132.2 ºC.

*NMR and HRMS data for the product **3n***:

^{1}H NMR (600 MHz, $CDCl_3$) δ (ppm): 7.98 (d, *J* = 8.4 Hz, 2H), 7.35 (d, *J* = 8.4 Hz, 2H), 4.26 – 3.99 (m, 3H), 3.38 (tt, *J* = 10.8, 3.6 Hz, 1H), 3.33 (s, 3H), 3.01 – 2.80 (m, 2H), 1.90 – 1.75 (m, 2H), 1.74 – 1.60 (m, 2H), 1.45 (s, 9H), 1.23 (s, 6H).

^{13}C NMR (151 MHz, $CDCl_3$) δ (ppm): 200.8, 176.6, 154.6, 148.2, 135.3, 129.5, 128.6, 79.7, 73.1, 43.6, 42.9, 40.7, 28.6, 28.4, 28.3.

HRMS (ESI-TOF) *m/z*: $[M + Na]^+$ Calcd for $C_{22}H_{32}N_2O_5Na^+$ 427.2203; Found 427.2204

<u>*N*-(4-(3-(4-(*tert*-butyl)phenyl)-2-methylpropanoyl)phenyl)-2-hydroxy-*N*,2-dimethylpropanamide 3o</u>

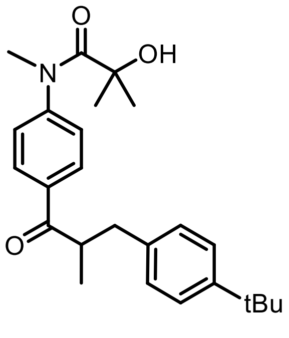

Prepared according to the ***General Procedure B*** to afford **3o** (22.9 mg) in 58% yield as colorless oil.

*NMR and HRMS data for the product **3o***:

^{1}H NMR (600 MHz, $CDCl_3$) δ (ppm): 7.95 (d, *J* = 8.4 Hz, 2H), 7.31 (d, *J* = 8.4 Hz, 2H), 7.28 (d, *J* = 8.4 Hz, 2H), 7.12 (d, *J* = 7.8 Hz, 2H), 4.10 (s, 1H), 3.74 – 3.67 (m, 1H), 3.32 (s, 3H),

3.12 (dd, J = 13.8, 7.2 Hz, 1H), 2.69 (dd, J = 13.8, 7.8 Hz, 1H), 1.28 (s, 9H), 1.23 – 1.21 (m, 9H).

^{13}C NMR (151 MHz, CDCl$_3$) δ (ppm): 202.7, 176.6, 149.2, 147.9, 136.5, 136.1, 129.5, 128.7, 128.5, 125.3, 73.1, 43.0, 40.7, 38.9, 34.3, 31.3, 28.7, 17.4.

HRMS (ESI-TOF) *m/z*: [M + Na]$^+$ Calcd for $C_{25}H_{33}NO_3Na^+$ 418.2353; Found 418.2363.

N-(4-benzoylphenyl)-2-hydroxy-N,2-dimethylpropanamide 3p

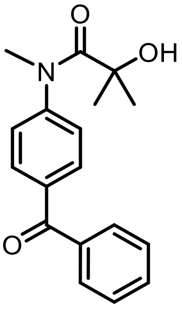

Prepared according to the ***General Procedure B*** to afford **3p** (27.1 mg) in 91% yield as colorless oil.

*NMR and HRMS data for the product **3p***:

^{1}H NMR (600 MHz, CDCl$_3$) δ (ppm): 7.87 (d, J = 9.0 Hz, 2H), 7.80 (d, J = 8.4 Hz, 2H), 7.62 (t, J = 7.8 Hz, 1H), 7.51 (t, J = 7.8 Hz, 2H), 7.37 (d, J = 8.4 Hz, 2H), 4.14 (s, 1H), 3.37 (s, 3H), 1.27 (s, 6H).

^{13}C NMR (151 MHz, CDCl$_3$) δ (ppm): 195.4, 176.7, 147.6, 137.3, 137.0, 132.8, 131.2, 129.9, 128.4, 128.3, 73.1, 40.8, 28.7.

HRMS (ESI-TOF) *m/z*: [M + Na]$^+$ Calcd for $C_{18}H_{19}NO_3Na^+$ 320.1257; Found 320.1267.

N-(4-(4-bromobenzoyl)phenyl)-2-hydroxy-N,2-dimethylpropanamide 3q

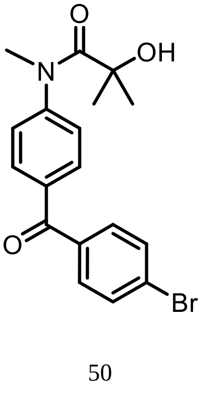

Prepared according to the ***General Procedure B*** to afford **3q** (33.1 mg) in 88% yield as white solid, m.p. = 112.9 – 115.0 °C.

*NMR and HRMS data for the product **3q***:

^{1}H NMR (600 MHz, CDCl$_3$) δ (ppm): 7.83 (d, J = 9.0 Hz, 2H), 7.67 (d, J = 9.0 Hz, 2H), 7.64 (d, J = 8.4 Hz, 2H), 7.37 (d, J = 8.4 Hz, 2H), 4.07 (brs, 1H), 3.37 (s, 3H), 1.28 (s, 6H).

^{13}C NMR (151 MHz, CDCl$_3$) δ (ppm): 194.3, 176.6, 147.9, 136.7, 135.7, 131.8, 131.4, 131.1, 128.3, 127.9, 73.1, 40.7, 28.7.

HRMS (ESI-TOF) *m/z*: [M + Na]$^+$ Calcd for $C_{18}H_{18}{}^{79}BrNO_3Na^+$ 398.0362, $C_{18}H_{18}{}^{81}BrNO_3Na^+$ 400.0342; Found 398.0370, 400.0351.

<u>*N*-(4-(4-fluorobenzoyl)phenyl)-2-hydroxy-*N*,2-dimethylpropanamide 3r</u>

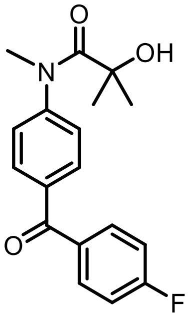

Prepared according to the ***General Procedure B*** to afford **3r** (24.0 mg) in 76% yield as white solid, m.p. = 105.5 – 108.4 °C.

*NMR and HRMS data for the product **3r***:

^{1}H NMR (600 MHz, CDCl$_3$) δ (ppm): 7.86 – 7.83 (m, 4H), 7.38 (d, J = 9.0 Hz, 2H), 7.19 (t, J = 8.4 Hz, 2H), 4.09 (s, 1H), 3.37 (s, 3H), 1.28 (s, 6H).

^{13}C NMR (151 MHz, CDCl$_3$) δ (ppm): 193.9, 176.7, 165.6 (C-F, $^{1}J_{C\text{-}F}$ = 254.4 Hz), 147.7, 137.1, 133.2 (C-F, $^{4}J_{C\text{-}F}$ = 2.9 Hz), 132.6 (C-F, $^{3}J_{C\text{-}F}$ = 8.6 Hz), 131.1, 128.3, 115.7 (C-F, $^{2}J_{C\text{-}F}$ = 21.7 Hz), 73.1, 40.8, 28.7.

^{19}F NMR (564 MHz, CDCl$_3$) δ (ppm): -104.80 – -104.95 (m, 1F).

HRMS (ESI-TOF) *m/z*: [M + Na]$^+$ Calcd for $C_{18}H_{18}FNO_3Na^+$ 338.1163; Found 338.1164.

N-(4-(4-cyanobenzoyl)phenyl)-2-hydroxy-N,2-dimethylpropanamide 3s

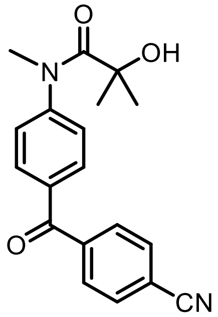

Prepared according to the ***General Procedure B*** to afford **3s** (23.9 mg) in 74% yield as white solid, m.p. = 129.8 – 132.6 °C.

*NMR and HRMS data for the product **3s***:

^{1}H NMR (600 MHz, $CDCl_3$) δ (ppm): 7.88 (d, J = 7.8 Hz, 2H), 7.85 (d, J = 8.4 Hz, 2H), 7.81 (d, J = 8.4 Hz, 2H), 7.40 (d, J = 7.8 Hz, 2H), 3.95 (brs, 1H), 3.38 (s, 3H), 1.30 (s, 6H).

^{13}C NMR (151 MHz, $CDCl_3$) δ (ppm): 193.7, 176.6, 148.6, 140.6, 135.7, 132.3, 131.3, 130.1, 128.4, 117.8, 116.0, 73.2, 40.7, 28.7.

HRMS (ESI-TOF) *m/z*: [M + H]$^+$ Calcd for $C_{19}H_{19}N_2O_3^+$ 323.1390; Found 323.1390.

2-hydroxy-N,2-dimethyl-N-(4-(4-(trifluoromethyl)benzoyl)phenyl)propanamide 3t

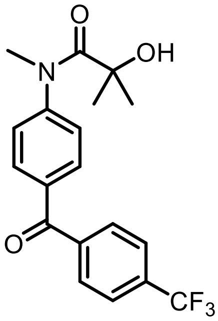

Prepared according to the ***General Procedure B*** to afford **3t** (19.4 mg) in 53% yield as colorless oil.

*NMR and HRMS data for the product **3t***:

^{1}H NMR (600 MHz, $CDCl_3$) δ (ppm): 7.90 (d, J = 7.8 Hz, 2H), 7.87 (d, J = 8.4 Hz, 2H), 7.78 (d, J = 7.8 Hz, 2H), 7.40 (d, J = 8.4 Hz, 2H), 4.01 (s, 1H), 3.38 (s, 3H), 1.30 (s, 6H).

^{13}C NMR (151 MHz, $CDCl_3$) δ (ppm): 194.2, 176.6, 148.4, 140.1, 136.2, 134.1 (C-F, $^2J_{C\text{-}F}$ = 33.4 Hz), 131.3, 130.1, 128.4, 125.5 (C-F, $^3J_{C\text{-}F}$ = 4.4 Hz), 123.5 (C-F, $^1J_{C\text{-}F}$ = 273.3 Hz), 73.2, 40.7, 28.7.

^{19}F NMR (564 MHz, $CDCl_3$) δ (ppm): -62.96 (s, 1F).

HRMS (ESI-TOF) *m/z*: $[M + Na]^+$ Calcd for $C_{19}H_{18}F_3NO_3Na^+$ 388.1131; Found 388.1141.

2-hydroxy-*N*,2-dimethyl-N-(4-(4-methylbenzoyl)phenyl)propanamide 3u

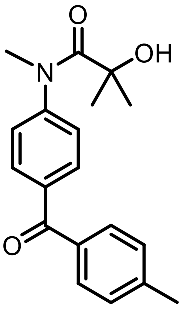

Prepared according to the ***General Procedure B*** to afford **3u** (19.6 mg) in 63% yield as white solid, m.p. = 96.6 – 98.2°C.

*NMR and HRMS data for the product **3u***:

^{1}H NMR (600 MHz, $CDCl_3$) δ (ppm): 7.84 (d, *J* = 8.4 Hz, 2H), 7.71 (d, *J* = 8.4 Hz, 2H), 7.36 (d, *J* = 8.4 Hz, 2H), 7.30 (d, *J* = 7.8 Hz, 2H), 4.16 (s, 1H), 3.37 (s, 3H), 2.45 (s, 3H), 1.27 (s, 6H).

^{13}C NMR (151 MHz, $CDCl_3$) δ (ppm): 195.1, 176.7, 147.3, 143.8, 137.7, 134.3, 131.1, 130.2, 129.2, 128.2, 73.1, 40.8, 28.7, 21.7.

HRMS (ESI-TOF) *m/z*: $[M + Na]^+$ Calcd for $C_{19}H_{21}NO_3Na^+$ 334.1414; Found 334.1421

methyl 4-(4-(2-hydroxy-*N*,2-dimethylpropanamido)benzoyl)benzoate 3v

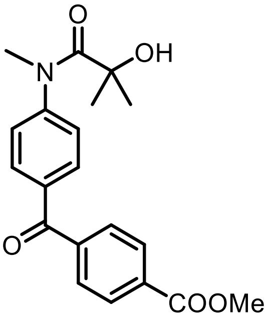

Prepared according to the ***General Procedure B*** to afford **3v** (22.7 mg) in 64% yield as white solid, m.p. = 119.8 – 121.3 °C.

*NMR and HRMS data for the product **3v***:

^{1}H NMR (600 MHz, CDCl$_3$) δ (ppm): 8.16 (d, J = 8.4 Hz, 2H), 7.87 (d, J = 9.0 Hz, 2H), 7.83 (d, J = 8.4 Hz, 2H), 7.39 (d, J = 8.4 Hz, 2H), 4.04 (s, 1H), 3.96 (s, 3H), 3.37 (s, 3H), 1.28 (s, 6H).

^{13}C NMR (151 MHz, CDCl$_3$) δ (ppm): 194.7, 176.6, 166.1, 148.2, 140.7, 136.5, 133.6, 131.3, 129.7, 129.6, 128.4, 73.2, 52.5, 40.7, 28.7.

HRMS (ESI-TOF) *m/z*: $[M + Na]^+$ Calcd for $C_{20}H_{21}NO_5Na^+$ 378.1312; Found 378.1322.

***N*-(4-(3-fluorobenzoyl)phenyl)-2-hydroxy-*N*,2-dimethylpropanamide 3w**

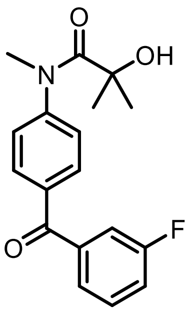

Prepared according to the ***General Procedure B*** to afford **3w** (24.9 mg) in 79% yield as colorless oil.

*NMR and HRMS data for the product **3w***:

^{1}H NMR (600 MHz, CDCl$_3$) δ (ppm): 7.86 (d, J = 7.8 Hz, 2H), 7.57 (d, J = 7.8 Hz, 1H), 7.51 – 7.47 (m, 2H), 7.39 (d, J = 8.4 Hz, 2H), 7.32 (td, J = 8.4, 3.6 Hz, 1H), 4.08 (brs, 1H), 3.37 (s, 3H), 1.28 (s, 6H).

^{13}C NMR (151 MHz, CDCl$_3$) δ (ppm): 193.9, 176.7, 162.5 (C-F, $^1J_{C\text{-}F}$ = 248.5 Hz), 148.0, 139.1 (C-F, $^3J_{C\text{-}F}$ = 5.7 Hz), 136.6, 131.2, 130.2 (C-F, $^3J_{C\text{-}F}$ = 8.6 Hz), 128.4, 125.7 (C-F, $^4J_{C\text{-}F}$ = 2.9 Hz), 119.8 (C-F, $^2J_{C\text{-}F}$ = 21.7 Hz), 116.6 (C-F, $^2J_{C\text{-}F}$ = 23.1 Hz), 73.1, 40.8, 28.7.

^{19}F NMR (564 MHz, CDCl$_3$) δ (ppm): -111.34 – -111.39 (m, 1F).

HRMS (ESI-TOF) *m/z*: $[M + Na]^+$ Calcd for $C_{18}H_{18}FNO_3Na^+$ 338.1163; Found 338.1166.

N-(4-(3-chlorobenzoyl)phenyl)-2-hydroxy-N,2-dimethylpropanamide 3x

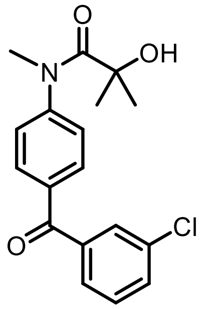

Prepared according to the ***General Procedure B*** to afford **3x** (15.9 mg) in 48% yield as colorless oil.

*NMR and HRMS data for the product **3x***:

^{1}H NMR (600 MHz, $CDCl_3$) δ (ppm): 7.86 (d, J = 8.4 Hz, 2H), 7.78 (s, 1H), 7.66 (d, J = 7.8 Hz, 1H), 7.59 (d, J = 7.8 Hz, 1H), 7.45 (t, J = 7.8 Hz, 1H), 7.39 (d, J = 7.8 Hz, 2H), 4.05 (s, 1H), 3.38 (s, 3H), 1.29 (s, 6H).

^{13}C NMR (151 MHz, $CDCl_3$) δ (ppm): 193.9, 176.7, 148.1, 138.7, 136.5, 134.8, 132.7, 131.2, 129.79, 129.76, 128.4, 128.0, 73.2, 40.8, 28.7.

HRMS (ESI-TOF) *m/z*: $[M + Na]^+$ Calcd for $C_{18}H_{18}{}^{35}ClNO_3Na^+$ 354.0867, $C_{18}H_{18}{}^{37}ClNO_3Na^+$ 356.0838; Found 354.0870, 356.0848.

N-(4-(3-bromobenzoyl)phenyl)-2-hydroxy-N,2-dimethylpropanamide 3y

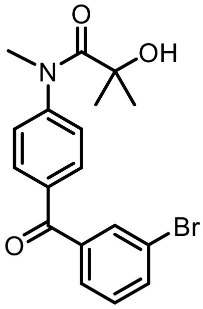

Prepared according to the ***General Procedure B*** to afford **3y** (23.7 mg) in 63% yield as colorless oil.

*NMR and HRMS data for the product **3y***:

^{1}H NMR (600 MHz, $CDCl_3$) δ (ppm): 7.93 (s, 1H), 7.85 (d, J = 9.0 Hz, 2H), 7.74 (d, J = 8.4 Hz, 1H), 7.70 (d, J = 7.2 Hz, 1H), 7.36 – 7.41 (3H), 3.38 (s, 3H), 1.28 (s, 6H).

^{13}C NMR (151 MHz, $CDCl_3$) δ (ppm): 193.8, 176.7, 148.1, 138.9, 136.5, 135.6, 132.6, 131.2, 130.0, 128.44, 128.38, 122.8, 73.2, 40.8, 28.7.

HRMS (ESI-TOF) *m/z*: $[M + Na]^+$ Calcd for $C_{18}H_{18}{}^{79}BrNO_3Na^+$ 398.0362, $C_{18}H_{18}{}^{81}BrNO_3Na^+$ 400.0342; Found 398.0370, 400.0348.

2-hydroxy-*N*-(4-(3-methoxybenzoyl)phenyl)-*N*,2-dimethylpropanamide 3z

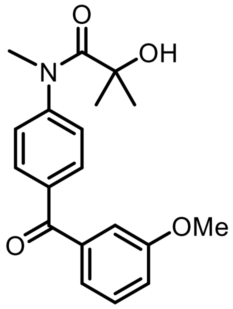

Prepared according to the ***General Procedure B*** to afford **3z** (23.9 mg) in 73% yield as colorless oil.

*NMR and HRMS data for the product **3z**:*

^{1}H NMR (600 MHz, $CDCl_3$) δ (ppm): 7.86 (d, *J* = 8.4 Hz, 2H), 7.40 (t, *J* = 7.8 Hz, 1H), 7.37 – 7.35 (m, 3H), 7.31 (d, *J* = 7.8 Hz, 1H), 7.16 (d, *J* = 8.4 Hz, 1H), 4.13 (s, 1H), 3.86 (s, 3H), 3.36 (s, 3H), 1.26 (s, 6H).

^{13}C NMR (151 MHz, $CDCl_3$) δ (ppm): 195.1, 176.7, 159.7, 147.6, 138.3, 137.3, 131.2, 129.4, 128.2, 122.7, 119.1, 114.4, 73.1, 55.5, 40.8, 28.7.

HRMS (ESI-TOF) *m/z*: $[M + Na]^+$ Calcd for $C_{19}H_{21}NO_4Na^+$ 350.1363; Found 350.1361

***N*-(4-(2-fluorobenzoyl)phenyl)-2-hydroxy-*N*,2-dimethylpropanamide 3aa**

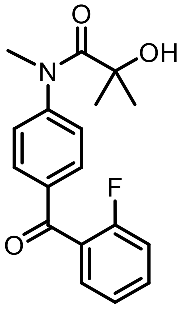

Prepared according to the ***General Procedure B*** to afford **3aa** (30.9 mg) in 98% yield as white solid, m.p. = 82.9 – 84.5 ºC.

*NMR and HRMS data for the product **3aa***:

^{1}H NMR (600 MHz, $CDCl_3$) δ (ppm): 7.88 (d, J = 8.4 Hz, 2H), 7.59 – 7.54 (m, 2H), 7.35 (d, J = 7.8 Hz, 2H), 7.29 (t, J = 7.8 Hz, 1H), 7.17 (t, J = 9.0 Hz, 1H), 4.10 (s, 1H), 3.35 (s, 3H), 1.24 (s, 6H).

^{13}C NMR (151 MHz, $CDCl_3$) δ (ppm): 192.1, 176.7, 160.1 (C-F, $^1J_{C\text{-}F}$ = 251.6 Hz), 148.3, 137.1, 133.6 (C-F, $^3J_{C\text{-}F}$ = 5.6 Hz), 130.9, 130.8, 128.4, 126.4 (C-F, $^2J_{C\text{-}F}$ = 14.5 Hz), 124.5 (C-F, $^3J_{C\text{-}F}$ = 2.9 Hz), 116.3 (C-F, $^2J_{C\text{-}F}$ = 21.6 Hz), 73.1, 40.7, 28.7.

^{19}F NMR (564 MHz, $CDCl_3$) δ (ppm): -110.20 – -110.25 (m, 1F).

HRMS (ESI-TOF) *m/z*: $[M + Na]^+$ Calcd for $C_{18}H_{18}FNO_3Na^+$ 338.1163; Found 338.1172.

2-hydroxy-*N*-(4-(2-hydroxybenzoyl)phenyl)-*N*,2-dimethylpropanamide 3ab

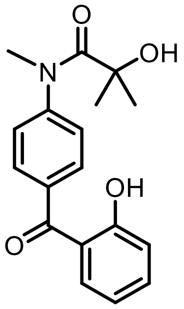

Prepared according to the ***General Procedure B*** to afford **3ab** (20.4 mg) in 65% yield as colorless oil.

*NMR and HRMS data for the product **3ab***:

^{1}H NMR (600 MHz, $CDCl_3$) δ (ppm): 11.88 (s, 1H), 7.75 (d, J = 8.4 Hz, 2H), 7.56 – 7.53 (m, 2H), 7.40 (d, J = 8.4 Hz, 2H), 7.09 (d, J = 8.4 Hz, 1H), 6.91 (t, J = 7.8 Hz, 1H), 4.10 (s, 1H), 3.38 (s, 3H), 1.30 (s, 6H).

^{13}C NMR (151 MHz, $CDCl_3$) δ (ppm): 200.2, 176.7, 163.3, 147.3, 137.6, 136.7, 133.2, 130.4, 128.3, 118.9, 118.8, 118.7, 73.1, 40.8, 28.7.

HRMS (ESI-TOF) *m/z*: $[M + Na]^+$ Calcd for $C_{18}H_{19}NO_4Na^+$ 336.1206; Found 336.1215.

<u>*N*-(4-(2-fluoro-4-methoxybenzoyl)phenyl)-2-hydroxy-*N*,2-dimethylpropanamide 3ac</u>

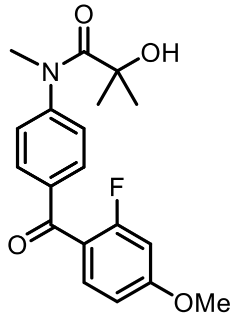

Prepared according to the ***General Procedure B*** to afford **3ac** (29.4 mg) in 85% yield as white solid, m.p. = 78.5 – 79.8 ºC.

*NMR and HRMS data for the product **3ac***:

^{1}H NMR (600 MHz, CDCl$_3$) δ (ppm): 7.84 (d, *J* = 8.4 Hz, 2H), 7.61 (t, *J* = 8.4 Hz, 1H), 7.34 (d, *J* = 8.4 Hz, 2H), 6.81 (d, *J* = 8.4 Hz, 1H), 6.66 (d, *J* = 11.4 Hz, 1H), 4.17 (s, 1H), 3.88 (s, 3H), 3.35 (s, 3H), 1.23 (s, 6H).

^{13}C NMR (151 MHz, CDCl$_3$) δ (ppm): 191.4, 176.7, 164.4 (C-F, $^3J_{C-F}$ = 10.1 Hz), 162.0 (C-F, $^1J_{C-F}$ = 254.4 Hz), 147.7, 138.2, 132.7 (C-F, $^3J_{C-F}$ = 4.4 Hz), 130.6, 128.3, 118.7 (C-F, $^2J_{C-F}$ = 14.3 Hz), 110.6, 101.9 (C-F, $^2J_{C-F}$ = 26.1 Hz), 73.1, 55.9, 40.7, 28.7.

^{19}F NMR (564 MHz, CDCl$_3$) δ (ppm): -105.86 (t, *J* = 9.9 Hz, 1F).

HRMS (ESI-TOF) *m/z*: [M + Na]$^+$ Calcd for $C_{19}H_{20}FNO_4Na^+$ 368.1269; Found 368.1278.

<u>*N*-(4-(2-bromo-4-chlorobenzoyl)phenyl)-2-hydroxy-*N*,2-dimethylpropanamide 3ad</u>

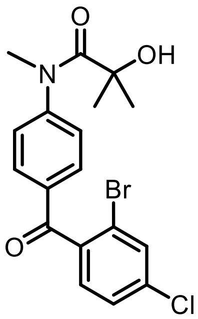

Prepared according to the ***General Procedure B*** to afford **3ad** (20.9 mg) in 51% yield as white solid, m.p. = 133.6 – 135.8 ºC.

*NMR and HRMS data for the product **3ad***:

^{1}H NMR (600 MHz, CDCl$_3$) δ (ppm): 7.84 (d, J = 9.0 Hz, 2H), 7.69 (s, 1H), 7.44 (d, J = 8.4 Hz, 1H), 7.35 (d, J = 8.4 Hz, 2H), 7.32 (d, J = 8.4 Hz, 1H), 3.98 (s, 1H), 3.36 (s, 3H), 1.26 (s, 6H).

^{13}C NMR (151 MHz, CDCl$_3$) δ (ppm): 193.7, 176.6, 149.0, 138.4, 137.0, 135.4, 133.1, 131.3, 130.0, 128.6, 127.8, 120.2, 73.2, 40.6, 28.7.

HRMS (ESI-TOF) *m/z*: [M + Na]$^+$ Calcd for $C_{18}H_{17}{}^{79}Br^{35}ClNO_3Na^+$ 431.9973, $C_{18}H_{17}{}^{81}Br^{35}ClNO_3Na^+$ 433.9953; Found 431.9980, 433.9958.

N-(4-(3-fluoro-4-methylbenzoyl)phenyl)-2-hydroxy-N,2-dimethylpropanamide 3ae

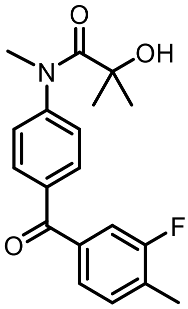

Prepared according to the ***General Procedure B*** to afford **3ae** (24.0 mg) in 73% yield as colorless oil.

*NMR and HRMS data for the product **3ae***:

^{1}H NMR (600 MHz, CDCl$_3$) δ (ppm): 7.84 (d, J = 7.8 Hz, 2H), 7.47 (t, J = 8.4 Hz, 2H), 7.37 (d, J = 8.4 Hz, 2H), 7.31 (t, J = 7.8 Hz, 1H), 4.10 (s, 1H), 3.37 (s, 3H), 2.37 (s, 3H), 1.27 (s, 6H).

^{13}C NMR (151 MHz, CDCl$_3$) δ (ppm): 193.9, 176.7, 161.0 (C-F, $^1J_{C\text{-}F}$ = 247.2 Hz), 147.7, 137.0, 136.5 (C-F, $^3J_{C\text{-}F}$ = 5.7 Hz), 131.5 (C-F, $^3J_{C\text{-}F}$ = 4.4 Hz), 131.1, 130.7 (C-F, $^2J_{C\text{-}F}$ = 17.4 Hz), 128.3, 125.7 (C-F, $^4J_{C\text{-}F}$ = 2.9 Hz), 116.4 (C-F, $^2J_{C\text{-}F}$ = 23.1 Hz), 73.1, 40.8, 28.7, 14.8.

^{19}F NMR (564 MHz, CDCl$_3$) δ (ppm): -115.77 (t, J = 8.7 Hz, 1F).

HRMS (ESI-TOF) *m/z*: [M + H]$^+$ Calcd for $C_{19}H_{21}FNO_3^+$ 330.1500; Found 330.1492.

***N*-(4-(3-fluoro-4-methoxybenzoyl)phenyl)-2-hydroxy-*N*,2-dimethylpropanamide 3af**

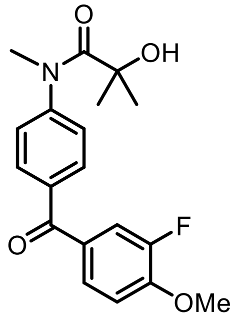

Prepared according to the ***General Procedure B*** to afford **3af** (29.7 mg) in 86% yield as white solid, m.p. = 65.7 – 68.2 ºC.

*NMR and HRMS data for the product **3af***:

^{1}H NMR (600 MHz, CDCl$_3$) δ (ppm): 7.83 (d, J = 8.4 Hz, 2H), 7.54 (d, J = 8.4 Hz, 1H), 7.38 (d, J = 8.4 Hz, 2H), 7.30 – 7.28 (m, 1H), 7.15 (dd, J = 10.3, 7.8 Hz, 1H), 4.09 (brs, 1H), 3.95 (s, 3H), 3.37 (s, 3H), 1.27 (s, 6H).

^{13}C NMR (151 MHz, CDCl$_3$) δ (ppm): 194.0, 176.7, 155.5 (C-F, $^1J_{C\text{-}F}$ = 255.9 Hz), 148.2 (C-F, $^2J_{C\text{-}F}$ = 11.6 Hz), 147.7, 137.2, 133.4 (C-F, $^3J_{C\text{-}F}$ = 4.4 Hz), 131.0, 128.3, 124.2 (C-F, $^3J_{C\text{-}F}$ = 7.2 Hz), 115.6 (C-F, $^2J_{C\text{-}F}$ = 18.7 Hz), 114.5 (C-F, $^4J_{C\text{-}F}$ = 3.0 Hz), 73.1, 56.4, 40.8, 28.7.

^{19}F NMR (564 MHz, CDCl$_3$) δ (ppm): -126.54 – -126.6 (m, 1F).

HRMS (ESI-TOF) *m/z*: [M + H]$^+$ Calcd for $C_{19}H_{21}FNO_4^+$ 346.1449; Found 346.1441.

***N*-(4-(3,5-dichlorobenzoyl)phenyl)-2-hydroxy-*N*,2-dimethylpropanamide 3ag**

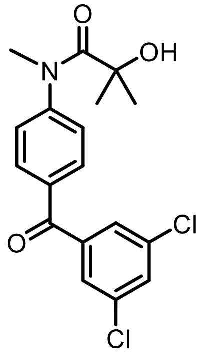

Prepared according to the ***General Procedure B*** to afford **3ag** (27.8 mg) in 76% yield as colorless oil.

*NMR and HRMS data for the product **3ag***:

^{1}H NMR (600 MHz, CDCl$_3$) δ (ppm): 7.85 (d, *J* = 9.0 Hz, 2H), 7.64 (s, 2H), 7.60 (s, 1H), 7.40 (d, *J* = 8.4 Hz, 2H), 3.96 (brs, 1H), 3.39 (s, 3H), 1.31 (s, 6H).

^{13}C NMR (151 MHz, CDCl$_3$) δ (ppm): 192.5, 176.6, 148.6, 139.7, 135.8, 135.5, 132.4, 131.2, 128.4, 128.0, 73.2, 40.7, 28.7.

HRMS (ESI-TOF) *m/z*: [M + H]$^+$ Calcd for $C_{18}H_{18}{}^{35}Cl^{37}ClNO_3^+$ 368.0629, $C_{18}H_{18}{}^{37}Cl^{37}ClNO_3^+$ 370.0599; Found 368.0635, 370.0594.

N-(4-(2-naphthoyl)phenyl)-2-hydroxy-*N*,2-dimethylpropanamide 3ah

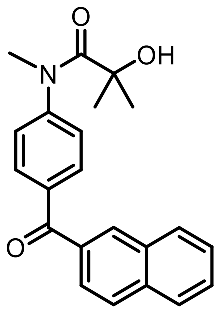

Prepared according to the ***General Procedure B*** to afford **3ah** (28.5 mg) in 82% yield as white solid, m.p. = 107.8 – 109.3 ºC.

*NMR and HRMS data for the product **3ah***:

^{1}H NMR (600 MHz, CDCl$_3$) δ (ppm): 8.26 (s, 1H), 7.97 – 7.92 (m, 6H), 7.63 (t, *J* = 7.8 Hz, 1H), 7.57 (t, *J* = 7.2 Hz, 1H), 7.40 (d, *J* = 7.8 Hz, 2H), 4.15 (s, 1H), 3.40 (s, 3H), 1.31 (s, 6H).

^{13}C NMR (151 MHz, CDCl$_3$) δ (ppm): 195.4, 176.7, 147.6, 137.5, 135.4, 134.2, 132.2, 131.8, 131.3, 129.4, 128.6, 128.5, 128.3, 127.8, 127.0, 125.5, 73.1, 40.8, 28.7.

HRMS (ESI-TOF) *m/z*: [M + Na]$^+$ Calcd for $C_{22}H_{21}NO_3Na^+$ 370.1414; Found 370.1424.

N-(4-(furan-2-carbonyl)phenyl)-2-hydroxy-*N*,2-dimethylpropanamide 3ai

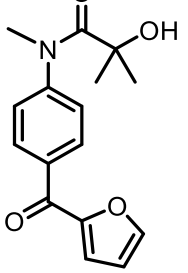

Prepared according to the ***General Procedure B*** to afford **3ai** (26.1 mg) in 91% yield as colorless oil.

*NMR and HRMS data for the product **3ai***:

^{1}H NMR (600 MHz, $CDCl_3$) δ (ppm): 8.06 (d, *J* = 8.4 Hz, 2H), 7.72 (s, 1H), 7.38 (d, *J* = 8.4 Hz, 2H), 7.29 (d, *J* = 4.2 Hz, 1H), 6.62 (d, *J* = 3.6 Hz, 1H), 4.13 (s, 1H), 3.35 (s, 3H), 1.25 (s, 6H).

^{13}C NMR (151 MHz, $CDCl_3$) δ (ppm): 180.9, 176.7, 152.1, 147.7, 147.3, 136.7, 130.6, 128.4, 120.7, 112.4, 73.1, 40.8, 28.7.

HRMS (ESI-TOF) *m/z*: $[M + Na]^+$ Calcd for $C_{16}H_{17}NO_4Na^+$ 310.1050; Found 310.1059.

2-hydroxy-*N*,2-dimethyl-*N*-(4-(thiophene-2-carbonyl)phenyl)propanamide 3aj

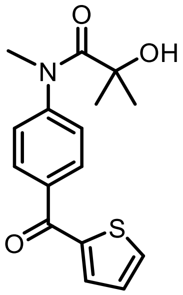

Prepared according to the ***General Procedure B*** to afford **3aj** (27.9 mg) in 92% yield as white solid, m.p. = 106.1 – 107.5 °C.

*NMR and HRMS data for the product **3aj***:

^{1}H NMR (600 MHz, $CDCl_3$) δ (ppm): 7.92 (d, *J* = 9.0 Hz, 2H), 7.76 (d, *J* = 4.8 Hz, 1H), 7.65 (d, *J* = 4.8 Hz, 1H), 7.39 (d, *J* = 8.4 Hz, 2H), 7.19 (dd, *J* = 4.8, 3.0 Hz, 1H), 4.15 (s, 1H), 3.37 (s, 3H), 1.26 (s, 6H).

^{13}C NMR (151 MHz, $CDCl_3$) δ (ppm): 186.8, 176.7, 147.5, 143.0, 137.8, 134.9, 134.8, 130.4, 128.4, 128.1, 73.1, 40.8, 28.7.

HRMS (ESI-TOF) *m/z*: $[M + Na]^+$ Calcd for $C_{16}H_{17}NO_3SNa^+$ 326.0821; Found 326.0831.

2-hydroxy-*N*,2-dimethyl-*N*-(4-(thiophene-3-carbonyl)phenyl)propanamide 3ak

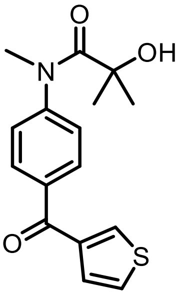

Prepared according to the ***General Procedure B*** to afford **3ak** (23.1 mg) in 76% yield as white solid, m.p. = 95.8 – 98.4 ºC.

*NMR and HRMS data for the product **3ak***:

^{1}H NMR (600 MHz, CDCl$_3$) δ (ppm): 7.95 (d, J = 4.2 Hz, 1H), 7.91 (d, J = 9.0 Hz, 2H), 7.59 (d, J = 6.0 Hz, 1H), 7.41 (dd, J = 5.4, 3.0 Hz, 1H), 7.38 (d, J = 9.0 Hz, 2H), 4.14 (s, 1H), 3.37 (s, 3H), 1.26 (s, 6H).

^{13}C NMR (151 MHz, CDCl$_3$) δ (ppm): 188.6, 176.7, 147.5, 140.8, 138.3, 134.1, 130.6, 128.44, 128.38, 126.6, 73.1, 40.8, 28.7.

HRMS (ESI-TOF) *m/z*: [M + Na]$^+$ Calcd for $C_{16}H_{17}NO_3SNa^+$ 326.0821; Found 326.0814.

2-hydroxy-*N*,2-dimethyl-*N*-(4-picolinoylphenyl)propanamide 3al

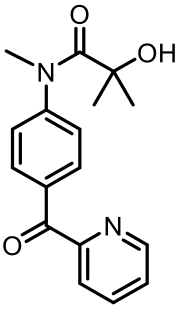

Prepared according to the ***General Procedure B*** to afford **3al** (28.3 mg) in 95% yield as colorless oil.

*NMR and HRMS data for the product **3al***:

^{1}H NMR (600 MHz, CDCl$_3$) δ (ppm): 8.71 (d, J = 4.2 Hz, 1H), 8.18 (d, J = 8.4 Hz, 2H), 8.08 (d, J = 8.4 Hz, 1H), 7.92 (t, J = 7.8 Hz, 1H), 7.51 (dd, J = 7.8, 4.8 Hz, 1H), 7.36 (d, J = 8.4 Hz, 2H), 4.16 (brs, 1H), 3.33 (s, 3H), 1.24 (s, 6H).

^{13}C NMR (151 MHz, $CDCl_3$) δ (ppm): 192.2, 176.7, 154.5, 148.5, 147.8, 137.2, 135.9, 132.4, 128.1, 126.5, 124.7, 73.1, 40.7, 28.7.

HRMS (ESI-TOF) *m/z*: [M + Na]$^+$ Calcd for $C_{17}H_{18}N_2O_3Na^+$ 321.1210; Found 321.1215.

2-hydroxy-*N*,2-dimethyl-*N*-(4-(pyrazine-2-carbonyl)phenyl)propanamide 3am

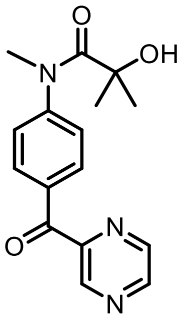

Prepared according to the ***General Procedure B*** to afford **3am** (28.1 mg) in 94% yield as colorless oil.

*NMR and HRMS data for the product **3am***:

^{1}H NMR (600 MHz, $CDCl_3$) δ (ppm): 9.27 (s, 1H), 8.78 (d, J = 2.4 Hz, 1H), 8.67 (d, J = 1.8 Hz, 1H), 8.19 (d, J = 8.4 Hz, 2H), 7.37 (d, J = 8.4 Hz, 2H), 4.05 (brs, 1H), 3.34 (s, 3H), 1.25 (s, 6H).

^{13}C NMR (151 MHz, $CDCl_3$) δ (ppm): 190.7, 176.6, 149.3, 148.5, 147.1, 146.2, 142.8, 135.0, 132.2, 128.2, 73.2, 40.7, 28.7.

HRMS (ESI-TOF) *m/z*: [M + H]$^+$ Calcd for $C_{16}H_{18}N_3O_3^+$ 300.1343; Found 300.1351.

2-hydroxy-*N*,2-dimethyl-*N*-(4-(quinoxaline-2-carbonyl)phenyl)propanamide 3an

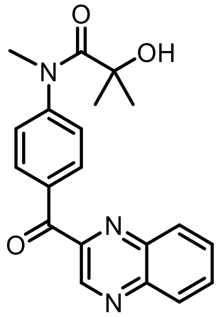

Prepared according to the ***General Procedure B*** to afford **3an** (32.5 mg) in 93% yield as white solid, m.p. = 128.8 – 131.6 °C.

*NMR and HRMS data for the product **3an***:

^{1}H NMR (600 MHz, CDCl$_3$) δ (ppm): 9.51 (s, 1H), 8.36 (d, *J* = 8.4 Hz, 2H), 8.20 (t, *J* = 7.8 Hz, 2H), 7.92 (t, *J* = 8.4 Hz, 1H), 7.87 (t, *J* = 8.4 Hz, 1H), 7.42 (d, *J* = 7.8 Hz, 2H), 4.10 (brs, 1H), 3.38 (s, 3H), 1.29 (s, 6H).

^{13}C NMR (151 MHz, CDCl$_3$) δ (ppm): 190.8, 176.6, 148.6, 148.0, 145.2, 143.2, 140.3, 135.0, 132.6, 132.3, 131.0, 130.4, 129.4, 128.2, 73.2, 40.7, 28.7.

HRMS (ESI-TOF) *m/z*: [M + Na]$^+$ Calcd for $C_{20}H_{19}N_3O_3Na^+$ 372.1319; Found 372.1312.

N-(2-bromo-4-(quinoxaline-2-carbonyl)phenyl)-2-hydroxy-N,2-dimethylpropanamide 3ao

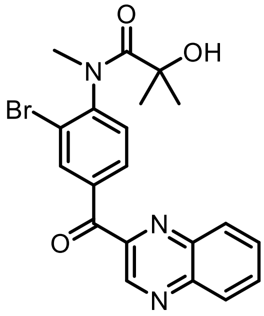

Prepared according to the ***General Procedure B*** to afford **3ao** (33.0 mg) in 77% yield as white solid, m.p. = 125.8 – 128.5 ºC.

*NMR and HRMS data for the product **3ao***:

^{1}H NMR (600 MHz, CDCl$_3$) δ (ppm): 9.51 (s, 1H), 8.64 (s, 1H), 8.33 (d, *J* = 7.2 Hz, 1H), 8.20 (d, *J* = 7.2 Hz, 2H), 7.92 (t, *J* = 7.2 Hz, 1H), 7.87 (t, *J* = 7.2 Hz, 1H), 7.45 (d, *J* = 7.8 Hz, 1H), 3.32 (s, 3H), 1.45 (s, 3H), 1.34 (s, 3H).

^{13}C NMR (151 MHz, CDCl$_3$) δ (ppm): 189.4, 176.3, 147.9, 147.6, 145.1, 143.5, 140.3, 136.54, 136.48, 132.4, 131.3, 131.0, 130.5, 129.8, 129.5, 123.6, 73.9, 39.0, 29.3, 27.6.

HRMS (ESI-TOF) *m/z*: [M + Na]$^+$ Calcd for $C_{20}H_{18}{}^{79}BrN_3O_3Na^+$ 450.0424, $C_{20}H_{18}{}^{81}BrN_3O_3Na^+$ 452.0404; Found 450.0425, 452.0408.

2-hydroxy-N,2-dimethyl-N-(4-(quinoxaline-2-carbonyl)-2-(trifluoromethyl)phenyl)propanamide 3ap

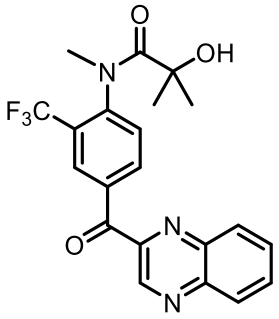

Prepared according to the ***General Procedure B*** to afford **3ap** (30.9 mg) in 74% yield as colorless oil.

*NMR and HRMS data for the product **3ap***:

^{1}H NMR (600 MHz, CDCl$_3$) δ (ppm): 9.57 (s, 1H), 8.80 (s, 1H), 8.58 (d, J = 8.4 Hz, 1H), 8.23 (d, J = 8.4 Hz, 1H), 8.20 (d, J = 8.4 Hz, 1H), 7.94 (t, J = 7.8 Hz, 1H), 7.89 (t, J = 7.2 Hz, 1H), 7.44 (d, J = 8.4 Hz, 1H), 3.38 (s, 3H), 1.55 (s, 3H), 1.48 (s, 3H).

^{13}C NMR (151 MHz, CDCl$_3$) δ (ppm): 189.5, 176.4, 147.54, 147.50, 145.2, 143.5, 140.4, 135.8, 135.3, 132.5, 131.1, 130.9 (q, J = 4.8 Hz), 130.5, 129.9, 129.6, 127.5 (q, J = 30.4 Hz), 123.2 (q, J = 273.3 Hz), 74.5, 40.6, 28.7.

^{19}F NMR (564 MHz, CDCl$_3$) δ (ppm): -60.86 (s, 1F).

HRMS (ESI-TOF) *m/z*: [M + Na]$^+$ Calcd for $C_{21}H_{18}F_3N_3O_3Na^+$ 440.1192; Found 440.1196.

2-hydroxy-*N*-(2-iodo-4-(pyrazine-2-carbonyl)phenyl)-*N*,2-dimethylpropanamide 3aq

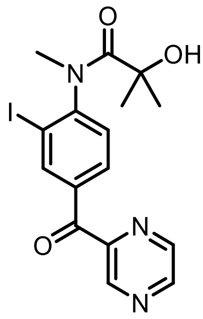

Prepared according to the ***General Procedure B*** to afford **3aq** (30.6 mg) in 72% yield as colorless oil.

*NMR and HRMS data for the product **3aq***:

^{1}H NMR (600 MHz, CDCl$_3$) δ (ppm): 9.28 (s, 1H), 8.80 (d, *J* = 3.0 Hz, 1H), 8.69 – 8.67 (m, 2H), 8.20 (d, *J* = 8.4 Hz, 1H), 7.38 (d, *J* = 7.8 Hz, 1H), 3.28 (brs, 4H), 1.47 (s, 3H), 1.35 (s, 3H).

^{13}C NMR (151 MHz, CDCl$_3$) δ (ppm): 189.2, 176.0, 151.3, 149.0, 147.3, 146.2, 142.8, 142.5, 136.2, 132.0, 128.6, 74.0, 39.2, 29.3, 27.9.

HRMS (ESI-TOF) *m/z*: [M + Na]$^+$ Calcd for $C_{16}H_{16}IN_3O_3Na^+$ 448.0129; Found 448.0121.

2-hydroxy-*N*,2-dimethyl-*N*-(2-methyl-4-(3-phenylpropanoyl)phenyl)propanamide 3ar

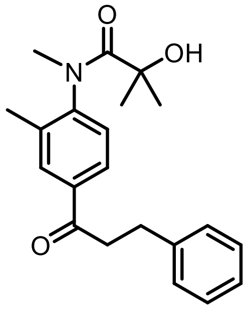

Prepared according to the ***General Procedure B*** to afford **3ar** (29.9 mg) in 88% yield as colorless oil.

*NMR and HRMS data for the product **3ar***:

^{1}H NMR (600 MHz, CDCl$_3$) δ (ppm): 7.87 (s, 1H), 7.81 (d, *J* = 8.4 Hz, 1H), 7.30 (t, *J* = 7.8 Hz, 2H), 7.26 – 7.24 (m, 3H), 7.21 (t, *J* = 7.2 Hz, 1H), 3.98 (brs, 1H), 3.43 – 3.21 (m, 5H), 3.07 (t, *J* = 7.8 Hz, 2H), 2.32 (s, 3H), 1.29 (s, 3H), 1.02 (s, 3H).

^{13}C NMR (151 MHz, CDCl$_3$) δ (ppm): 198.2, 176.6, 146.6, 141.0, 136.9, 136.8, 131.1, 129.2, 128.5, 128.4, 126.6, 126.2, 73.2, 40.5, 39.2, 30.0, 29.7, 26.5, 17.8.

HRMS (ESI-TOF) *m/z*: [M + Na]$^+$ Calcd for $C_{21}H_{25}NO_3Na^+$ 362.1727; Found 426.0684, 362.1723.

2-hydroxy-*N*-(2-methoxy-4-(3-phenylpropanoyl)phenyl)-*N*,2-dimethylpropanamide 3as

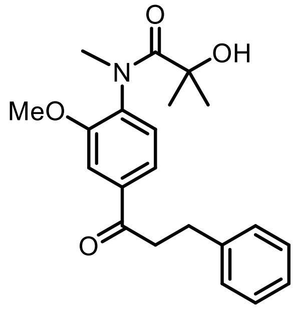

Prepared according to the ***General Procedure B*** to afford **3as** (19.9 mg) in 56% yield as colorless oil.

*NMR and HRMS data for the product **3as***:

^{1}H NMR (600 MHz, $CDCl_3$) δ (ppm): 7.58 – 7.52 (m, 2H), 7.31 (t, *J* = 7.8 Hz, 2H), 7.29 – 7.25 (m, 3H), 7.22 (t, *J* = 7.8 Hz, 1H), 4.30 (s, 1H), 3.89 (s, 3H), 3.31 (t, *J* = 7.8 Hz, 2H), 3.21 (s, 3H), 3.08 (t, *J* = 7.8 Hz, 2H), 1.26 (brs, 3H), 1.02 (brs, 3H).

^{13}C NMR (151 MHz, $CDCl_3$) δ (ppm): 198.1, 177.1, 155.8, 140.9, 138.2, 136.7, 130.0, 128.6, 128.4, 126.2, 120.8, 110.7, 72.8, 55.7, 40.5, 39.1, 30.1, 29.2, 26.3.

HRMS (ESI-TOF) *m/z*: $[M + Na]^+$ Calcd for $C_{21}H_{25}NO_4Na^+$ 378.1676; Found 378.1685.

2-hydroxy-*N*,2-dimethyl-*N*-(5-(pyrazine-2-carbonyl)-[1,1'-biphenyl]-2-yl)propanamide 3at

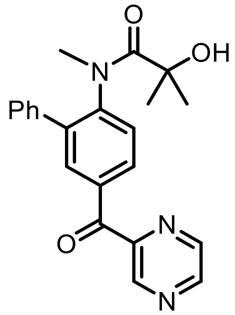

Prepared according to the ***General Procedure B*** to afford **3at** (34.2 mg) in 91% yield as white solid, m.p. = 112.2 – 115.1 °C.

*NMR and HRMS data for the product **3at***:

^{1}H NMR (600 MHz, $CDCl_3$) δ (ppm): 9.26 (s, 1H), 8.76 (s, 1H), 8.66 (s, 1H), 8.18 (s, 1H), 8.15 (d, *J* = 7.8 Hz, 1H), 7.41 – 7.34 (m, 6H), 3.07 (s, 3H), 1.37 (s, 6H).

^{13}C NMR (151 MHz, CDCl$_3$) δ (ppm): 190.9, 176.7, 149.7, 147.2, 146.9, 146.1, 142.8, 139.5, 138.0, 135.1, 134.0, 131.1, 128.6, 128.5, 128.2, 128.1, 73.4, 39.6, 28.1.

HRMS (ESI-TOF) *m/z*: [M + H]$^+$ Calcd for $C_{22}H_{22}N_3O_3^+$ 376.1656; Found 376.1653.

__*N*-(3-fluoro-4-(pyrazine-2-carbonyl)phenyl)-2-hydroxy-*N*,2-dimethylpropanamide 3au__

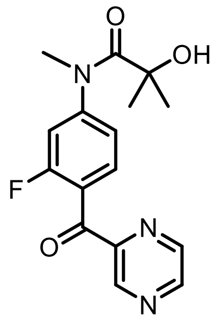

Prepared according to the ***General Procedure B*** to afford **3au** (27.9 mg) in 88% yield as colorless oil.

*NMR and HRMS data for the product **3au***:

^{1}H NMR (600 MHz, CDCl$_3$) δ (ppm): 9.25 (d, J = 1.2 Hz, 1H), 8.79 (d, J = 1.8 Hz, 1H), 8.64 (t, J = 1.8 Hz, 1H), 7.78 (t, J = 7.8 Hz, 1H), 7.20 (dd, J = 8.4, 2.4 Hz, 1H), 7.07 (dd, J = 10.2, 2.4 Hz, 1H), 3.84 (brs, 1H), 3.37 (s, 3H), 1.34 (s, 6H).

^{13}C NMR (151 MHz, CDCl$_3$) δ (ppm): 191.0, 176.5, 161.0 (d, J = 257.3 Hz), 149.5 (d, J = 10.1 Hz), 148.8, 147.7, 144.7, 143.5, 132.0 (d, J = 2.9 Hz), 124.9 (d, J = 13.0 Hz), 124.0 (d, J = 2.9 Hz), 116.2 (d, J = 23.1 Hz), 73.4, 40.5, 28.7.

^{19}F NMR (564 MHz, CDCl$_3$) δ (ppm): -104.94 (t, J = 8.7 Hz, 1F).

HRMS (ESI-TOF) *m/z*: [M + Na]$^+$ Calcd for $C_{16}H_{16}FN_3O_3Na^+$ 340.1068; Found 340.1073.

__*N*-(3-bromo-4-(quinoxaline-2-carbonyl)phenyl)-2-hydroxy-*N*,2-dimethylpropanamide 3av__

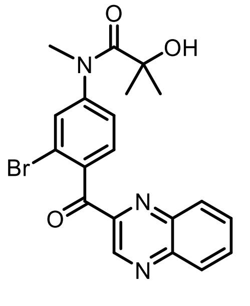

Prepared according to the ***General Procedure B*** to afford **3av** (22.7 mg) in 53% yield as white solid, m.p. = 128.5 – 129.2 ºC.

*NMR and HRMS data for the product **3av***:

^{1}H NMR (600 MHz, CDCl$_3$) δ (ppm): 9.59 (s, 1H), 8.20 (d, *J* = 8.4 Hz, 1H), 8.05 (d, *J* = 8.4 Hz, 1H), 7.91 (t, *J* = 7.8 Hz, 1H), 7.83 (t, *J* = 7.8 Hz, 1H), 7.62 (d, *J* = 7.8 Hz, 1H), 7.58 (s, 1H), 7.38 (d, *J* = 8.4 Hz, 1H), 3.41 (s, 3H), 1.36 (s, 6H).

^{13}C NMR (151 MHz, CDCl$_3$) δ (ppm): 194.2, 176.6, 146.9, 146.5, 144.1, 143.6, 140.9, 138.8, 132.8, 132.7, 131.2, 131.0, 130.6, 129.4, 126.9, 120.9, 73.3, 40.7, 28.7.

HRMS (ESI-TOF) *m/z*: [M + Na]$^+$ Calcd for $C_{20}H_{18}{}^{79}BrN_3O_3Na^+$ 450.0424, $C_{20}H_{18}{}^{81}BrN_3O_3Na^+$ 452.0404; Found 450.0418, 452.0400.

***N*-(3-cyano-4-(pyrazine-2-carbonyl)phenyl)-2-hydroxy-*N*,2-dimethylpropanamide 3aw**

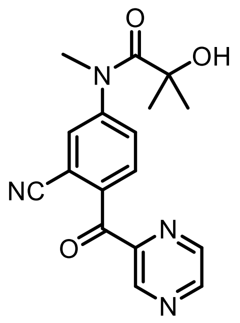

Prepared according to the ***General Procedure B*** ($KHCO_3$ as the base) to afford **3aw** (19.1 mg) in 59% yield as colorless oil.

*NMR and HRMS data for the product **3aw***:

^{1}H NMR (600 MHz, CDCl$_3$) δ (ppm): 9.41 (d, *J* = 1.2 Hz, 1H), 8.85 (d, *J* = 2.4 Hz, 1H), 8.67 (t, *J* = 1.8 Hz, 1H), 8.01 (d, *J* = 8.4 Hz, 1H), 7.73 (s, 1H), 7.61 (d, *J* = 8.4 Hz, 1H), 3.44 (s, 3H), 3.33 (brs, 1H), 1.44 (s, 6H).

^{13}C NMR (151 MHz, CDCl$_3$) δ (ppm): 190.3, 176.3, 148.2, 148.1, 147.6, 146.1, 143.2, 137.7, 133.0, 132.7, 131.2, 116.4, 113.7, 73.8, 40.3, 28.8.

HRMS (ESI-TOF) *m/z*: [M + H]$^+$ Calcd for $C_{17}H_{17}N_4O_3^+$ 325.1295; Found 325.1298.

2-hydroxy-*N*,2-dimethyl-*N*-(3-methyl-4-(3-phenylpropanoyl)phenyl)propanamide 3ax

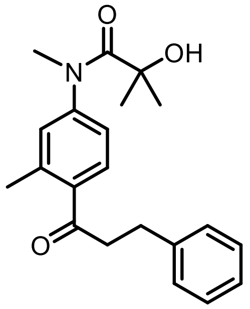

Prepared according to the ***General Procedure B*** to afford **3ax** (21.0 mg) in 62% yield as colorless oil.

*NMR and HRMS data for the product **3ax***:

^{1}H NMR (600 MHz, CDCl$_3$) δ (ppm): 7.62 (d, *J* = 9.0 Hz, 1H), 7.29 (t, *J* = 7.8 Hz, 2H), 7.24 – 7.19 (m, 3H), 7.13 – 7.11 (m, 2H), 4.20 (s, 1H), 3.30 (s, 3H), 3.23 (t, *J* = 7.8 Hz, 2H), 3.05 (t, *J* = 7.8 Hz, 2H), 2.47 (s, 3H), 1.22 (s, 6H).

^{13}C NMR (151 MHz, CDCl$_3$) δ (ppm): 202.4, 176.7, 146.1, 140.8, 140.0, 137.7, 131.7, 129.4, 128.5, 128.4, 126.2, 125.5, 73.0, 43.3, 40.8, 30.2, 28.7, 21.2.

HRMS (ESI-TOF) *m/z*: $[M + Na]^+$ Calcd for $C_{21}H_{25}NO_3Na^+$ 362.1727; Found 362.1733.

***N*-(2,6-difluoro-4-(pyrazine-2-carbonyl)phenyl)-2-hydroxy-*N*,2-dimethylpropanamide 3ay**

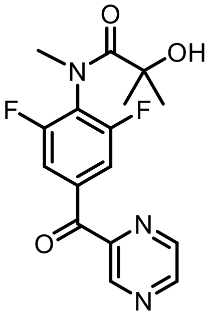

Prepared according to the ***General Procedure B*** to afford **3ay** (18.4 mg) in 55% yield as colorless oil.

*NMR and HRMS data for the product **3ay***:

^{1}H NMR (600 MHz, CDCl$_3$) δ (ppm): 9.31 (s, 1H), 8.83 (s, 1H), 8.70 (s, 1H), 7.89 (d, *J* = 7.2 Hz, 2H), 3.54 (brs, 1H), 3.25 (s, 3H), 1.32 (s, 6H).

^{13}C NMR (151 MHz, CDCl$_3$) δ (ppm): 188.1, 176.6, 158.4 (d, J = 254.4 Hz), 148.4, 147.6, 146.3, 142.8, 136.2 (t, J = 8.8 Hz), 126.3, 114.8, 74.2, 38.8, 27.8.

^{19}F NMR (564 MHz, CDCl$_3$) δ (ppm): -115.53 (d, J = 9.0 Hz, 2F).

HRMS (ESI-TOF) *m/z*: [M + Na]$^+$ Calcd for $C_{16}H_{15}F_2N_3O_3Na^+$ 358.0974; Found 358.0978.

***N*-(2,6-dimethoxy-4-(pyrazine-2-carbonyl)phenyl)-2-hydroxy-*N*,2-dimethylpropanamide 3az**

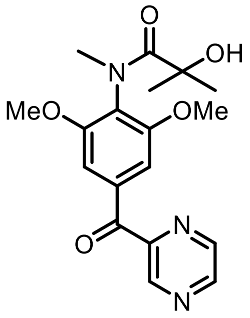

Prepared according to the ***General Procedure B*** to afford **3az** (30.9 mg) in 86% yield as white solid, m.p. = 163.0 – 165.2 ºC.

*NMR and HRMS data for the product **3az***:

^{1}H NMR (600 MHz, CDCl$_3$) δ (ppm): 9.27 (d, J = 1.2 Hz, 1H), 8.81 (d, J = 1.8 Hz, 1H), 8.69 (t, J = 1.8 Hz, 1H), 7.38 (s, 2H), 4.41 (brs, 1H), 3.86 (s, 6H), 3.13 (s, 3H), 1.11 (s, 6H).

^{13}C NMR (151 MHz, CDCl$_3$) δ (ppm): 190.7, 177.6, 156.3, 149.5, 147.1, 146.3, 142.7, 136.2, 125.6, 106.8, 72.9, 55.8, 37.6, 27.1.

HRMS (ESI-TOF) *m/z*: [M + Na]$^+$ Calcd for $C_{18}H_{21}N_3O_5Na^+$ 382.1373; Found 382.1372.

***N*-(3-fluoro-5-methyl-4-(pyrazine-2-carbonyl)phenyl)-2-hydroxy-*N*,2-dimethylpropanamide 3ba**

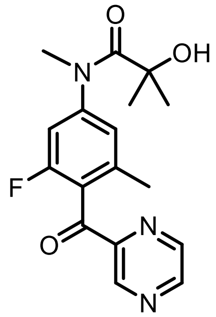

Prepared according to the ***General Procedure B*** to afford **3ba** (17.8 mg) in 54% yield as white solid, m.p. = 84.5 – 87.1 ºC.

*NMR and HRMS data for the product **3ba***:

^{1}H NMR (600 MHz, CDCl$_3$) δ (ppm): 9.33 (s, 1H), 8.79 (s, 1H), 8.62 (s, 1H), 7.00 (s, 1H), 6.88 (d, J = 9.6 Hz, 1H), 3.35 (s, 3H), 2.32 (s, 3H), 1.32 (s, 6H).

^{13}C NMR (151 MHz, CDCl$_3$) δ (ppm): 193.5, 176.6, 160.2 (d, J = 250.2 Hz), 148.2, 148.1, 146.6 (d, J = 10.1 Hz), 144.4, 144.0, 140.3 (d, J = 4.4 Hz), 126.2, 125.8 (d, J = 15.9 Hz), 113.1 (d, J = 23.1 Hz), 73.2, 40.6, 28.7, 19.5.

^{19}F NMR (564 MHz, CDCl$_3$) δ (ppm): -109.20 (d, J = 10.7 Hz, 1F).

HRMS (ESI-TOF) *m/z*: [M + H]$^+$ Calcd for $C_{17}H_{19}FN_3O_3^+$ 332.1405; Found 332.1400.

<u>*N*-(5-chloro-2-methyl-4-(pyrazine-2-carbonyl)phenyl)-2-hydroxy-*N*,2-dimethylpropanamide 3bb</u>

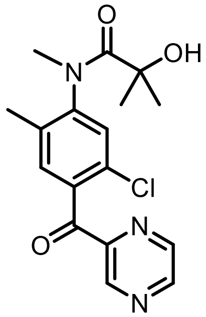

Prepared according to the ***General Procedure B*** to afford **3bb** (21.9 mg) in 63% yield as colorless oil.

*NMR and HRMS data for the product **3bb***:

^{1}H NMR (600 MHz, CDCl$_3$) δ (ppm): 9.30 (s, 1H), 8.77 (d, J = 2.4 Hz, 1H), 8.61 (t, J = 1.8 Hz, 1H), 7.44 (s, 1H), 7.25 (s, 1H), 3.64 (brs, 1H), 3.29 (s, 3H), 2.28 (s, 3H), 1.42 (s, 3H), 1.26 (s, 3H).

^{13}C NMR (151 MHz, CDCl$_3$) δ (ppm): 193.5, 176.5, 148.5, 147.6, 146.2, 145.1, 143.6, 137.1, 135.2, 132.8, 130.0, 129.8, 73.4, 39.2, 29.3, 27.4, 17.2.

HRMS (ESI-TOF) *m/z*: [M + Na]$^+$ Calcd for $C_{17}H_{18}{}^{35}ClN_3O_3Na^+$ 370.0929, $C_{17}H_{18}{}^{37}ClN_3O_3Na^+$ 372.0900; Found 370.0932, 372.0908.

<u>*N*-(2-fluoro-5-methyl-4-(pyrazine-2-carbonyl)phenyl)-2-hydroxy-*N*,2-dimethylpropanamide 3bc</u>

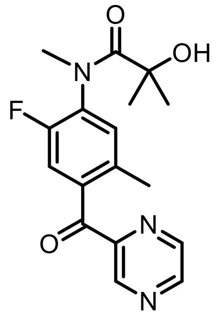

Prepared according to the ***General Procedure B*** to afford **3bc** (24.8 mg) in 75% yield as colorless oil.

*NMR and HRMS data for the product **3bc***:

^{1}H NMR (600 MHz, CDCl$_3$) δ (ppm): 9.33 (d, *J* = 1.2 Hz, 1H), 8.80 (d, *J* = 1.8 Hz, 1H), 8.65 (t, *J* = 1.8 Hz, 1H), 7.31 (d, *J* = 9.6 Hz, 1H), 7.21 (d, *J* = 7.8 Hz, 1H), 3.95 (brs, 1H), 3.31 (s, 3H), 2.37 (s, 3H), 1.31 (s, 6H).

^{13}C NMR (151 MHz, CDCl$_3$) δ (ppm): 193.6, 176.9, 155.4 (d, *J* = 250.2 Hz), 148.5, 147.6, 145.7, 143.4, 137.2, 135.1 (d, *J* = 4.4 Hz), 134.1 (d, *J* = 13.0 Hz), 132.8, 118.6 (d, *J* = 21.7 Hz), 73.2, 39.7, 28.1, 19.8.

^{19}F NMR (564 MHz, CDCl$_3$) δ (ppm): -123.24 (s, 1F).

HRMS (ESI-TOF) *m/z*: [M + H]$^+$ Calcd for $C_{17}H_{19}FN_3O_3^+$ 332.1405; Found 332.1405.

<u>*N*-(2,3-dichloro-4-(quinoxaline-2-carbonyl)phenyl)-2-hydroxy-*N*,2-dimethylpropanamide 3bd</u>

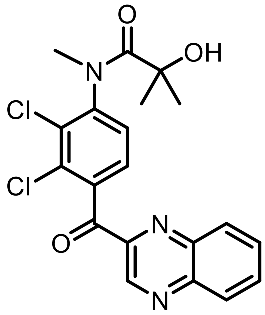

Prepared according to the ***General Procedure B*** (KHCO$_3$ as the base) to afford **3bd** (22.2 mg) in 53% yield as white solid, m.p. = 147.1 – 149.9 ºC.

*NMR and HRMS data for the product **3bd***:

^{1}H NMR (600 MHz, CDCl$_3$) δ (ppm): 9.60 (s, 1H), 8.21 (d, J = 8.4 Hz, 1H), 8.04 (d, J = 7.8 Hz, 1H), 7.91 (t, J = 7.8 Hz, 1H), 7.82 (t, J = 7.8 Hz, 1H), 7.51 (d, J = 7.2 Hz, 1H), 7.40 (d, J = 8.4 Hz, 1H), 3.44 – 3.25 (m, 4H), 1.47 (s, 3H), 1.37 (s, 3H).

^{13}C NMR (151 MHz, CDCl$_3$) δ (ppm): 193.0, 176.4, 146.5, 145.3, 143.9, 141.0, 139.1, 133.4, 132.8, 132.2, 131.0, 130.7, 129.5, 128.0, 127.9, 73.9, 38.9, 29.2, 27.5.

HRMS (ESI-TOF) *m/z*: [M + H]$^+$ Calcd for $C_{20}H_{18}{}^{35}Cl^{35}ClN_3O_3^+$ 418.0720, $C_{20}H_{18}{}^{37}Cl^{35}ClN_3O_3^+$ 420.0691; Found 418.0710, 420.0692.

***N*-(2,3-dimethoxy-4-(3-phenylpropanoyl)phenyl)-2-hydroxy-*N*,2-dimethylpropanamide 3be**

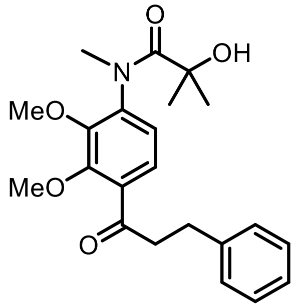

Prepared according to the ***General Procedure B*** to afford **3be** (23.1 mg) in 60% yield as colorless oil.

*NMR and HRMS data for the product **3be***:

^{1}H NMR (600 MHz, CDCl$_3$) δ (ppm): 7.32 – 7.27 (m, 3H), 7.24 (d, J = 7.2 Hz, 2H), 7.20 (t, J = 7.2 Hz, 1H), 7.00 (d, J = 8.4 Hz, 1H), 4.04 – 3.92 (m, 4H), 3.81 (s, 3H), 3.31 – 3.27 (m, 5H), 3.04 (t, J = 7.8 Hz, 2H), 1.28 (brs, 6H).

^{13}C NMR (151 MHz, CDCl$_3$) δ (ppm): 200.9, 177.1, 152.7, 149.9, 141.2, 141.1, 134.6, 128.5, 128.4, 126.1, 124.4, 123.8, 73.1, 61.1, 60.7, 56.0, 44.8, 40.1, 30.1.

HRMS (ESI-TOF) *m/z*: [M + H]$^+$ Calcd for $C_{22}H_{28}NO_5^+$ 386.1962; Found 386.1966.

2-hydroxy-*N*,2-dimethyl-*N*-(4-(3-phenylpropanoyl)naphthalen-1-yl)propanamide 3bf

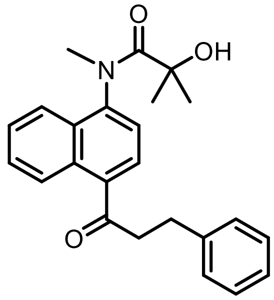

Prepared according to the ***General Procedure B*** to afford **3bf** (28.9 mg) in 77% yield as colorless oil.

*NMR and HRMS data for the product **3bf***:

^{1}H NMR (600 MHz, CDCl$_3$) δ (ppm): 8.50 (d, J = 9.6 Hz, 1H), 7.83 (d, J = 3.6 Hz, 1H), 7.76 (d, J = 7.8 Hz, 1H), 7.63 (d, J = 9.6 Hz, 2H), 7.39 (d, J = 7.8 Hz, 1H), 7.30 (t, J = 7.8 Hz, 2H), 7.27 – 7.25 (m, 2H), 7.21 (t, J = 7.2 Hz, 1H), 4.02 (brs, 1H), 3.43 – 3.38 (m, 5H), 3.15 (t, J = 7.8 Hz, 2H), 1.30 (s, 3H), 0.82 (s, 3H).

^{13}C NMR (151 MHz, CDCl$_3$) δ (ppm): 203.0, 177.5, 143.5, 140.7, 137.3, 131.3, 131.0, 128.6, 128.4, 128.0, 126.6, 126.3, 124.8, 123.1, 73.3, 44.0, 40.4, 30.4, 29.7, 27.4.

HRMS (ESI-TOF) *m/z*: [M + H]$^+$ Calcd for $C_{24}H_{26}NO_3^+$ 376.1907; Found 376.1899.

2-hydroxy-*N*,2-dimethyl-*N*-(4-(quinoxaline-2-carbonyl)-5,6,7,8-tetrahydronaphthalen-1-yl)propanamide 3bg

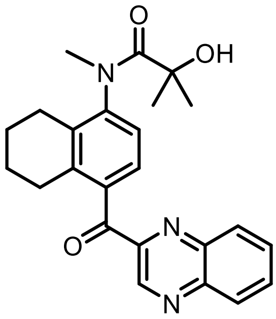

Prepared according to the ***General Procedure B*** to afford **3bg** (37.1 mg) in 92% yield as colorless oil.

*NMR and HRMS data for the product **3bg***:

^{1}H NMR (600 MHz, CDCl$_3$) δ (ppm): 9.56 (s, 1H), 8.20 (d, *J* = 8.4 Hz, 1H), 8.08 (d, *J* = 6.6 Hz, 1H), 7.90 (t, *J* = 7.2 Hz, 1H), 7.83 (t, *J* = 7.2 Hz, 1H), 7.40 (d, *J* = 7.8 Hz, 1H), 7.12 (d, J = 7.8 Hz, 1H), 3.96 (brs, 1H), 3.29 (s, 3H), 2.87 – 2.77 (m, 3H), 2.64 – 2.58 (m, 1H), 1.95 – 1.69 (m, 4H), 1.37 (s, 3H), 1.13 (s, 3H).

^{13}C NMR (151 MHz, CDCl$_3$) δ (ppm): 196.3, 176.8, 148.1, 144.6, 143.6, 140.9, 139.8, 137.3, 136.9, 132.4, 130.8, 130.6, 129.5, 128.2, 125.2, 73.4, 39.1, 29.9, 27.9, 26.8, 25.6, 22.3, 21.8.

HRMS (ESI-TOF) *m/z*: [M + Na]$^+$ Calcd for $C_{24}H_{25}N_3O_3Na^+$ 426.1788; Found 426.1786.

2-hydroxy-*N*,2-dimethyl-*N*-(8-(pyrazine-2-carbonyl)-2,3-dihydrobenzo[b][1,4]dioxin-5-yl)propanamide 3bh

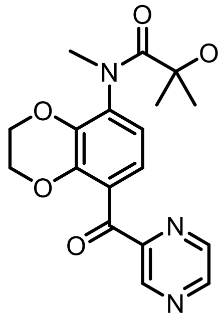

Prepared according to the ***General Procedure B*** ($KHCO_3$ as the base) to afford **3bh** (30.7 mg) in 86% yield as colorless oil.

*NMR and HRMS data for the product **3bh***:

^{1}H NMR (600 MHz, CDCl$_3$) δ (ppm): 9.19 (s, 1H), 8.75 (s, 1H), 8.63 (s, 1H), 7.18 (d, *J* = 8.4 Hz, 1H), 6.93 (d, *J* = 7.8 Hz, 1H), 4.29 (brs, 2H), 4.11 (s, 2H), 3.25 (s, 3H), 1.33 (s, 3H), 1.14 (s, 3H).

^{13}C NMR (151 MHz, CDCl$_3$) δ (ppm): 193.0, 177.2, 149.5, 147.2, 144.7, 144.0, 143.4, 140.4, 135.5, 127.0, 121.9, 121.8, 72.9, 64.1, 63.9, 39.1, 29.3, 26.5.

HRMS (ESI-TOF) *m/z*: [M + Na]$^+$ Calcd for $C_{18}H_{19}N_3O_5Na^+$ 380.1217; Found 380.1222.

***N*-benzyl-2-hydroxy-2-methyl-*N*-(4-(3-phenylpropanoyl)phenyl)propanamide 3bi**

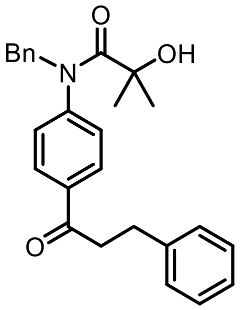

Prepared according to the ***General Procedure B*** to afford **3bi** (27.3 mg) in 68% yield as colorless oil.

*NMR and HRMS data for the product **3bi***:

^{1}H NMR (600 MHz, $CDCl_3$) δ (ppm): 7.89 (d, *J* = 8.4 Hz, 2H), 7.31 – 7.26 (m, 5H), 7.24 (d, *J* = 7.2 Hz, 2H), 7.21 (t, *J* = 7.2 Hz, 1H), 7.15 – 7.12 (m, 2H), 7.07 (d, *J* = 9.0 Hz, 2H), 4.91 (s, 2H), 4.16 (s, 1H), 3.28 (t, *J* = 7.8 Hz, 2H), 3.06 (t, *J* = 7.8 Hz, 2H), 1.18 (s, 6H).

^{13}C NMR (151 MHz, $CDCl_3$) δ (ppm): 198.0, 176.5, 146.0, 140.9, 136.5, 136.4, 129.9, 128.84, 128.81, 128.54, 128.52, 128.4, 127.8, 126.2, 73.3, 56.1, 40.5, 29.9, 28.8.

HRMS (ESI-TOF) *m/z*: $[M + Na]^+$ Calcd for $C_{26}H_{27}NO_3Na^+$ 424.1883; Found 424.1890.

<u>2-hydroxy-*N*-isopropyl-2-methyl-*N*-(4-(pyrazine-2-carbonyl)phenyl)propanamide 3bj</u>

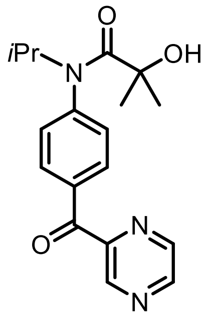

Prepared according to the ***General Procedure B*** to afford **3bj** (19.0 mg) in 58% yield as white solid, m.p. = 104.1 – 106.9 ºC.

*NMR and HRMS data for the product **3bj***:

^{1}H NMR (600 MHz, $CDCl_3$) δ (ppm): 9.29 (d, *J* = 1.2 Hz, 1H), 8.81 (d, *J* = 2.4 Hz, 1H), 8.70 (t, *J* = 1.8 Hz, 1H), 8.23 (d, *J* = 9.0 Hz, 2H), 7.31 (d, *J* = 8.4 Hz, 2H), 5.03 – 4.99 (m, 1H), 1.14 (s, 6H), 1.08 (d, *J* = 7.2 Hz, 6H).

^{13}C NMR (151 MHz, $CDCl_3$) δ (ppm): 190.7, 176.0, 149.3, 147.1, 146.2, 142.8, 142.5, 135.4, 131.5, 131.4, 73.2, 49.3, 28.8, 20.7.

HRMS (ESI-TOF) *m/z*: $[M + Na]^+$ Calcd for $C_{18}H_{21}N_3O_3Na^+$ 350.1475; Found 350.1477.

2-hydroxy-*N*-methyl-*N*-(4-(3-phenylpropanoyl)phenyl)propanamide 3bk

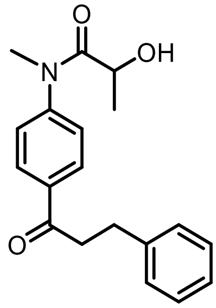

Prepared according to the ***General Procedure B*** to afford **3bk** (19.9 mg) in 64% yield as white solid, m.p. = 81.2 – 85.0 ºC.

*NMR and HRMS data for the product **3bk***:

^{1}H NMR (600 MHz, CDCl$_3$) δ (ppm): 8.01 (d, *J* = 8.4 Hz, 2H), 7.33 – 7.28 (m, 4H), 7.25 (d, *J* = 7.8 Hz, 2H), 7.20 (t, *J* = 7.2 Hz, 1H), 4.28 (brs, 1H), 3.39 – 3.33 (m, 4H), 3.30 (t, *J* = 7.8 Hz, 2H), 3.09 (t, *J* = 7.8 Hz, 2H), 1.12 (d, *J* = 3.6 Hz, 3H).

^{13}C NMR (151 MHz, CDCl$_3$) δ (ppm): 197.9, 175.4, 146.6, 141.0, 136.5, 129.7, 128.6, 128.4, 127.3, 126.3, 64.9, 40.5, 37.8, 30.1, 21.2.

HRMS (ESI-TOF) *m/z*: $[M + Na]^+$ Calcd for $C_{19}H_{21}NO_3Na^+$ 334.1414; Found 334.1416.

1-hydroxy-*N*-methyl-*N*-(4-(pyrazine-2-carbonyl)phenyl)cyclobutane-1-carboxamide 3bl

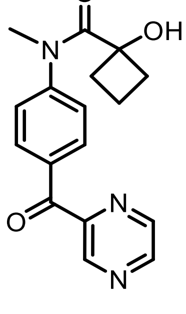

Prepared according to the ***General Procedure B*** to afford **3bl** (18.1 mg) in 58% yield as colorless oil.

*NMR and HRMS data for the product **3bl***:

^{1}H NMR (600 MHz, CDCl$_3$) δ (ppm): 9.26 (d, J = 1.2 Hz, 1H), 8.79 (d, J = 2.4 Hz, 1H), 8.68 (s, 1H), 8.15 (d, J = 8.4 Hz, 2H), 7.43 (d, J = 8.4 Hz, 2H), 3.36 (s, 3H), 2.96 (brs, 1H), 2.57 – 2.47 (m, 2H), 1.98 – 1.91 (m, 1H), 1.89 – 1.81 (m, 2H), 1.67 – 1.58 (m, 1H).

^{13}C NMR (151 MHz, CDCl$_3$) δ (ppm): 190.8, 173.6, 149.7, 148.4, 146.9, 146.1, 142.8, 133.8, 132.0, 126.5, 77.8, 38.6, 34.4, 13.7.

HRMS (ESI-TOF) *m/z*: [M + H]$^+$ Calcd for $C_{17}H_{18}N_3O_3^+$ 312.1343; Found 312.1338.

2-Hydroxy-*N*-methyl-*N*-(4-(quinoxaline-2-carbonyl)phenyl)acetamide 3bm

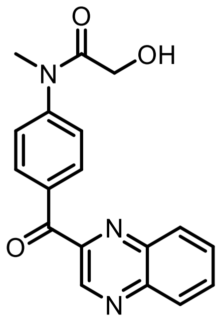

Prepared according to the ***General Procedure B*** to afford **3bm (**30.3mg) 94% yield as yellow oil.

*NMR and HRMS data for the product **3bm***:

^{1}H NMR (600 MHz, CDCl$_3$) δ (ppm): 9.54 (s, 1H), 8.39 (d, J = 9.0 Hz, 2H), 8.21 (t, J = 9.6 Hz, 2H), 7.94-7.87 (m, 2H), 7.39 (d, J = 8.4 Hz, 2H), 4.00 (s, 2H), 3.40 (s, 3H), 3.35 (s, 1H).

^{13}C NMR (151 MHz, CDCl$_3$) δ (ppm): 190.7, 171.8, 148.0, 145.9, 145.2, 143.4, 140.4, 135.4, 133.0, 132.3, 131.0, 130.4, 129.6, 126.5, 60.7, 37.1.

HRMS (ESI-TOF) *m/z*: [M + Na]$^+$ Calcd for $C_{18}H_{15}N_3O_3Na^+$ 344.1006; Found 344.1000.

Difunctionalization via 6-membered MI

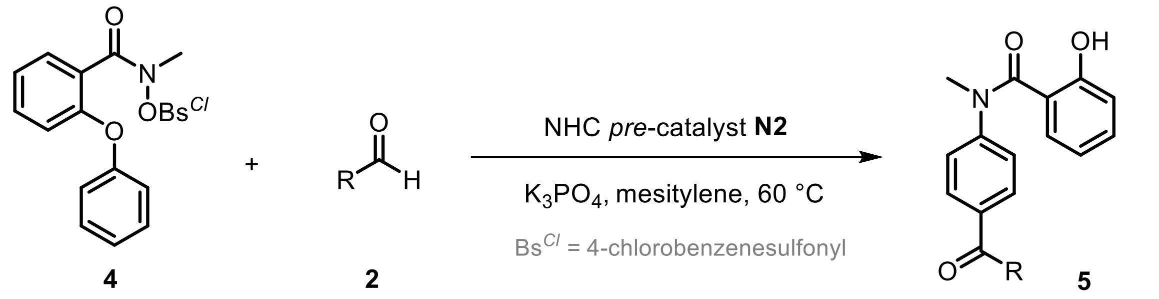

To an oven-dried Schlenck tube was added substrate **4** (0.10 mmol), NHC **N2** (10 mol %) and K_3PO_4 (0.20 mmol). The Schlenck tube was subjected to three cycles of pressurization/depressurization using dry Ar. After that, under the protection of Ar atmosphere, a solution of aldehydes **2** (0.25 mmol) in dry mesitylene (1 mL) was added, and the reaction mixture was stirred at 60 °C for 12 hours. Then the mixture was purified by column chromatography on silica gel to afford the corresponding products **5**, which were dried under vacuum and further analyzed by ^{1}H NMR, ^{13}C NMR, HRMS, etc.

2-hydroxy-*N*-methyl-*N*-(4-(quinoxaline-2-carbonyl)phenyl)benzamide 5a

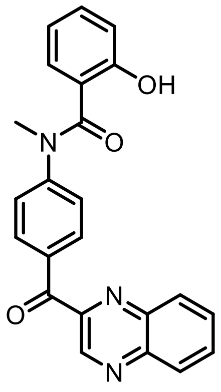

Prepared according to the above procedure to afford **5a (**24mg) 62% yield as red solid, m.p. = 168.5 – 173.8 ºC.

*NMR and HRMS data for the product **5a***:

^{1}H NMR (600 MHz, $CDCl_3$) δ (ppm): 10.61 (s, 1H), 9.48 (s, 1H), 8.26 (d, *J* = 9.0 Hz, 2H), 8.20-8.15 (m, 2H), 7.92-7.84 (m, 2H), 7.25 (d, *J* = 9.0 Hz, 2H), 7.19 (t, *J* = 6.6 Hz, 1H), 6.94 (d, *J* = 8.4 Hz, 1H), 6.77-6.75 (m, 1H), 6.46 (t, *J* = 7.8 Hz, 1H), 3.55 (s, 3H).

^{13}C NMR (151 MHz, $CDCl_3$) δ (ppm): 190.6, 171.8, 160.5, 149.8, 148.2, 145.2, 143.2, 140.2, 133.4, 133.2, 132.8, 132.2, 130.9, 130.4, 130.3, 129.4, 126.1, 118.1, 118.0, 115.7, 38.8.

HRMS (ESI-TOF) *m/z*: $[M + H]^+$ Calcd for $C_{23}H_{17}N_3O_3H^+$ 384.1343; Found 384.1336.

2-hydroxy-*N*-methyl-*N*-(4-picolinoylphenyl)benzamide 5b

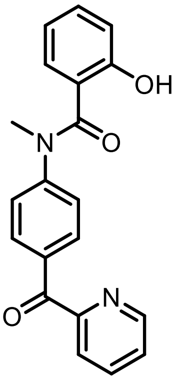

Prepared according to the above procedure to afford **5b (**16.2mg) 49% yield as colorless oil.

*NMR and HRMS data for the product **5b***:

^{1}H NMR (600 MHz, $CDCl_3$) δ (ppm): 10.68 (s, 1H), 8.71 (d, *J* = 4.8 Hz, 1H), 8.10 (d, *J* = 8.4 Hz, 2H), 8.05 (d, *J* = 7.8 Hz, 1H), 7.91 (t, *J* = 7.8 Hz, 1H), 7.51-7.49 (m, 1H), 7.22-7.18 (m, 3H), 6.94 (d, *J* = 8.4 Hz, 1H), 6.75 (d, *J* = 7.8 Hz, 1H), 6.44 (t, *J* = 7.8 Hz, 1H), 3.53 (s, 3H).

^{13}C NMR (151 MHz, $CDCl_3$) δ (ppm): 192.1, 171.8, 160.5, 154.7, 149.1, 148.4, 137.2, 134.2, 133.2, 132.6, 130.4, 126.4, 125.9, 124.7, 118.1, 118.0, 115.7, 38.9.

HRMS (ESI-TOF) *m/z*: $[M + H]^+$ Calcd for $C_{20}H_{16}N_2O_3H^+$ 333.1234; Found 333.1231.

2-hydroxy-*N*-methyl-*N*-(4-(quinoline-2-carbonyl)phenyl)benzamide 5c

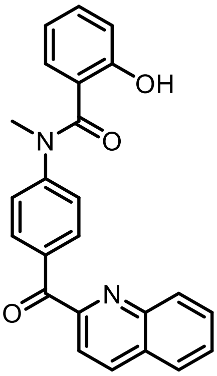

Prepared according to the above procedure to afford **5c (**13.7mg) 38% yield as colorless oil.

*NMR and HRMS data for the product **5c***:

^{1}H NMR (600 MHz, CDCl$_3$) δ (ppm): 10.69 (s, 1H), 8.36 (d, *J* = 8.4 Hz, 1H), 8.27 (d, *J* = 9.0 Hz, 2H), 8.16 (d, *J* = 8.4 Hz, 1H), 8.12 (d, *J* = 9.0 Hz, 1H), 7.92 (d, *J* = 8.4 Hz, 1H), 7.79 (d, *J* = 7.2 Hz, 1H), 7.68 (d, *J* = 6.6 Hz, 1H), 7.24 (d, *J* = 9.0 Hz, 2H), 7.21 (t, *J* = 7.2 Hz, 1H), 6.96 (d, *J* = 8.4 Hz, 1H), 6.78 (d, *J* = 8.4 Hz, 1H), 6.47 (t, *J* = 7.8 Hz, 1H), 3.57 (s, 3H).
^{13}C NMR (151 MHz, CDCl$_3$) δ (ppm): 192.1, 171.8, 160.5, 154.2, 149.2, 146.6, 137.3, 134.2, 133.2, 133.0, 130.5, 130.4, 130.2, 129.0, 128.6, 127.7, 125.9, 120.7, 118.1, 118.0, 115.8, 38.9.
HRMS (ESI-TOF) *m/z*: $[M + H]^+$ Calcd for $C_{24}H_{18}N_2O_3H^+$ 383.1390; Found 383.1387.

*Hydrolysis of **5a***

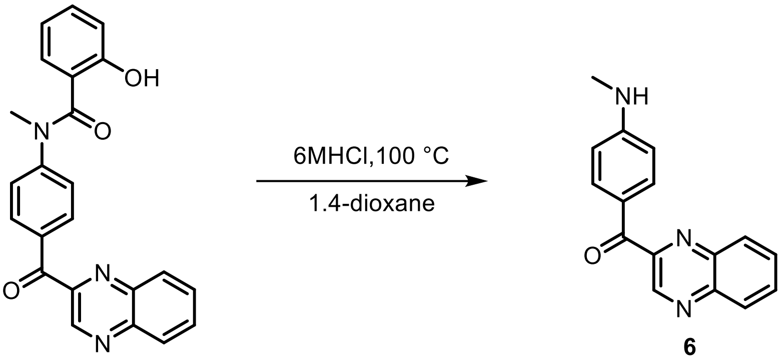

5a **6**

To an oven-dried Schlenck tube was added substrate **5a** (0.63mmol). Then, added 1 mL HCl solution (in 1,4-dioxane, 6 M) slowly, and further stirred at 100 °C for 12 h. The resulting mixture was diluted with water (5.0 mL) and extracted with ethyl acetate (3 × 10 mL), washed with 1M $NaHCO_3$ aq. (2 × 20 mL), and the aqueous layer was extracted with ethyl acetate (3 × 10 mL). The organic layer was combined and dried over sodium sulfate and filtered. The filtrate was evaporated in vacuo to give a residue, which was purified by column chromatography on silica gel to afford the compound **6**. The product was finally dried under vacuum and further analyzed by ^{1}H NMR, ^{13}C NMR, HRMS, etc.

2-hydroxy-*N*-methyl-*N*-(4-(quinoxaline-2-carbonyl)phenyl)benzamide 6

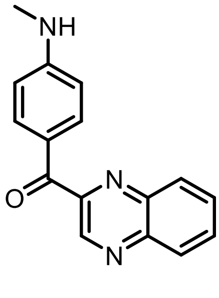

Prepared according to the above procedure(0.63mmol) to afford **6** (12.3 mg) in 74% yield as yellow solid.

*NMR and HRMS data for the product **6**:*

^{1}H NMR (600 MHz, CDCl$_3$) δ (ppm): 9.39 (s, 1H), 8.21-8.16 (m, 4H), 7.89-7.83 (m, 2H), 6.63 (d, *J* = 8.4 Hz, 2H), 4.51 (s, 1H), 2.95 (s, 3H).

^{13}C NMR (151 MHz, CDCl$_3$) δ (ppm): 189.8, 153.8, 150.4, 145.6, 142.8, 140.4, 134.0, 131.3, 130.5, 130.2, 129.3, 124.3, 111.1, 77.2, 77.0, 76.8, 30.0.

HRMS (ESI-TOF) *m/z*: [M + H]$^+$ Calcd for $C_{16}H_{14}N_3OH^+$ 264.1131; Found 264.1124.

Supplementary background of the aromatic C-H acylation (methods and selectivity):

As depicted in the figure below, classical electrophilic substitutions, such as Friedel–Crafts reactions, typically require harsh conditions (strong acids, elevated temperatures). Radical acylative additions, often initiated photochemically or electrochemically, are largely governed by the arene's intrinsic electronic properties. Both approaches rely on the intrinsic electronic nature of the aromatic ring to determine site-selectivity. Directing-group-assisted transition-metal catalysis typically leads to *ortho*-selective acylation. In contrast, our approach, involving radical coupling between a dearomatized carbon-centered radical and an NHC-bound ketyl radical, achieves exclusive *para*-acylation while simultaneously swapping the heteroatom at the *ipso* position.

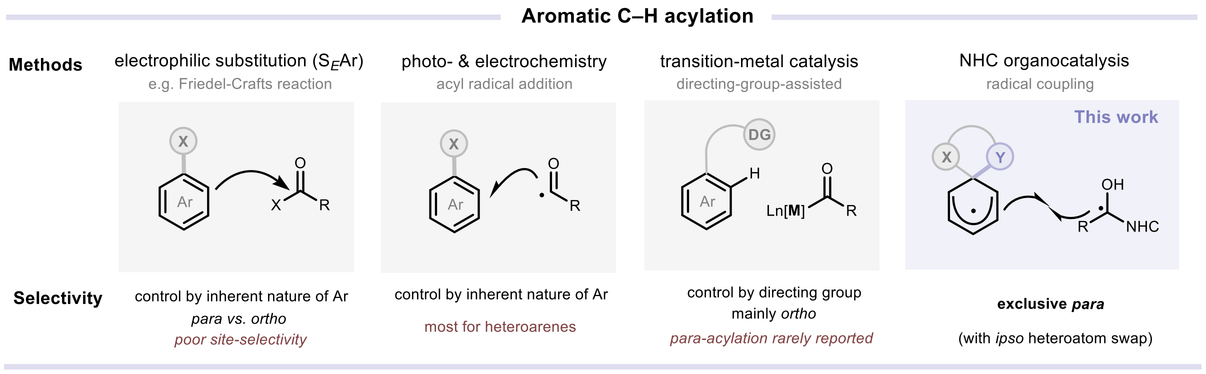

Comparison of the bond cleavage selectivity between our previous work and this study

In our earlier work, the reaction proceeded via cleavage of the newly formed C–Y bond, resulting in arene mono-functionalization. In contrast, the current system selectively cleaves the pre-existing C–X bond, enabling para-selective acylation accompanied by simultaneous functionalization at the C–X site. This inversion of bond-cleavage preference gives rise to an efficient arene difunctionalization manifold. Importantly, this work represents a significant advance in the field of Smiles rearrangement, as it enables the installation of a functional group onto the migrating aryl unit during the rearrangement process.

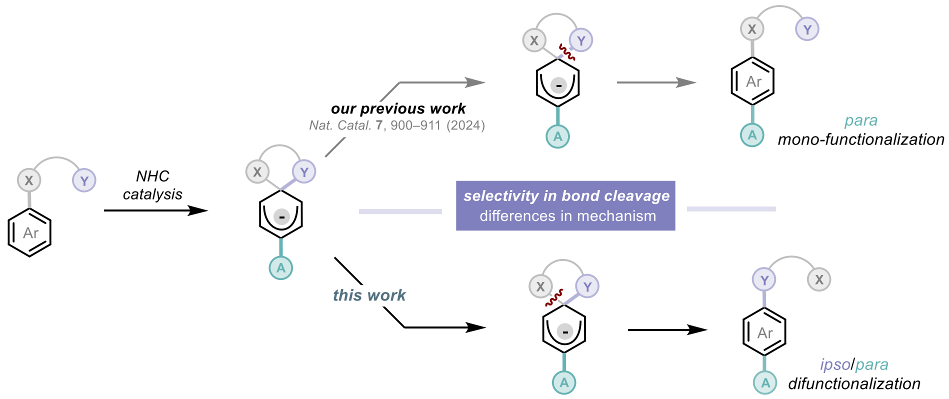

5. Investigations on Ring Expansion Reaction

General Procedure C: *the NHC-catalyzed ring expansion*

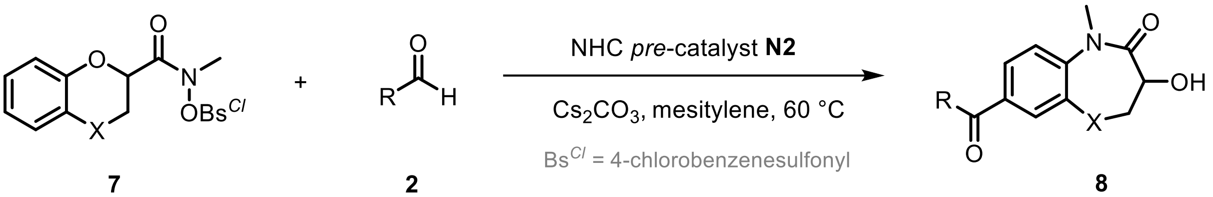

To an oven-dried Schlenck tube was added substrates **7** (0.10 mmol), NHC **N2** (10 mol %) and Cs_2CO_3 (0.20 mmol). The Schlenck tube was subjected to three cycles of pressurization/depressurization using dry Ar. After that, under the protection of Ar atmosphere, a solution of aldehydes **2** (0.25 mmol) in dry mesitylene (1 mL) was added, and the reaction mixture was stirred at 60 °C for 12 hours. Then the mixture was purified by column chromatography on silica gel to afford the corresponding ketone products **8**, which were dried under vacuum and further analyzed by ^{1}H NMR, ^{13}C NMR, HRMS, etc.

8-benzoyl-3-hydroxy-5-methyl-2,3-dihydrobenzo[*b*][1,4]oxazepin-4(5*H*)-one 8a

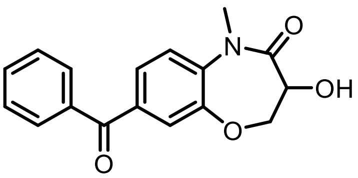

Prepared according to the ***General Procedure C*** to afford **8a** (20.0 mg) in 67% yield as colorless oil.

NMR and HRMS data for the product ***8a***:

^{1}H NMR (600 MHz, $CDCl_3$) δ (ppm): 7.79 (d, *J* = 7.2 Hz, 2H), 7.68 (d, *J* = 7.8 Hz, 1H), 7.65 – 7.59 (m, 2H), 7.51 (t, *J* = 7.8 Hz, 2H), 7.31 (d, *J* = 8.4 Hz, 1H), 4.62 (dd, *J* = 9.6, 7.2 Hz, 1H), 4.44 – 4.37 (m, 1H), 4.25 (t, *J* = 10.2 Hz, 1H), 3.60 (d, *J* = 6.0 Hz, 1H), 3.51 (s, 3H).

^{13}C NMR (151 MHz, $CDCl_3$) δ (ppm): 194.7, 172.4, 150.1, 139.3, 137.0, 136.5, 132.8, 129.9, 128.5, 127.2, 124.4, 122.6, 79.0, 66.5, 35.4.

HRMS (ESI-TOF) *m/z*: $[M + H]^+$ Calcd for $C_{17}H_{16}NO_4^+$ 298.1074; Found 298.1078.

8-(4-bromobenzoyl)-3-hydroxy-5-methyl-2,3-dihydrobenzo[*b*][1,4]oxazepin-4(5*H*)-one 8b

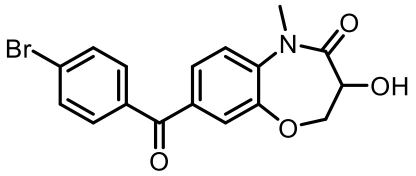

Prepared according to the ***General Procedure C*** to afford **8b** (22.9 mg) in 61% yield as colorless oil.

*NMR and HRMS data for the product **8b***:

^{1}H NMR (600 MHz, CDCl$_3$) δ (ppm): 7.68 – 7.63 (m, 5H), 7.60 (s, 1H), 7.31 (d, *J* = 8.4 Hz, 1H), 4.61 (dd, *J* = 9.6, 7.2 Hz, 1H), 4.42 – 4.38 (m, 1H), 4.25 (t, *J* = 10.2 Hz, 1H), 3.60 (d, *J* = 6.6 Hz, 1H), 3.51 (s, 3H).

^{13}C NMR (151 MHz, CDCl$_3$) δ (ppm): 193.6, 172.4, 150.2, 139.6, 135.9, 135.7, 131.8, 131.4, 127.9, 127.1, 124.3, 122.7, 79.0, 66.5, 35.4.

HRMS (ESI-TOF) *m/z*: $[M + H]^+$ Calcd for $C_{17}H_{15}{}^{79}BrNO_4^+$ 376.0179, $C_{17}H_{15}{}^{81}BrNO_4^+$ 378.0159; Found 376.0175, 378.0160.

3-hydroxy-5-methyl-8-(4-methylbenzoyl)-2,3-dihydrobenzo[*b*][1,4]oxazepin-4(5*H*)-one 8c

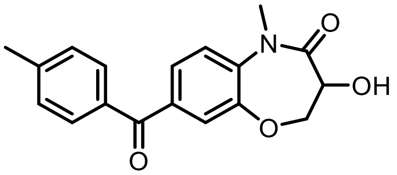

Prepared according to the ***General Procedure C*** to afford **8c** (13.1 mg) in 42% yield as colorless oil.

*NMR and HRMS data for the product **8c***:

^{1}H NMR (600 MHz, CDCl$_3$) δ (ppm): 7.71 (d, *J* = 7.2 Hz, 2H), 7.65 (d, *J* = 8.4 Hz, 1H), 7.61 (s, 1H), 7.34 – 7.29 (m, 3H), 4.61 (dd, *J* = 9.6, 7.2 Hz, 1H), 4.42 – 4.39 (m, 1H), 4.24 (t, *J* = 10.2 Hz, 1H), 3.59 (brs, 1H), 3.51 (s, 3H), 2.45 (s, 3H).

^{13}C NMR (151 MHz, CDCl$_3$) δ (ppm): 194.5, 172.4, 150.1, 143.7, 139.1, 136.9, 134.3, 130.1, 129.2, 127.1, 124.3, 122.5, 79.0, 66.5, 35.4, 21.7.

HRMS (ESI-TOF) *m/z*: $[M + H]^+$ Calcd for $C_{18}H_{18}NO_4^+$ 312.1230; Found 312.1232.

8-(3-chlorobenzoyl)-3-hydroxy-5-methyl-2,3-dihydrobenzo[*b*][1,4]oxazepin-4(5*H*)-one 8d

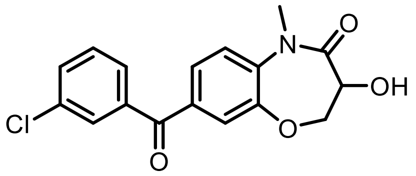

Prepared according to the ***General Procedure C*** to afford **8d** (19.8 mg) in 60% yield as colorless oil.

*NMR and HRMS data for the product **8d***:

^{1}H NMR (600 MHz, $CDCl_3$) δ (ppm): 7.76 (s, 1H), 7.65 (d, *J* = 8.4 Hz, 2H), 7.62 (s, 1H), 7.58 (d, *J* = 8.4 Hz, 1H), 7.45 (t, *J* = 7.8 Hz, 1H), 7.32 (d, *J* = 7.8 Hz, 1H), 4.61 (dd, *J* = 10.2, 7.2 Hz, 1H), 4.44 – 4.37 (m, 1H), 4.25 (t, *J* = 10.2 Hz, 1H), 3.61 (d, *J* = 6.0 Hz, 1H), 3.51 (s, 3H).

^{13}C NMR (151 MHz, $CDCl_3$) δ (ppm): 193.2, 172.4, 150.2, 139.7, 138.6, 135.7, 134.7, 132.7, 129.8, 129.7, 127.9, 127.1, 124.3, 122.7, 79.0, 66.5, 35.4.

HRMS (ESI-TOF) *m/z*: $[M + H]^+$ Calcd for $C_{17}H_{15}{}^{35}ClNO_4^+$ 332.0684, $C_{17}H_{15}{}^{37}ClNO_4^+$ 334.0655; Found 332.0687, 334.0660.

3-hydroxy-8-(3-methoxybenzoyl)-5-methyl-2,3-dihydrobenzo[*b*][1,4]oxazepin-4(5*H*)-one 8e

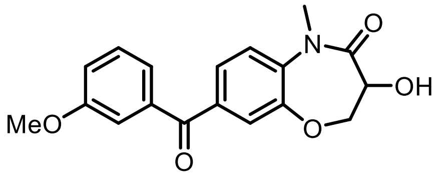

Prepared according to the ***General Procedure C*** to afford **8e** (15.0 mg) in 46% yield as white solid, m.p. = 87.1 – 89.9 ºC.

*NMR and HRMS data for the product **8e***:

^{1}H NMR (600 MHz, $CDCl_3$) δ (ppm): 7.68 (d, *J* = 7.8 Hz, 1H), 7.64 (s, 1H), 7.40 (t, *J* = 7.8 Hz, 1H), 7.34 – 7.32 (m, 2H), 7.30 (d, *J* = 7.8 Hz, 1H), 7.16 (d, *J* = 8.4 Hz, 1H), 4.62 (dd, *J* =

9.6, 7.2 Hz, 1H), 4.41 (dd, J = 10.2, 7.2 Hz, 1H), 4.25 (t, J = 10.2 Hz, 1H), 3.87 (s, 3H), 3.58 (brs, 1H), 3.52 (s, 3H).

^{13}C NMR (151 MHz, CDCl$_3$) δ (ppm): 194.5, 172.4, 159.7, 150.1, 139.3, 138.3, 136.5, 129.4, 127.2, 124.4, 122.6, 122.5, 119.1, 114.3, 79.0, 66.5, 55.5, 35.4.

HRMS (ESI-TOF) *m/z*: [M + H]$^+$ Calcd for $C_{18}H_{18}NO_5^+$ 328.1179; Found 328.1181.

8-(2-fluorobenzoyl)-3-hydroxy-5-methyl-2,3-dihydrobenzo[*b*][1,4]oxazepin-4(5*H*)-one 8f

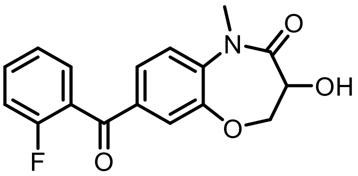

Prepared according to the ***General Procedure C*** to afford **8f** (16.0 mg) in 51% yield as white solid, m.p. = 115.5 – 117.6 °C.

*NMR and HRMS data for the product **8f**:*

^{1}H NMR (600 MHz, CDCl$_3$) δ (ppm): 7.70 (d, J = 9.0 Hz, 1H), 7.64 (s, 1H), 7.58 – 7.55 (m, 2H), 7.32 – 7.29 (m, 2H), 7.18 (t, J = 9.0 Hz, 1H), 4.61 (dd, J = 9.6, 6.6 Hz, 1H), 4.41 – 4.37 (m, 1H), 4.24 (t, J = 10.2 Hz, 1H), 3.57 (d, J = 6.6 Hz, 1H), 3.50 (s, 3H).

^{13}C NMR (151 MHz, CDCl$_3$) δ (ppm): 191.5, 172.4, 160.0 (d, J = 253.1 Hz), 150.3, 140.2, 136.2, 133.5 (d, J = 7.2 Hz), 130.7, 126.9, 126.4 (d, J = 14.5 Hz), 124.5 (d, J = 4.2 Hz), 124.1, 122.7, 116.4 (d, J = 21.6 Hz), 79.0, 66.6, 35.4.

^{19}F NMR (564 MHz, CDCl$_3$) δ (ppm): -110.50 – -110.59 (m, 1F).

HRMS (ESI-TOF) *m/z*: [M + H]$^+$ Calcd for $C_{17}H_{15}FNO_4^+$ 316.0980; Found 316.0980.

3-hydroxy-8-(2-hydroxybenzoyl)-5-methyl-2,3-dihydrobenzo[*b*][1,4]oxazepin-4(5*H*)-one 8g

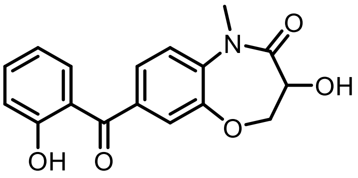

Prepared according to the ***General Procedure C*** to afford **8g** (16.9 mg) in 54% yield as white

solid, m.p. = 169.5 – 171.6 °C.

*NMR and HRMS data for the product **8g***:

^{1}H NMR (600 MHz, CDCl$_3$) δ (ppm): 11.83 (s, 1H), 7.58 (d, *J* = 7.2 Hz, 1H), 7.57 – 7.53 (m, 2H), 7.52 (s, 1H), 7.33 (d, *J* = 8.4 Hz, 1H), 7.09 (d, *J* = 8.4 Hz, 1H), 6.91 (t, *J* = 7.8 Hz, 1H), 4.63 (dd, *J* = 10.2, 7.2 Hz, 1H), 4.44 – 4.41 (m, 1H), 4.26 (t, *J* = 10.2 Hz, 1H), 3.61 (d, *J* = 6.0 Hz, 1H), 3.52 (s, 3H).

^{13}C NMR (151 MHz, CDCl$_3$) δ (ppm): 199.3, 172.4, 163.2, 150.2, 138.9, 136.7, 133.1, 126.3, 123.7, 122.7, 118.8, 118.7, 118.6, 79.0, 66.5, 35.4.

HRMS (ESI-TOF) *m/z*: [M + H]$^+$ Calcd for $C_{17}H_{16}NO_5^+$ 314.1023; Found 314.1024.

3-hydroxy-5-methyl-8-(quinoxaline-2-carbonyl)-2,3-dihydrobenzo[*b*][1,4]oxazepin-4(5*H*)-one 8h

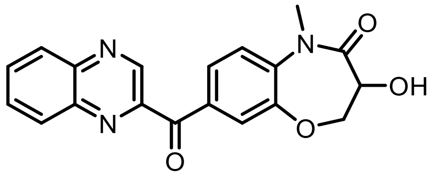

Prepared according to the ***General Procedure C*** to afford **8h** (18.8 mg) in 54% yield as white solid, m.p. = 217.1 – 219.6 °C.

*NMR and HRMS data for the product **8h***:

^{1}H NMR (600 MHz, CDCl$_3$) δ (ppm): 9.52 (s, 1H), 8.22 (t, *J* = 7.2 Hz, 2H), 8.18 (d, *J* = 7.8 Hz, 1H), 8.14 (s, 1H), 7.94 (t, *J* = 7.8 Hz, 1H), 7.89 (t, *J* = 7.8 Hz, 1H), 7.37 (d, *J* = 8.4 Hz, 1H), 4.66 (dd, *J* = 10.2, 7.2 Hz, 1H), 4.45 (dd, *J* = 10.2, 7.2 Hz, 1H), 4.27 (t, *J* = 10.2 Hz, 1H), 3.60 (brs, 1H), 3.54 (s, 3H).

^{13}C NMR (151 MHz, CDCl$_3$) δ (ppm): 190.1, 172.5, 150.1, 148.0, 145.2, 143.3, 140.4, 140.3, 134.3, 132.4, 131.0, 130.4, 129.5, 128.6, 125.7, 122.5, 79.0, 66.6, 35.4.

HRMS (ESI-TOF) *m/z*: [M + H]$^+$ Calcd for $C_{19}H_{16}N_3O_4^+$ 350.1135; Found 350.1128.

3-hydroxy-5-methyl-8-(pyrazine-2-carbonyl)-2,3-dihydrobenzo[*b*][1,4]oxazepin-4(5*H*)-one 8i

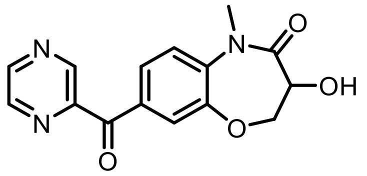

Prepared according to the ***General Procedure C*** to afford **8i** (22.1 mg) in 74% yield as white solid, m.p. = 163.3 – 165.1 ºC.

*NMR and HRMS data for the product **8i**:*

^{1}H NMR (600 MHz, CDCl$_3$) δ (ppm): 9.29 (d, *J* = 1.2 Hz, 1H), 8.81 (d, *J* = 3.0 Hz, 1H), 8.69 (dd, *J* = 2.4, 1.2 Hz, 1H), 8.02 (d, *J* = 8.4 Hz, 1H), 7.99 (s, 1H), 7.33 (d, *J* = 9.0 Hz, 1H), 4.64 (dd, *J* = 10.2, 7.2 Hz, 1H), 4.41 (dd, *J* = 10.8, 7.2 Hz, 1H), 4.25 (t, *J* = 10.2 Hz, 1H), 3.60 (brs, 1H), 3.51 (s, 3H).

^{13}C NMR (151 MHz, CDCl$_3$) δ (ppm): 189.9, 172.4, 150.0, 149.3, 147.1, 146.2, 142.8, 140.3, 134.2, 128.2, 125.4, 122.5, 79.0, 66.6, 35.4.

HRMS (ESI-TOF) *m/z*: [M + H]$^+$ Calcd for $C_{15}H_{14}N_3O_4^+$ 300.0979; Found 300.0980.

3-hydroxy-5-methyl-8-(3-(methylthio)propanoyl)-2,3-dihydrobenzo[*b*][1,4]oxazepin-4(5*H*)-one 8j

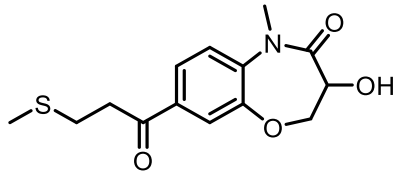

Prepared according to the ***General Procedure C*** to afford **8j** (12.0 mg) in 41% yield as white solid, m.p. = 103.7 – 105.2 ºC.

*NMR and HRMS data for the product **8j**:*

^{1}H NMR (600 MHz, CDCl$_3$) δ (ppm): 7.82 (d, *J* = 8.4 Hz, 1H), 7.75 (s, 1H), 7.29 (d, *J* = 8.4 Hz, 1H), 4.62 (dd, *J* = 10.2, 7.2 Hz, 1H), 4.39 – 4.32 (m, 1H), 4.25 (t, *J* = 10.2 Hz, 1H), 3.58 (brs, 1H), 3.49 (s, 3H), 3.26 (t, *J* = 7.2 Hz, 2H), 2.90 (t, *J* = 7.2 Hz, 2H), 2.17 (s, 3H).

^{13}C NMR (151 MHz, CDCl$_3$) δ (ppm): 196.6, 172.4, 150.4, 140.0, 135.4, 125.1, 122.8, 122.5, 79.0, 66.5, 38.6, 35.4, 28.3, 16.0.

HRMS (ESI-TOF) *m/z*: $[M + H]^+$ Calcd for $C_{14}H_{18}NO_4S^+$ 296.0951; Found 296.0953.

8-acetyl-3-hydroxy-5-methyl-2,3-dihydrobenzo[*b*][1,4]oxazepin-4(5*H*)-one 8k

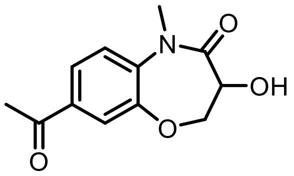

Prepared according to the ***General Procedure C*** to afford **8k** (10.1 mg) in 43% yield as colorless oil.

*NMR and HRMS data for the product **8k***:

^{1}H NMR (600 MHz, $CDCl_3$) δ (ppm): 7.82 (d, *J* = 8.4 Hz, 1H), 7.75 (s, 1H), 7.29 (d, *J* = 8.4 Hz, 1H), 4.62 (dd, *J* = 10.2, 7.2 Hz, 1H), 4.36 (dd, *J* = 10.2, 7.2 Hz, 1H), 4.25 (t, *J* = 10.2 Hz, 1H), 3.58 (brs, 1H), 3.49 (s, 3H), 2.60 (s, 3H).

^{13}C NMR (151 MHz, $CDCl_3$) δ (ppm): 196.2, 172.4, 150.4, 139.9, 136.0, 125.4, 122.8, 122.7, 79.0, 66.5, 35.4, 26.6.

HRMS (ESI-TOF) *m/z*: $[M + H]^+$ Calcd for $C_{12}H_{14}NO_4^+$ 236.0917; Found 236.0922.

8-(3-(4-(*tert*-butyl)phenyl)-2-methylpropanoyl)-3-hydroxy-5-methyl-2,3-dihydrobenzo[*b*][1,4]oxazepin-4(5*H*)-one 8l

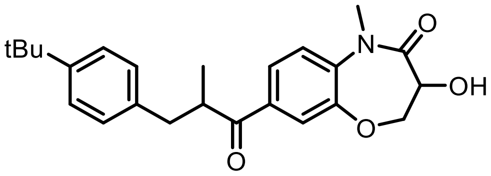

Prepared according to the ***General Procedure C*** to afford **8l** (13.8 mg, a mixture of 1:1 dr isomers) in 35% yield as colorless oil.

*NMR and HRMS data for the product **8l***:

^{1}H NMR (600 MHz, $CDCl_3$) δ (ppm): 7.78 – 7.73 (m, 2H), 7.69 (s, 2H), 7.28 (dd, *J* = 8.4, 3.6 Hz, 4H), 7.24 (d, *J* = 9.0 Hz, 2H), 7.12 (dd, *J* = 8.4, 2.4 Hz, 4H), 4.62 – 4.57 (m, 2H), 4.37 – 4.32 (m, 2H), 4.23 (td, *J* = 10.2, 3.0 Hz, 2H), 3.71 – 3.62 (m, 2H), 3.56 (dd, *J* = 6.6, 2.4 Hz,

2H), 3.48 (s, 6H), 3.11 (dd, *J* = 13.8, 6.6 Hz, 2H), 2.70 – 2.66 (m, 2H), 1.29 (d, *J* = 1.8 Hz, 18H), 1.22 (d, *J* = 6.6 Hz, 6H).

^{13}C NMR (151 MHz, $CDCl_3$) δ (ppm): 202.0, 172.4, 150.4, 149.2, 139.6, 136.5, 135.5, 128.69, 128.67, 125.4, 125.3, 122.17, 122.69, 79.0, 66.5, 42.9, 42.8, 38.88, 38.85, 35.3, 34.4, 31.3, 17.5, 17.4.

HRMS (ESI-TOF) *m/z*: $[M + H]^+$ Calcd for $C_{24}H_{30}NO_4^+$ 396.2169; Found 396.2174.

3-hydroxy-5-methyl-8-(pyrazine-2-carbonyl)-2,3-dihydrobenzo[*b*][1,4]thiazepin-4(5*H*)-one 8m

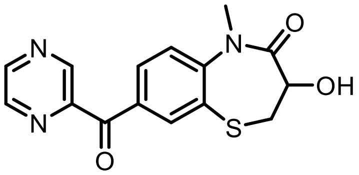

Prepared according to the ***General Procedure C*** to afford **8m** (30.0 mg) in 95% yield as white solid, m.p. = 178.1 – 181.2 ºC.

*NMR and HRMS data for the product **8m***:

^{1}H NMR (600 MHz, $CDCl_3$) δ (ppm): 9.31 (d, *J* = 1.8 Hz, 1H), 8.83 (d, *J* = 2.4 Hz, 1H), 8.71 (t, *J* = 1.8 Hz, 1H), 8.43 (s, 1H), 8.22 (d, *J* = 8.4 Hz, 1H), 7.39 (d, *J* = 8.4 Hz, 1H), 4.22 – 4.17 (m, 1H), 3.73 (dd, *J* = 10.8, 7.2 Hz, 1H), 3.62 (d, *J* = 8.4 Hz, 1H), 3.51 (s, 3H), 2.93 (*t*, J = 10.8 Hz, 1H).

^{13}C NMR (151 MHz, $CDCl_3$) δ (ppm): 190.0, 173.4, 149.9, 149.1, 147.3, 146.3, 142.8, 138.1, 134.1, 132.7, 127.7, 123.7, 67.7, 39.9, 36.5.

HRMS (ESI-TOF) *m/z*: $[M + H]^+$ Calcd for $C_{15}H_{14}N_3O_3S^+$ 316.0750; Found 316.0750.

3-hydroxy-1,5-dimethyl-7-(pyrazine-2-carbonyl)-1,3,4,5-tetrahydro-2H-benzo[b][1,4]diazepin-2-one 8n

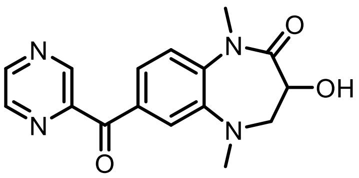

Prepared according to the ***General Procedure C*** to afford **8n** (14.0 mg) in 45% yield yellow oil.

*NMR and HRMS data for the product **8n***:

^{1}H NMR (600 MHz, $CDCl_3$) δ (ppm): 9.26 (d, J = 1.2 Hz, 1H), 8.79 (d, J = 2.4 Hz, 1H), 8.68 (t, J = 1.8 Hz, 1H), 7.84 (d, J = 2.4 Hz, 1H), 7.80 (dd, J = 8.4, 1.2 Hz, 1H), 7.24 (d, J = 8.4 Hz, 1H), 4.14 (dd, J = 10.2, 6.6 Hz, 1H), 3.48-3.45 (m, 4H), 3.36 (t, J = 10.2 Hz, 1H), 3.06 (d, J = 15.0 Hz, 1H), 2.81 (s, 3H).

^{13}C NMR (151 MHz, $CDCl_3$) δ (ppm): 190.9, 173.4, 149.7, 147.0, 146.2, 144.2, 142.8, 140.4, 133.9, 126.2, 122.5, 121.4, 66.2, 65.6, 41.1, 36.2.

HRMS (ESI-TOF) *m/z*: $[M + H]^+$ Calcd for $C_{16}H_{16}N_4O_3^+$ 313.1295; Found 313.1294.

6. Late-stage Functionalization of Complex Bioactive Molecules

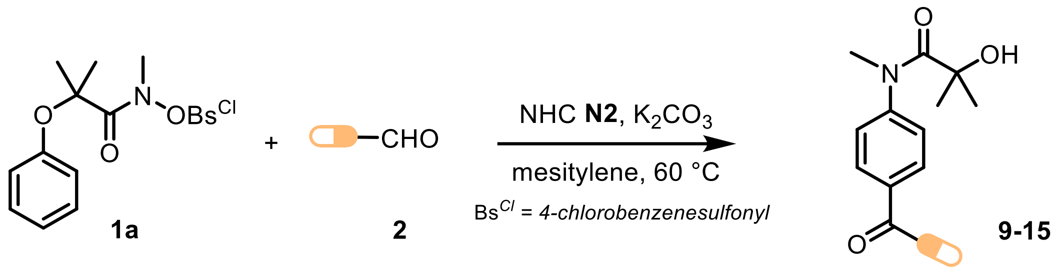

To an oven-dried Schlenck tube was added substrate **1a** (0.10 mmol), NHC **N2** (10 mol %) and K_2CO_3 (0.20 mmol). The Schlenck tube was subjected to three cycles of pressurization/depressurization using dry Ar. After that, under the protection of Ar atmosphere, a solution of aldehydes derived from pharmaceuticals **2** (0.25 mmol) in dry mesitylene (1 mL) was added, and the reaction mixture was stirred at 60 °C for 12 hours. Then the mixture was purified by column chromatography on silica gel to afford the corresponding ketone products **9-15**, which were dried under vacuum and further analyzed by ^{1}H NMR, ^{13}C NMR, HRMS, etc.

4-(4-(2-hydroxy-*N*,2-dimethylpropanamido)benzoyl)phenyl (*R*)-4-((5*S*,8*R*,9*S*,10*S*,13*R*,14*S*,17*R*)-10,13-dimethyl-3,7,12-trioxohexadecahydro-1*H*-cyclopenta[*a*]phenanthren-17-yl) pentanoate 9

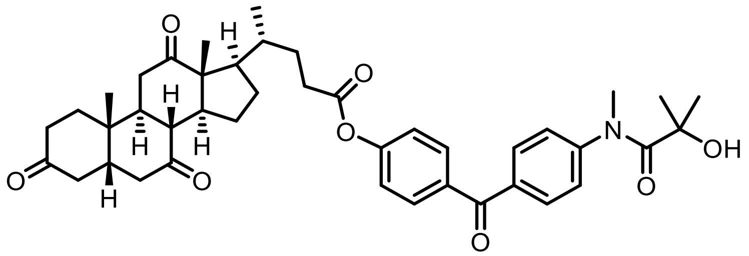

Prepared according to the above procedure to afford **9** (41.2 mg) in 59% yield as white solid, m.p. = 158.1 – 160.2 ºC.

*NMR and HRMS data for the product **9**:*

^{1}H NMR (600 MHz, $CDCl_3$) δ (ppm): 7.86 – 7.80 (m, 4H), 7.36 (d, *J* = 8.4 Hz, 2H), 7.22 (d, *J* = 9.0 Hz, 2H), 4.12 (brs, 1H), 3.36 (s, 3H), 2.92 – 2.82 (m, 3H), 2.70 – 2.65 (m, 1H), 2.58 – 2.53 (m, 1H), 2.37 – 2.18 (m, 6H), 2.15 – 1.93 (m, 7H), 1.89 – 1.83 (m, 1H), 1.61 (td, *J* = 14.4, 4.2 Hz, 1H), 1.56 – 1.49 (m, 1H), 1.41 – 1.35 (m, 5H), 1.31 – 1.22 (m, 7H), 1.08 (s, 3H), 0.91 (d, *J* = 7.2 Hz, 3H).

^{13}C NMR (151 MHz, CDCl$_3$) δ (ppm): 211.9, 209.0, 208.6, 194.2, 176.6, 172.0, 154.2, 147.6, 137.1, 134.3, 131.5, 131.1, 128.2, 121.7, 73.1, 56.8, 51.7, 48.9, 46.7, 45.51, 45.48, 44.9, 42.7, 40.7, 38.6, 36.4, 35.9, 35.4, 35.2, 31.5, 30.2, 28.6, 27.6, 25.1, 21.8, 18.6, 11.8.

HRMS (ESI-TOF) *m/z*: [M+Na]$^+$ Calcd for $C_{42}H_{51}NO_8Na^+$ 720.3507; Found 720.3508.

4-(4-(2-hydroxy-*N*,2-dimethylpropanamido)benzoyl)phenyl 5-(2,5-dimethylphenoxy)-2,2-dimethylpentanoate 10

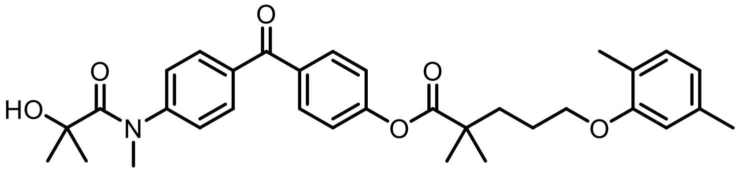

Prepared according to the above procedure to afford **10** (40.4 mg) in 74% yield as colorless oil.

*NMR and HRMS data for the product **10***:

^{1}H NMR (600 MHz, CDCl$_3$) δ (ppm): 7.86 (d, J = 8.4 Hz, 2H), 7.84 (d, J = 8.4 Hz, 2H), 7.38 (d, J = 8.4 Hz, 2H), 7.18 (d, J = 9.0 Hz, 2H), 7.00 (d, J = 7.2 Hz, 1H), 6.66 (d, J = 7.2 Hz, 1H), 6.63 (s, 1H), 4.13 (s, 1H), 4.00 (t, J = 5.4 Hz, 2H), 3.38 (s, 3H), 2.30 (s, 3H), 2.17 (s, 3H), 1.94 – 1.86 (m, 4H), 1.40 (s, 6H), 1.28 (s, 6H).

^{13}C NMR (151 MHz, CDCl$_3$) δ (ppm): 194.2, 176.7, 175.8, 156.8, 154.6, 147.7, 137.2, 136.5, 134.3, 131.5, 131.1, 130.3, 128.3, 123.5, 121.7, 120.8, 111.9, 73.1, 67.6, 42.6, 40.8, 37.1, 28.7, 25.2, 25.1, 21.4, 15.7.

HRMS (ESI-TOF) *m/z*: [M+Na]$^+$ Calcd for $C_{33}H_{39}NO_6Na^+$ 568.2670; Found 568.2677.

(1*R*,2*S*,5*R*)-2-isopropyl-5-methylcyclohexyl 3-(4-(2-hydroxy-*N*,2-dimethylpropanamido)benzoyl)benzoate 11

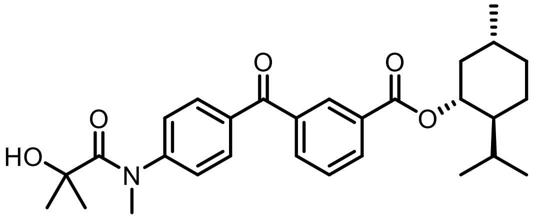

Prepared according to the above procedure to afford **11** (24.5 mg) in 51% yield as colorless oil.

*NMR and HRMS data for the product **11***:

^{1}H NMR (600 MHz, $CDCl_3$) δ (ppm): 8.43 (s, 1H), 8.28 (d, J = 7.2 Hz, 1H), 7.99 (d, J = 7.2 Hz, 1H), 7.88 (d, J = 9.0 Hz, 2H), 7.60 (t, J = 7.8 Hz, 1H), 7.39 (d, J = 8.4 Hz, 2H), 4.96 (td, J = 10.8, 4.2 Hz, 1H), 4.11 (s, 1H), 3.38 (s, 3H), 2.12 – 2.10 (m, 1H), 1.96 – 1.89 (m, 1H), 1.74 – 1.72 (m, 2H), 1.59 – 1.52 (m, 2H), 1.28 (s, 6H), 1.16 – 1.08 (m, 2H), 0.96 – 0.89 (m, 7H), 0.80 (d, J = 7.2 Hz, 3H).

^{13}C NMR (151 MHz, $CDCl_3$) δ (ppm): 194.5, 176.7, 165.1, 147.9, 137.3, 136.7, 133.7, 133.5, 131.3, 130.9, 128.7, 128.4, 75.5, 73.1, 47.2, 40.9, 40.8, 34.2, 31.4, 28.7, 26.6, 23.7, 22.0, 20.7, 16.6.

HRMS (ESI-TOF) *m/z*: $[M+Na]^+$ Calcd for $C_{29}H_{37}NO_5Na^+$ 502.2564; Found 502.2567.

4-(4-(2-hydroxy-*N*,2-dimethylpropanamido)benzoyl)phenyl 3,6-dichloro-2-methoxybenzoate 12

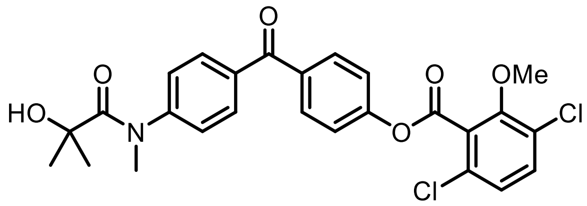

Prepared according to the above procedure to afford **12** (33.0 mg) in 64% yield as white solid, m.p. = 105.9 – 108.7 °C.

*NMR and HRMS data for the product **12***:

^{1}H NMR (600 MHz, $CDCl_3$) δ (ppm): 7.91 (d, J = 9.0 Hz, 2H), 7.88 (d, J = 7.8 Hz, 2H), 7.45 (d, J = 9.0 Hz, 1H), 7.42 (d, J = 8.4 Hz, 2H), 7.39 (d, J = 8.4 Hz, 2H), 7.21 (d, J = 8.4 Hz, 1H), 4.13 (brs, 1H), 4.01 (s, 3H), 3.38 (s, 3H), 1.28 (s, 6H).

^{13}C NMR (151 MHz, $CDCl_3$) δ (ppm): 194.1, 176.7, 162.6, 154.1, 153.8, 147.8, 137.0, 135.1, 132.6, 131.7, 131.2, 129.8, 129.3, 128.3, 126.9, 126.0, 121.7, 73.1, 62.4, 40.8, 28.7.

HRMS (ESI-TOF) *m/z*: $[M + Na]^+$ Calcd for $C_{26}H_{23}{}^{35}Cl^{35}ClNO_6Na^+$ 538.0795, $C_{26}H_{23}{}^{37}Cl^{35}ClNO_6Na^+$ 540.0766; Found 538.0805, 540.0762.

4-(4-(2-hydroxy-*N*,2-dimethylpropanamido)benzoyl)phenyl stearate 13

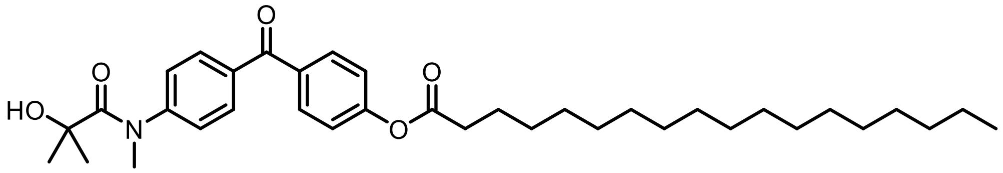

Prepared according to the above procedure to afford **13** (31.9 mg) in 43% yield as white solid, m.p. = 68.6 – 70.3 ºC.

*NMR and HRMS data for the product **13***:

^{1}H NMR (600 MHz, CDCl$_3$) δ (ppm): 7.88 – 7.80 (m, 4H), 7.36 (d, *J* = 8.4 Hz, 2H), 7.22 (d, *J* = 9.0 Hz, 2H), 4.13 (s, 1H), 3.36 (s, 3H), 2.58 (t, *J* = 7.2 Hz, 2H), 1.78 – 1.73 (m, 2H), 1.43 – 1.38 (m, 2H), 1.37 – 1.24 (m, 32H), 0.86 (t, *J* = 6.6 Hz, 3H).

^{13}C NMR (151 MHz, CDCl$_3$) δ (ppm): 194.1, 176.6, 171.7, 154.3, 147.7, 137.1, 134.3, 131.5, 131.1, 128.2, 121.7, 73.1, 49.0, 40.7, 34.3, 33.9, 31.8, 29.61, 29.57, 29.5, 29.4, 29.3, 29.2, 29.0, 28.6, 25.6, 24.9, 24.8, 22.6, 14.0.

HRMS (ESI-TOF) *m/z*: [M + Na]$^+$ Calcd for $C_{36}H_{53}NO_5Na^+$ 602.3816; Found 602.3824.

4-(4-(2-hydroxy-*N*,2-dimethylpropanamido)benzoyl)phenyl 2-(4-isobutylphenyl)propanoate 14

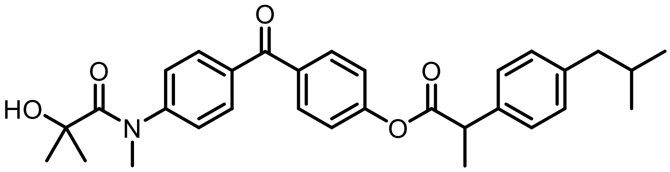

Prepared according to the above procedure to afford **14** (26.1 mg) in 52% yield as white solid, m.p. = 79.8 – 83.5 ºC.

*NMR and HRMS data for the product **14***:

^{1}H NMR (600 MHz, CDCl$_3$) δ (ppm): 7.84 (d, *J* = 9.0 Hz, 2H), 7.81 (d, *J* = 9.0 Hz, 2H), 7.36 (d, *J* = 7.8 Hz, 2H), 7.30 (d, *J* = 8.4 Hz, 2H), 7.18 – 7.12 (m, 4H), 4.14 (s, 1H), 3.97 (q, *J* = 7.2 Hz, 1H), 3.36 (s, 3H), 2.47 (d, *J* = 7.2 Hz, 2H), 1.90 – 1.83 (m, 1H), 1.62 (d, *J* = 6.6 Hz, 3H), 1.26 (s, 6H), 0.91 (d, *J* = 6.0 Hz, 6H).

^{13}C NMR (151 MHz, $CDCl_3$) δ (ppm): 194.2, 176.7, 172.7, 154.4, 147.6, 141.1, 137.2, 136.8, 134.4, 131.5, 131.2, 129.6, 128.3, 127.2, 121.6, 73.1, 45.3, 45.0, 40.8, 30.2, 28.7, 22.4, 18.4.

HRMS (ESI-TOF) *m/z*: $[M + Na]^+$ Calcd for $C_{31}H_{35}NO_5Na^+$ 524.2407; Found 524.2413.

<u>3-(4-(2-hydroxy-*N*,2-dimethylpropanamido)benzoyl)phenyl 2-((3-chloro-2-methylphenyl)amino)benzoate 15</u>

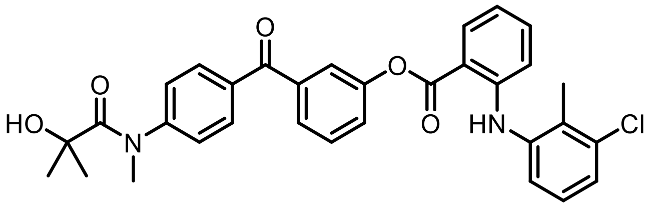

Prepared according to the above procedure (1,4-dioxane as the solvent) to afford **15** (38.0 mg) in 68% yield as white solid, m.p. = 81.5 – 83.9 ºC.

*NMR and HRMS data for the product **15***:

^{1}H NMR (600 MHz, $CDCl_3$) δ (ppm): 9.18 (s, 1H), 8.21 (d, *J* = 8.4 Hz, 1H), 7.92 (d, *J* = 9.0 Hz, 2H), 7.73 (d, *J* = 8.4 Hz, 1H), 7.69 (s, 1H), 7.60 (t, *J* = 7.8 Hz, 1H), 7.50 (d, *J* = 8.4 Hz, 1H), 7.40 – 7.36 (m, 3H), 7.24 (d, *J* = 8.4 Hz, 2H), 7.14 (t, *J* = 7.8 Hz, 1H), 6.84 (d, *J* = 8.4 Hz, 1H), 6.81 (t, *J* = 7.8 Hz, 1H), 4.07 (brs, 1H), 3.38 (s, 3H), 2.31 (s, 3H), 1.28 (s, 6H).

^{13}C NMR (151 MHz, $CDCl_3$) δ (ppm): 194.1, 176.7, 167.0, 150.7, 149.6, 147.9, 139.9, 138.5, 136.8, 135.7, 135.4, 132.0, 131.9, 131.3, 129.7, 128.4, 127.5, 127.0, 126.5, 126.2, 123.49, 123.46, 117.1, 114.0, 109.8, 73.1, 40.8, 28.7, 15.0.

HRMS (ESI-TOF) *m/z*: $[M + Na]^+$ Calcd for $C_{32}H_{29}{}^{35}ClN_2O_5Na^+$ 579.1657, $C_{32}H_{29}{}^{37}ClN_2O_5Na^+$ 581.1628; Found 579.1666, 581.1637.

7. Synthetic Applications

i) *In-situ removal of the side chain*

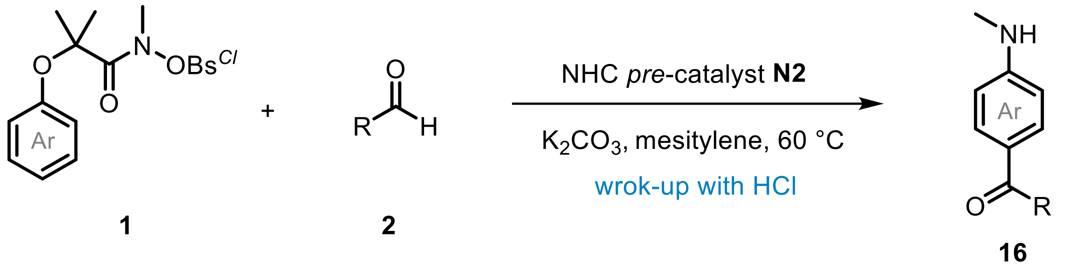

To an oven-dried Schlenck tube was added substrates **1** (0.10 mmol), NHC **N2** (10 mol %) and K_2CO_3 (0.20 mmol). The Schlenck tube was subjected to three cycles of pressurization/depressurization using dry Ar. After that, under the protection of Ar atmosphere, a solution of aldehydes **2** (0.25 mmol) in dry mesitylene (1 mL) was added, and the reaction mixture was stirred at 60 °C for 12 hours. Then, to the reaction mixture was added 1.5 mL HCl solution (in 1,4-dioxane, 4 M) slowly, and further stirred at 60 °C for 5 h. The resulting mixture was diluted with water (5.0 mL) and extracted with ethyl acetate (3 × 10 mL), washed with 1M $NaHCO_3$ aq. (2 × 20 mL), and the aqueous layer was extracted with ethyl acetate (3 × 10 mL). The organic layer was combined and dried over sodium sulfate and filtered. The filtrate was evaporated in vacuo to give a residue, which was purified by column chromatography on silica gel to afford the compound **16**. The product was finally dried under vacuum and further analyzed by ^{1}H NMR, ^{13}C NMR, HRMS, etc.

1-(4-(methylamino)phenyl)-3-phenylpropan-1-one 16a

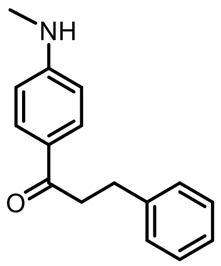

Prepared according to the above procedure to afford **16a** (18.7 mg) in 78% yield as white solid, m.p. = 100.6 – 103.0 °C.

NMR and HRMS data for the product ***16a***:

^{1}H NMR (600 MHz, CDCl$_3$) δ (ppm): 7.85 (d, *J* = 9.0 Hz, 2H), 7.30 (t, *J* = 7.8 Hz, 2H), 7.26 (d, *J* = 7.2 Hz, 2H), 7.20 (t, *J* = 7.2 Hz, 1H), 6.55 (d, *J* = 9.0 Hz, 2H), 4.30 (brs, 1H), 3.20 (t, *J*

= 7.8 Hz, 2H), 3.05 (t, J = 7.8 Hz, 2H), 2.89 (s, 3H).

^{13}C NMR (151 MHz, $CDCl_3$) δ (ppm): 197.4, 153.0, 141.8, 130.5, 128.4, 126.1, 125.9, 111.1, 39.7, 30.6, 30.1.

HRMS (ESI-TOF) *m/z*: $[M + Na]^+$ Calcd for $C_{16}H_{17}NONa^+$ 262.1202; Found 262.1205.

(4-(methylamino)phenyl)(phenyl)methanone 16b

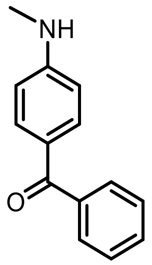

Prepared according to the above procedure to afford **16b** (18.0 mg) in 85% yield as colorless oil.

*NMR and HRMS data for the product **16b***:

^{1}H NMR (600 MHz, $CDCl_3$) δ (ppm): 7.76 (d, J = 9.0 Hz, 2H), 7.72 (d, J = 7.8 Hz, 2H), 7.53 (t, J = 7.2 Hz, 1H), 7.45 (t, J = 7.8 Hz, 2H), 6.59 (d, J = 9.0 Hz, 2H), 4.33 (s, 1H), 2.92 (s, 3H).

^{13}C NMR (151 MHz, $CDCl_3$) δ (ppm): 195.2, 153.0, 139.2, 133.0, 131.2, 129.4, 128.0, 126.1, 111.0, 30.1.

HRMS (ESI-TOF) *m/z*: $[M + H]^+$ Calcd for $C_{14}H_{13}NOH^+$ 212.1070; Found 212.1064.

(4-(methylamino)phenyl)(thiophen-2-yl)methanone 16c

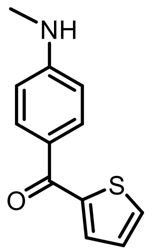

Prepared according to the above procedure to afford **16c** (16.1 mg) in 74% yield as colorless oil.

*NMR and HRMS data for the product **13c***:

^{1}H NMR (600 MHz, $CDCl_3$) δ (ppm): 7.84 (d, J = 7.8 Hz, 2H), 7.62 (d, J = 4.8 Hz, 2H), 7.12 (s, 1H), 6.59 (d, J = 8.4 Hz, 2H), 4.39 (brs, 1H), 2.90 (s, 3H).

^{13}C NMR (151 MHz, $CDCl_3$) δ (ppm): 186.3, 152.9, 144.3, 133.0, 132.3, 132.0, 127.4, 126.4,

111.1, 30.1.

HRMS (ESI-TOF) *m/z*: $[M + H]^+$ Calcd for $C_{12}H_{11}NOSH^+$ 218.0634; Found 218.0627..

(3-fluoro-4-methoxyphenyl)(4-(methylamino)phenyl)methanone 16d

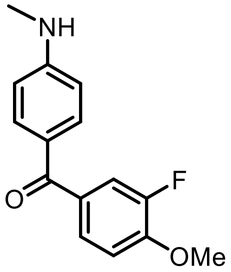

Prepared according to the above procedure to afford **16d** (20.0 mg) in 77% yield as colorless oil.

*NMR and HRMS data for the product **16d***:

^{1}H NMR (600 MHz, $CDCl_3$) δ (ppm): 7.72 (d, J = 8.4 Hz, 2H), 7.40 (d, J = 8.4 Hz, 1H), 7.26-7.24 (m, 1H), 7.11 (t, J = 9.6 Hz, 1H), 6.58 (d, J = 8.4 Hz, 2H), 4.34 (brs, 1H), 3.92 (s, 3H), 2.91 (s, 3H).

^{13}C NMR (151 MHz, $CDCl_3$) δ (ppm): 193.8, 154.4 (C-F, $^1J_{C\text{-}F}$ =251.6 Hz), 153.0, 147.6 (C-F, $^3J_{C\text{-}F}$ =10.1 Hz), 135.5 (C-F, $^4J_{C\text{-}F}$ =2.9 Hz), 132.9, 125.8, 123.1 (C-F, $^3J_{C\text{-}F}$ =7.2 Hz), 115.2 (C-F, $^2J_{C\text{-}F}$ =18.9 Hz), 114.5, 111.0, 56.4, 30.1.

^{19}F NMR (564 MHz, $CDCl_3$) δ (ppm): -130.07 – -130.12 (m, 1F).

HRMS (ESI-TOF) *m/z*: $[M + H]^+$ Calcd for $C_{15}H_{14}FNO_2H^+$ 260.1081; Found 260.1075..

(3-methyl-4-(methylamino)phenyl)(phenyl)methanone 16e

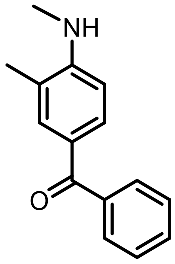

Prepared according to the above procedure to afford **16e** (18.0 mg) in 80% yield as colorless oil.

*NMR and HRMS data for the product **16e***:

^{1}H NMR (600 MHz, $CDCl_3$) δ (ppm): 7.72 (d, J = 7.8 Hz, 2H), 7.69-7.67 (m, 2H), 7.53 (t, J = 7.8 Hz, 1H), 7.45 (t, J = 7.8 Hz, 2H), 6.57 (d, J = 8.4 Hz, 1H), 4.18 (brs, 1H), 2.97 (s, 3H),

2.16 (s, 3H).

^{13}C NMR (151 MHz, CDCl$_3$) δ (ppm): 195.5, 151.2, 139.3, 132.4, 131.7, 131.1, 129.4, 128.0, 125.5, 120.8, 107.4, 30.3, 17.2.

HRMS (ESI-TOF) *m/z*: $[M + H]^+$ Calcd for $C_{15}H_{15}NOH^+$ 226.1226; Found 226.1220.

<u>(4-(methylamino)-5,6,7,8-tetrahydronaphthalen-1-yl)(quinoxalin-2-yl)methanone 16f</u>

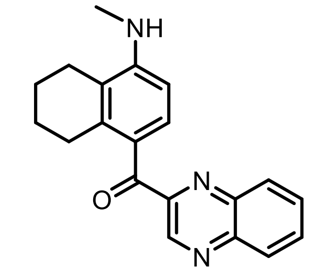

Prepared according to the above procedure to afford **16f** (24.7 mg) in 78% yield as red oil.

*NMR and HRMS data for the product **16f***:

^{1}H NMR (600 MHz, CDCl$_3$) δ (ppm): 9.34 (s, 1H), 8.16 (m, 2H), 7.85-7.78 (m, 2H), 7.48 (d, J = 8.4 Hz, 1H), 6.40 (d, J = 8.4 Hz, 1H), 4.29 (brs, 1H), 3.16 (t, J = 6.6 Hz, 2H), 2.94 (s, 3H), 2.40 (t, J = 6.6 Hz, 2H), 1.89-1.85 (m, 2H), 1.76-1.72 (m, 2H).

^{13}C NMR (151 MHz, CDCl$_3$) δ (ppm): 193.2, 151.8, 150.7, 145.5, 142.6, 141.7, 140.6, 134.6, 131.1, 130.4, 130.2, 129.2, 123.3, 121.3, 104.4, 30.3, 29.1, 24.2, 22.5, 22.2.

HRMS (ESI-TOF) *m/z*: $[M + H]^+$ Calcd for $C_{20}H_{19}N_3OH^+$ 318.1061; Found318.1598..

ii) *Oxidation of the alkyl ketone*

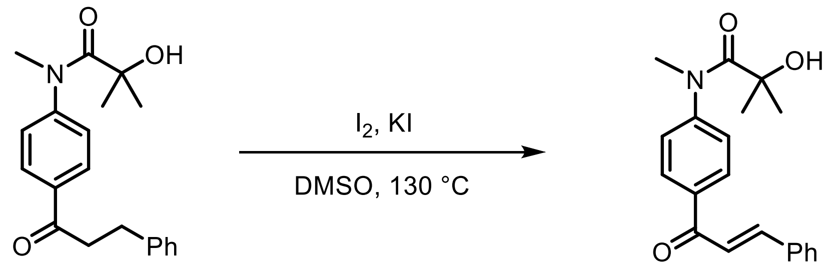

3a **17**

To an oven-dried Schlenck tube was added I_2 (0.25 mmol) and KI (1.0 mmol). The Schlenck tube was subjected to three cycles of pressurization/depressurization using dry Ar. After that, under the protection of Ar atmosphere, a solution of product **3a** (0.1 mmol) in DMSO (1 mL) was added, and the reaction mixture was stirred at 130 °C for 16 h. Then the resulting mixture

was diluted with water (5.0 mL) and extracted with ethyl acetate (3 × 10 mL). The organic layer was combined and dried over sodium sulfate and filtered. The filtrate was evaporated in vacuo to give a residue, then the mixture was purified by column chromatography on silica gel to afford the compound **17**, which were dried under vacuum and further analyzed by ^{1}H NMR, ^{13}C NMR, HRMS, etc.

N-(4-cinnamoylphenyl)-2-hydroxy-N,2-dimethylpropanamide 17

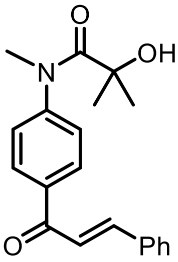

Prepared according to the above procedure to afford **17** (21.0 mg) in 65% yield as light yellow oil.

*NMR and HRMS data for the product **17***:

^{1}H NMR (600 MHz, $CDCl_3$) δ (ppm): 8.08 (d, *J* = 8.4 Hz, 2H), 7.85 (d, *J* = 15.6 Hz, 1H), 7.66 (d, *J* = 7.8 Hz, 2H), 7.53 (d, *J* = 16.2 Hz, 1H), 7.45 – 7.42 (m, 3H), 7.40 (d, *J* = 9.0 Hz, 2H), 4.16 (brs, 1H), 3.36 (s, 3H), 1.25 (s, 6H).

^{13}C NMR (151 MHz, $CDCl_3$) δ (ppm): 189.1, 176.7, 147.9, 145.7, 137.9, 134.6, 130.9, 129.7, 129.0, 128.7, 128.6, 121.4, 73.1, 40.8, 28.7.

HRMS (ESI-TOF) *m/z*: $[M + H]^+$ Calcd for $C_{20}H_{22}NO_3^+$ 324.1594; Found 324.1598.

iii) *DIBAL-H reduction*

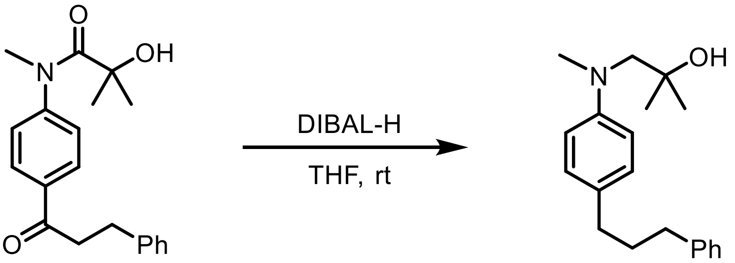

3a **18**

Under Ar atmosphere, product **3a** (0.1 mmol) was dissolved into THF (0.5 mL). To the solution, DIBAL-H (1 mL, 1.0 M in hexane) was added dropwise, and the reaction mixture was stirred at room temperature for 48 h. The solution was filtered, then evaporated in vacuo to give

a residue. The mixture was purified by column chromatography on silica gel to afford the compound **18**, which were dried under vacuum and further analyzed by ^{1}H NMR, ^{13}C NMR, HRMS, etc.

2-methyl-1-(methyl(4-(3-phenylpropyl)phenyl)amino)propan-2-ol 18

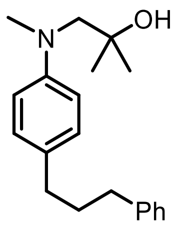

Prepared according to the above procedure to afford **18** (22.0 mg) in 74% yield as colorless oil.

NMR and HRMS data for the product ***18***:

^{1}H NMR (600 MHz, CDCl$_3$) δ (ppm): 7.29 (t, J = 7.8 Hz, 2H), 7.21 – 7.18 (m, 3H), 7.07 (d, J = 9.0 Hz, 2H), 6.84 (d, J = 9.0 Hz, 2H), 3.27 (s, 2H), 3.01 (s, 3H), 2.66 (t, J = 7.8 Hz, 2H), 2.58 (t, J = 7.8 Hz, 2H), 1.96 – 1.91 (m, 3H), 1.30 (s, 6H).

^{13}C NMR (151 MHz, CDCl$_3$) δ (ppm): 149.3, 142.5, 130.8, 128.9, 128.4, 128.2, 125.6, 112.8, 72.6, 64.8, 41.2, 35.4, 34.3, 33.2, 27.8.

HRMS (ESI-TOF) *m/z*: $[M + H]^+$ Calcd for $C_{20}H_{28}NO^+$ 298.2165; Found 298.2166.

iv) *Selective reduction of the ketone*

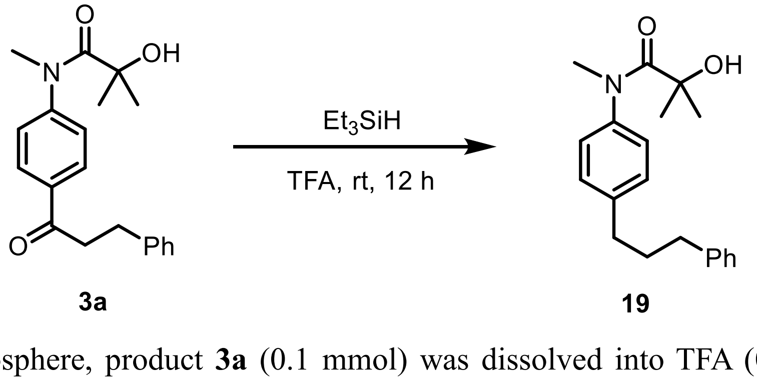

Under Ar atmosphere, product **3a** (0.1 mmol) was dissolved into TFA (0.67 mL). To the solution, triethylsilane (0.58 mmol) was added dropwise, and the reaction mixture was stirred at room temperature for 12 h. The reaction was neutralized by saturated $NaHCO_3$ aq., and extracted with ethyl acetate (3 × 10 mL). The combined organic layer was dried over sodium sulfate and filtered. The filtrate was evaporated in vacuo to give a residue, then the mixture was

purified by column chromatography on silica gel to afford the compound **19**, which were dried under vacuum and further analyzed by ^{1}H NMR, ^{13}C NMR, HRMS, etc.

2-hydroxy-*N*,2-dimethyl-*N*-(4-(3-phenylpropyl)phenyl)propanamide 19

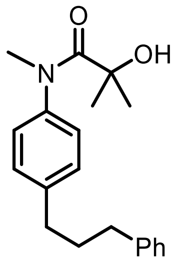

Prepared according to the above procedure to afford **19** (24.0 mg) in 77% yield as colorless oil.

*NMR and HRMS data for the product **19***:

^{1}H NMR (600 MHz, $CDCl_3$) δ (ppm): 7.29 (t, *J* = 7.8 Hz, 2H), 7.24 – 7.17 (m, 5H), 7.14 (d, *J* = 8.4 Hz, 2H), 4.53 (s, 1H), 3.30 (s, 3H), 2.72 – 2.63 (m, 4H), 2.00 – 1.95 (m, 2H), 1.15 (s, 6H).

^{13}C NMR (151 MHz, $CDCl_3$) δ (ppm): 177.0, 143.0, 141.8, 141.3, 129.3, 128.44, 128.36, 125.9, 72.7, 41.0, 35.3, 34.9, 32.8, 28.6.

HRMS (ESI-TOF) *m/z*: $[M + Na]^+$ Calcd for $C_{20}H_{25}NO_2Na^+$ 334.1778; Found 334.1773.

v) *The construction of oxindole skeleton*

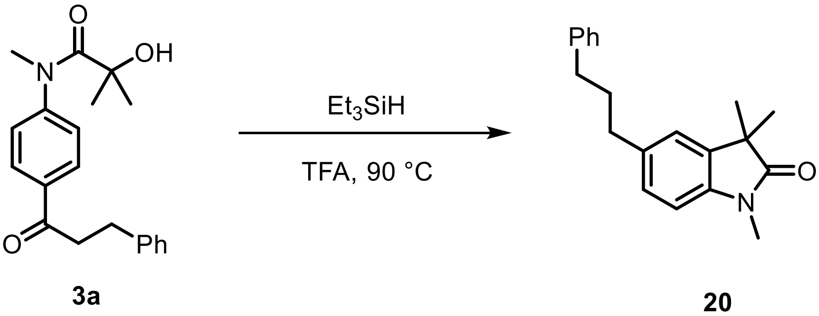

3a **20**

Under Ar atmosphere, product **3a** (0.1 mmol) was dissolved into TFA (0.67 mL). To the solution, triethylsilane (0.58 mmol) was added dropwise, and the reaction mixture was stirred at 90 °C for 12 h. The reaction was neutralized by saturated $NaHCO_3$ aq., and extracted with ethyl acetate (3 × 10 mL). The combined organic layer was dried over sodium sulfate and filtered. The filtrate was evaporated in vacuo to give a residue, then the mixture was purified

by column chromatography on silica gel to afford the compound **20**, which were dried under vacuum and further analyzed by ^{1}H NMR, ^{13}C NMR, HRMS, etc.

1,3,3-trimethyl-5-(3-phenylpropyl)indolin-2-one 20

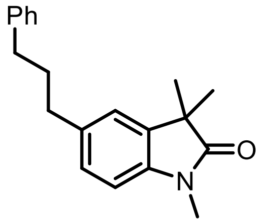

Prepared according to the above procedure to afford **20** (12.0 mg) in 41% yield as colorless oil.

*NMR and HRMS data for the product **20***:

^{1}H NMR (600 MHz, $CDCl_3$) δ (ppm): 7.29 (t, *J* = 7.8 Hz, 2H), 7.23 – 7.17 (m, 3H), 7.07 (d, *J* = 7.8 Hz, 1H), 7.03 (s, 1H), 6.76 (d, *J* = 7.8 Hz, 1H), 3.20 (s, 3H), 2.68 – 2.63 (m, 4H), 1.98 – 1.92 (m, 2H), 1.36 (s, 6H).

^{13}C NMR (151 MHz, $CDCl_3$) δ (ppm): 181.4, 142.2, 140.6, 136.6, 135.9, 128.4, 128.3, 127.3, 125.8, 122.5, 107.7, 44.2, 35.4, 35.2, 33.3, 26.2, 24.4.

HRMS (ESI-TOF) *m/z*: $[M + Na]^+$ Calcd for $C_{20}H_{23}NONa^+$ 316.1672; Found 316.1678.

vi) *The intramolecular C-O coupling reaction*

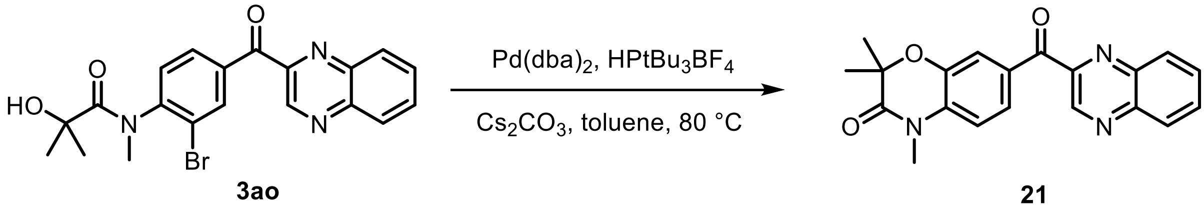

To an oven-dried Schlenck tube was added $Pd(dba)_2$ (1 mol %), $HPtBu_3BF_4$ (2 mol %) and Cs_2CO_3 (0.12 mmol). The Schlenck tube was subjected to three cycles of pressurization/depressurization using dry Ar. After that, under the protection of Ar atmosphere, dry toluene (1 mL) was added, and the reaction mixture was stirred at 80 °C for 30 min. Then resulting solution was cooled to room temperature, and added compound **3ao** (0.1 mmol) under the protection of Ar atmosphere, and the reaction mixture was stirred at 80 °C for 16 h. Then the mixture was purified by column chromatography on silica gel to afford the corresponding ketone products **21**, which were dried under vacuum and further analyzed by ^{1}H NMR, ^{13}C

NMR, HRMS, etc.

2,2,4-trimethyl-7-(quinoxaline-2-carbonyl)-2*H*-benzo[*b*][1,4]oxazin-3(4*H*)-one 21

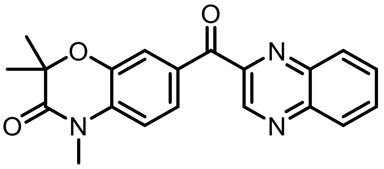

Prepared according to the above procedure to afford **21** (32.0 mg) in 92% yield as white solid, m.p. = 128.7 – 131.1 °C.

*NMR and HRMS data for the product **21***:

^{1}H NMR (600 MHz, CDCl$_3$) δ (ppm): 9.46 (s, 1H), 8.21 (t, *J* = 9.0 Hz, 2H), 8.03 (d, *J* = 7.2 Hz, 1H), 7.93 – 7.90 (m, 2H), 7.87 (t, *J* = 7.2 Hz, 1H), 7.07 (d, *J* = 8.4 Hz, 1H), 3.42 (s, 3H), 1.54 (s, 6H).

^{13}C NMR (151 MHz, CDCl$_3$) δ (ppm): 190.2, 169.1, 148.7, 145.3, 143.1, 142.9, 140.3, 134.6, 132.0, 130.9, 130.8, 130.4, 129.4, 126.7, 120.1, 113.8, 78.1, 28.9, 24.0.

HRMS (ESI-TOF) *m/z*: [M + Na]$^+$ Calcd for $C_{20}H_{17}N_3O_3Na^+$ 370.1162; Found 370.1159.

vii) *Oxidation of the alcohol*

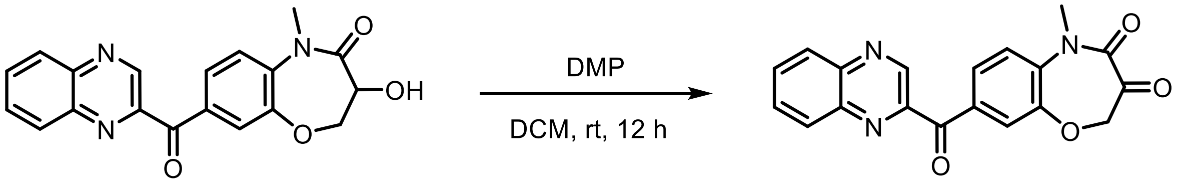

5h **22**

To a solution of product **5h** (0.1 mmol) in dichloromethane (1 mL) was added Dess-Martin periodinane (0.5 mmol) at 0 °C. The reaction mixture was stirred at room temperature for 12 h. Then the reaction was quenched with a saturated $NaHCO_3$ solution containing 10 % $Na_2S_2O_3$. The organic layer was then washed with saturated brine solution (2 × 10 mL), dried over anhydrous sodium sulfate and filtered. The filtrate was evaporated in vacuo to give a residue, then the mixture was purified by column chromatography on silica gel to afford the compound **22**, which were dried under vacuum and further analyzed by ^{1}H NMR, ^{13}C NMR, HRMS, etc.

5-methyl-8-(quinoxaline-2-carbonyl)benzo[*b*][1,4]oxazepine-3,4(2*H*,5*H*)-dione 22

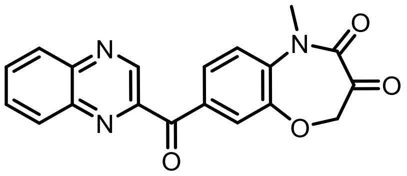

Prepared according to the above procedure to afford **22** (24.3 mg) in 70% yield as light red solid, m.p. = 157.2 – 158.1 ºC.

*NMR and HRMS data for the product **22***:

^{1}H NMR (600 MHz, $CDCl_3$) δ (ppm): 9.53 (s, 1H), 8.27 (d, *J* = 8.4 Hz, 1H), 8.23 – 8.20 (m, 3H), 7.94 (t, *J* = 7.8 Hz, 1H), 7.90 (t, *J* = 7.8 Hz, 1H), 7.44 (d, *J* = 8.4 Hz, 1H), 4.90 (s, 2H), 3.54 (s, 3H).

^{13}C NMR (151 MHz, $CDCl_3$) δ (ppm): 199.3, 189.6, 165.7, 151.2, 147.7, 145.2, 143.3, 140.24, 140.17, 134.4, 132.5, 131.1, 130.4, 130.0, 129.5, 126.1, 123.2, 83.3, 35.0.

HRMS (ESI-TOF) *m/z*: $[M + Na]^+$ Calcd for $C_{19}H_{13}N_3O_4Na^+$ 370.0798; Found 370.0799.

8. Mechanism Studies

i) *The control experiments with radical scavenger TEMPO*

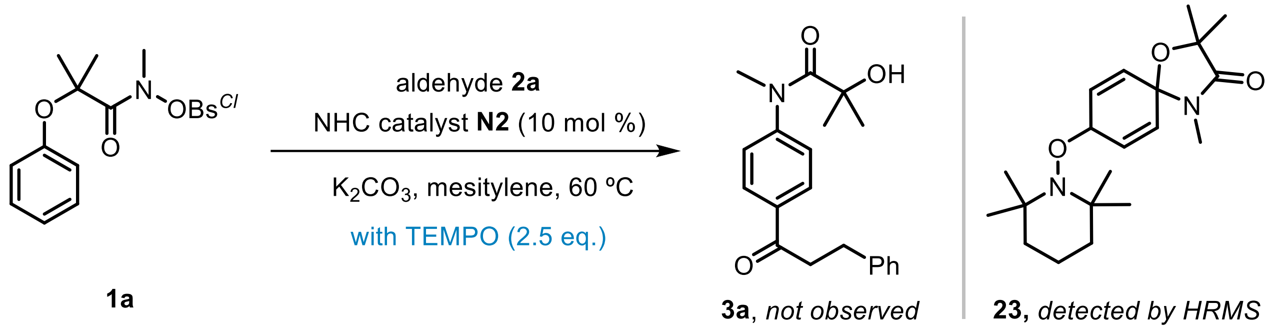

1a **3a**, *not observed* **23,** *detected by HRMS*

To an oven-dried Schlenck tube was added substrate **1a** (0.10 mmol), NHC **N2** (10 mol %) and K_2CO_3 (0.2 mmol), TEMPO (0.25 mmol). The Schlenck tube was subjected to three cycles of pressurization/depressurization using dry Ar. After that, under the protection of Ar atmosphere, a solution of aldehyde **2a** (0.25 mmol) in dry mesitylene (1 mL) was added and the reaction mixture was stirred at 60 °C for 12 hours, and no product **3a** was observed. Then the mixture was monitored and analyzed by HRMS analysis, the desired product **3a** was not observed. Instead, a TEMPO-trapped intermediate **23** was detected by HRMS.

HRMS (ESI-TOF) m/z: $[M + H]^+$ calculated for $C_{20}H_{333}N_2O_3^+$: 349.2486, found: 349.2485.

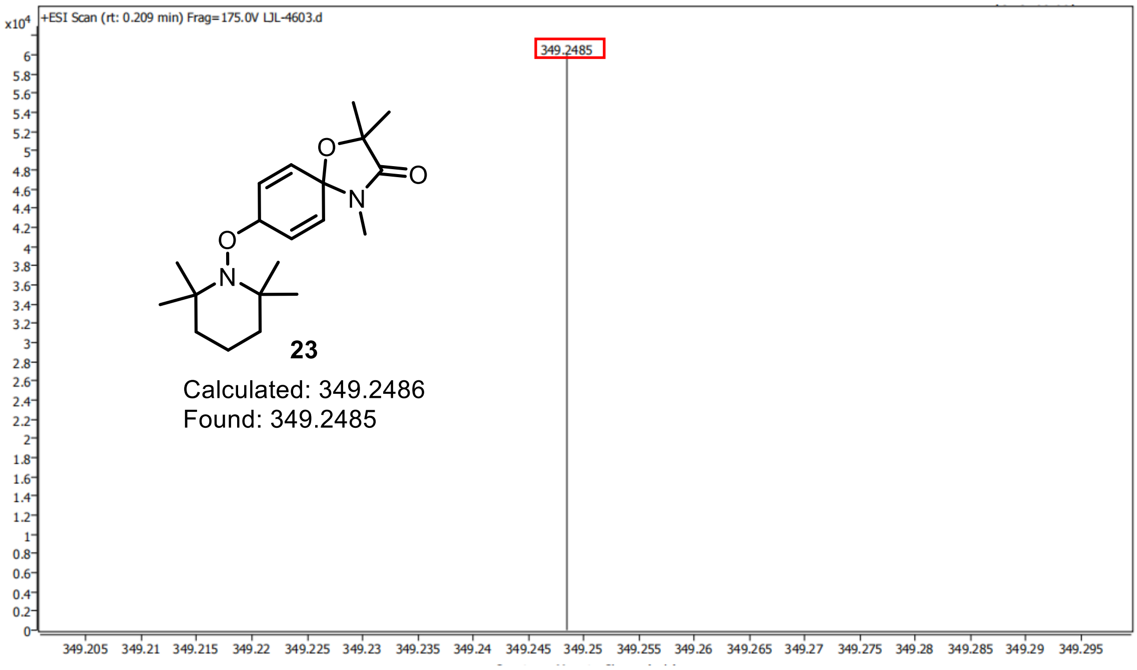

ii) *Radical clock experiment*

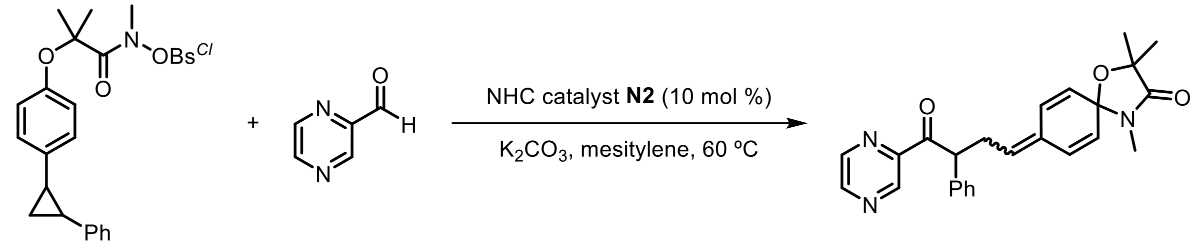

1b

24, 24%, E/Z = 1.4:1

To an oven-dried Schlenck tube was added substrate **1b** (0.10 mmol), NHC **N2** (10 mol %) and K_2CO_3 (0.2 mmol). The Schlenck tube was subjected to three cycles of pressurization/depressurization using dry Ar. After that, under the protection of Ar atmosphere, a solution of pyrazinyl aldehyde (0.25 mmol) in mesitylene (1 mL) was added and the reaction mixture was stirred at 60 °C for 12 hours. After the reaction finished, the resulting mixture were purified by column chromatography on silica gel eluting from petroleum ether/ethyl acetate to afford the product **24** in 24% yield as white solid.

<u>2,2,4-trimethyl-8-(4-oxo-3-phenyl-4-(pyrazin-2-yl)butylidene)-1-oxa-4-azaspiro[4.5]deca-6,9-dien-3-one 24</u>

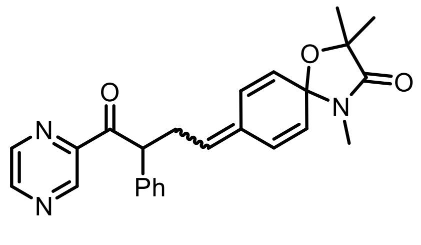

Prepared according to the above procedure to afford **24** (10.0 mg, a mixture of 1.4:1 isomers) in 24% yield as white solid, m.p. = 143.5 – 154.4 °C.

*NMR and HRMS data for the product **24***:

^{1}H NMR (600 MHz, $CDCl_3$) δ (ppm): 9.18 (d, J = 1.2 Hz, 1H), 8.68-8.67 (m, 1H), 8.59-8.58 (m, 1H), 7.34-7.32 (m, 2H), 7.28-7.24 (m, 2H), 7.21-7.17 (m, 1H), 6.83 (dd, J = 10.2, 1.2 Hz, 1H), 6.35-6.32 (m, 1H), 5.73-5.68 (m, 1H), 5.60-5.58 (m, 1H), 5.47-5.43 (m, 1H), 5.32-5.27 (m, 1H), 3.24-3.14 (m, 1H), 2.94-2.87 (m, 1H), 2.54 (s, 3H), 1.45-1.43 (m, 6H).

^{13}C NMR (151 MHz, $CDCl_3$) δ (ppm): 199.3, 173.3, 147.7, 146.9, 144.5, 143.4, 137.1, 136.0, 133.1, 128.9, 128.8, 128.1, 127.5, 125.7, 87.8, 79.4, 51.2, 30.8, 26.8, 25.5.

HRMS (ESI-TOF) *m/z*: $[M + H]^+$ Calcd for $C_{25}H_{25}N_3O_3H^+$ 416.1969; Found 416.1978.

iii) *Isolation of the dearomatized intermediate*

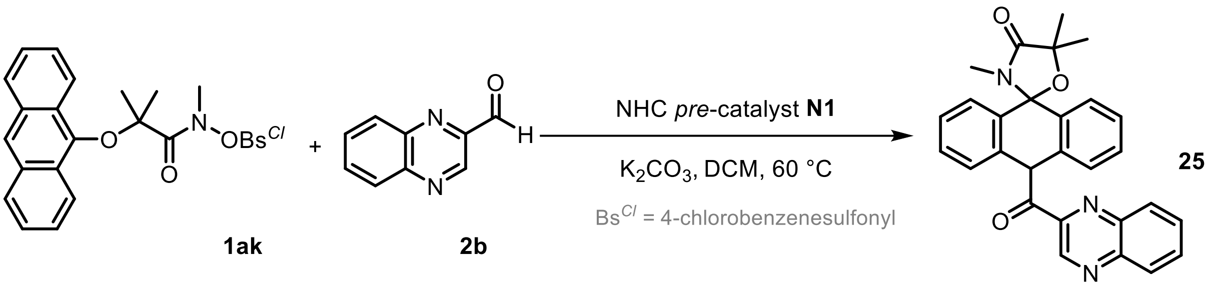

To an oven-dried Schlenck tube was added substrate **1ak** (0.10 mmol), NHC **N2** (10 mol %) and K_2CO_3 (0.20 mmol). The Schlenck tube was subjected to three cycles of pressurization/depressurization using dry Ar. After that, under the protection of Ar atmosphere, a solution of aldehyde **2b** (0.25 mmol) in dry DCM (1 mL) was added, and the reaction mixture was stirred at 60 °C for 12 hours. Then the mixture was purified by column chromatography on silica gel to afford the corresponding product **24**, which were dried under vacuum and further analyzed by ^{1}H NMR, ^{13}C NMR, HRMS, etc.

3',5',5'-trimethyl-10-(quinoxaline-2-carbonyl)-10H-spiro[anthracene-9,2'-oxazolidin]-4'-one 25

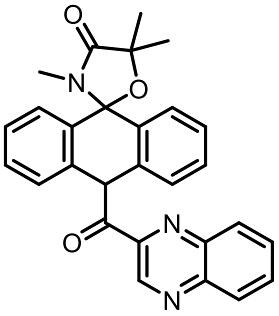

Prepared according to the above procedure to afford **25 (**29.3mg) 65% yield as yellow solid. m.p. = 88.3 – 92.5 ºC.

*NMR and HRMS data for the product **25***:

^{1}H NMR (600 MHz, CDCl₃) δ (ppm): 9.47 (s, 1H), 8.45 (d, *J* = 5.4 Hz, 1H), 8.23 (d, *J* = 6.0 Hz, 1H), 7.98 (d, *J* = 4.2 Hz, 2H), 7.51-7.40 (m, 6H), 7.29 (t, *J* = 6.6 Hz, 2H), 7.20 (s, 1H), 2.65 (s, 3H), 1.59 (s, 6H)

^{13}C NMR (151 MHz, CDCl₃) δ (ppm): 196.5, 176.0, 145.8, 144.8, 143.9, 140.9, 135.5, 135.3, 132.7, 131.0, 130.7, 129.6, 129.2, 129.0, 128.4, 126.6, 91.6, 80.5, 49.0, 27.4, 27.2

HRMS (ESI-TOF) *m/z*: $[M + H]^+$ Calcd for $C_{28}H_{23}N_3O_3H^+$ 450.1812;Found 450.1811.

iv) *Reactions with para-substituted **1c** and **1d***

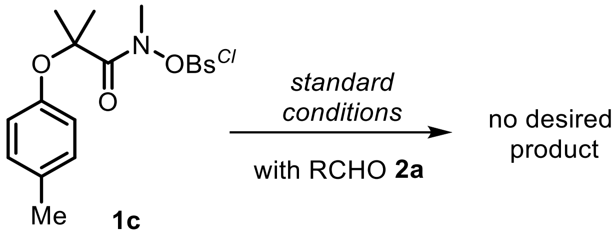

1c

To an oven-dried Schlenck tube was added substrate **1c** (0.10 mmol), NHC **N2** (10 mol %) and K_2CO_3 (0.20 mmol). The Schlenck tube was subjected to three cycles of pressurization/depressurization using dry Ar. Then, under the protection of Ar atmosphere, a solution of aldehyde **2a** (0.25 mmol) in dry mesitylene (1 mL) was added and the reaction mixture was stirred at 60 °C for 12 hours. After that, no desired product was observed.

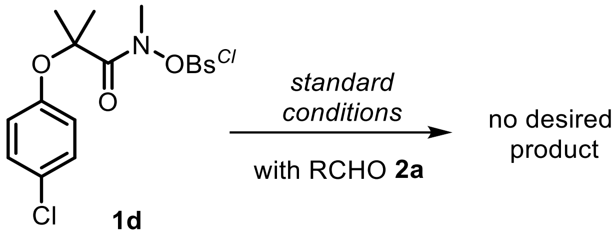

1d

To an oven-dried Schlenck tube was added substrate **1d** (0.10 mmol), NHC **N2** (10 mol %) and K_2CO_3 (0.20 mmol). The Schlenck tube was subjected to three cycles of pressurization/depressurization using dry Ar. Then, under the protection of Ar atmosphere, a solution of aldehyde **2a** (0.25 mmol) in dry mesitylene (1 mL) was added and the reaction mixture was stirred at 60 °C for 12 hours. After that, no desired product was observed.

v) *Proposed mechanism*

On the basis of the foregoing experimental evidence, a plausible mechanism for the present difunctionalization reaction is proposed, as depicted in the following Figure. The catalytic cycle is initiated by the formation of deprotonated Breslow intermediate **I** from the free carbene catalyst, aldehyde **2** and base. A single-electron transfer (SET) from intermediate **I** to arene **1** generates the NHC-bound ketyl radical **II** and the *N*-centered radical **III**. Subsequent intramolecular addition of *N*-centered radical to the *ipso* position of arene produces the radical Meisenheimer intermediate (MI) **IV**. Radical cross-coupling between **II** and **IV** then happens at *para* position to regenerate the NHC catalyst and affords the intermediate **V**. Then, deprotonation of **V** gives an anonic MI **VI**. Finally, the two-electron C–O bond fragmentation delivers the rearomatized product **3**.

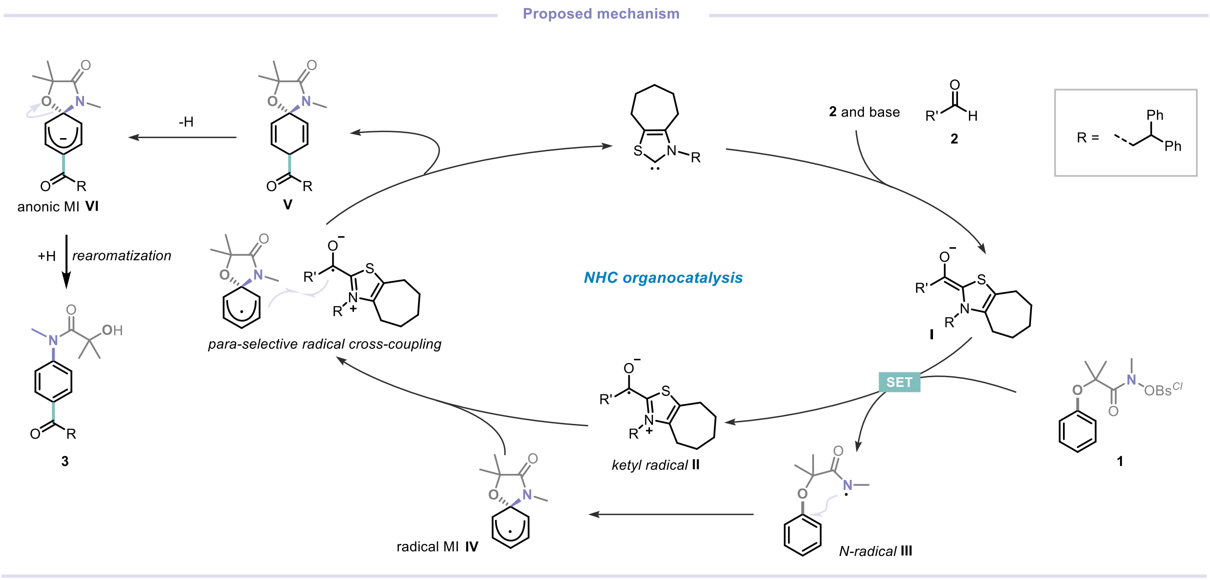

9. Crystal Data and Structure Refinement

i) *Crystal preparation and measurement for* ***3q***

To a tube containing **3q** (30 mg) was added a 10:1 mixture of petroleum ether and dichloromethane (about 5 mL). Tube was sealed up and kept aside for 3 days at room temperature to obtain crystals. The crystals were subjected for single crystal XRD to determine the structure of **3q**. The data were collected by a Bruker APEX-II CCD equipped with an Mo radiation source (Kα = 0.71073 Å) at 300 K. CCDC 2354052 (**3q**) contains the supplementary crystallographic data for this paper.

Crystal Data (at 50% probability level)

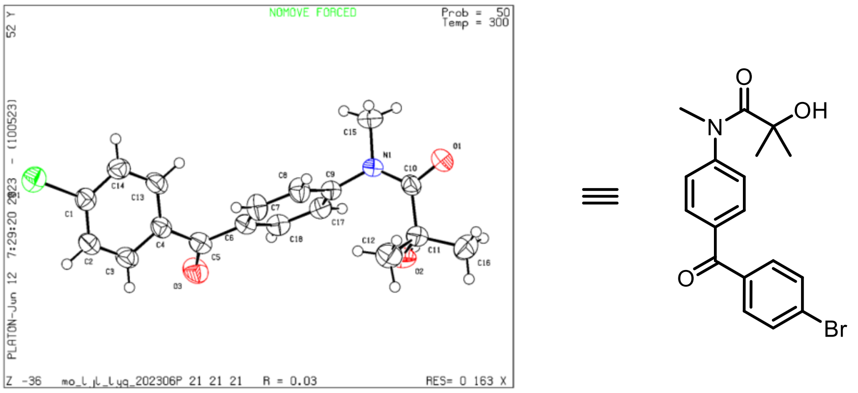

3q

Identification code	**3q**
Empirical formula	$C_{18}H_{18}BrNO_3$
Formula weight	376.24
Temperature/K	300.0
Crystal system	orthorhombic
Space group	$P2_12_12_1$
a/Å	7.4843(4)
b/Å	8.0103(6)
c/Å	27.8470(18)
α/°	90
β/°	90
γ/°	90
Volume/Å^3	1669.47(19)
Z	4
ρcalcg/cm^3	1.497
μ/mm^{-1}	2.476

F(000)	768.0
Crystal size/mm^3	0.26 × 0.22 × 0.16
Radiation	MoKα (λ = 0.71073)
2Θ range for data collection/°	5.292 to 55.042
Index ranges	-9≤h≤9, -10≤k≤ 9, -36≤l≤35
Reflections collected	26424
Independent reflections	3833 [R_{int} = 0.0604, R_{sigma} = 0.0466]
Data/restraints/parameters	3833/0/212
Goodness-of-fit on F^2	1.010
Final R indexes [I>=2σ (I)]	R_1 = 0.0319, wR_2 = 0.0650
Final R indexes [all data]	R_1 = 0.0589, wR_2 = 0.0735
Largest diff. peak/hole / e Å^{-3}	0.21/-0.49

ii) *Crystal preparation and measurement for **8h***

To a tube containing **8h** (50 mg) was added a 1:1 mixture of petroleum ether and dichloromethane (about 10 mL). Tube was sealed up and kept aside for 5 days at room temperature to obtain crystals. The crystals were subjected for single crystal XRD to determine the structure of **8h**. The data were collected by a Bruker APEX-II CCD equipped with a Cu radiation source (Kα = 1.54178 Å) at 293 K. CCDC 2353700 (**8h**) contains the supplementary crystallographic data for this paper.

Crystal Data (at 50% probability level)

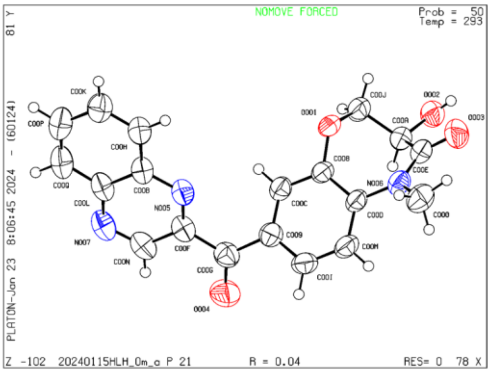

≡

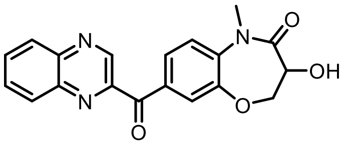

8h

Identification code	**8h**
Chemical formula	$C_{19}H_{15}N_3O_4$
Formula weight	349.34 g/mol
Temperature	293(2) K
Wavelength	1.54178 Å

Crystal system	monoclinic
Space group	P 1 21 1
Unit cell dimensions	a = 9.2208(2) Å α = 90°
	b = 6.6894(2) Å β = 90.9360(10)°
	c = 13.0510(3) Å γ = 90°
Volume	804.90(4) $Å^3$
Z	2
Density (calculated)	1.441 g/cm^3
Absorption coefficient	0.858 mm^{-1}
F(000)	364
Theta range for data collection	3.39 to 68.35°
Index ranges	-11<=h<=11, -8<=k<=7, -15<=l<=15
Reflections collected	16571
Independent reflections	2927 [R(int) = 0.0495]
Coverage of independent reflections	99.6%
Absorption correction	Multi-Scan
Structure solution technique	direct methods
Structure solution program	SHELXT 2014/5 (Sheldrick, 2014)
Refinement method	Full-matrix least-squares on F^2
Refinement program	SHELXL-2016/6 (Sheldrick, 2016)
Function minimized	$\Sigma w(F_o^2 - F_c^2)^2$
Data / restraints / parameters	2927 / 1 / 240
Goodness-of-fit on F^2	1.070
Final R indexes [I>=2σ (I)]	R_1 = 0.0446, wR_2 = 0.1252
Final R indexes [all data]	R_1 = 0.0479, wR_2 = 0.1319
Weighting scheme	$w=1/[\sigma^2(F_o^2)+(0.1000P)^2]$ where $P=(F_o^2+2F_c^2)/3$
Absolute structure parameter	-0.03(13)
Largest diff. peak and hole	0.132 and -0.200 $eÅ^{-3}$
R.M.S. deviation from mean	0.51 $^{-3}$

10. DFT computational calculation studies

i) *Computational methods*

Density functional theory (DFT) calculations were performed with Gaussian 16 suite of programs.[11] All geometry optimizations were operated to locate all of the stationary points, using the dispersion-corrected M06-2X density functional theory method[12,13] with 6-31G(d) basis set[14,15] for all atoms (with the guess=mix keyword as implemented in Gaussian 16 for open shell calculation). The integral equation formalism variant of the polarizable continuum model (IEF-PCM) was used to account for the solvation effects of mesitylene for all calculations, using self-consistent reaction field (SCRF) method.[16-19] In the meantime, the stability of the DFT wave-function of the auxiliary Kohn–Sham determinant was examined.[20] Harmonic vibrational frequency calculations were conducted at the same level of theory used for the geometry optimizations to characterize all stationary points. Herein, minima have zero imaginary frequencies, and transition states (TS) have one imaginary vibrational frequency. Intrinsic reaction coordinate (IRC) was used to identify transition states connecting two relevant minima.[21] To get more accurate energies, single point energies were calculated with the IEF-PCM solvation model in mesitylene solution with the(U)M06-2X functional and a 6-311++G(2d,2p) basis set for all atoms. Unless otherwise noted, the Gibbs free energies of formation (ΔG, kcal/mol) are relative to the initial reactants obtained at the (U)M06-2X/6-311++G(2d,2p)/IEF-PCM(mesitylene)//(U)M06-2X /6-31G(d)/IEF-PCM (mesitylene) level of theory, under experimental temperature of 333.15 K. Mulliken spin distribution and natural population analysis (NPA)[22,23] are obtained at the same level of theory as the geometry optimization. To further investigate the reactivity in the ring-opening process, analysis of the Laplacian bond order (LBO) and the Laplacian of electron density[24] for key intermediates and transition states was performed through Multiwfn software.[25] DFT-optimized structures are illustrated using *CYLView* v1.0 software.[26]

ii) *Computational results*

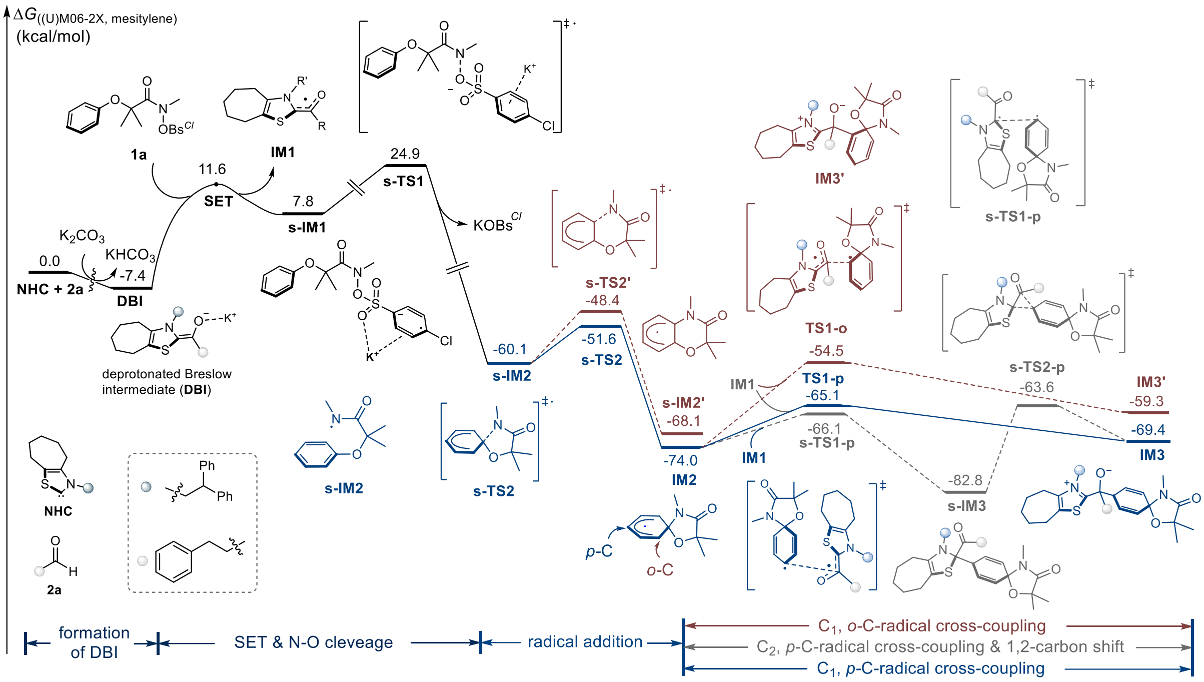

Figure S1. Gibbs free energy profiles for the formation of **IM3**. Gibbs free energies (Δ*G*, kcal/mol) were obtained at the (U)M06-2X/6-311++G(2d,2p)/IEF-PCM(mesitylene)//(U)M06-2X/6-31G(d)/IEF-PCM(mesitylene) level of theory, under experimental temperature of 333.15 K.

As show in Figure S1, a deprotonated Breslow intermediate (**DBI**) is firstly generated from **NHC** catalysis and aldehyde **2a**, and subsequent the single electron transfer (SET) affords an NHC-bound ketyl radical intermediate **IM1** and the radical **s-IM1** derived from the substrate **1a**. Next, the N–O bond cleavage takes place via **s-TS1** (Δ*G* = 24.9 kcal/mol) to form an nitrogen-centred admidyl radical **s-IM2** and the accompanying the release of KOBs^{Cl}. Then, a radical Meisenheimer intermediate **IM2** is generated *via* **s-TS2** with a Gibbs energy barrier of 8.5 kcal/mol. Alternatively, a competitive radical intermediate **s-IM2'** is generated *via* **s-TS2'** with a Gibbs energy barrier of 11.7 kcal/mol. These results indicated that the formation of radical Meisenheimer intermediate is kinetically and thermodynamically favourable.

Starting from **IM2**, the *para*-selective radical cross-coupling between **IM1** and **IM2** occurs through transition state **TS1-p** significantly lower than that of the *ortho*-coupling pathway via **TS1-o** ($\Delta G^{\ddagger}$, 8.9 *vs.* 19.5 kcal/mol). This energy difference rationalizes the observed site-selectivity in the reaction. In addition, the *para*-selective radical cross-coupling between the C_2 site of **IM1** and **IM2** occurs through transition state **s-TS1-p** with a Gibbs

energy barrier of 7.9 kcal/mol to form the **s-IM3**. And then, the 1,2-aryl migration occurs via **s-TS2-p** ($\Delta G^{\ddagger}$ = 19.2 kcal/mol) to form the intermediate **IM3**. Thereby, a potential side pathway (grey part) of 1,2-aryl migration appears to be energetically less favorable.

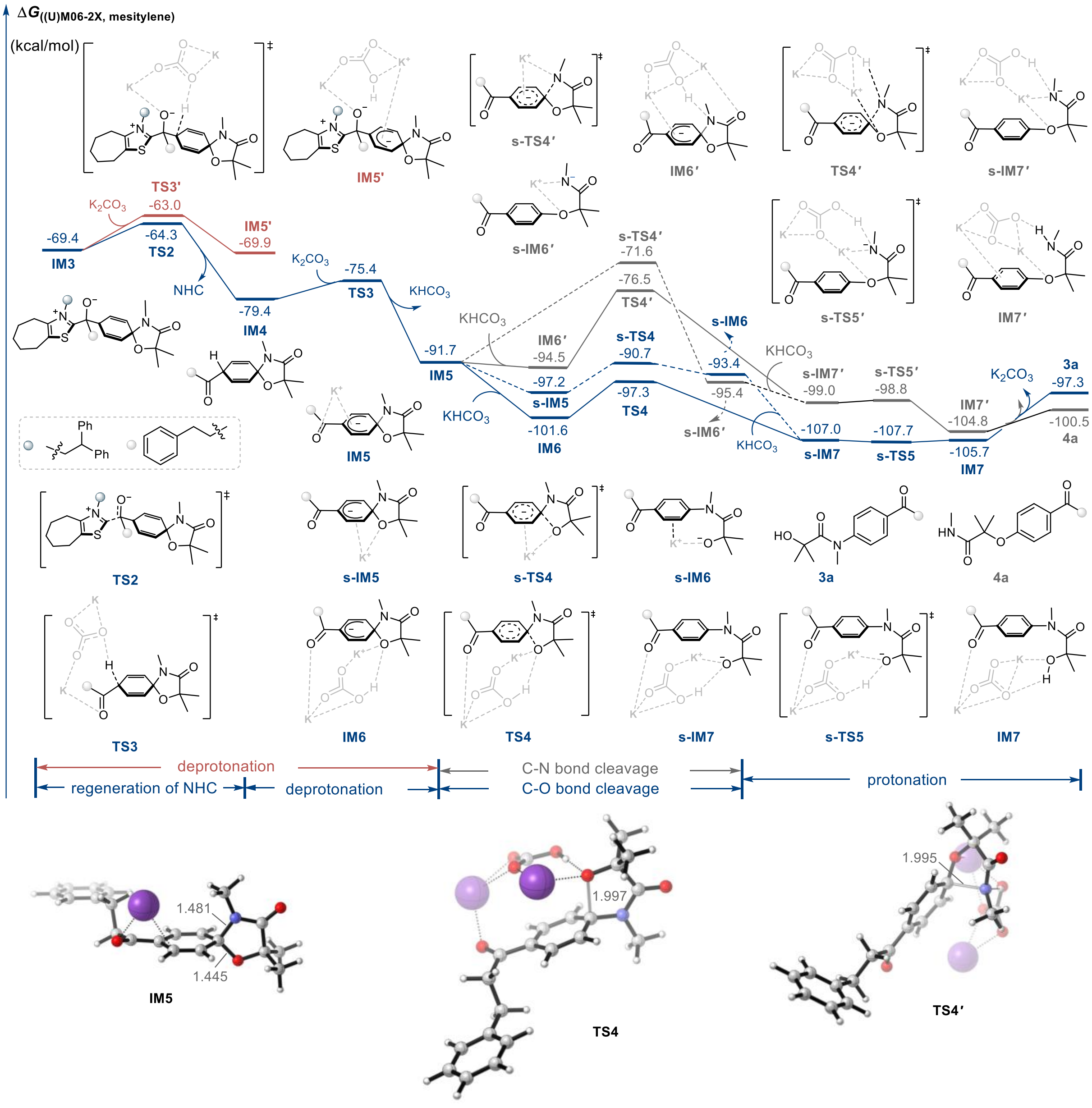

Figure S2. Gibbs free energy profiles for the formation of **3a** from **IM3**. DFT-optimized structures for the key intermediate **IM5** and transition states **TS4**, **TS4'** are shown. Selected distances shown in 3D-optimized structures are in Angstrom (Å). Gibbs free energies (ΔG, kcal/mol) were obtained at the (U)M06-2X/6-311++G(2d,2p)/IEF-PCM(mesitylene)//(U)M06-2X/6-31G(d)/IEF-PCM(mesitylene) level of theory, under experimental temperature of 333.15 K.

Next, comparison of Gibbs free energy profiles for the C–O and C–N bond breakage is shown in Figure S2. As shown in Figure S2, starting from **IM3**, the release of the NHC catalyst yields intermediate **IM4** (via **TS2**, $\Delta G^{\ddagger}$ = 5.1 kcal/mol). Subsequently, **IM4** undergoes deprotonation via **TS3** ($\Delta G^{\ddagger}$ = 4.0 kcal/mol) to form the anionic Meisenheimer intermediate **IM5** and release the $KHCO_3$ molecule. Next, the $KHCO_3$-assisted C–O bond breakage takes place via **TS4** to generate $KHCO_3$–bound intermediate **IM7**. Finally, the release of $KHCO_3$ yields the final rearomatized product **3a**. The C–N bond breakage as a competitor has the significantly higher energy barrier of 20.8 kcal/mol calculated for the corresponding transition state **TS4'**, affording the product **4a**. Thus, the C–O bond scission pathway (**TS4**, ΔG = –97.3 kcal/mol) being kinetically favored over C–N bond cleavage (**TS4'**, ΔG = –76.5 kcal/mol). A competing C–O and C–N bond-breakage pathways involving inexistent $KHCO_3$ were also considered. The calculations results indicate that the pathways of inexistent $KHCO_3$ require significantly higher energies.

A competing pathway involving direct deprotonation of **IM3** prior to catalyst dissociation was also considered (See Figure S2). Starting from **IM3**, the direct deprotonation process happens via transition state **TS3'** with the higher energy barrier $\Delta G^{\ddagger}$ of 6.4 kcal/mol than that of **TS2** located at the release of the NHC catalys ($\Delta G^{\ddagger}$ = 5.1 kcal/mol). Thus, the reaction pathways of direct deprotonation of **IM3** is considered less favorable.

New transition state structures (4 for C1-selectivity and 4 for C2-selectivity) were identified that exhibit proper spin density characteristics (Figure 3A). As shown in the energy profile below (from **IM2** to **IM3**), the highest energy barriers for the two pathways remain very close (-65.1 vs. -63.6 kcal/mol). Therefore, these results supports that two pathways (direct ketyl radical coupling and C2-coupling/1,2-migration) are competitive under the reaction conditions.

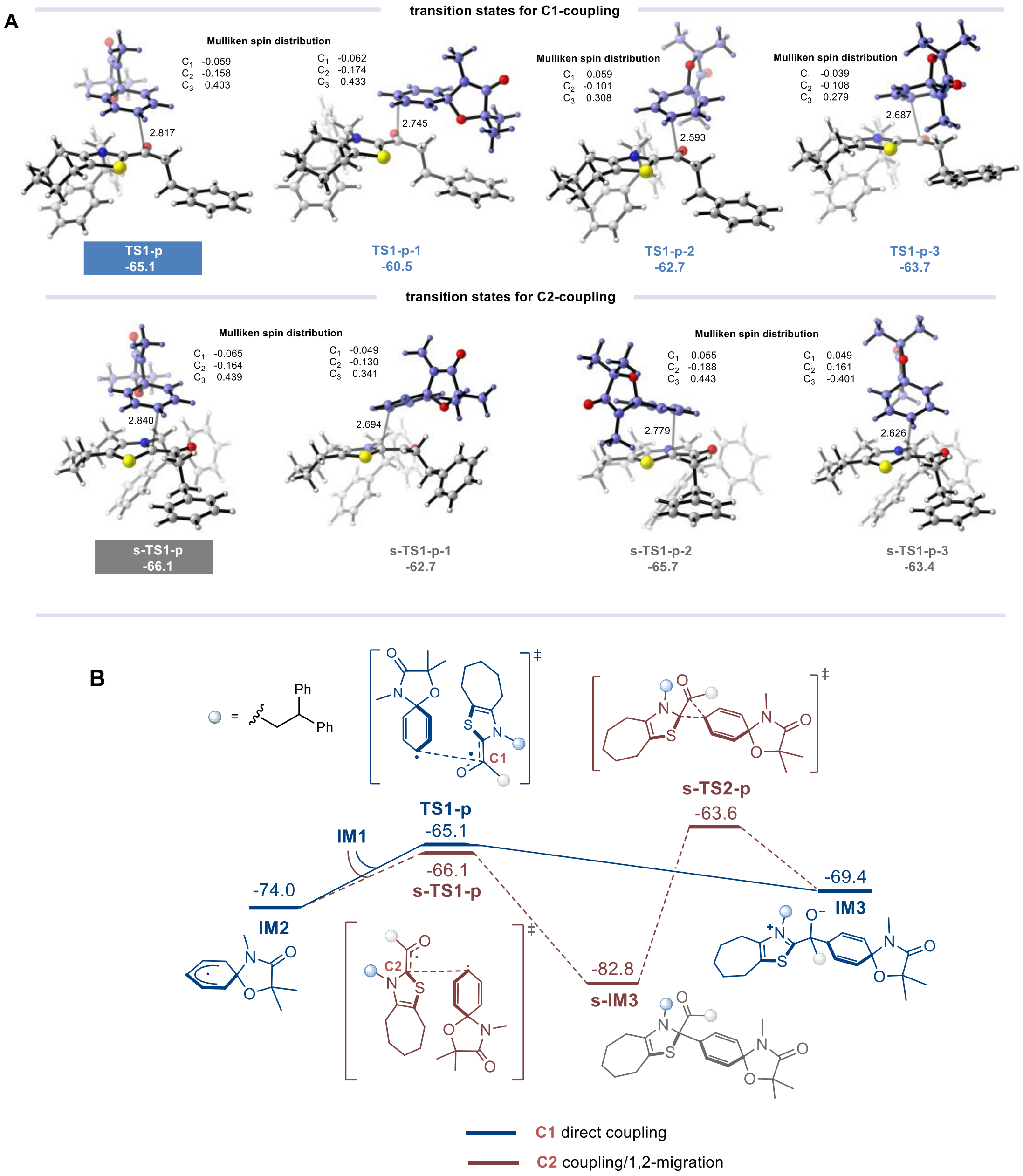

Figure S3. A Transition states for **TS1-p/s-TS1-p** at the (U)M06-2X/6-311++G(2d,2p)/IEF-PCM(mesitylene)//(U)M06-2X/6-31G(d)/IEF-PCM(mesitylene) level of theory, under experimental temperature of 333.15 K. **B** Competitive pathways of radical couplings.

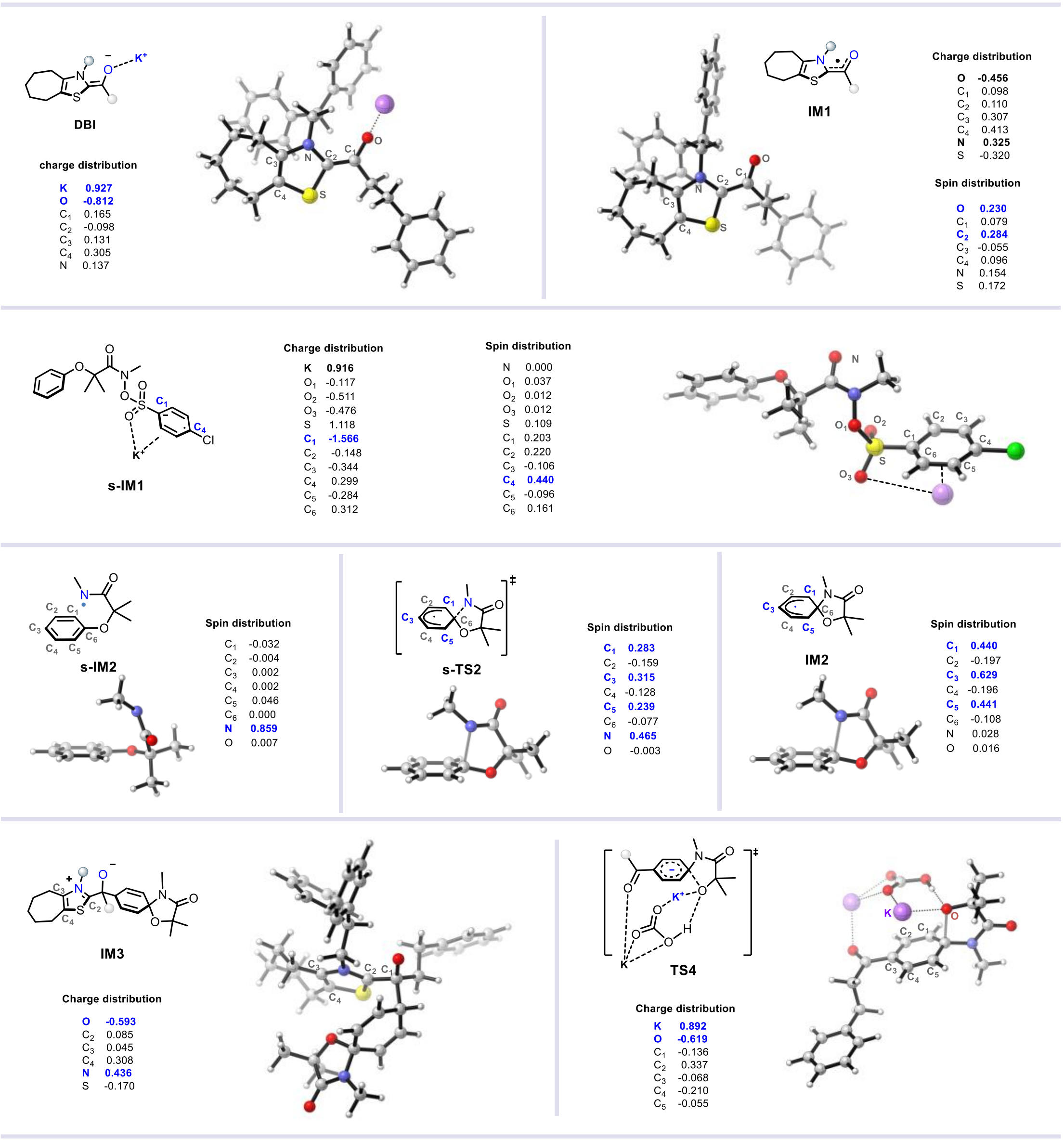

Figure S4. Mulliken charge and spin distributions for the typical species at the (U)M06-2X/6-311++G(2d,2p)/IEF-PCM(mesitylene)//(U)M06-2X/6-31G(d)/IEF-PCM(mesitylene) level of theory, under experimental temperature of 333.15 K.

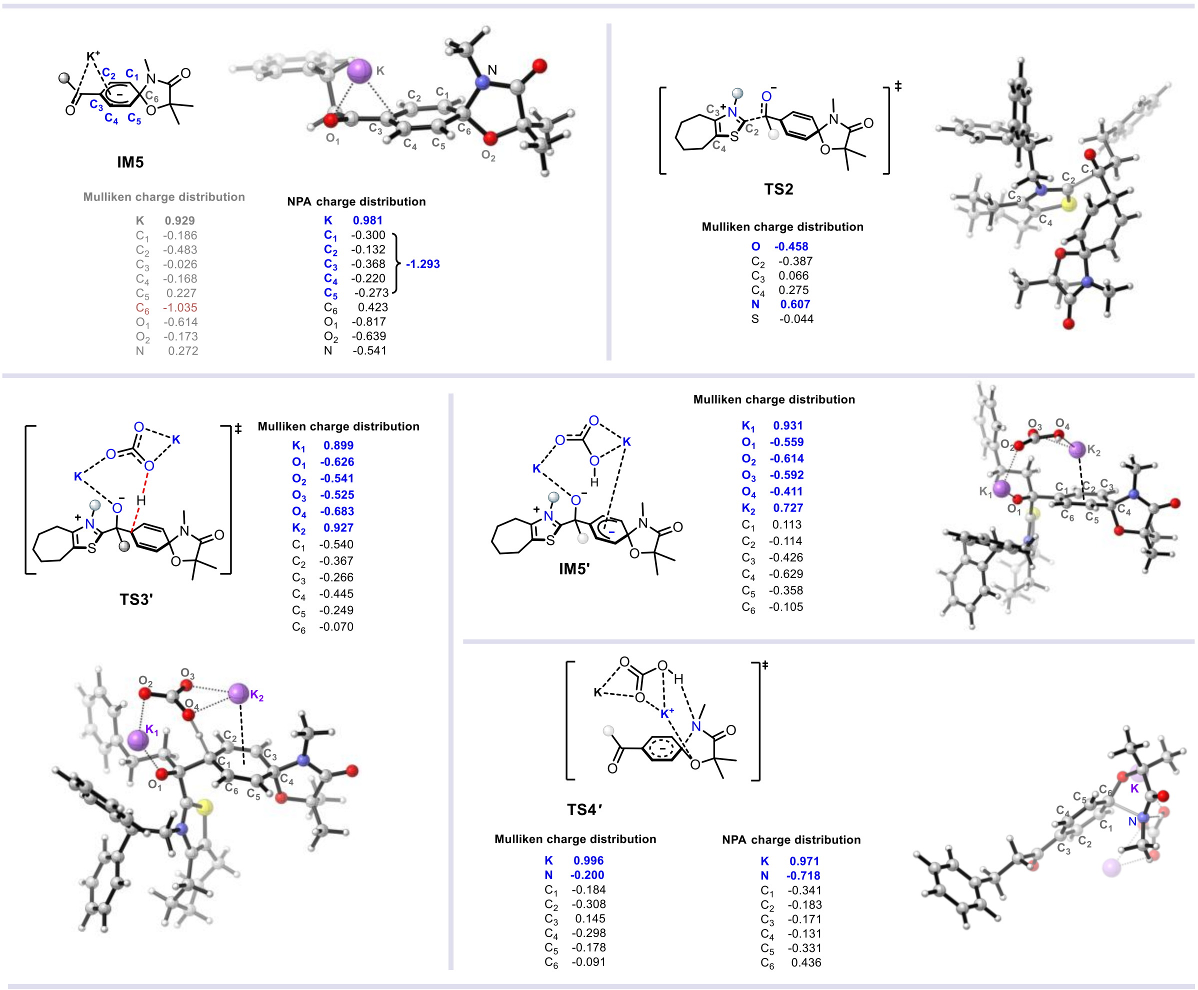

Figure S5. Mulliken or NPA charge distributions for the typical species at the (U)M06-2X/6-311++G(2d,2p)/IEF-PCM(mesitylene)//(U)M06-2X/6-31G(d)/IEF-PCM(mesitylene) level of theory, under experimental temperature of 333.15 K.

To further investigate the origin of the observed selectivity, we recalculated the Laplacian Bond Order (LBO) for the C–O and C–N bonds in the updated **TS4** (Figure S5). While the absolute values have slightly increased compared to our previous results (from 0.212 vs. 0.486 to 0.260 vs. 0.521), the relative trend remains consistent. These results suggest that the C–O bond is significantly weaker than the C–N bond, well supporting preferential C–O cleavage.

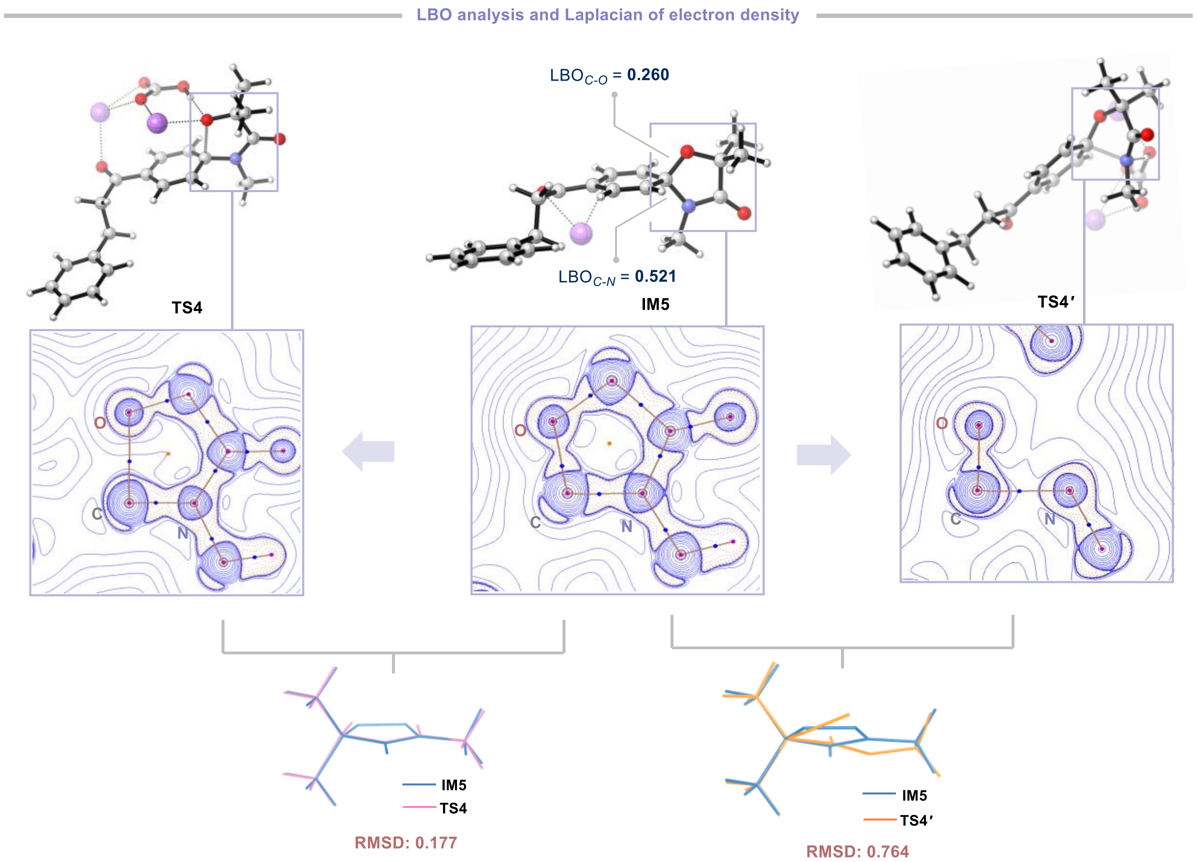

Figure S6. Analysis of Laplacian bond order, Laplacian of electron density and root mean square displacement/deviation (RMSD) for **IM5**, **TS4** and **TS4′**.

In addition, the analysis of Laplacian of electron density reveals that the spiro five-membered ring in the revised **TS4** also adopts a nearly planar geometry, similar to the precursor intermediate **IM5**. In contrast, **TS4′**, the transition state leading to the C–N cleavage, exhibits pronounced geometric distortion relative to **IM5**. This structural difference is quantified using root mean square displacement/deviation (RMSD) of atomic positions between **IM5** and each transition state. The RMSD value for **IM5**/**TS4′** is substantially higher than that for **IM5**/**TS4** (0.764 *vs.* 0.177), confirming greater geometric perturbation along the C–N cleavage pathway. Therefore, we propose that this ring distortion in **TS4′**, rather than differential hyperconjugation, is a possible factor disfavoring the C–N cleavage pathway.

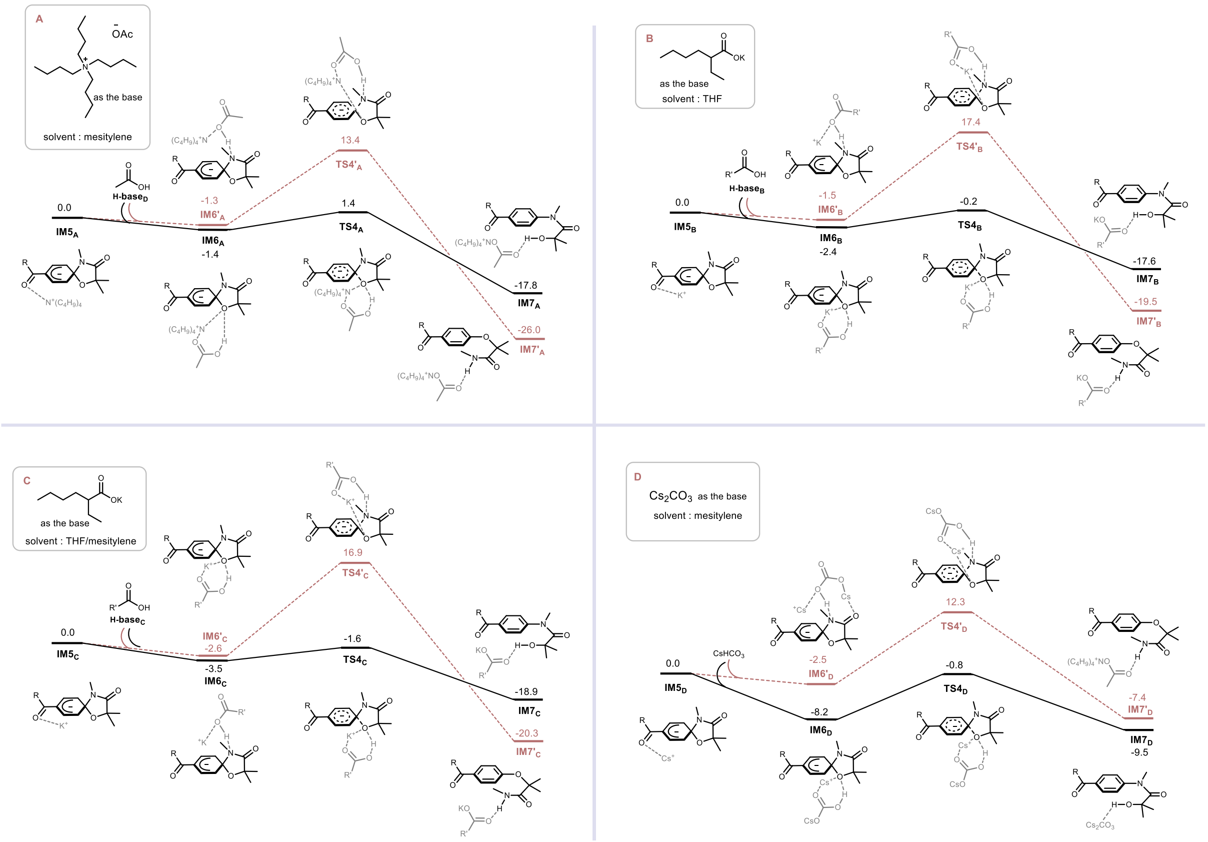

Figure S7. Base assessment in different solvent. **A** *n*Bu_4OAc in mesitylene. **B** potassium 2-ethylhexanoate in THF. **C** potassium 2-ethylhexanoate in THF/mesitylene 1:1. **D** Cs_2CO_3 in mesitylene.

A homogeneous system using *n*Bu_4OAc as the base was evaluated in our computational model. As shown in the figure below, the transition state for C–N bond cleavage is higher in energy by 13.1 kcal/mol compared to that for C–O bond cleavage, in excellent agreement with the experimental observations. Furthermore, additional DFT calculations to examine different base/solvent combinations in heterogeneous systems was performed, in order to evaluate the generality of the computational approach. The calculated energy profiles for the C–N versus C–O bond cleavage pathways show consistent trends across these different conditions, with energy barrier differences remaining greater than 10 kcal/mol in all cases.

iii) *Data of energies*

Table S8. Zero-point energies (*ZPE*, hartree), thermal correction to Gibbs free energy (G_0, hartree) , single-point energies (SP-E, hartree), total energies (E_c, hartree) corrected by the addition of *ZPE* and SP-E, sum of electronic and thermal free energies (G_c, hartree) with the addition of SP-E and thermal corrections, and relative energies (ΔE, kcal/mol) and relative Gibbs free energies (ΔG, kcal/mol) of various species for the formation of **IM3** in the presence of K_2CO_3 at the (U)M06-2X/6-311++G(2d,2p)/IEF-PCM(mesitylene)//(U)M06-2X/6-31G(d)/IEF-PCM(mesitylene) level of theory, under experimental temperature of 333.15 K. IF represents imaginary frequencies (cm^{-1}).

Species	*ZPE*	G_0	SP-E	E_c (ZPE + SP-E)	ΔE	G_c (G_0 + SP-E)	ΔG	IF
**NHC**	0.40231	0.34362	-1304.97887	-1304.57655		-1304.63525		
**1a**	0.33387	0.26777	-1947.71070	-1947.37684		-1947.44294		
**2a**	0.16828	0.12578	-424.13942	-423.97115		-424.01365		
K_2CO_3	0.01716	-0.02094	-1463.79932	-1463.78216		-1463.82025		
**NHC** + **1a** + **2a** + K_2CO_3	0.92162	0.71622	-5140.62831	-5139.70670	0.0	-5139.91209	0.0	
**DBI**	0.56276	0.48424	-2328.53841	-2327.97565		-2328.05418		
$KHCO_3$	0.02863	-0.00541	-864.42133	-864.39270		-864.42673		
**DBI** + **1a** + $KHCO_3$	0.92526	0.74659	-5140.67045	-5139.74519	-24.2	-5139.92385	-7.4	
**s-IM1**	0.33175	0.26121	-2547.66195	-2547.33020		-2547.40074		
**IM1**	0.56325	0.48645	-1728.55868	-1727.99542		-1728.07223		
**s-IM1** + **IM1** + $KHCO_3$	0.92363	0.74225	-5140.64195	-5139.71832	-7.3	-5139.89971	7.8	
**s-TS1**	0.32913	0.26057	-2547.63402	-2547.30489		-2547.37345		3254.71*i*
**s-TS1** + **IM1** + $KHCO_3$	0.92102	0.74161	-5140.61403	-5139.69301	8.6	-5139.87241	24.9	
**s-IM2**	0.23320	0.18483	-632.66830	-632.43509		-632.48347		

KOBs^{Cl}	0.09641	0.04960	-1915.07497	-1914.97856		-1915.02537		
**s-IM2** + KOBs^{Cl} + **IM1** + KHCO$_3$	0.92150	0.71546	-5140.72327	-5139.80178	-59.7	-5140.00781	-60.1	
**s-TS2**	0.23228	0.18620	-632.65617	-632.42389		-632.46997		463.44*i*
**s-TS2** + KOBs^{Cl} + **IM1** + KHCO$_3$	0.92057	0.71684	-5140.71114	-5139.79057	-52.6	-5139.99431	-51.6	
**IM2**	0.23410	0.18682	-632.69247	-632.45838		-632.50565		
**IM2** + KOBs^{Cl} + **IM1** + KHCO$_3$	0.92239	0.71746	-5140.74745	-5139.82506	-74.3	-5140.02999	-74.0	
**s-TS2’**	0.23339	0.18766	-632.65256	-632.41916		-632.46490		385.73*i*
**s-TS2’** + KOBs^{Cl} + **IM1** + KHCO$_3$	0.92169	0.71829	-5140.70753	-5139.78584	-49.7	-5139.98924	-48.4	
**s-IM2’**	0.23557	0.18958	-632.68578	-632.45021		-632.49620		
**s-IM2’** + KOBs^{Cl} + **IM1** + KHCO$_3$	0.92386	0.72021	-5140.74076	-5139.81690	-69.1	-5140.02054	-68.1	
**TS1-p**	0.79832	0.69935	-2361.26314	-2360.46482		-2360.56379		75.13*i*
**TS1-p** + KOBs^{Cl} + KHCO$_3$	0.92336	0.74354	-5140.75944	-5139.83608	-81.2	-5140.01590	-65.1	
**TS1-p-1**	0.79812	0.70260	-2361.25900	-2360.46088		-2360.55640		95.17*i*
**TS1-p-1** + KOBs^{Cl} + KHCO$_3$	0.92316	0.74679	-5140.75530	-5139.83213	-78.7	-5140.00851	-60.5	
**TS1-p-2**	0.79890	0.70142	-2361.26128	-2360.46238		-2360.55987		210.39*i*
**TS1-p-2** + KOBs^{Cl} + KHCO$_3$	0.92394	0.74561	-5140.75758	-5139.83364	-79.7	-5140.01198	-62.7	
**TS1-p-3**	0.79902	0.70169	-2361.26311	-2360.46410		-2360.56142		162.65*i*
**TS1-p-3** + KOBs^{Cl} + KHCO$_3$	0.92406	0.74588	-5140.75941	-5139.83535	-80.7	-5140.01353	-63.7	
**s-TS1-p**	0.79791	0.70006	-2361.26545	-2360.46754		-2360.56539		129.69*i*
**s-TS1-p** + KOBs^{Cl} + KHCO$_3$	0.92295	0.74425	-5140.76174	-5139.83880	-82.9	-5140.01750	-66.1	
**s-TS1-p-1**	0.79861	0.70175	-2361.26161	-2360.46300		-2360.55986		128.76*i*
**s-TS1-p-1** + KOBs^{Cl} + KHCO$_3$	0.92365	0.74594	-5140.75791	-5139.83426	-80.0	-5140.01197	-62.7	

**s-TS1-p-2**	0.79799	0.69849	-2361.26323	-2360.46524		-2360.56474		74.12*i*
**s-TS1-p-2** + KOBs^{Cl} + $KHCO_3$	0.92303	0.74268	-5140.75953	-5139.83650	-81.4	-5140.01685	-65.7	
**s-TS1-p-3**	0.79832	0.69875	-2361.25969	-2360.46137		-2360.56094		232.93*i*
**s-TS1-p-3** + KOBs^{Cl} + $KHCO_3$	0.92337	0.74294	-5140.75599	-5139.83263	-79.0	-5140.01305	-63.4	
**s-IM3**	0.80163	0.70321	-2361.29508	-2360.49345		-2360.59187		
**s-IM3** + KOBs^{Cl} + $KHCO_3$	0.92667	0.74739	-5140.79138	-5139.86471	-99.2	-5140.04398	-82.8	
**s-TS2-p**	0.80052	0.70668	-2361.26802	-2360.46749		-2360.56133		
**s-TS2-p** + KOBs^{Cl} + $KHCO_3$	0.92556	0.75087	-5140.76432	-5139.83875	-82.9	-5140.01344	-63.6	
**IM3**	0.80184	0.70717	-2361.27778	-2360.47594		-2360.57061		
**IM3** + KOBs^{Cl} + $KHCO_3$	0.92688	0.75136	-5140.77408	-5139.84720	-88.2	-5140.02272	-69.4	
**TS1-o**	0.80055	0.70717	-2361.25393	-2360.45338		-2360.54675		139.18*i*
**TS1-o** + KOBs^{Cl} + $KHCO_3$	0.92559	0.75136	-5140.75022	-5139.82464	-74.0	-5139.99886	-54.5	
**IM3'**	0.80218	0.70848	-2361.26291	-2360.46073		-2360.55443		
**IM3'** + KOBs^{Cl} + $KHCO_3$	0.92722	0.75267	-5140.75921	-5139.83199	-78.6	-5140.00654	-59.3	

Table S9. Zero-point energies (*ZPE*, hartree), thermal correction to Gibbs free energy (G_0, hartree) , single-point energies (SP-E, hartree), total energies (E_c, hartree) corrected by the addition of *ZPE* and SP-E, sum of electronic and thermal free energies (G_c, hartree) with the addition of SP-E and thermal corrections, and relative energies (ΔE, kcal/mol) and relative Gibbs free energies (ΔG, kcal/mol) of various species for the formation of products **3a** and **4a** starting from **IM3** in the presence of K_2CO_3 at the (U)M06-2X/6-311++G(2d,2p)/IEF-PCM(mesitylene)//(U)M06-2X/6-31G(d)/IEF-PCM(mesitylene) level of theory, under experimental temperature of 333.15 K. IF represents imaginary frequencies (cm^{-1}).

Species	*ZPE*	G_0	SP-E	E_c (ZPE + SP-E)	ΔE	G_c (G_0 + SP-E)	ΔG	IF
**IM3**	0.80184	0.70717	-2361.27778	-2360.47594		-2360.57061		
K_2CO_3	0.01716	-0.02094	-1463.79932	-1463.78216		-1463.82025		
**IM3** + K_2CO_3	0.81900	0.68623	-3825.07710	-3824.25810	0.0	-3824.39087	0.0	
**TS2**	0.80061	0.70669	-2361.26919	-2360.46858		-2360.56250		148.39*i*
**TS2** + K_2CO_3	0.81777	0.68575	-3825.06850	-3824.25074	4.6	-3824.38275	5.1	
**IM4**	0.39659	0.33138	-1056.28262	-1055.88604		-1055.95124		
**NHC**	0.40231	0.34362	-1304.97887	-1304.57655		-1304.63525		
**IM4** + **NHC** + K_2CO_3	0.81606	0.65406	-3825.06080	-3824.24475	8.4	-3824.40674	-10.0	
**TS3**	0.41091	0.33349	-2520.09867	-2519.68776		-2519.76519		404.26*i*
**TS3** + **NHC**	0.81322	0.67710	-3825.07754	-3824.26431	-3.9	-3824.40044	-6.0	
**IM5**	0.38524	0.31765	-1655.68207	-1655.29683		-1655.36442		
$KHCO_3$	0.02863	-0.00541	-864.42133	-864.39270		-864.42673		
**IM5** + **NHC** + $KHCO_3$	0.81618	0.65586	-3825.08226	-3824.26608	-5.0	-3824.42640	-22.3	
**IM6**	0.41483	0.33676	-2520.14374	-2519.72891		-2519.80698		
**IM6** + **NHC**	0.81715	0.68037	-3825.12261	-3824.30546	-29.7	-3824.44223	-32.2	

**TS4**	0.41433	0.33773	-2520.13783	-2519.72350		-2519.80010		229.31*i*
**TS4** + **NHC**	0.81664	0.68135	-3825.11670	-3824.30006	-26.3	-3824.43535	-27.9	
**IM7**	0.41534	0.34042	-2520.15384	-2519.73850		-2519.81342		
**IM7** + **NHC**	0.81765	0.68404	-3825.13271	-3824.31506	-35.7	-3824.44867	-36.3	
**s-IM5**	0.38440	0.31607	-1655.68922	-1655.30482		-1655.37315		
**s-IM5** + **NHC** + $KHCO_3$	0.81534	0.65428	-3825.08941	-3824.27407	-10.0	-3824.43514	-27.8	
**s-TS4**	0.38302	0.31517	-1655.67796	-1655.29493		-1655.36279		157.91*i*
**s-TS4** + **NHC** + $KHCO_3$	0.81396	0.65338	-3825.07815	-3824.26419	-3.8	-3824.42477	-21.3	
**s-IM6**	0.38374	0.31509	-1655.68226	-1655.29852		-1655.36717		
**s-IM6** + **NHC** + $KHCO_3$	0.81468	0.65330	-3825.08245	-3824.26777	-6.1	-3824.42915	-24.0	
**s-IM7**	0.41315	0.33700	-2520.15258	-2519.73944		-2519.81558		
**s-IM7** + **NHC**	0.81546	0.68062	-3825.13145	-3824.31599	-36.3	-3824.45083	-37.6	
**s-TS5**	0.41058	0.33573	-2520.15245	-2519.74187		-2519.81672		649.58*i*
**s-TS5** + **NHC**	0.81289	0.67934	-3825.13132	-3824.31842	-37.9	-3824.45197	-38.3	
**3a**	0.39652	0.33091	-1056.31078	-1055.91426		-1055.97987		
**NHC**	0.40231	0.34362	-1304.97887	-1304.57655		-1304.63525		
**3a** + **NHC** + K_2CO_3	0.81599	0.65359	-3825.08896	-3824.27297	-9.3	-3824.43537	-27.9	
**IM6’**	0.41449	0.33657	-2520.13221	-2519.71772		-2519.79564		
**IM6’** + **NHC**	0.81680	0.68018	-3825.11107	-3824.29428	-22.7	-3824.43089	-25.1	
**TS4’**	0.41285	0.33544	-2520.10232	-2519.68948		-2519.76688		305.31*i*
**TS4’** + **NHC**	0.81516	0.67906	-3825.08119	-3824.26603	-5.0	-3824.40213	-7.1	
**IM7’**	0.41546	0.33861	-2520.15059	-2519.73513		-2519.81199		

**IM7’** + **NHC**	0.81777	0.68222	-3825.12946	-3824.31168	-33.6	-3824.44724	-35.4	
**s-TS4’**	0.38274	0.31621	-1655.64863	-1655.26590		-1655.33243		300.16*i*
**s-TS4’** + **NHC + $KHCO_3$**	0.81368	0.65442	-3825.04883	-3824.23515	14.4	-3824.39441	-2.2	
**s-IM6’**	0.38467	0.31703	-1655.68732	-1655.30265		-1655.37030		
**s-IM6’** + **NHC + $KHCO_3$**	0.81562	0.65524	-3825.08752	-3824.27190	-8.7	-3824.43228	-26.0	
**s-IM7’**	0.41490	0.33504	-2520.13779	-2519.72289		-2519.80275		
**s-IM7’** + **NHC**	0.81721	0.67865	-3825.11665	-3824.29944	-25.9	-3824.43800	-29.6	
**s-TS5’**	0.41021	0.33048	-2520.13297	-2519.72277		-2519.80249		994.09*i*
**s-TS5’** + **NHC**	0.81252	0.67409	-3825.11184	-3824.29932	-25.9	-3824.43774	-29.4	
**4a**	0.39680	0.32953	-1056.31449	-1055.91769		-1055.98496		
**NHC**	0.40231	0.34362	-1304.97887	-1304.57655		-1304.63525		
**4a** + **NHC** + K_2CO_3	0.81627	0.65221	-3825.09267	-3824.27640	-11.5	-3824.44046	-31.1	
**TS3’**	0.81542	0.70850	-3825.08909	-3824.27367	-9.8	-3824.38060	6.4	1191.55*i*
**IM5’**	0.82917	0.72497	-3825.11658	-3824.28741	-18.4	-3824.39161	-0.5	

Table S10. Zero-point energies (*ZPE*, hartree), thermal correction to Gibbs free energy (G_0, hartree) , single-point energies (SP-E, hartree), total energies (E_c, hartree) corrected by the addition of *ZPE* and SP-E, sum of electronic and thermal free energies (G_c, hartree) with the addition of SP-E and thermal corrections, and relative energies (ΔE, kcal/mol) and relative Gibbs free energies (ΔG, kcal/mol) of various species from **IM5** to **IM7** in the presence of *n*Bu_4OAc base at the (U)M06-2X/6-311++G(2d,2p)/IEF-PCM(mesitylene)//(U)M06-2X/6-31G(d)/IEF-PCM(mesitylene) level of theory, under experimental temperature of 333.15 K. IF represents imaginary frequencies (cm^{-1}).

Species	*ZPE*	G_0	SP-E	E_c (ZPE + SP-E)	ΔE	G_c (G_0 + SP-E)	ΔG	IF
**IM5$_A$**	0.956513	0.798109	-1741.676772	-1740.720259		-1740.87866		
**H-base$_A$**	0.06909	0.0321	-229.069682	-229.000592		-229.03758		
**IM5$_A$ + H-base$_A$**	1.025603	0.830209	-1970.746454	-1969.720851	0.0	-1969.91625	0.0	
**IM6$_A$**	1.027792	0.857496	-1970.775927	-1969.748135	-17.1	-1969.91843	-1.4	
**TS4$_A$**	1.02482	0.855798	-1970.769855	-1969.745035	-15.2	-1969.91406	1.4	313.87*i*
**IM7$_A$**	1.028295	0.860034	-1970.804612	-1969.776317	-34.8	-1969.94458	-17.8	
**IM6'$_A$**	1.028102	0.861437	-1970.779771	-1969.751669	-19.3	-1969.91833	-1.3	
**TS4'$_A$**	1.024729	0.852805	-1970.747638	-1969.722909	-1.29	-1969.89483	13.4	307.59*i*
**IM7'$_A$**	1.02853	0.851622	-1970.809345	-1969.780815	-37.6	-1969.95772	-26.0	

Table S11. Zero-point energies (*ZPE*, hartree), thermal correction to Gibbs free energy (G_0, hartree) , single-point energies (SP-E, hartree), total energies (E_c, hartree) corrected by the addition of *ZPE* and SP-E, sum of electronic and thermal free energies (G_c, hartree) with the addition of SP-E and thermal corrections, and relative energies (ΔE, kcal/mol) and relative Gibbs free energies (ΔG, kcal/mol) of various species from **IM5** to **IM7** in the presence of potassium 2-ethylhexanoate base at the (U)M06-2X/6-311++G(2d,2p)/IEF-PCM(THF)//(U)M06-2X/6-31G(d)/IEF-PCM(THF) level of theory, under experimental temperature of 333.15 K. IF represents imaginary frequencies (cm^{-1}).

Species	*ZPE*	G_0	SP-E	E_c (ZPE + SP-E)	ΔE	G_c (G_0 + SP-E)	ΔG	IF
**IM5$_B$**	0.415715	0.317177	-1655.70153	-1655.285815		-1655.38435		
**H-base$_B$**	0.251882	0.189922	-464.906765	-464.654883		-464.71684		
**IM5$_B$ + H-base$_B$**	0.667597	0.507099	-2120.608295	-2119.940698	0.0	-2120.10120	0.0	
**IM6$_B$**	0.669626	0.530037	-2120.63508	-2119.965454	-15.5	-2120.10504	-2.4	
**TS4$_B$**	0.667431	0.52933	-2120.630823	-2119.963392	-14.2	-2120.10149	-0.2	293.34*i*
**IM7$_B$**	0.670109	0.536934	-2120.666183	-2119.996074	-34.7	-2120.12925	-17.6	
**IM6'$_B$**	0.669641	0.532502	-2120.636125	-2119.966484	-16.2	-2120.10362	-1.5	
**TS4'$_B$**	0.666336	0.531712	-2120.60514	-2119.938804	1.2	-2120.07343	17.4	376.48*i*
**IM7'$_B$**	0.67047	0.535926	-2120.668238	-2119.997768	-35.8	-2120.13231	-19.5	

Table S12. Zero-point energies (*ZPE*, hartree), thermal correction to Gibbs free energy (G_0, hartree) , single-point energies (SP-E, hartree), total energies (E_c, hartree) corrected by the addition of *ZPE* and SP-E, sum of electronic and thermal free energies (G_c, hartree) with the addition of SP-E and thermal corrections, and relative energies (ΔE, kcal/mol) and relative Gibbs free energies (ΔG, kcal/mol) of various species from **IM5** to **IM7** in the presence of potassium 2-ethylhexanoate base at the (U)M06-2X/6-311++G(2d,2p)/IEF-PCM(THF/mesitylene)//(U)M06-2X/6-31G(d)/IEF-PCM(THF/mesitylene) level of theory, under experimental temperature of 333.15 K. IF represents imaginary frequencies (cm^{-1}).

Species	*ZPE*	G_0	SP-E	E_c (ZPE + SP-E)	ΔE	G_c (G_0 + SP-E)	ΔG	IF
**IM5**$_C$	0.415767	0.317342	-1655.696163	-1655.280396		-1655.37882		
**H-base**$_C$	0.251918	0.189476	-464.905534	-464.653616		-464.71606		
**IM5**$_C$ + **H-base**$_C$	0.667685	0.506818	-2120.601697	-2119.934012	0.0	-2120.09488	0.0	
**IM6**$_C$	0.669662	0.530883	-2120.631299	-2119.961637	-17.3	-2120.10042	-3.5	
**TS4**$_C$	0.667598	0.529859	-2120.627228	-2119.95963	-16.1	-2120.09737	-1.6	286.67*i*
**IM7**$_C$	0.670249	0.537466	-2120.662521	-2119.992272	-36.6	-2120.12506	-18.9	
**IM6'**$_C$	0.669771	0.533369	-2120.63233	-2119.962559	-17.9	-2120.09896	-2.6	
**TS4'**$_C$	0.666417	0.532518	-2120.600499	-2119.934082	0.0	-2120.06798	16.9	373.40*i*
**IM7'**$_C$	0.670631	0.53656	-2120.663861	-2119.99323	-37.2	-2120.12730	-20.3	

Table S13. Zero-point energies (*ZPE*, hartree), thermal correction to Gibbs free energy (G_0, hartree) , single-point energies (SP-E, hartree), total energies (E_c, hartree) corrected by the addition of *ZPE* and SP-E, sum of electronic and thermal free energies (G_c, hartree) with the addition of SP-E and thermal corrections, and relative energies (ΔE, kcal/mol) and relative Gibbs free energies (ΔG, kcal/mol) of various species from **IM5** to **IM7** in the presence of Cs_2CO_3 base at the (U)M06-2X/6-311++G(2d,2p)/IEF-PCM(mesitylene)//(U)M06-2X/6-31G(d)/IEF-PCM(mesitylene) level of theory, under experimental temperature of 333.15 K. IF represents imaginary frequencies (cm^{-1}).

Species	*ZPE*	G_0	SP-E	E_c (ZPE + SP-E)	ΔE	G_c (G_0 + SP-E)	ΔG	IF
**IM5$_D$**	0.415815	0.314967	-1075.853395	-1075.43758		-1075.53843		
$CsHCO_3$	0.035708	-0.008276	-284.587081	-284.551373		-284.59536		
**IM5$_D$** + $CsHCO_3$	0.451523	0.306691	-1360.440476	-1359.988953	0.0	-1360.13379	0.0	
**IM6$_D$**	0.453805	0.330767	-1360.477568	-1360.023763	-21.8	-1360.14680	-8.2	
**TS4$_D$**	0.452247	0.332552	-1360.467566	-1360.015319	-16.5	-1360.13501	-0.8	193.20*i*
**IM7$_D$**	0.452561	0.334971	-1360.483956	-1360.031395	-26.6	-1360.14899	-9.5	
**IM6'$_D$**	0.453768	0.331553	-1360.469372	-1360.015604	-16.7	-1360.13782	-2.5	
**TS4'$_D$**	0.451485	0.331413	-1360.445645	-1359.99416	-3.3	-1360.11423	12.3	285.15*i*
**IM7'$_D$**	0.453214	0.33289	-1360.478437	-1360.025223	-22.8	-1360.14555	-7.4	

11. Copies of ^{1}H, ^{13}C NMR Spectra

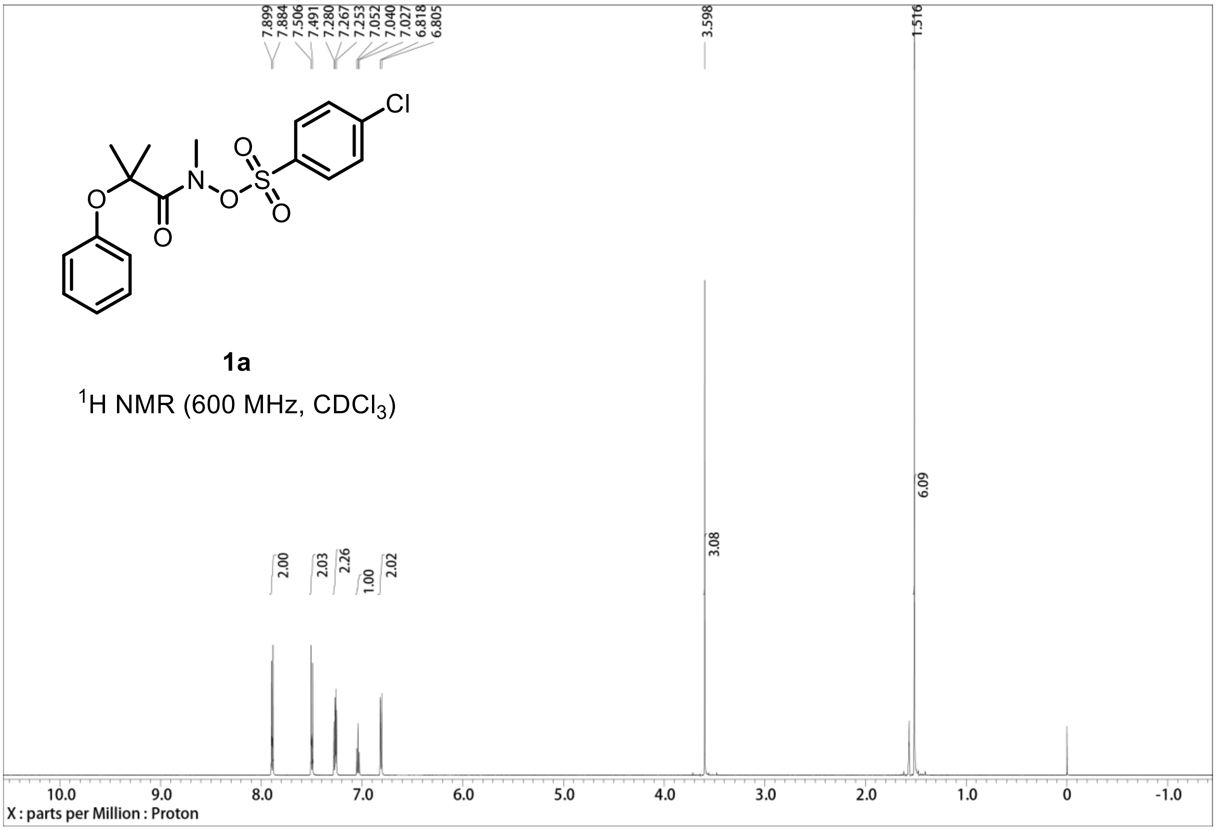

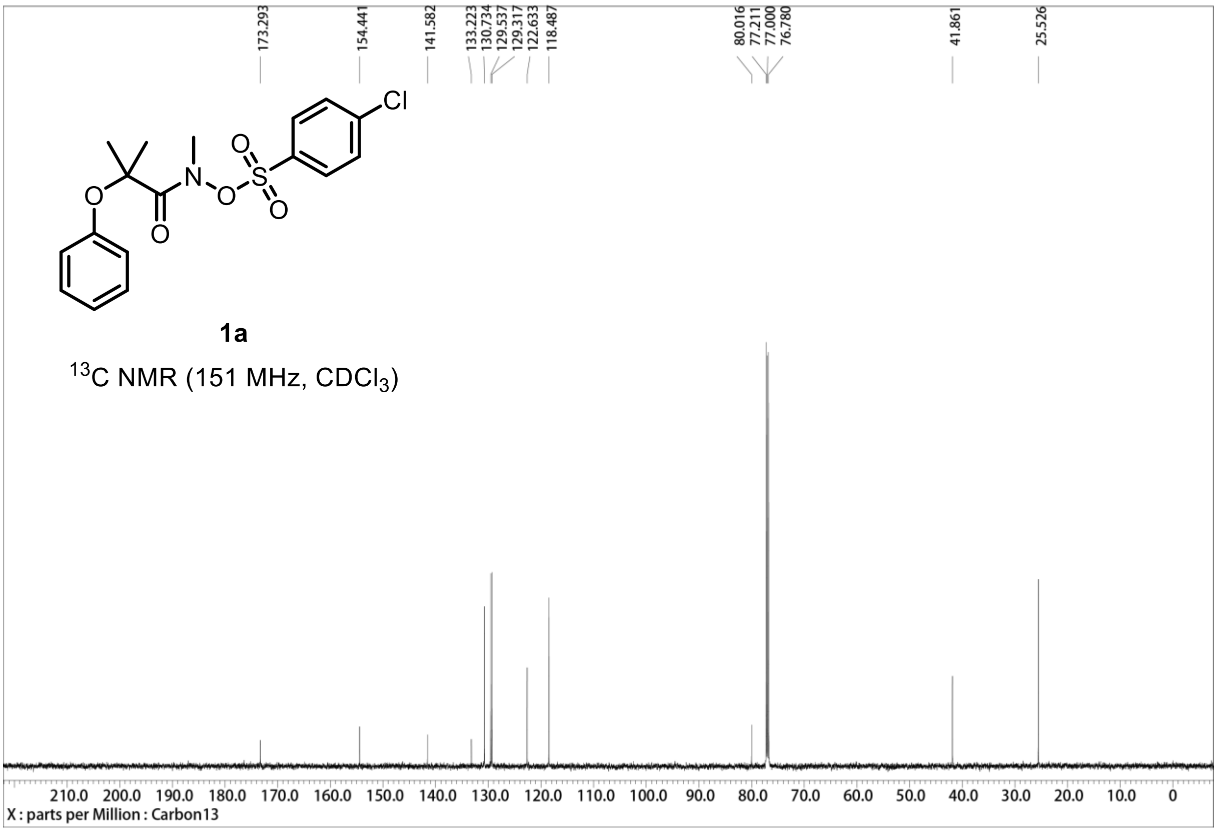

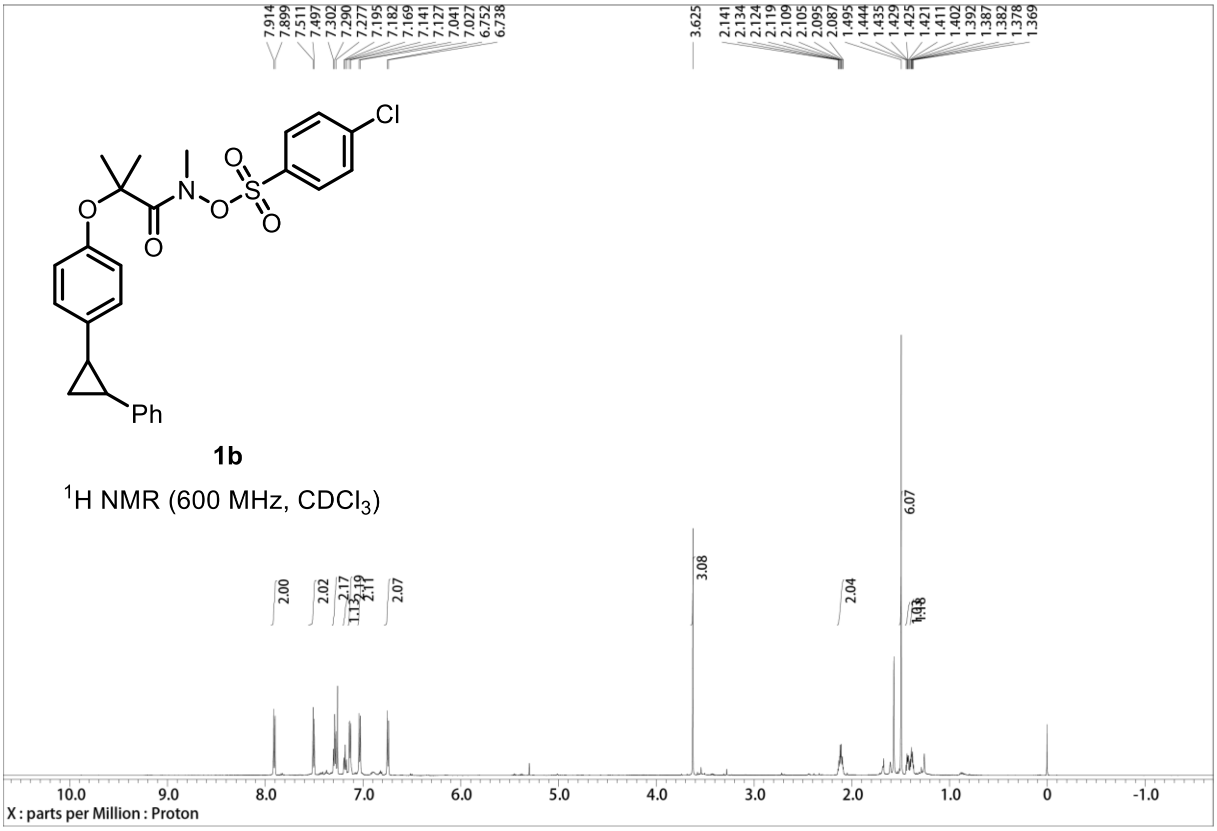

1b

^{1}H NMR (600 MHz, $CDCl_3$)

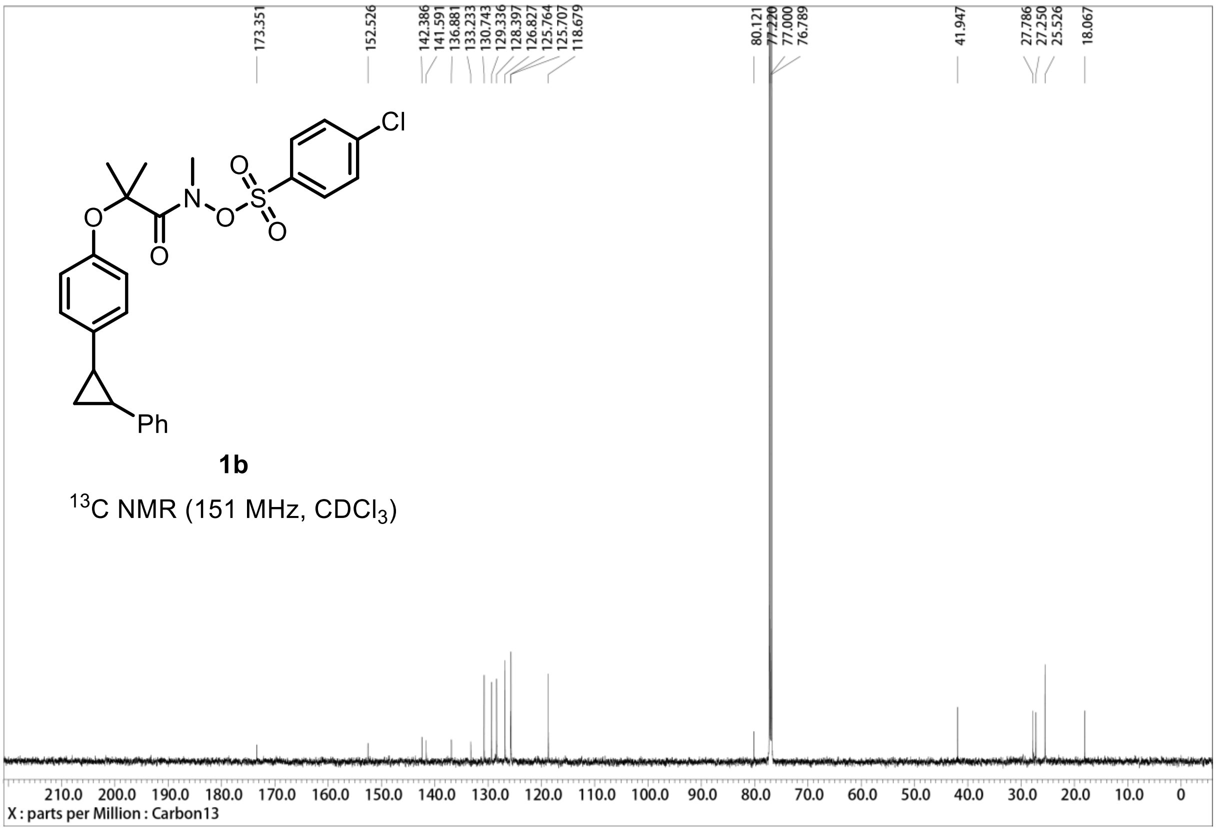

1b

^{13}C NMR (151 MHz, $CDCl_3$)

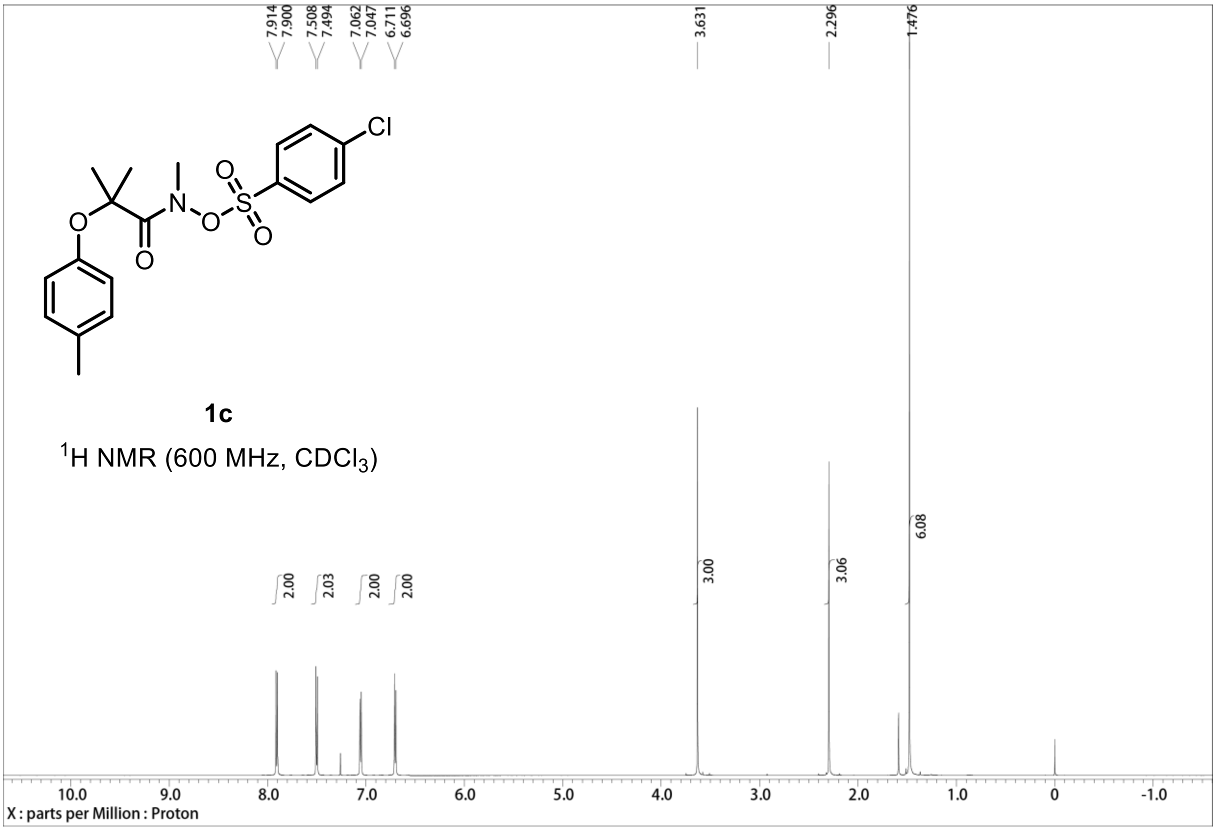
7.914
7.900
7.508
7.494
7.062
7.047
6.711
6.696
3.631
2.296
1.476
Cl
O
O
S
N
O
O
O
1c
^{1}H NMR (600 MHz, CDCl$_3$)
2.00
2.03
2.00
2.00
3.00
3.06
6.08
10.0 9.0 8.0 7.0 6.0 5.0 4.0 3.0 2.0 1.0 0 -1.0
X : parts per Million : Proton

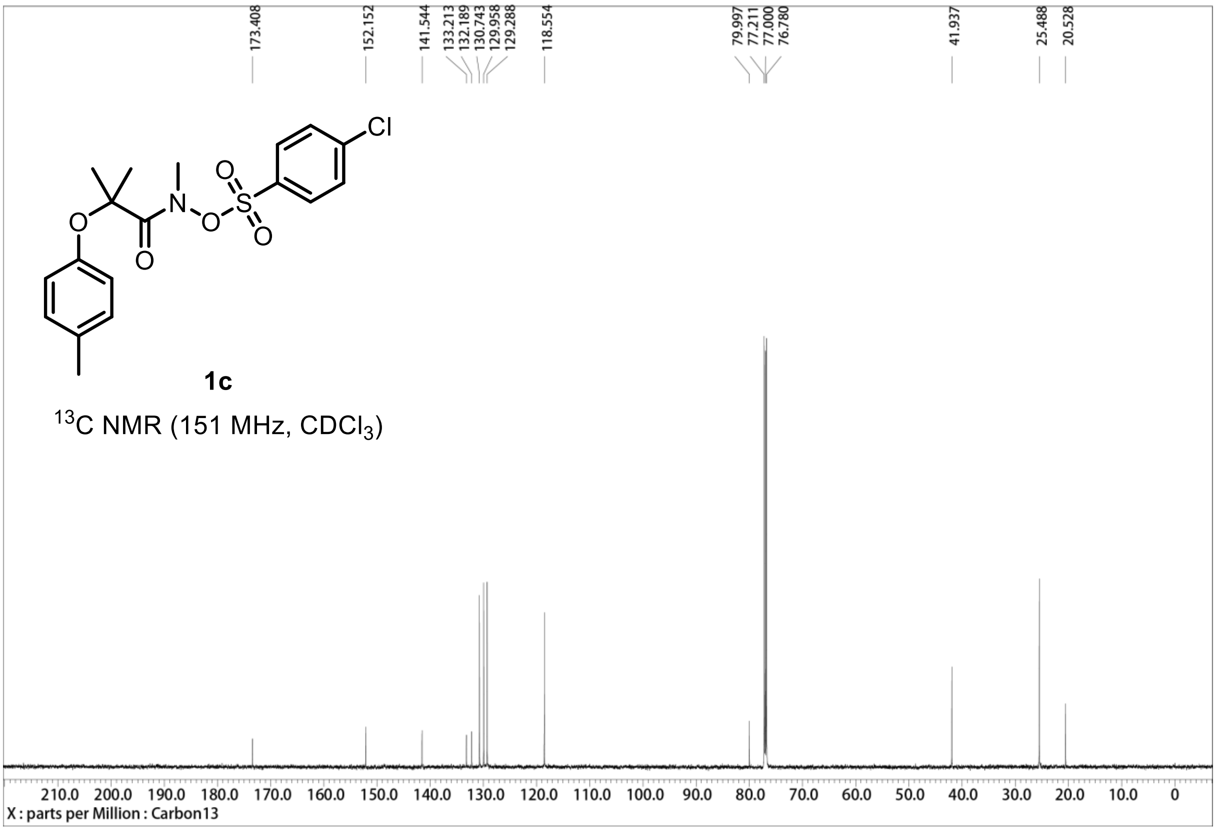
173.408
152.152
141.544
133.213
132.189
130.743
129.958
129.288
118.554
79.997
77.211
77.000
76.780
41.937
25.488
20.528
Cl
O
O
S
N
O
O
O
1c
^{13}C NMR (151 MHz, CDCl$_3$)
210.0 200.0 190.0 180.0 170.0 160.0 150.0 140.0 130.0 120.0 110.0 100.0 90.0 80.0 70.0 60.0 50.0 40.0 30.0 20.0 10.0 0
X : parts per Million : Carbon13

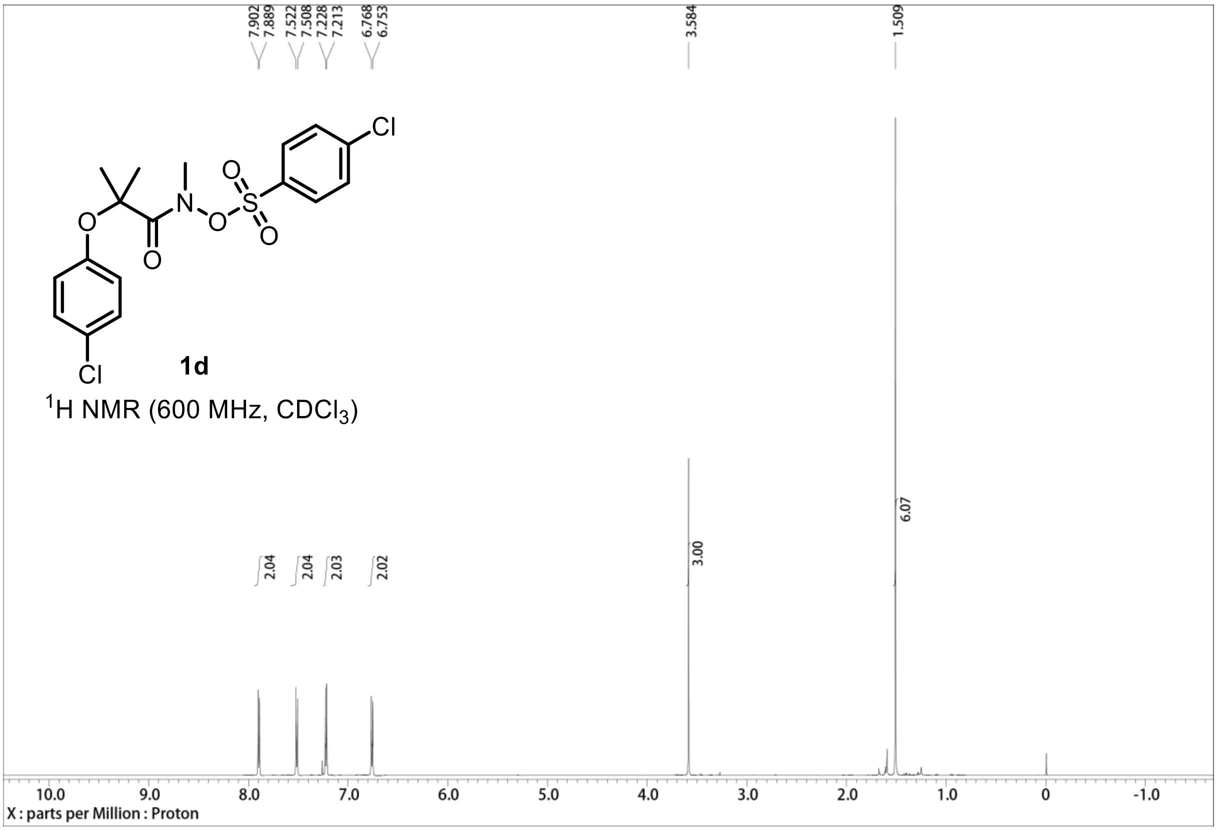
7.902
7.889
7.522
7.508
7.228
7.213
6.768
6.753
3.584
1.509
Cl
O
S
O
N
O
O
O
O
Cl
1d
^{1}H NMR (600 MHz, CDCl$_3$)
2.04
2.04
2.03
2.02
3.00
6.07
X : parts per Million : Proton

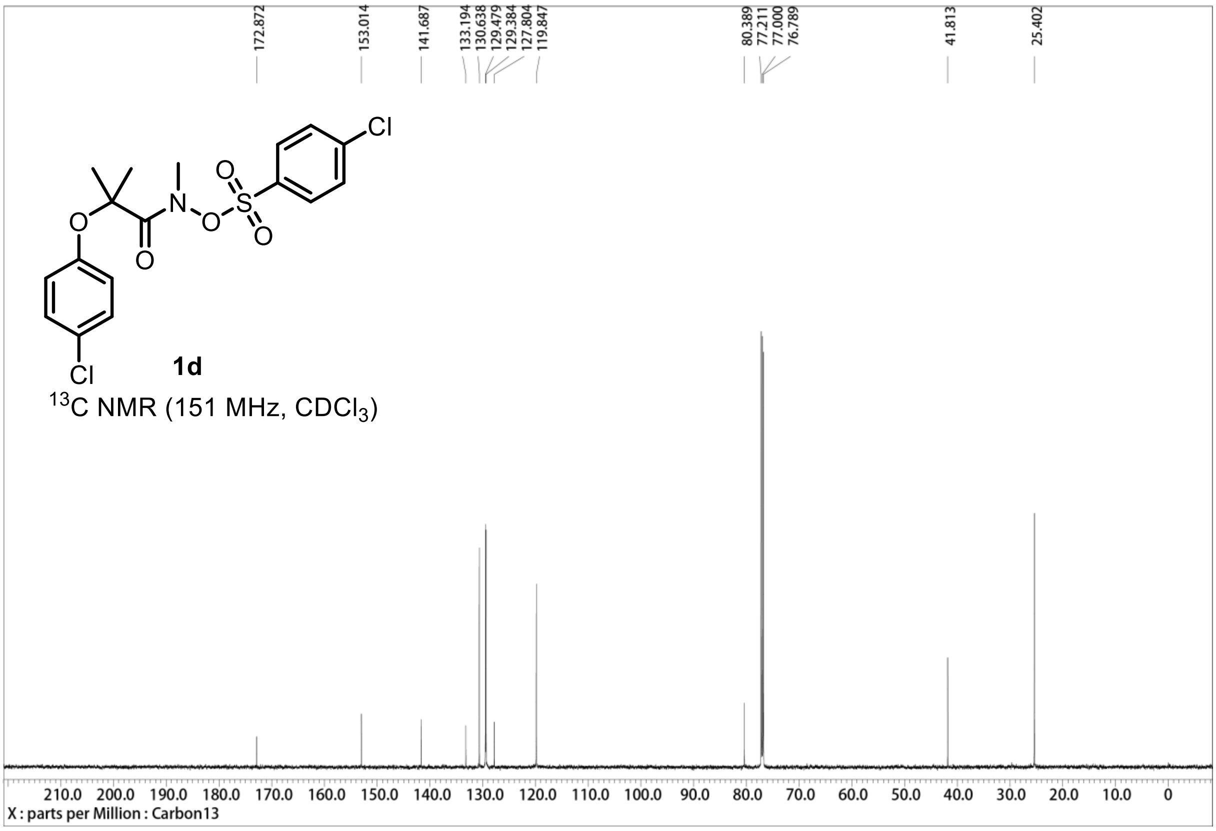
172.872
153.014
141.687
133.194
130.638
129.479
129.384
127.804
119.847
80.389
77.211
77.000
76.789
41.813
25.402
Cl
O
S
O
N
O
O
O
O
Cl
1d
^{13}C NMR (151 MHz, CDCl$_3$)
X : parts per Million : Carbon13

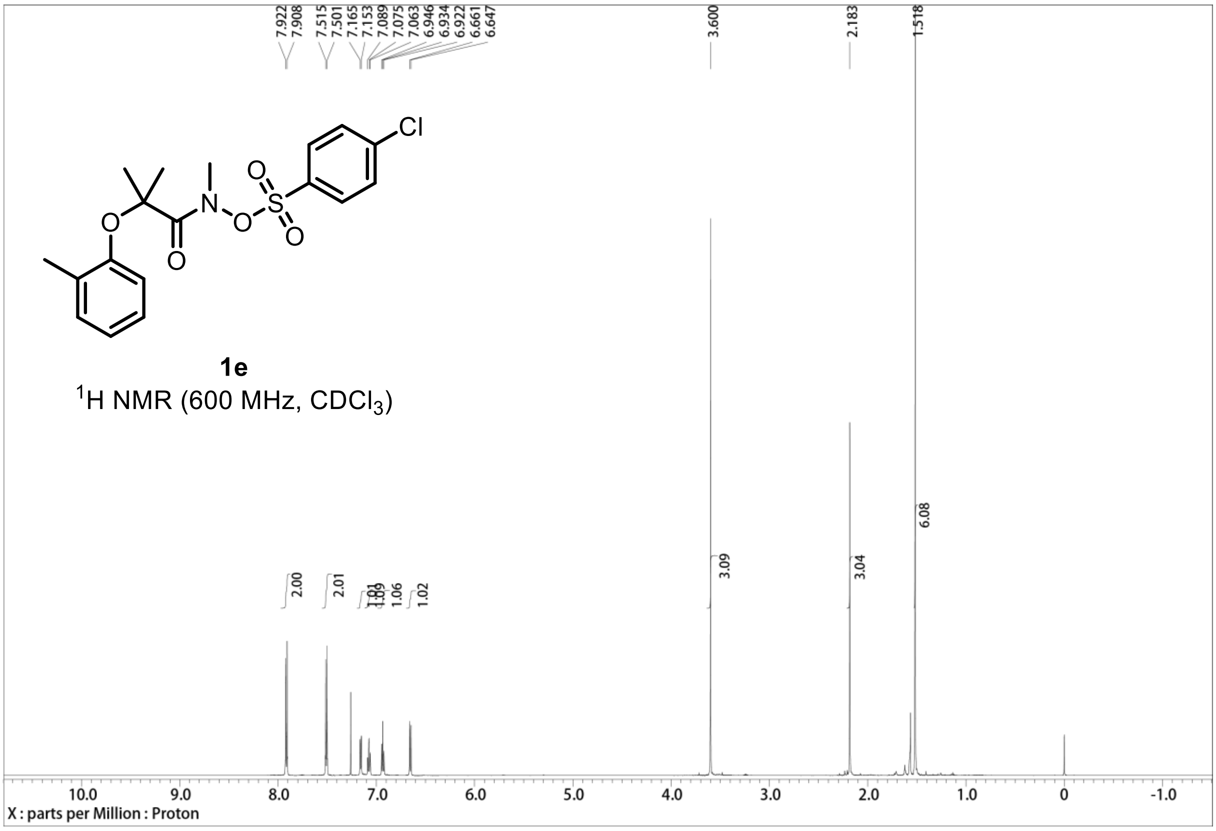

7.922
7.908
7.515
7.501
7.165
7.153
7.089
7.075
7.063
6.946
6.934
6.922
6.661
6.647
3.600
2.183
1.518
Cl
O
S
O
N
O
O
O
1e
1H NMR (600 MHz, CDCl3)
2.00
2.01
1.01
1.09
1.06
1.02
3.09
3.04
6.08
10.0
9.0
8.0
7.0
6.0
5.0
4.0
3.0
2.0
1.0
0
-1.0
X : parts per Million : Proton

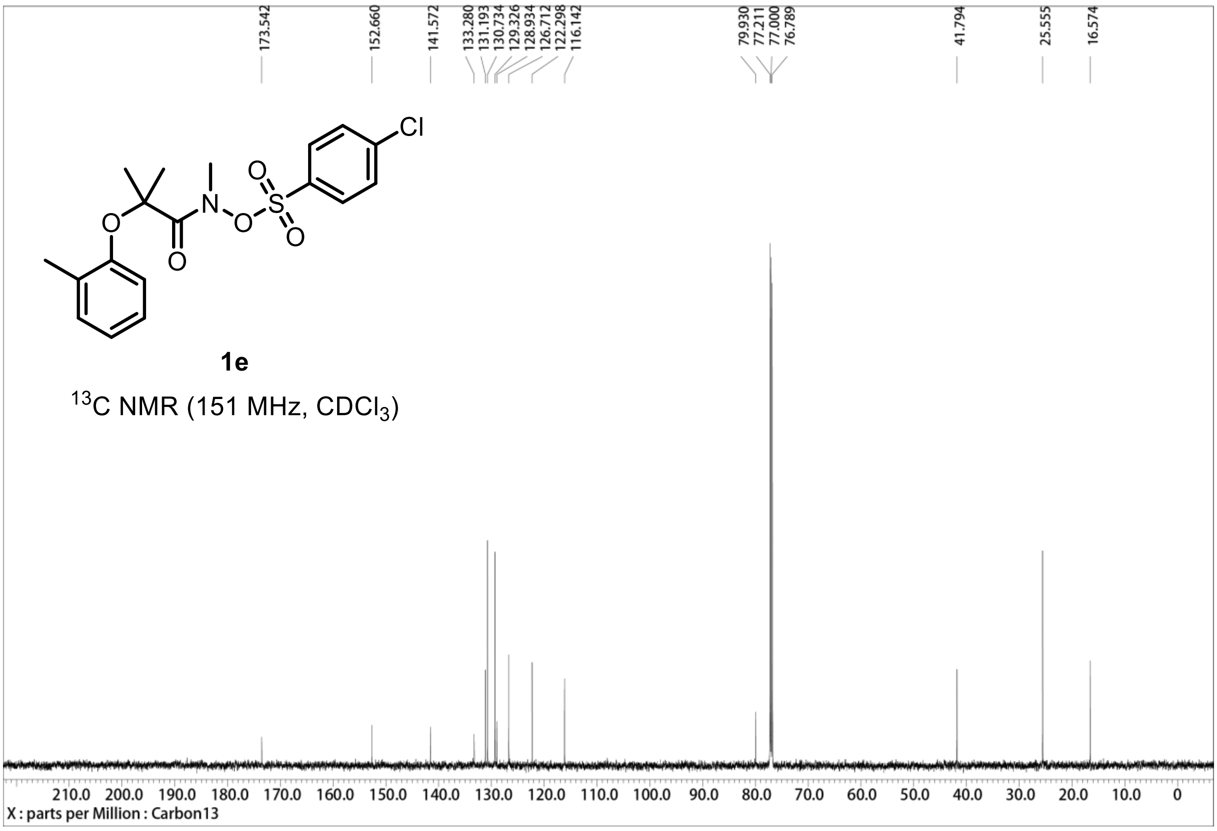

173.542
152.660
141.572
133.280
131.193
130.734
129.326
128.934
126.712
122.298
116.142
79.930
77.211
77.000
76.789
41.794
25.555
16.574
Cl
O
S
O
N
O
O
O
1e
13C NMR (151 MHz, CDCl3)
210.0
200.0
190.0
180.0
170.0
160.0
150.0
140.0
130.0
120.0
110.0
100.0
90.0
80.0
70.0
60.0
50.0
40.0
30.0
20.0
10.0
0
X : parts per Million : Carbon13

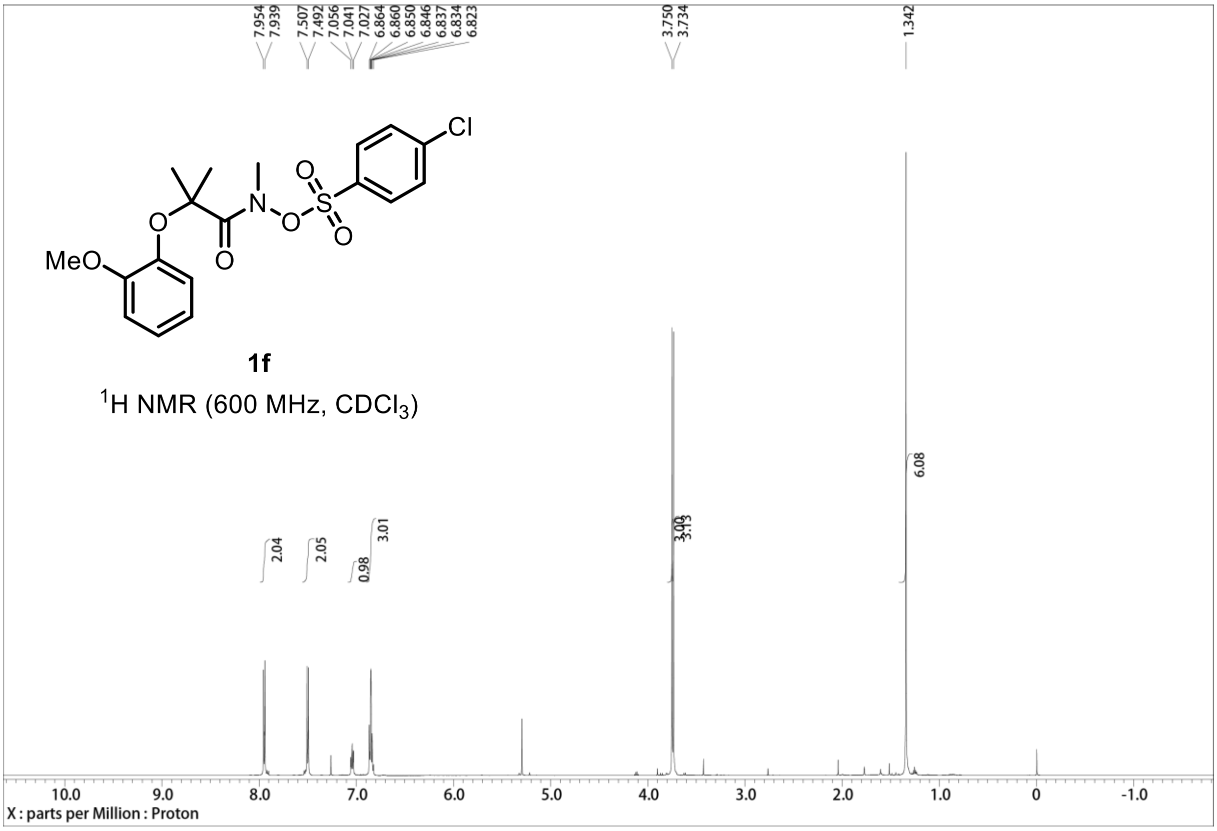
7.954
7.939
7.507
7.492
7.056
7.041
7.027
6.864
6.860
6.850
6.846
6.837
6.834
6.823
3.750
3.734
1.342
Cl
O
O
S
N
O
O
MeO
O
1f
^{1}H NMR (600 MHz, CDCl$_3$)
2.04
2.05
0.98
3.01
3.00
3.13
6.08
10.0
9.0
8.0
7.0
6.0
5.0
4.0
3.0
2.0
1.0
0
-1.0
X : parts per Million : Proton

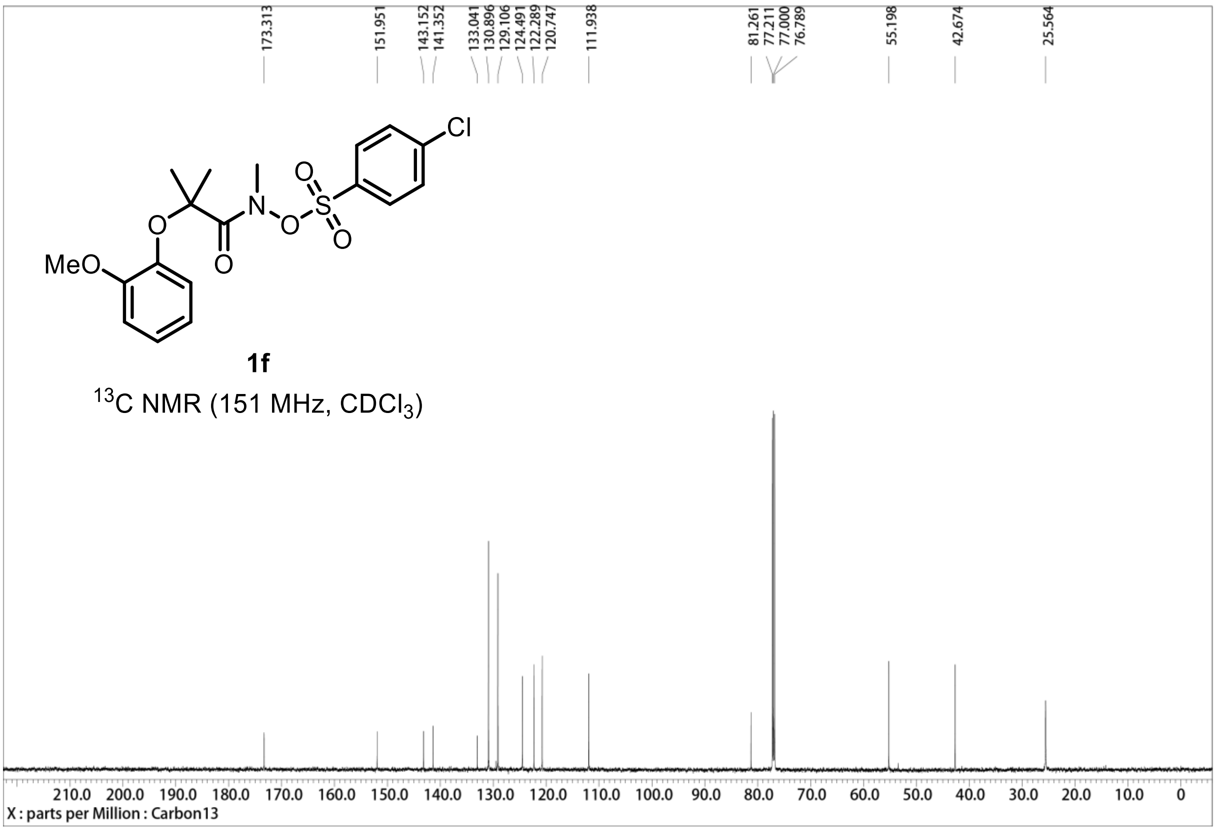
173.313
151.951
143.152
141.352
133.041
130.896
129.106
124.491
122.289
120.747
111.938
81.261
77.211
77.000
76.789
55.198
42.674
25.564
Cl
O
O
S
N
O
O
MeO
O
1f
^{13}C NMR (151 MHz, CDCl$_3$)
210.0
200.0
190.0
180.0
170.0
160.0
150.0
140.0
130.0
120.0
110.0
100.0
90.0
80.0
70.0
60.0
50.0
40.0
30.0
20.0
10.0
0
X : parts per Million : Carbon13

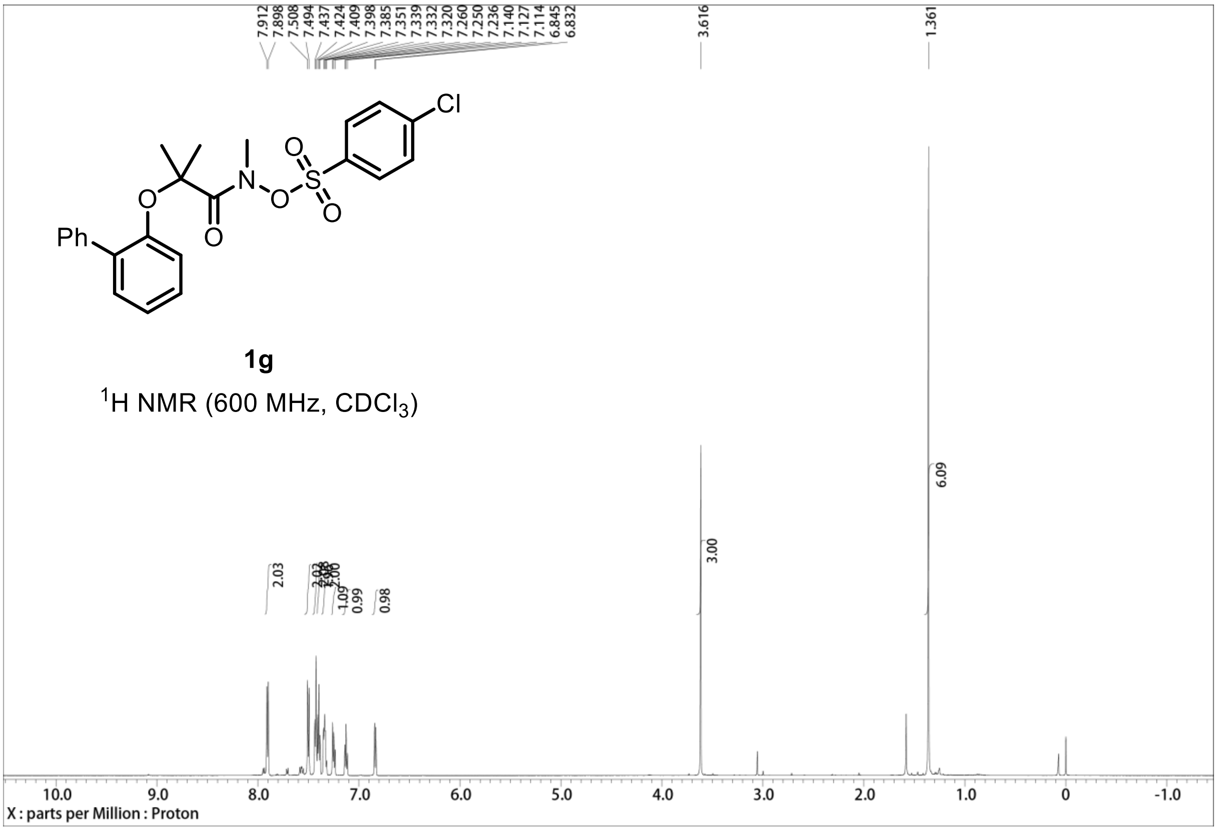

7.912
7.898
7.508
7.494
7.437
7.424
7.409
7.398
7.385
7.351
7.339
7.332
7.320
7.260
7.250
7.236
7.140
7.127
7.114
6.845
6.832
3.616
1.361
Cl
O
O
S
N
O
O
O
O
Ph
1g
^{1}H NMR (600 MHz, CDCl$_3$)
6.09
3.00
2.03
0.99
0.98
10.0
9.0
8.0
7.0
6.0
5.0
4.0
3.0
2.0
1.0
0
-1.0
X : parts per Million : Proton

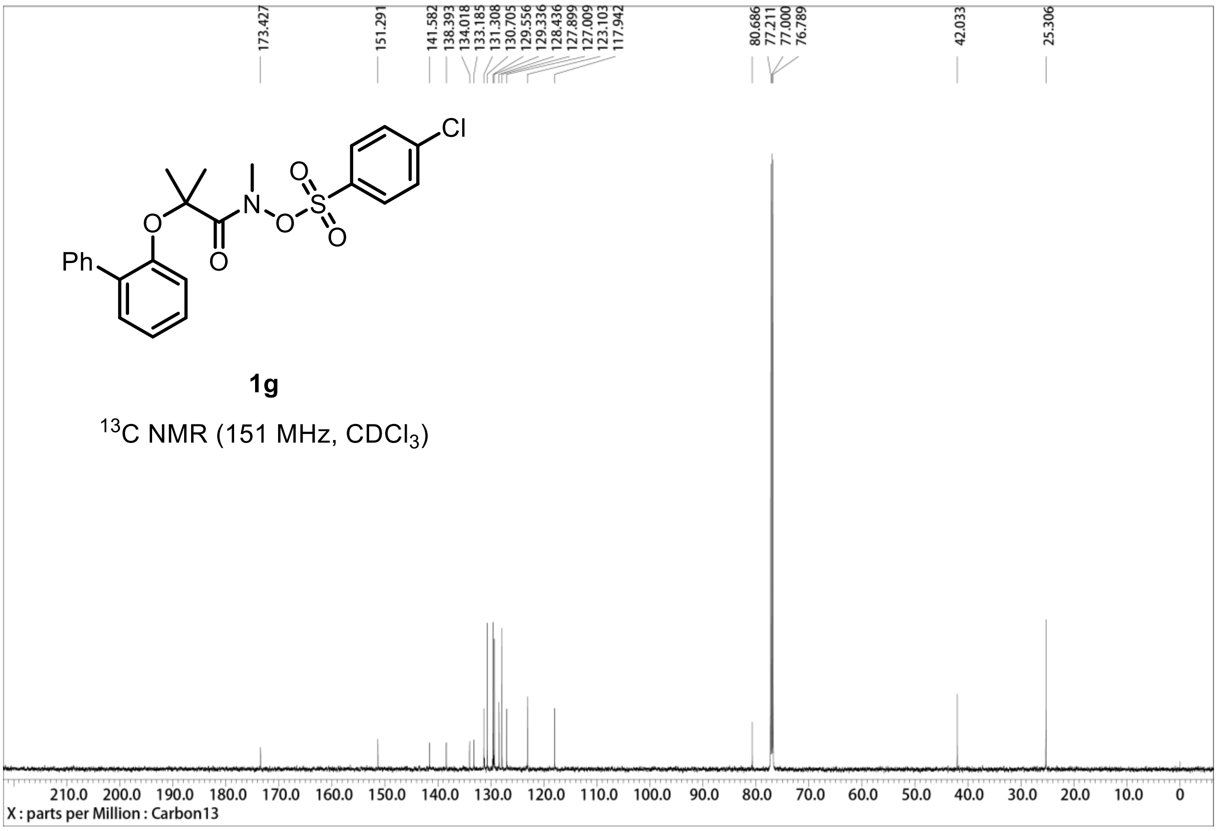

173.427
151.291
141.582
138.393
134.018
133.185
131.308
130.705
129.556
129.336
128.436
127.899
127.009
123.103
117.942
80.686
77.211
77.000
76.789
42.033
25.306
Cl
Ph
1g
^{13}C NMR (151 MHz, CDCl$_3$)
210.0
200.0
190.0
180.0
170.0
160.0
150.0
140.0
130.0
120.0
110.0
100.0
90.0
80.0
70.0
60.0
50.0
40.0
30.0
20.0
10.0
0
X : parts per Million : Carbon13

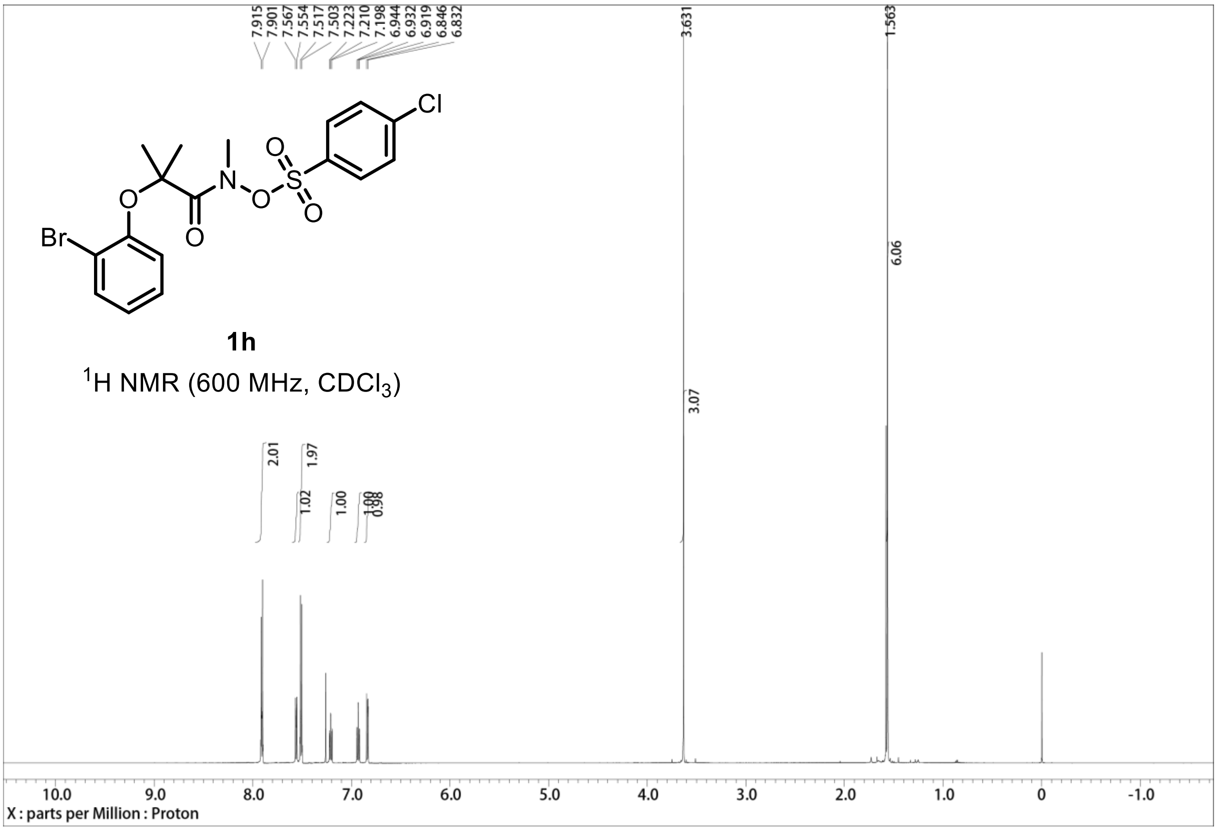

7.915
7.901
7.567
7.554
7.517
7.503
7.223
7.210
7.198
6.944
6.932
6.919
6.846
6.832
3.631
1.563
Cl
O
S
O
N
O
O
O
Br
1h
^{1}H NMR (600 MHz, CDCl$_3$)
6.06
3.07
2.01
1.97
1.02
1.00
10.0
9.0
8.0
7.0
6.0
5.0
4.0
3.0
2.0
1.0
0
-1.0
X : parts per Million : Proton

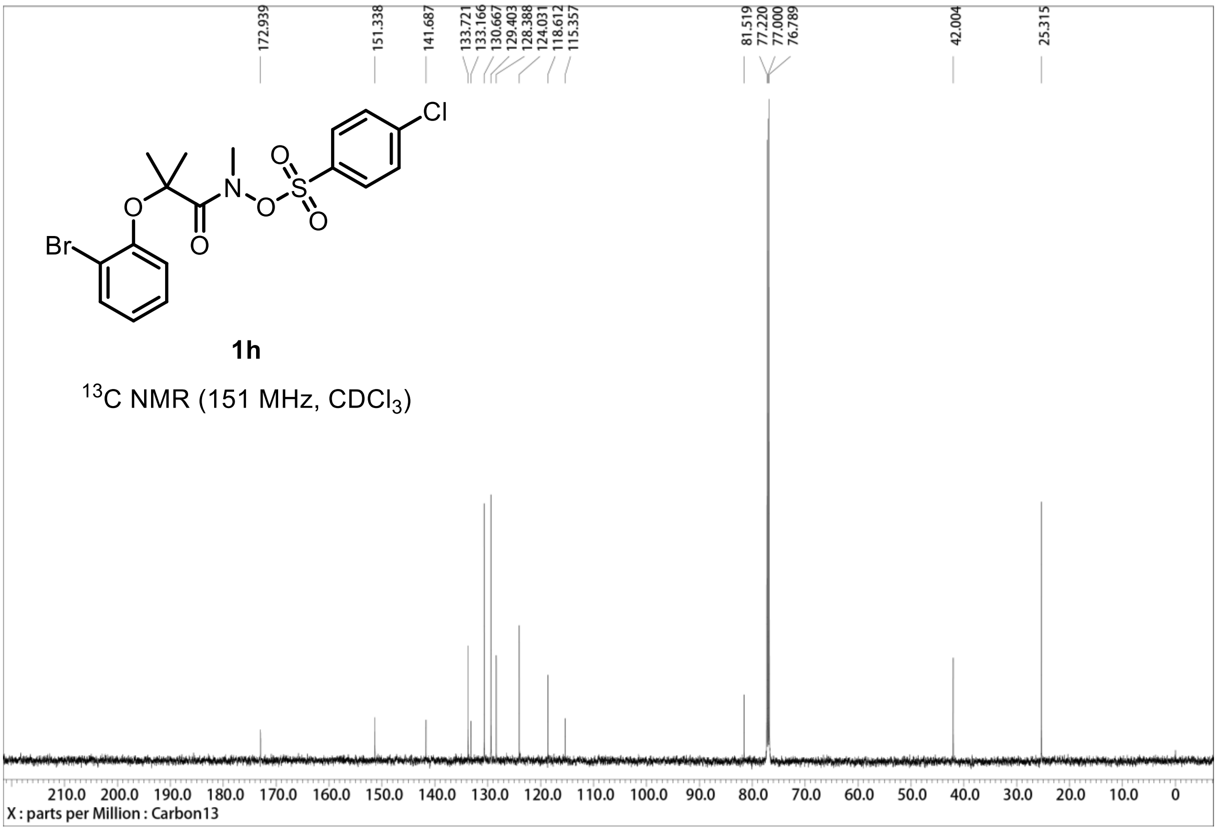

172.939
151.338
141.687
133.721
133.166
130.667
129.403
128.388
124.031
118.612
115.357
81.519
77.220
77.000
76.789
42.004
25.315
Cl
O
S
O
N
O
O
O
Br
1h
^{13}C NMR (151 MHz, CDCl$_3$)
210.0
200.0
190.0
180.0
170.0
160.0
150.0
140.0
130.0
120.0
110.0
100.0
90.0
80.0
70.0
60.0
50.0
40.0
30.0
20.0
10.0
0
X : parts per Million : Carbon13

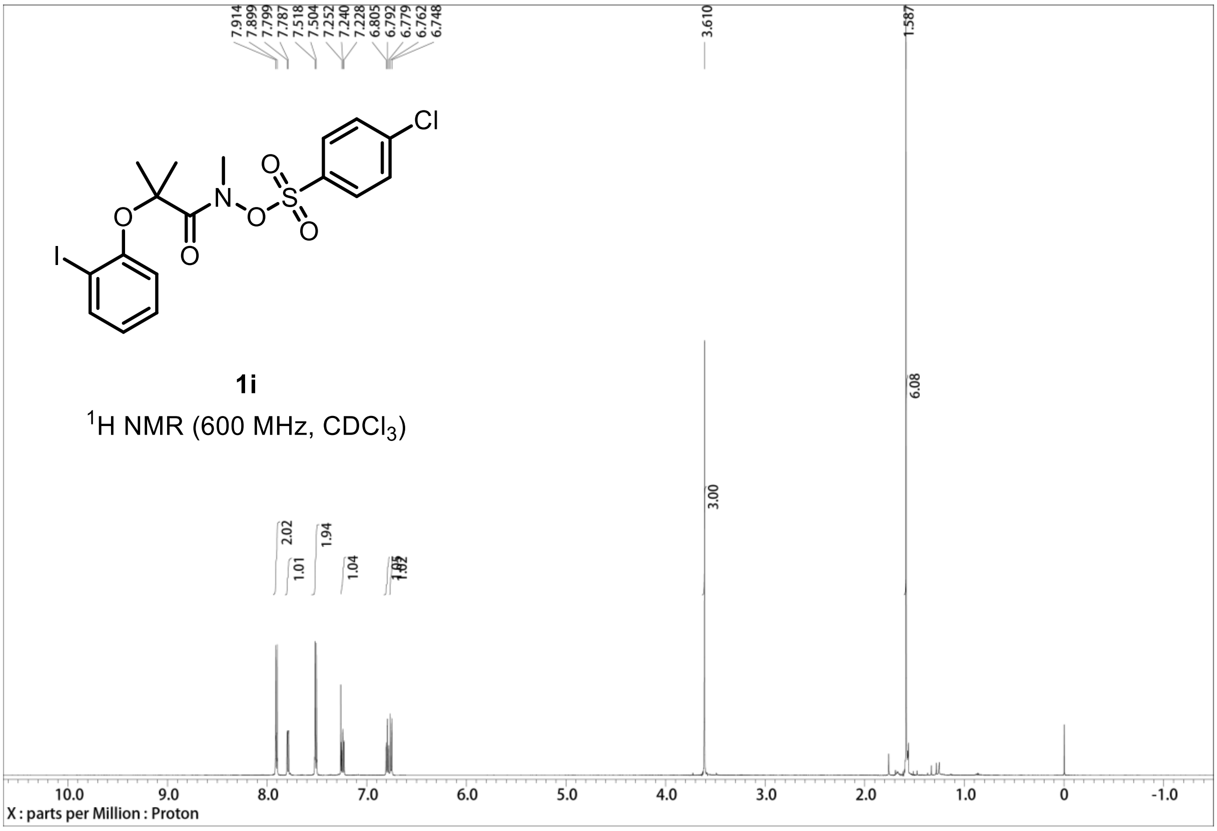
7.914 7.899 7.799 7.787 7.518 7.504 7.252 7.240 7.228 6.805 6.792 6.779 6.762 6.748
3.610
1.587
Cl
O
S
O
N
O
O
O
I
1i
^{1}H NMR (600 MHz, $CDCl_3$)
2.02
1.01
1.94
1.04
3.00
6.08
10.0 9.0 8.0 7.0 6.0 5.0 4.0 3.0 2.0 1.0 0 -1.0
X : parts per Million : Proton

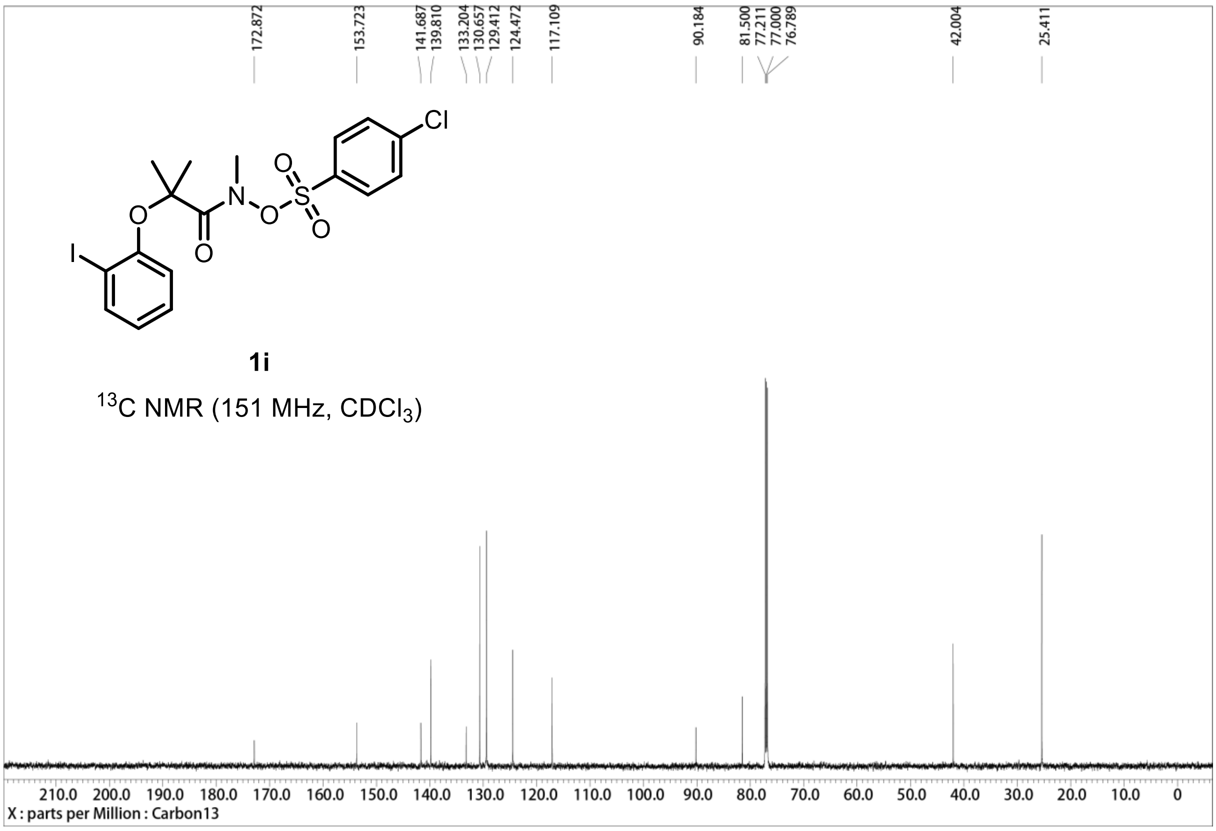
172.872
153.723
141.687 139.810
133.204 130.657 129.412 124.472
117.109
90.184
81.500 77.211 77.000 76.789
42.004
25.411
Cl
O
S
O
N
O
O
O
I
1i
^{13}C NMR (151 MHz, $CDCl_3$)
210.0 200.0 190.0 180.0 170.0 160.0 150.0 140.0 130.0 120.0 110.0 100.0 90.0 80.0 70.0 60.0 50.0 40.0 30.0 20.0 10.0 0
X : parts per Million : Carbon13

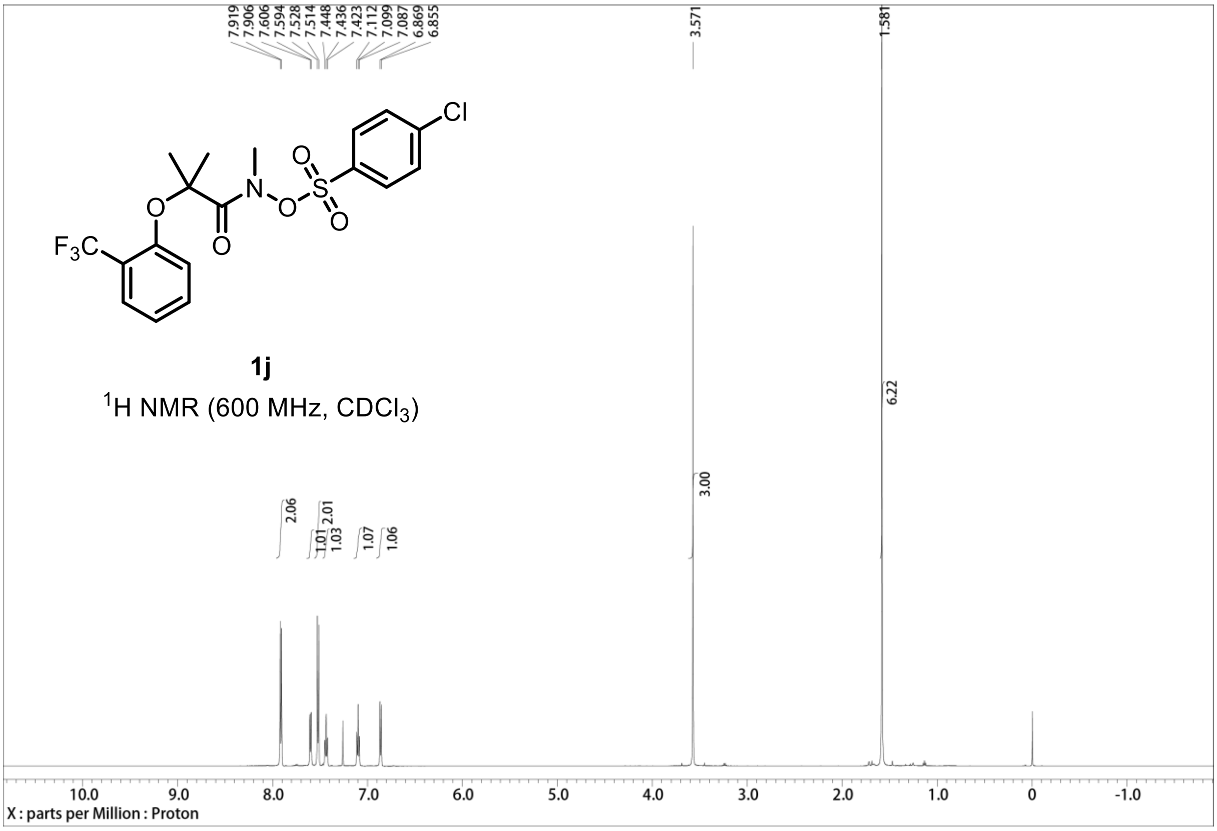
7.919
7.906
7.606
7.594
7.528
7.514
7.448
7.436
7.423
7.112
7.099
7.087
6.869
6.855
3.571
1.581
Cl
O
N
O
S
O
O
O
O
F3C
1j
1H NMR (600 MHz, CDCl3)
2.06
1.01
2.01
1.03
1.07
1.06
3.00
6.22
X : parts per Million : Proton

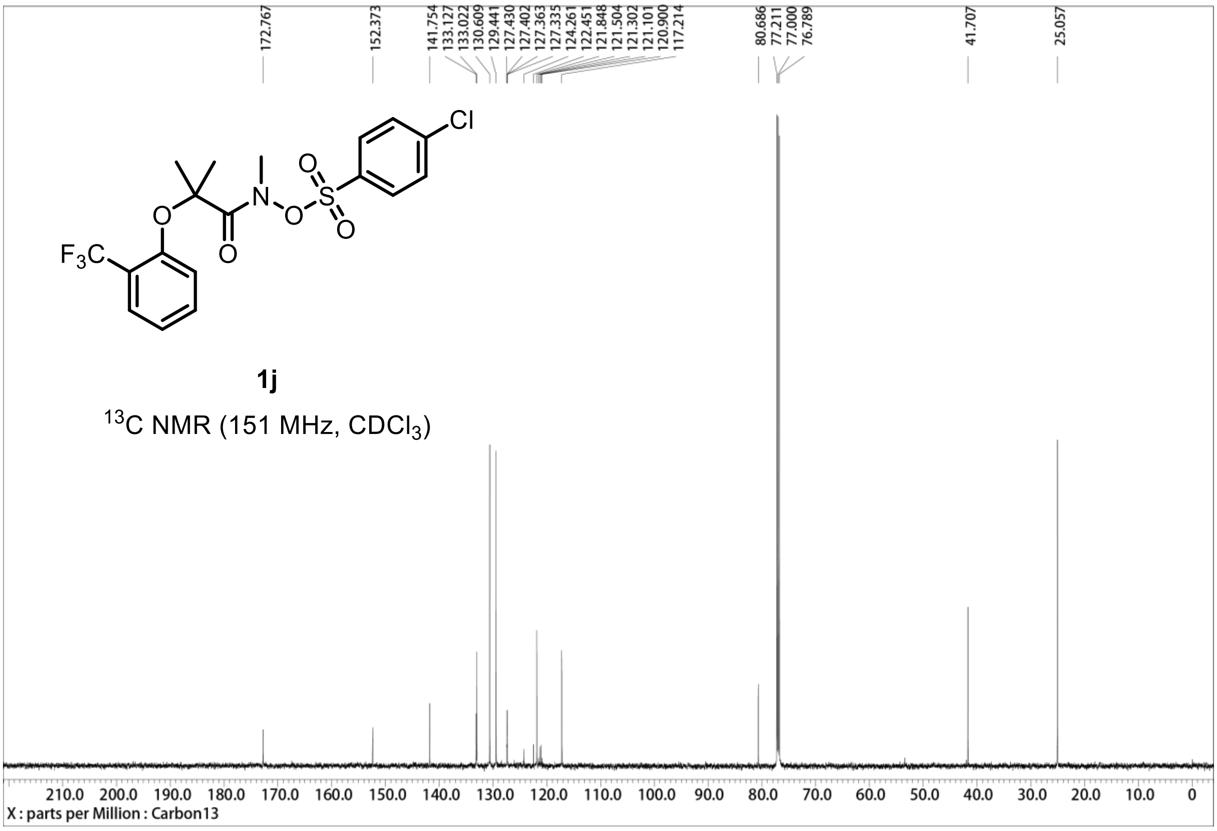
172.767
152.373
141.754
133.127
133.022
130.609
129.441
127.430
127.402
127.363
127.335
124.261
122.451
121.848
121.504
121.302
121.101
120.900
117.214
80.686
77.211
77.000
76.789
41.707
25.057
Cl
F3C
1j
13C NMR (151 MHz, CDCl3)
X : parts per Million : Carbon13

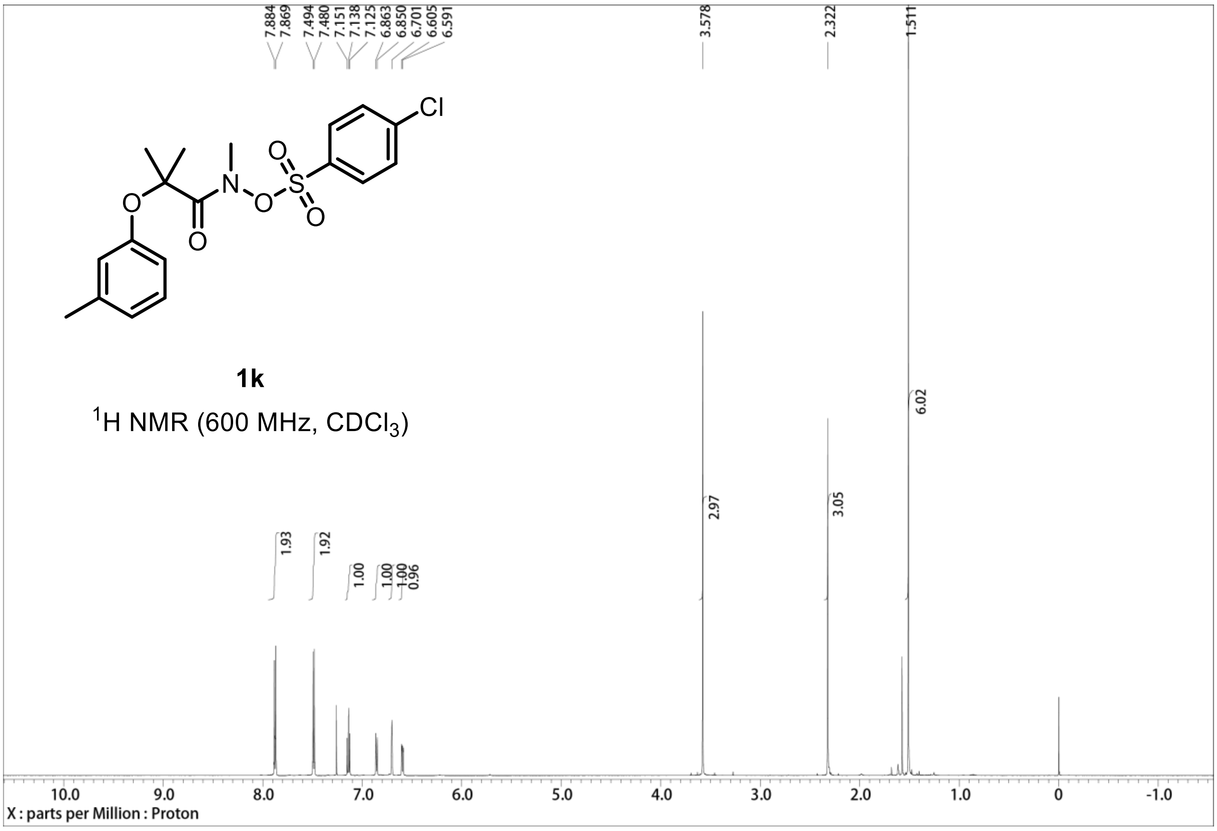
7.884
7.869
7.494
7.480
7.151
7.138
7.125
6.863
6.850
6.701
6.605
6.591
3.578
2.322
1.511
Cl
O
N
O
S
O
O
O
O
1k
1H NMR (600 MHz, CDCl3)
6.02
2.97
3.05
1.93
1.92
1.00
1.00
1.00
0.96
10.0
9.0
8.0
7.0
6.0
5.0
4.0
3.0
2.0
1.0
0
-1.0
X : parts per Million : Proton

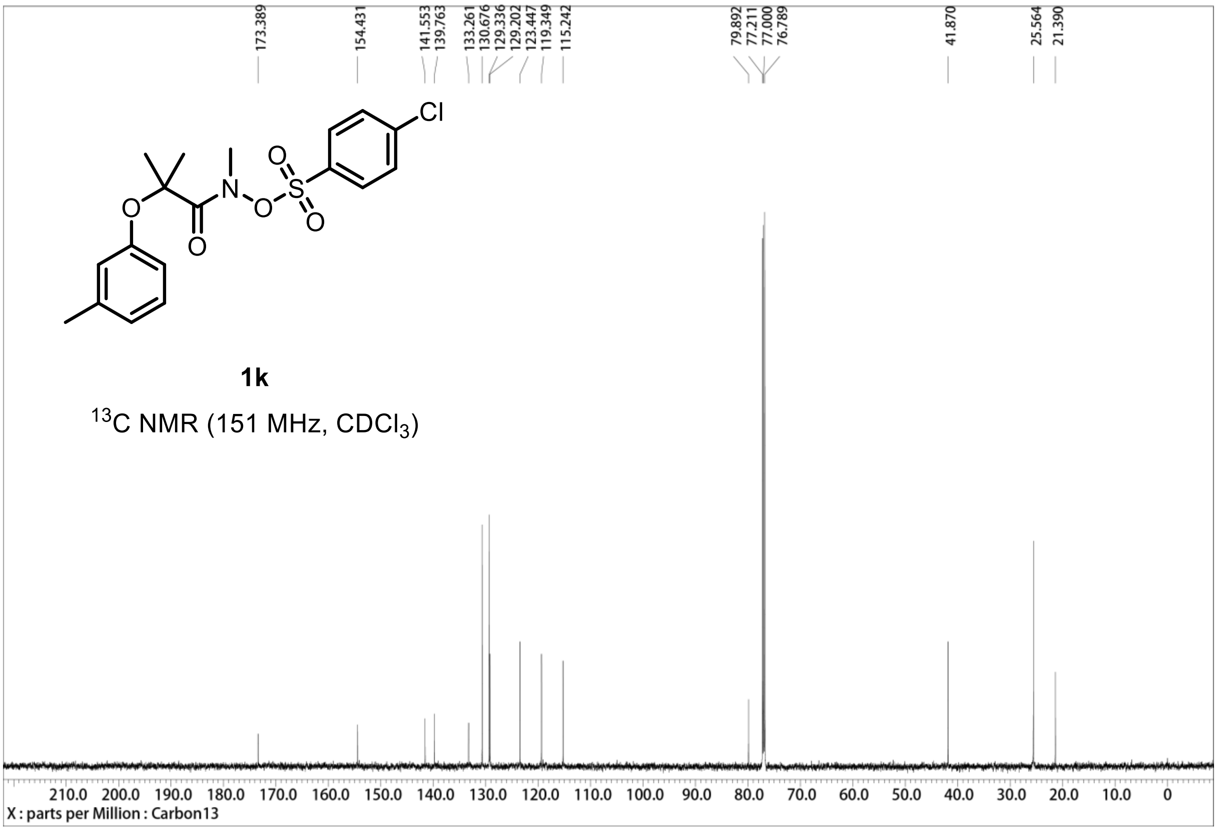
173.389
154.431
141.553
139.763
133.261
130.676
129.336
129.202
123.447
119.349
115.242
79.892
77.211
77.000
76.789
41.870
25.564
21.390
Cl
O
N
O
S
O
O
O
O
1k
13C NMR (151 MHz, CDCl3)
210.0
200.0
190.0
180.0
170.0
160.0
150.0
140.0
130.0
120.0
110.0
100.0
90.0
80.0
70.0
60.0
50.0
40.0
30.0
20.0
10.0
0
X : parts per Million : Carbon13

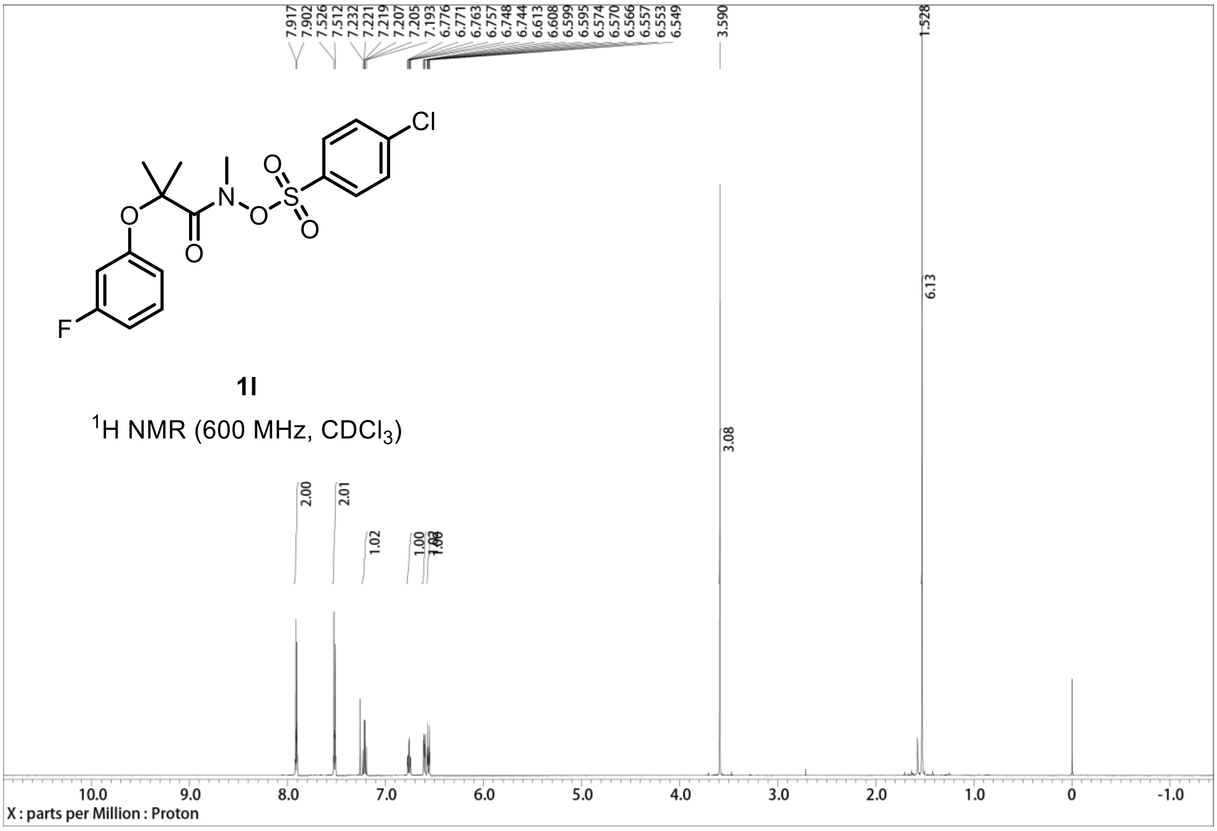
7.917 7.902 7.526 7.512 7.232 7.221 7.219 7.207 7.205 7.193 6.776 6.771 6.763 6.757 6.748 6.744 6.613 6.608 6.599 6.595 6.574 6.570 6.566 6.557 6.553 6.549
3.590
1.528
Cl
O
N
O
S
O
O
O
F
1l
1H NMR (600 MHz, CDCl3)
2.00
2.01
1.02
1.00
3.08
6.13
10.0 9.0 8.0 7.0 6.0 5.0 4.0 3.0 2.0 1.0 0 -1.0
X : parts per Million : Proton

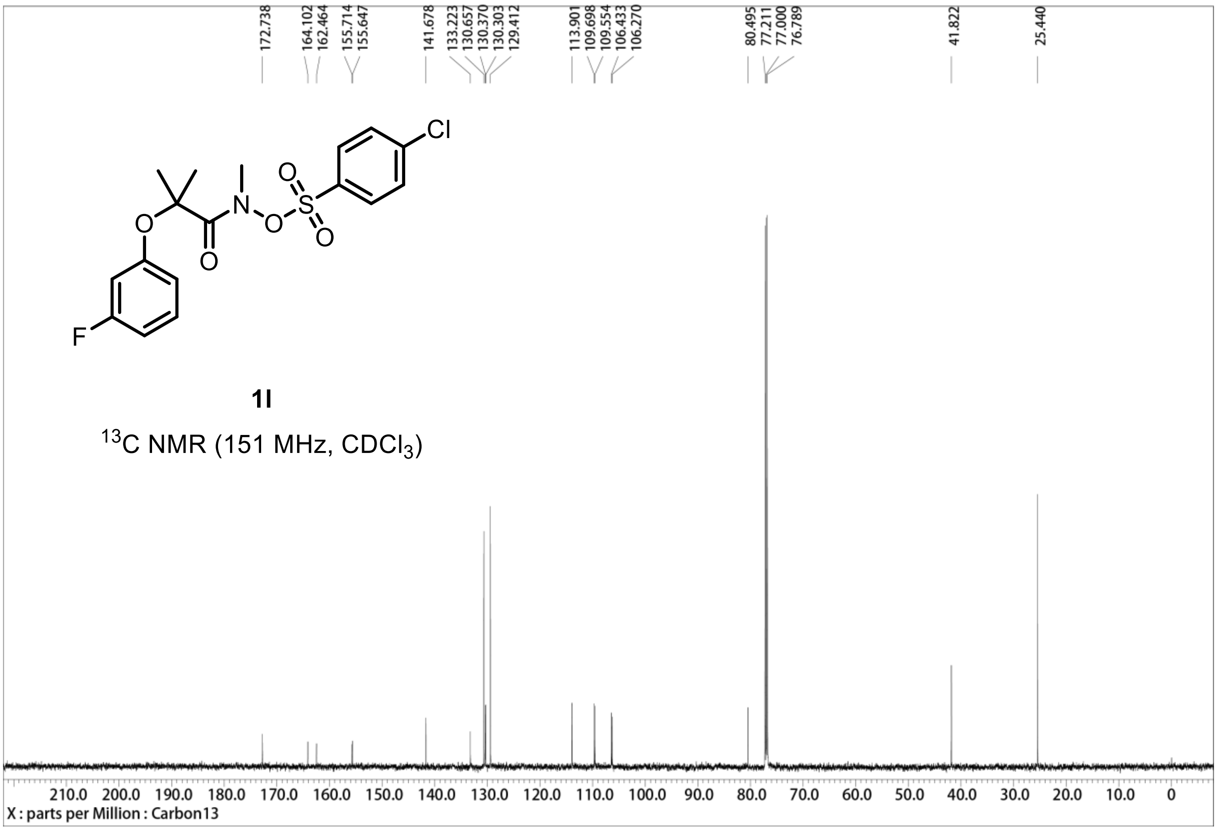
172.738
164.102 162.464
155.714 155.647
141.678
133.223 130.657 130.370 130.303 129.412
113.901 109.698 109.554 106.433 106.270
80.495 77.211 77.000 76.789
41.822
25.440
Cl
O
N
O
S
O
O
O
F
1l
13C NMR (151 MHz, CDCl3)
210.0 200.0 190.0 180.0 170.0 160.0 150.0 140.0 130.0 120.0 110.0 100.0 90.0 80.0 70.0 60.0 50.0 40.0 30.0 20.0 10.0 0
X : parts per Million : Carbon13

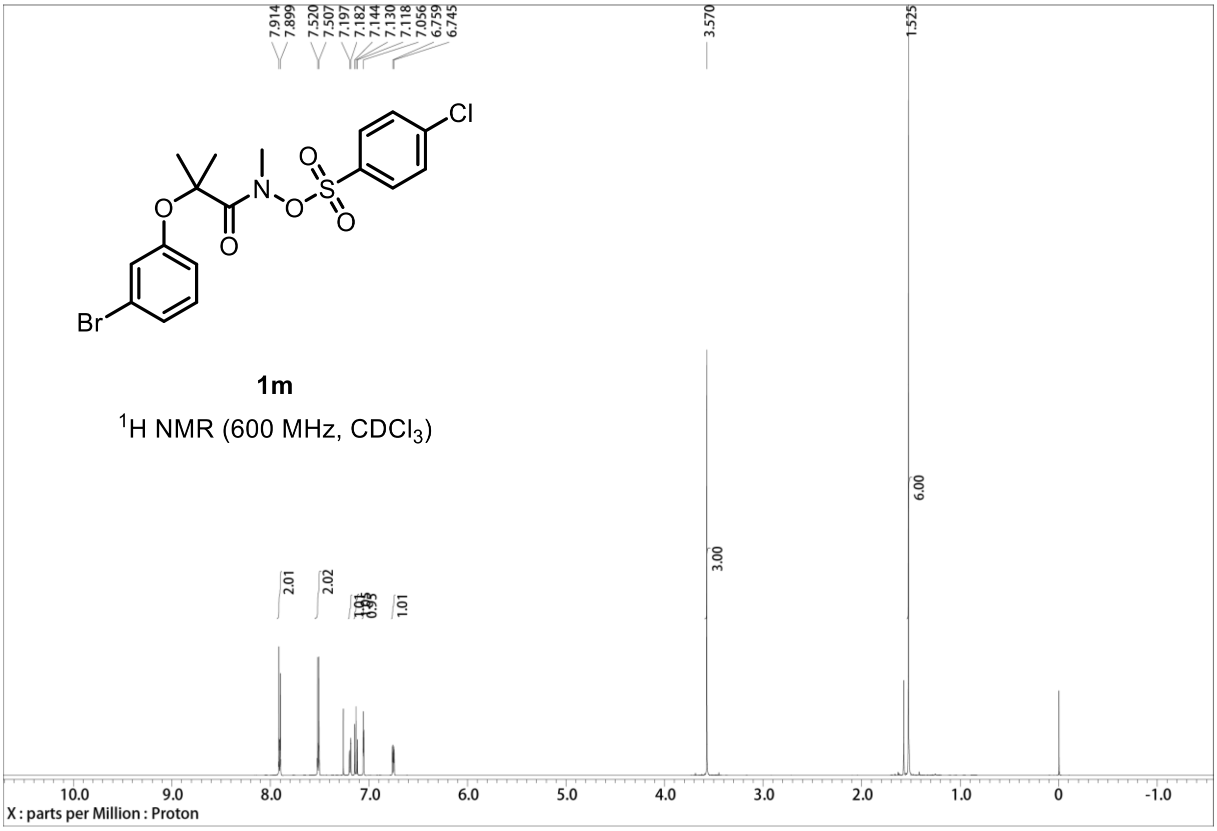
7.914
7.899
7.520
7.507
7.197
7.182
7.144
7.130
7.118
7.056
6.759
6.745
3.570
1.525
Cl
O
O
S
N
O
O
O
O
Br
1m
1H NMR (600 MHz, CDCl3)
2.01
2.02
1.01
3.00
6.00
10.0
9.0
8.0
7.0
6.0
5.0
4.0
3.0
2.0
1.0
0
-1.0
X : parts per Million : Proton

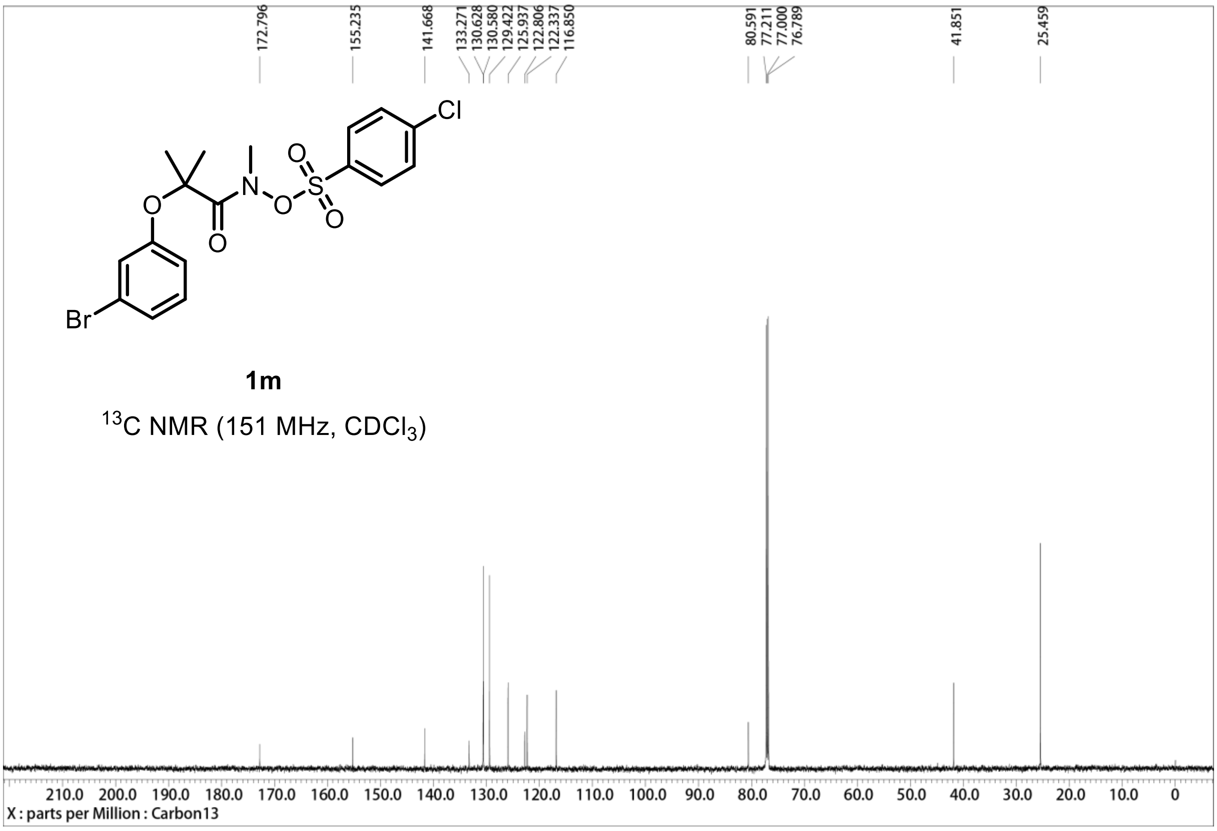
172.796
155.235
141.668
133.271
130.628
130.580
129.422
125.937
122.806
122.337
116.850
80.591
77.211
77.000
76.789
41.851
25.459
Cl
O
O
S
N
O
O
O
O
Br
1m
13C NMR (151 MHz, CDCl3)
210.0
200.0
190.0
180.0
170.0
160.0
150.0
140.0
130.0
120.0
110.0
100.0
90.0
80.0
70.0
60.0
50.0
40.0
30.0
20.0
10.0
0
X : parts per Million : Carbon13

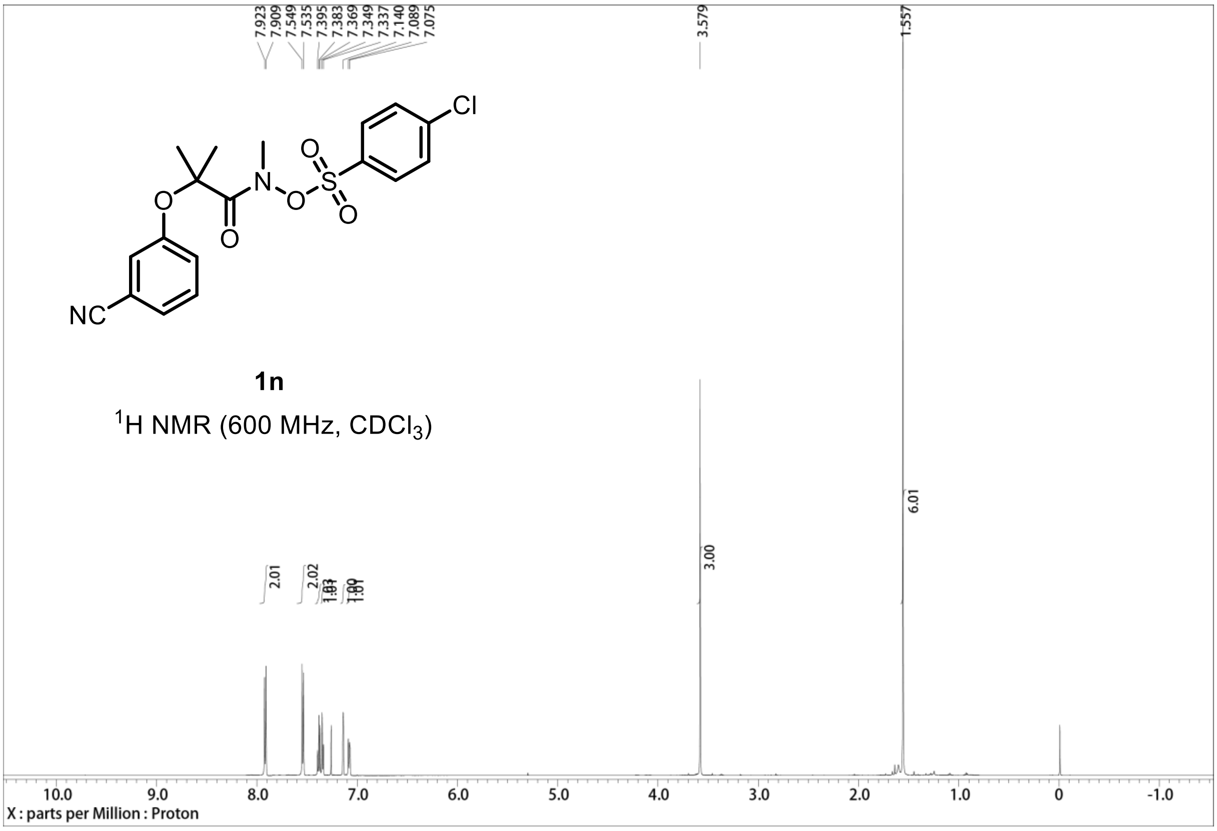

7.923
7.909
7.549
7.535
7.395
7.383
7.369
7.349
7.337
7.140
7.089
7.075
3.579
1.557
Cl
O
S
N
O
O
O
O
NC
1n
1H NMR (600 MHz, CDCl3)
2.01
2.02
3.00
6.01
10.0
9.0
8.0
7.0
6.0
5.0
4.0
3.0
2.0
1.0
0
-1.0
X : parts per Million : Proton

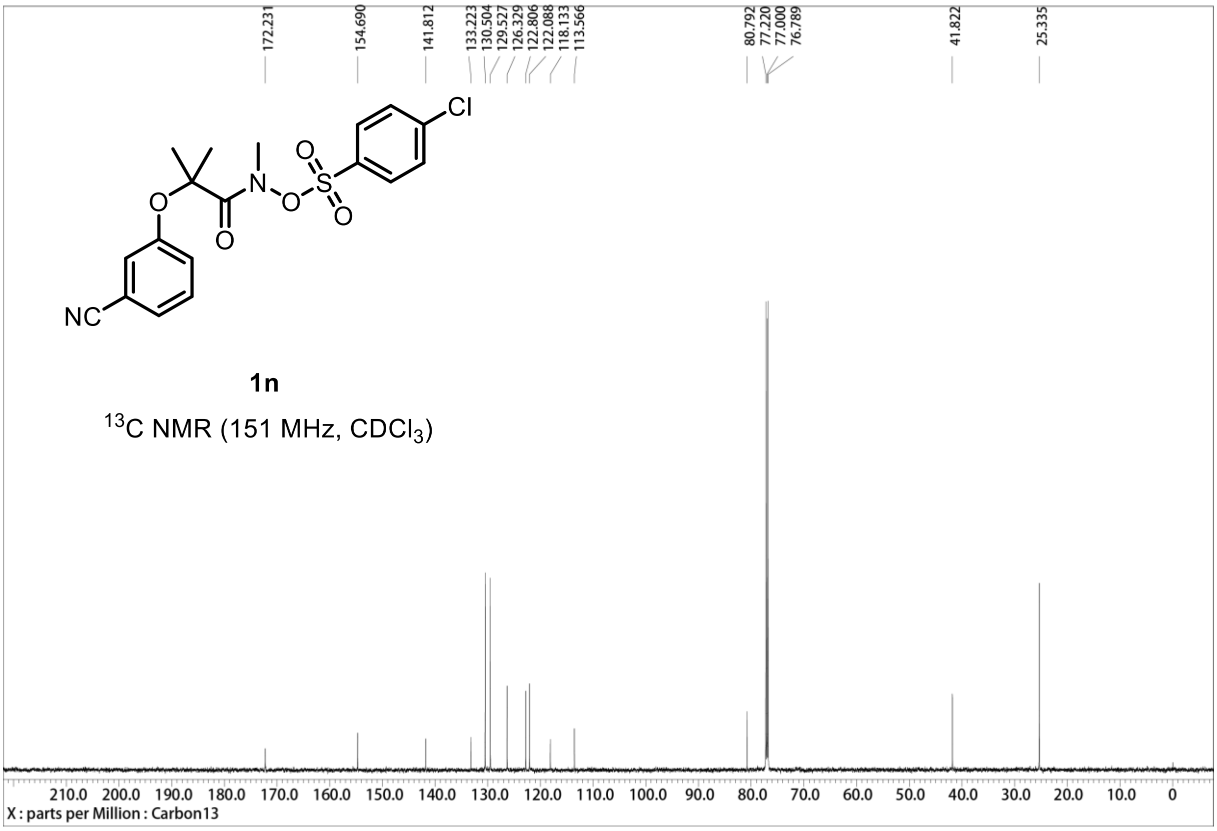

172.231
154.690
141.812
133.223
130.504
129.527
126.329
122.806
122.088
118.133
113.566
80.792
77.220
77.000
76.789
41.822
25.335
Cl
O
S
N
O
O
O
O
NC
1n
13C NMR (151 MHz, CDCl3)
210.0
200.0
190.0
180.0
170.0
160.0
150.0
140.0
130.0
120.0
110.0
100.0
90.0
80.0
70.0
60.0
50.0
40.0
30.0
20.0
10.0
0
X : parts per Million : Carbon13

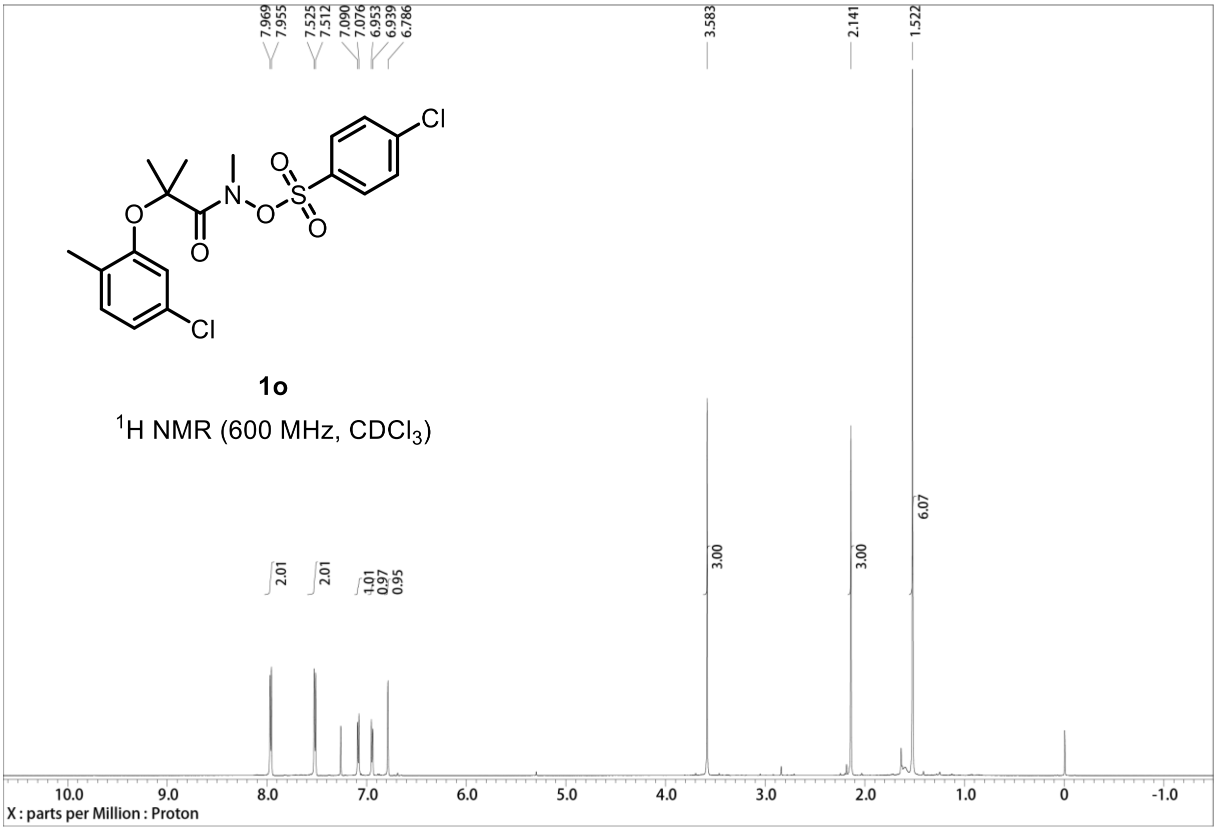
7.969
7.955
7.525
7.512
7.090
7.076
6.953
6.939
6.786
3.583
2.141
1.522
Cl
O
O
N
O
S
O
O
O
Cl
1o
^{1}H NMR (600 MHz, CDCl$_3$)
2.01
2.01
1.01
0.97
0.95
3.00
3.00
6.07
10.0 9.0 8.0 7.0 6.0 5.0 4.0 3.0 2.0 1.0 0 -1.0
X : parts per Million : Proton

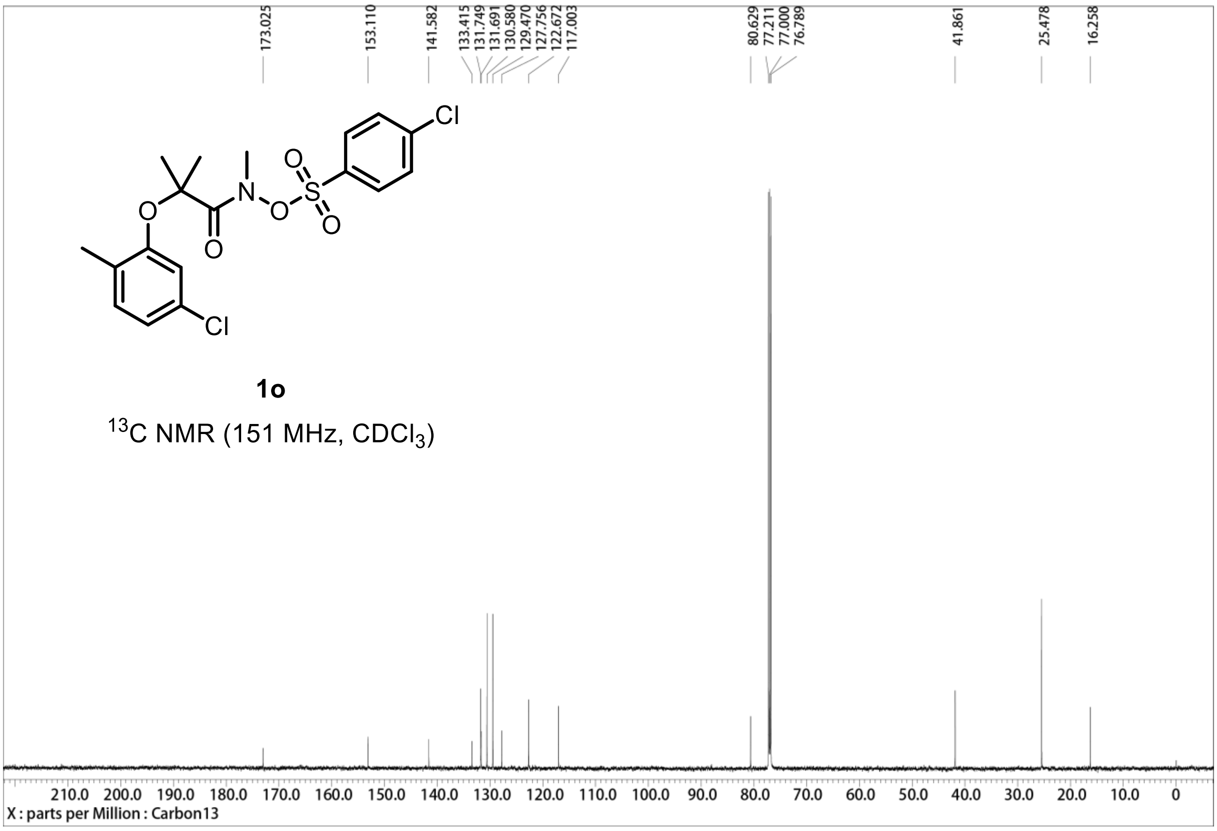
173.025
153.110
141.582
133.415
131.749
131.691
130.580
129.470
127.756
122.672
117.003
80.629
77.211
77.000
76.789
41.861
25.478
16.258
Cl
O
O
N
O
S
O
O
O
Cl
1o
^{13}C NMR (151 MHz, CDCl$_3$)
210.0 200.0 190.0 180.0 170.0 160.0 150.0 140.0 130.0 120.0 110.0 100.0 90.0 80.0 70.0 60.0 50.0 40.0 30.0 20.0 10.0 0
X : parts per Million : Carbon13

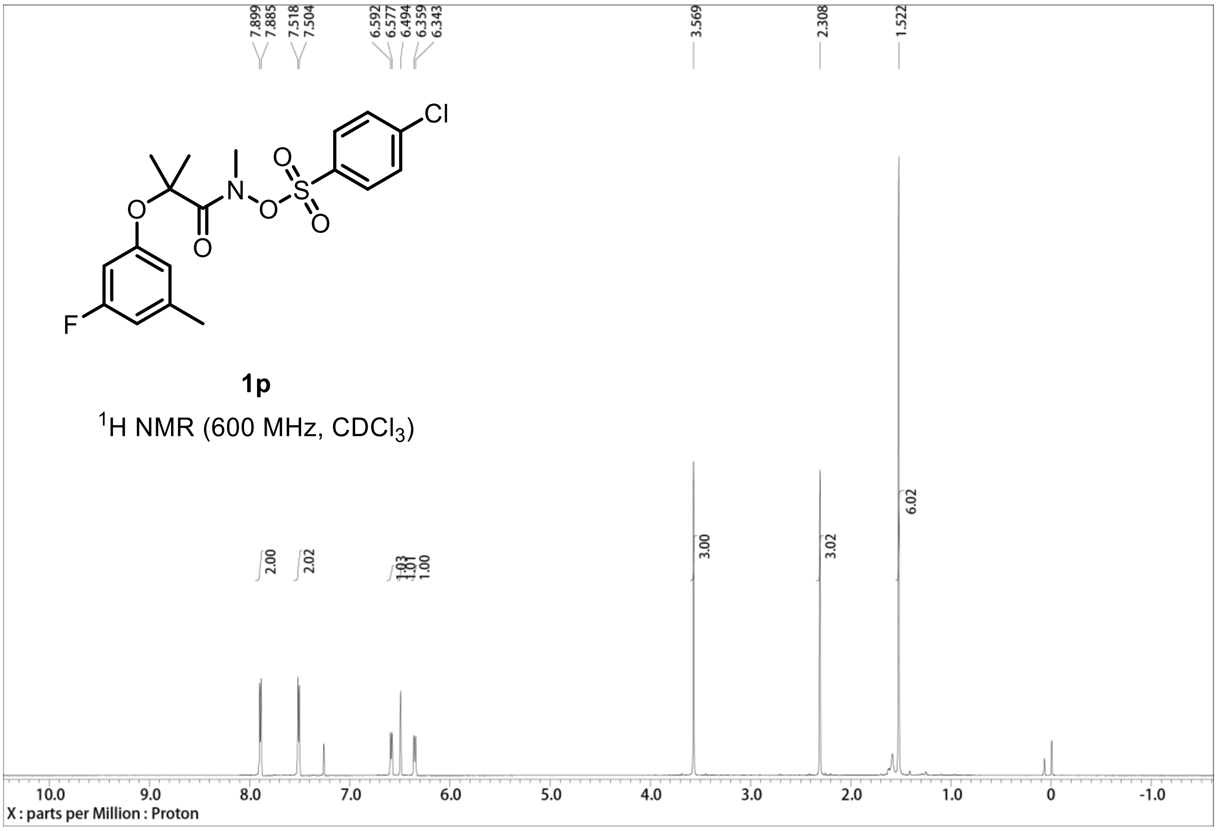
7.899
7.885
7.518
7.504
6.592
6.577
6.494
6.359
6.343
3.569
2.308
1.522
Cl
O
S
O
N
O
O
O
F
1p
1H NMR (600 MHz, CDCl3)
2.00
2.02
1.03
1.01
1.00
3.00
3.02
6.02
10.0
9.0
8.0
7.0
6.0
5.0
4.0
3.0
2.0
1.0
0
-1.0
X : parts per Million : Proton

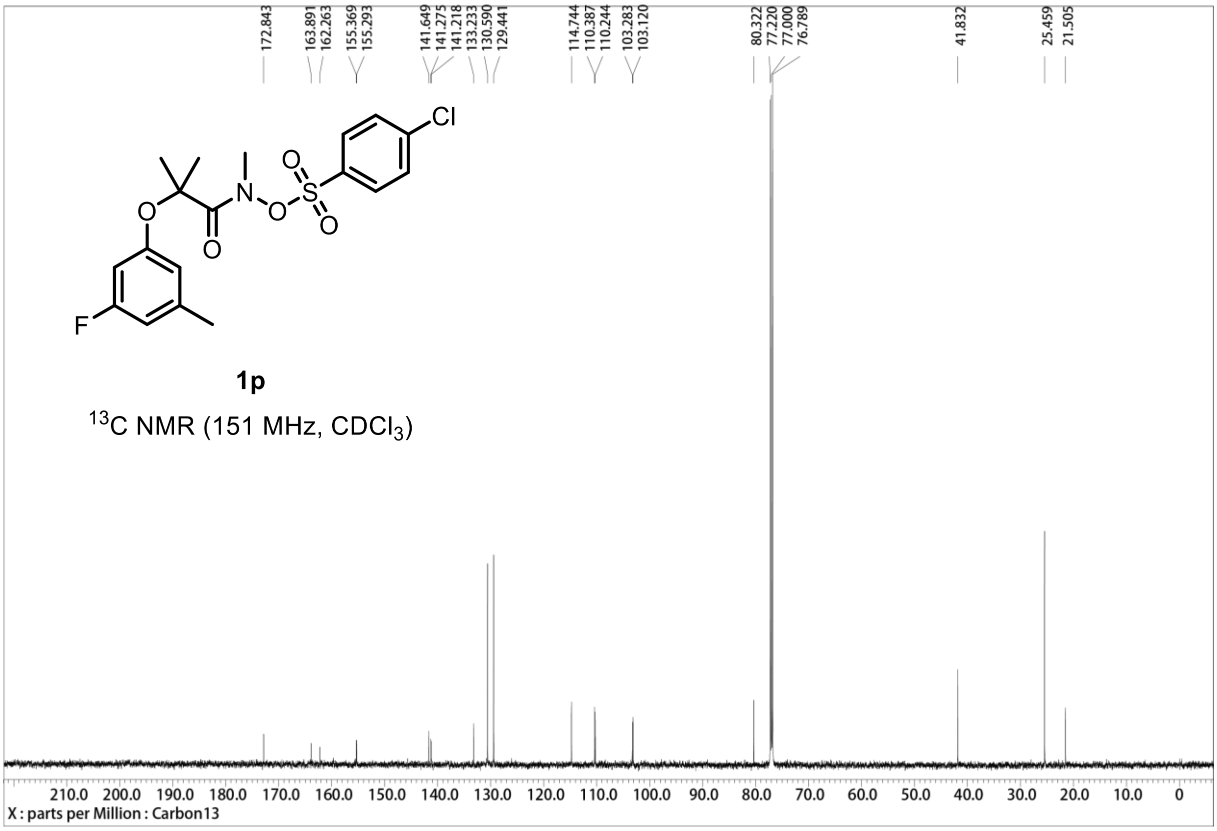
172.843
163.891
162.263
155.369
155.293
141.649
141.275
141.218
133.233
130.590
129.441
114.744
110.387
110.244
103.283
103.120
80.322
77.220
77.000
76.789
41.832
25.459
21.505
Cl
O
S
O
N
O
O
O
F
1p
13C NMR (151 MHz, CDCl3)
210.0
200.0
190.0
180.0
170.0
160.0
150.0
140.0
130.0
120.0
110.0
100.0
90.0
80.0
70.0
60.0
50.0
40.0
30.0
20.0
10.0
0
X : parts per Million : Carbon13

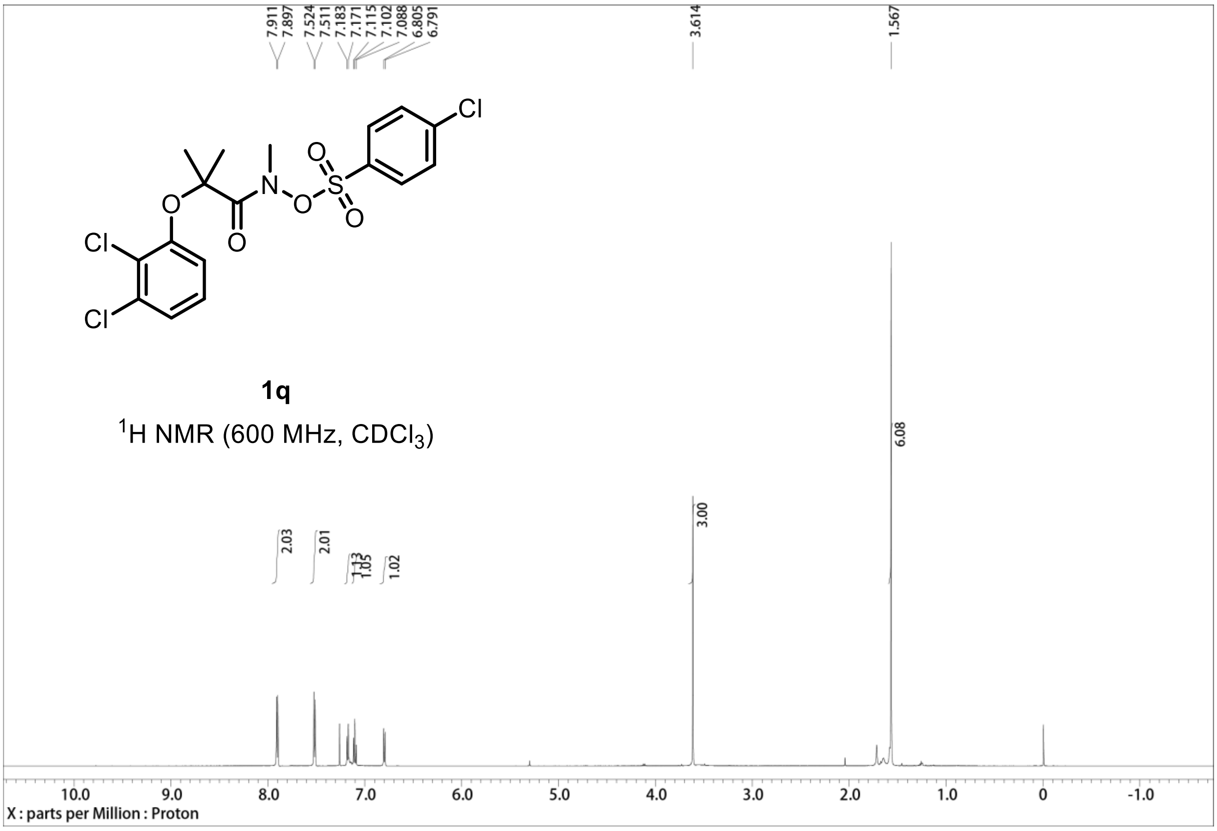
1q
1H NMR (600 MHz, CDCl3)
X : parts per Million : Proton

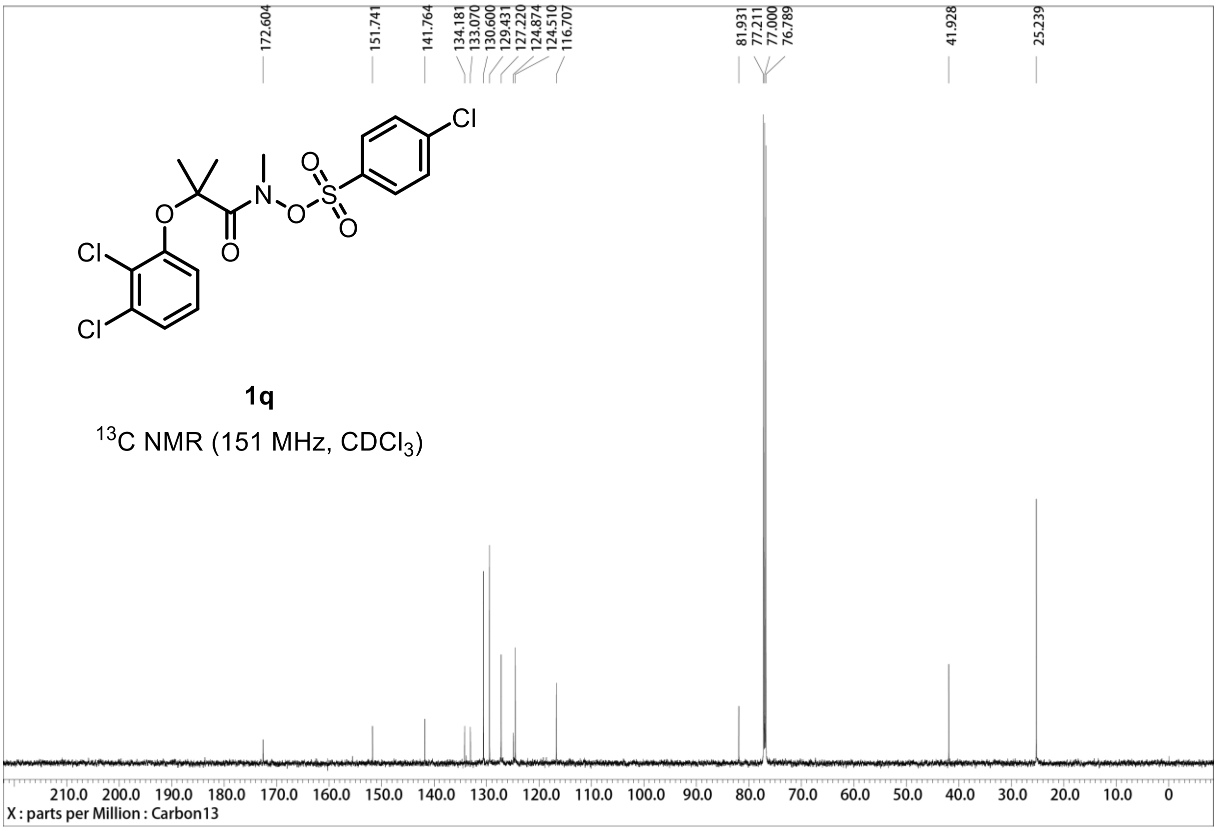
1q
13C NMR (151 MHz, CDCl3)
X : parts per Million : Carbon13

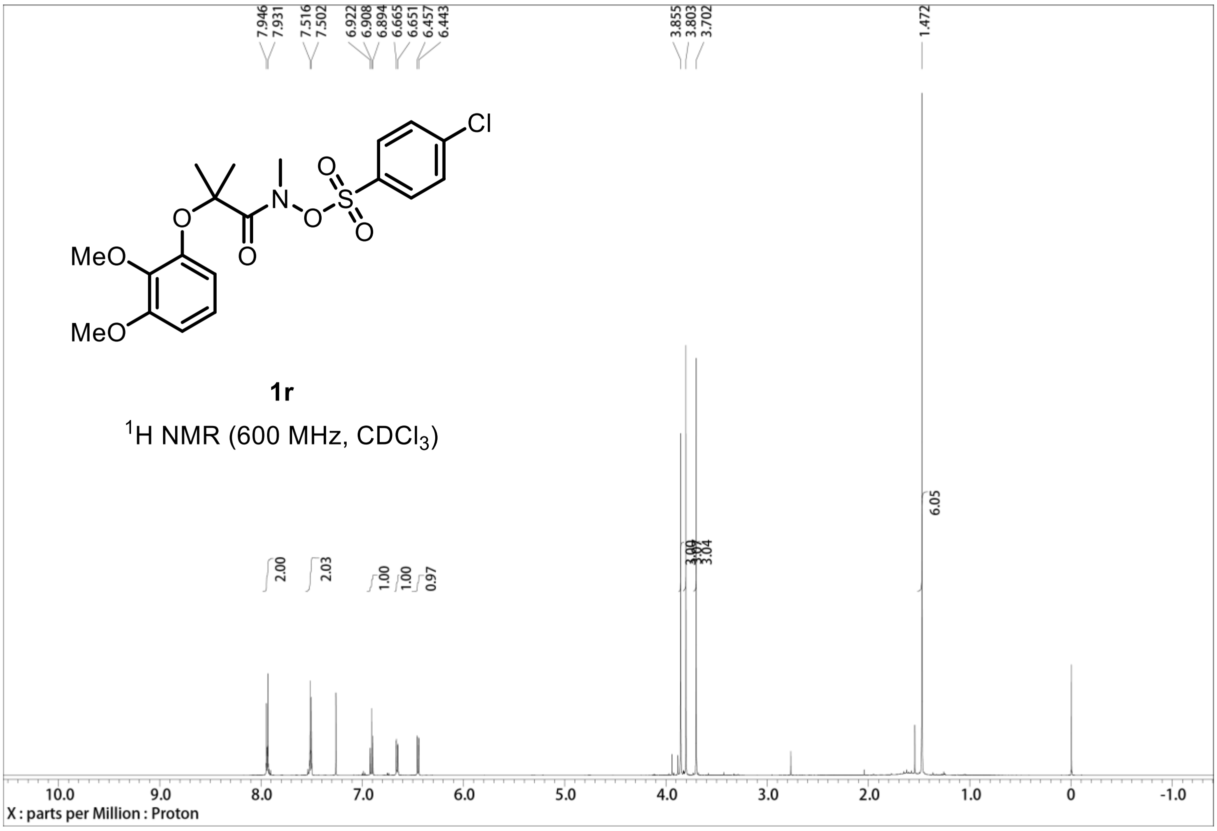
7.946
7.931
7.516
7.502
6.922
6.908
6.894
6.665
6.651
6.457
6.443
3.855
3.803
3.702
1.472
Cl
O
N
O
S
O
O
O
MeO
MeO
1r
1H NMR (600 MHz, CDCl3)
6.05
2.00
2.03
1.00
1.00
0.97
3.04
10.0
9.0
8.0
7.0
6.0
5.0
4.0
3.0
2.0
1.0
0
-1.0
X : parts per Million : Proton

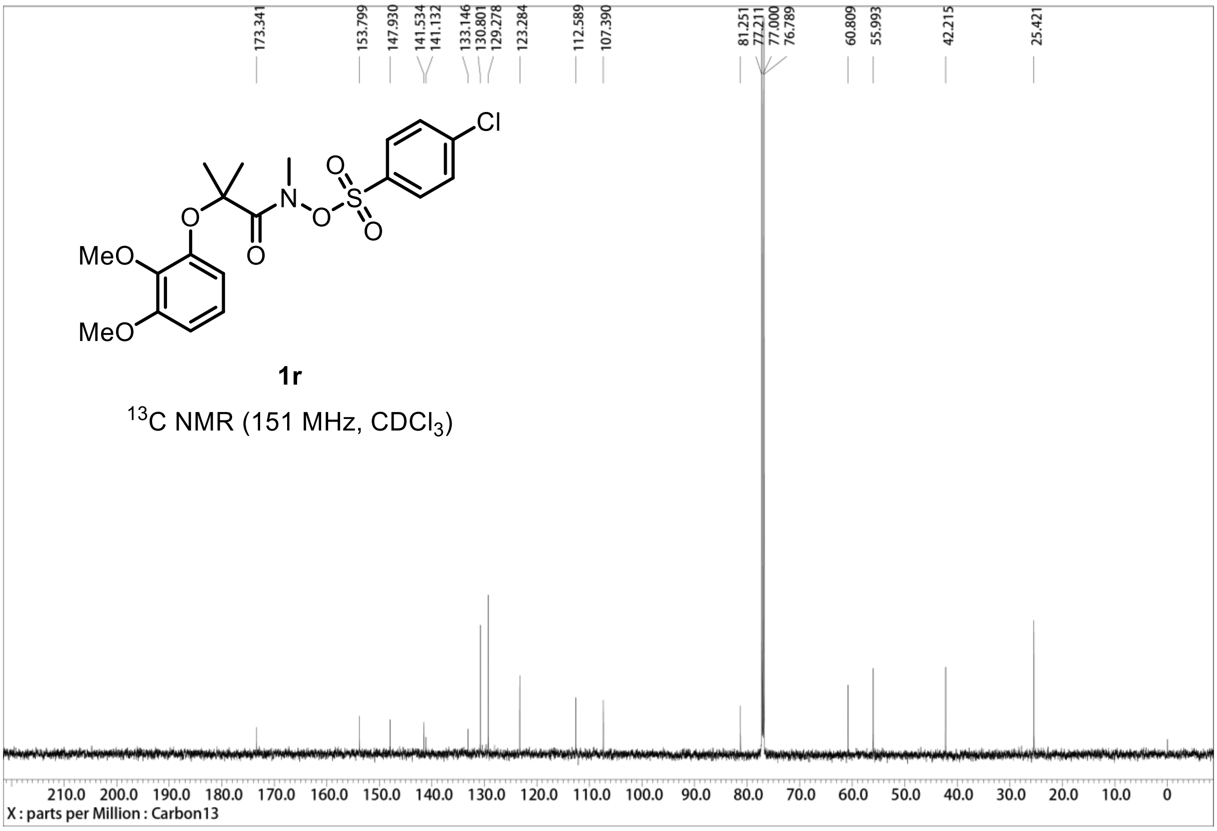
173.341
153.799
147.930
141.534
141.132
133.146
130.801
129.278
123.284
112.589
107.390
81.251
77.211
77.000
76.789
60.809
55.993
42.215
25.421
Cl
O
N
O
S
O
O
O
MeO
MeO
1r
13C NMR (151 MHz, CDCl3)
210.0
200.0
190.0
180.0
170.0
160.0
150.0
140.0
130.0
120.0
110.0
100.0
90.0
80.0
70.0
60.0
50.0
40.0
30.0
20.0
10.0
0
X : parts per Million : Carbon13

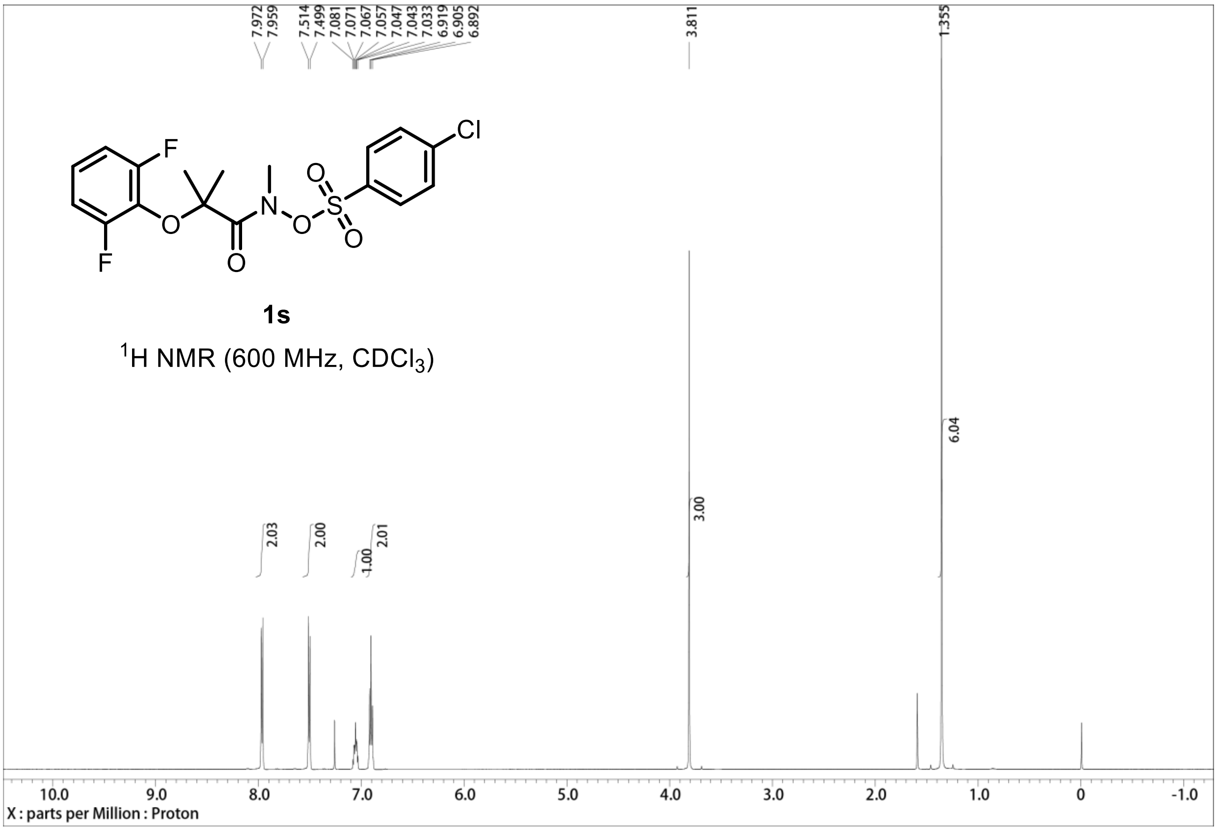

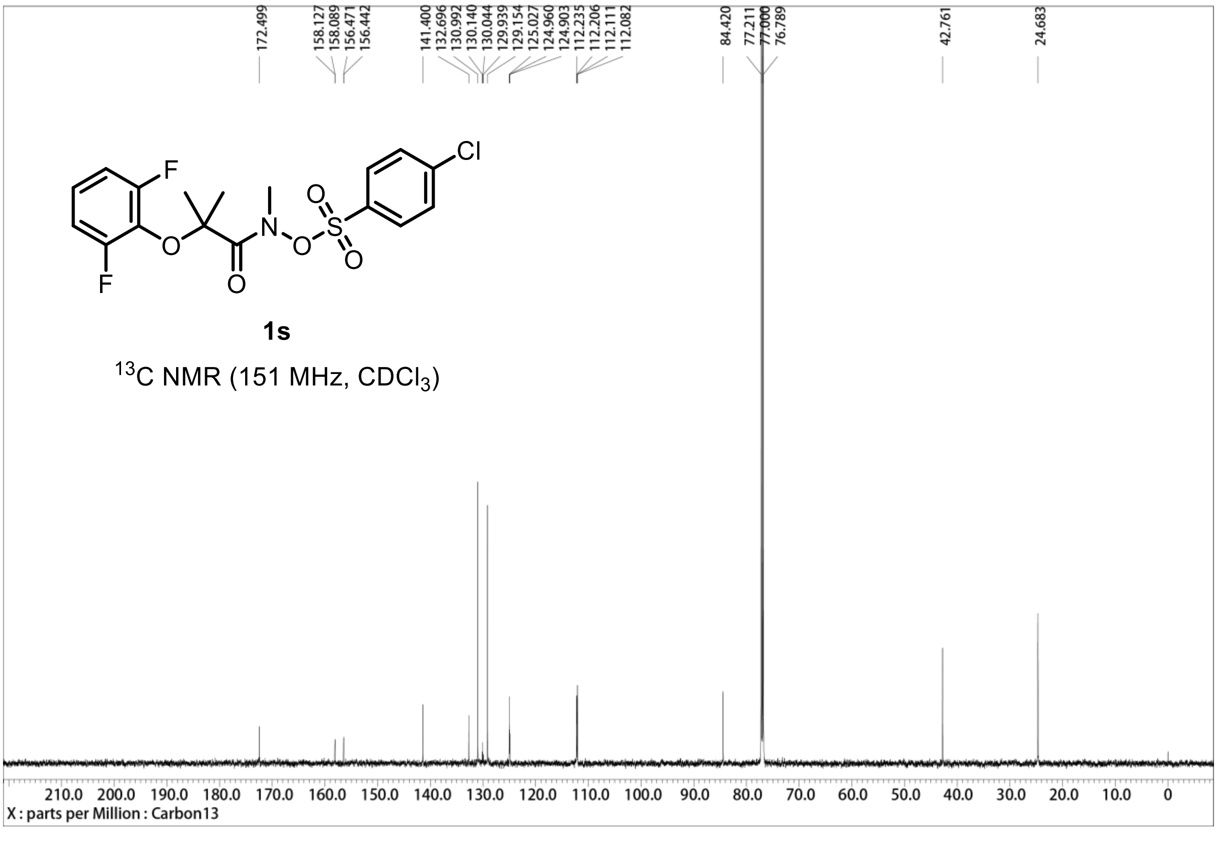

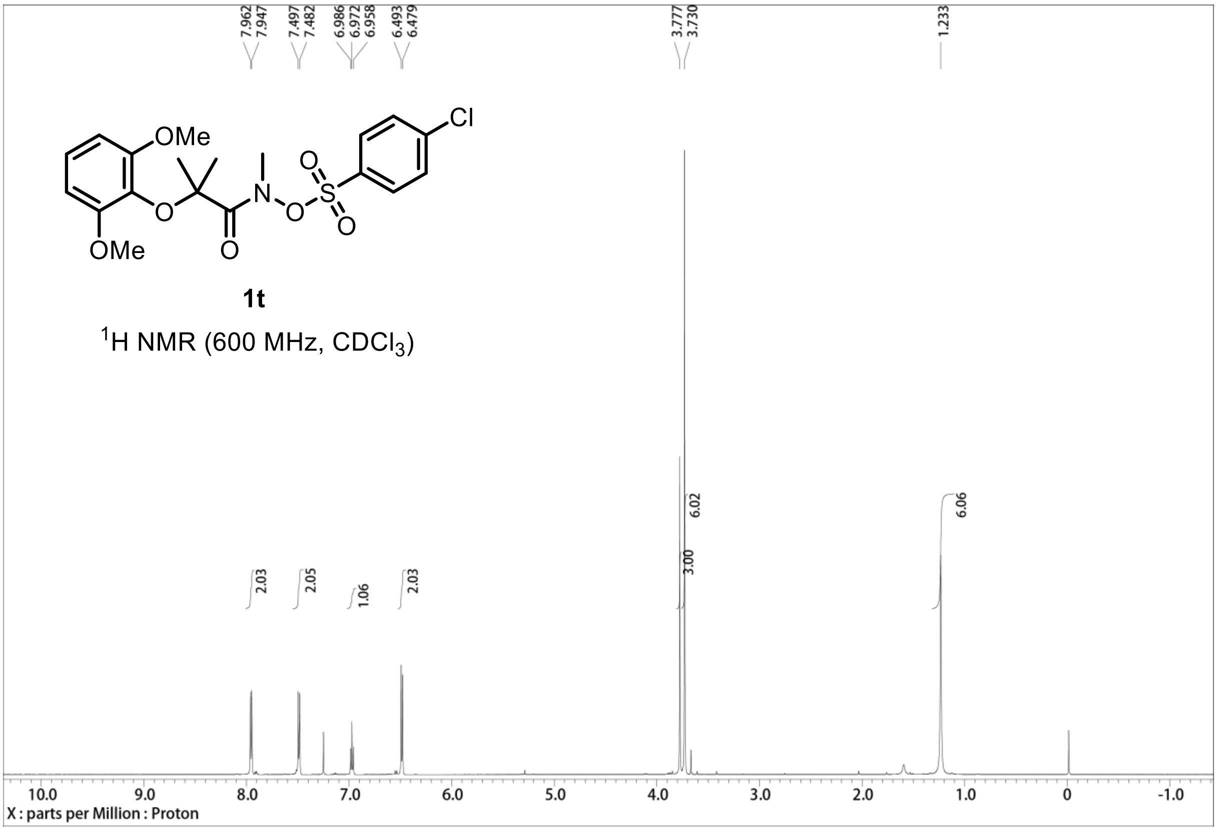
7.962
7.947
7.497
7.482
6.986
6.972
6.958
6.493
6.479
3.777
3.730
1.233
OMe
Cl
O
N
O
S
O
O
OMe
O
1t
1H NMR (600 MHz, CDCl3)
2.03
2.05
1.06
2.03
3.00
6.02
6.06
10.0
9.0
8.0
7.0
6.0
5.0
4.0
3.0
2.0
1.0
0
-1.0
X : parts per Million : Proton

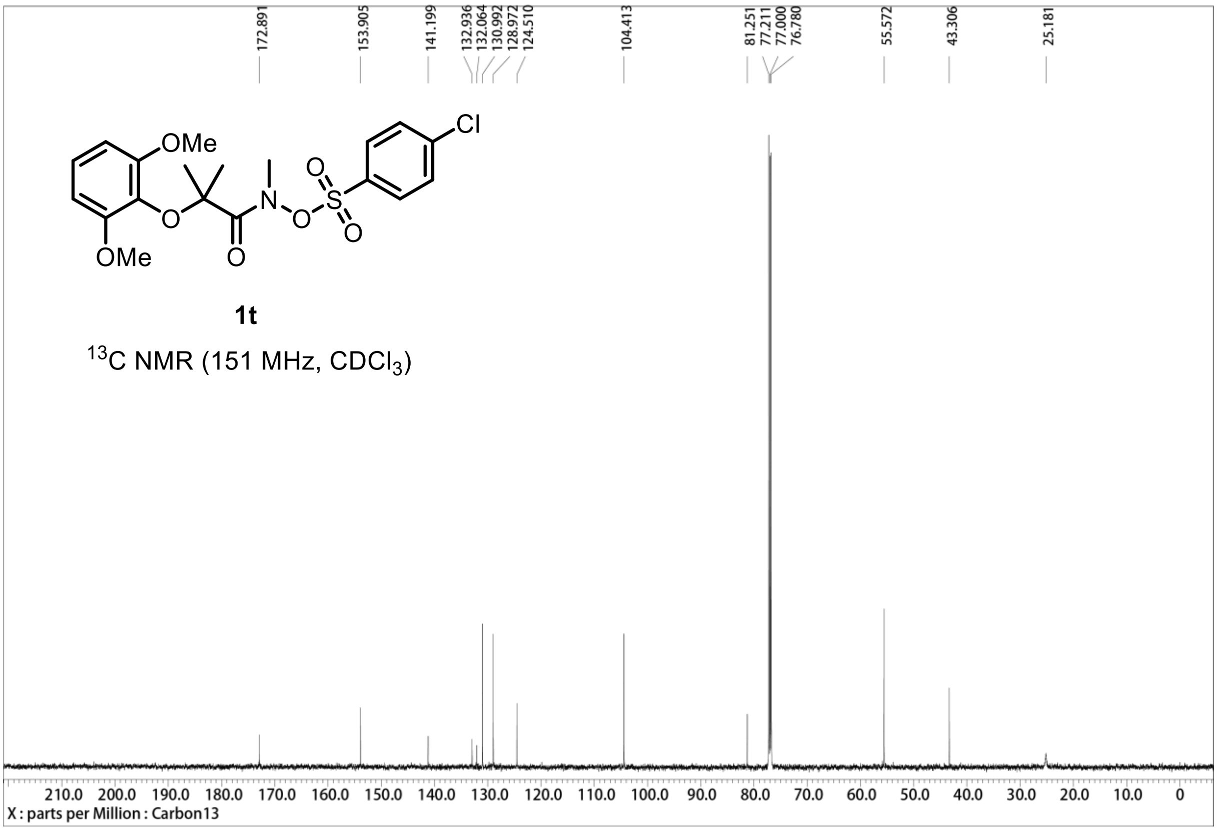
172.891
153.905
141.199
132.936
132.064
130.992
128.972
124.510
104.413
81.251
77.211
77.000
76.780
55.572
43.306
25.181
OMe
Cl
O
N
O
S
O
O
OMe
O
1t
13C NMR (151 MHz, CDCl3)
210.0
200.0
190.0
180.0
170.0
160.0
150.0
140.0
130.0
120.0
110.0
100.0
90.0
80.0
70.0
60.0
50.0
40.0
30.0
20.0
10.0
0
X : parts per Million : Carbon13

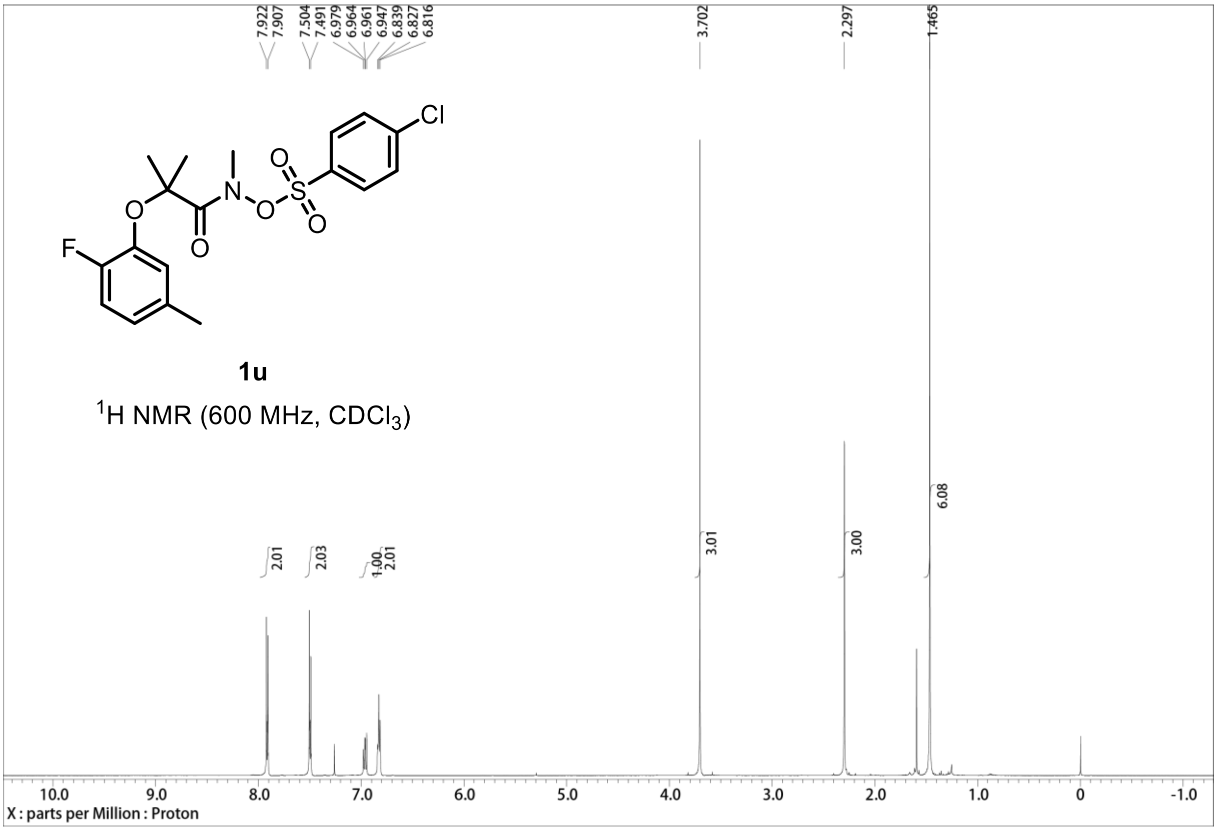

7.922
7.907
7.504
7.491
6.979
6.964
6.961
6.947
6.839
6.827
6.816
3.702
2.297
1.465
Cl
O
S
O
N
O
O
O
F
1u
^{1}H NMR (600 MHz, $CDCl_3$)
2.01
2.03
1.00
2.01
3.01
3.00
6.08
10.0
9.0
8.0
7.0
6.0
5.0
4.0
3.0
2.0
1.0
0
-1.0
X : parts per Million : Proton

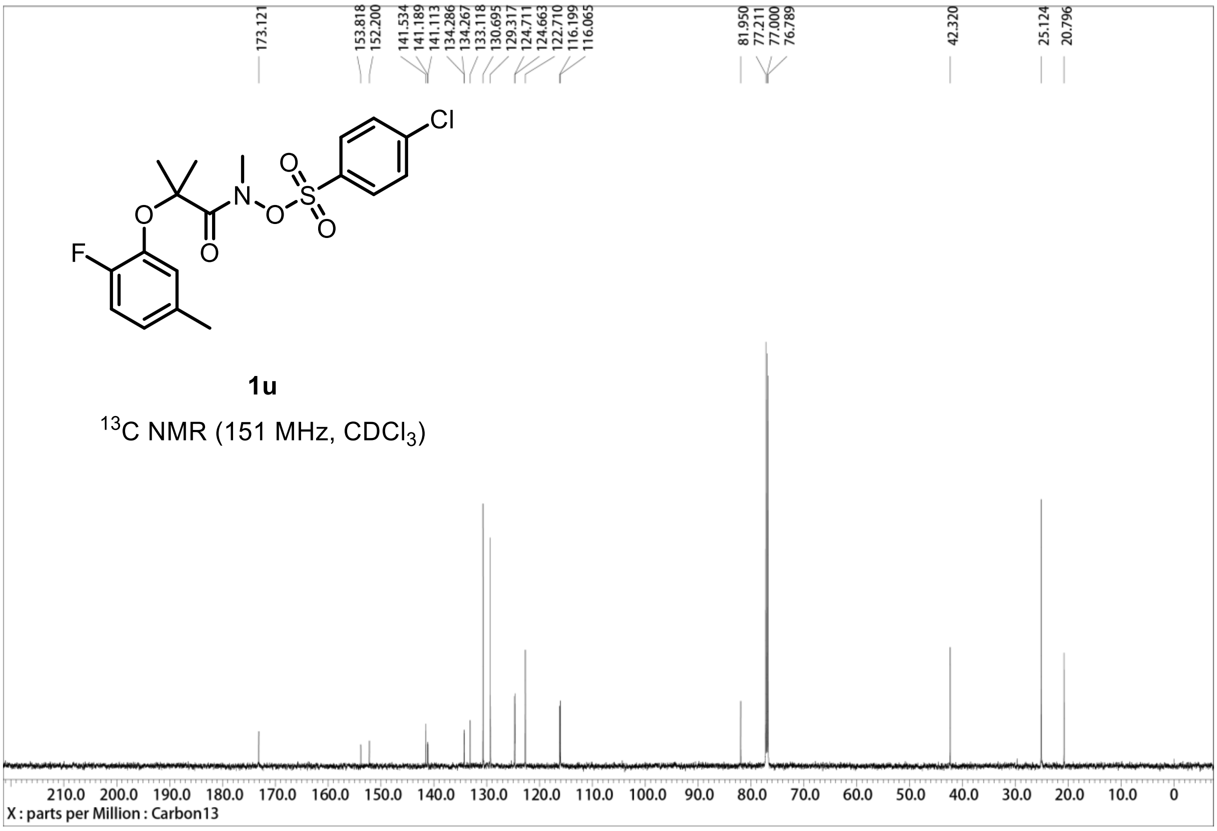

173.121
153.818
152.200
141.534
141.189
141.113
134.286
134.267
133.118
130.695
129.317
124.711
124.663
122.710
116.199
116.065
81.950
77.211
77.000
76.789
42.320
25.124
20.796
Cl
O
S
O
N
O
O
O
F
1u
^{13}C NMR (151 MHz, $CDCl_3$)
210.0
200.0
190.0
180.0
170.0
160.0
150.0
140.0
130.0
120.0
110.0
100.0
90.0
80.0
70.0
60.0
50.0
40.0
30.0
20.0
10.0
0
X : parts per Million : Carbon13

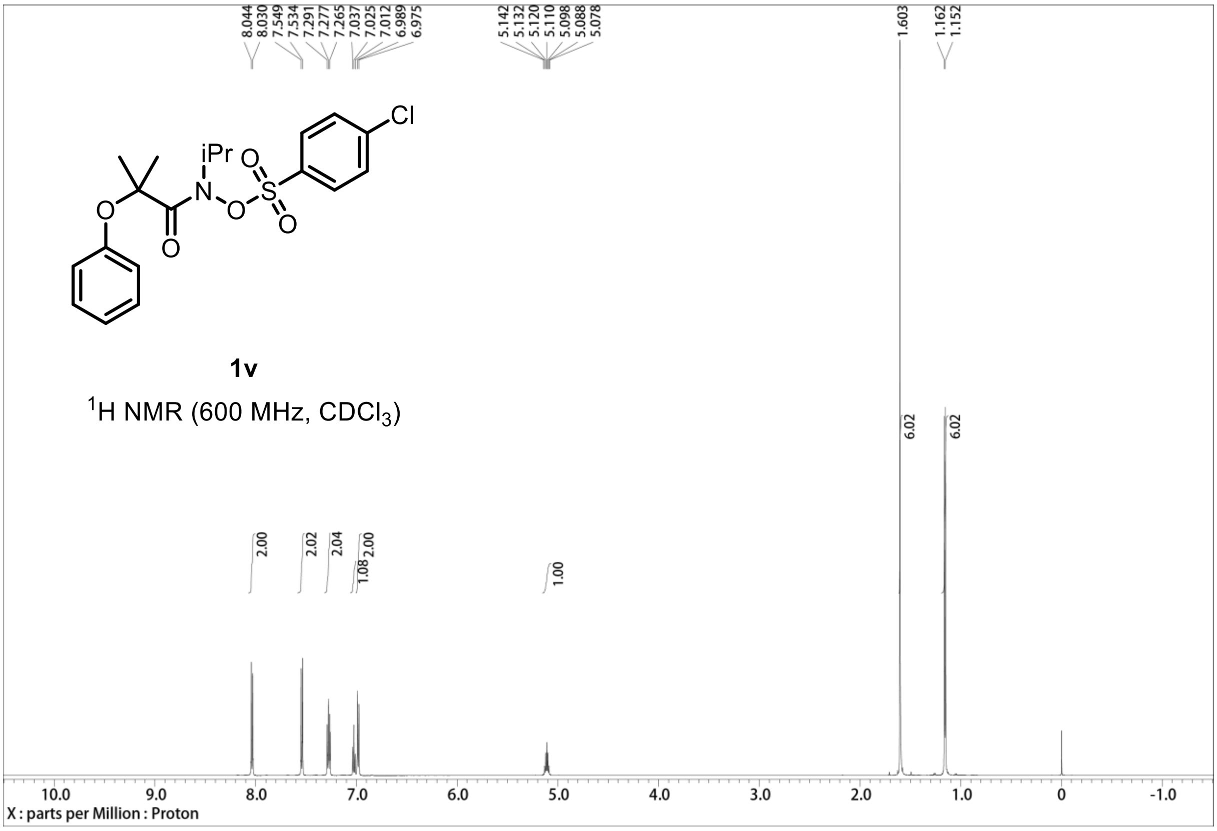
8.044 8.030 7.549 7.534 7.291 7.277 7.265 7.037 7.025 7.012 6.989 6.975
5.142 5.132 5.120 5.110 5.098 5.088 5.078
1.603
1.162 1.152
Cl
iPr
O
N
S
1v
^{1}H NMR (600 MHz, CDCl$_3$)
2.00 2.02 2.04 1.08 2.00
1.00
6.02 6.02
10.0 9.0 8.0 7.0 6.0 5.0 4.0 3.0 2.0 1.0 0 -1.0
X : parts per Million : Proton

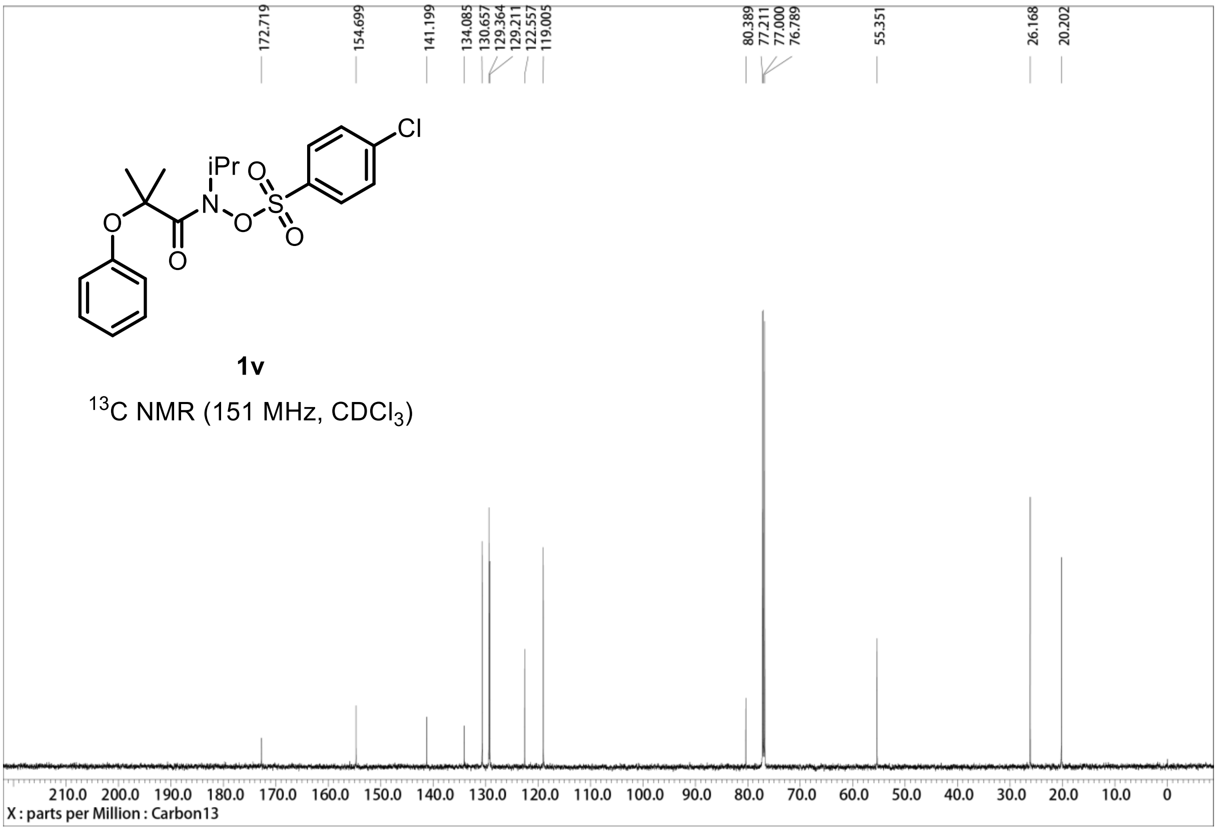
172.719
154.699
141.199
134.085 130.657 129.364 129.211 122.557 119.005
80.389 77.211 77.000 76.789
55.351
26.168 20.202
Cl
iPr
O
N
S
1v
^{13}C NMR (151 MHz, CDCl$_3$)
210.0 200.0 190.0 180.0 170.0 160.0 150.0 140.0 130.0 120.0 110.0 100.0 90.0 80.0 70.0 60.0 50.0 40.0 30.0 20.0 10.0 0
X : parts per Million : Carbon13

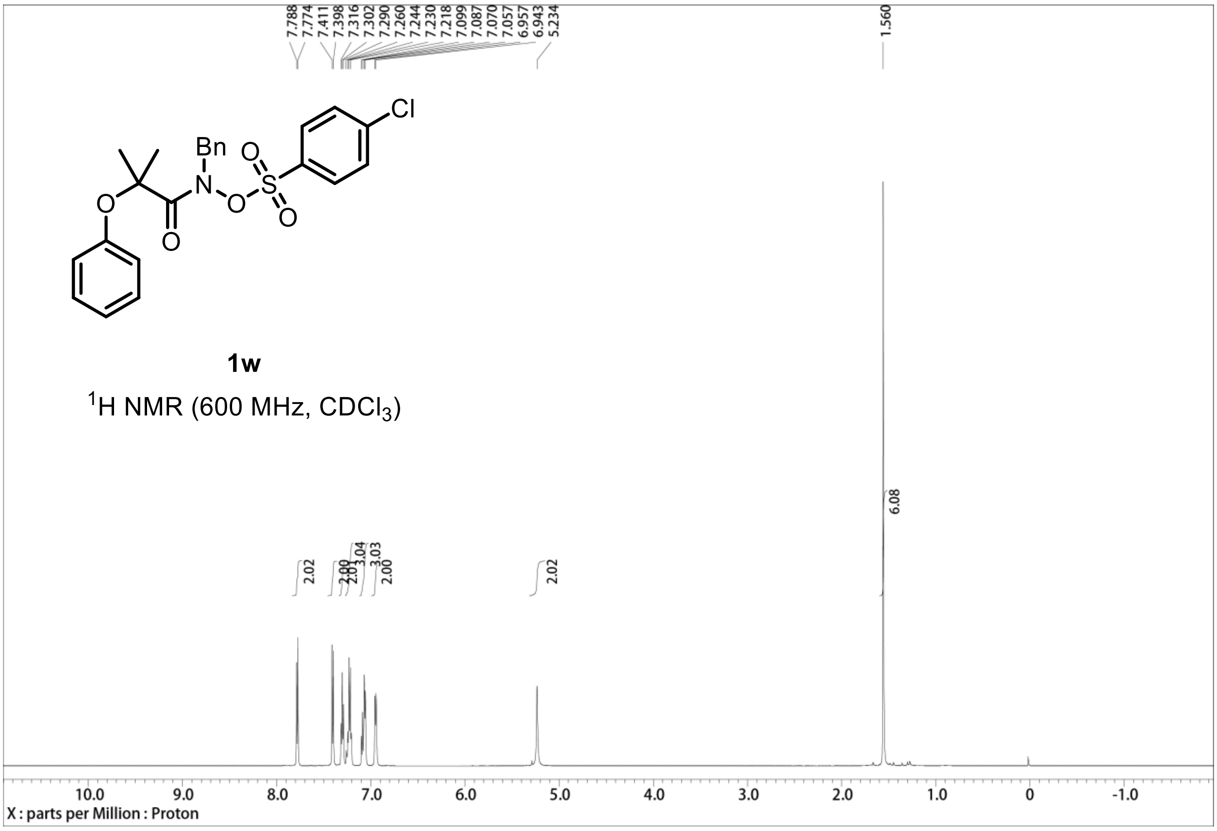
7.788
7.774
7.411
7.398
7.316
7.302
7.290
7.260
7.244
7.230
7.218
7.099
7.087
7.070
7.057
6.957
6.943
5.234
1.560
Bn
Cl
1w
^{1}H NMR (600 MHz, CDCl$_3$)
6.08
2.02
2.00
2.01
3.04
3.03
2.00
2.02
10.0
9.0
8.0
7.0
6.0
5.0
4.0
3.0
2.0
1.0
0
-1.0
X : parts per Million : Proton

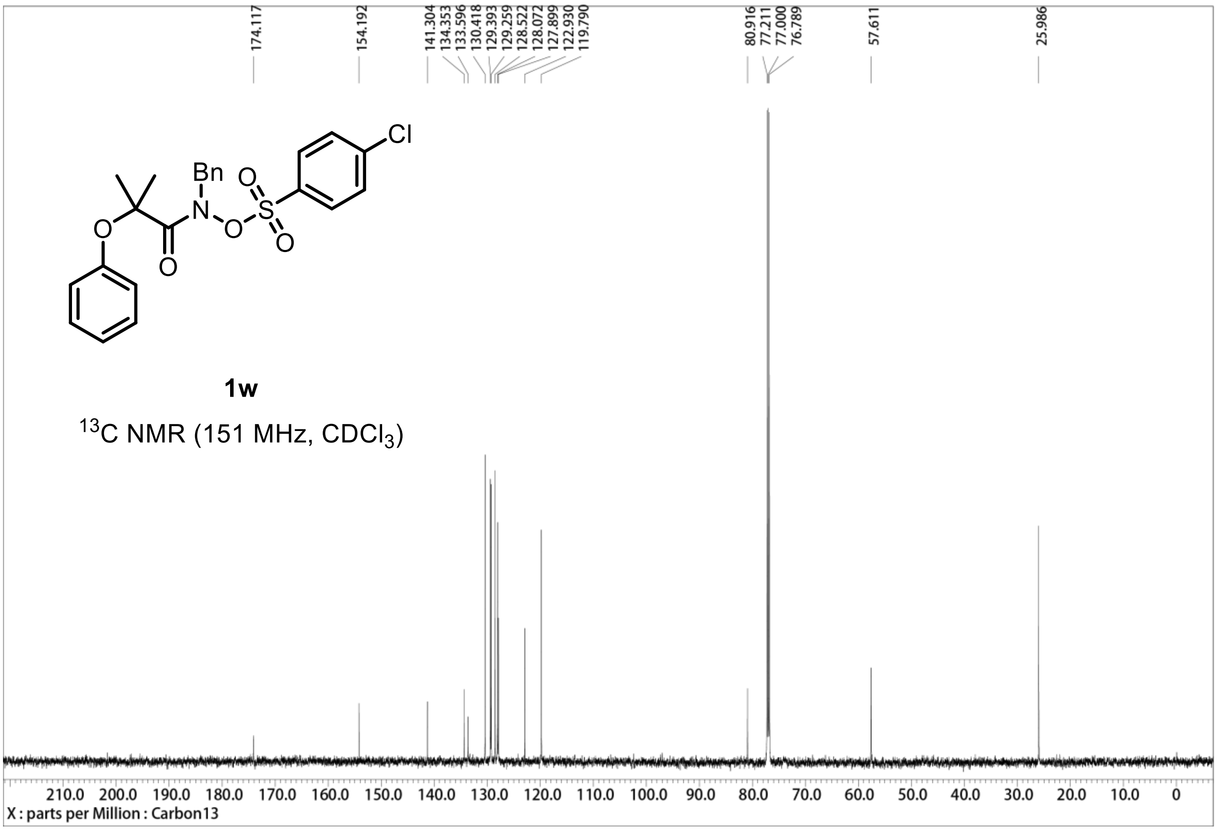
174.117
154.192
141.304
134.353
133.596
130.418
129.393
129.259
128.522
128.072
127.899
122.930
119.790
80.916
77.211
77.000
76.789
57.611
25.986
Bn
Cl
1w
^{13}C NMR (151 MHz, CDCl$_3$)
210.0
200.0
190.0
180.0
170.0
160.0
150.0
140.0
130.0
120.0
110.0
100.0
90.0
80.0
70.0
60.0
50.0
40.0
30.0
20.0
10.0
0
X : parts per Million : Carbon13

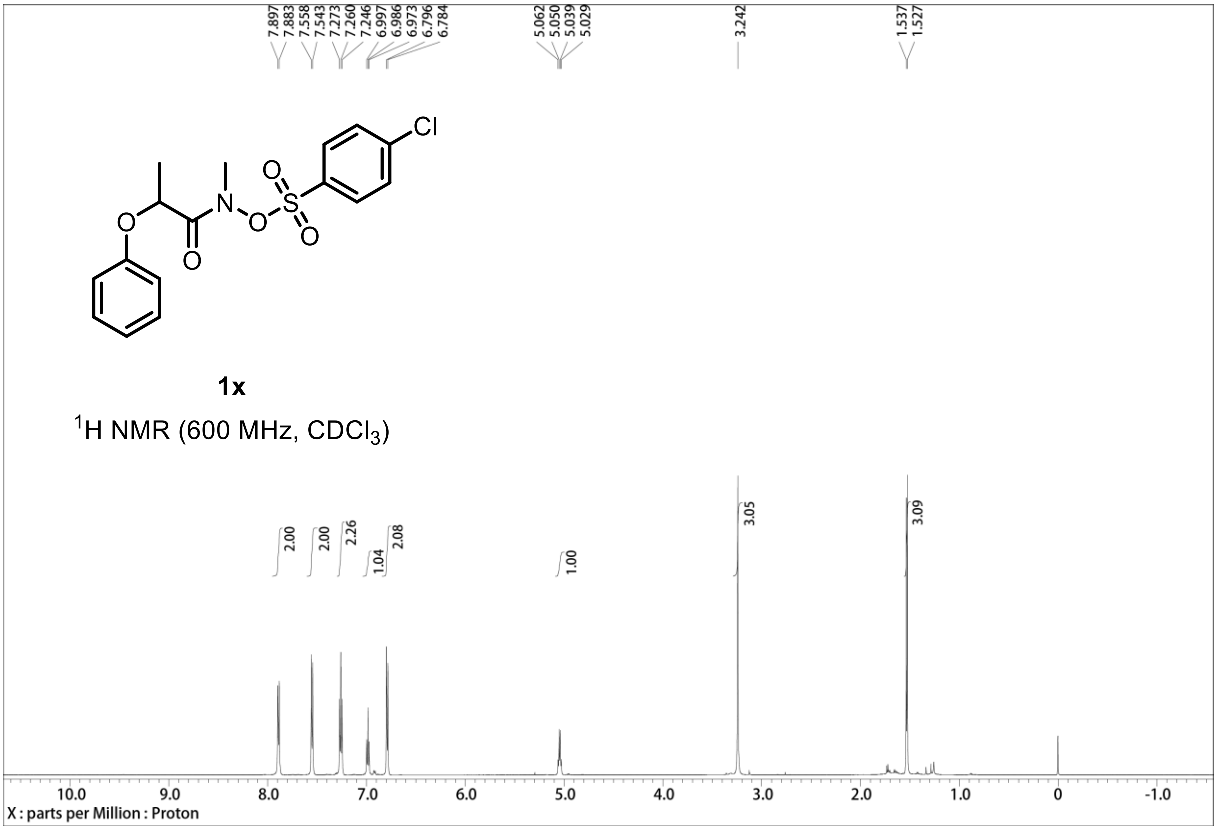

7.897
7.883
7.558
7.543
7.273
7.260
7.246
6.997
6.986
6.973
6.796
6.784
5.062
5.050
5.039
5.029
3.242
1.537
1.527
Cl
O
S
O
N
O
O
O
1x
^{1}H NMR (600 MHz, CDCl$_3$)
2.00
2.00
2.26
1.04
2.08
1.00
3.05
3.09
10.0
9.0
8.0
7.0
6.0
5.0
4.0
3.0
2.0
1.0
0
-1.0
X : parts per Million : Proton

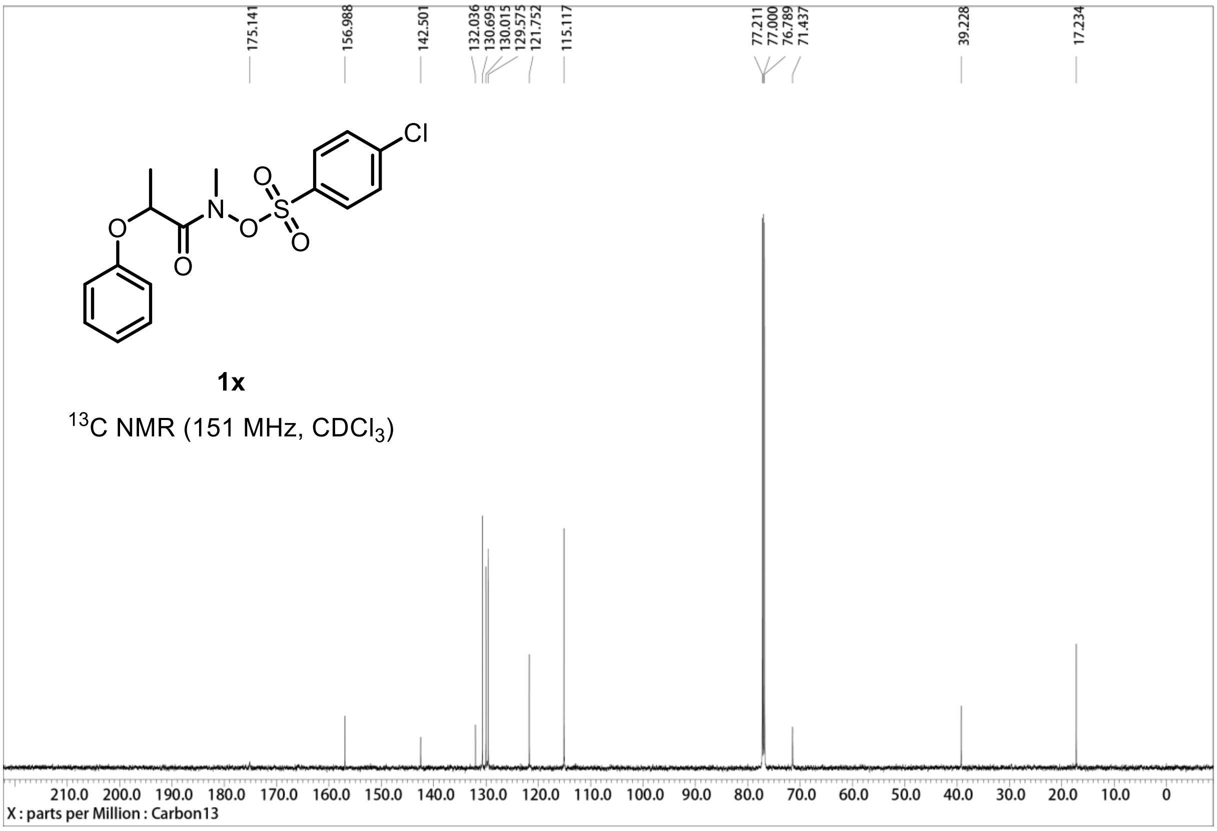

175.141
156.988
142.501
132.036
130.695
130.015
129.575
121.752
115.117
77.211
77.000
76.789
71.437
39.228
17.234
Cl
O
S
O
N
O
O
O
1x
^{13}C NMR (151 MHz, CDCl$_3$)
210.0
200.0
190.0
180.0
170.0
160.0
150.0
140.0
130.0
120.0
110.0
100.0
90.0
80.0
70.0
60.0
50.0
40.0
30.0
20.0
10.0
0
X : parts per Million : Carbon13

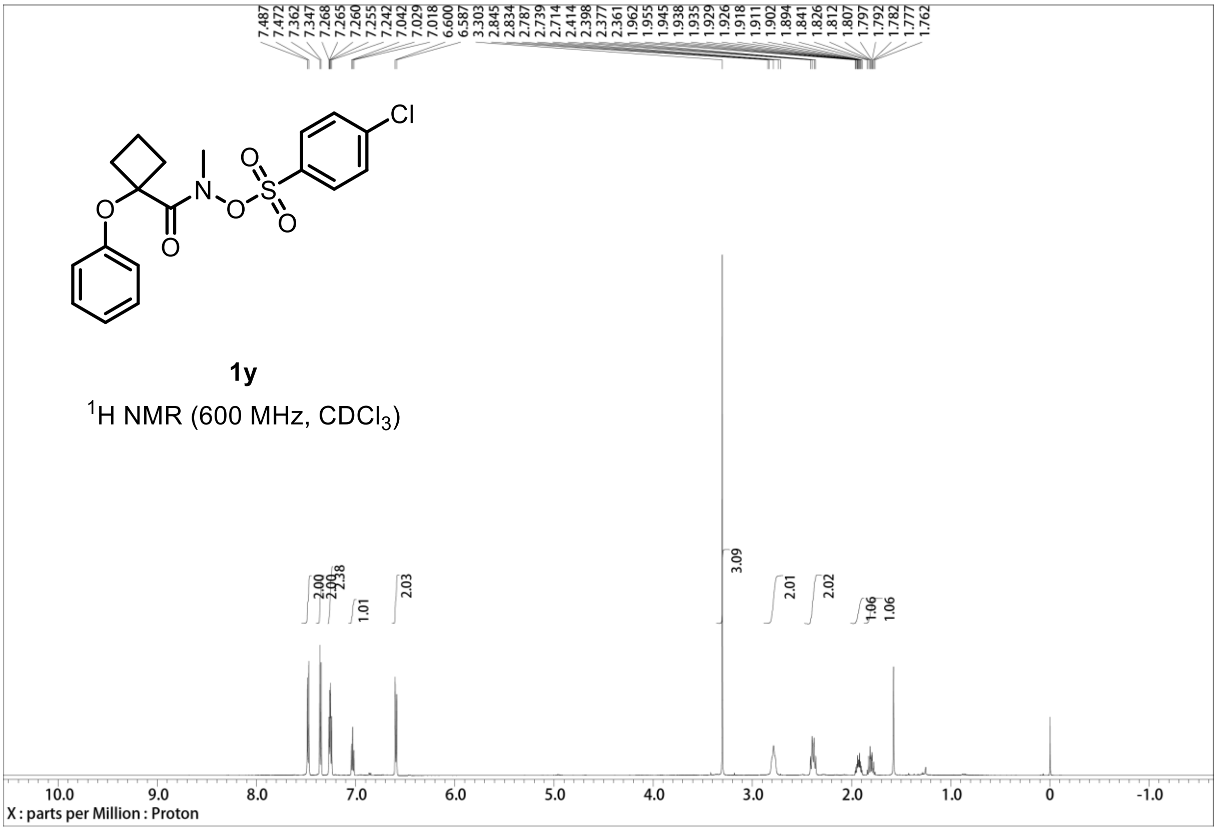
7.487 7.472 7.362 7.347 7.268 7.265 7.260 7.255 7.242 7.042 7.029 7.018 6.600 6.587 3.303 2.845 2.834 2.787 2.739 2.714 2.414 2.398 2.377 2.361 1.962 1.955 1.945 1.938 1.935 1.929 1.926 1.918 1.911 1.902 1.894 1.841 1.826 1.812 1.807 1.797 1.792 1.782 1.777 1.762
Cl
O
N
O
S
O
O
O
1y
1H NMR (600 MHz, CDCl3)
2.00 2.00 2.38 1.01 2.03 3.09 2.01 2.02 1.06 1.06
10.0 9.0 8.0 7.0 6.0 5.0 4.0 3.0 2.0 1.0 0 -1.0
X : parts per Million : Proton

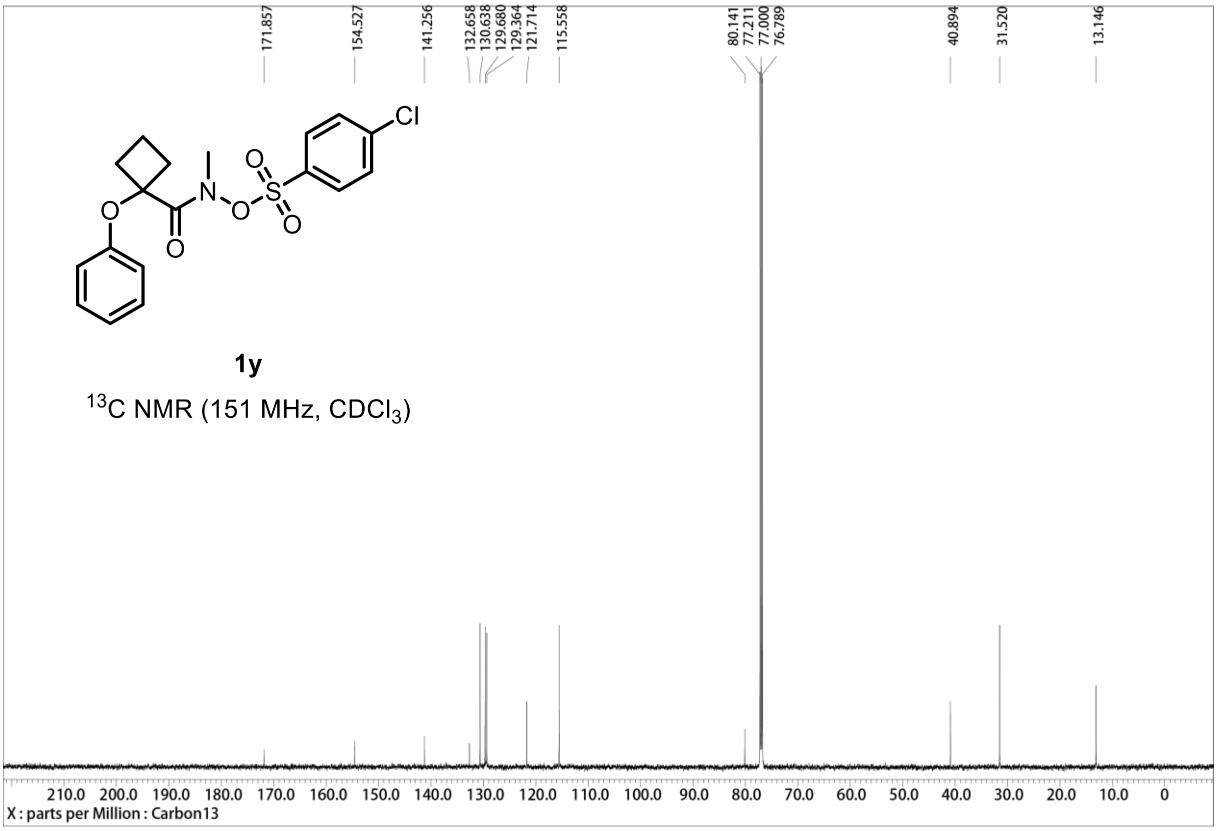
171.857 154.527 141.256 132.658 130.638 129.680 129.364 121.714 115.558 80.141 77.211 77.000 76.789 40.894 31.520 13.146
Cl
O
N
O
S
O
O
O
1y
13C NMR (151 MHz, CDCl3)
210.0 200.0 190.0 180.0 170.0 160.0 150.0 140.0 130.0 120.0 110.0 100.0 90.0 80.0 70.0 60.0 50.0 40.0 30.0 20.0 10.0 0
X : parts per Million : Carbon13

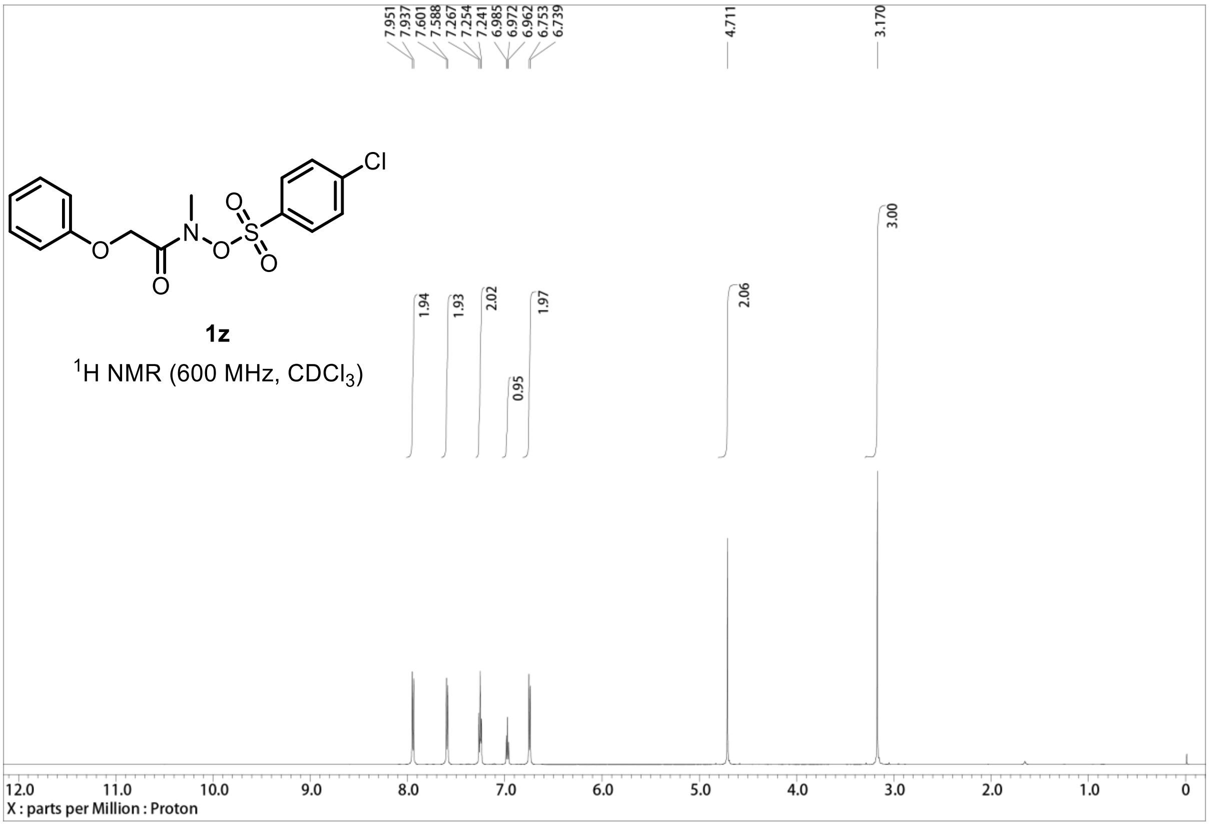

7.951
7.937
7.601
7.588
7.267
7.254
7.241
6.985
6.972
6.962
6.753
6.739
4.711
3.170
Cl
O
N
O
S
O
O
O
O
1z
^{1}H NMR (600 MHz, CDCl$_3$)
1.94
1.93
2.02
0.95
1.97
2.06
3.00
12.0
11.0
10.0
9.0
8.0
7.0
6.0
5.0
4.0
3.0
2.0
1.0
0
X : parts per Million : Proton

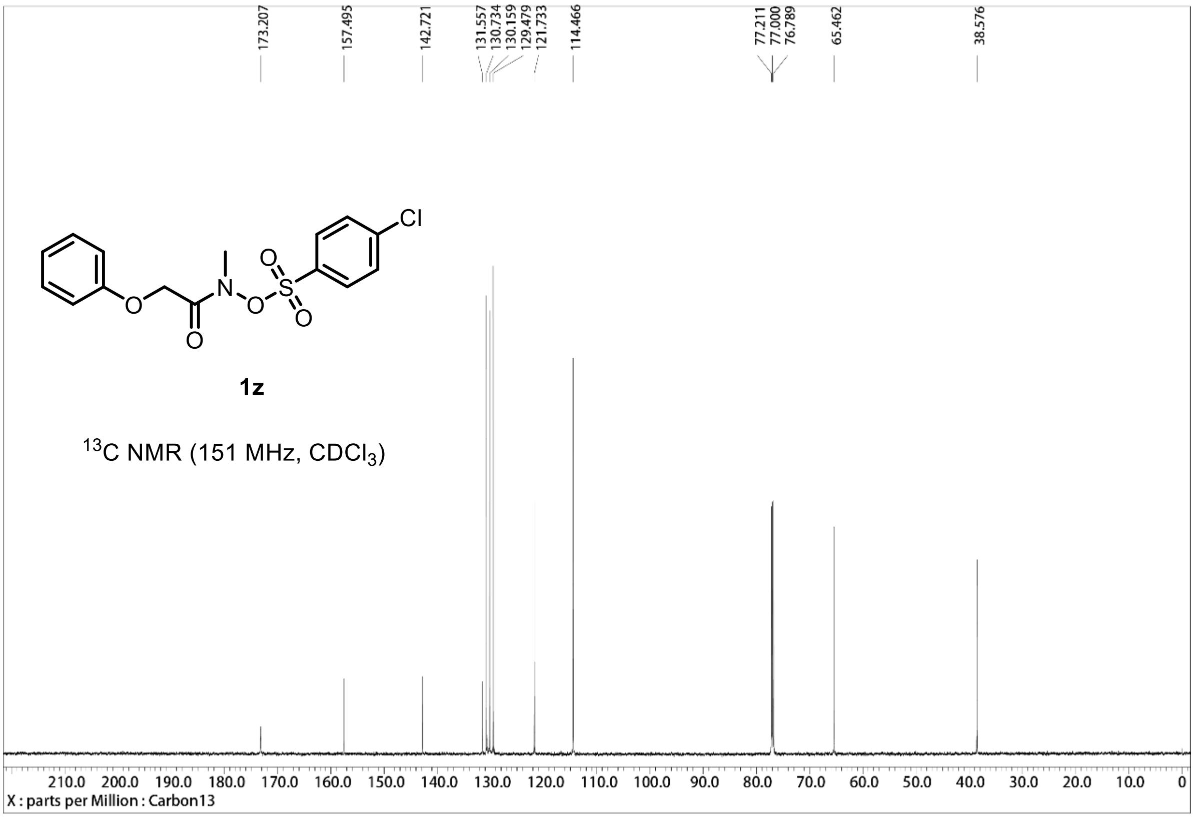

173.207
157.495
142.721
131.557
130.734
130.159
129.479
121.733
114.466
77.211
77.000
76.789
65.462
38.576
Cl
O
N
O
S
O
O
O
O
1z
^{13}C NMR (151 MHz, CDCl$_3$)
210.0
200.0
190.0
180.0
170.0
160.0
150.0
140.0
130.0
120.0
110.0
100.0
90.0
80.0
70.0
60.0
50.0
40.0
30.0
20.0
10.0
0
X : parts per Million : Carbon13

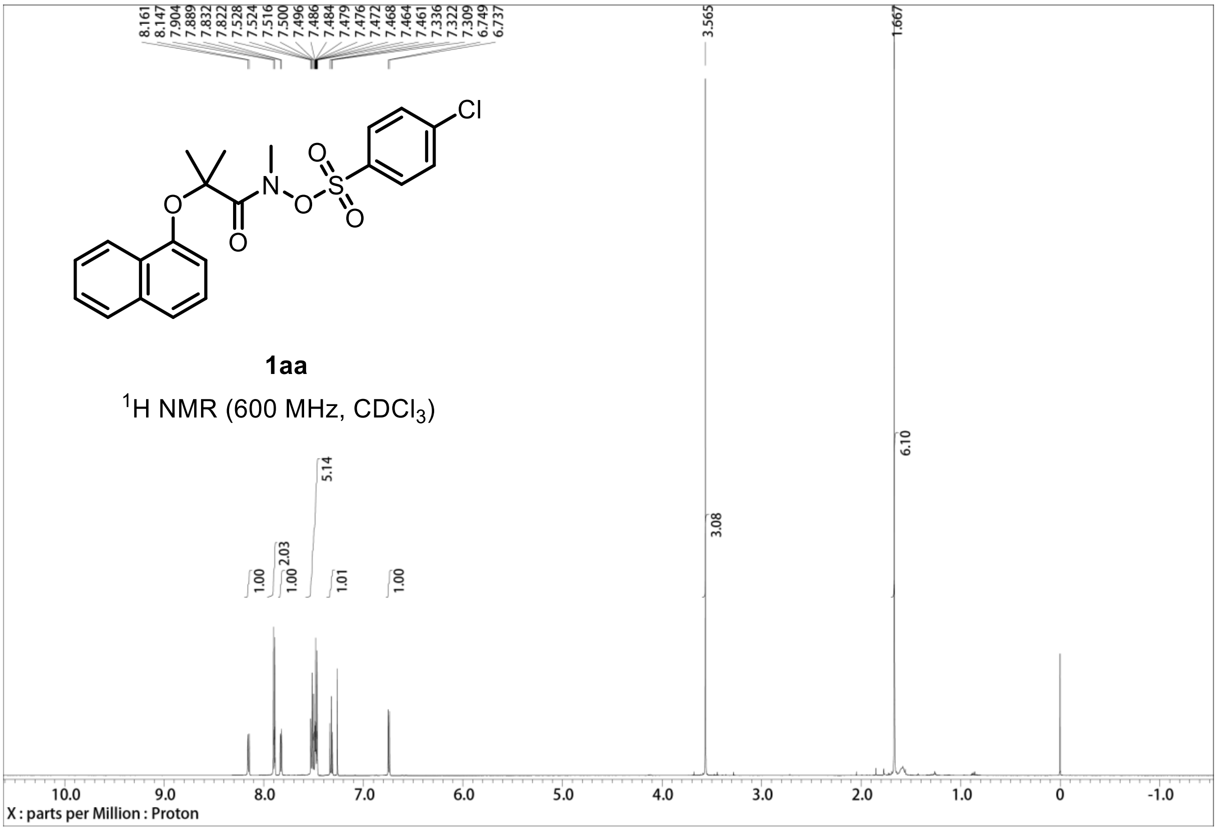
8.161
8.147
7.904
7.889
7.832
7.822
7.528
7.524
7.516
7.500
7.496
7.486
7.484
7.479
7.476
7.472
7.468
7.464
7.461
7.336
7.322
7.309
6.749
6.737
3.565
1.667
Cl
O
O
S
O
N
O
O
1aa
^{1}H NMR (600 MHz, $CDCl_3$)
1.00
1.00
2.03
5.14
1.01
1.00
3.08
6.10
10.0
9.0
8.0
7.0
6.0
5.0
4.0
3.0
2.0
1.0
0
-1.0
X : parts per Million : Proton

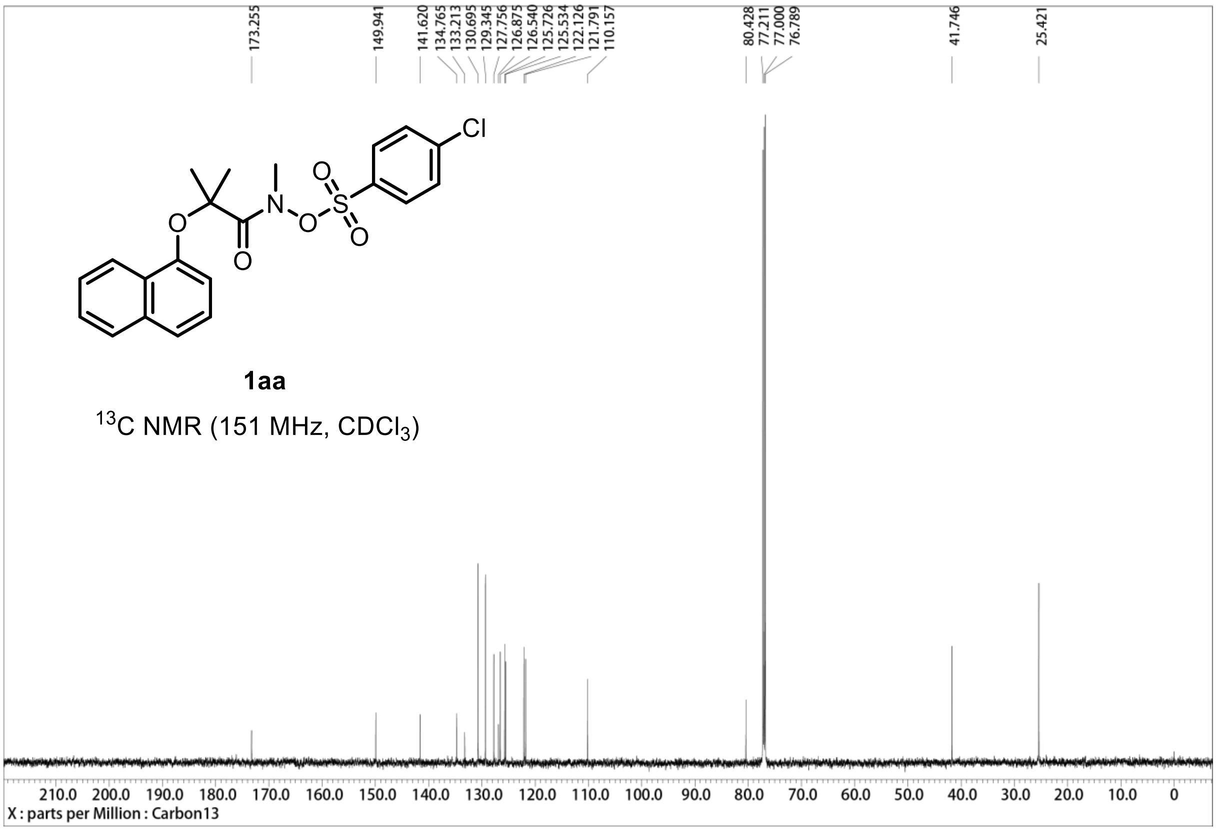
173.255
149.941
141.620
134.765
133.213
130.695
129.345
127.756
126.875
126.540
125.726
125.534
122.126
121.791
110.157
80.428
77.211
77.000
76.789
41.746
25.421
Cl
O
O
S
O
N
O
O
1aa
^{13}C NMR (151 MHz, $CDCl_3$)
210.0
200.0
190.0
180.0
170.0
160.0
150.0
140.0
130.0
120.0
110.0
100.0
90.0
80.0
70.0
60.0
50.0
40.0
30.0
20.0
10.0
0
X : parts per Million : Carbon13

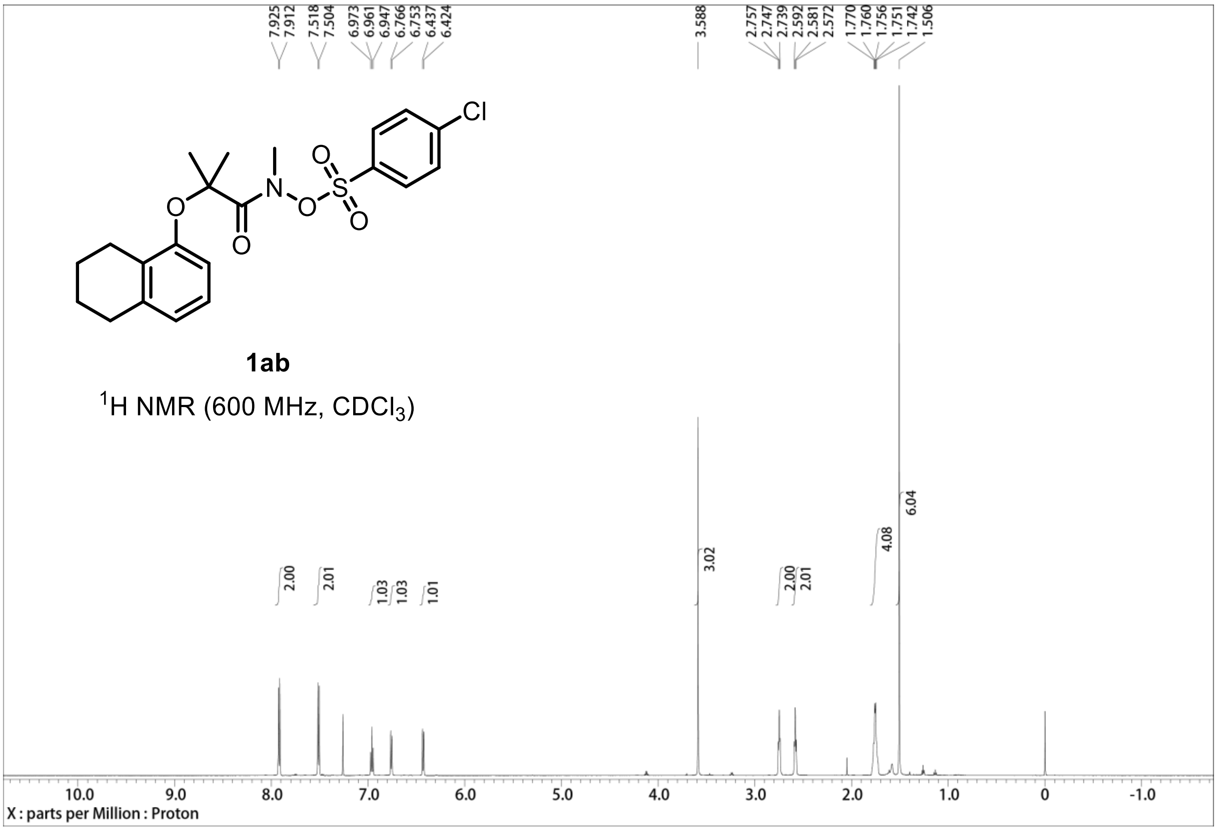
7.925 7.912 7.518 7.504 6.973 6.961 6.947 6.766 6.753 6.437 6.424 3.588 2.757 2.747 2.739 2.592 2.581 2.572 1.770 1.760 1.756 1.751 1.742 1.506
Cl
O
S
N
O
1ab
¹H NMR (600 MHz, CDCl₃)
2.00 2.01 1.03 1.03 1.01 3.02 2.00 2.01 4.08 6.04
X : parts per Million : Proton

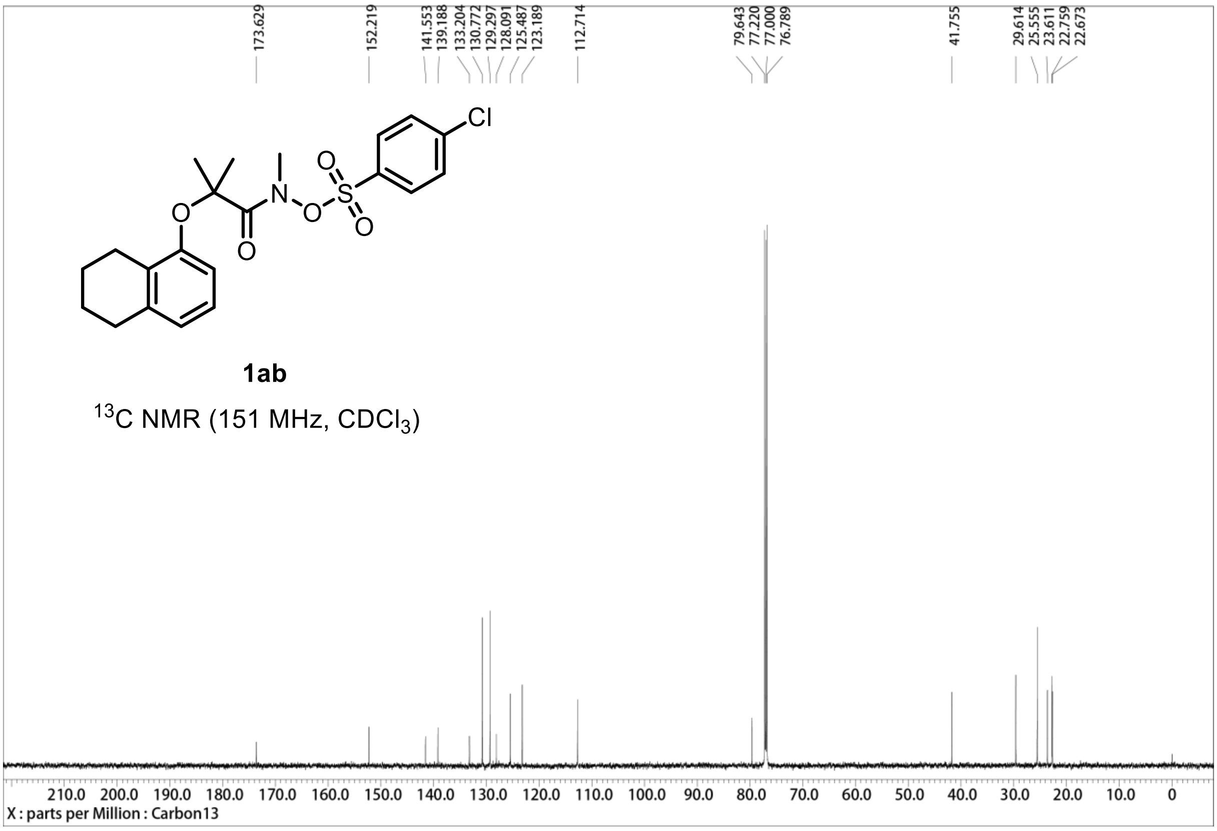
173.629 152.219 141.553 139.188 133.204 130.772 129.297 128.091 125.487 123.189 112.714 79.643 77.220 77.000 76.789 41.755 29.614 25.555 23.611 22.759 22.673
Cl
O
S
N
O
1ab
¹³C NMR (151 MHz, CDCl₃)
X : parts per Million : Carbon13

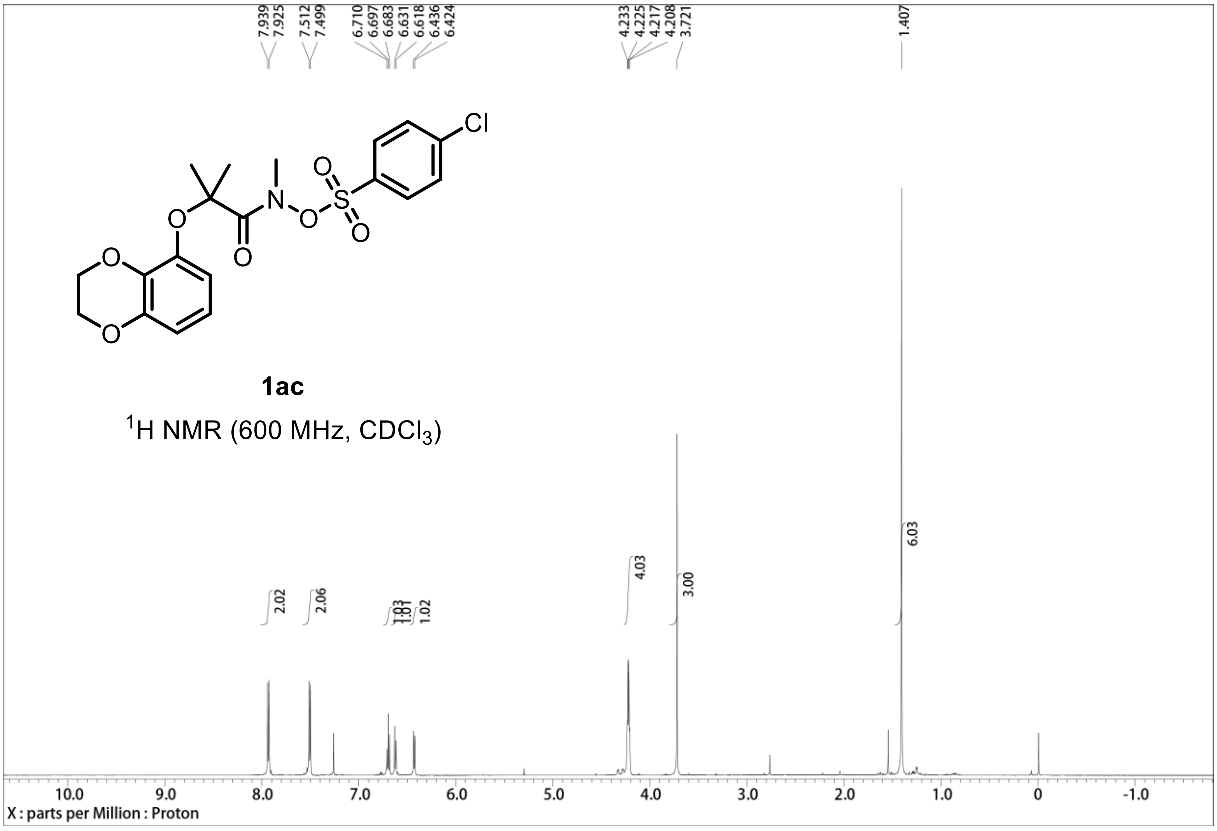
7.939
7.925
7.512
7.499
6.710
6.697
6.683
6.631
6.618
6.436
6.424
4.233
4.225
4.217
4.208
3.721
1.407
Cl
O
O
S
N
O
O
O
O
O
1ac
^{1}H NMR (600 MHz, CDCl$_3$)
2.02
2.06
1.03
1.01
1.02
4.03
3.00
6.03
10.0
9.0
8.0
7.0
6.0
5.0
4.0
3.0
2.0
1.0
0
-1.0
X : parts per Million : Proton

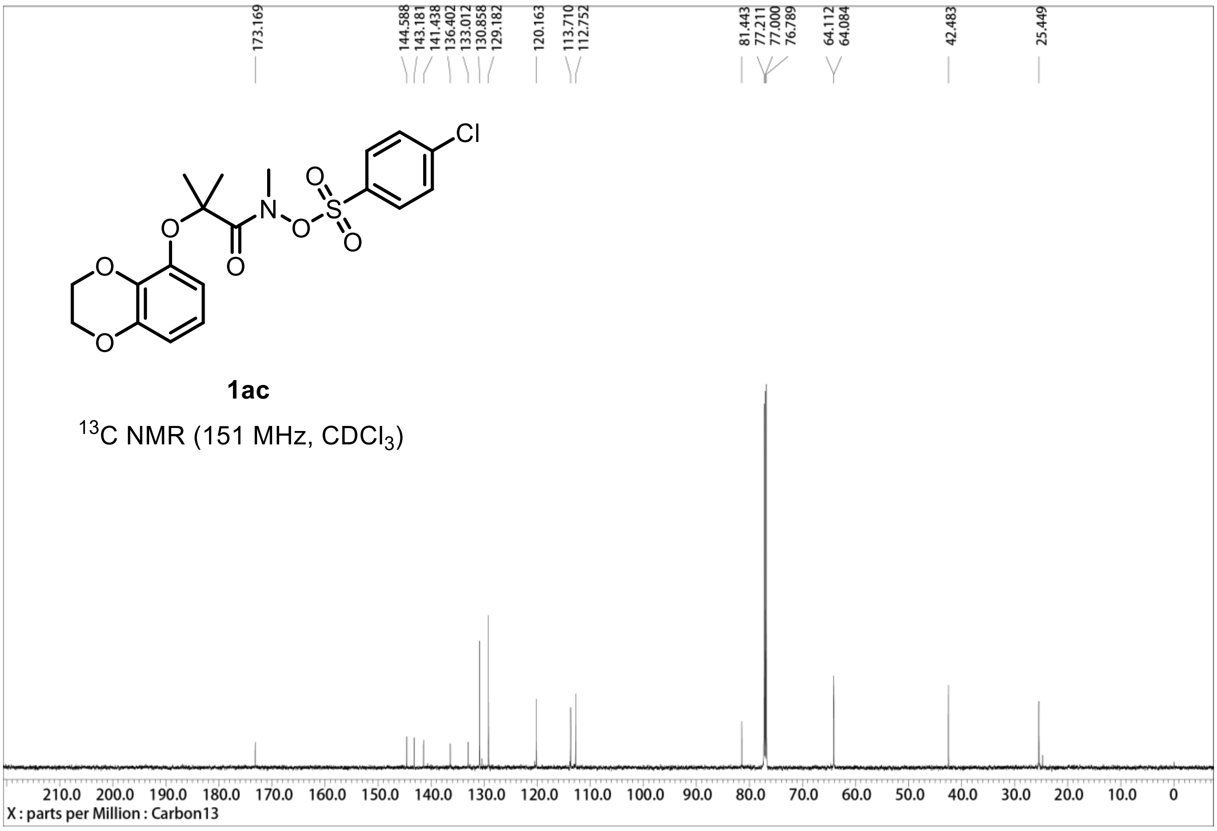
173.169
144.588
143.181
141.438
136.402
133.012
130.858
129.182
120.163
113.710
112.752
81.443
77.211
77.000
76.789
64.112
64.084
42.483
25.449
Cl
O
O
S
N
O
O
O
O
O
1ac
^{13}C NMR (151 MHz, CDCl$_3$)
210.0
200.0
190.0
180.0
170.0
160.0
150.0
140.0
130.0
120.0
110.0
100.0
90.0
80.0
70.0
60.0
50.0
40.0
30.0
20.0
10.0
0
X : parts per Million : Carbon13

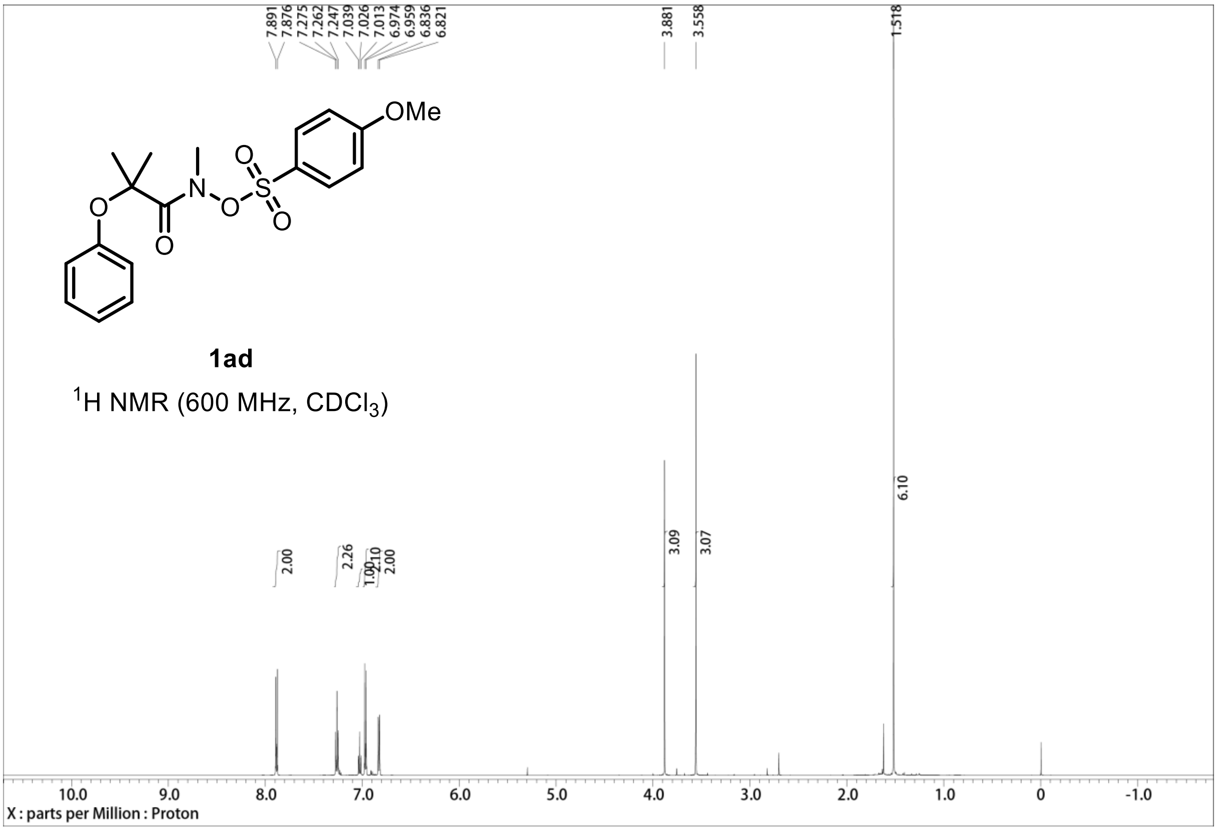
7.891
7.876
7.275
7.262
7.247
7.039
7.026
7.013
6.974
6.959
6.836
6.821
3.881
3.558
1.518
OMe
N
O
S
O
O
O
O
1ad
1H NMR (600 MHz, CDCl3)
2.00
2.26
1.00
2.10
2.00
3.09
3.07
6.10
10.0
9.0
8.0
7.0
6.0
5.0
4.0
3.0
2.0
1.0
0
-1.0
X : parts per Million : Proton

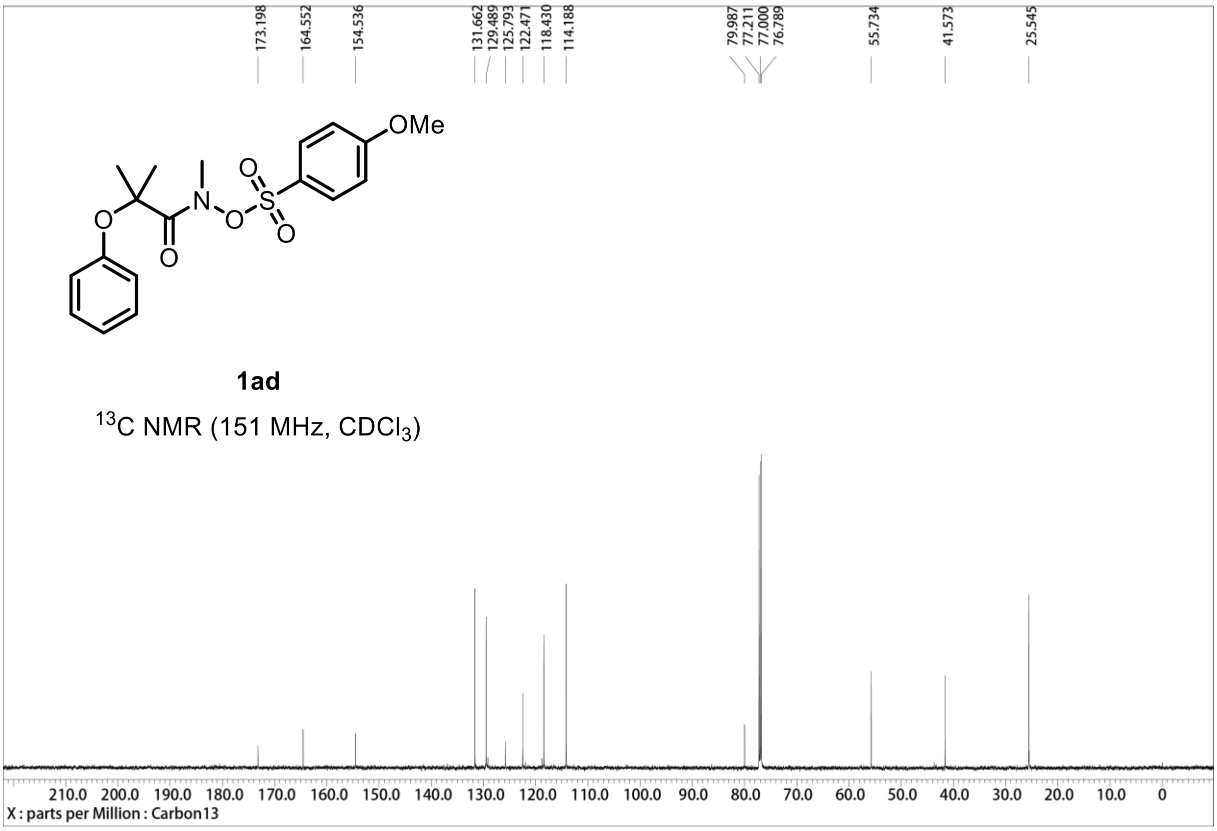
173.198
164.552
154.536
131.662
129.489
125.793
122.471
118.430
114.188
79.987
77.211
77.000
76.789
55.734
41.573
25.545
OMe
N
O
S
O
O
O
O
1ad
13C NMR (151 MHz, CDCl3)
210.0
200.0
190.0
180.0
170.0
160.0
150.0
140.0
130.0
120.0
110.0
100.0
90.0
80.0
70.0
60.0
50.0
40.0
30.0
20.0
10.0
0
X : parts per Million : Carbon13

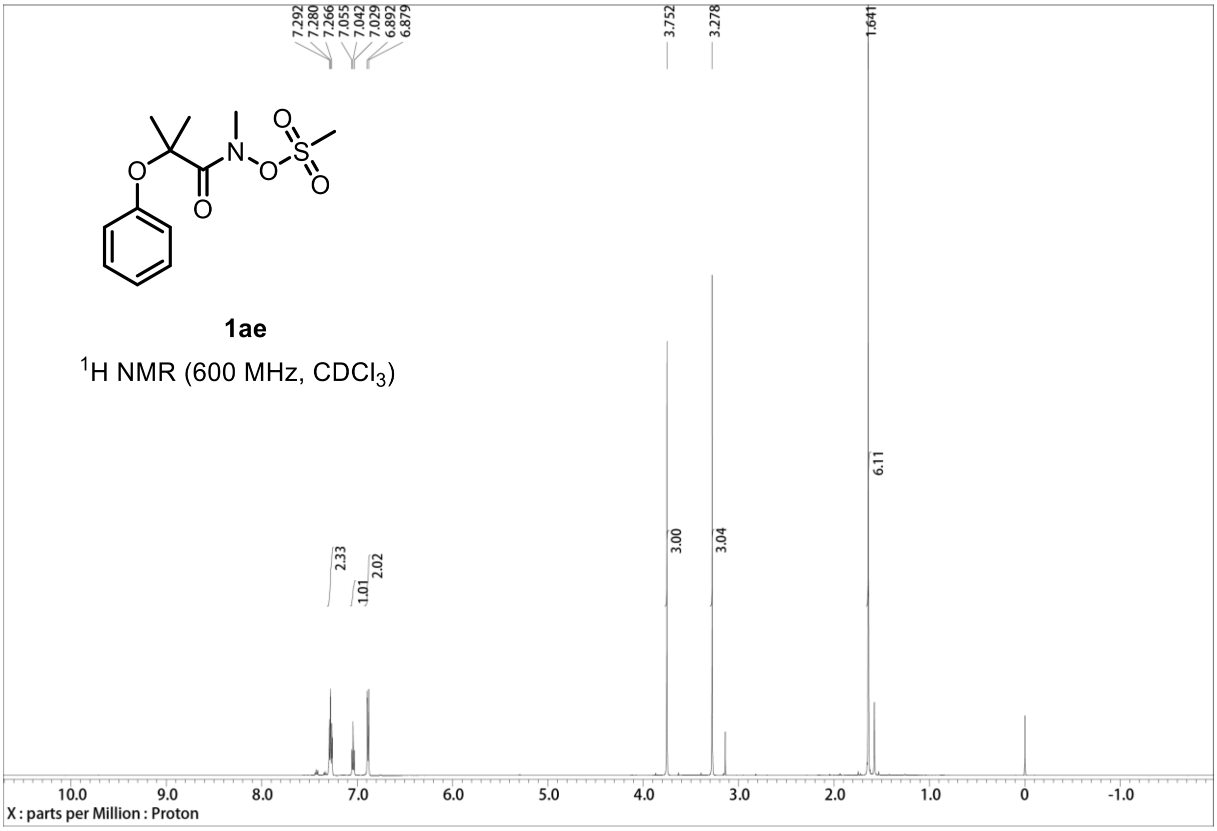
7.292
7.280
7.266
7.055
7.042
7.029
6.892
6.879
3.752
3.278
1.641
1ae
1H NMR (600 MHz, CDCl3)
6.11
3.00
3.04
2.33
1.01
2.02
10.0 9.0 8.0 7.0 6.0 5.0 4.0 3.0 2.0 1.0 0 -1.0
X : parts per Million : Proton

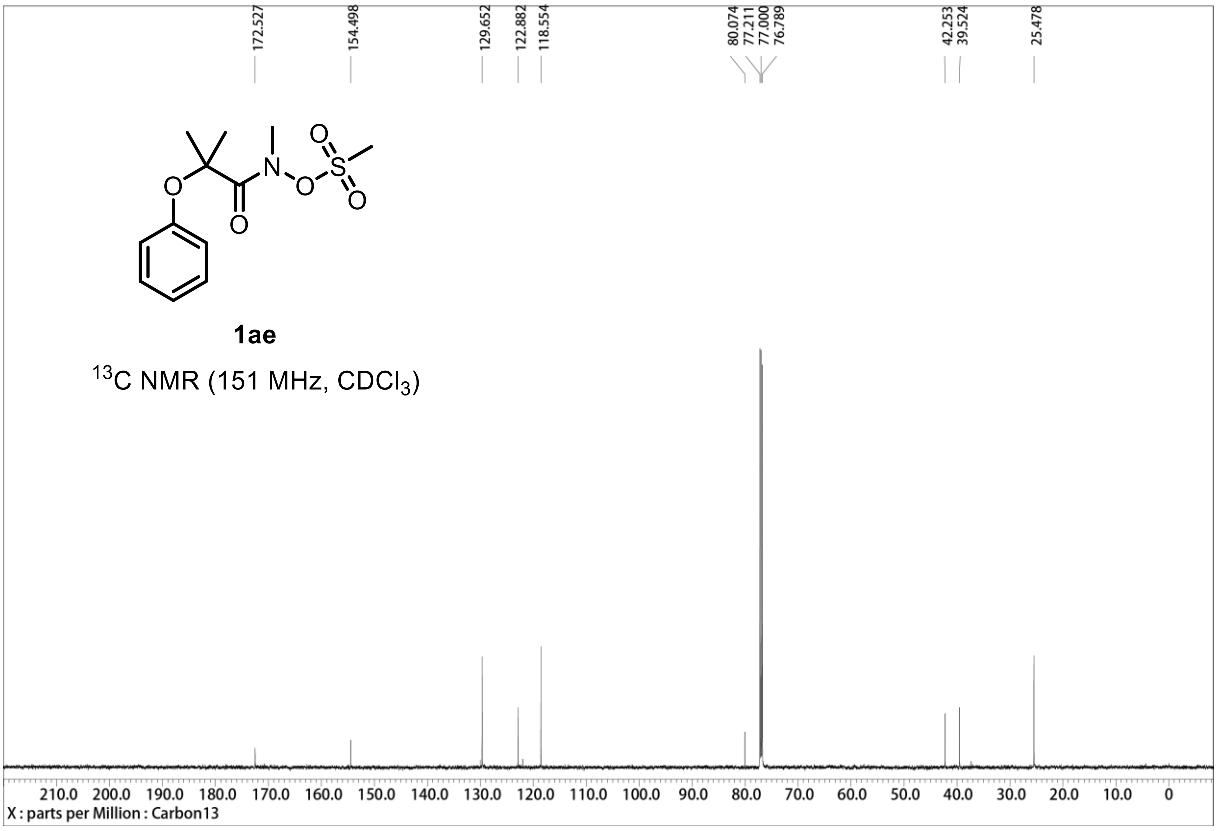
172.527
154.498
129.652
122.882
118.554
80.074
77.211
77.000
76.789
42.253
39.524
25.478
1ae
13C NMR (151 MHz, CDCl3)
210.0 200.0 190.0 180.0 170.0 160.0 150.0 140.0 130.0 120.0 110.0 100.0 90.0 80.0 70.0 60.0 50.0 40.0 30.0 20.0 10.0 0
X : parts per Million : Carbon13

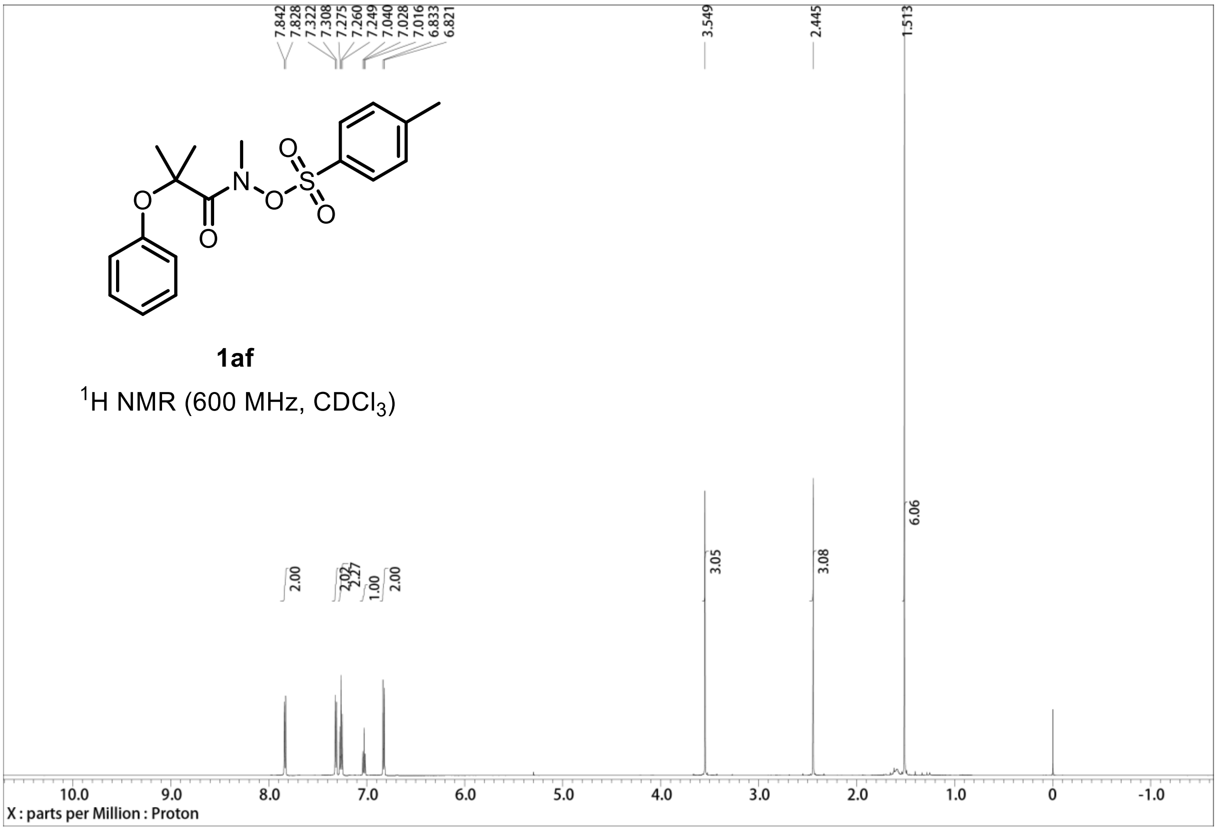
7.842
7.828
7.322
7.308
7.275
7.260
7.249
7.040
7.028
7.016
6.833
6.821
3.549
2.445
1.513
2.00
2.02
2.27
1.00
2.00
3.05
3.08
6.06
1af
^{1}H NMR (600 MHz, $CDCl_3$)
10.0
9.0
8.0
7.0
6.0
5.0
4.0
3.0
2.0
1.0
0
-1.0
X : parts per Million : Proton

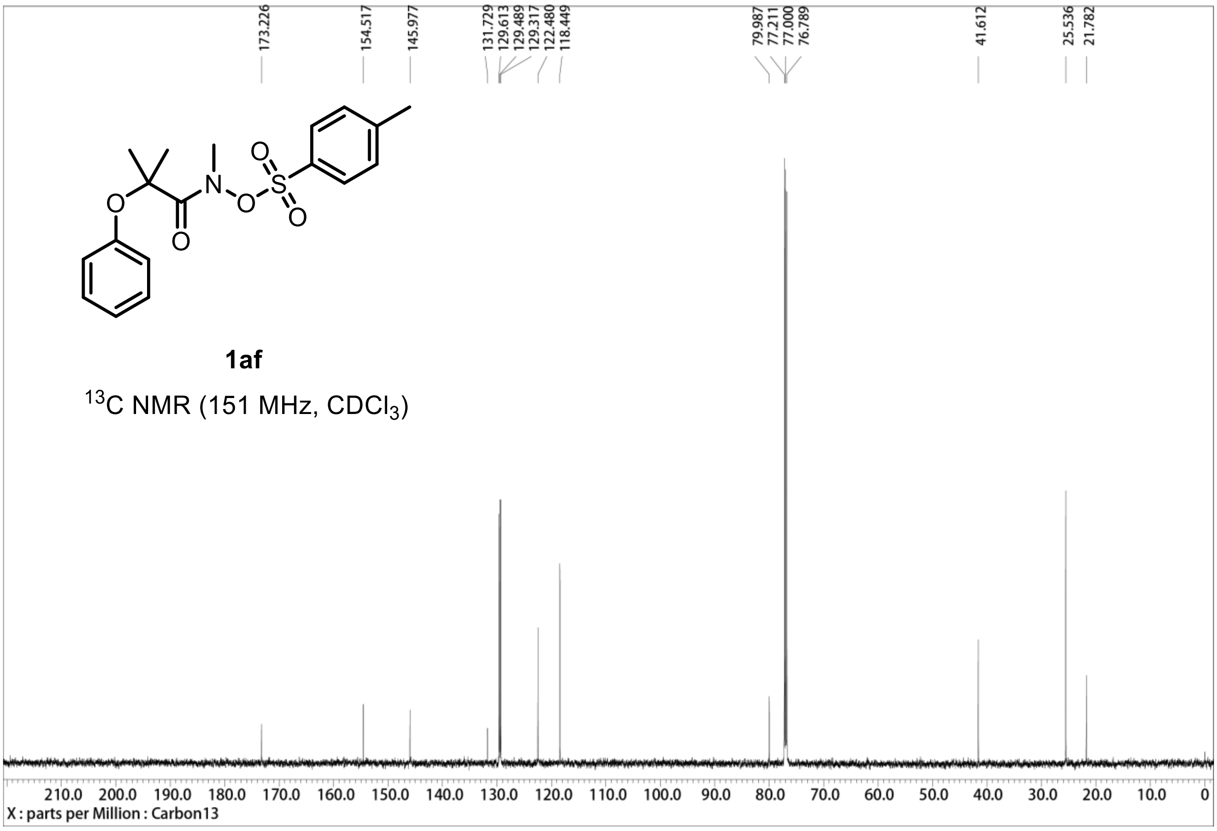
173.226
154.517
145.977
131.729
129.613
129.489
129.317
122.480
118.449
79.987
77.211
77.000
76.789
41.612
25.536
21.782
1af
^{13}C NMR (151 MHz, $CDCl_3$)
210.0
200.0
190.0
180.0
170.0
160.0
150.0
140.0
130.0
120.0
110.0
100.0
90.0
80.0
70.0
60.0
50.0
40.0
30.0
20.0
10.0
0
X : parts per Million : Carbon13

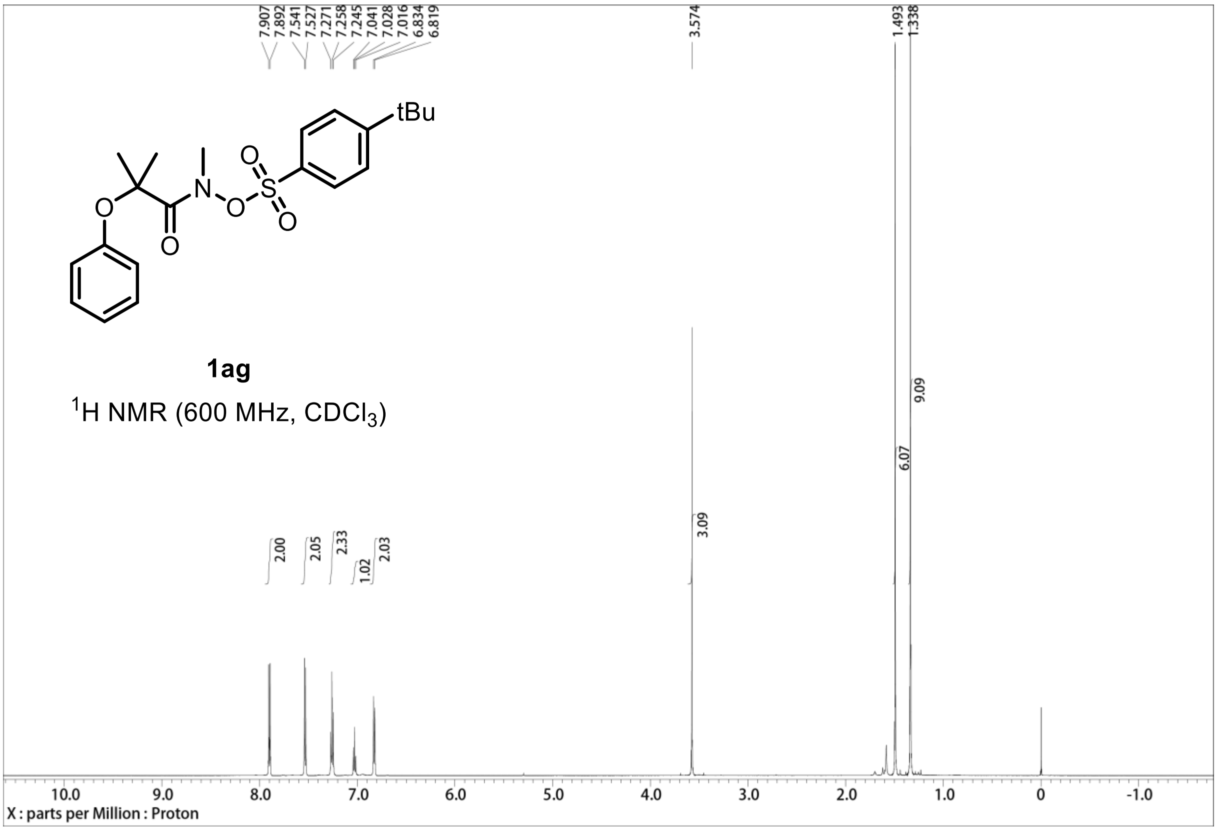
7.907
7.892
7.541
7.527
7.271
7.258
7.245
7.041
7.028
7.016
6.834
6.819
3.574
1.493
1.338
tBu
1ag
1H NMR (600 MHz, CDCl3)
2.00
2.05
2.33
1.02
2.03
3.09
6.07
9.09
X : parts per Million : Proton

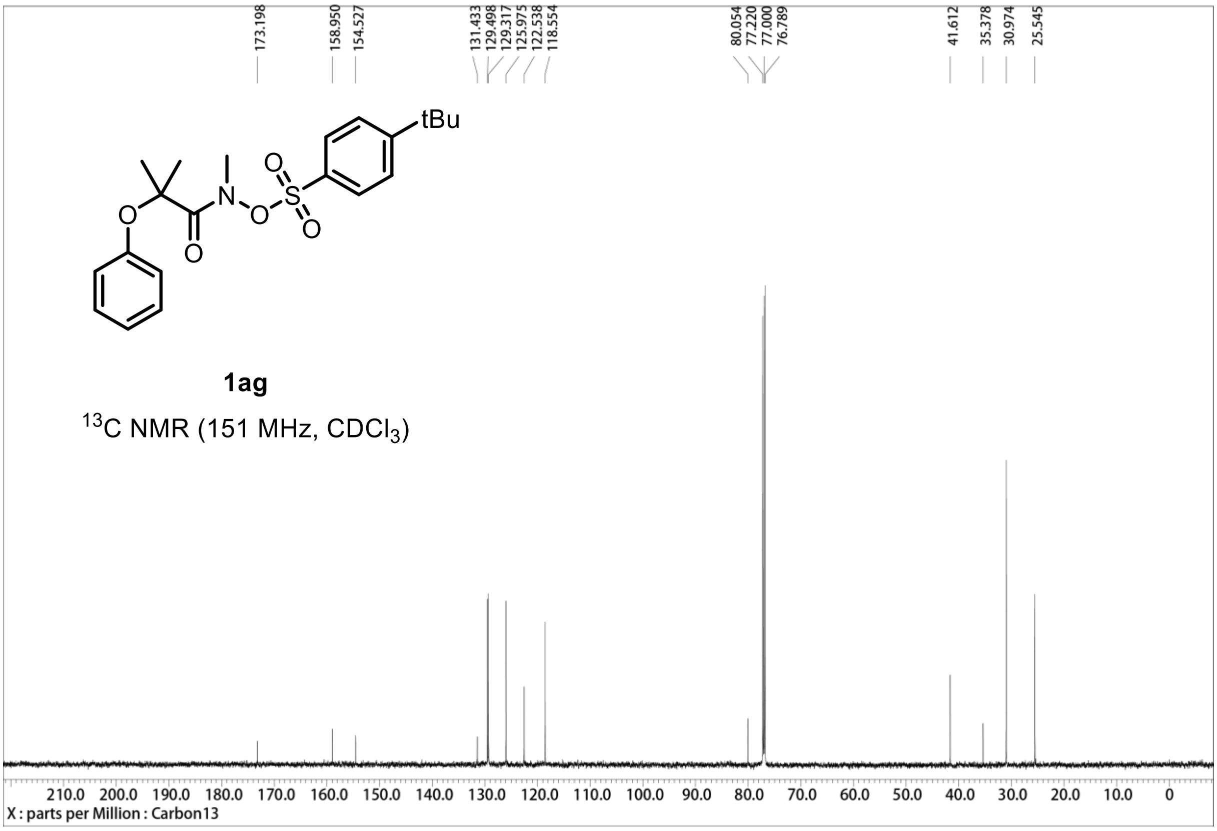
173.198
158.950
154.527
131.433
129.498
129.317
125.975
122.538
118.554
80.054
77.220
77.000
76.789
41.612
35.378
30.974
25.545
tBu
1ag
13C NMR (151 MHz, CDCl3)
X : parts per Million : Carbon13

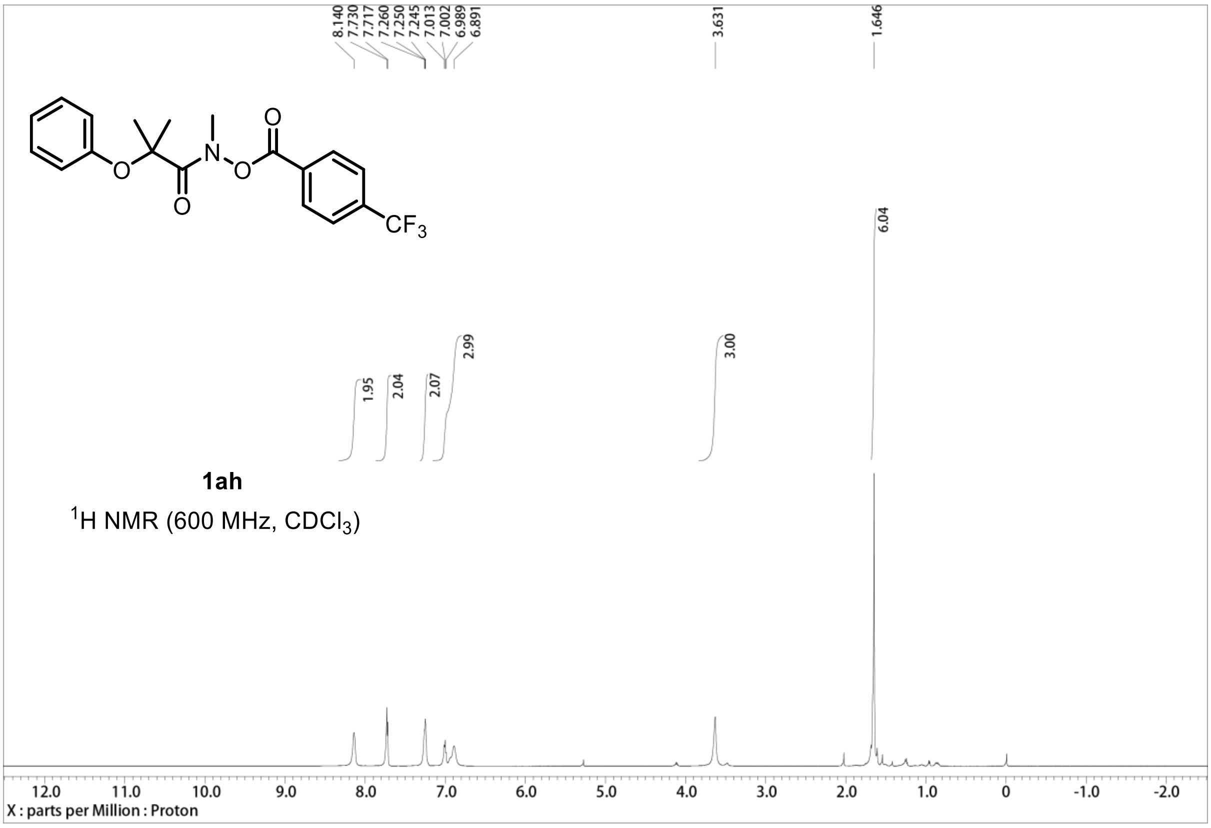
8.140
7.730
7.717
7.260
7.250
7.245
7.013
7.002
6.989
6.891
3.631
1.646
O
N
O
O
O
CF3
6.04
1.95
2.04
2.07
2.99
3.00
1ah
1H NMR (600 MHz, CDCl3)
12.0 11.0 10.0 9.0 8.0 7.0 6.0 5.0 4.0 3.0 2.0 1.0 0 -1.0 -2.0
X : parts per Million : Proton

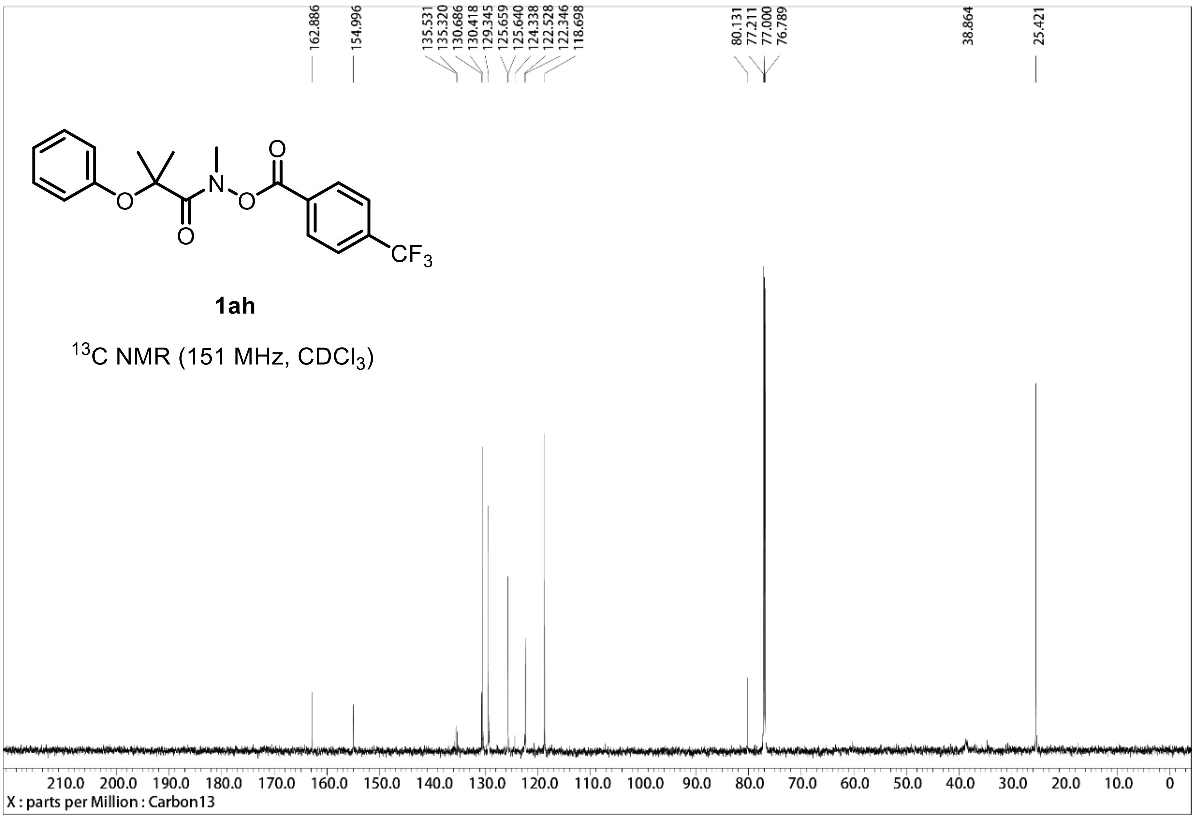
162.886
154.996
135.531
135.320
130.686
130.418
129.345
125.659
125.640
124.338
122.528
122.346
118.698
80.131
77.211
77.000
76.789
38.864
25.421
O
N
O
O
O
CF3
1ah
13C NMR (151 MHz, CDCl3)
210.0 200.0 190.0 180.0 170.0 160.0 150.0 140.0 130.0 120.0 110.0 100.0 90.0 80.0 70.0 60.0 50.0 40.0 30.0 20.0 10.0 0
X : parts per Million : Carbon13

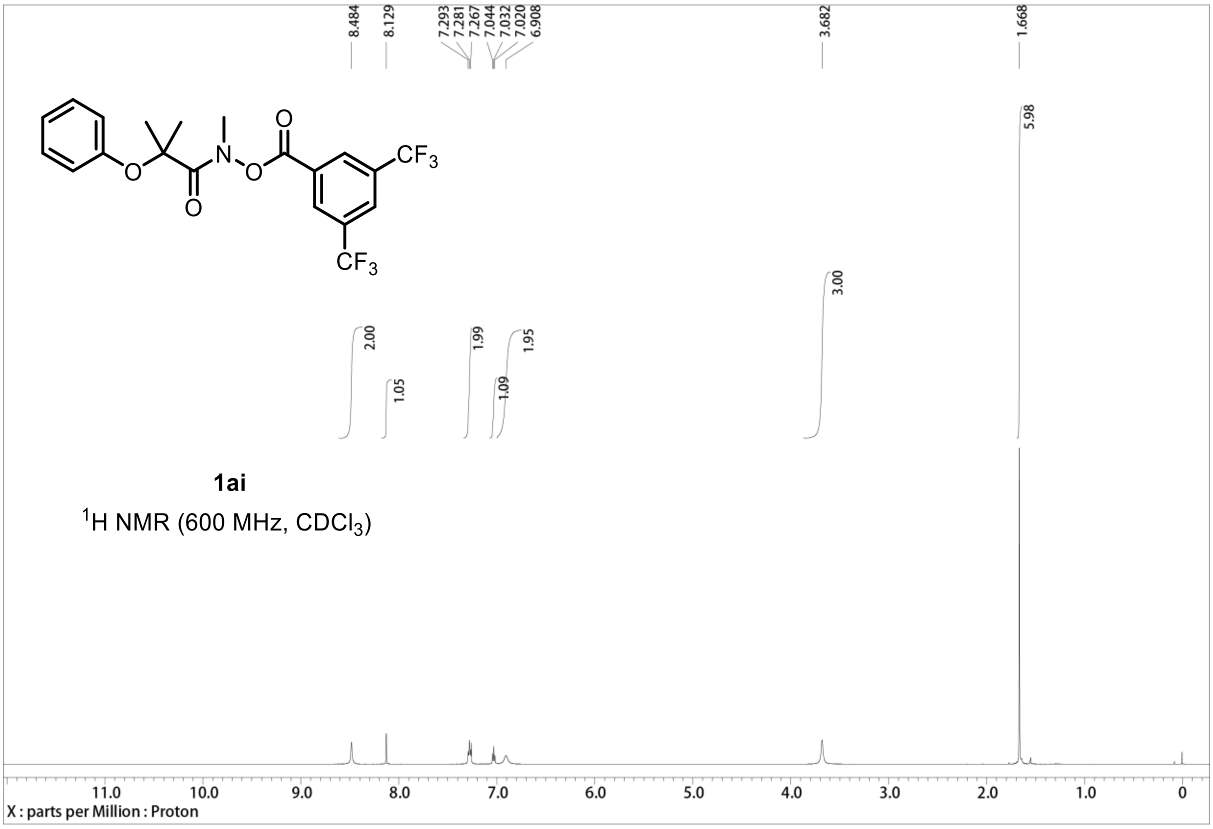
8.484
8.129
7.293
7.281
7.267
7.044
7.032
7.020
6.908
3.682
1.668
5.98
3.00
2.00
1.05
1.99
1.09
1.95
CF3
CF3
O
N
O
O
O
1ai
1H NMR (600 MHz, CDCl3)
11.0
10.0
9.0
8.0
7.0
6.0
5.0
4.0
3.0
2.0
1.0
0
X : parts per Million : Proton

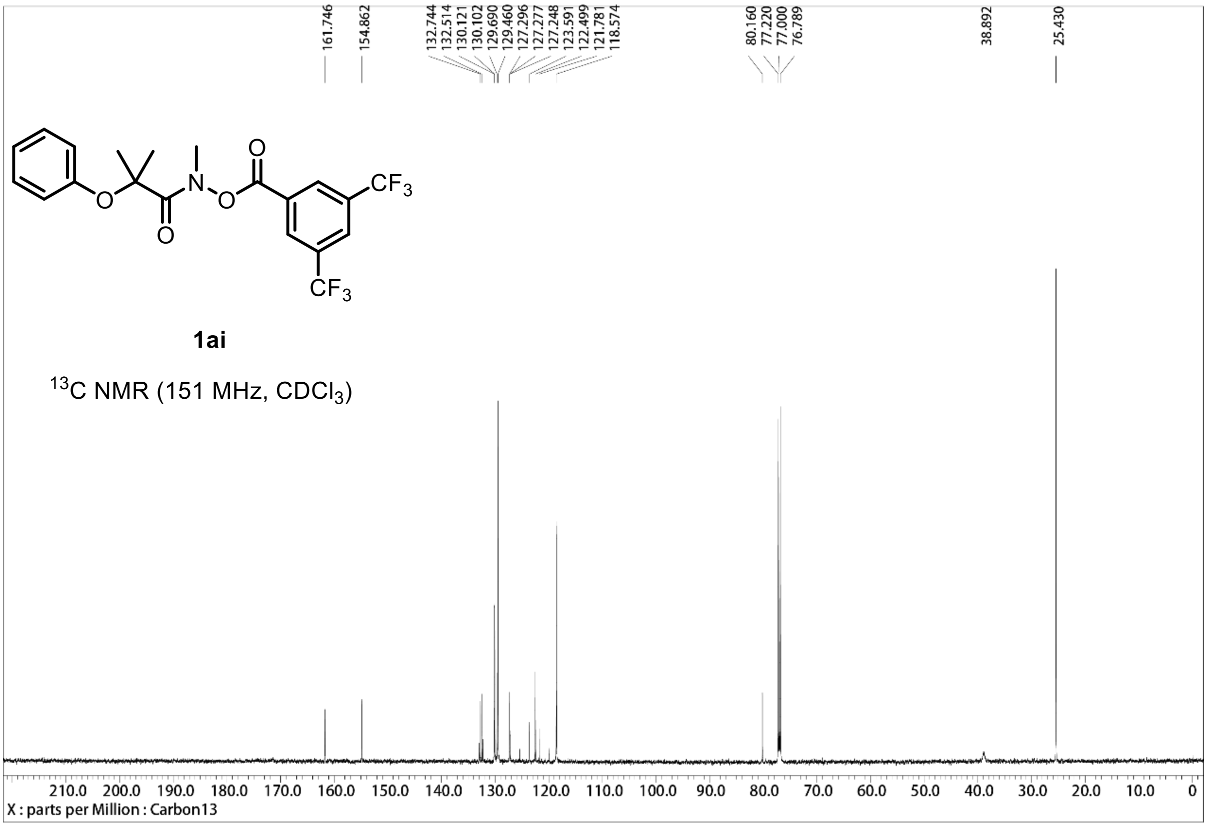
161.746
154.862
132.744
132.514
130.121
130.102
129.690
129.460
127.296
127.277
127.248
123.591
122.499
121.781
118.574
80.160
77.220
77.000
76.789
38.892
25.430
CF3
CF3
O
N
O
O
O
1ai
13C NMR (151 MHz, CDCl3)
210.0
200.0
190.0
180.0
170.0
160.0
150.0
140.0
130.0
120.0
110.0
100.0
90.0
80.0
70.0
60.0
50.0
40.0
30.0
20.0
10.0
0
X : parts per Million : Carbon13

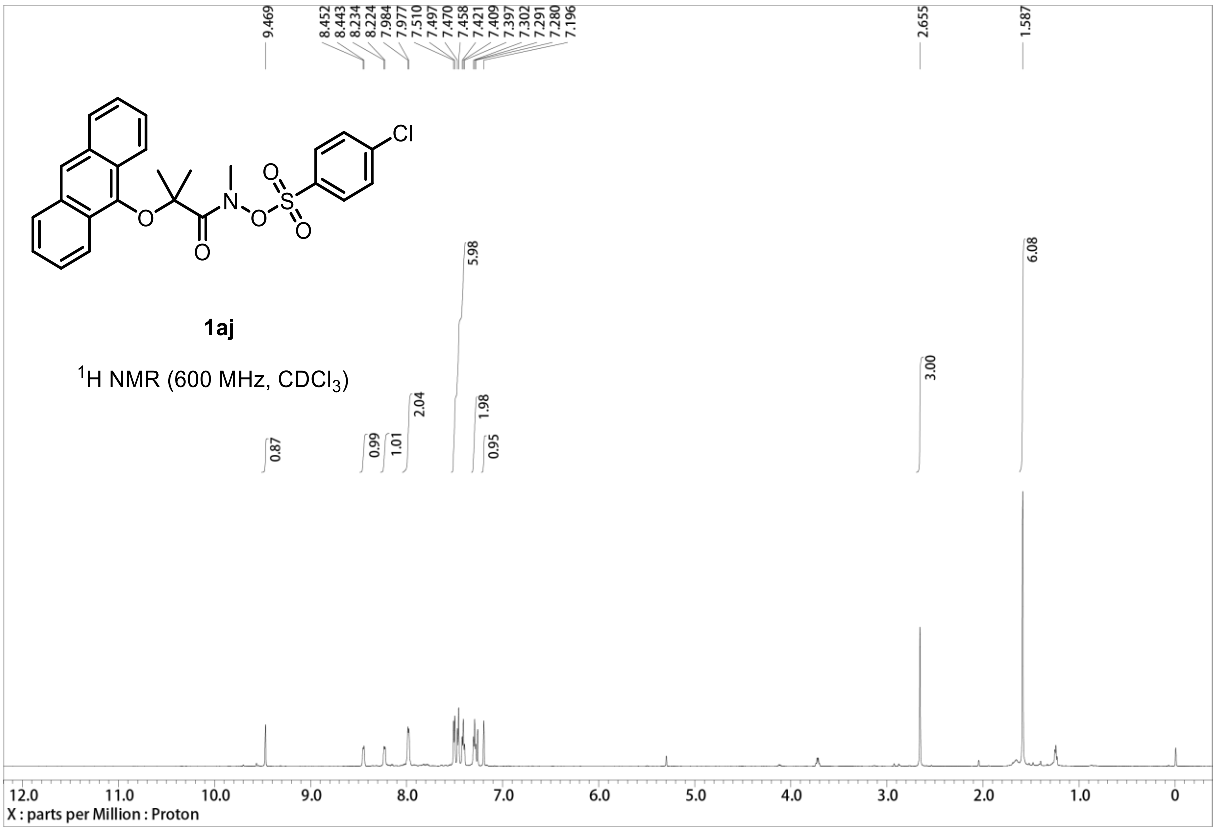
9.469
8.452
8.443
8.234
8.224
7.984
7.977
7.510
7.497
7.470
7.458
7.421
7.409
7.397
7.302
7.291
7.280
7.196
2.655
1.587
Cl
O
N
O
S
O
O
O
1aj
^{1}H NMR (600 MHz, $CDCl_3$)
0.87
0.99
1.01
2.04
5.98
1.98
0.95
3.00
6.08
12.0 11.0 10.0 9.0 8.0 7.0 6.0 5.0 4.0 3.0 2.0 1.0 0
X : parts per Million : Proton

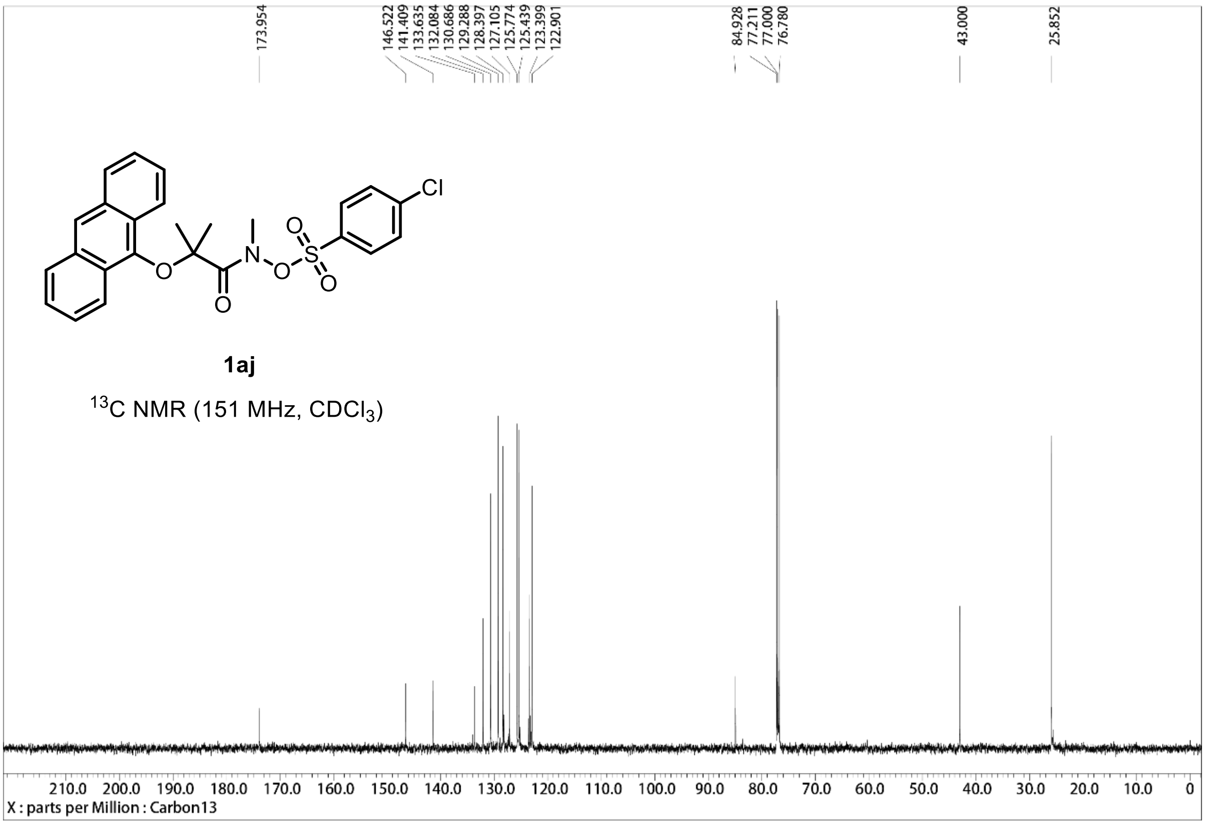
173.954
146.522
141.409
133.635
132.084
130.686
129.288
128.397
127.105
125.774
125.439
123.399
122.901
84.928
77.211
77.000
76.780
43.000
25.852
Cl
O
N
O
S
O
O
O
1aj
^{13}C NMR (151 MHz, $CDCl_3$)
210.0 200.0 190.0 180.0 170.0 160.0 150.0 140.0 130.0 120.0 110.0 100.0 90.0 80.0 70.0 60.0 50.0 40.0 30.0 20.0 10.0 0
X : parts per Million : Carbon13

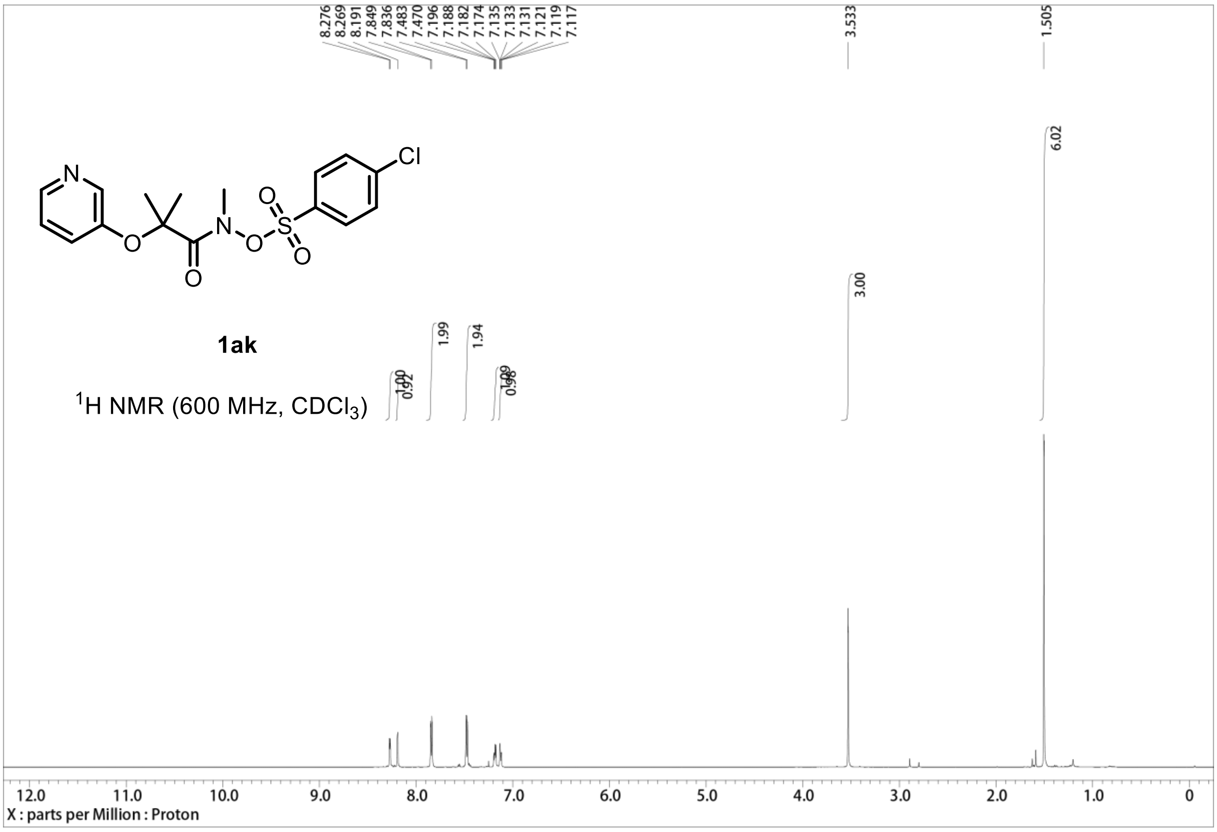
8.276
8.269
8.191
7.849
7.836
7.483
7.470
7.196
7.188
7.182
7.174
7.135
7.133
7.131
7.121
7.119
7.117
3.533
1.505
6.02
3.00
1.99
1.94
1.00
0.92
0.98
1ak
1H NMR (600 MHz, CDCl3)
12.0
11.0
10.0
9.0
8.0
7.0
6.0
5.0
4.0
3.0
2.0
1.0
0
X : parts per Million : Proton

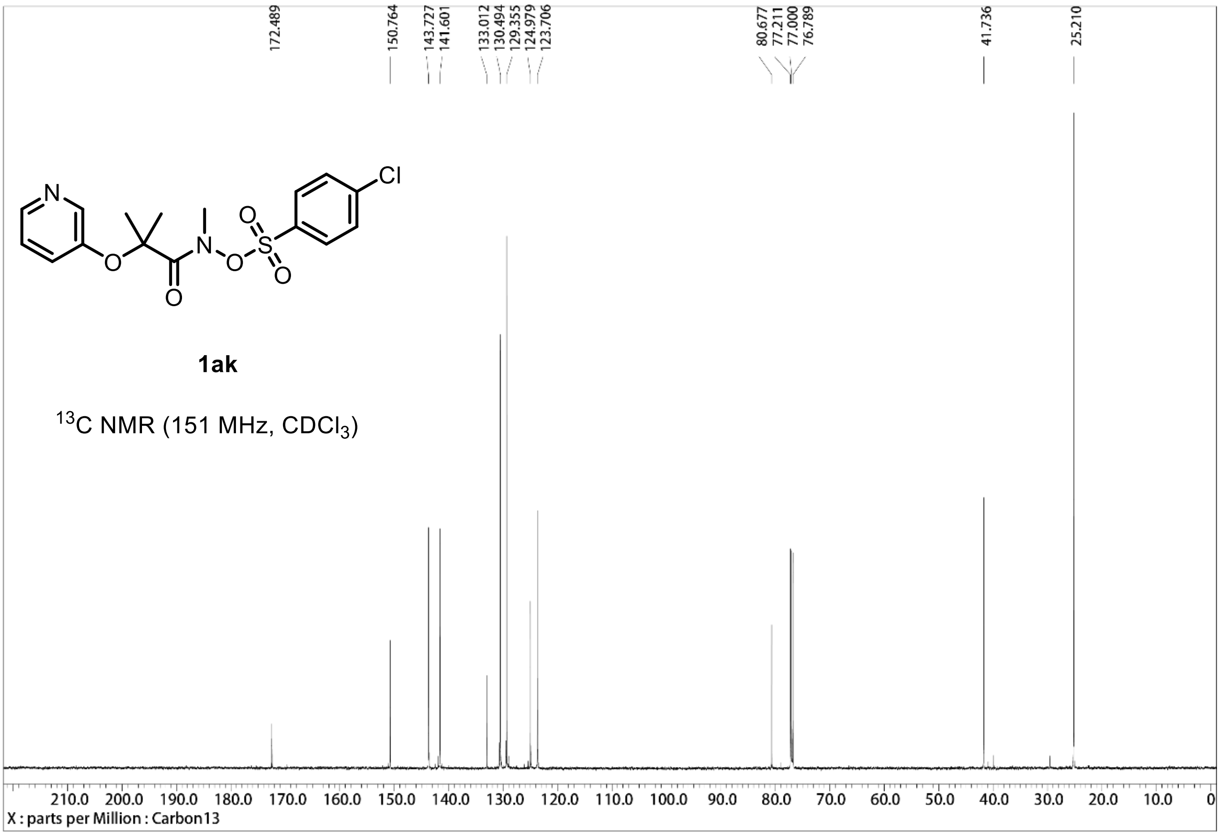
172.489
150.764
143.727
141.601
133.012
130.494
129.355
124.979
123.706
80.677
77.211
77.000
76.789
41.736
25.210
1ak
13C NMR (151 MHz, CDCl3)
210.0
200.0
190.0
180.0
170.0
160.0
150.0
140.0
130.0
120.0
110.0
100.0
90.0
80.0
70.0
60.0
50.0
40.0
30.0
20.0
10.0
0
X : parts per Million : Carbon13

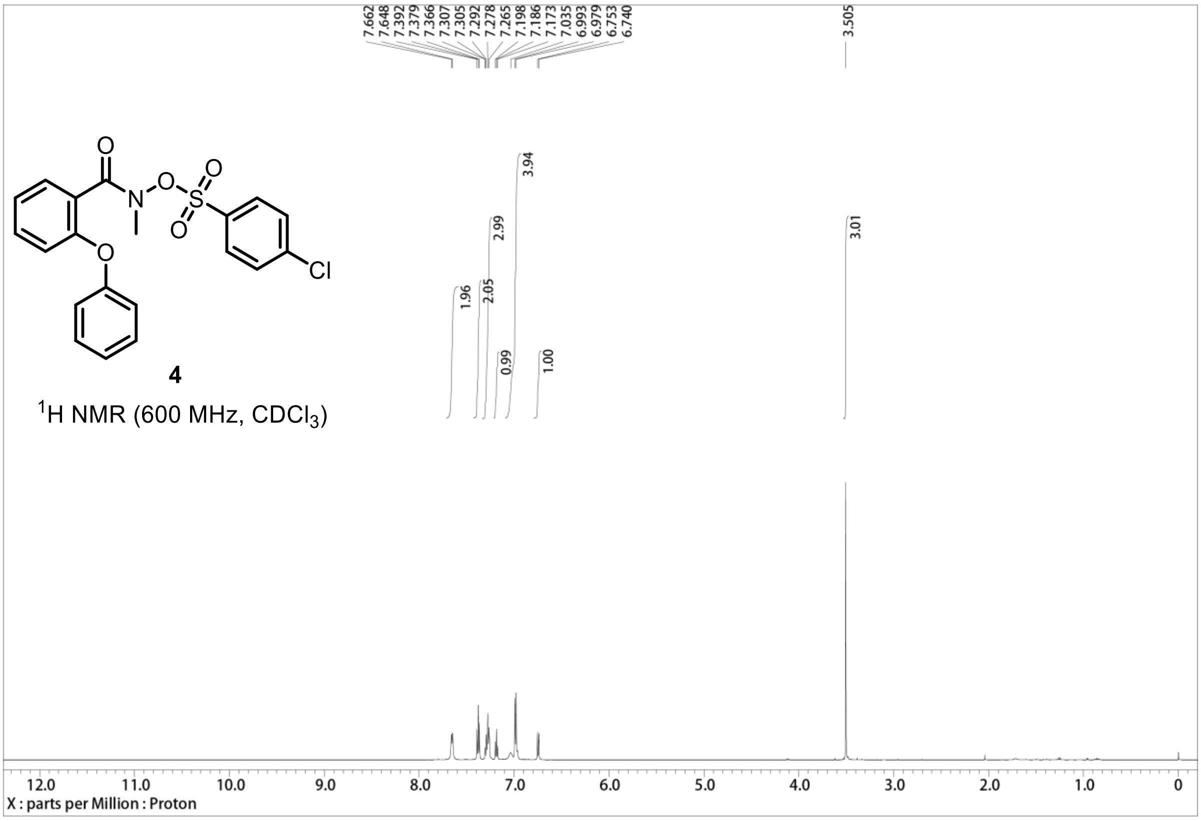
7.662
7.648
7.392
7.379
7.366
7.307
7.305
7.292
7.278
7.265
7.198
7.186
7.173
7.035
6.993
6.979
6.753
6.740
3.505
3.94
3.01
2.99
1.96
2.05
0.99
1.00
4
1H NMR (600 MHz, CDCl3)
X : parts per Million : Proton

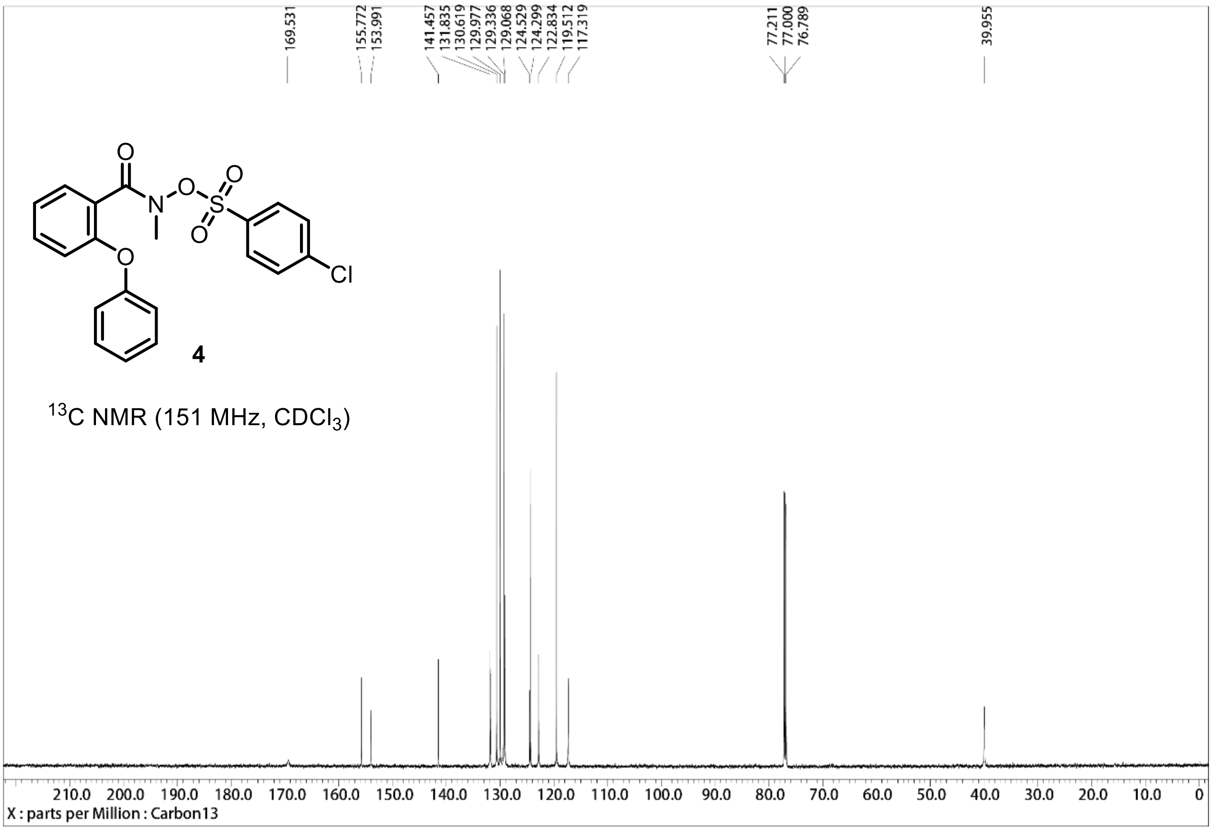
169.531
155.772
153.991
141.457
131.835
130.619
129.977
129.336
129.068
124.529
124.299
122.834
119.512
117.319
77.211
77.000
76.789
39.955
4
13C NMR (151 MHz, CDCl3)
X : parts per Million : Carbon13

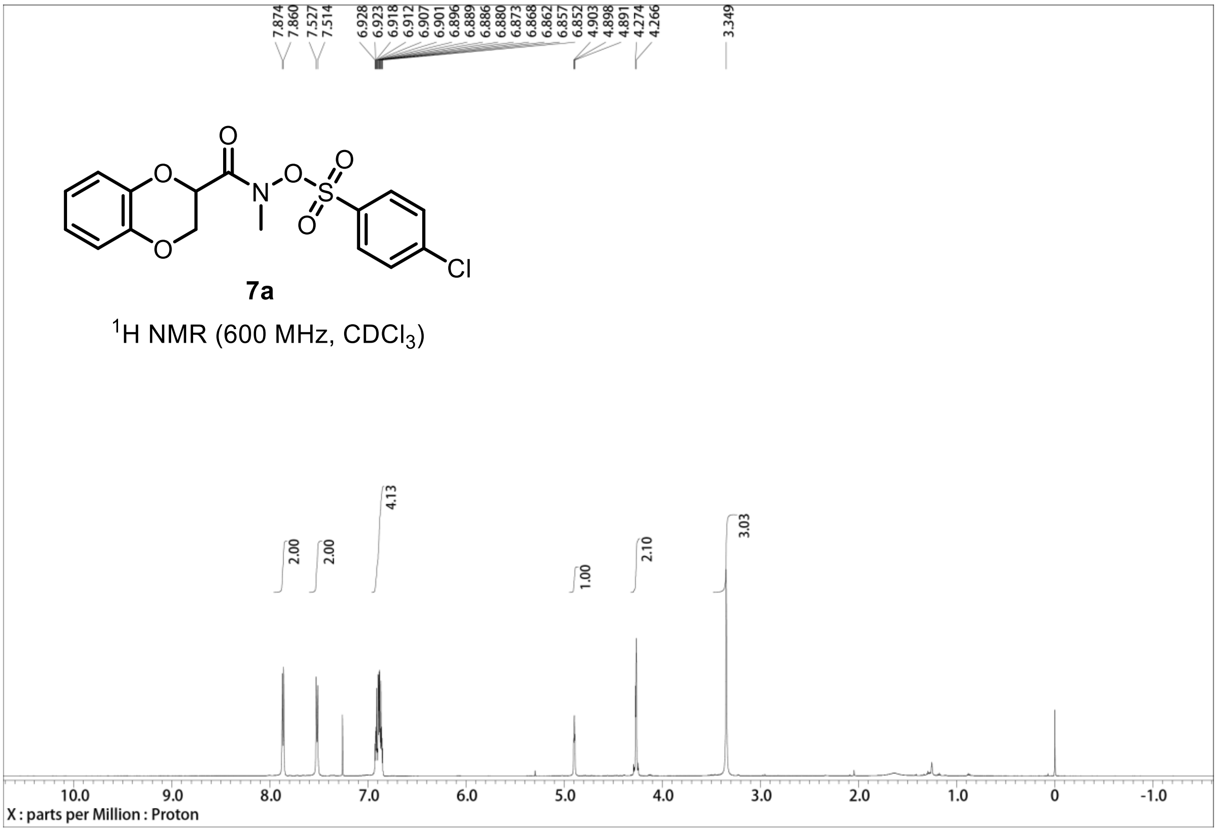
7.874
7.860
7.527
7.514
6.928
6.923
6.918
6.912
6.907
6.901
6.896
6.889
6.886
6.880
6.873
6.868
6.862
6.857
6.852
4.903
4.898
4.891
4.274
4.266
3.349
7a
1H NMR (600 MHz, CDCl3)
2.00
2.00
4.13
1.00
2.10
3.03
10.0 9.0 8.0 7.0 6.0 5.0 4.0 3.0 2.0 1.0 0 -1.0
X : parts per Million : Proton

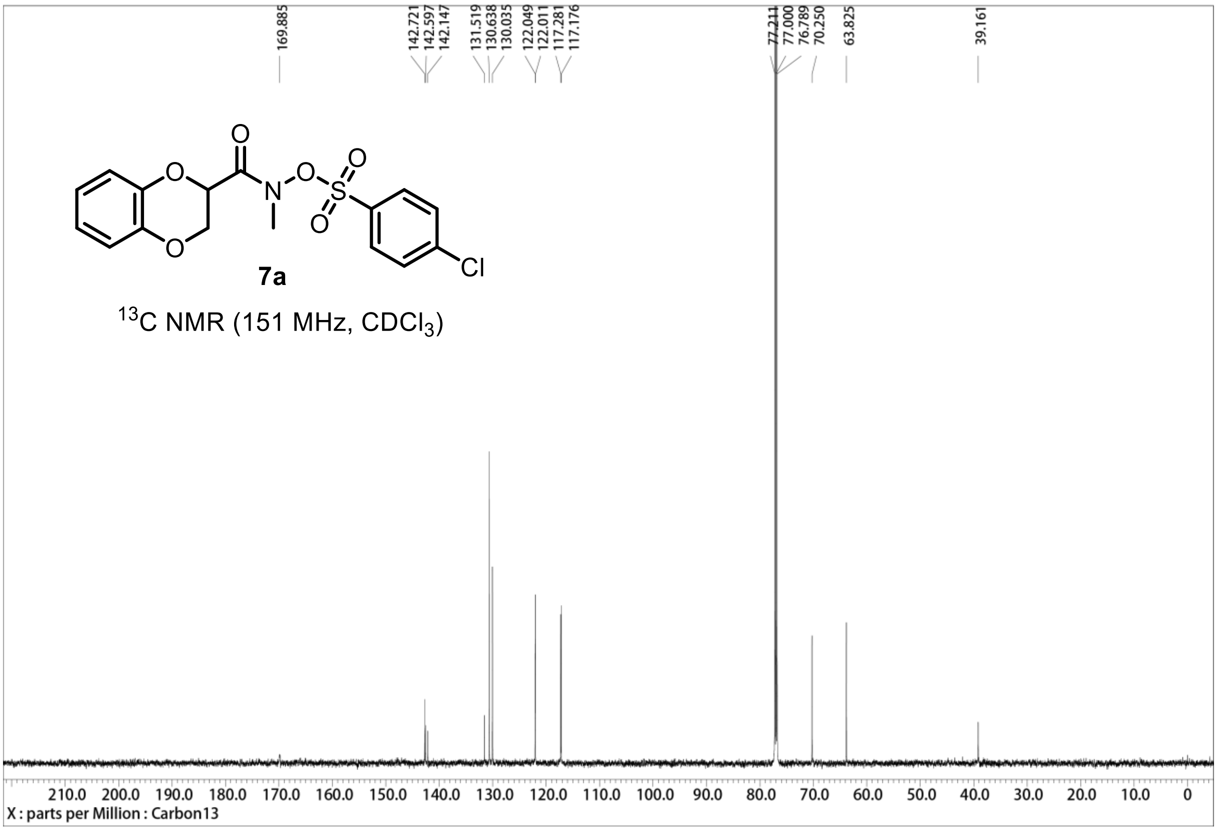
169.885
142.721
142.597
142.147
131.519
130.638
130.035
122.049
122.011
117.281
117.176
77.211
77.000
76.789
70.250
63.825
39.161
7a
13C NMR (151 MHz, CDCl3)
210.0 200.0 190.0 180.0 170.0 160.0 150.0 140.0 130.0 120.0 110.0 100.0 90.0 80.0 70.0 60.0 50.0 40.0 30.0 20.0 10.0 0
X : parts per Million : Carbon13

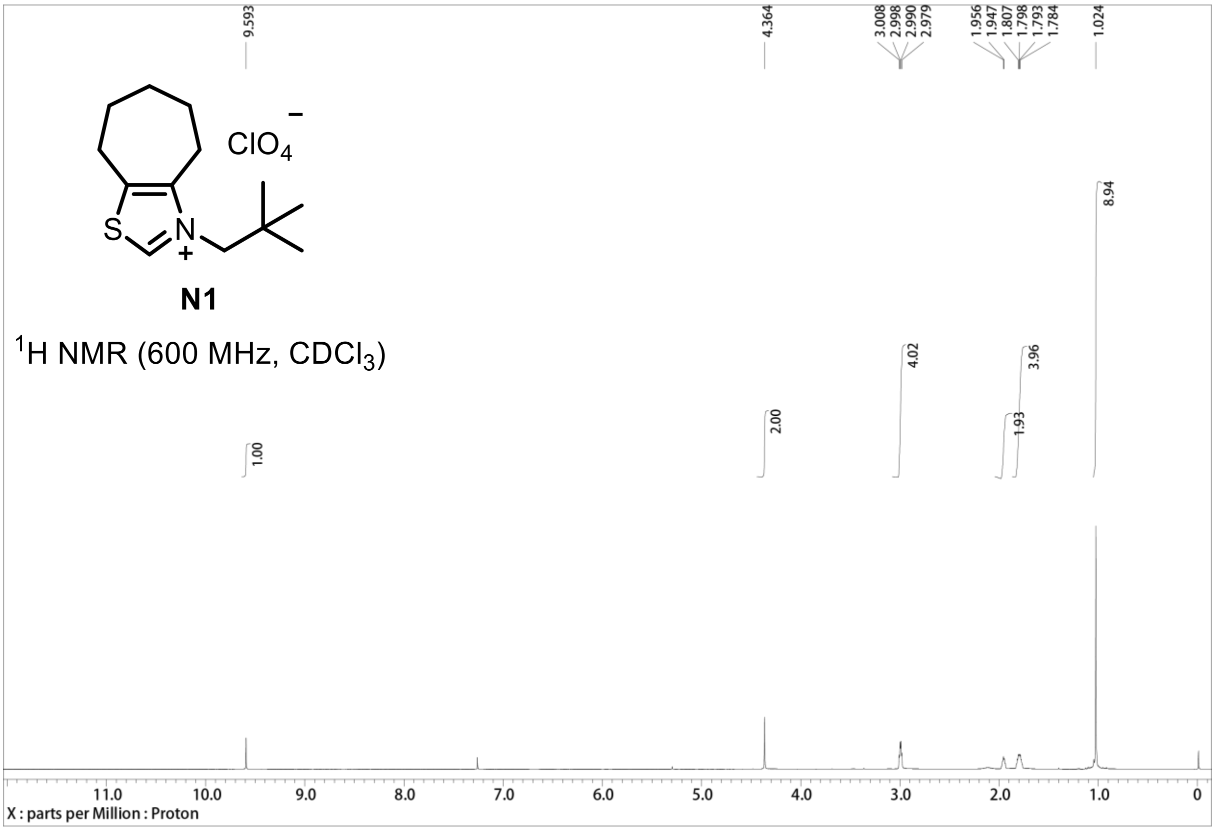
9.593
4.364
3.008
2.998
2.990
2.979
1.956
1.947
1.807
1.798
1.793
1.784
1.024
ClO4
S
N
N1
1H NMR (600 MHz, CDCl3)
1.00
2.00
4.02
1.93
3.96
8.94
11.0 10.0 9.0 8.0 7.0 6.0 5.0 4.0 3.0 2.0 1.0 0
X : parts per Million : Proton

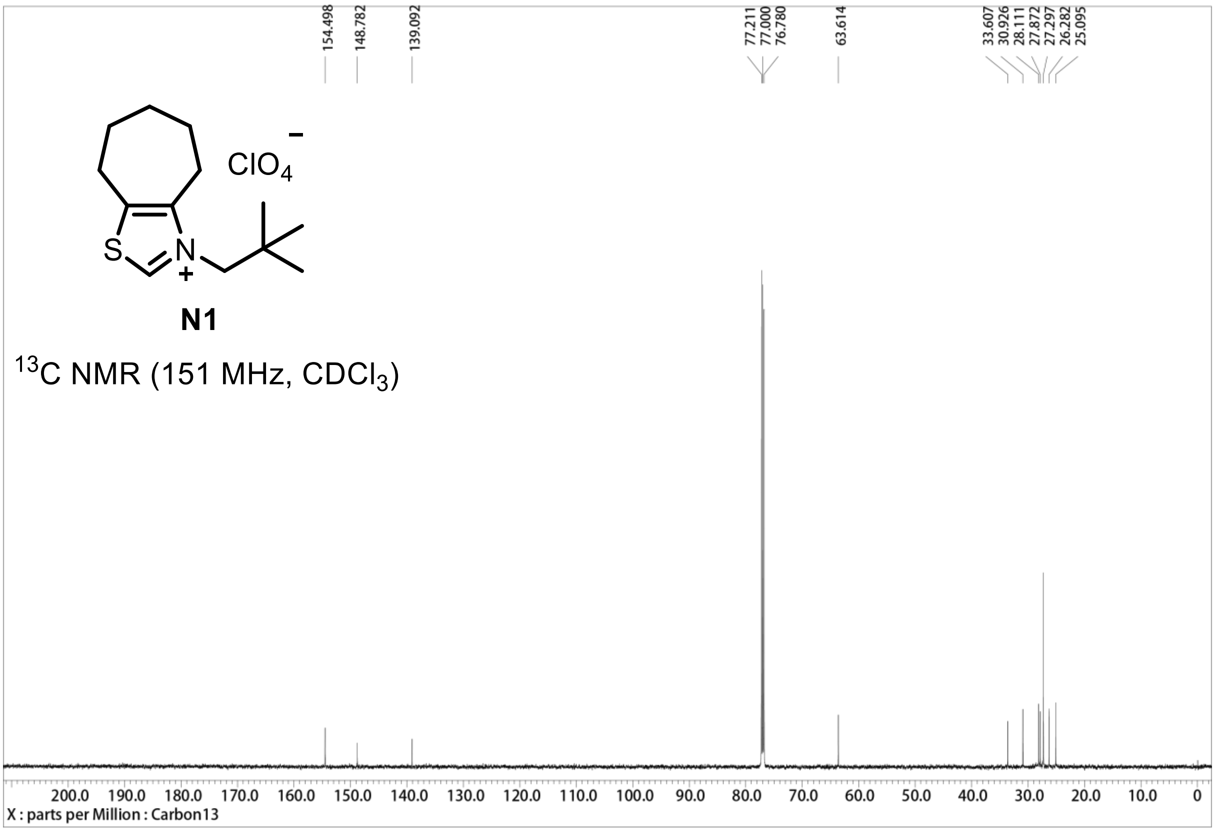
154.498
148.782
139.092
77.211
77.000
76.780
63.614
33.607
30.926
28.111
27.872
27.297
26.282
25.095
ClO4
S
N
N1
13C NMR (151 MHz, CDCl3)
200.0 190.0 180.0 170.0 160.0 150.0 140.0 130.0 120.0 110.0 100.0 90.0 80.0 70.0 60.0 50.0 40.0 30.0 20.0 10.0 0
X : parts per Million : Carbon13

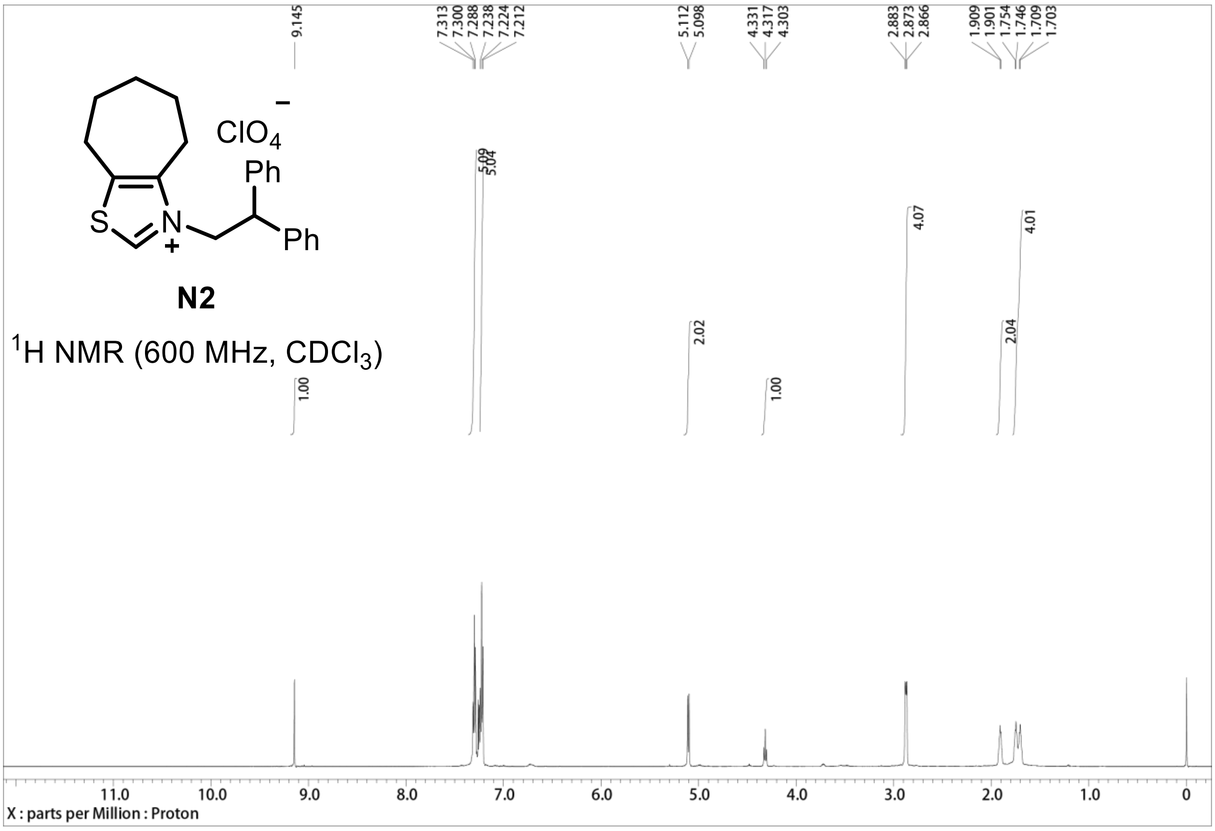

9.145
7.313
7.300
7.288
7.238
7.224
7.212
5.112
5.098
4.331
4.317
4.303
2.883
2.873
2.866
1.909
1.901
1.754
1.746
1.709
1.703
ClO4
Ph
Ph
S
N
N2
1H NMR (600 MHz, CDCl3)
1.00
5.09
5.04
2.02
1.00
4.07
2.04
4.01
11.0
10.0
9.0
8.0
7.0
6.0
5.0
4.0
3.0
2.0
1.0
0
X : parts per Million : Proton

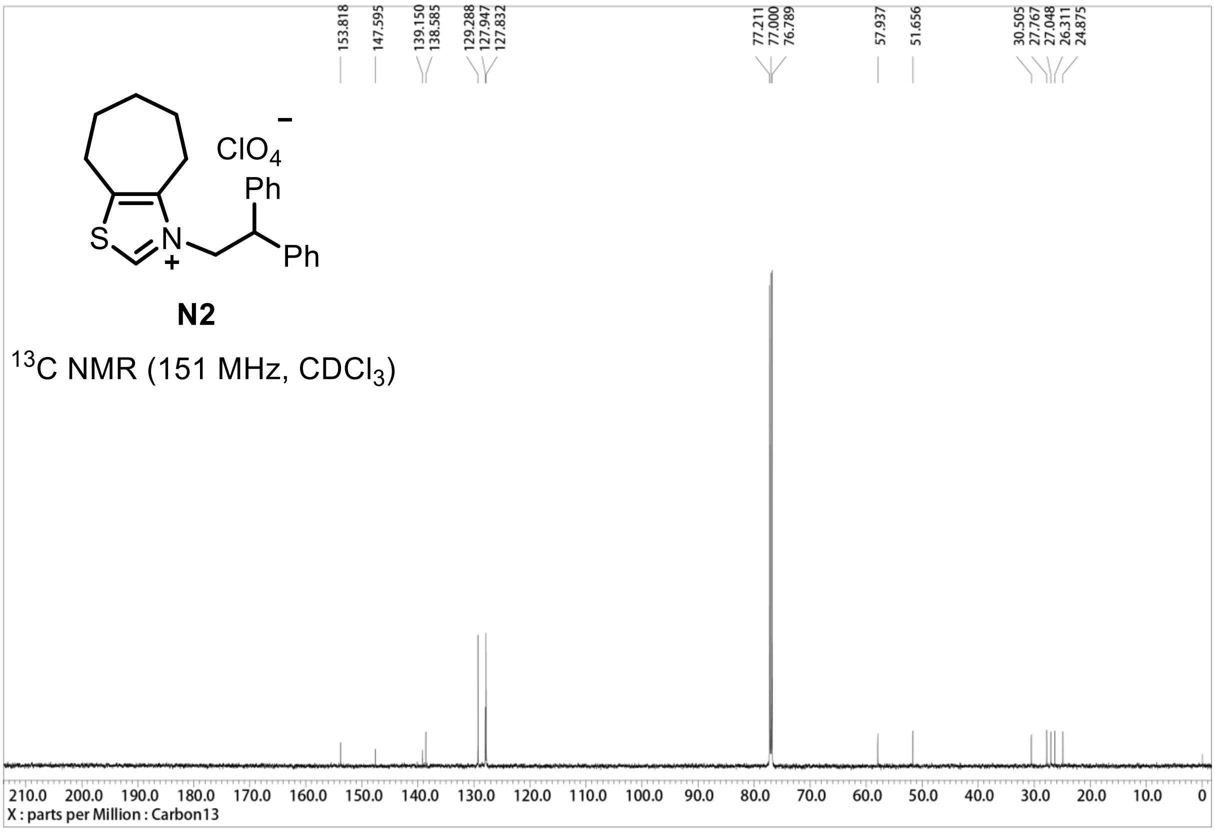

153.818
147.595
139.150
138.585
129.288
127.947
127.832
77.211
77.000
76.789
57.937
51.656
30.505
27.767
27.048
26.311
24.875
ClO4
Ph
Ph
S
N
N2
13C NMR (151 MHz, CDCl3)
210.0
200.0
190.0
180.0
170.0
160.0
150.0
140.0
130.0
120.0
110.0
100.0
90.0
80.0
70.0
60.0
50.0
40.0
30.0
20.0
10.0
0
X : parts per Million : Carbon13

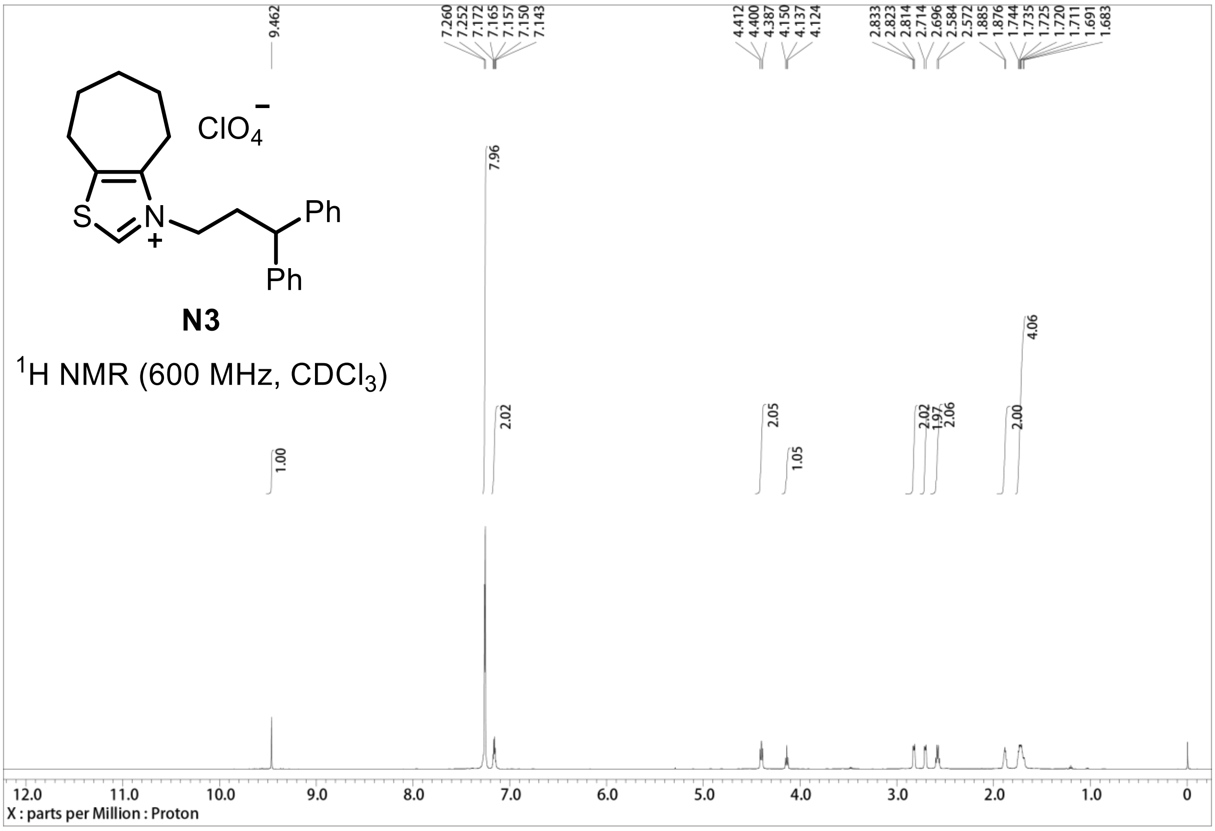

ClO4−
S
N
+
Ph
Ph
N3
1H NMR (600 MHz, CDCl3)
9.462
7.260 7.252 7.172 7.165 7.157 7.150 7.143
4.412 4.400 4.387 4.150 4.137 4.124
2.833 2.823 2.814 2.714 2.696 2.584 2.572 1.885 1.876 1.744 1.735 1.725 1.720 1.711 1.691 1.683
1.00
7.96
2.02
2.05
1.05
2.02 1.97 2.06
2.00
4.06
X : parts per Million : Proton

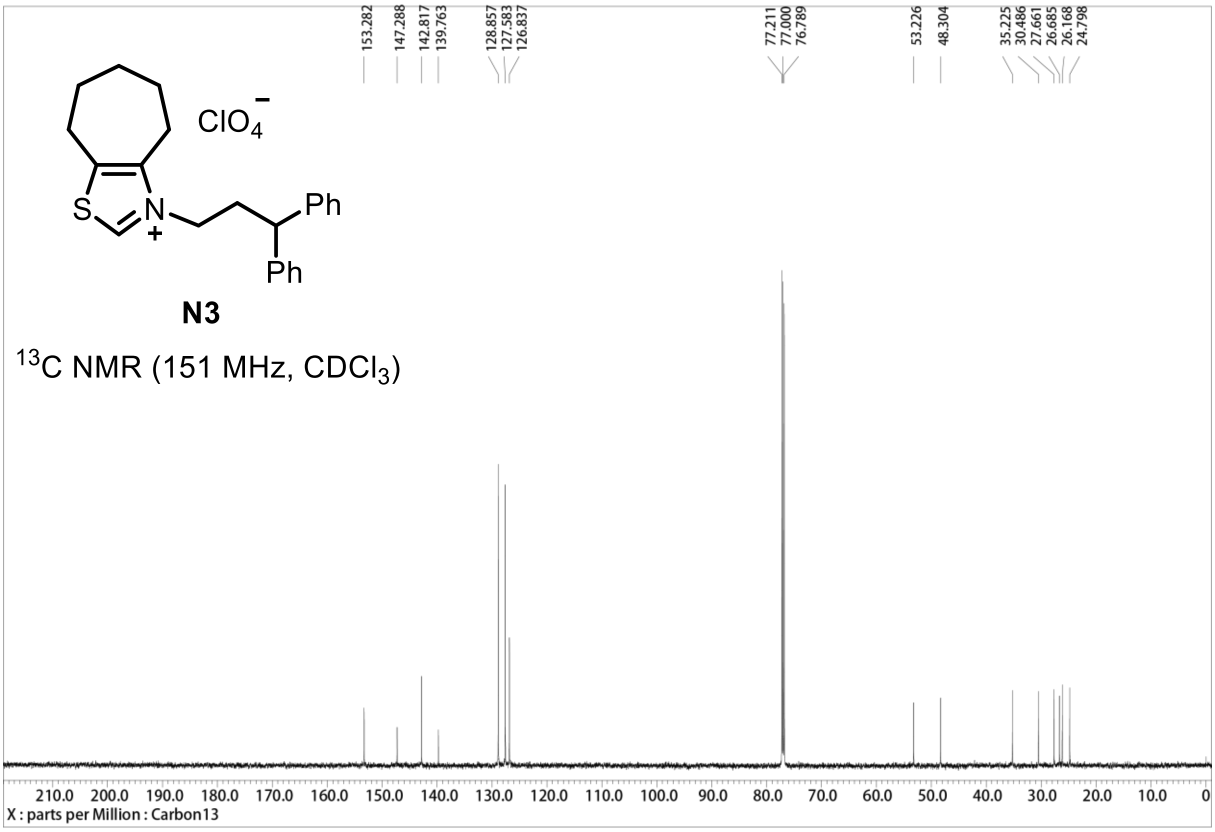

ClO4−
S
N
+
Ph
Ph
N3
13C NMR (151 MHz, CDCl3)
153.282 147.288 142.817 139.763
128.857 127.583 126.837
77.211 77.000 76.789
53.226 48.304
35.225 30.486 27.661 26.685 26.168 24.798
X : parts per Million : Carbon13

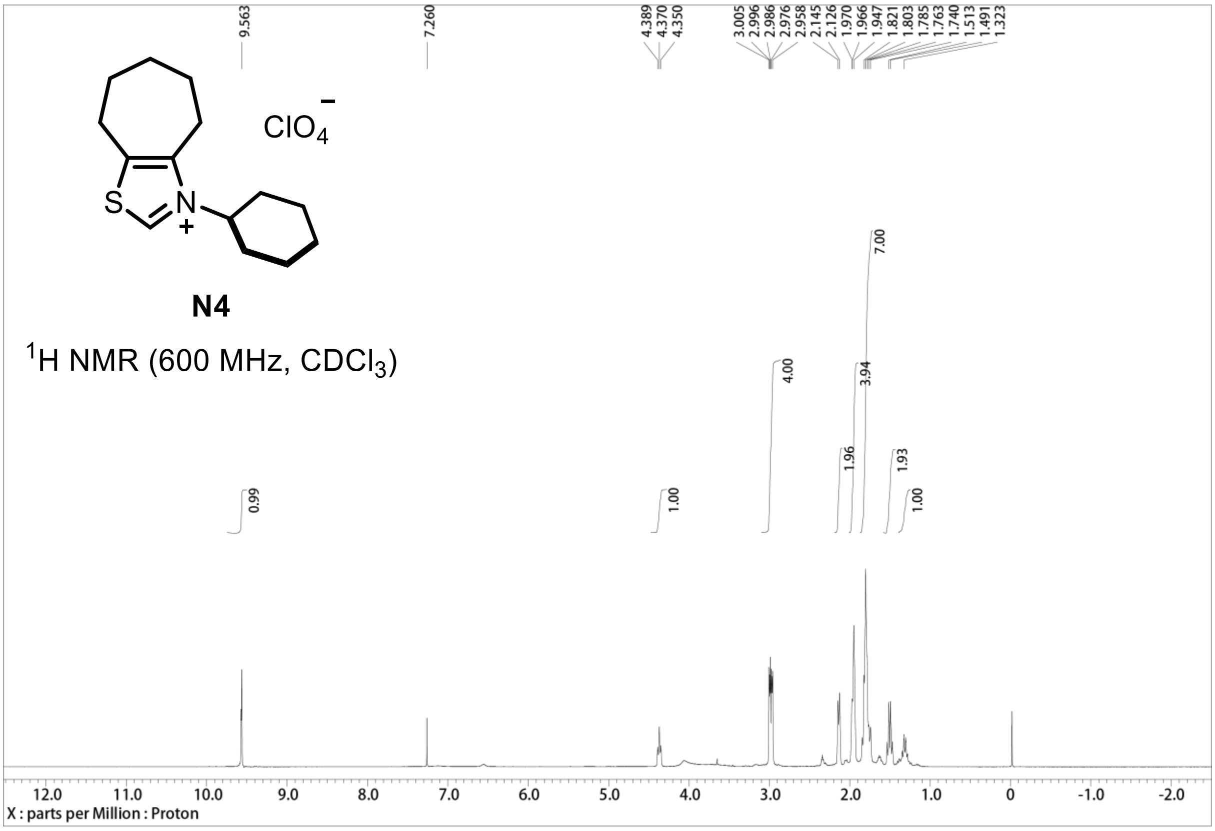

ClO4
S
N
N4
1H NMR (600 MHz, CDCl3)
9.563
7.260
4.389
4.370
4.350
3.005
2.996
2.986
2.976
2.958
2.145
2.126
1.970
1.966
1.947
1.821
1.803
1.785
1.763
1.740
1.513
1.491
1.323
0.99
1.00
4.00
1.96
3.94
7.00
1.93
1.00
X : parts per Million : Proton

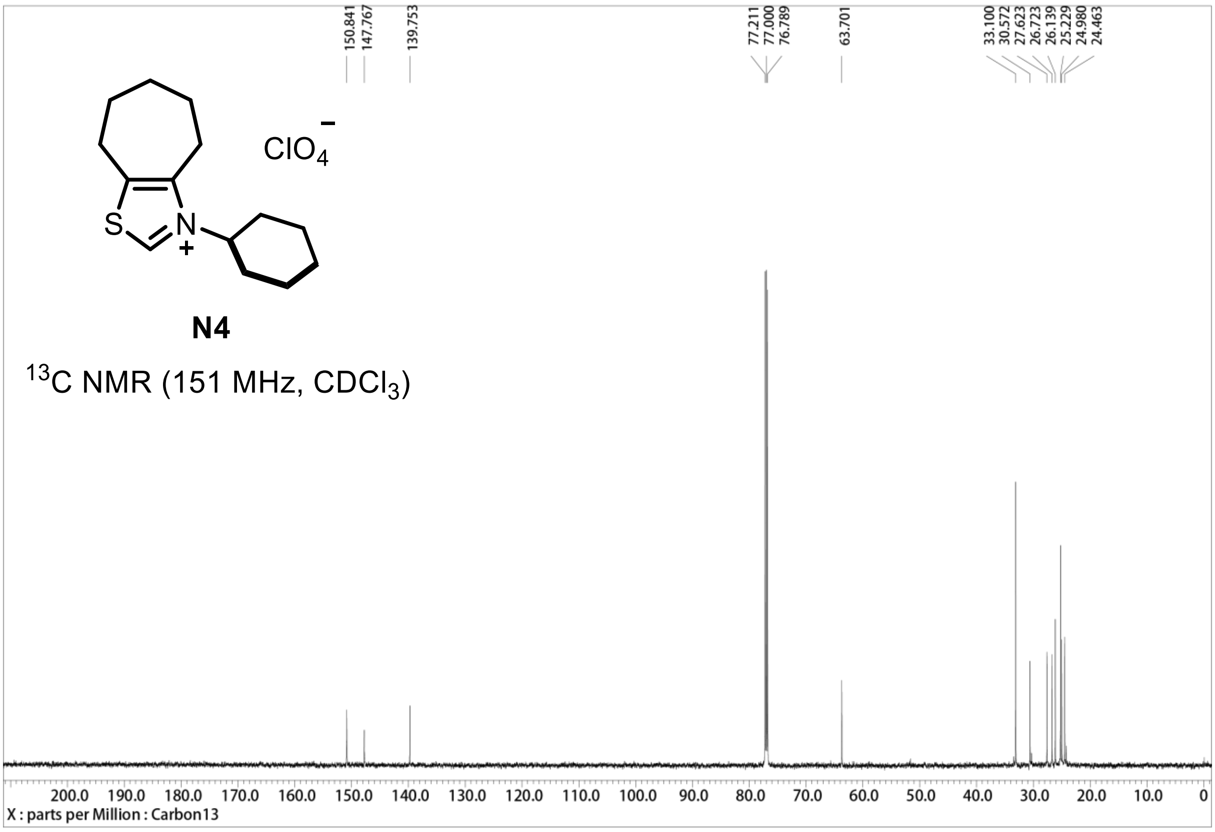

ClO4
S
N
N4
13C NMR (151 MHz, CDCl3)
150.841
147.767
139.753
77.211
77.000
76.789
63.701
33.100
30.572
27.623
26.723
26.139
25.229
24.980
24.463
X : parts per Million : Carbon13

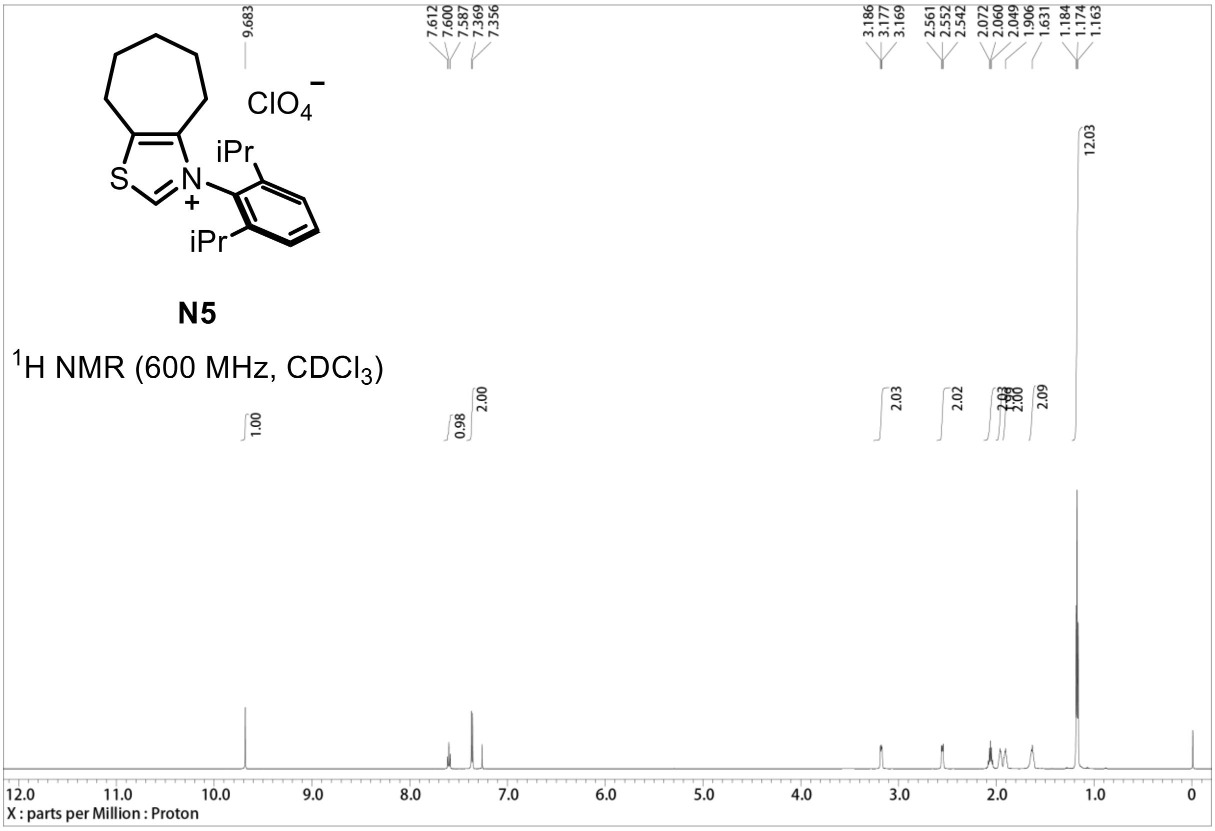
ClO4
iPr
S
N
+
iPr
N5
1H NMR (600 MHz, CDCl3)
9.683
7.612
7.600
7.587
7.369
7.356
3.186
3.177
3.169
2.561
2.552
2.542
2.072
2.060
2.049
1.906
1.631
1.184
1.174
1.163
12.03
1.00
0.98
2.00
2.03
2.02
2.09
12.0 11.0 10.0 9.0 8.0 7.0 6.0 5.0 4.0 3.0 2.0 1.0 0
X : parts per Million : Proton

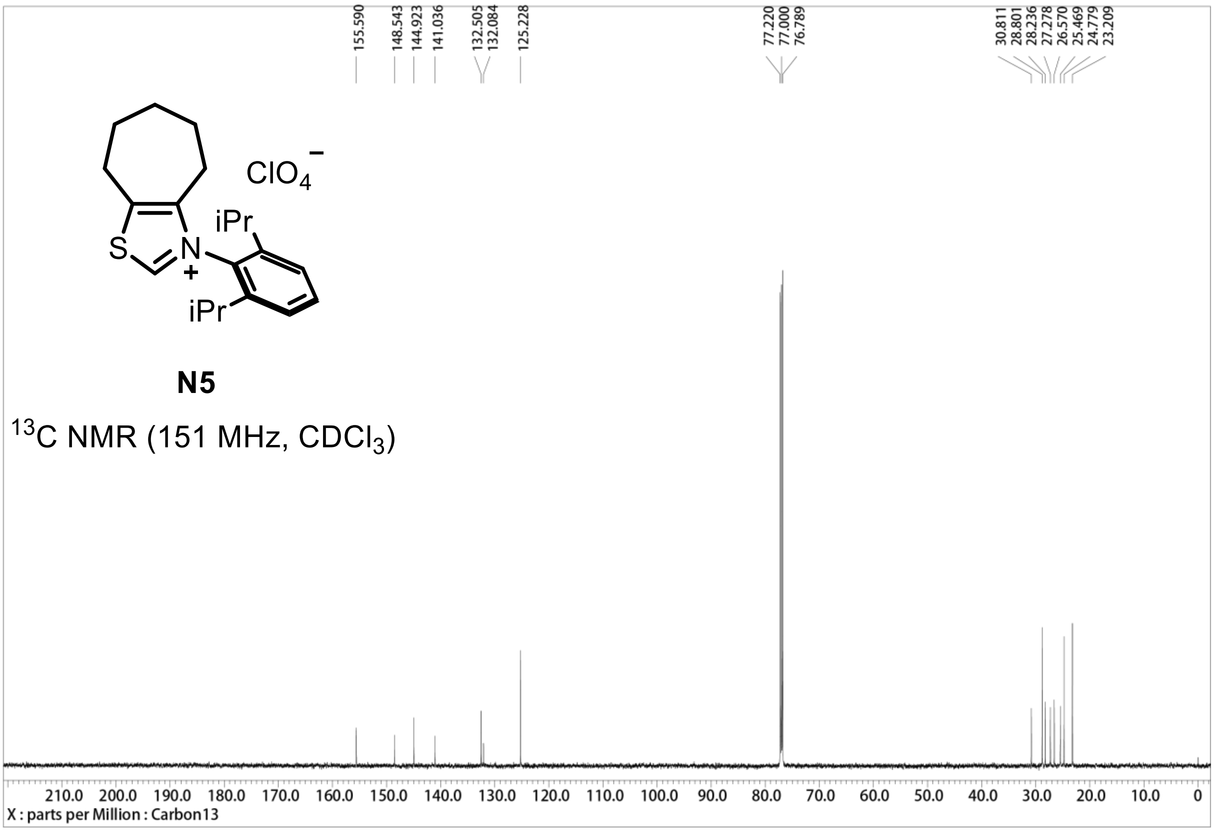
ClO4
iPr
S
N
+
iPr
N5
13C NMR (151 MHz, CDCl3)
155.590
148.543
144.923
141.036
132.505
132.084
125.228
77.220
77.000
76.789
30.811
28.801
28.236
27.278
26.570
25.469
24.779
23.209
210.0 200.0 190.0 180.0 170.0 160.0 150.0 140.0 130.0 120.0 110.0 100.0 90.0 80.0 70.0 60.0 50.0 40.0 30.0 20.0 10.0 0
X : parts per Million : Carbon13

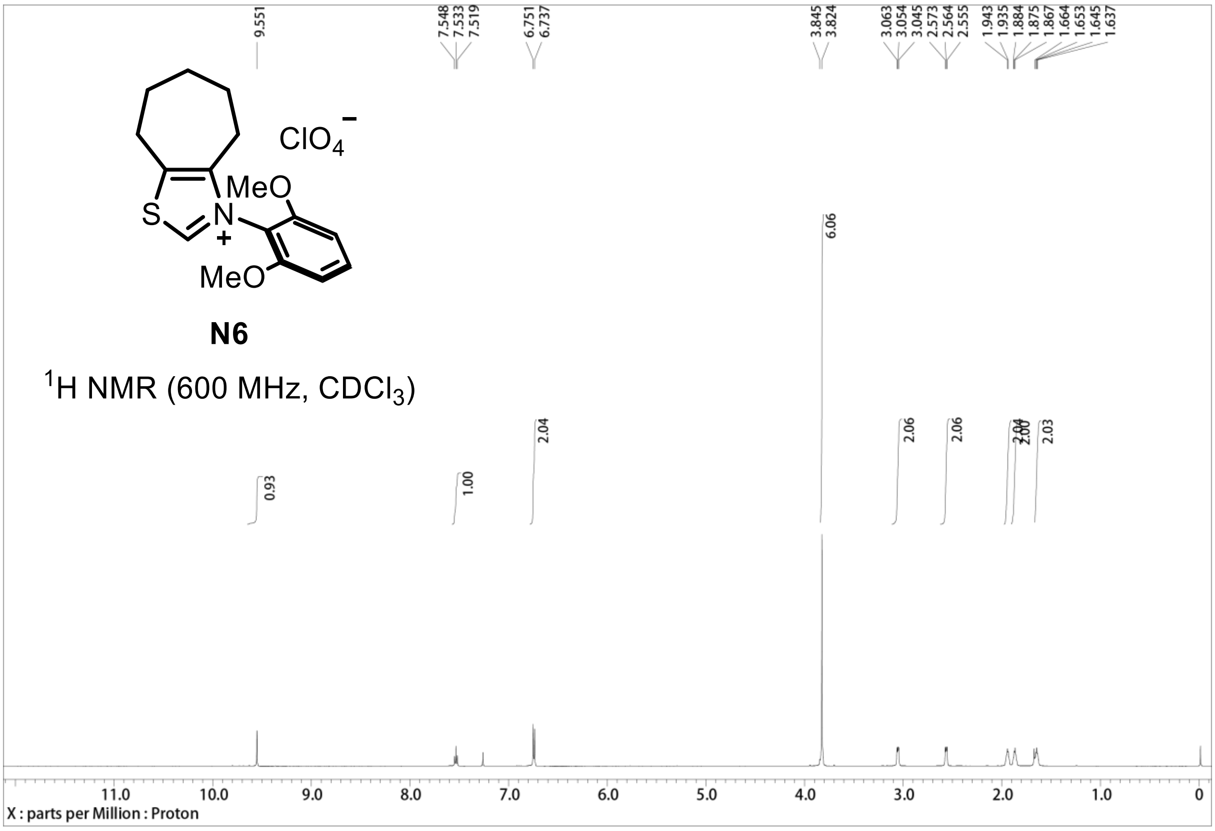
9.551
7.548
7.533
7.519
6.751
6.737
3.845
3.824
3.063
3.054
3.045
2.573
2.564
2.555
1.943
1.935
1.884
1.875
1.867
1.664
1.653
1.645
1.637
ClO4−
MeO
S
N+
MeO
N6
1H NMR (600 MHz, CDCl3)
0.93
1.00
2.04
6.06
2.06
2.06
2.04
2.07
2.03
11.0
10.0
9.0
8.0
7.0
6.0
5.0
4.0
3.0
2.0
1.0
0
X : parts per Million : Proton

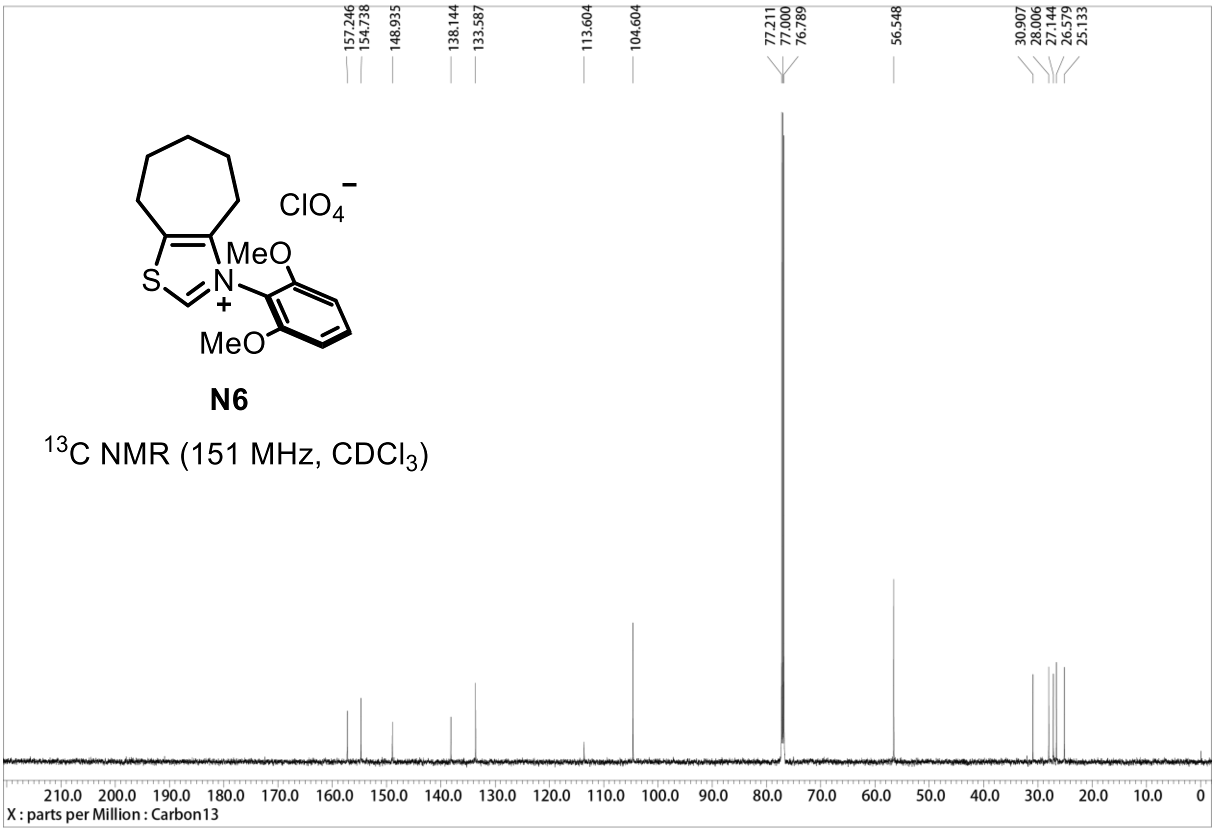
157.246
154.738
148.935
138.144
133.587
113.604
104.604
77.211
77.000
76.789
56.548
30.907
28.006
27.144
26.579
25.133
ClO4−
MeO
S
N+
MeO
N6
13C NMR (151 MHz, CDCl3)
210.0
200.0
190.0
180.0
170.0
160.0
150.0
140.0
130.0
120.0
110.0
100.0
90.0
80.0
70.0
60.0
50.0
40.0
30.0
20.0
10.0
0
X : parts per Million : Carbon13

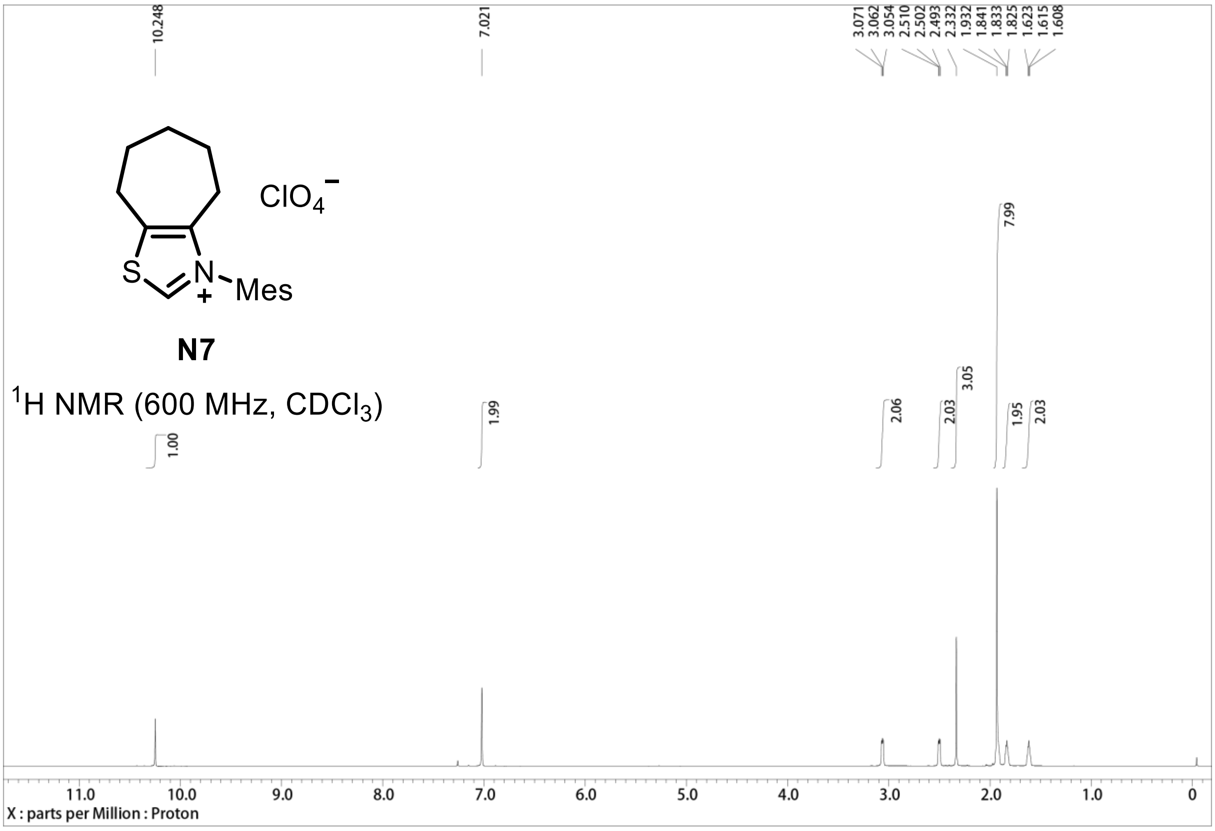
ClO4−
S
N
+
Mes
N7
1H NMR (600 MHz, CDCl3)
10.248
7.021
3.071
3.062
3.054
2.510
2.502
2.493
2.332
1.932
1.841
1.833
1.825
1.623
1.615
1.608
1.00
1.99
2.06
2.03
3.05
7.99
1.95
2.03
11.0
10.0
9.0
8.0
7.0
6.0
5.0
4.0
3.0
2.0
1.0
0
X : parts per Million : Proton

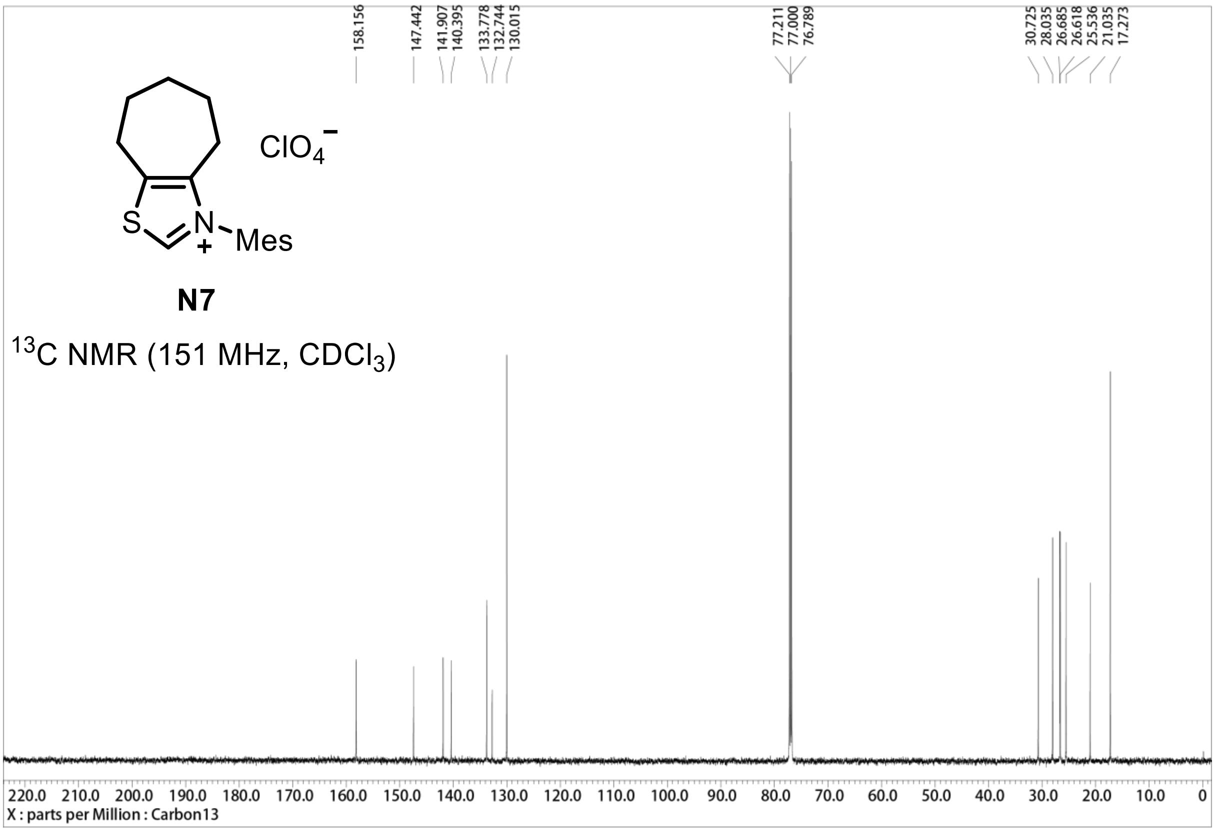
ClO4−
S
N
+
Mes
N7
13C NMR (151 MHz, CDCl3)
158.156
147.442
141.907
140.395
133.778
132.744
130.015
77.211
77.000
76.789
30.725
28.035
26.685
26.618
25.536
21.035
17.273
220.0
210.0
200.0
190.0
180.0
170.0
160.0
150.0
140.0
130.0
120.0
110.0
100.0
90.0
80.0
70.0
60.0
50.0
40.0
30.0
20.0
10.0
0
X : parts per Million : Carbon13

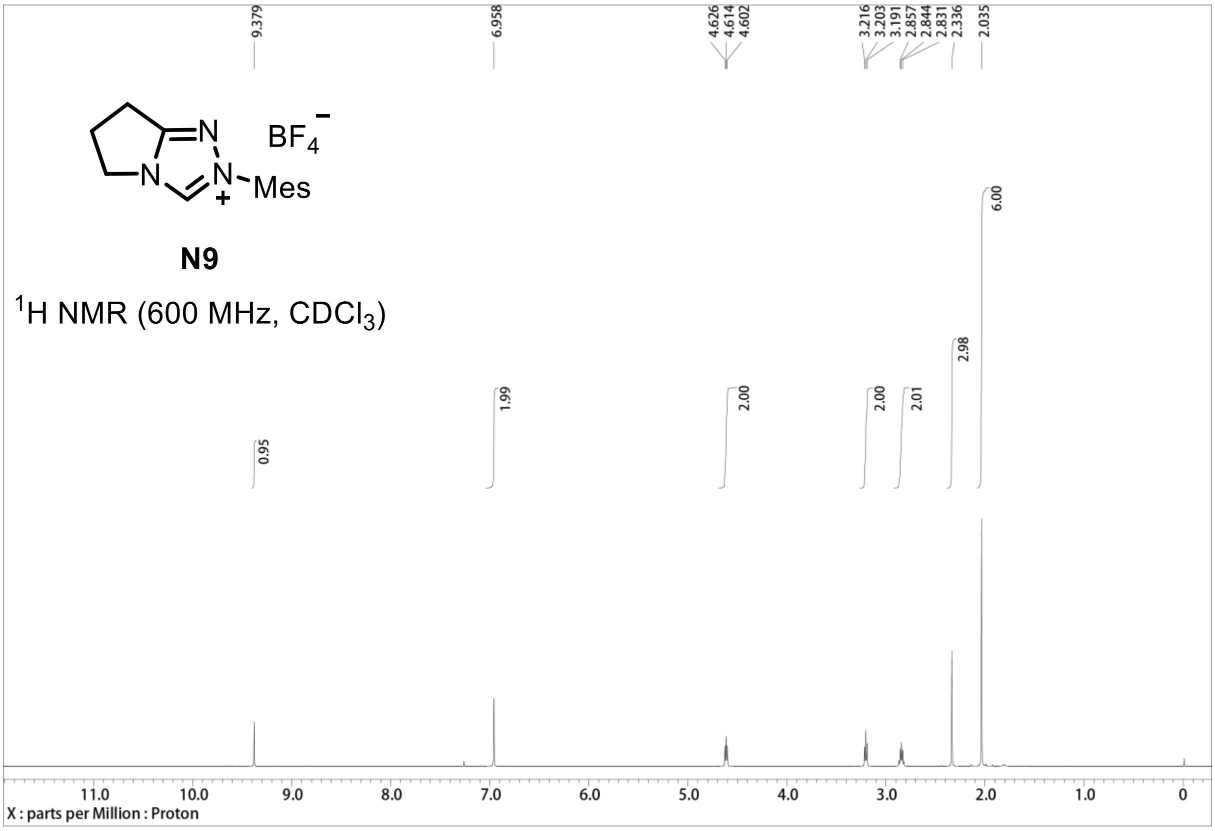
9.379
6.958
4.626
4.614
4.602
3.216
3.203
3.191
2.857
2.844
2.831
2.336
2.035
BF4
N
Mes
N9
1H NMR (600 MHz, CDCl3)
0.95
1.99
2.00
2.00
2.01
2.98
6.00
11.0 10.0 9.0 8.0 7.0 6.0 5.0 4.0 3.0 2.0 1.0 0
X : parts per Million : Proton

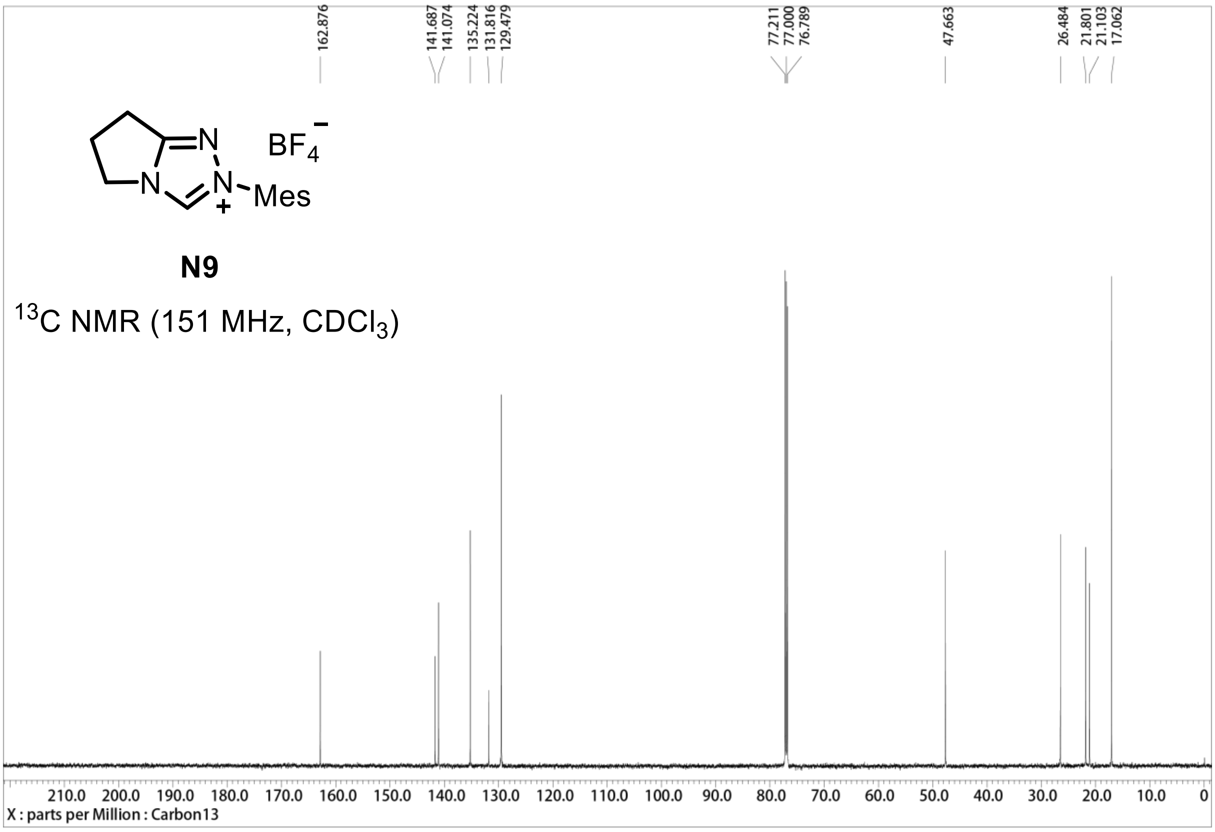
162.876
141.687
141.074
135.224
131.816
129.479
77.211
77.000
76.789
47.663
26.484
21.801
21.103
17.062
BF4
N
Mes
N9
13C NMR (151 MHz, CDCl3)
210.0 200.0 190.0 180.0 170.0 160.0 150.0 140.0 130.0 120.0 110.0 100.0 90.0 80.0 70.0 60.0 50.0 40.0 30.0 20.0 10.0 0
X : parts per Million : Carbon13

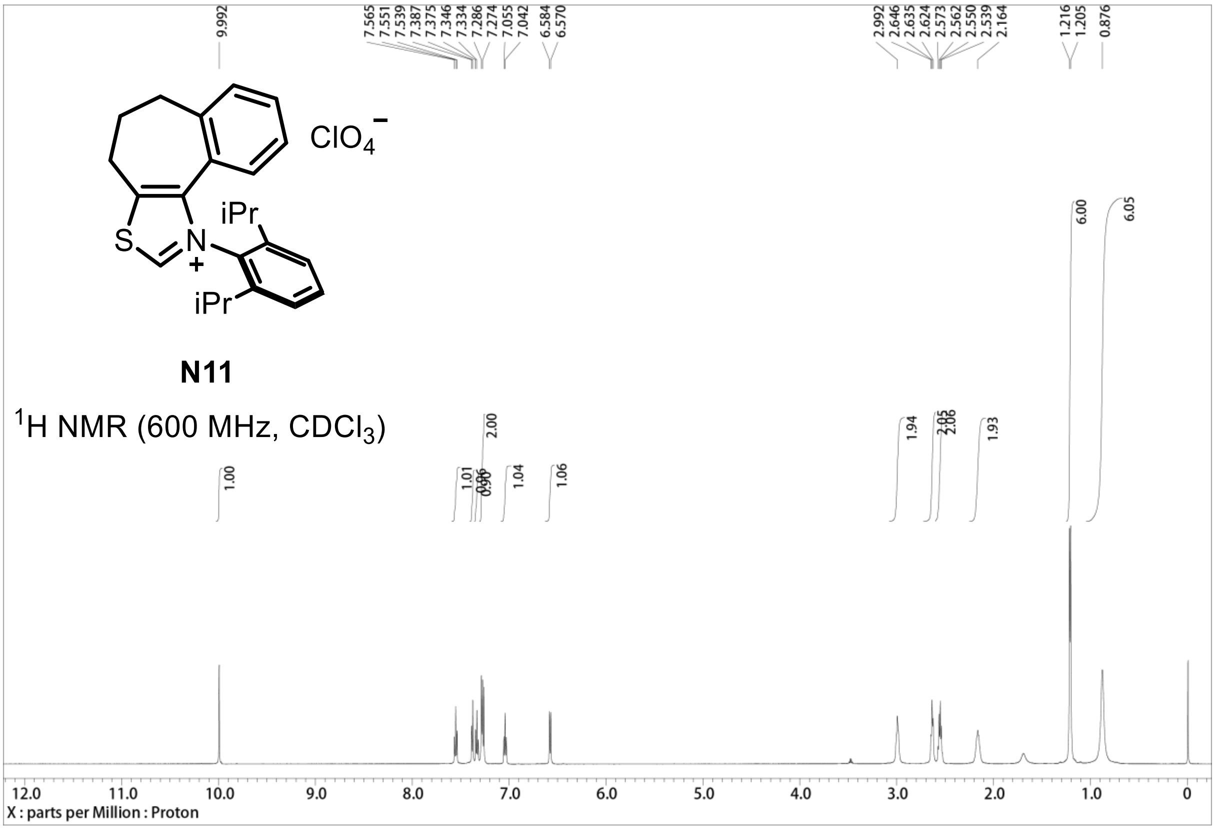

N11

^{1}H NMR (600 MHz, $CDCl_3$)

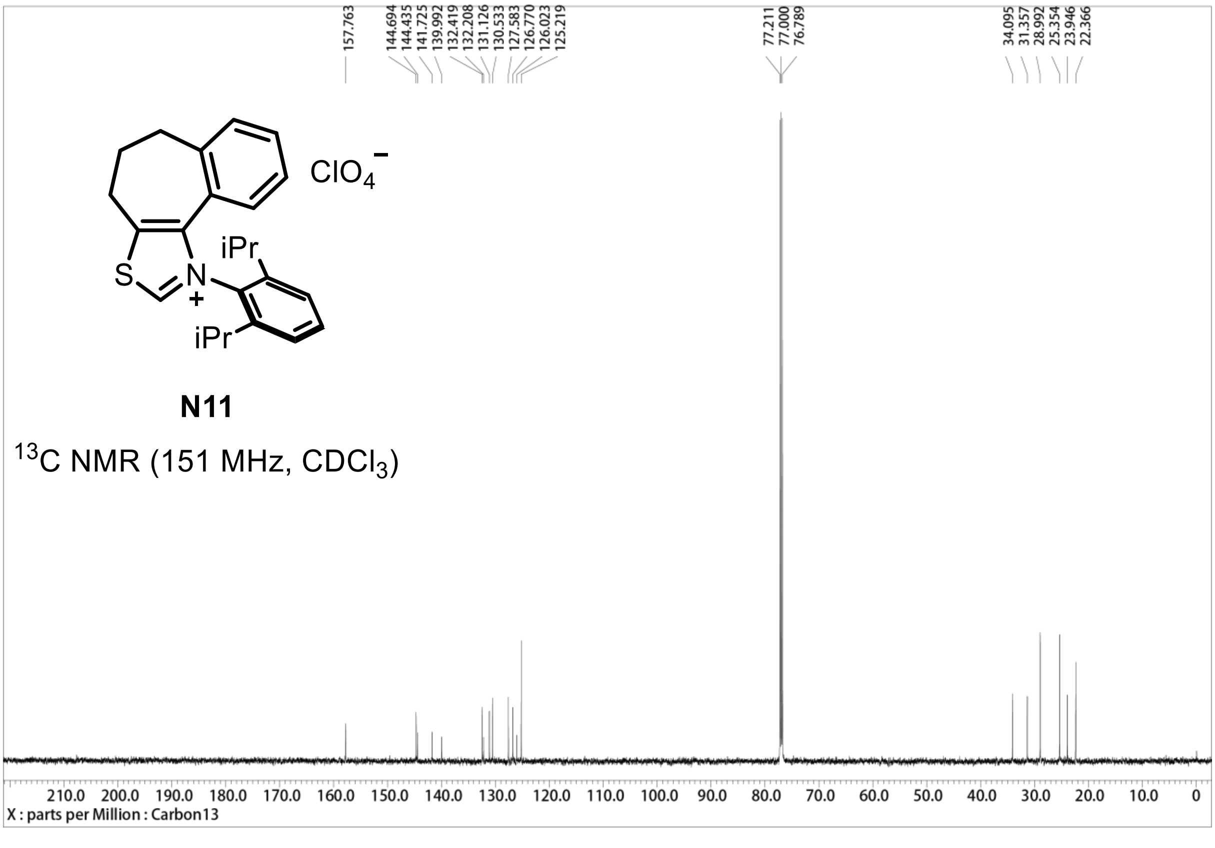

N11

^{13}C NMR (151 MHz, $CDCl_3$)

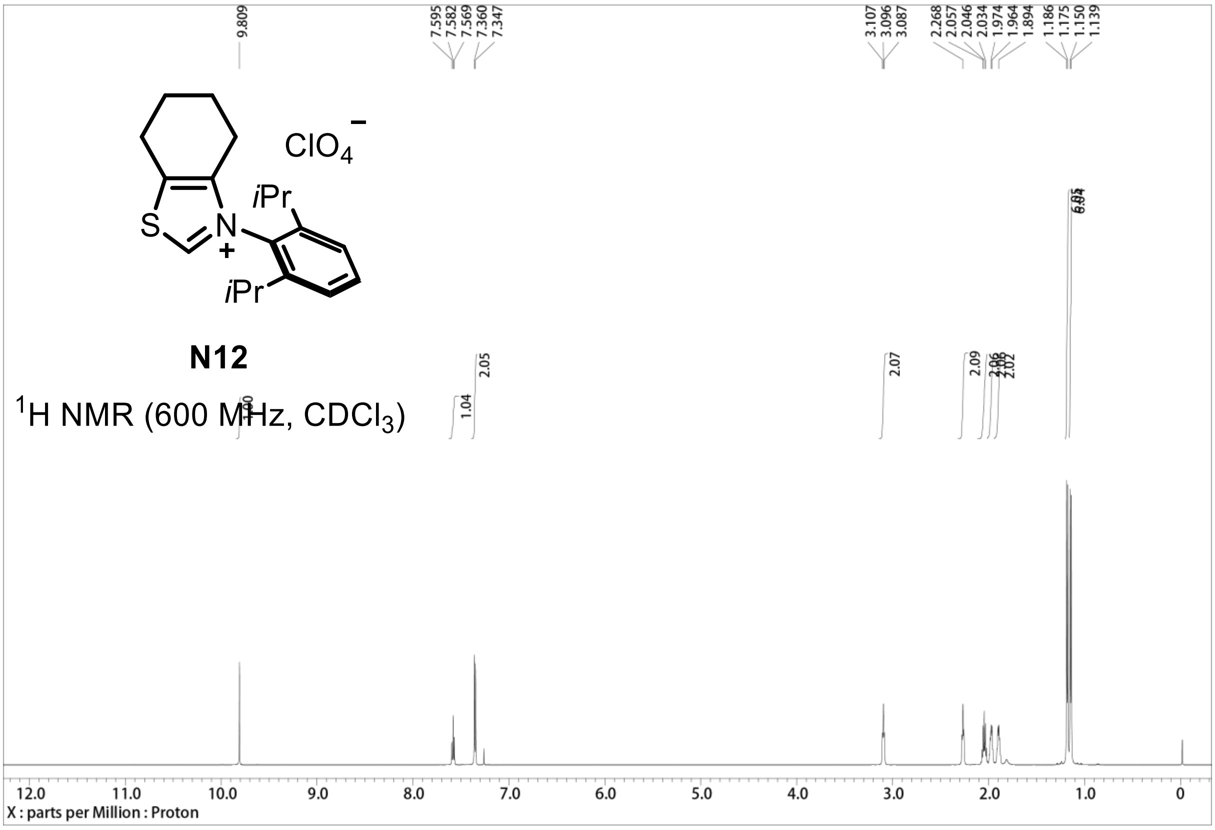

ClO4
iPr
S
N
iPr
N12
1H NMR (600 MHz, CDCl3)
9.809
7.595
7.582
7.569
7.360
7.347
3.107
3.096
3.087
2.268
2.057
2.046
2.034
1.974
1.964
1.894
1.186
1.175
1.150
1.139
1.04
2.05
2.07
2.09
2.02
12.0
11.0
10.0
9.0
8.0
7.0
6.0
5.0
4.0
3.0
2.0
1.0
0
X : parts per Million : Proton

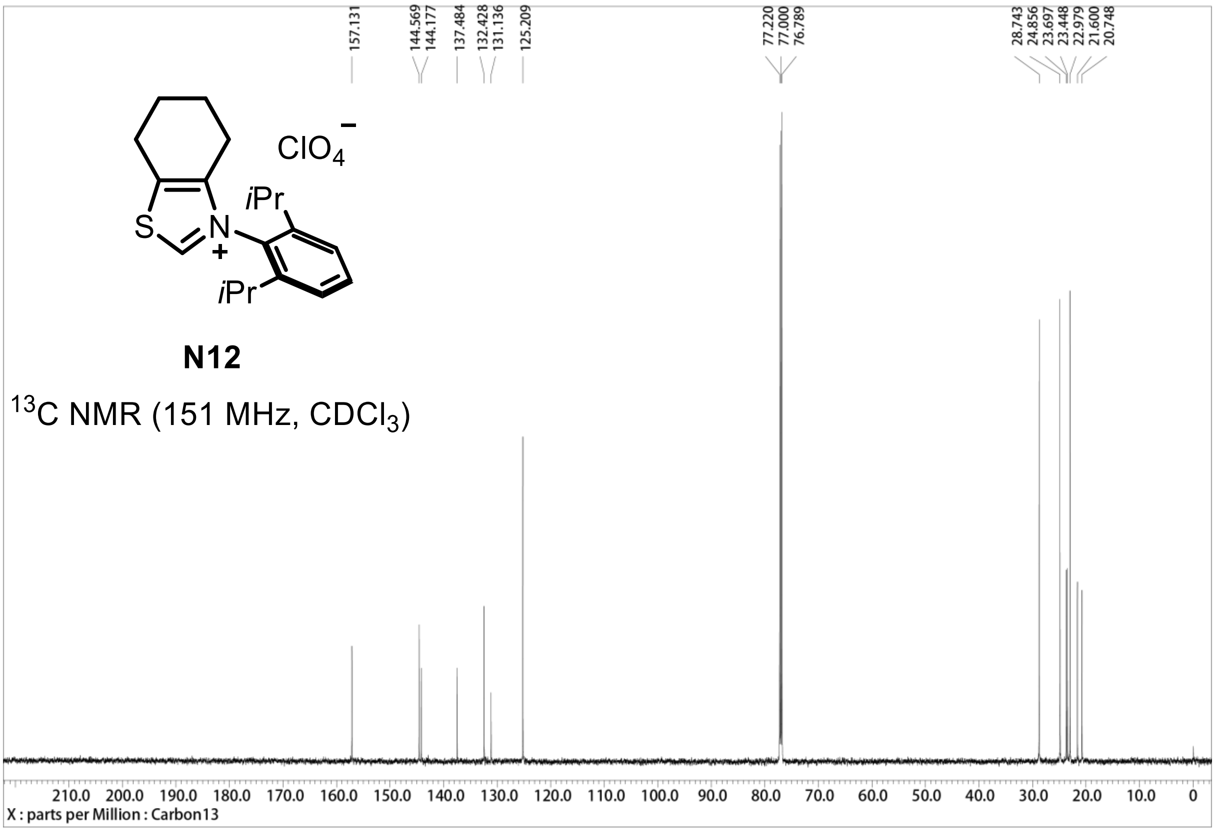

ClO4
iPr
S
N
iPr
N12
13C NMR (151 MHz, CDCl3)
157.131
144.569
144.177
137.484
132.428
131.136
125.209
77.220
77.000
76.789
28.743
24.856
23.697
23.448
22.979
21.600
20.748
210.0
200.0
190.0
180.0
170.0
160.0
150.0
140.0
130.0
120.0
110.0
100.0
90.0
80.0
70.0
60.0
50.0
40.0
30.0
20.0
10.0
0
X : parts per Million : Carbon13

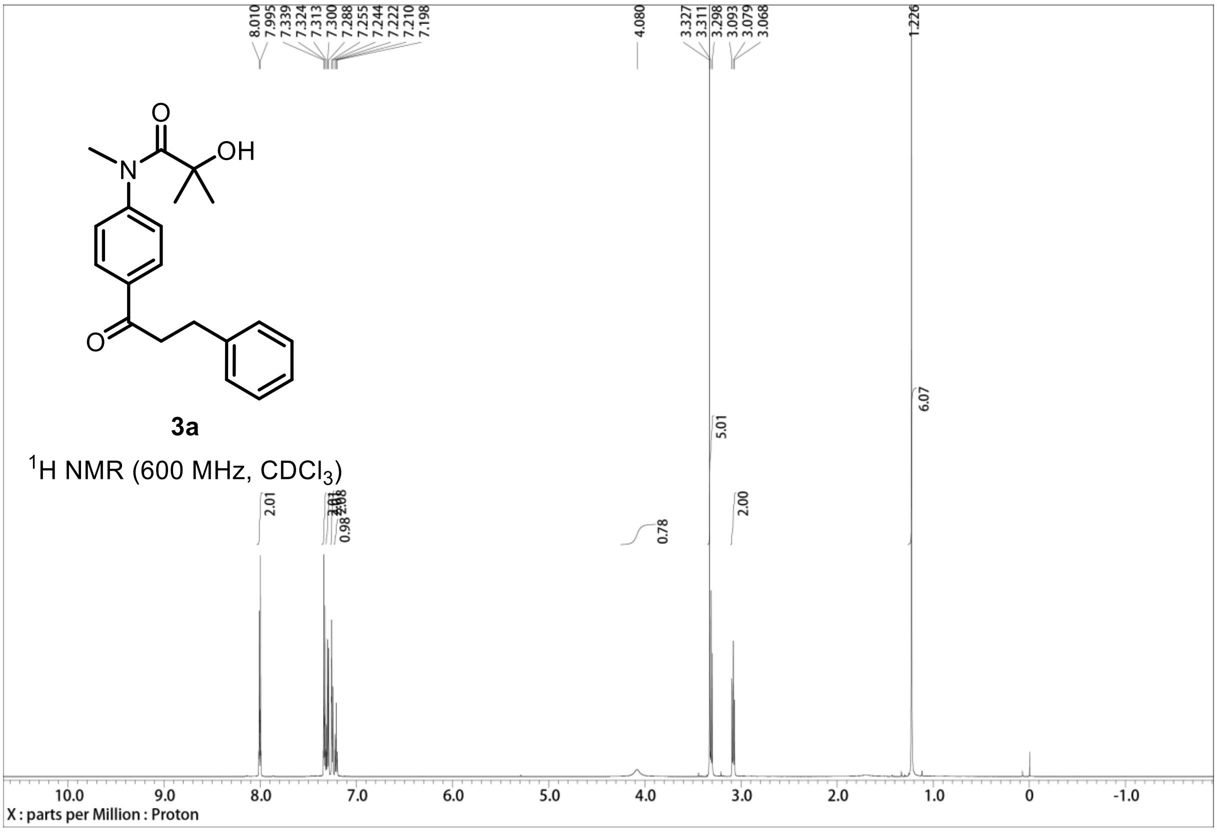
8.010
7.995
7.339
7.324
7.313
7.300
7.288
7.255
7.244
7.222
7.210
7.198
4.080
3.327
3.311
3.298
3.093
3.079
3.068
1.226
O
N
OH
O
3a
1H NMR (600 MHz, CDCl3)
2.01
0.98
0.78
5.01
2.00
6.07
10.0
9.0
8.0
7.0
6.0
5.0
4.0
3.0
2.0
1.0
0
-1.0
X : parts per Million : Proton

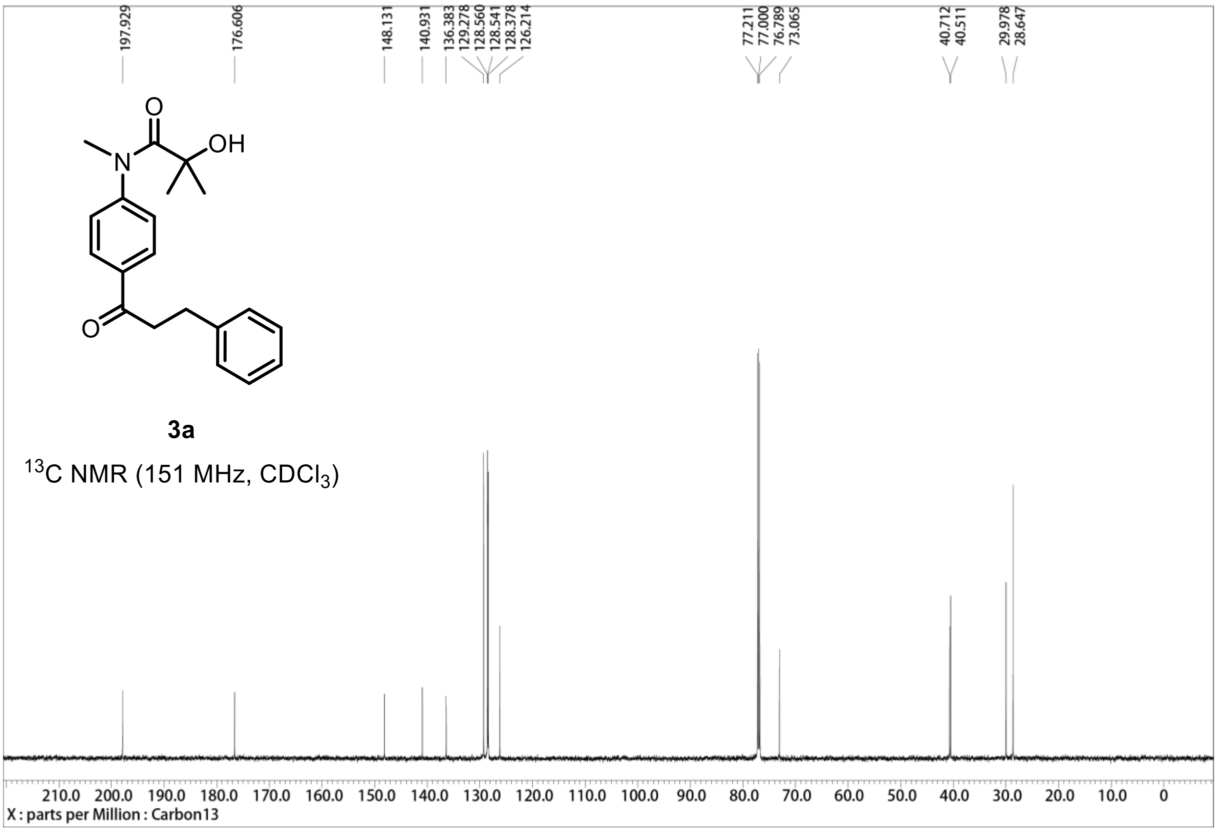
197.929
176.606
148.131
140.931
136.383
129.278
128.560
128.541
128.378
126.214
77.211
77.000
76.789
73.065
40.712
40.511
29.978
28.647
O
N
OH
O
3a
13C NMR (151 MHz, CDCl3)
210.0
200.0
190.0
180.0
170.0
160.0
150.0
140.0
130.0
120.0
110.0
100.0
90.0
80.0
70.0
60.0
50.0
40.0
30.0
20.0
10.0
0
X : parts per Million : Carbon13

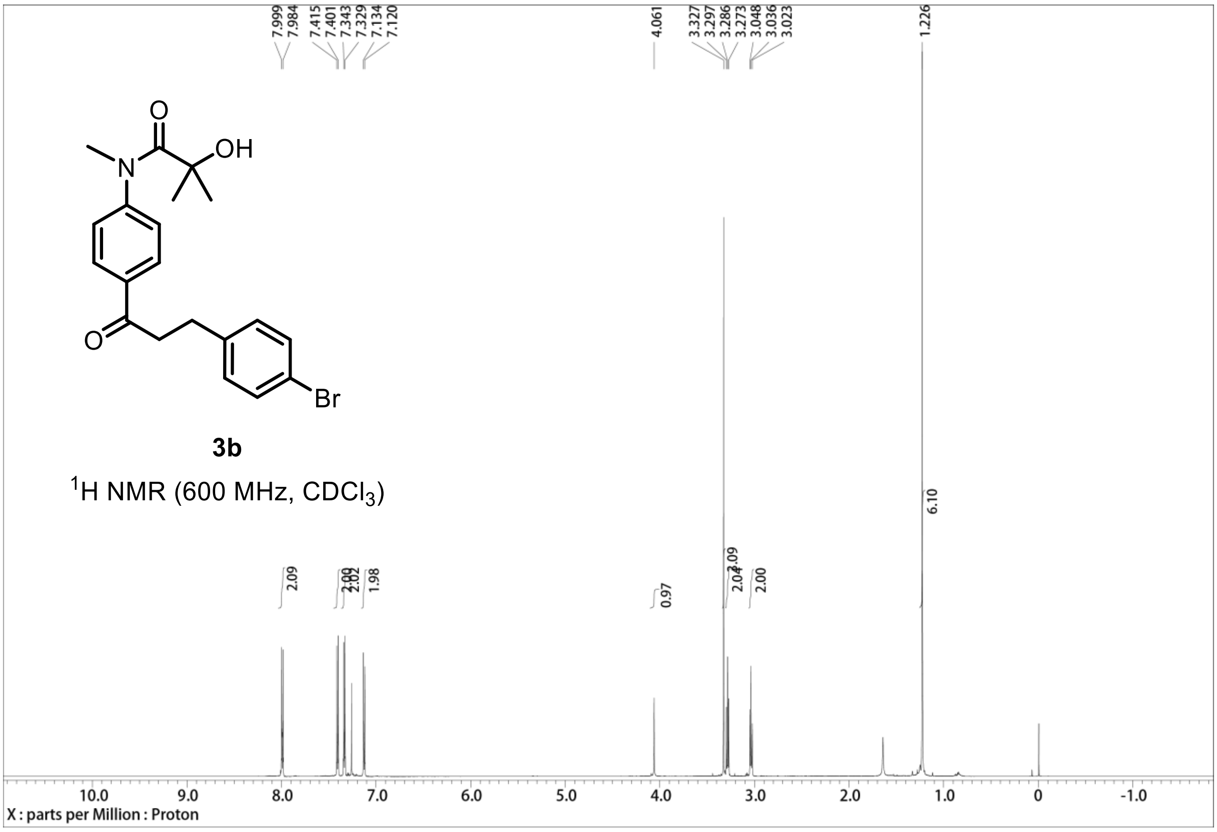
7.999
7.984
7.415
7.401
7.343
7.329
7.134
7.120
4.061
3.327
3.297
3.286
3.273
3.048
3.036
3.023
1.226
OH
O
N
O
Br
3b
^{1}H NMR (600 MHz, CDCl$_3$)
2.09
1.98
0.97
2.00
6.10
10.0
9.0
8.0
7.0
6.0
5.0
4.0
3.0
2.0
1.0
0
-1.0
X : parts per Million : Proton

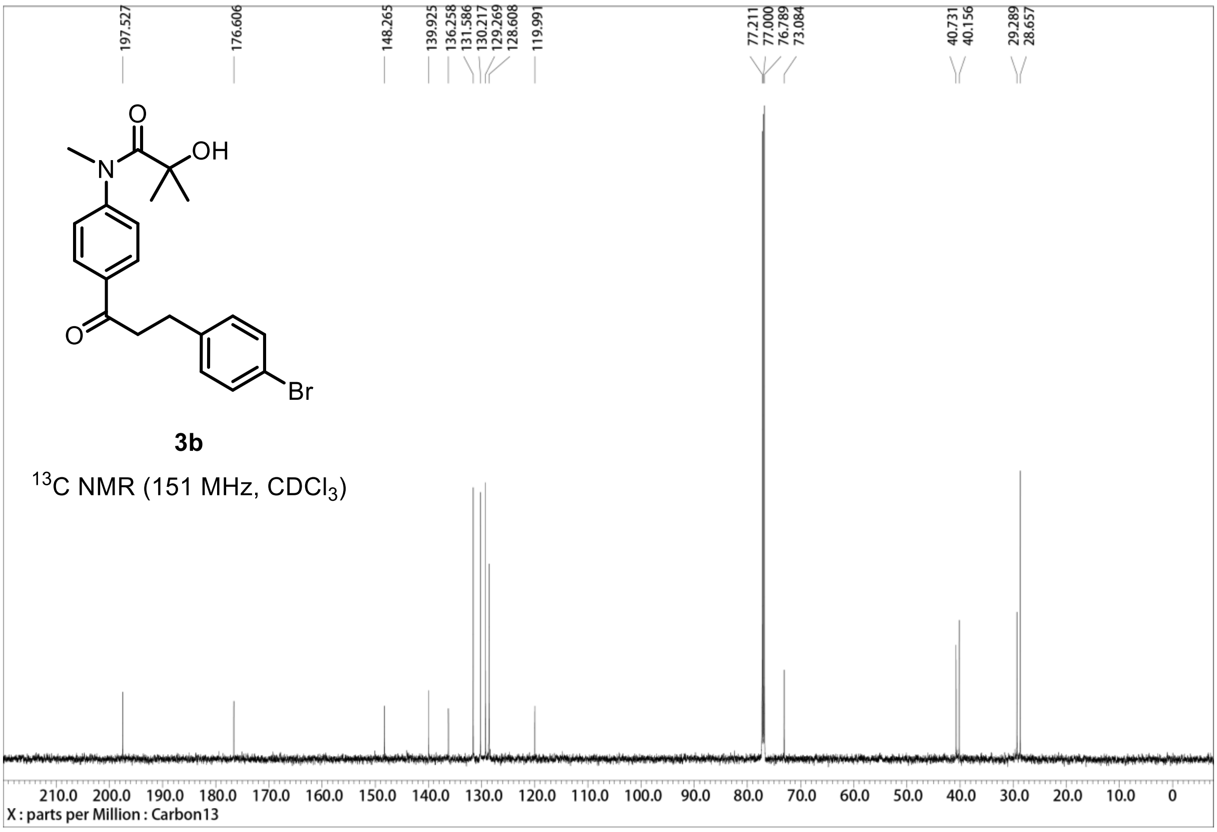
197.527
176.606
148.265
139.925
136.258
131.586
130.217
129.269
128.608
119.991
77.211
77.000
76.789
73.084
40.731
40.156
29.289
28.657
OH
O
N
O
Br
3b
^{13}C NMR (151 MHz, CDCl$_3$)
210.0
200.0
190.0
180.0
170.0
160.0
150.0
140.0
130.0
120.0
110.0
100.0
90.0
80.0
70.0
60.0
50.0
40.0
30.0
20.0
10.0
0
X : parts per Million : Carbon13

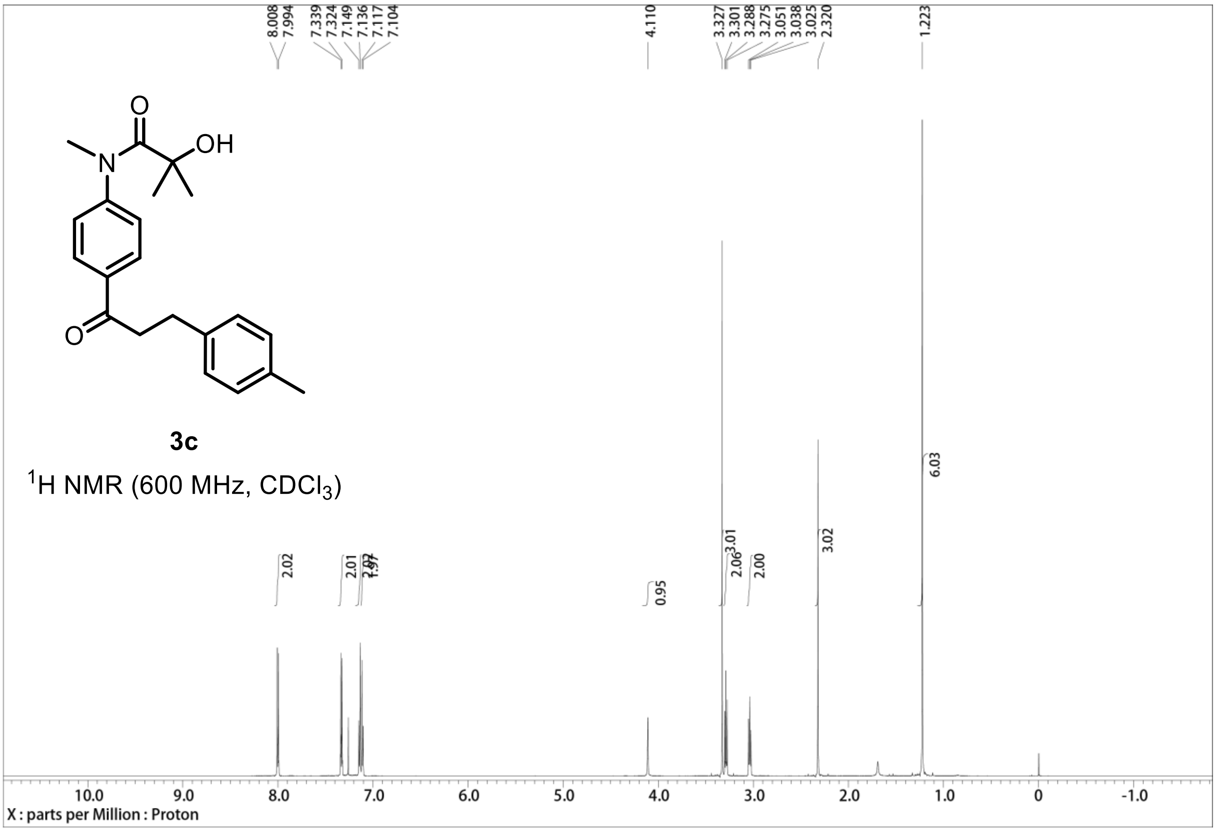

3c
^{1}H NMR (600 MHz, CDCl$_3$)
X : parts per Million : Proton

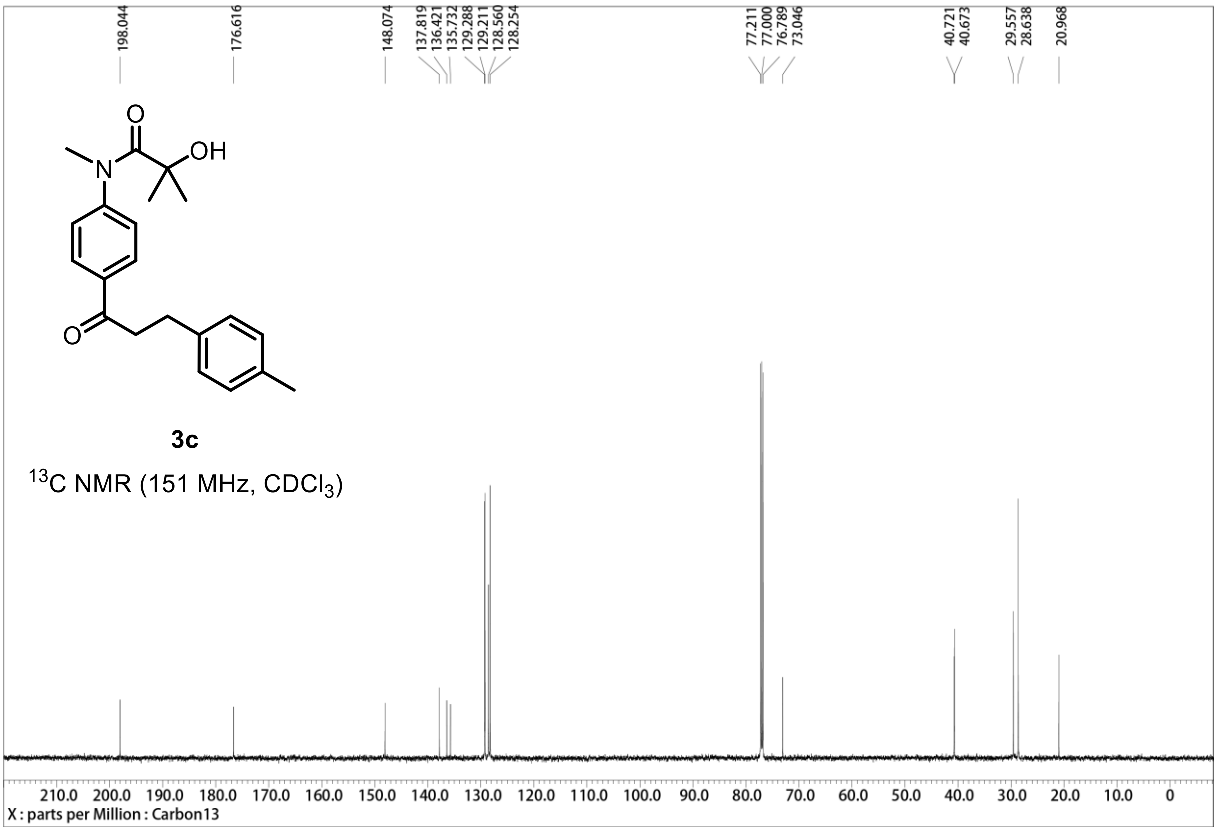

3c
^{13}C NMR (151 MHz, CDCl$_3$)
X : parts per Million : Carbon13

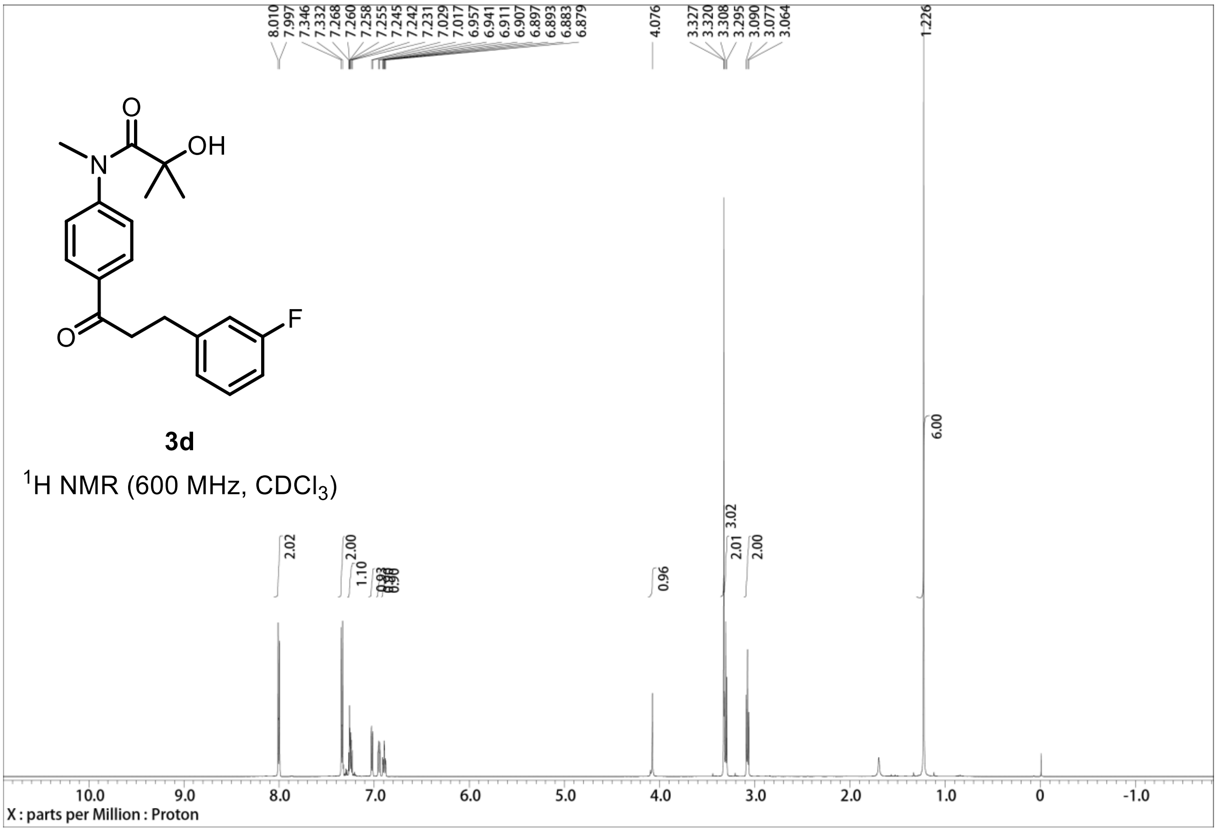
8.010 7.997 7.346 7.332 7.268 7.260 7.258 7.255 7.245 7.242 7.231 7.029 7.017 6.957 6.941 6.911 6.907 6.897 6.893 6.883 6.879
4.076
3.327 3.320 3.308 3.295 3.090 3.077 3.064
1.226
OH
N
O
O
F
3d
1H NMR (600 MHz, CDCl3)
2.02
2.00
1.10
0.96
3.02
2.01
2.00
6.00
10.0 9.0 8.0 7.0 6.0 5.0 4.0 3.0 2.0 1.0 0 -1.0
X : parts per Million : Proton

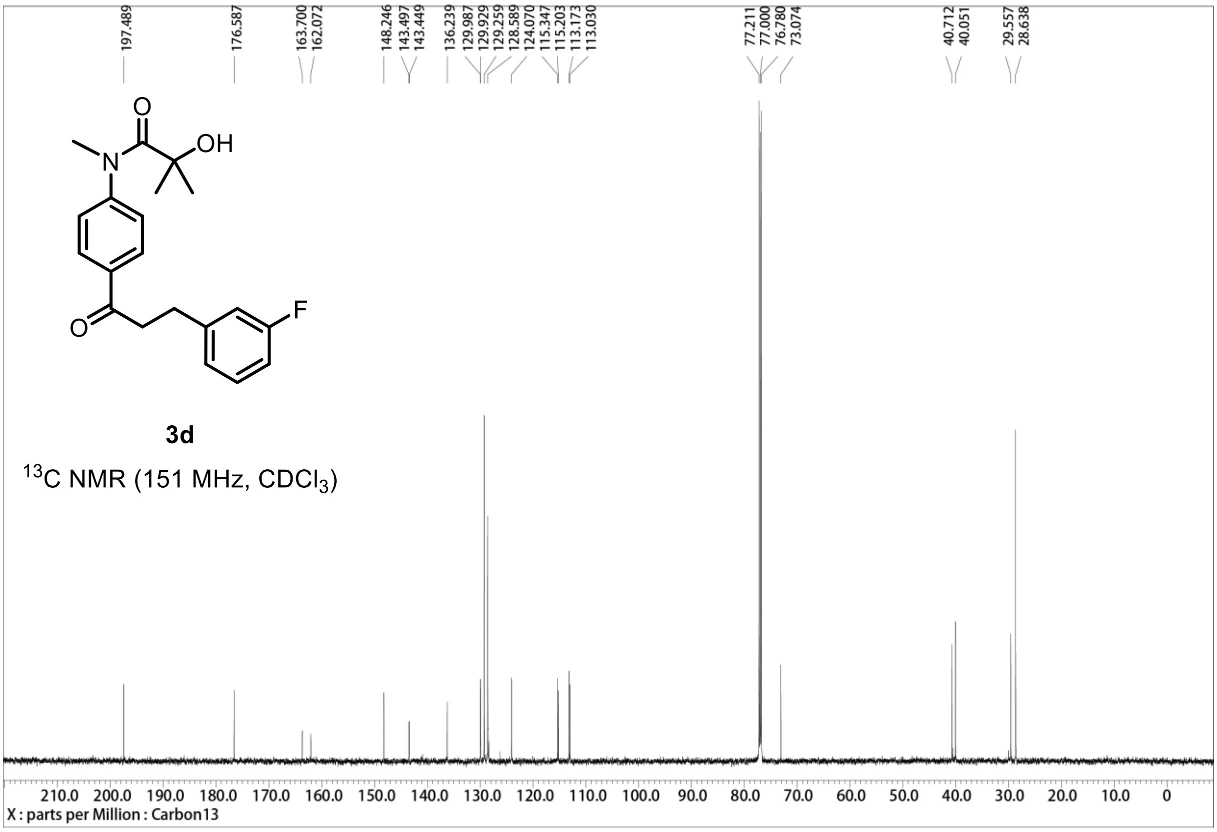
197.489
176.587
163.700 162.072
148.246 143.497 143.449
136.239 129.987 129.929 129.259 128.589 124.070 115.347 115.203 113.173 113.030
77.211 77.000 76.780 73.074
40.712 40.051
29.557 28.638
OH
N
O
O
F
3d
13C NMR (151 MHz, CDCl3)
210.0 200.0 190.0 180.0 170.0 160.0 150.0 140.0 130.0 120.0 110.0 100.0 90.0 80.0 70.0 60.0 50.0 40.0 30.0 20.0 10.0 0
X : parts per Million : Carbon13

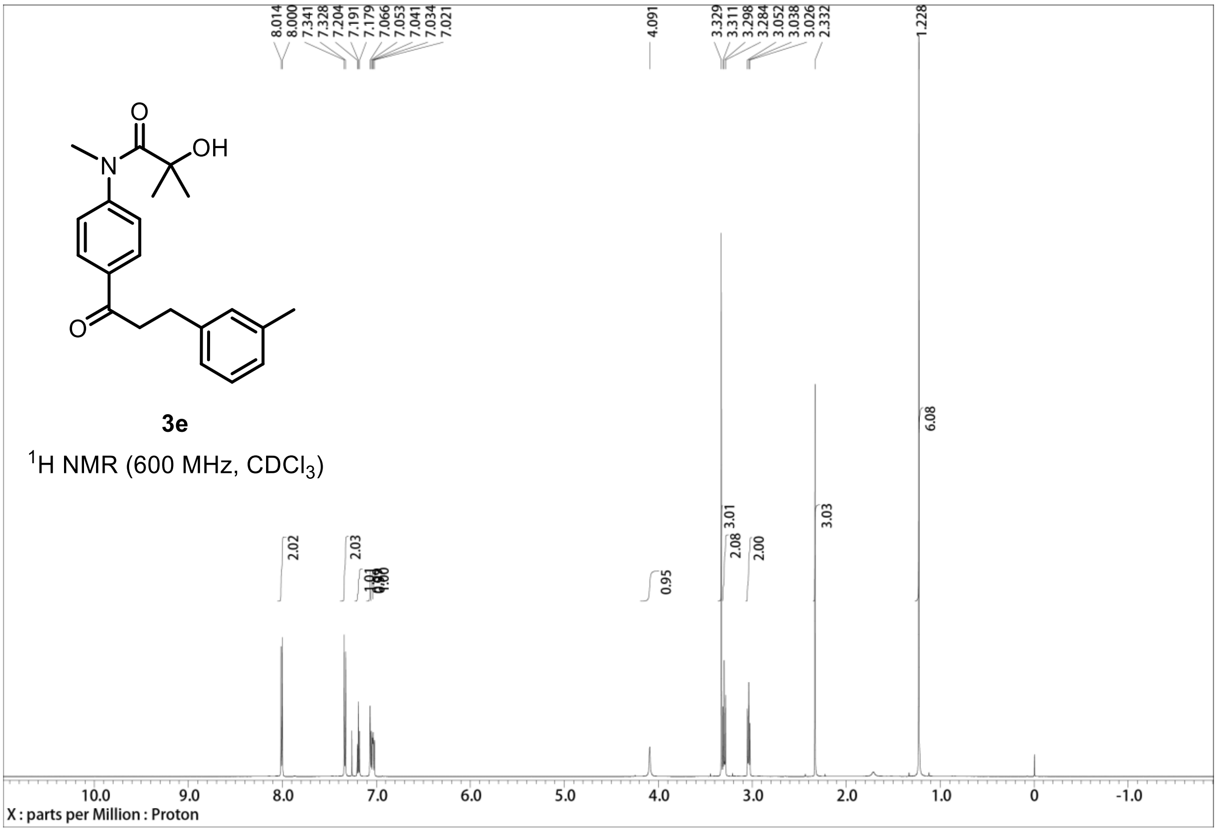

8.014
8.000
7.341
7.328
7.204
7.191
7.179
7.066
7.053
7.041
7.034
7.021
4.091
3.329
3.311
3.298
3.284
3.052
3.038
3.026
2.332
1.228
O
N
OH
O
3e
1H NMR (600 MHz, CDCl3)
2.02
2.03
0.95
3.01
2.08
2.00
3.03
6.08
X : parts per Million : Proton

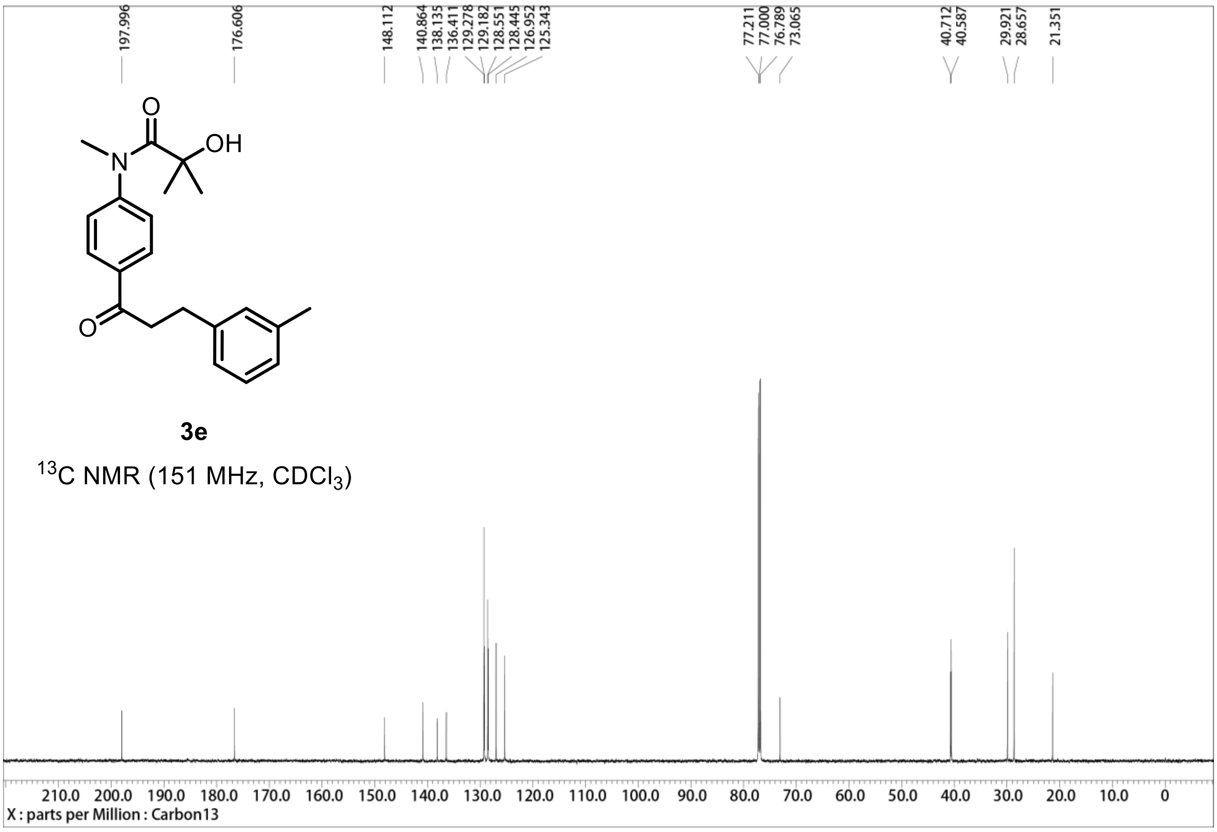

197.996
176.606
148.112
140.864
138.135
136.411
129.278
129.182
128.551
128.445
126.952
125.343
77.211
77.000
76.789
73.065
40.712
40.587
29.921
28.657
21.351
O
N
OH
O
3e
13C NMR (151 MHz, CDCl3)
X : parts per Million : Carbon13

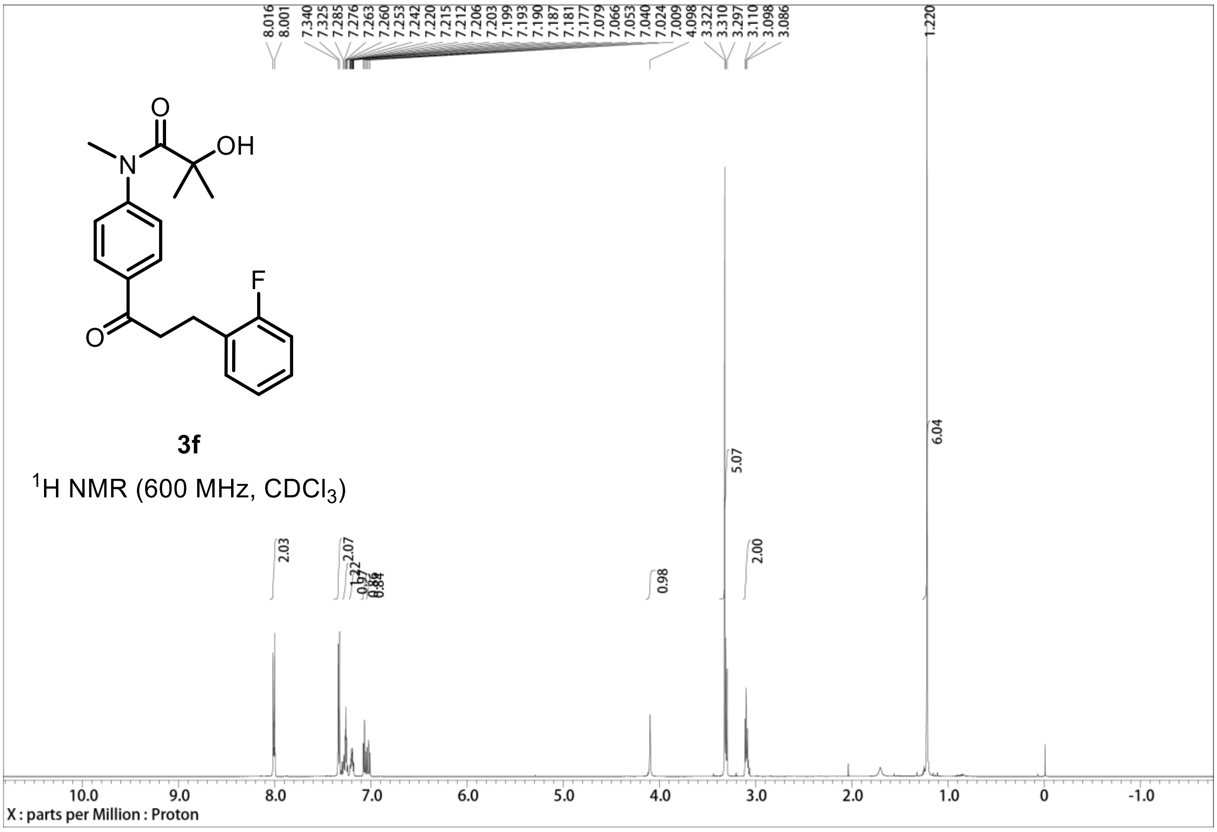
8.016
8.001
7.340
7.325
7.285
7.276
7.263
7.260
7.253
7.242
7.220
7.215
7.212
7.206
7.203
7.199
7.193
7.190
7.187
7.181
7.177
7.079
7.066
7.053
7.040
7.024
7.009
4.098
3.322
3.310
3.297
3.110
3.098
3.086
1.220
OH
N
O
F
O
3f
1H NMR (600 MHz, CDCl3)
2.03
2.07
0.98
5.07
2.00
6.04
10.0 9.0 8.0 7.0 6.0 5.0 4.0 3.0 2.0 1.0 0 -1.0
X : parts per Million : Proton

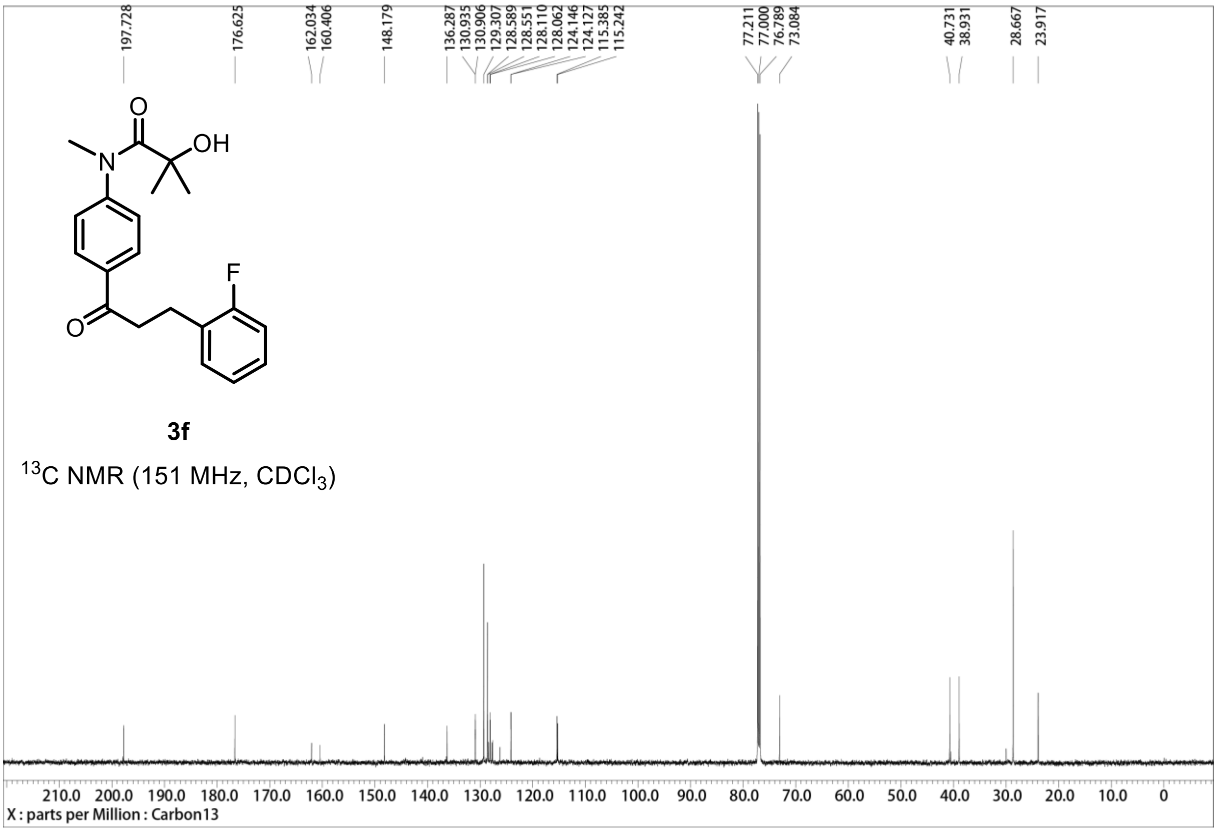
197.728
176.625
162.034
160.406
148.179
136.287
130.935
130.906
129.307
128.589
128.551
128.110
128.062
124.146
124.127
115.385
115.242
77.211
77.000
76.789
73.084
40.731
38.931
28.667
23.917
OH
N
O
F
O
3f
13C NMR (151 MHz, CDCl3)
210.0 200.0 190.0 180.0 170.0 160.0 150.0 140.0 130.0 120.0 110.0 100.0 90.0 80.0 70.0 60.0 50.0 40.0 30.0 20.0 10.0 0
X : parts per Million : Carbon13

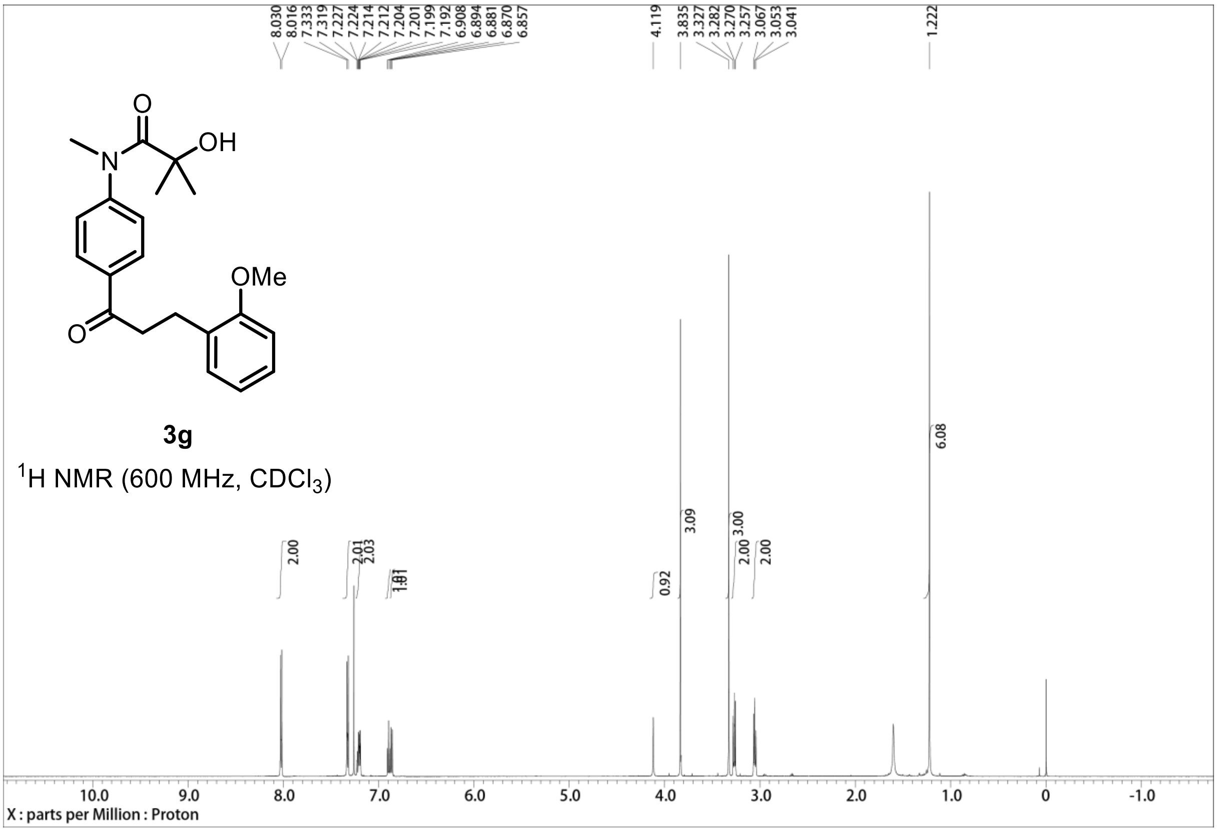
8.030
8.016
7.333
7.319
7.227
7.224
7.214
7.212
7.204
7.201
7.199
7.192
6.908
6.894
6.881
6.870
6.857
4.119
3.835
3.327
3.282
3.270
3.257
3.067
3.053
3.041
1.222
O
N
OH
OMe
O
3g
^{1}H NMR (600 MHz, CDCl$_3$)
2.00
2.01
2.03
3.09
0.92
3.00
2.00
2.00
6.08
10.0 9.0 8.0 7.0 6.0 5.0 4.0 3.0 2.0 1.0 0 -1.0
X : parts per Million : Proton

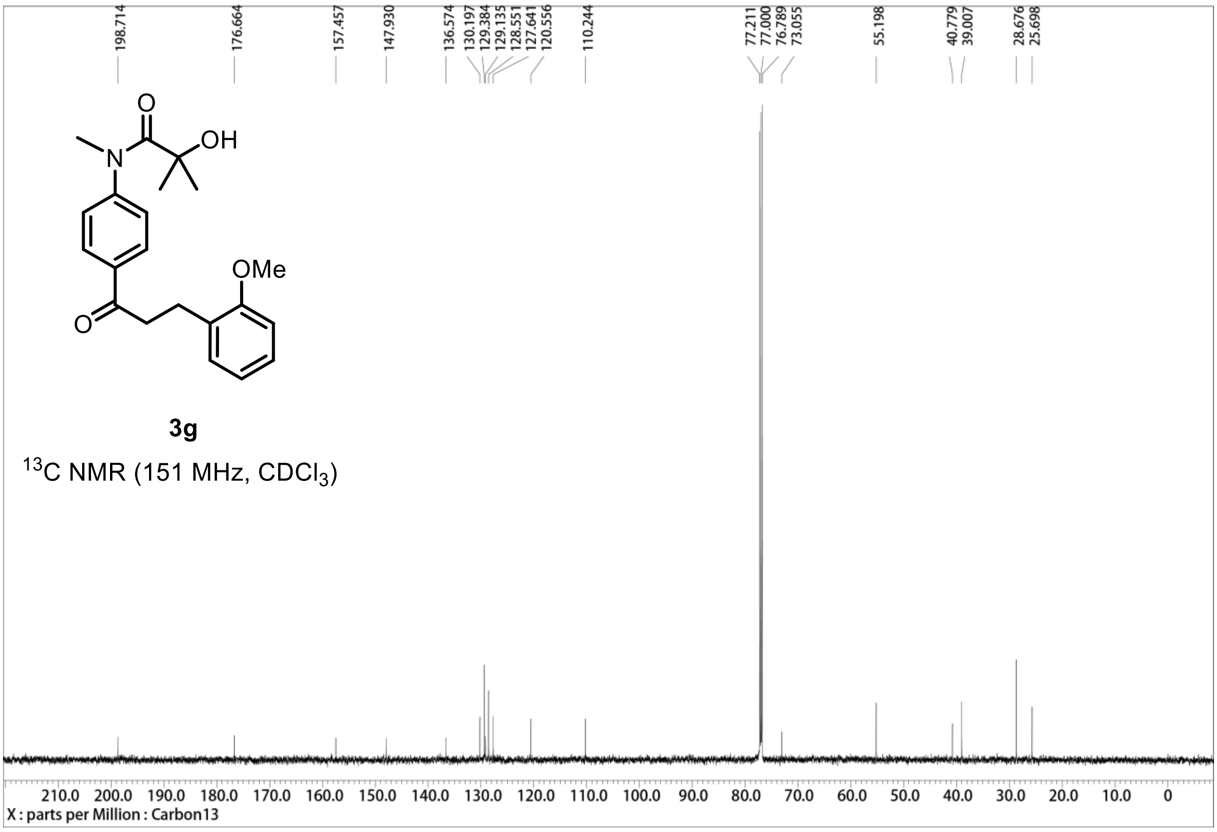
198.714
176.664
157.457
147.930
136.574
130.197
129.384
129.135
128.551
127.641
120.556
110.244
77.211
77.000
76.789
73.055
55.198
40.779
39.007
28.676
25.698
O
N
OH
OMe
O
3g
^{13}C NMR (151 MHz, CDCl$_3$)
210.0 200.0 190.0 180.0 170.0 160.0 150.0 140.0 130.0 120.0 110.0 100.0 90.0 80.0 70.0 60.0 50.0 40.0 30.0 20.0 10.0 0
X : parts per Million : Carbon13

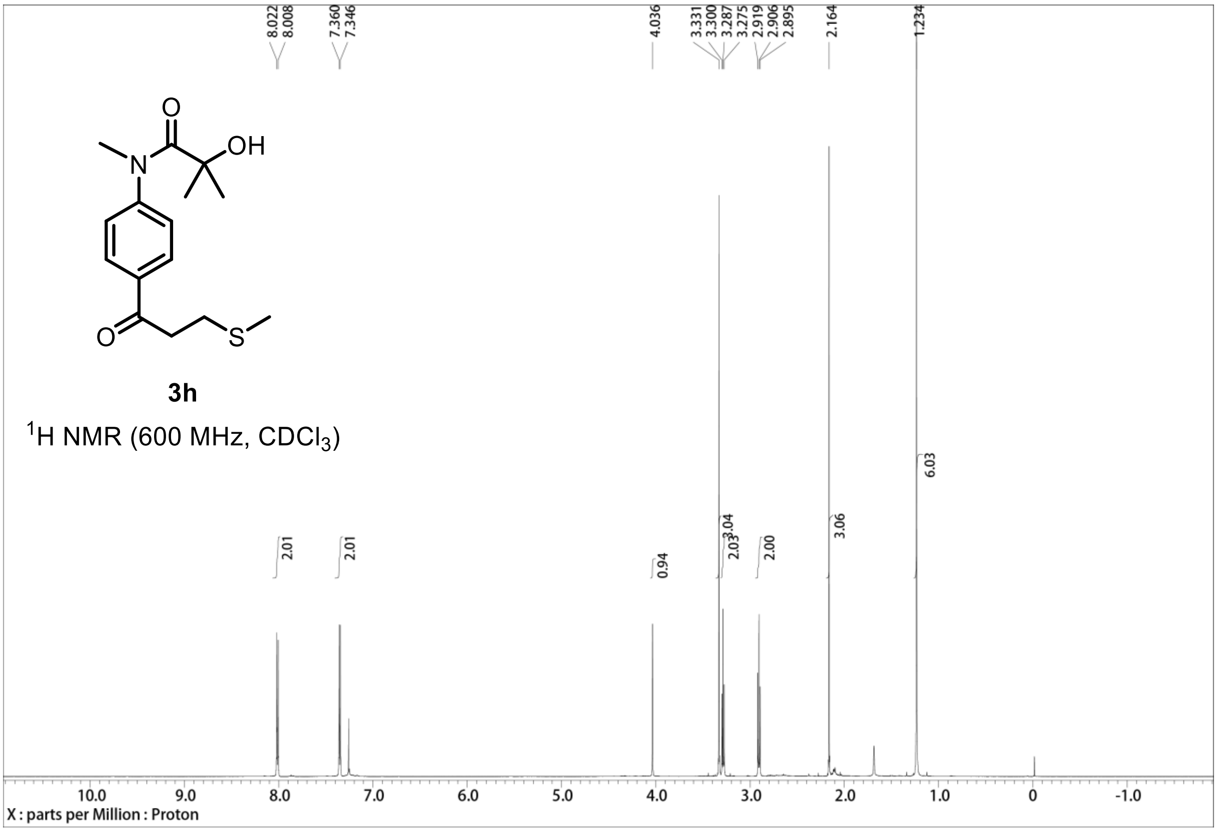
8.022
8.008
7.360
7.346
4.036
3.331
3.300
3.287
3.275
2.919
2.906
2.895
2.164
1.234
O
OH
N
O
S
3h
^{1}H NMR (600 MHz, $CDCl_3$)
2.01
2.01
0.94
3.04
2.03
2.00
3.06
6.03
10.0 9.0 8.0 7.0 6.0 5.0 4.0 3.0 2.0 1.0 0 -1.0
X : parts per Million : Proton

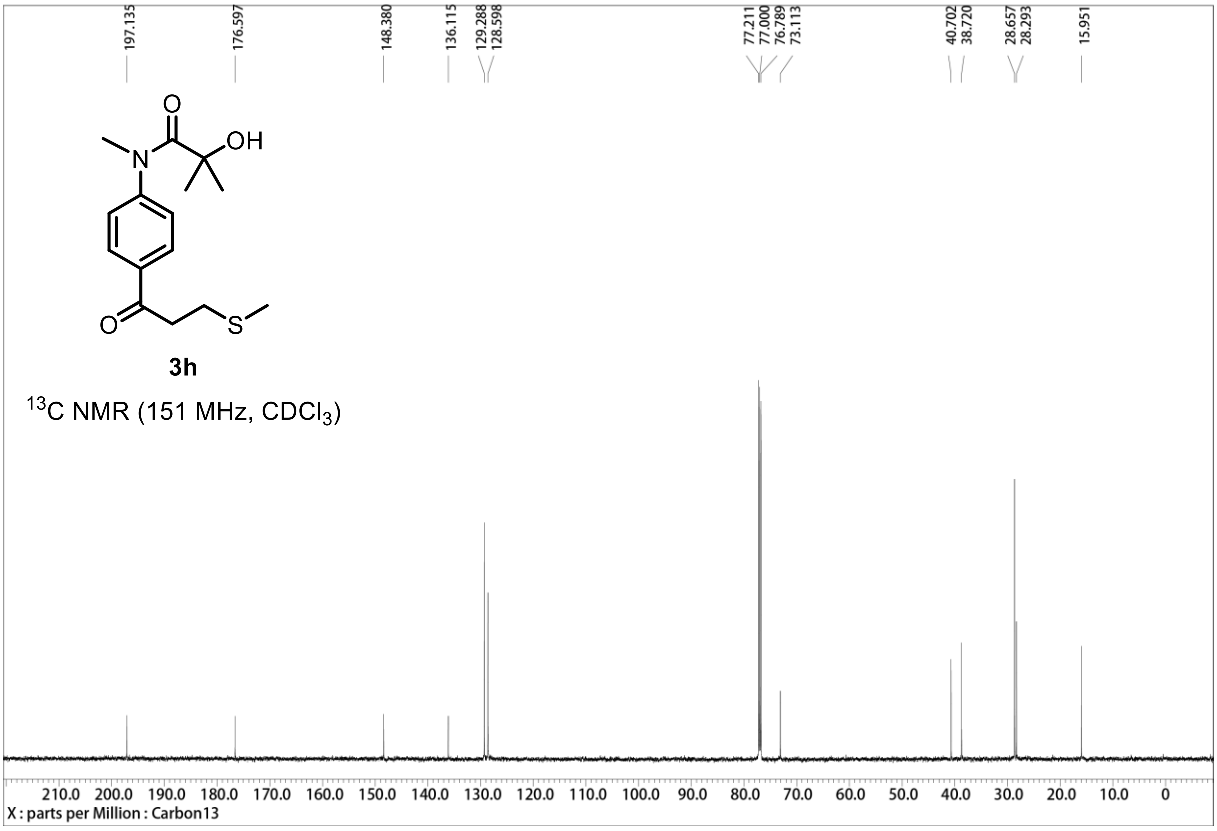
197.135
176.597
148.380
136.115
129.288
128.598
77.211
77.000
76.789
73.113
40.702
38.720
28.657
28.293
15.951
O
OH
N
O
S
3h
^{13}C NMR (151 MHz, $CDCl_3$)
210.0 200.0 190.0 180.0 170.0 160.0 150.0 140.0 130.0 120.0 110.0 100.0 90.0 80.0 70.0 60.0 50.0 40.0 30.0 20.0 10.0 0
X : parts per Million : Carbon13

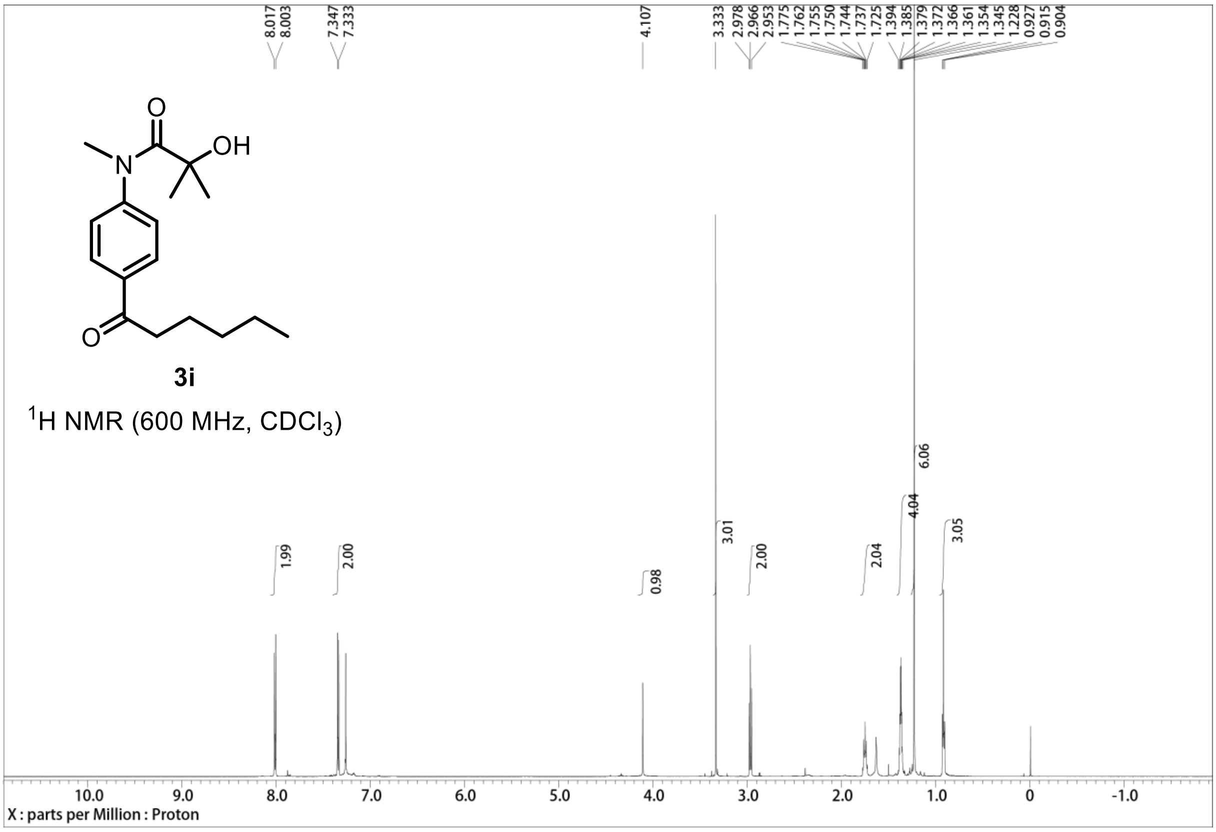
8.017
8.003
7.347
7.333
4.107
3.333
2.978
2.966
2.953
1.775
1.762
1.755
1.750
1.744
1.737
1.725
1.394
1.385
1.379
1.372
1.366
1.361
1.354
1.345
1.228
0.927
0.915
0.904
O
N
OH
O
3i
^{1}H NMR (600 MHz, CDCl$_3$)
1.99
2.00
0.98
3.01
2.00
2.04
4.04
6.06
3.05
10.0
9.0
8.0
7.0
6.0
5.0
4.0
3.0
2.0
1.0
0
-1.0
X : parts per Million : Proton

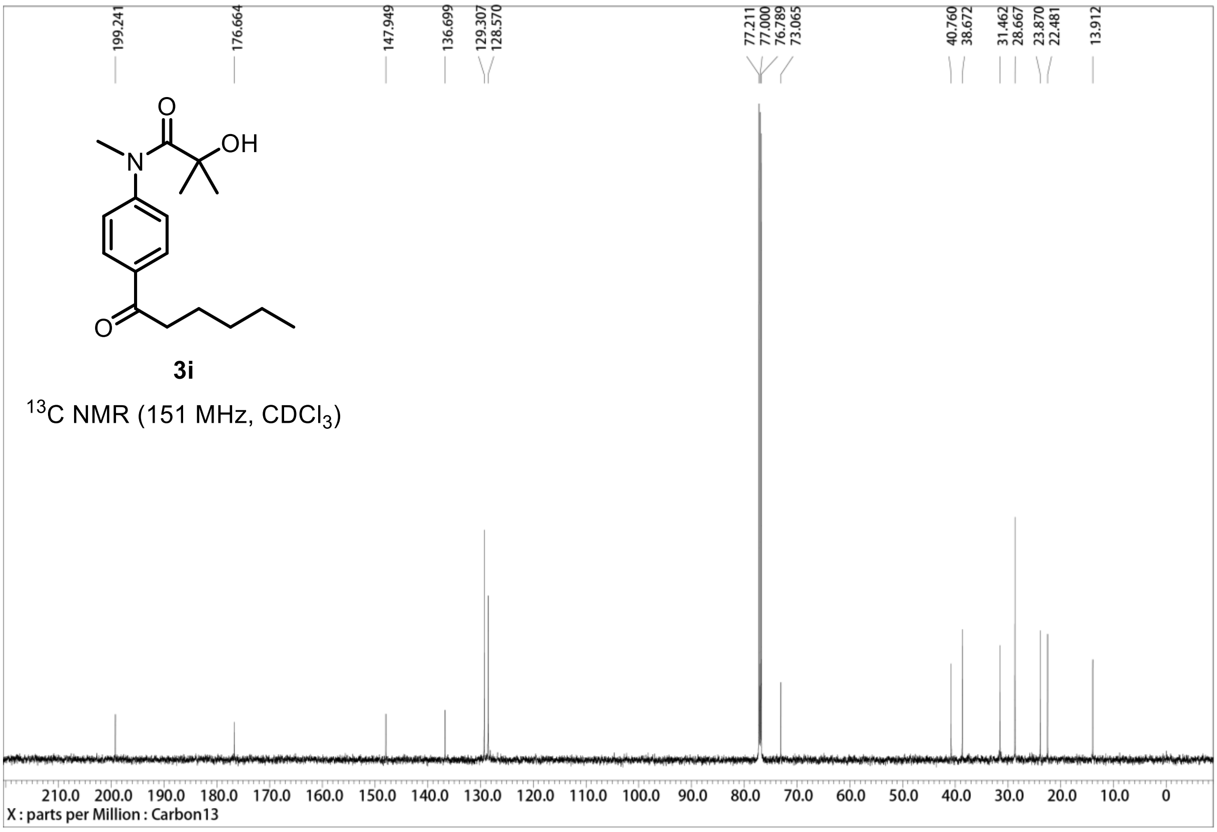
199.241
176.664
147.949
136.699
129.307
128.570
77.211
77.000
76.789
73.065
40.760
38.672
31.462
28.667
23.870
22.481
13.912
O
N
OH
O
3i
^{13}C NMR (151 MHz, CDCl$_3$)
210.0
200.0
190.0
180.0
170.0
160.0
150.0
140.0
130.0
120.0
110.0
100.0
90.0
80.0
70.0
60.0
50.0
40.0
30.0
20.0
10.0
0
X : parts per Million : Carbon13

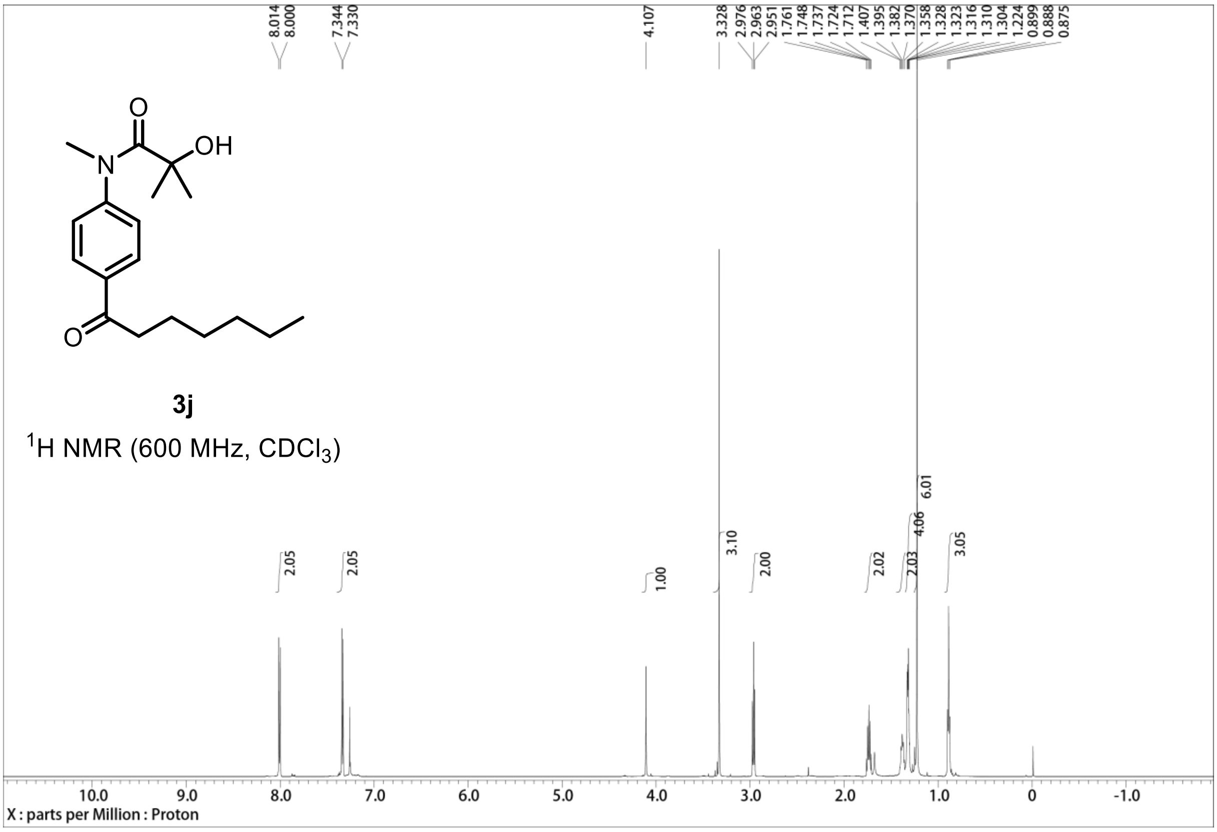
8.014
8.000
7.344
7.330
4.107
3.328
2.976
2.963
2.951
1.761
1.748
1.737
1.724
1.712
1.407
1.395
1.382
1.370
1.358
1.328
1.323
1.316
1.310
1.304
1.224
0.899
0.888
0.875
O
N
OH
O
3j
^{1}H NMR (600 MHz, $CDCl_3$)
2.05
2.05
1.00
3.10
2.00
2.02
2.03
4.06
6.01
3.05
10.0
9.0
8.0
7.0
6.0
5.0
4.0
3.0
2.0
1.0
0
-1.0
X : parts per Million : Proton

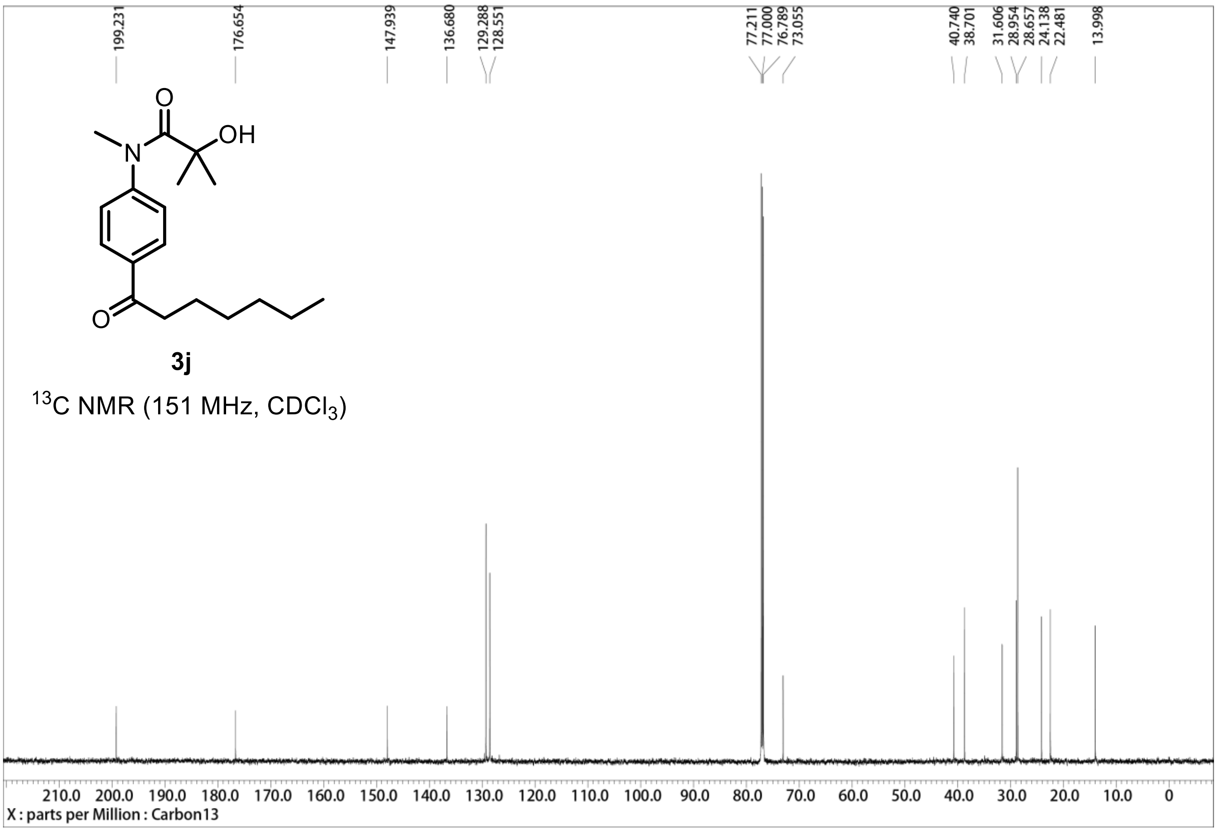
199.231
176.654
147.939
136.680
129.288
128.551
77.211
77.000
76.789
73.055
40.740
38.701
31.606
28.954
28.657
24.138
22.481
13.998
O
N
OH
O
3j
^{13}C NMR (151 MHz, $CDCl_3$)
210.0
200.0
190.0
180.0
170.0
160.0
150.0
140.0
130.0
120.0
110.0
100.0
90.0
80.0
70.0
60.0
50.0
40.0
30.0
20.0
10.0
0
X : parts per Million : Carbon13

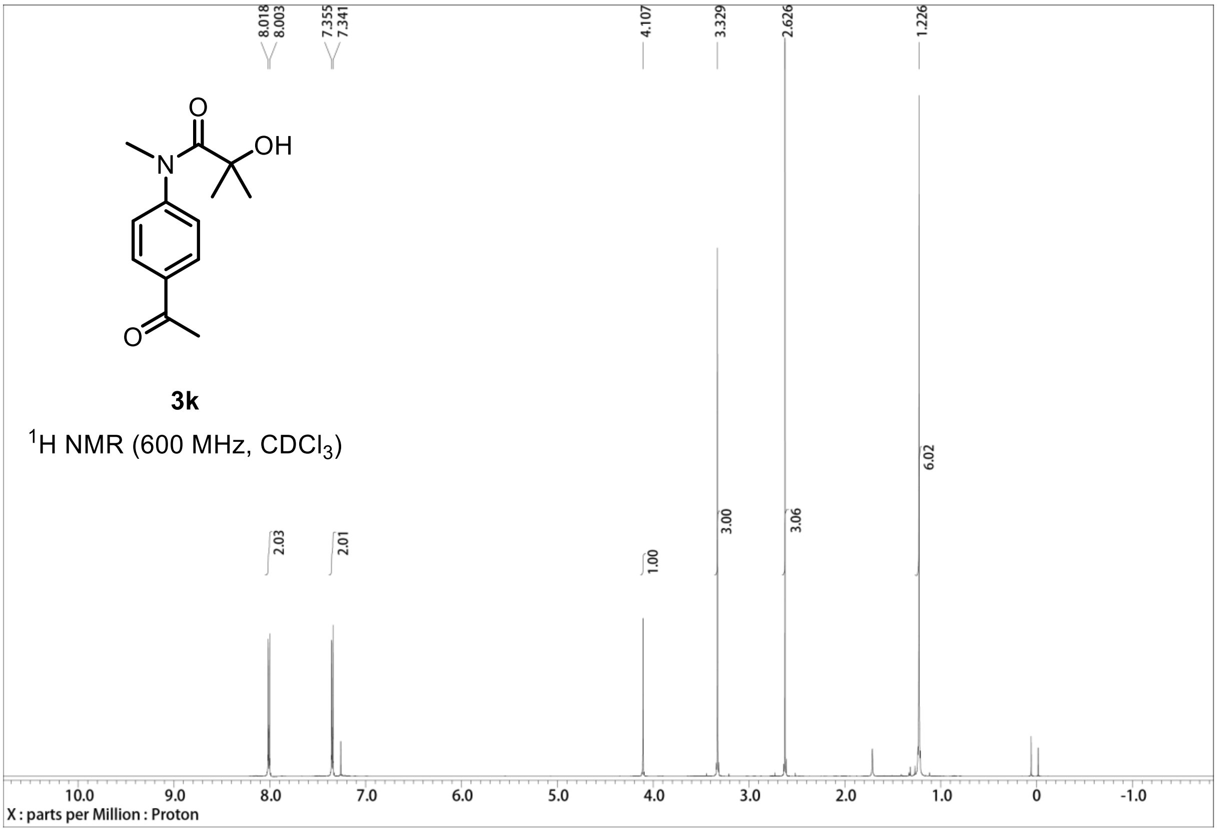
8.018
8.003
7.355
7.341
4.107
3.329
2.626
1.226
O
N
OH
O
3k
1H NMR (600 MHz, CDCl3)
2.03
2.01
1.00
3.00
3.06
6.02
10.0 9.0 8.0 7.0 6.0 5.0 4.0 3.0 2.0 1.0 0 -1.0
X : parts per Million : Proton

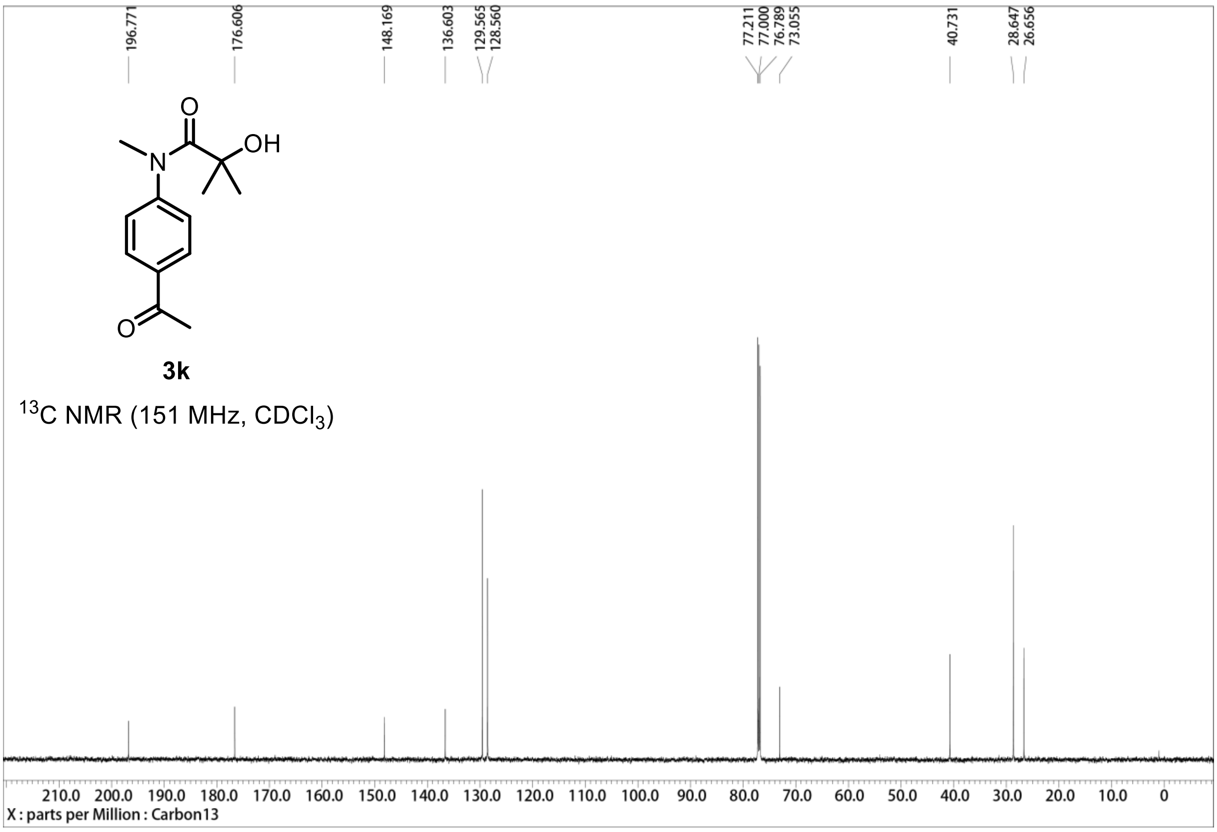
196.771
176.606
148.169
136.603
129.565
128.560
77.211
77.000
76.789
73.055
40.731
28.647
26.656
O
N
OH
O
3k
13C NMR (151 MHz, CDCl3)
210.0 200.0 190.0 180.0 170.0 160.0 150.0 140.0 130.0 120.0 110.0 100.0 90.0 80.0 70.0 60.0 50.0 40.0 30.0 20.0 10.0 0
X : parts per Million : Carbon13

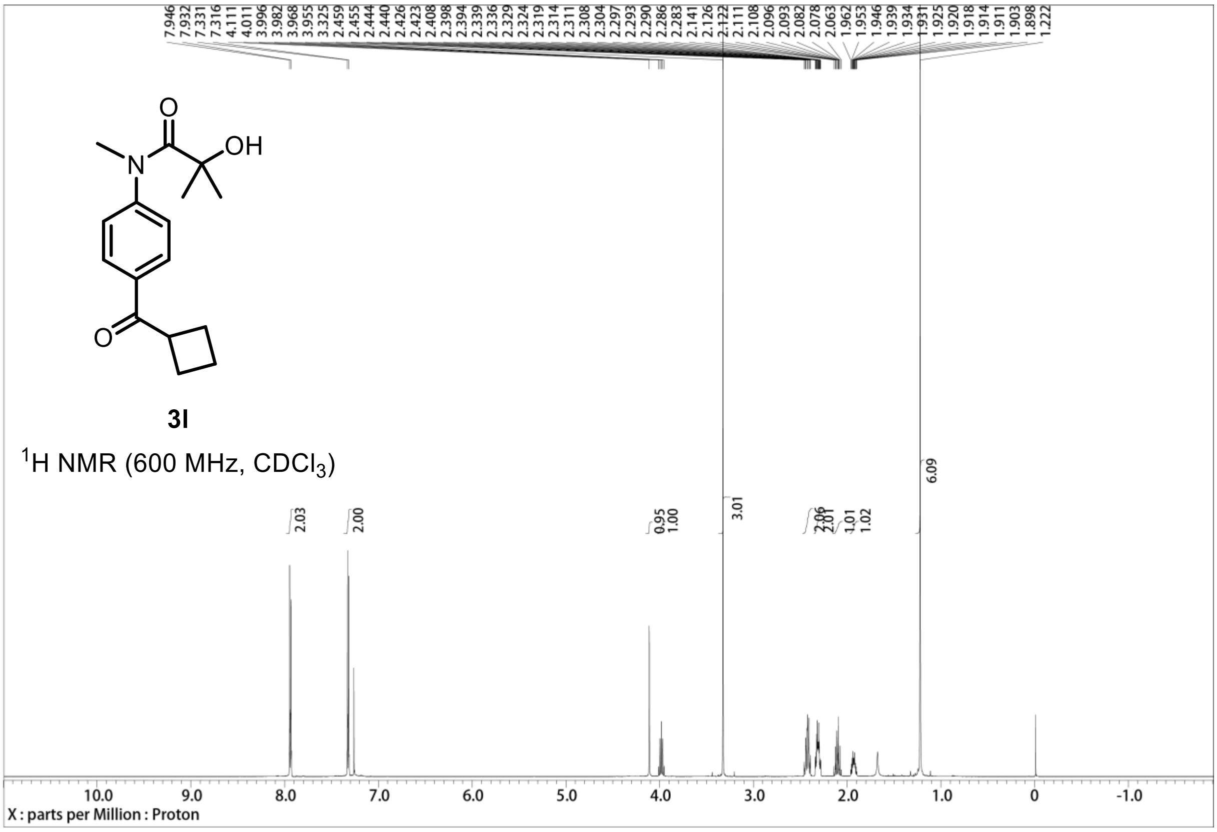
7.946 7.932 7.331 7.316 4.111 4.011 3.996 3.982 3.968 3.955 3.325 2.459 2.455 2.444 2.440 2.426 2.423 2.408 2.398 2.394 2.339 2.336 2.329 2.324 2.319 2.314 2.311 2.308 2.304 2.297 2.293 2.290 2.286 2.283 2.141 2.126 2.122 2.111 2.108 2.096 2.093 2.082 2.078 2.063 1.962 1.953 1.946 1.939 1.934 1.931 1.925 1.920 1.918 1.914 1.911 1.903 1.898 1.222
O
N
OH
O
3l
^{1}H NMR (600 MHz, $CDCl_3$)
2.03 2.00 0.95 1.00 3.01 2.06 2.01 1.01 1.02 6.09
10.0 9.0 8.0 7.0 6.0 5.0 4.0 3.0 2.0 1.0 0 -1.0
X : parts per Million : Proton

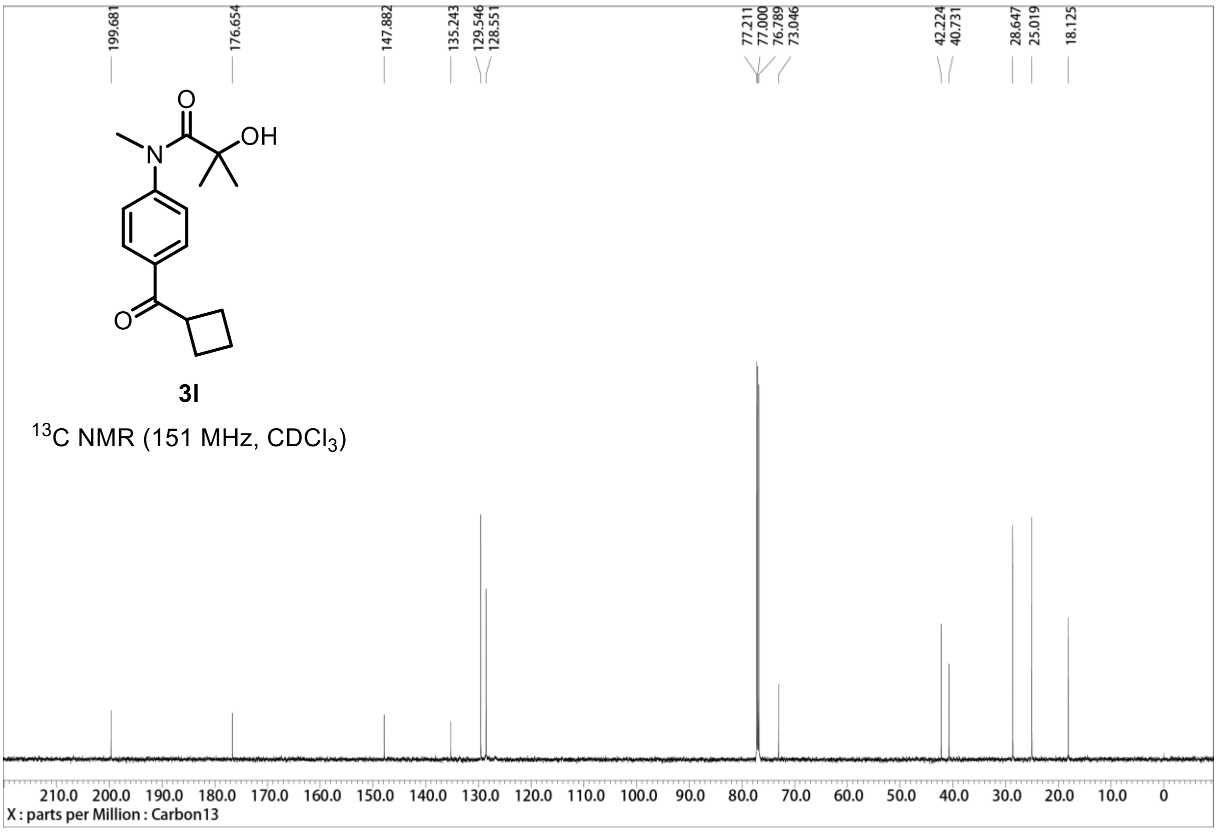
199.681 176.654 147.882 135.243 129.546 128.551 77.211 77.000 76.789 73.046 42.224 40.731 28.647 25.019 18.125
O
N
OH
O
3l
^{13}C NMR (151 MHz, $CDCl_3$)
210.0 200.0 190.0 180.0 170.0 160.0 150.0 140.0 130.0 120.0 110.0 100.0 90.0 80.0 70.0 60.0 50.0 40.0 30.0 20.0 10.0 0
X : parts per Million : Carbon13

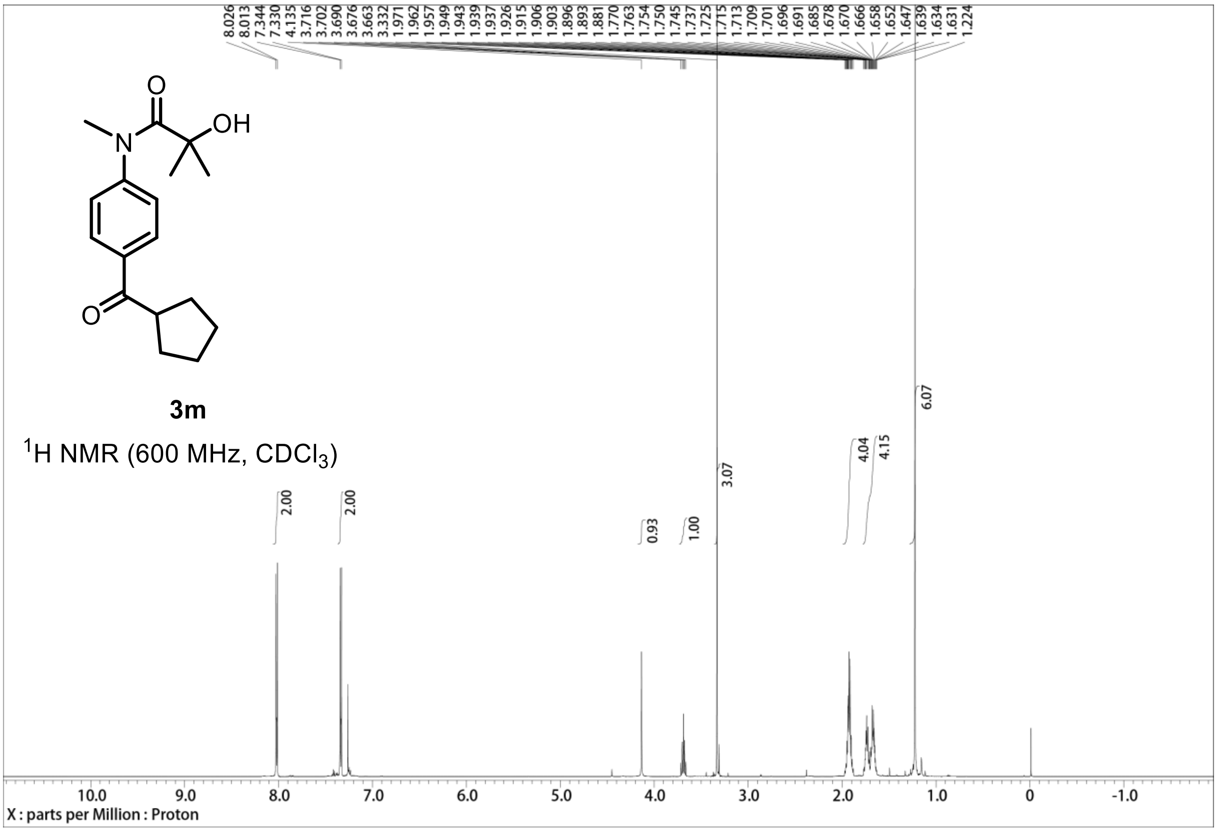

8.026
8.013
7.344
7.330
4.135
3.716
3.702
3.690
3.676
3.663
3.332
1.971
1.962
1.957
1.949
1.943
1.939
1.937
1.926
1.915
1.906
1.903
1.896
1.893
1.881
1.770
1.763
1.754
1.750
1.745
1.737
1.725
1.715
1.713
1.709
1.701
1.696
1.691
1.685
1.678
1.670
1.666
1.658
1.652
1.647
1.639
1.634
1.631
1.224
O
N
OH
O
3m
1H NMR (600 MHz, CDCl3)
2.00
2.00
0.93
1.00
3.07
4.04
4.15
6.07
X : parts per Million : Proton

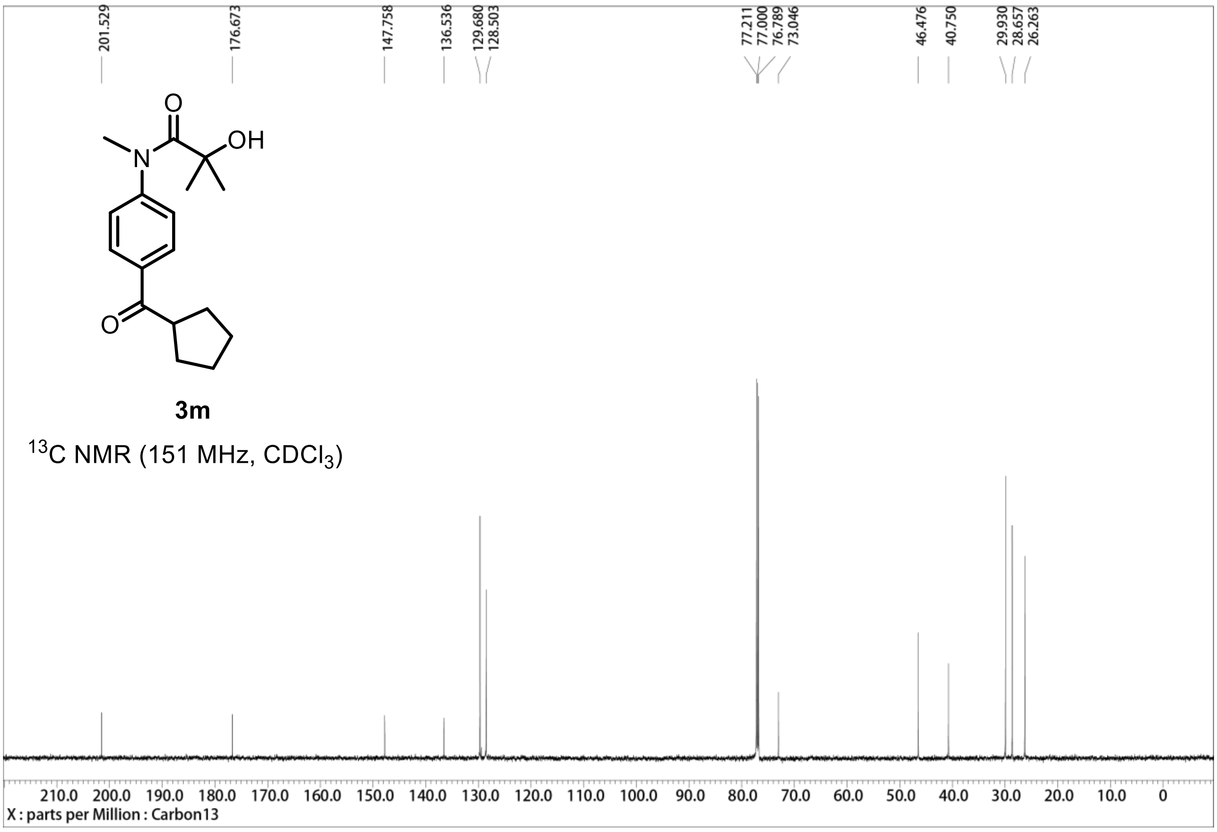

201.529
176.673
147.758
136.536
129.680
128.503
77.211
77.000
76.789
73.046
46.476
40.750
29.930
28.657
26.263
O
N
OH
O
3m
13C NMR (151 MHz, CDCl3)
X : parts per Million : Carbon13

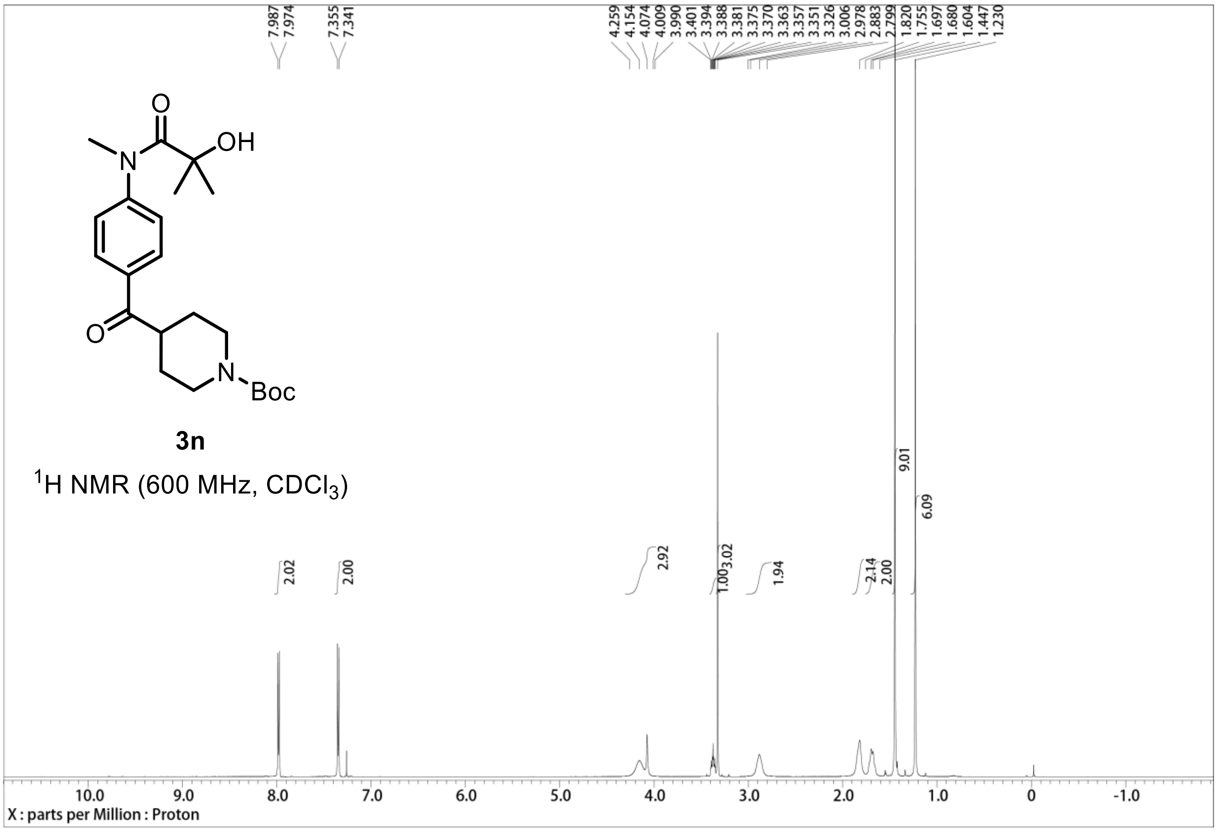
3n
^{1}H NMR (600 MHz, CDCl$_3$)
X : parts per Million : Proton

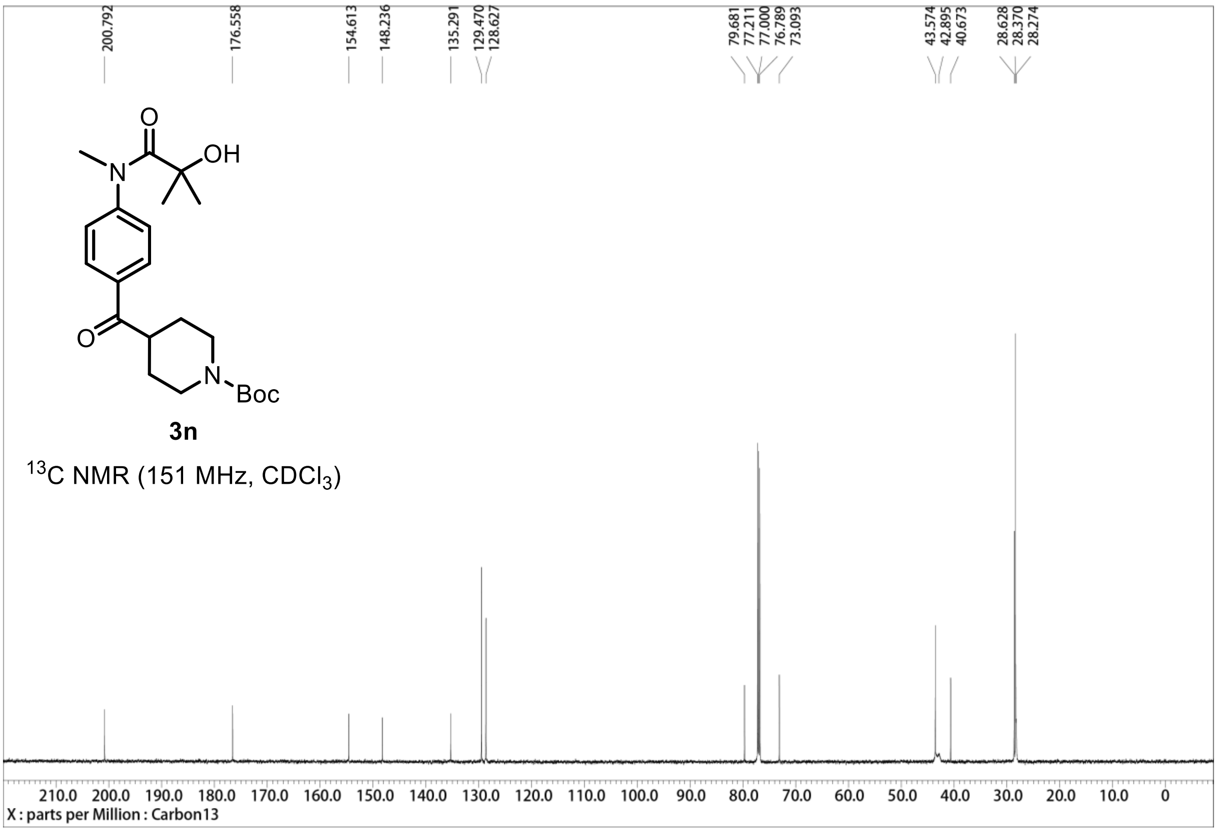
3n
^{13}C NMR (151 MHz, CDCl$_3$)
X : parts per Million : Carbon13

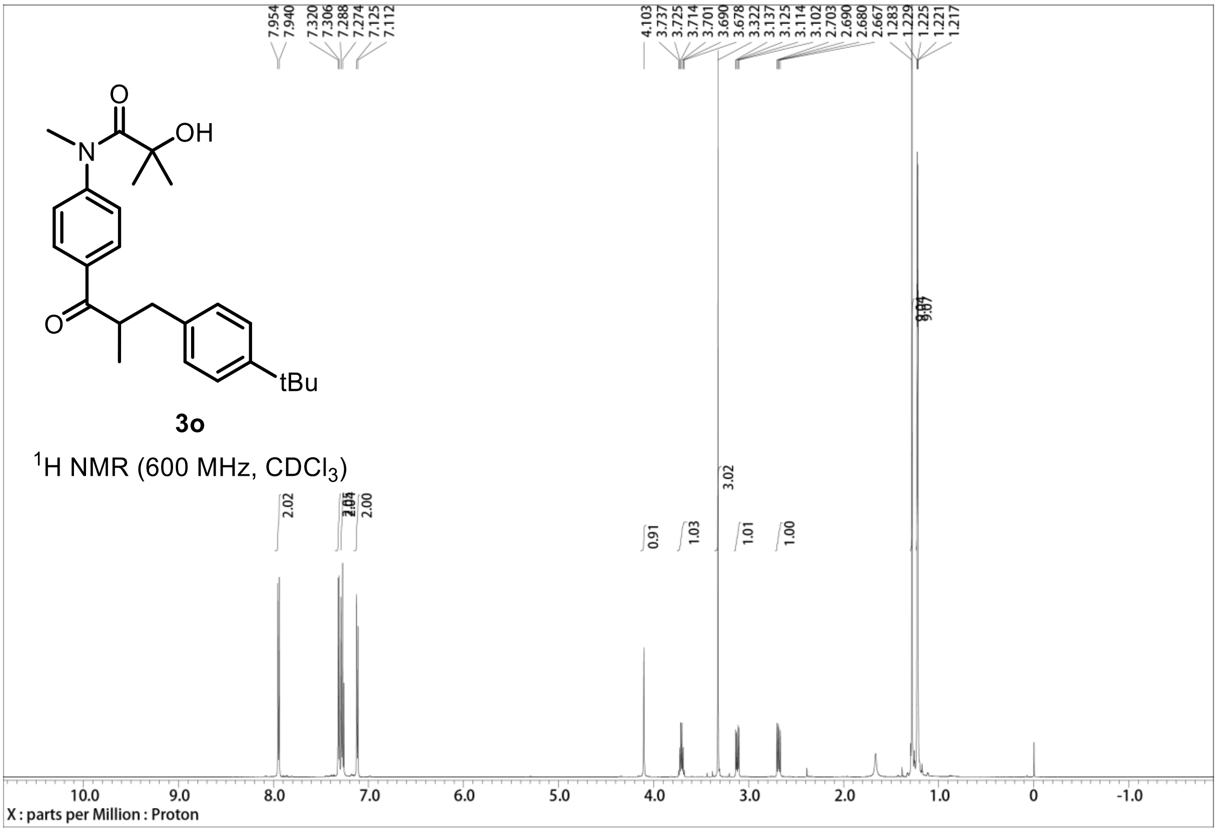
7.954
7.940
7.320
7.306
7.288
7.274
7.125
7.112
4.103
3.737
3.725
3.714
3.701
3.690
3.678
3.322
3.137
3.125
3.114
3.102
2.703
2.690
2.680
2.667
1.283
1.229
1.225
1.221
1.217
O
N
OH
O
tBu
3o
^{1}H NMR (600 MHz, $CDCl_3$)
2.02
2.00
0.91
1.03
3.02
1.01
1.00
10.0
9.0
8.0
7.0
6.0
5.0
4.0
3.0
2.0
1.0
0
-1.0
X : parts per Million : Proton

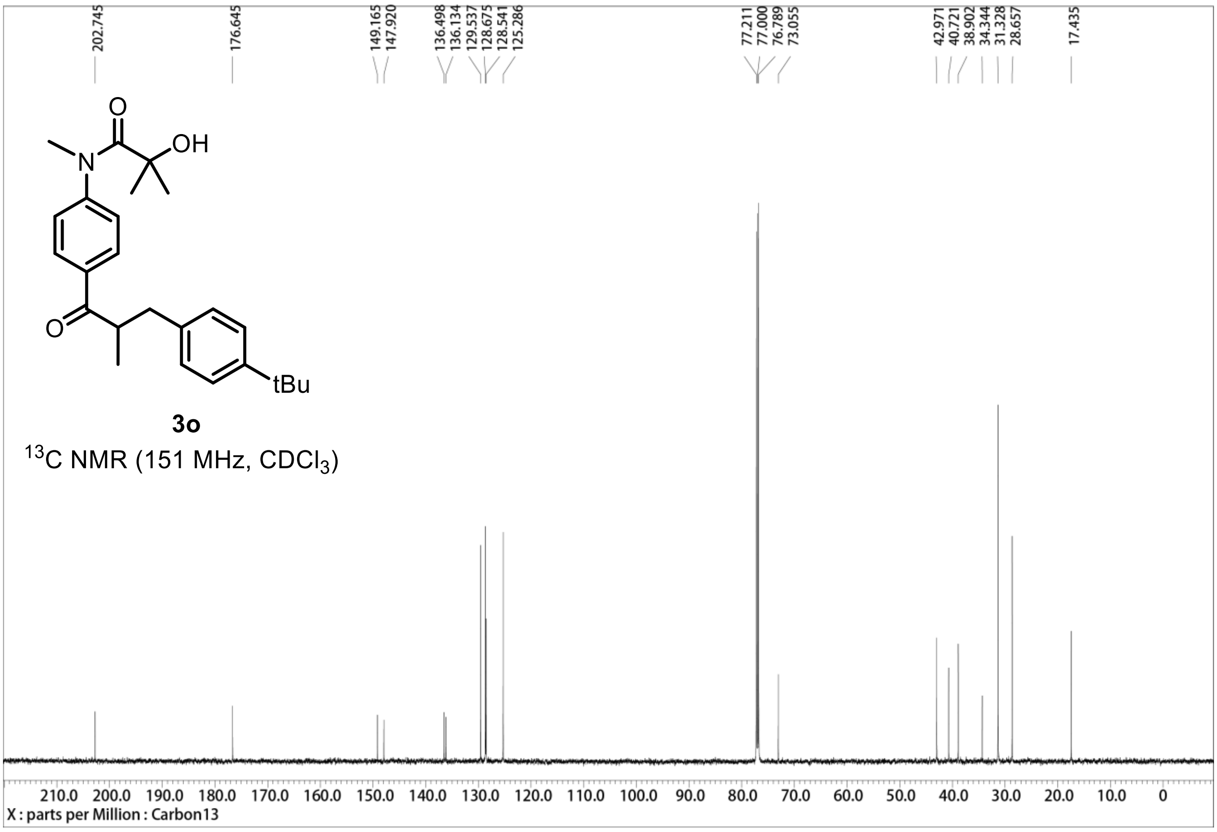
202.745
176.645
149.165
147.920
136.498
136.134
129.537
128.675
128.541
125.286
77.211
77.000
76.789
73.055
42.971
40.721
38.902
34.344
31.328
28.657
17.435
O
N
OH
O
tBu
3o
^{13}C NMR (151 MHz, $CDCl_3$)
210.0
200.0
190.0
180.0
170.0
160.0
150.0
140.0
130.0
120.0
110.0
100.0
90.0
80.0
70.0
60.0
50.0
40.0
30.0
20.0
10.0
0
X : parts per Million : Carbon13

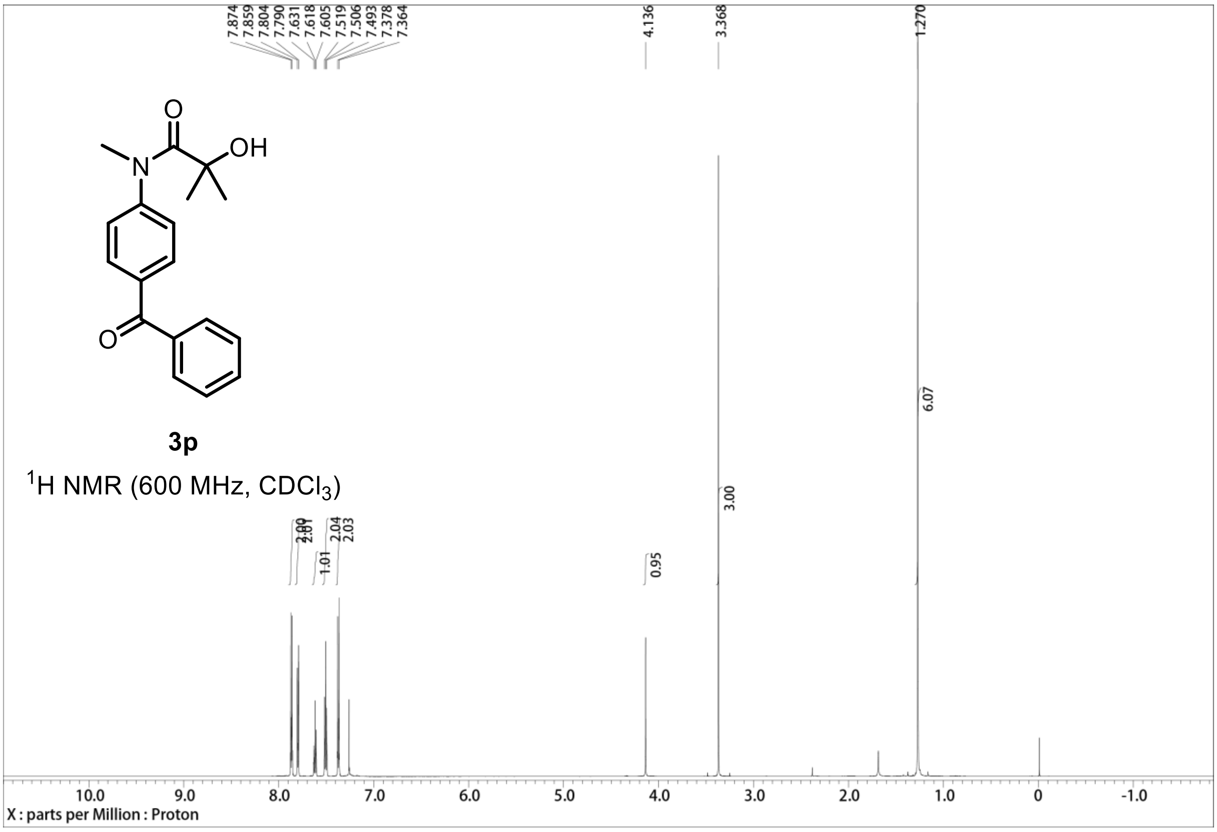
7.874
7.859
7.804
7.790
7.631
7.618
7.605
7.519
7.506
7.493
7.378
7.364
4.136
3.368
1.270
3p
^{1}H NMR (600 MHz, $CDCl_3$)
6.07
3.00
0.95
1.01
2.04
2.03
10.0 9.0 8.0 7.0 6.0 5.0 4.0 3.0 2.0 1.0 0 -1.0
X : parts per Million : Proton

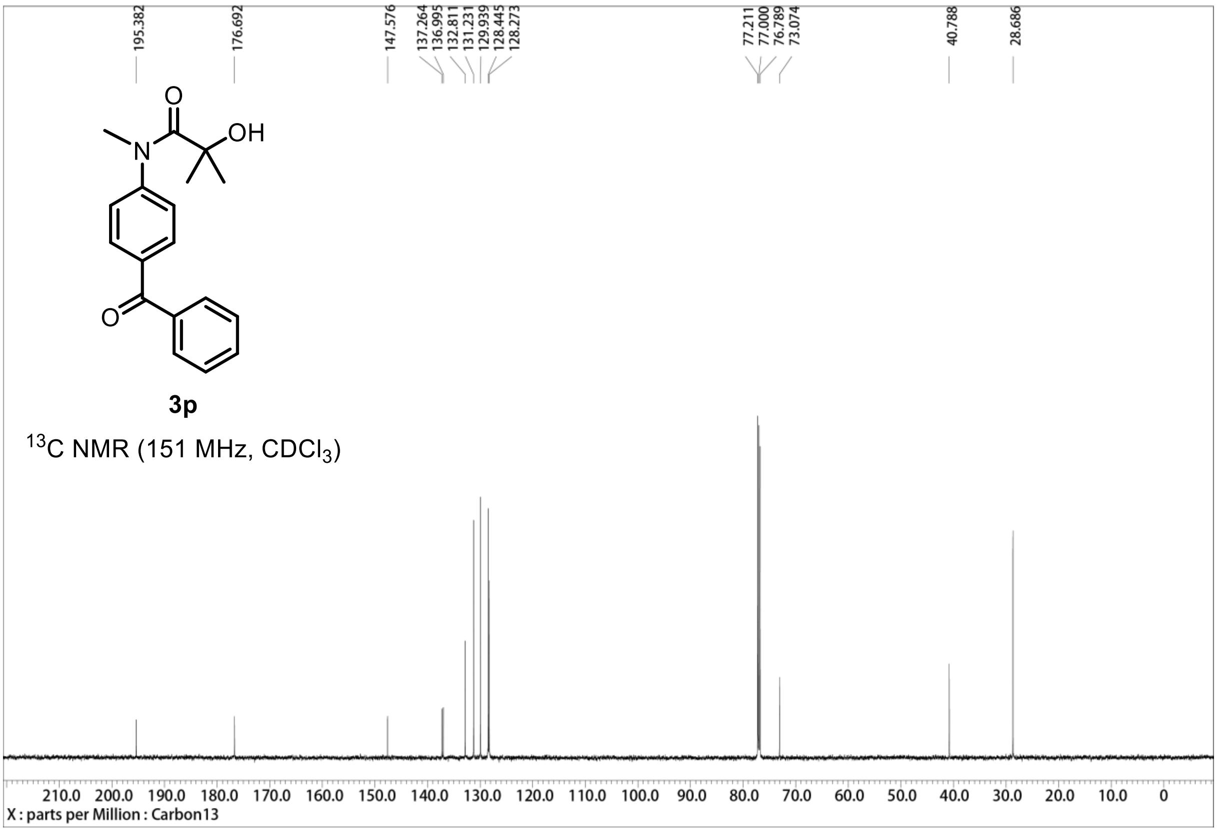
195.382
176.692
147.576
137.264
136.995
132.811
131.231
129.939
128.445
128.273
77.211
77.000
76.789
73.074
40.788
28.686
3p
^{13}C NMR (151 MHz, $CDCl_3$)
210.0 200.0 190.0 180.0 170.0 160.0 150.0 140.0 130.0 120.0 110.0 100.0 90.0 80.0 70.0 60.0 50.0 40.0 30.0 20.0 10.0 0
X : parts per Million : Carbon13

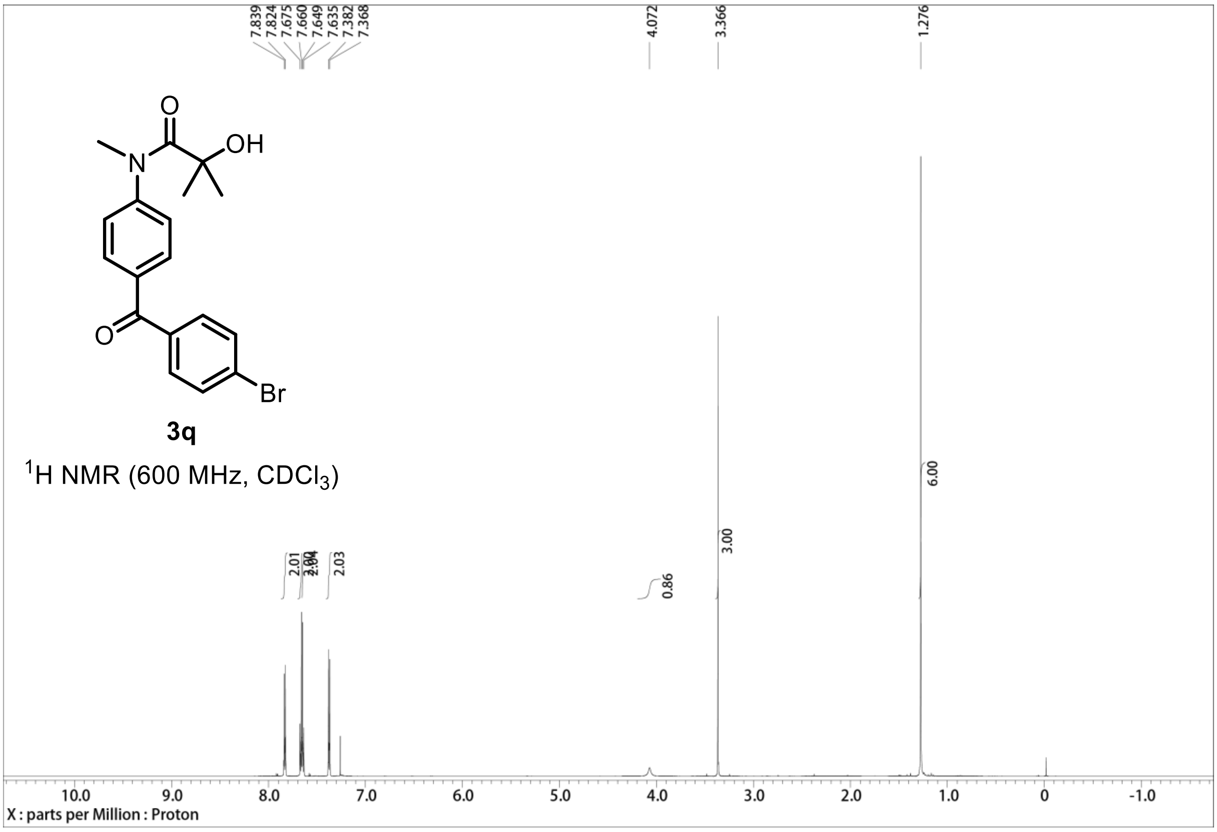
7.839
7.824
7.675
7.660
7.649
7.635
7.382
7.368
4.072
3.366
1.276
OH
O
N
O
Br
3q
1H NMR (600 MHz, CDCl3)
6.00
3.00
0.86
2.01
2.03
10.0 9.0 8.0 7.0 6.0 5.0 4.0 3.0 2.0 1.0 0 -1.0
X : parts per Million : Proton

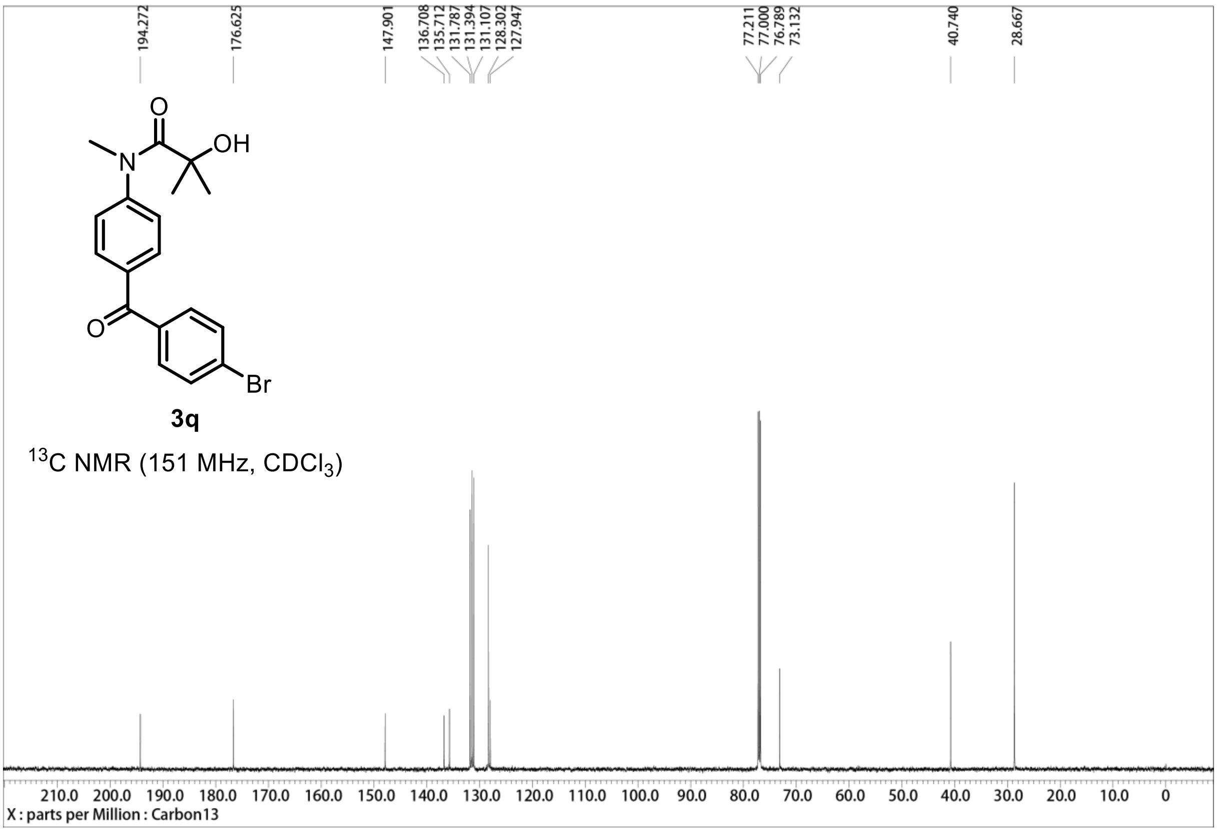
194.272
176.625
147.901
136.708
135.712
131.787
131.394
131.107
128.302
127.947
77.211
77.000
76.789
73.132
40.740
28.667
O
N
OH
O
Br
3q
13C NMR (151 MHz, CDCl3)
210.0 200.0 190.0 180.0 170.0 160.0 150.0 140.0 130.0 120.0 110.0 100.0 90.0 80.0 70.0 60.0 50.0 40.0 30.0 20.0 10.0 0
X : parts per Million : Carbon13

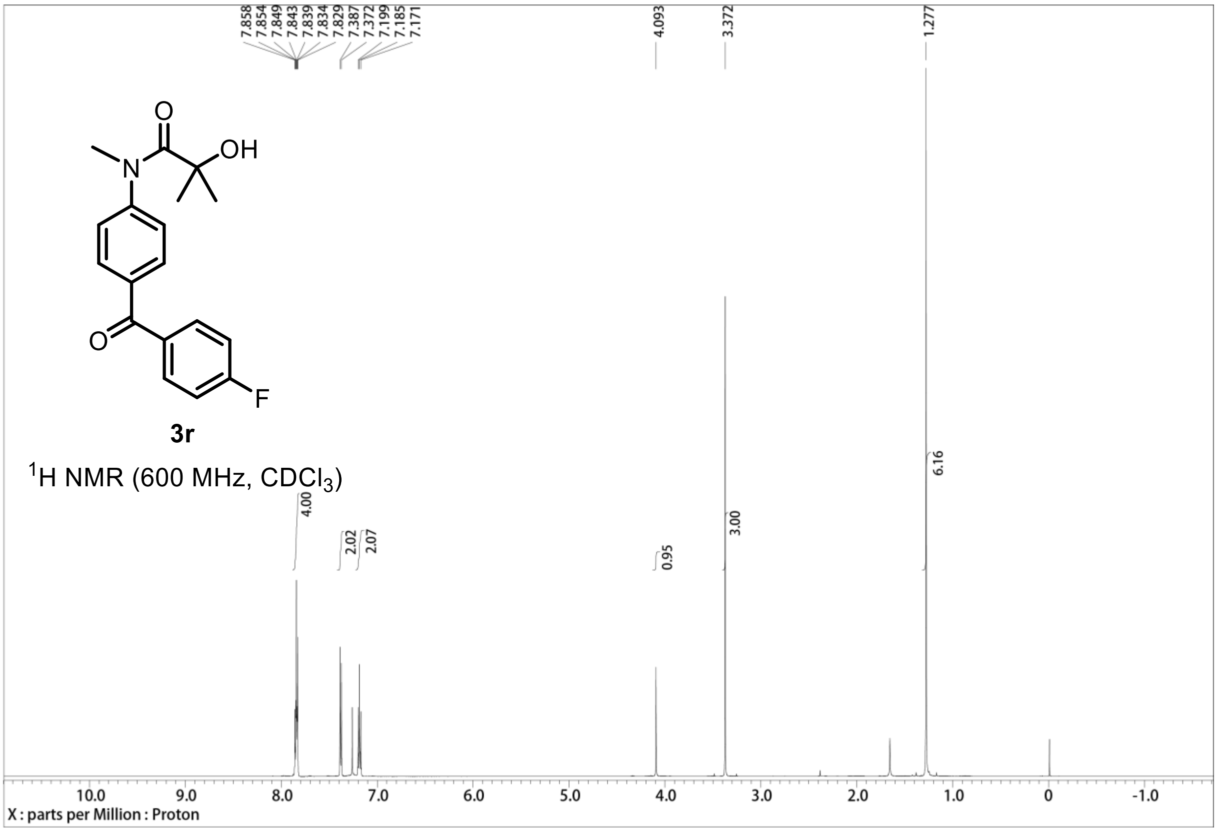
7.858
7.854
7.849
7.843
7.839
7.834
7.829
7.387
7.372
7.199
7.185
7.171
4.093
3.372
1.277
O
N
OH
O
F
3r
1H NMR (600 MHz, CDCl3)
4.00
2.02
2.07
0.95
3.00
6.16
10.0 9.0 8.0 7.0 6.0 5.0 4.0 3.0 2.0 1.0 0 -1.0
X : parts per Million : Proton

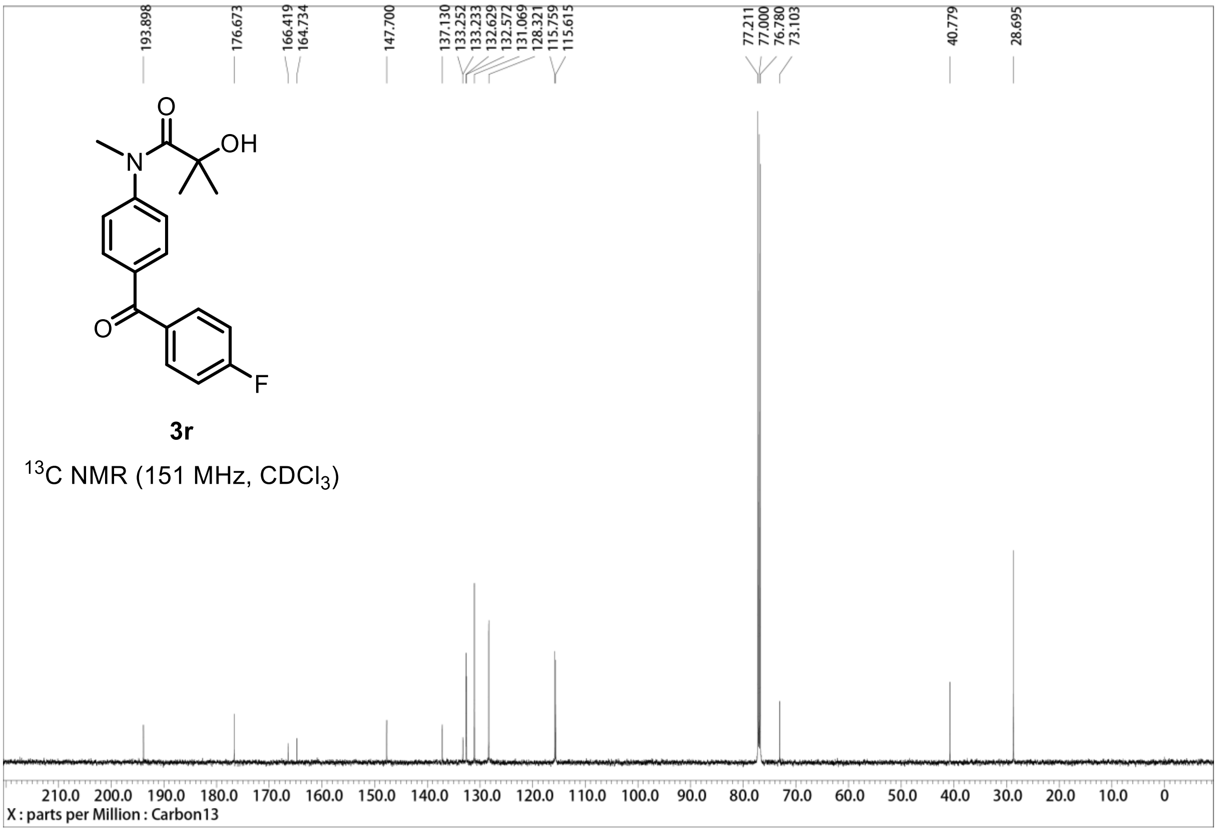
193.898
176.673
166.419
164.734
147.700
137.130
133.252
133.233
132.629
132.572
131.069
128.321
115.759
115.615
77.211
77.000
76.780
73.103
40.779
28.695
O
N
OH
O
F
3r
13C NMR (151 MHz, CDCl3)
210.0 200.0 190.0 180.0 170.0 160.0 150.0 140.0 130.0 120.0 110.0 100.0 90.0 80.0 70.0 60.0 50.0 40.0 30.0 20.0 10.0 0
X : parts per Million : Carbon13

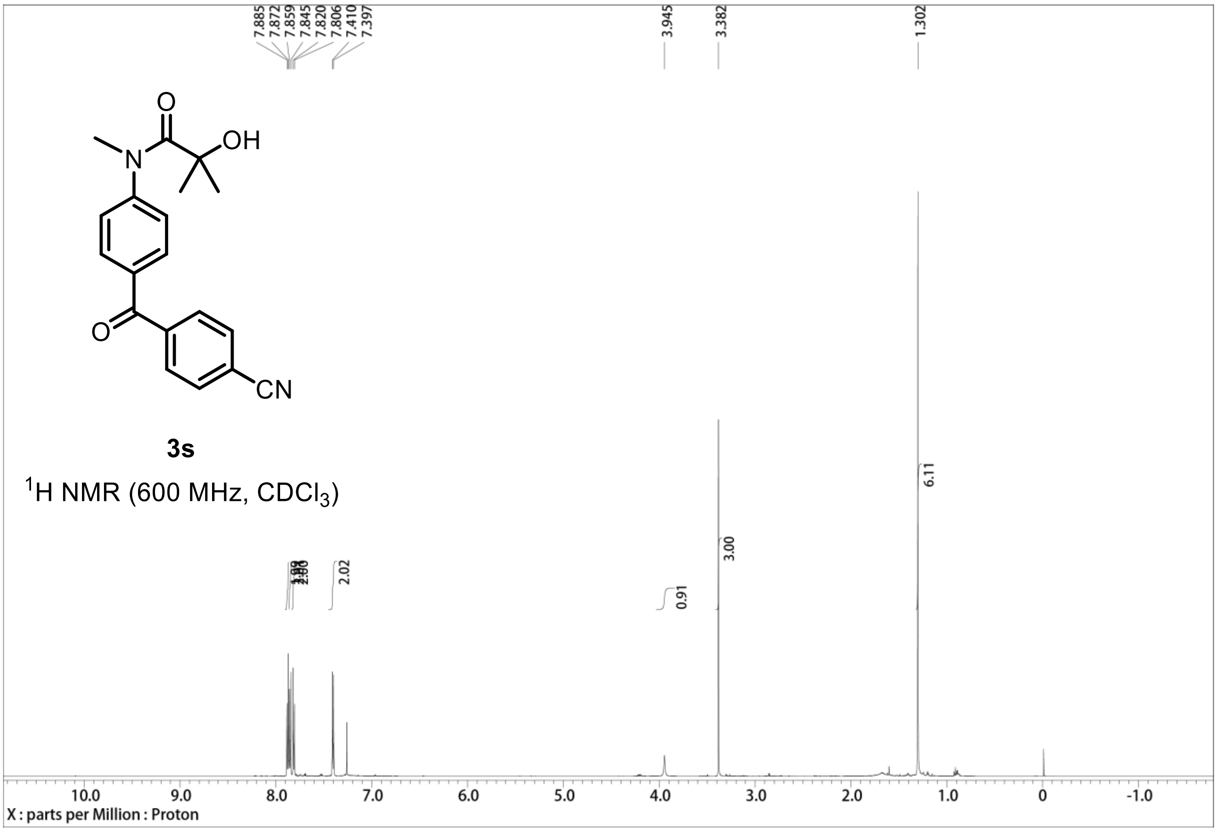
7.885
7.872
7.859
7.845
7.820
7.806
7.410
7.397
3.945
3.382
1.302
3s
1H NMR (600 MHz, CDCl3)
2.02
0.91
3.00
6.11
X : parts per Million : Proton

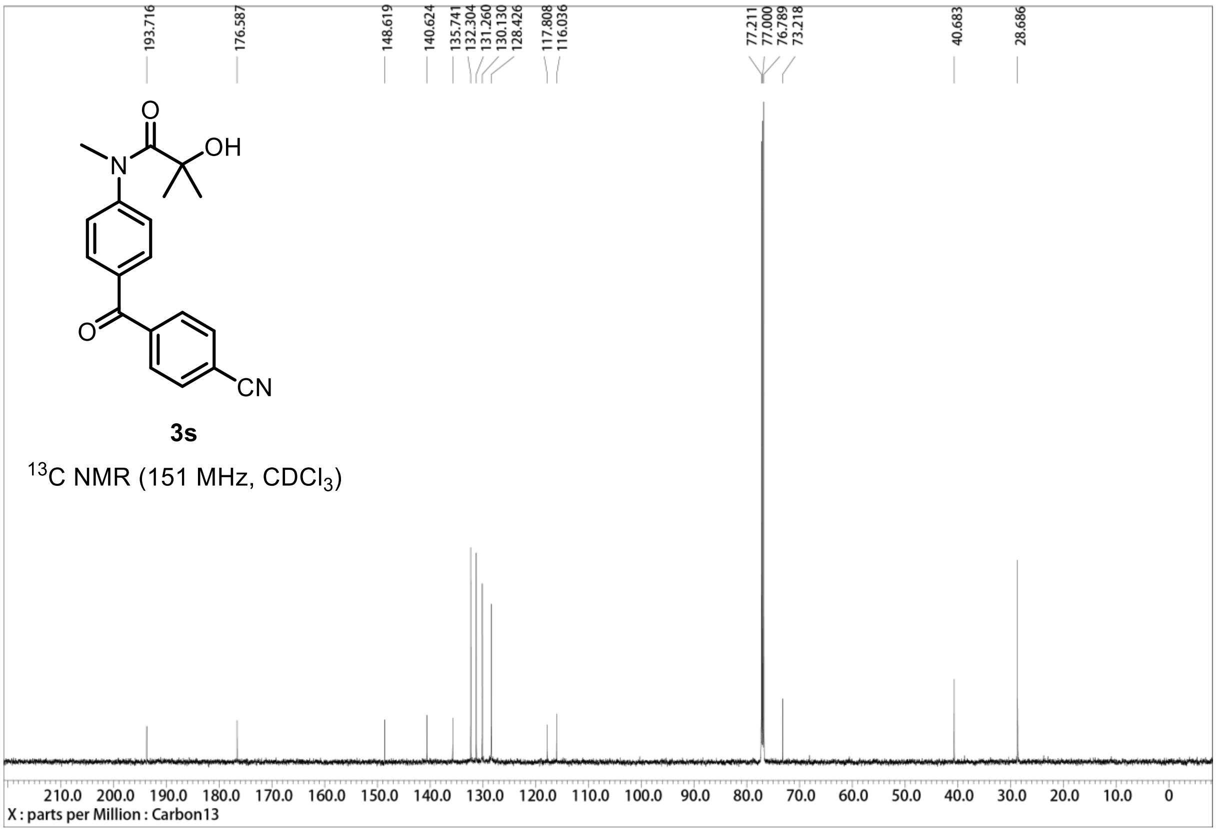
193.716
176.587
148.619
140.624
135.741
132.304
131.260
130.130
128.426
117.808
116.036
77.211
77.000
76.789
73.218
40.683
28.686
3s
13C NMR (151 MHz, CDCl3)
X : parts per Million : Carbon13

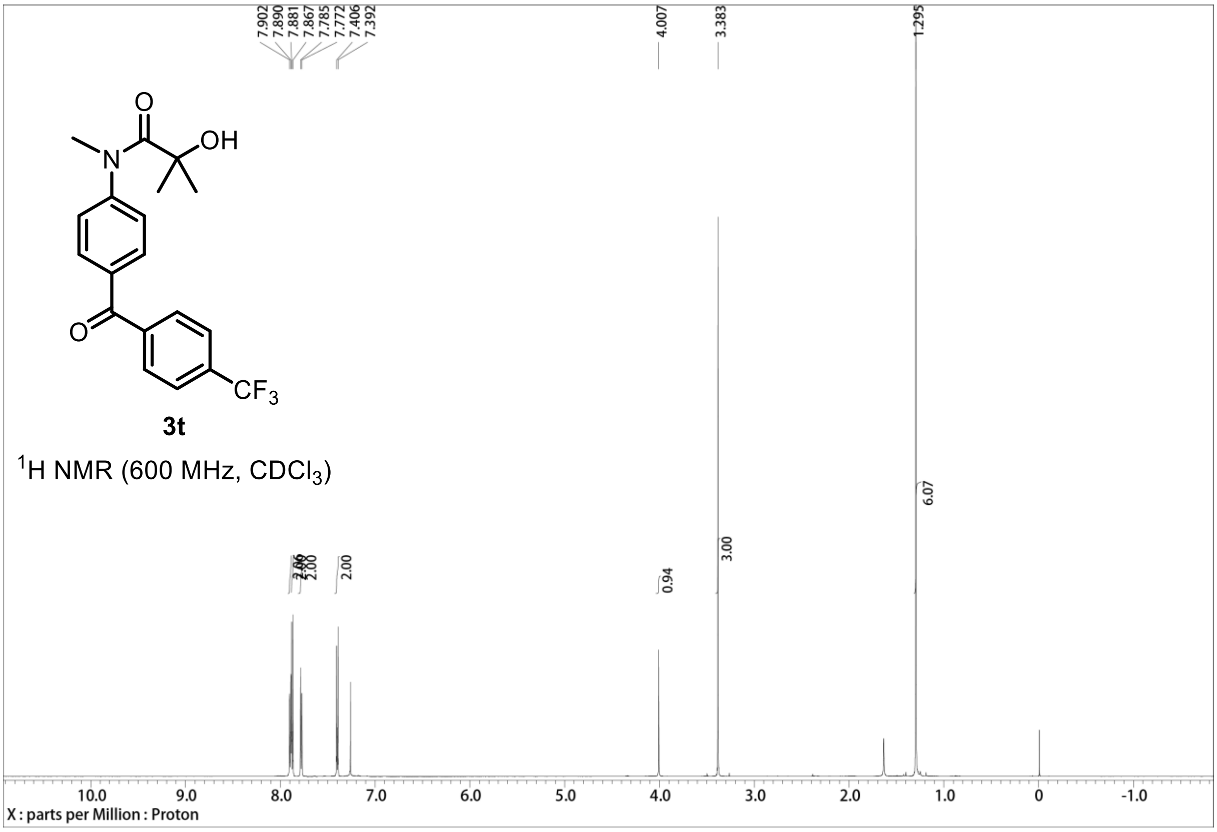
7.902
7.890
7.881
7.867
7.785
7.772
7.406
7.392
4.007
3.383
1.295
O
N
OH
O
CF3
3t
1H NMR (600 MHz, CDCl3)
2.00
2.00
0.94
3.00
6.07
10.0 9.0 8.0 7.0 6.0 5.0 4.0 3.0 2.0 1.0 0 -1.0
X : parts per Million : Proton

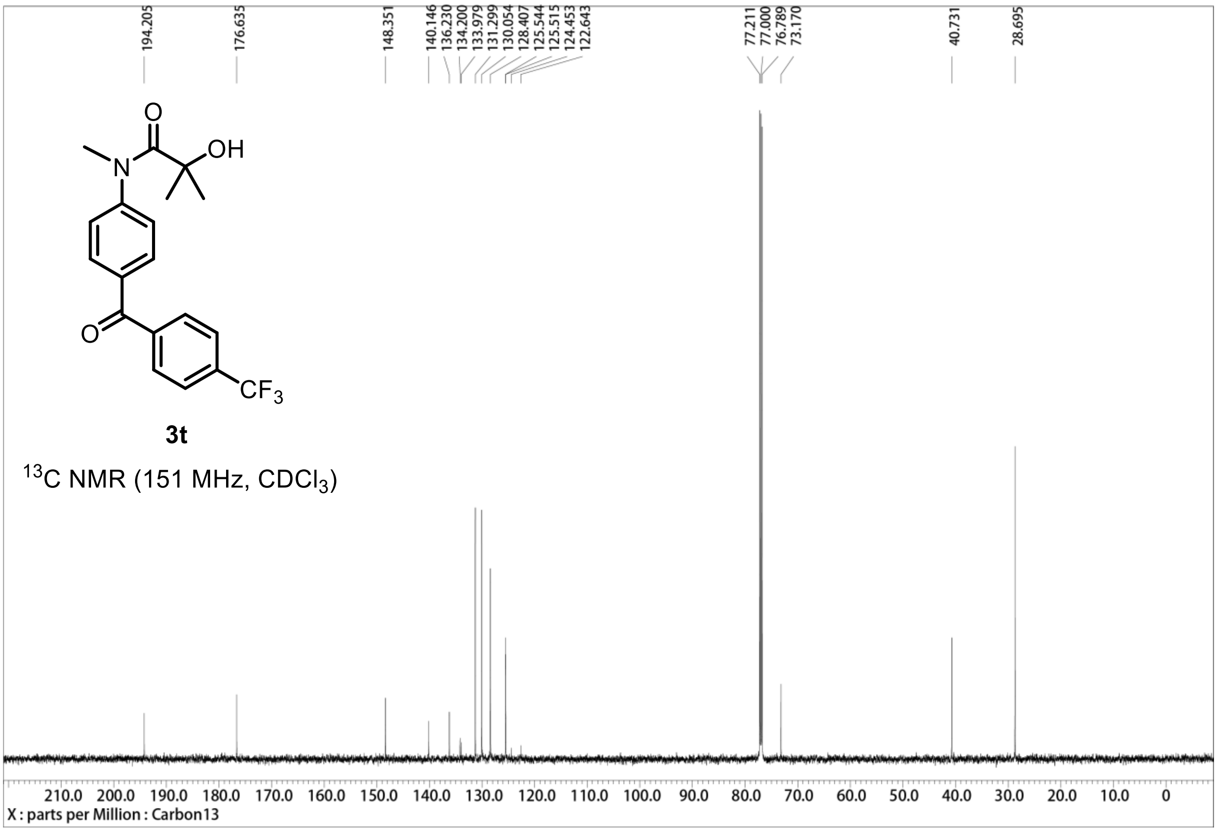
194.205
176.635
148.351
140.146
136.230
134.200
133.979
131.299
130.054
128.407
125.544
125.515
124.453
122.643
77.211
77.000
76.789
73.170
40.731
28.695
O
N
OH
O
CF3
3t
13C NMR (151 MHz, CDCl3)
210.0 200.0 190.0 180.0 170.0 160.0 150.0 140.0 130.0 120.0 110.0 100.0 90.0 80.0 70.0 60.0 50.0 40.0 30.0 20.0 10.0 0
X : parts per Million : Carbon13

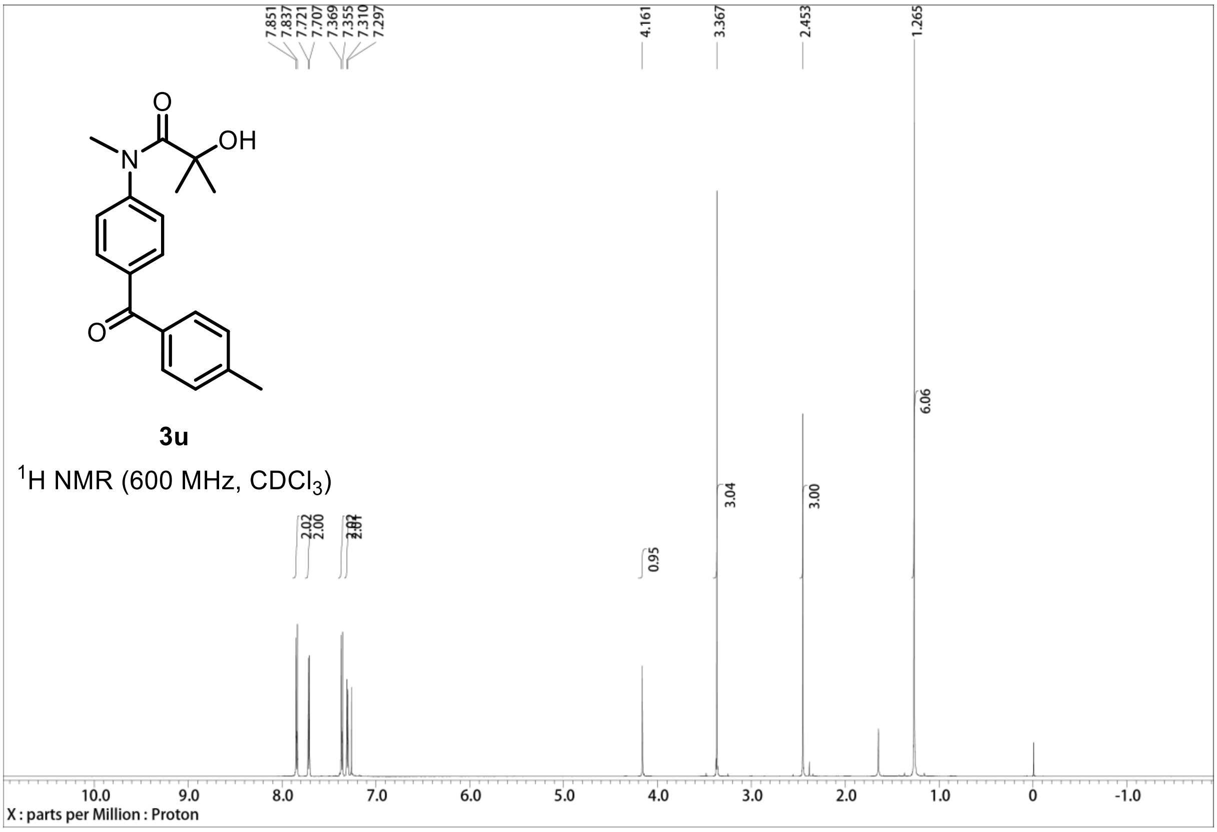
7.851
7.837
7.721
7.707
7.369
7.355
7.310
7.297
4.161
3.367
2.453
1.265
OH
N
O
O
3u
^{1}H NMR (600 MHz, $CDCl_3$)
2.02
2.00
2.02
2.01
0.95
3.04
3.00
6.06
10.0 9.0 8.0 7.0 6.0 5.0 4.0 3.0 2.0 1.0 0 -1.0
X : parts per Million : Proton

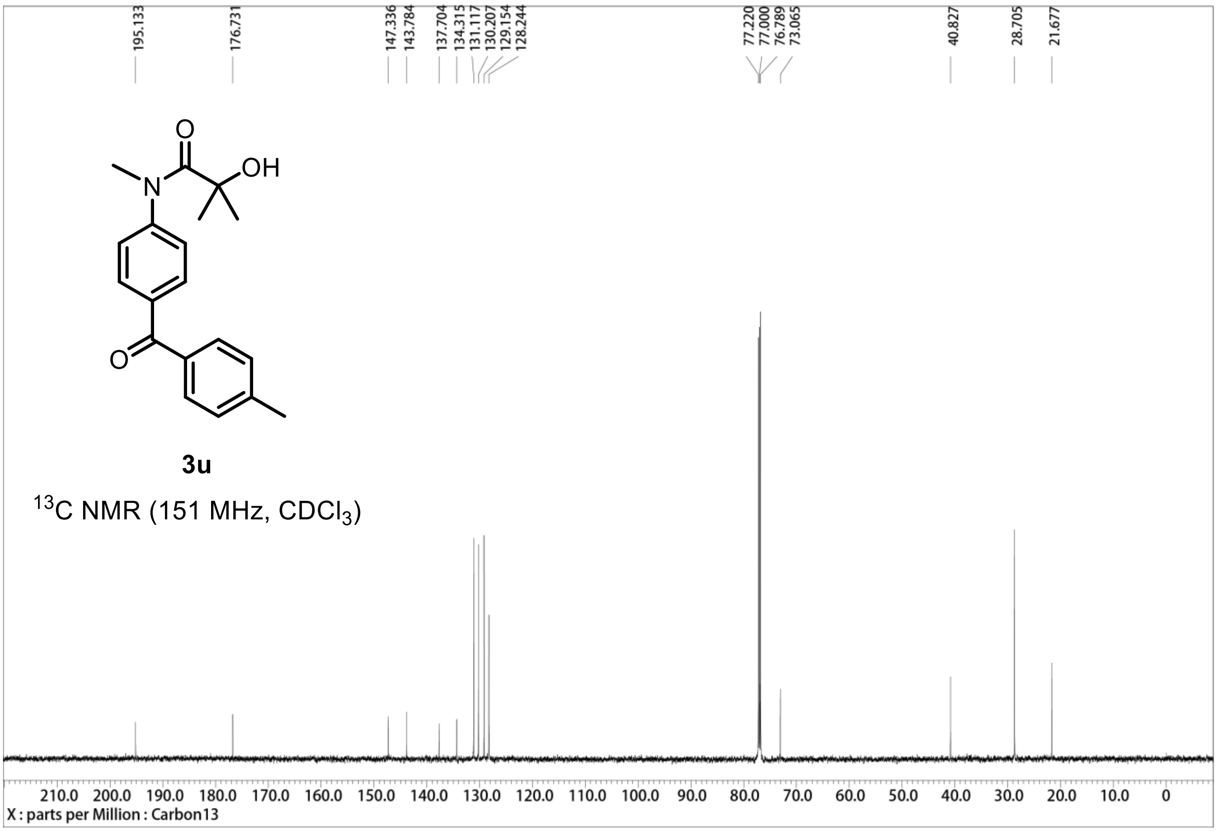
195.133
176.731
147.336
143.784
137.704
134.315
131.117
130.207
129.154
128.244
77.220
77.000
76.789
73.065
40.827
28.705
21.677
OH
N
O
O
3u
^{13}C NMR (151 MHz, $CDCl_3$)
210.0 200.0 190.0 180.0 170.0 160.0 150.0 140.0 130.0 120.0 110.0 100.0 90.0 80.0 70.0 60.0 50.0 40.0 30.0 20.0 10.0 0
X : parts per Million : Carbon13

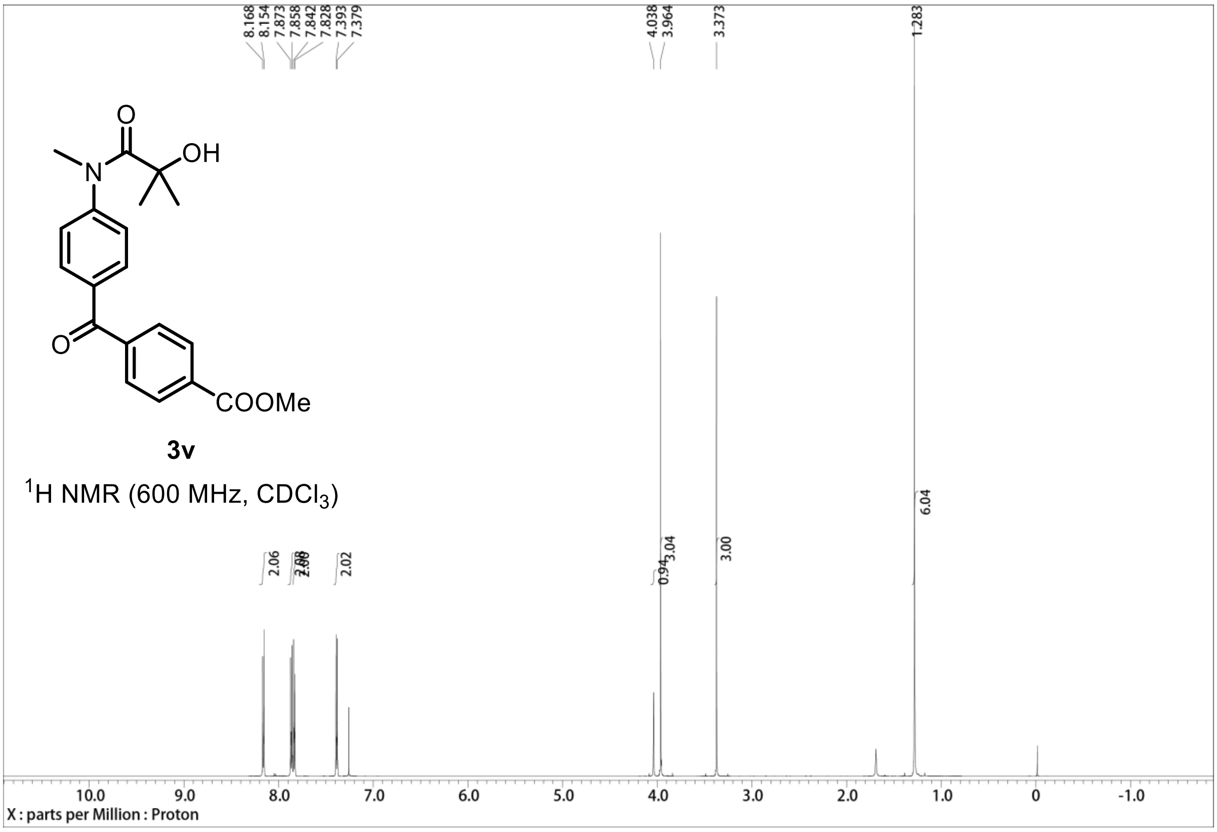
8.168
8.154
7.873
7.858
7.842
7.828
7.393
7.379
4.038
3.964
3.373
1.283
O
N
OH
O
COOMe
3v
^{1}H NMR (600 MHz, CDCl$_3$)
2.06
2.02
0.94
3.04
3.00
6.04
10.0
9.0
8.0
7.0
6.0
5.0
4.0
3.0
2.0
1.0
0
-1.0
X : parts per Million : Proton

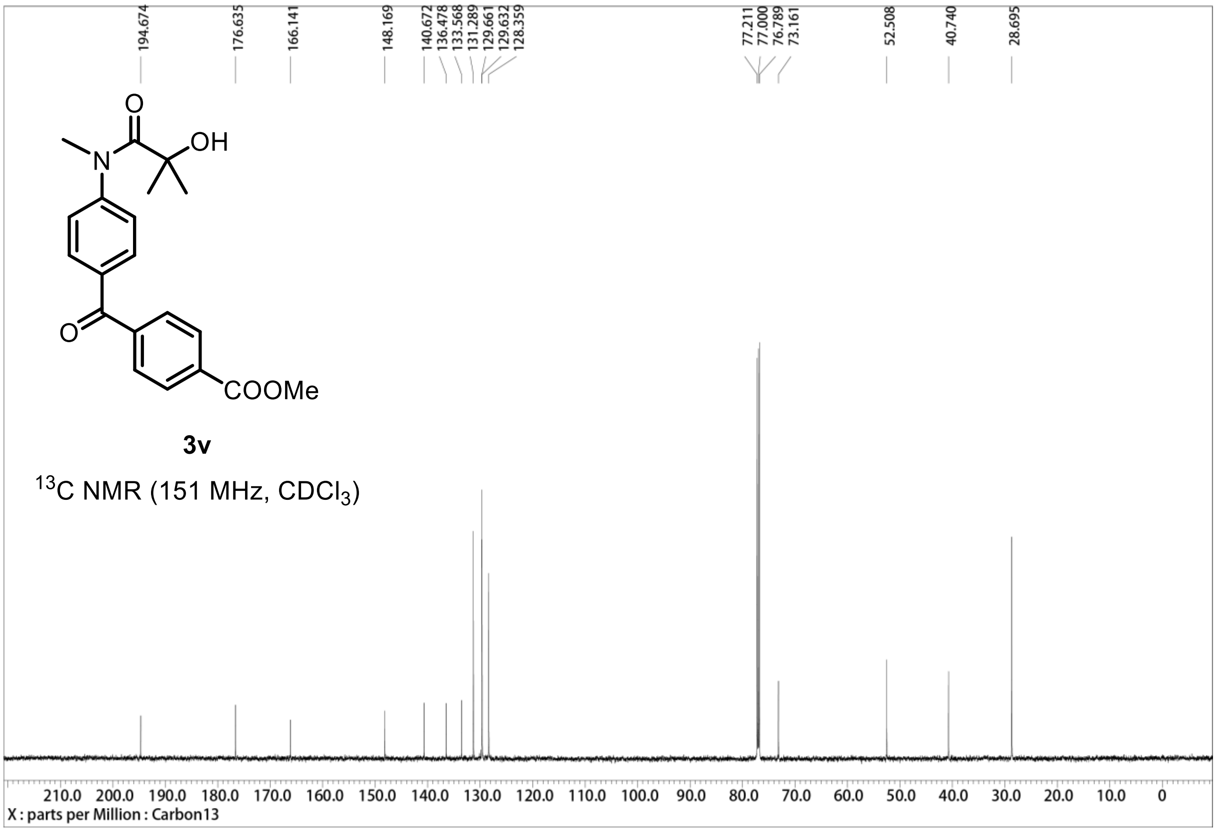
194.674
176.635
166.141
148.169
140.672
136.478
133.568
131.289
129.661
129.632
128.359
77.211
77.000
76.789
73.161
52.508
40.740
28.695
O
N
OH
O
COOMe
3v
^{13}C NMR (151 MHz, CDCl$_3$)
210.0
200.0
190.0
180.0
170.0
160.0
150.0
140.0
130.0
120.0
110.0
100.0
90.0
80.0
70.0
60.0
50.0
40.0
30.0
20.0
10.0
0
X : parts per Million : Carbon13

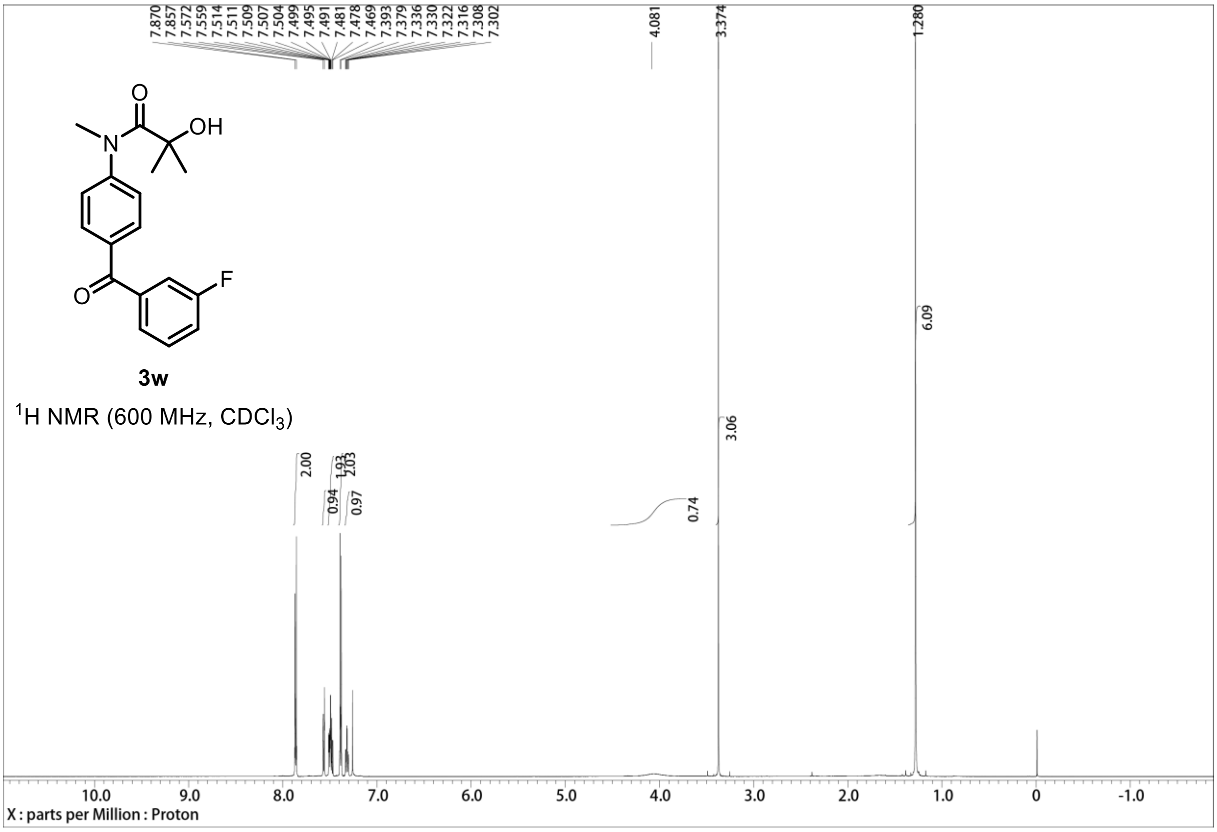
3w
¹H NMR (600 MHz, CDCl₃)
X : parts per Million : Proton

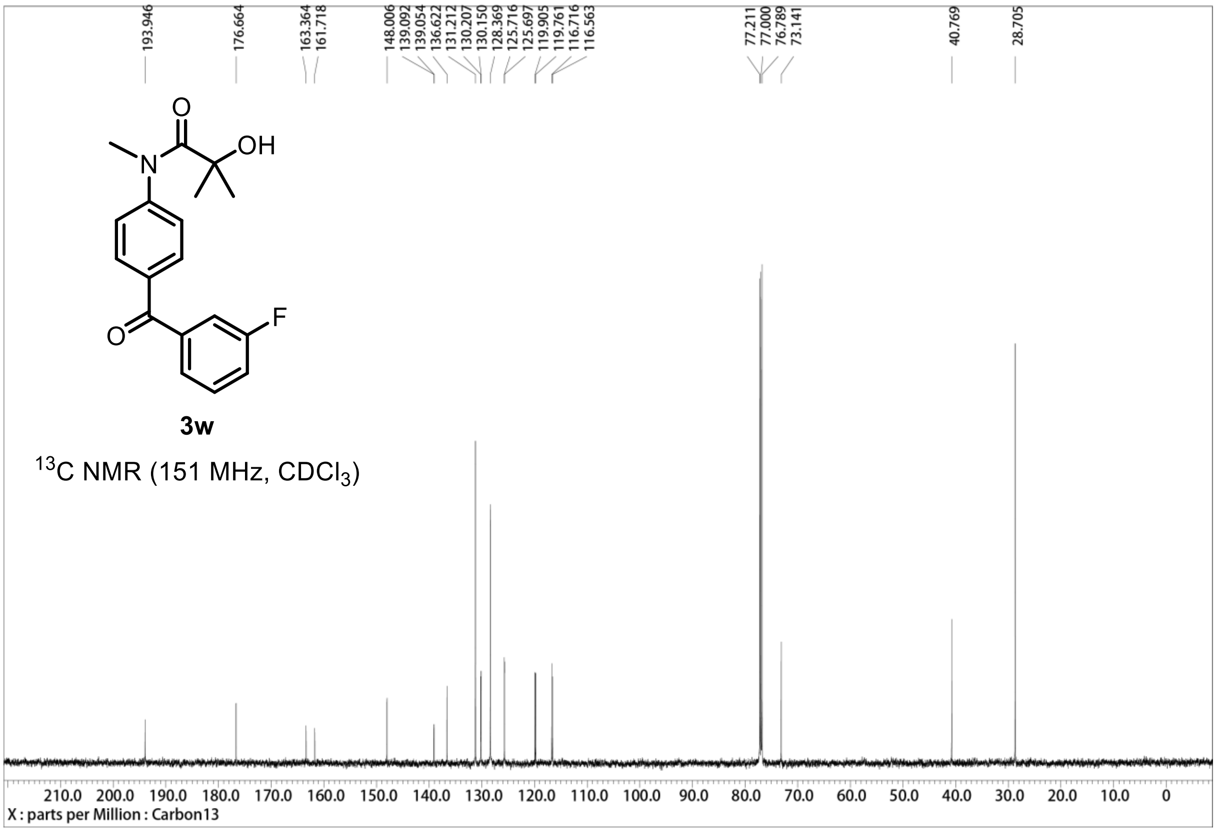
3w
¹³C NMR (151 MHz, CDCl₃)
X : parts per Million : Carbon13

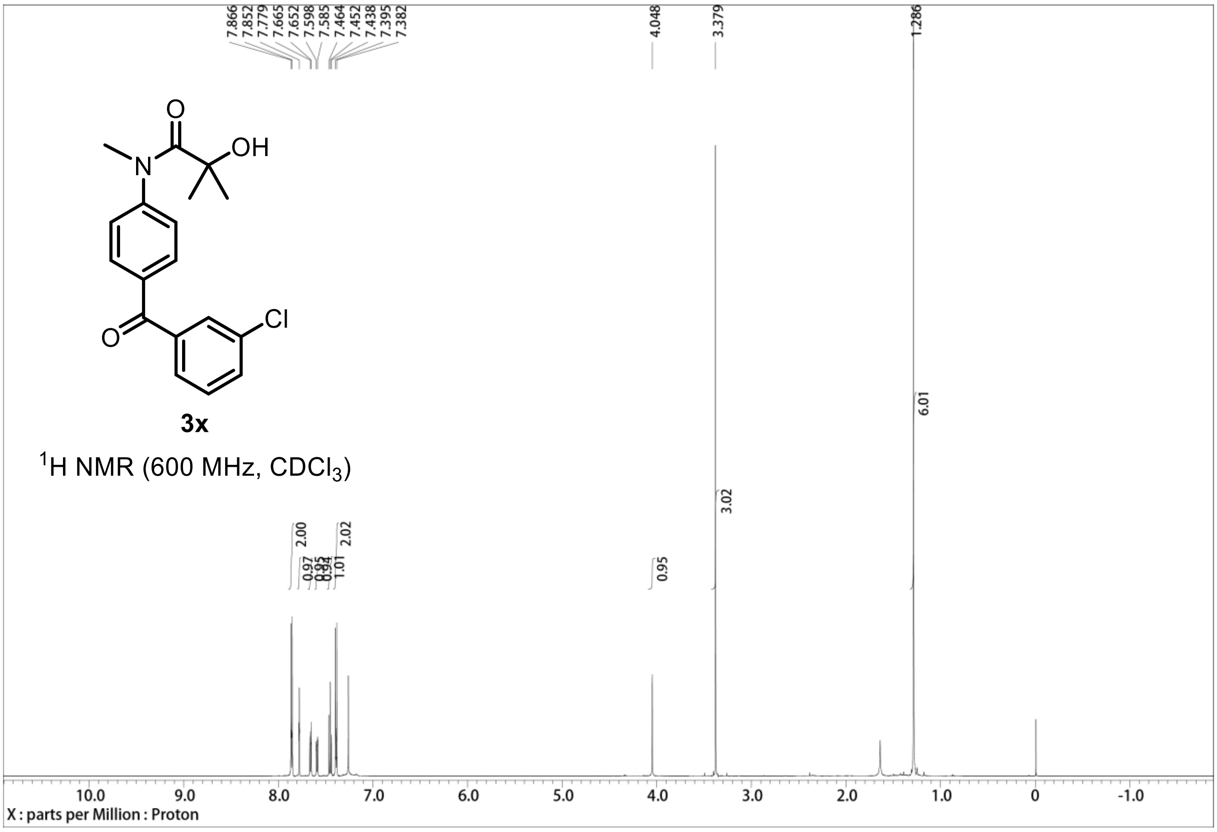
7.866
7.852
7.779
7.665
7.652
7.598
7.585
7.464
7.452
7.438
7.395
7.382
4.048
3.379
1.286
O
N
OH
O
Cl
3x
¹H NMR (600 MHz, CDCl₃)
6.01
3.02
2.00
0.97
1.01
2.02
0.95
10.0
9.0
8.0
7.0
6.0
5.0
4.0
3.0
2.0
1.0
0
-1.0
X : parts per Million : Proton

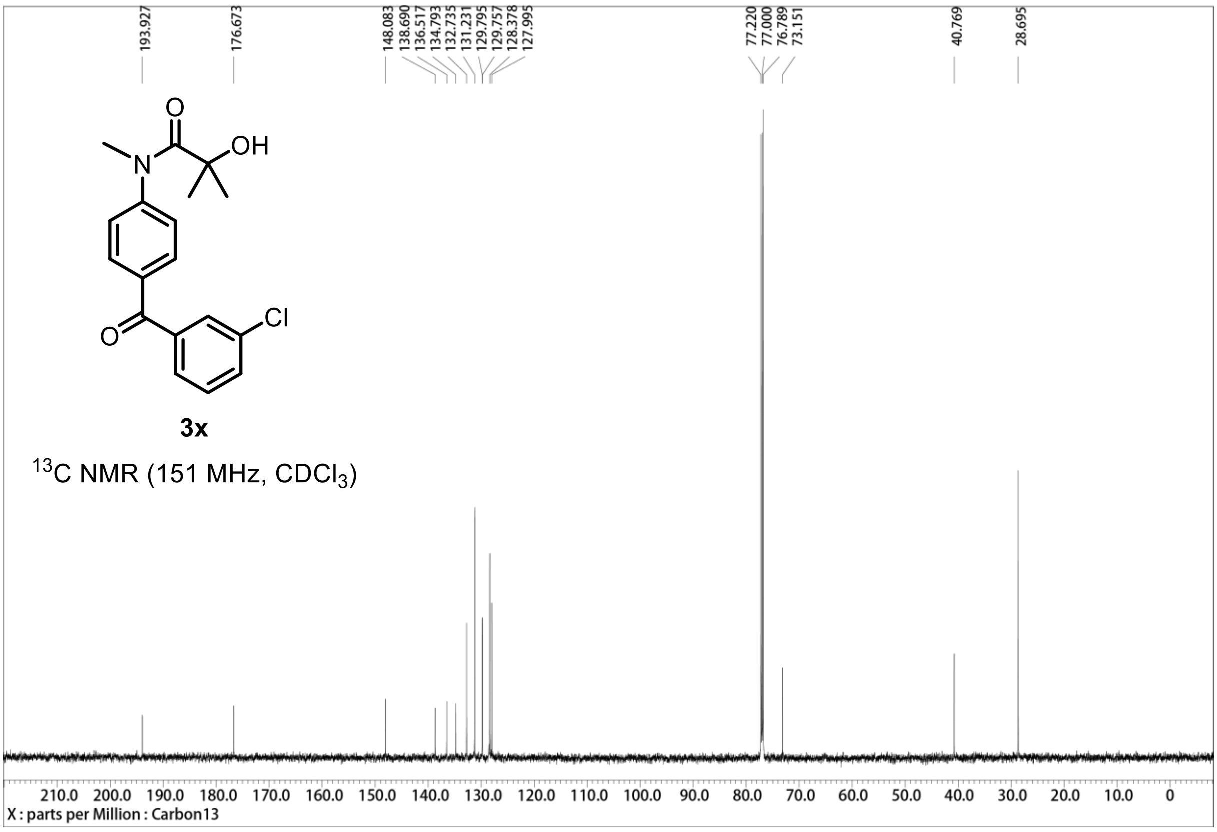
193.927
176.673
148.083
138.690
136.517
134.793
132.735
131.231
129.795
129.757
128.378
127.995
77.220
77.000
76.789
73.151
40.769
28.695
O
N
OH
O
Cl
3x
¹³C NMR (151 MHz, CDCl₃)
210.0
200.0
190.0
180.0
170.0
160.0
150.0
140.0
130.0
120.0
110.0
100.0
90.0
80.0
70.0
60.0
50.0
40.0
30.0
20.0
10.0
0
X : parts per Million : Carbon13

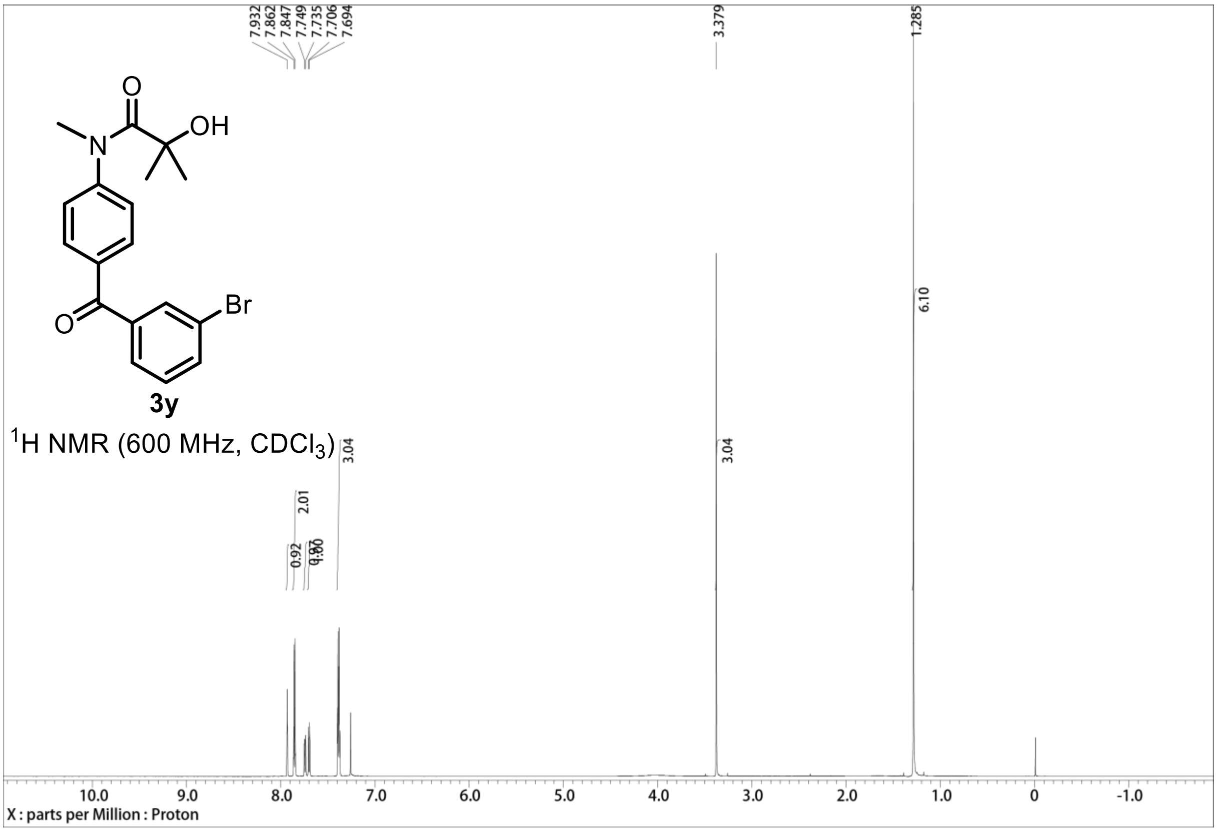
7.932
7.862
7.847
7.749
7.735
7.706
7.694
3.379
1.285
O
N
OH
Br
O
3y
1H NMR (600 MHz, CDCl3)
3.04
2.01
0.92
6.10
X : parts per Million : Proton

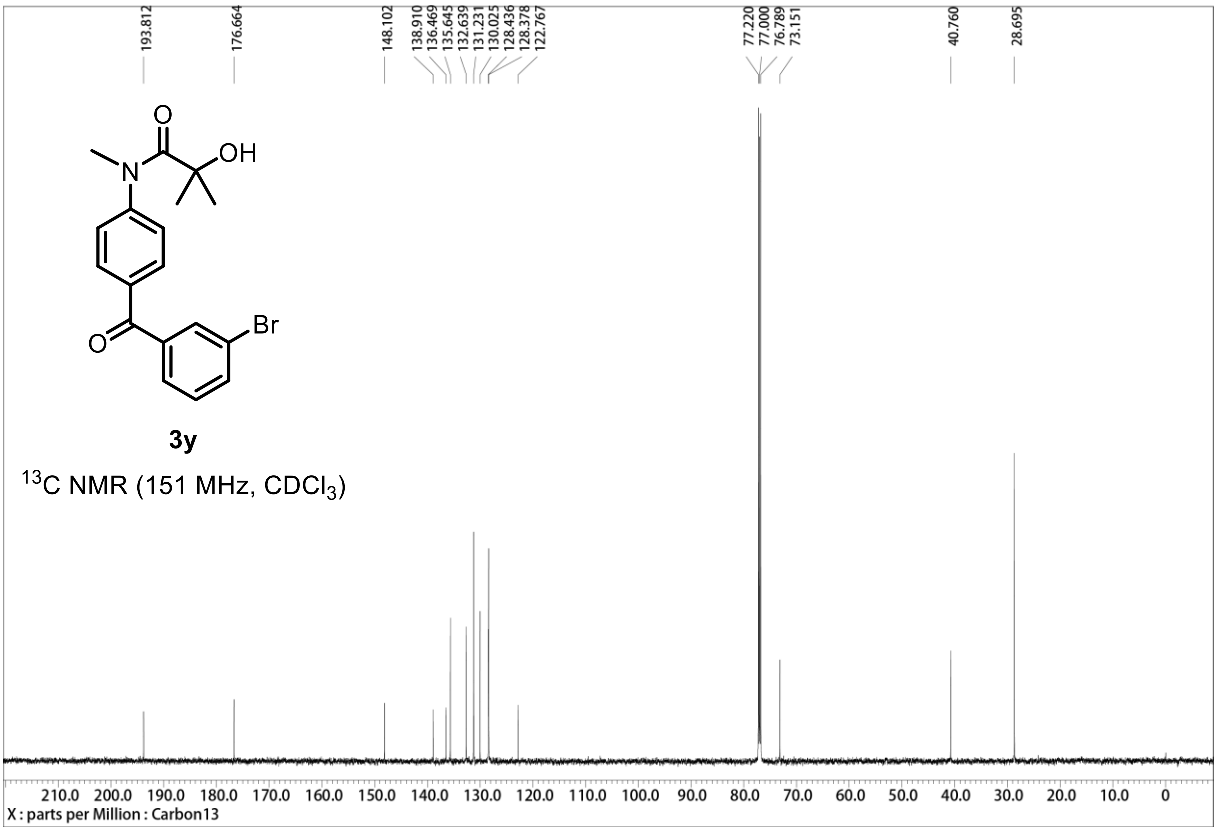
193.812
176.664
148.102
138.910
136.469
135.645
132.639
131.231
130.025
128.436
128.378
122.767
77.220
77.000
76.789
73.151
40.760
28.695
O
N
OH
Br
O
3y
13C NMR (151 MHz, CDCl3)
X : parts per Million : Carbon13

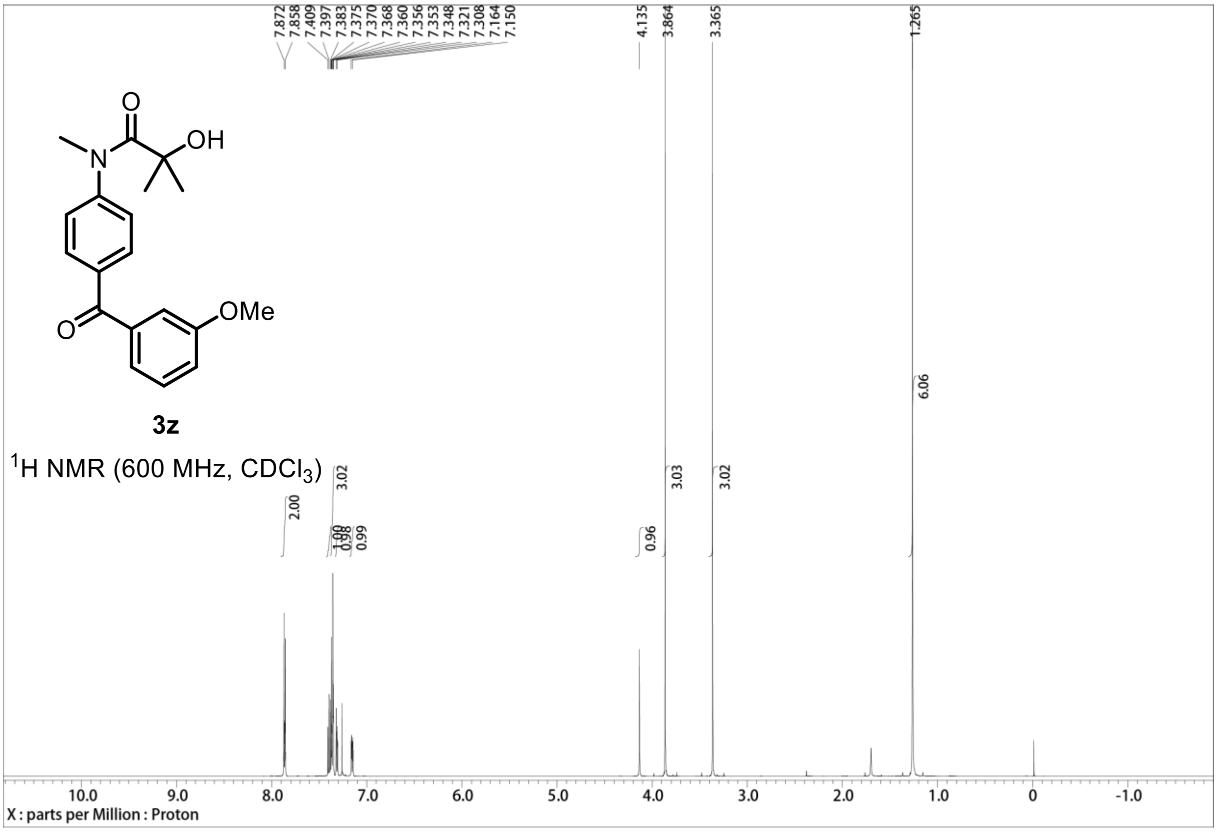
7.872
7.858
7.409
7.397
7.383
7.375
7.370
7.368
7.360
7.356
7.353
7.348
7.321
7.308
7.164
7.150
4.135
3.864
3.365
1.265
OH
O
N
O
OMe
3z
¹H NMR (600 MHz, CDCl₃)
2.00
3.02
1.00
0.98
0.99
0.96
3.03
3.02
6.06
10.0
9.0
8.0
7.0
6.0
5.0
4.0
3.0
2.0
1.0
0
-1.0
X : parts per Million : Proton

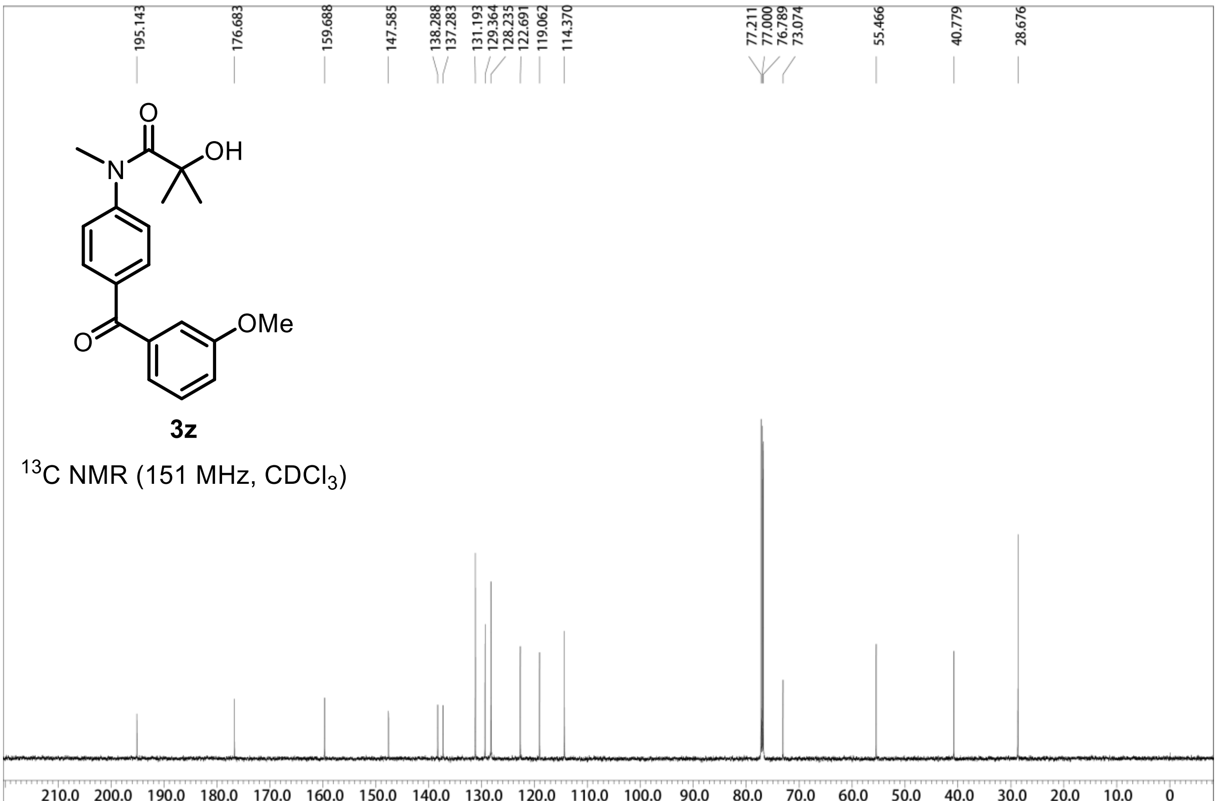
195.143
176.683
159.688
147.585
138.288
137.283
131.193
129.364
128.235
122.691
119.062
114.370
77.211
77.000
76.789
73.074
55.466
40.779
28.676
OH
O
N
O
OMe
3z
¹³C NMR (151 MHz, CDCl₃)
210.0
200.0
190.0
180.0
170.0
160.0
150.0
140.0
130.0
120.0
110.0
100.0
90.0
80.0
70.0
60.0
50.0
40.0
30.0
20.0
10.0
0
X : parts per Million : Carbon13

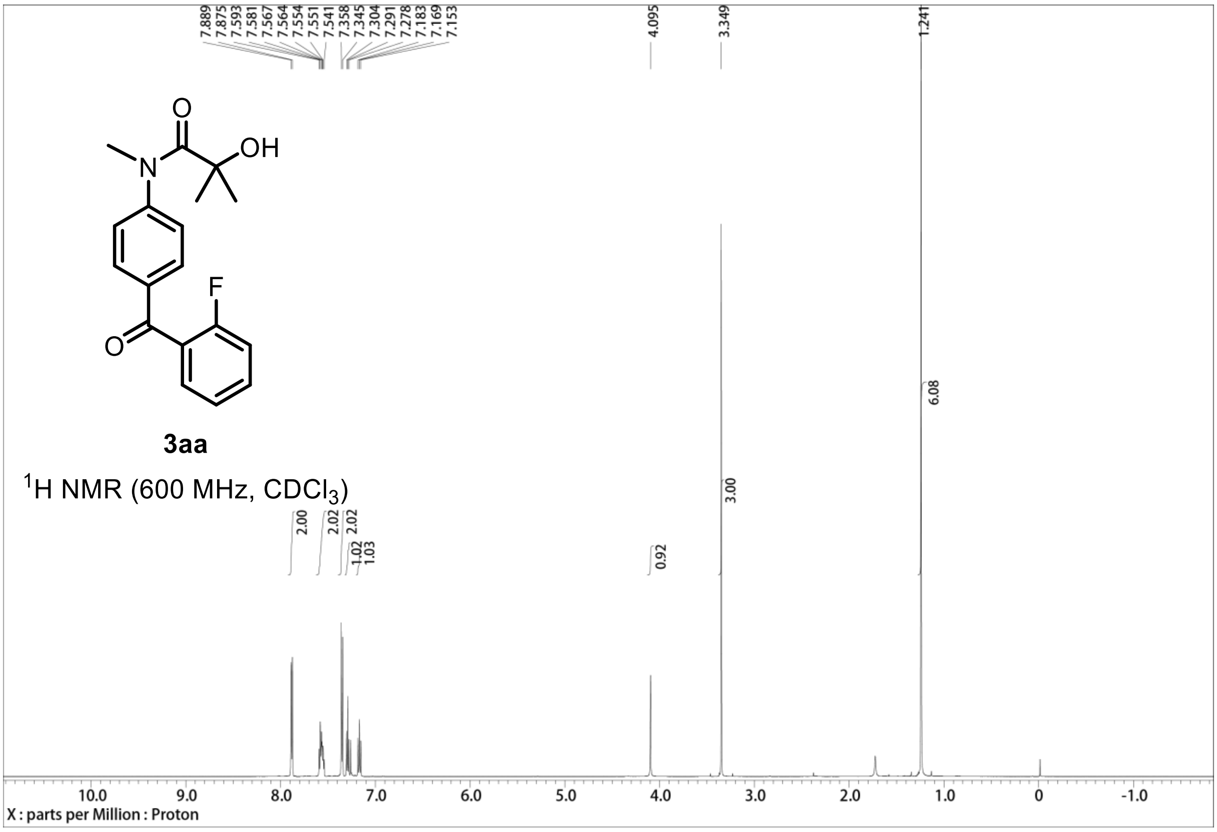
7.889
7.875
7.593
7.581
7.567
7.564
7.554
7.551
7.541
7.358
7.345
7.304
7.291
7.278
7.183
7.169
7.153
4.095
3.349
1.241
3aa
¹H NMR (600 MHz, CDCl₃)
2.00
2.02
2.02
1.02
1.03
0.92
3.00
6.08
10.0
9.0
8.0
7.0
6.0
5.0
4.0
3.0
2.0
1.0
0
-1.0
X : parts per Million : Proton

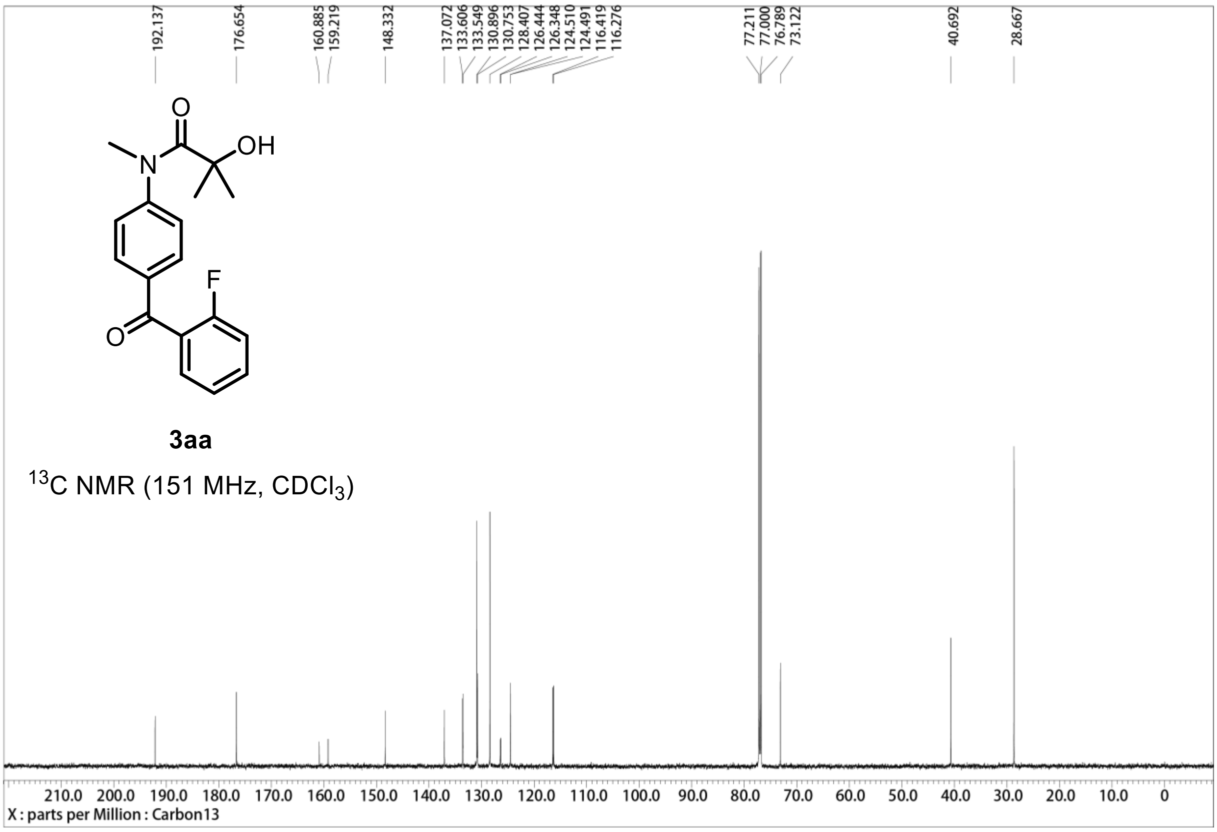
192.137
176.654
160.885
159.219
148.332
137.072
133.606
133.549
130.896
130.753
128.407
126.444
126.348
124.510
124.491
116.419
116.276
77.211
77.000
76.789
73.122
40.692
28.667
3aa
¹³C NMR (151 MHz, CDCl₃)
210.0
200.0
190.0
180.0
170.0
160.0
150.0
140.0
130.0
120.0
110.0
100.0
90.0
80.0
70.0
60.0
50.0
40.0
30.0
20.0
10.0
0
X : parts per Million : Carbon13

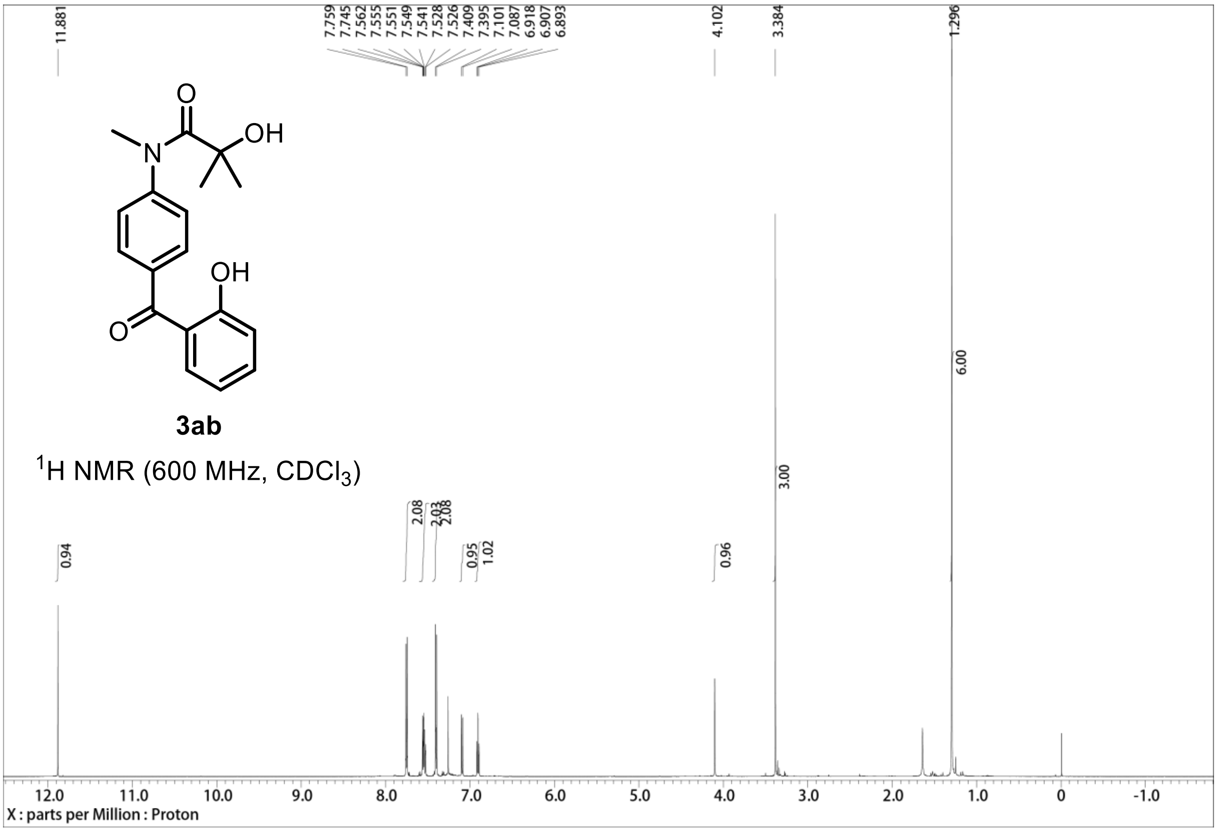

3ab
^{1}H NMR (600 MHz, CDCl$_3$)
X : parts per Million : Proton

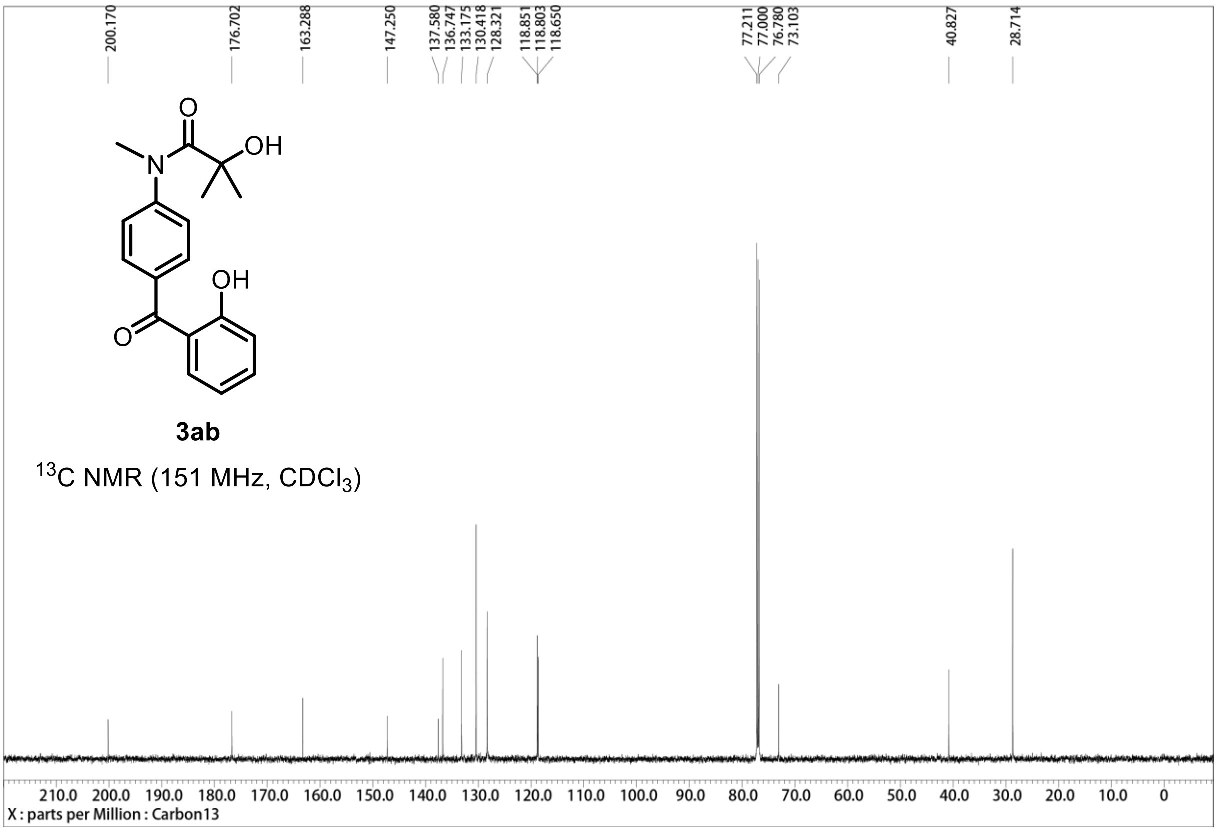

3ab
^{13}C NMR (151 MHz, CDCl$_3$)
X : parts per Million : Carbon13

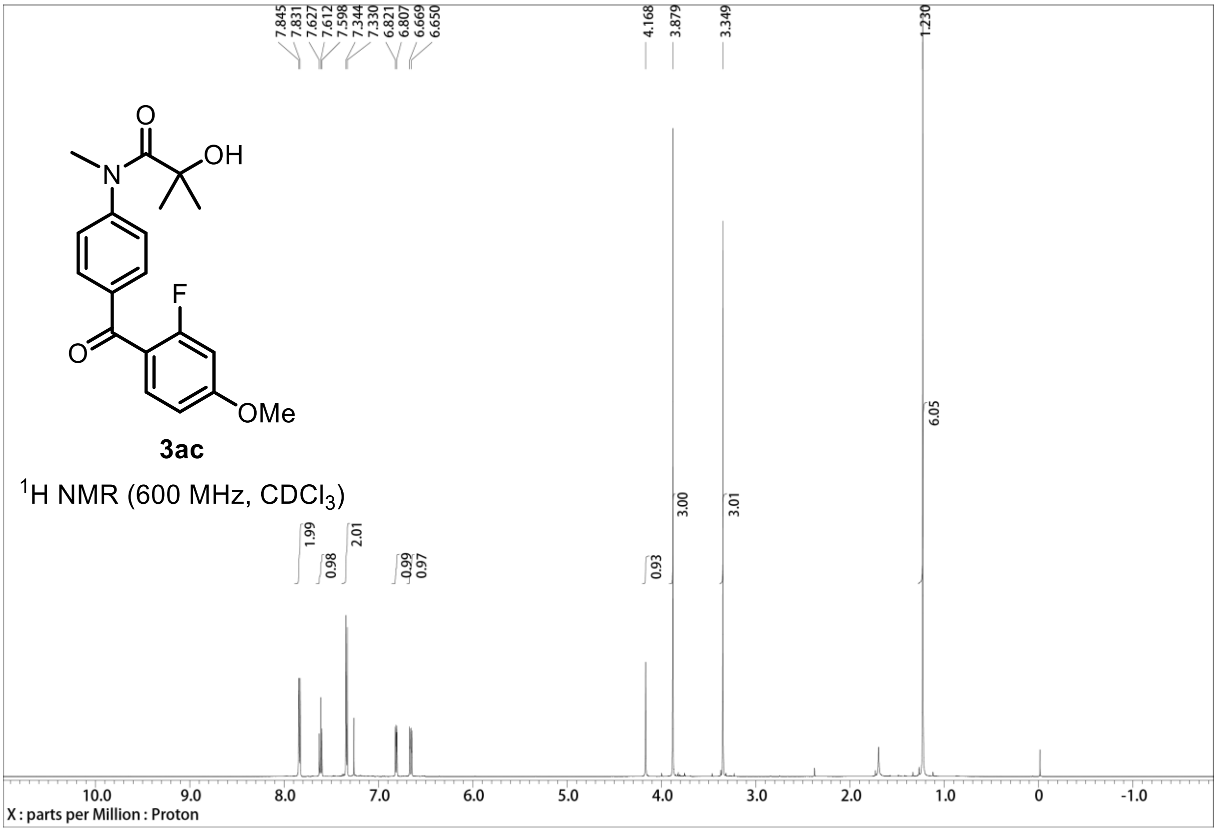
7.845
7.831
7.627
7.612
7.598
7.344
7.330
6.821
6.807
6.669
6.650
4.168
3.879
3.349
1.230
OH
N
O
F
O
OMe
3ac
1H NMR (600 MHz, CDCl3)
1.99
0.98
2.01
0.99
0.97
0.93
3.00
3.01
6.05
X : parts per Million : Proton

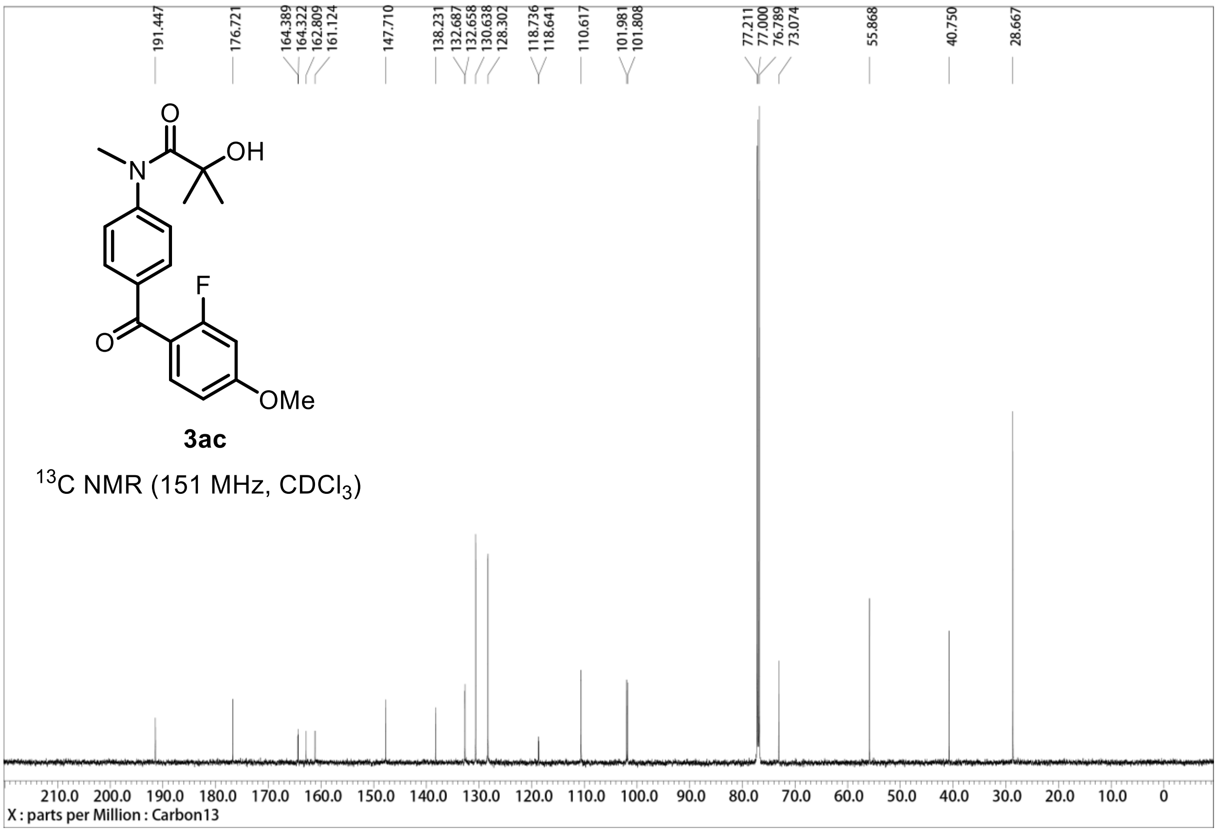
191.447
176.721
164.389
164.322
162.809
161.124
147.710
138.231
132.687
132.658
130.638
128.302
118.736
118.641
110.617
101.981
101.808
77.211
77.000
76.789
73.074
55.868
40.750
28.667
3ac
13C NMR (151 MHz, CDCl3)
X : parts per Million : Carbon13

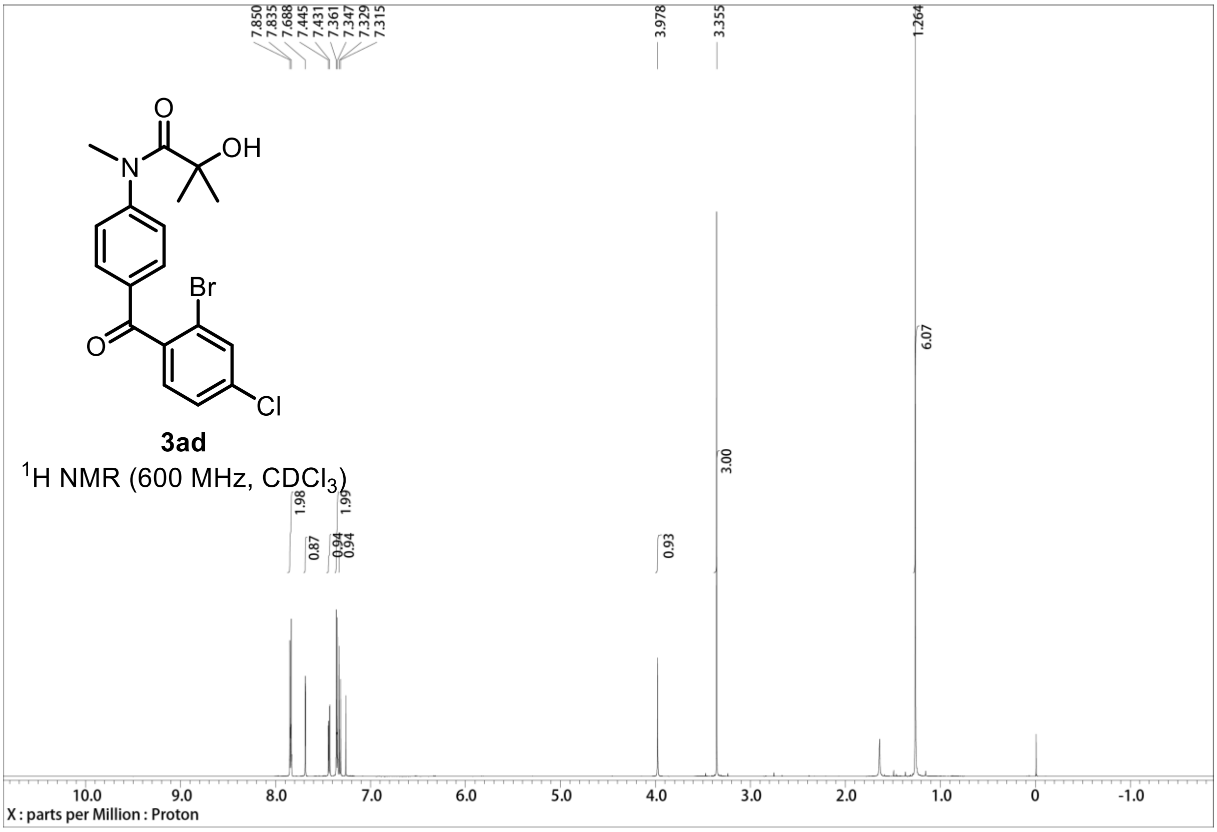

7.850
7.835
7.688
7.445
7.431
7.361
7.347
7.329
7.315
3.978
3.355
1.264
O
N
OH
Br
O
Cl
3ad
^{1}H NMR (600 MHz, CDCl$_3$)
1.98
0.87
0.94
0.94
1.99
0.93
3.00
6.07
10.0
9.0
8.0
7.0
6.0
5.0
4.0
3.0
2.0
1.0
0
-1.0
X : parts per Million : Proton

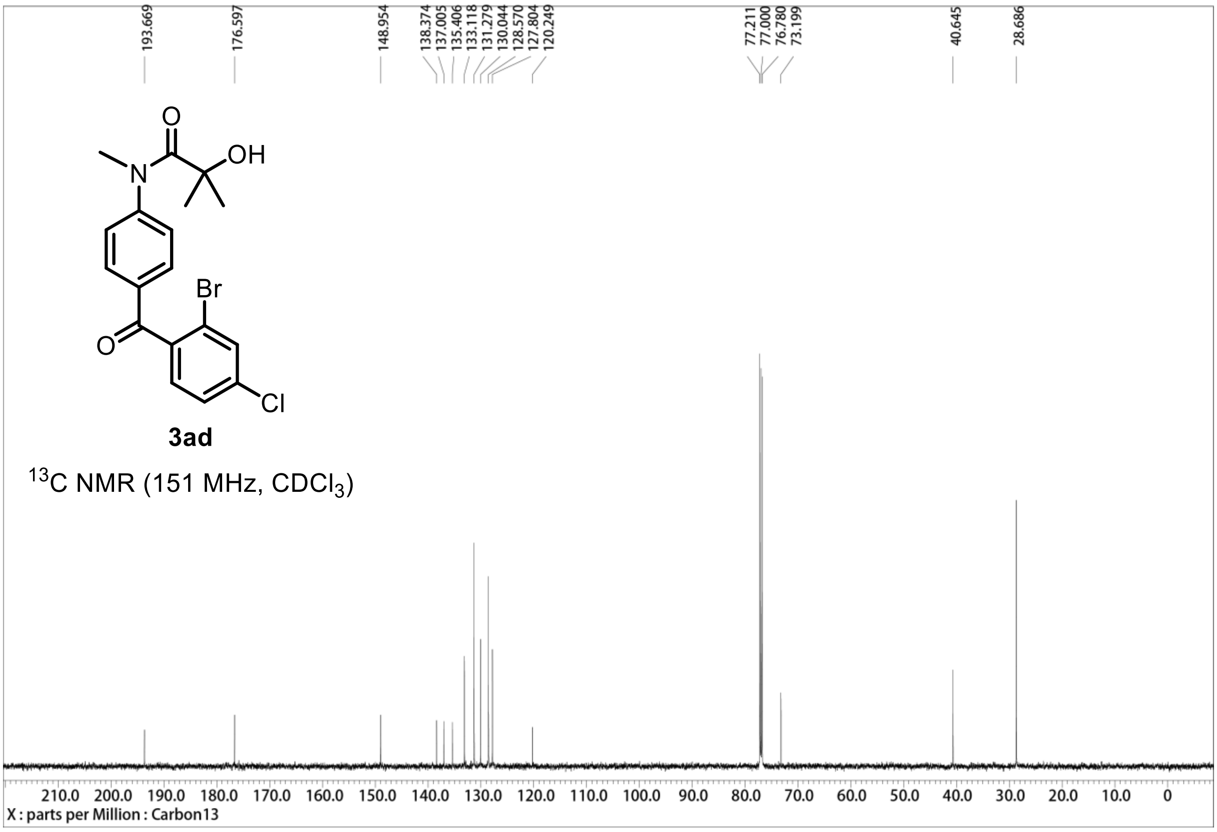

193.669
176.597
148.954
138.374
137.005
135.406
133.118
131.279
130.044
128.570
127.804
120.249
77.211
77.000
76.780
73.199
40.645
28.686
O
N
OH
Br
O
Cl
3ad
^{13}C NMR (151 MHz, CDCl$_3$)
210.0
200.0
190.0
180.0
170.0
160.0
150.0
140.0
130.0
120.0
110.0
100.0
90.0
80.0
70.0
60.0
50.0
40.0
30.0
20.0
10.0
0
X : parts per Million : Carbon13

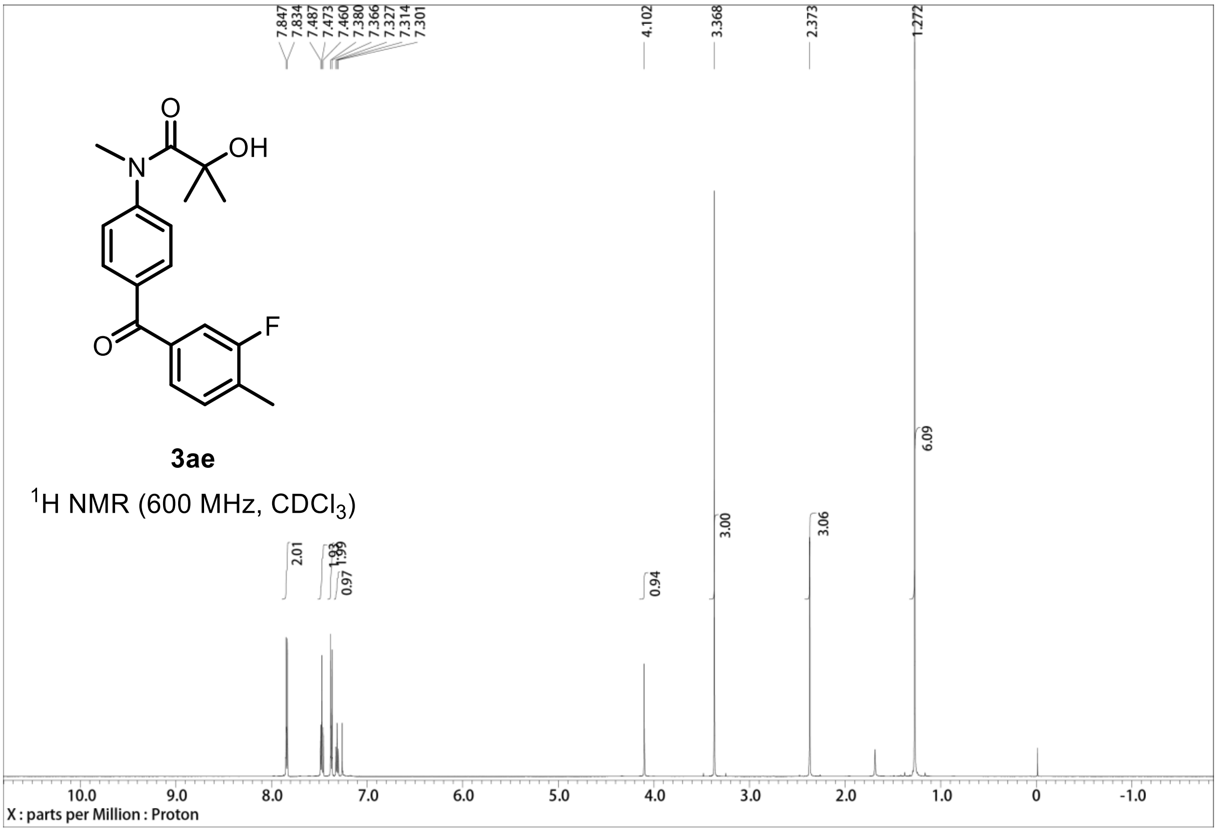

7.847
7.834
7.487
7.473
7.460
7.380
7.366
7.327
7.314
7.301
4.102
3.368
2.373
1.272
3ae
^{1}H NMR (600 MHz, CDCl$_3$)
2.01
1.93
1.99
0.97
0.94
3.00
3.06
6.09
X : parts per Million : Proton

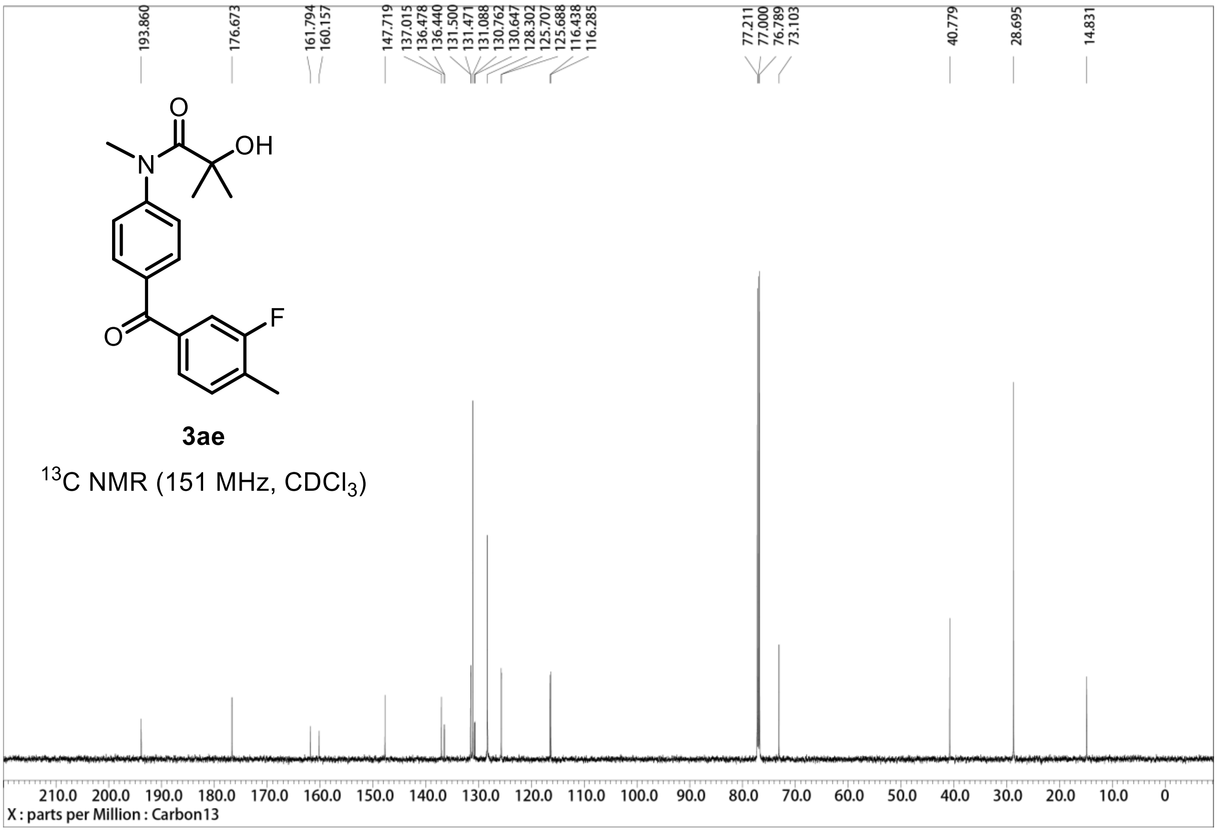

193.860
176.673
161.794
160.157
147.719
137.015
136.478
136.440
131.500
131.471
131.088
130.762
130.647
128.302
125.707
125.688
116.438
116.285
77.211
77.000
76.789
73.103
40.779
28.695
14.831
3ae
^{13}C NMR (151 MHz, CDCl$_3$)
X : parts per Million : Carbon13

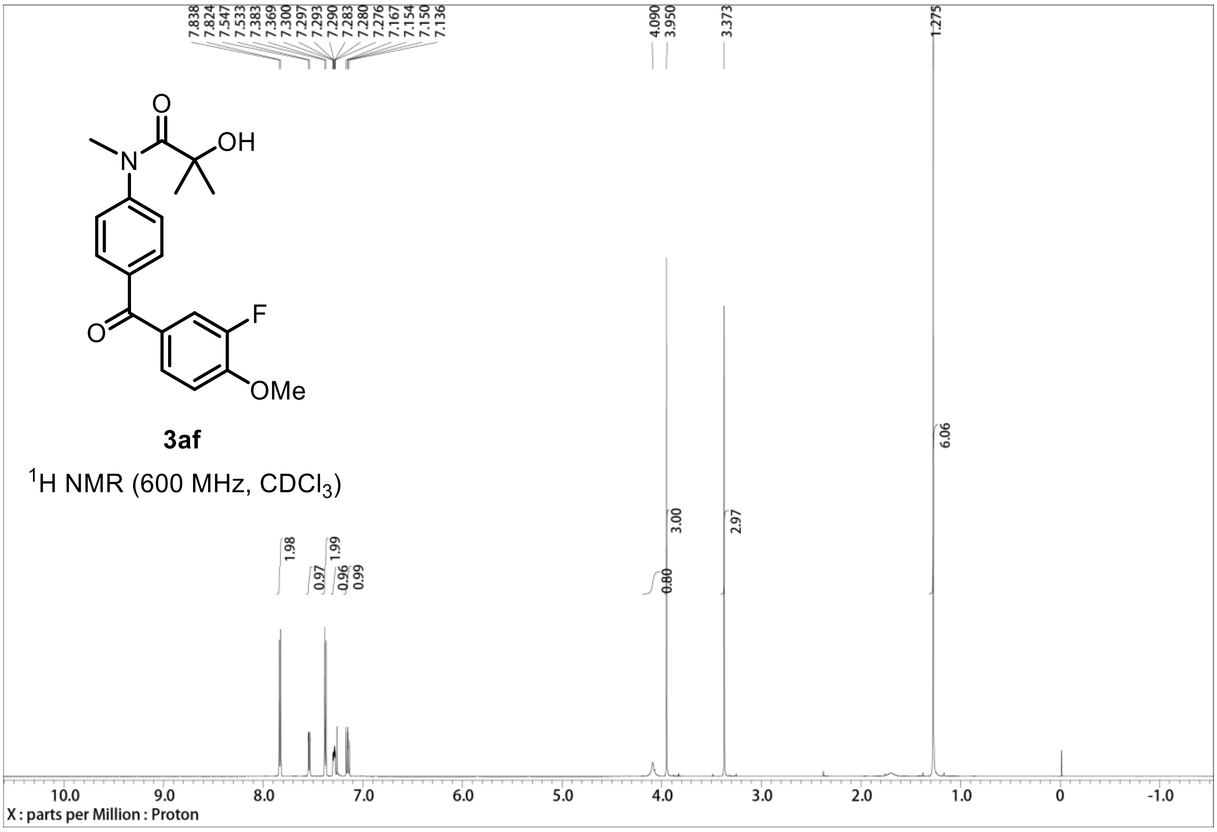
7.838
7.824
7.547
7.533
7.383
7.369
7.300
7.297
7.293
7.290
7.283
7.280
7.276
7.167
7.154
7.150
7.136
4.090
3.950
3.373
1.275
O
N
OH
O
F
OMe
3af
1H NMR (600 MHz, CDCl3)
1.98
0.97
1.99
0.96
0.99
0.80
3.00
2.97
6.06
10.0
9.0
8.0
7.0
6.0
5.0
4.0
3.0
2.0
1.0
0
-1.0
X : parts per Million : Proton

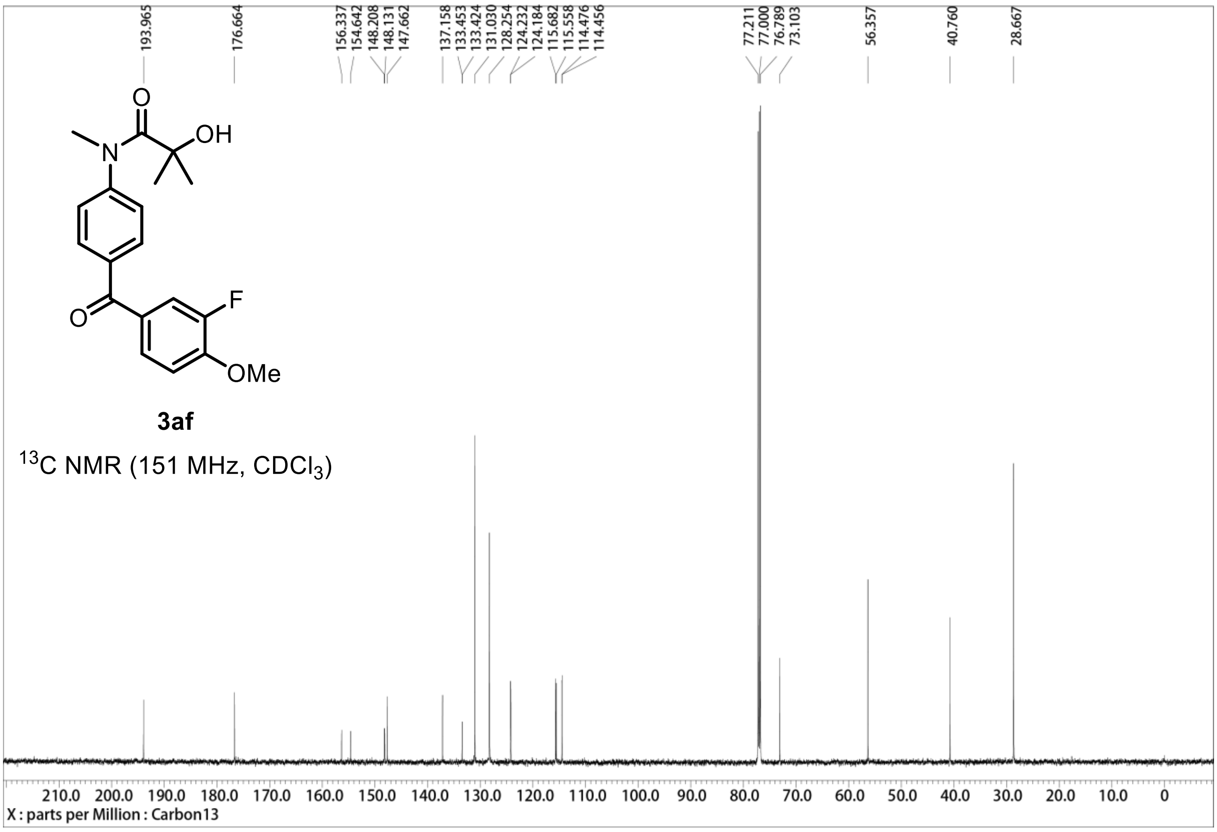
193.965
176.664
156.337
154.642
148.208
148.131
147.662
137.158
133.453
133.424
131.030
128.254
124.232
124.184
115.682
115.558
114.476
114.456
77.211
77.000
76.789
73.103
56.357
40.760
28.667
O
N
OH
O
F
OMe
3af
13C NMR (151 MHz, CDCl3)
210.0
200.0
190.0
180.0
170.0
160.0
150.0
140.0
130.0
120.0
110.0
100.0
90.0
80.0
70.0
60.0
50.0
40.0
30.0
20.0
10.0
0
X : parts per Million : Carbon13

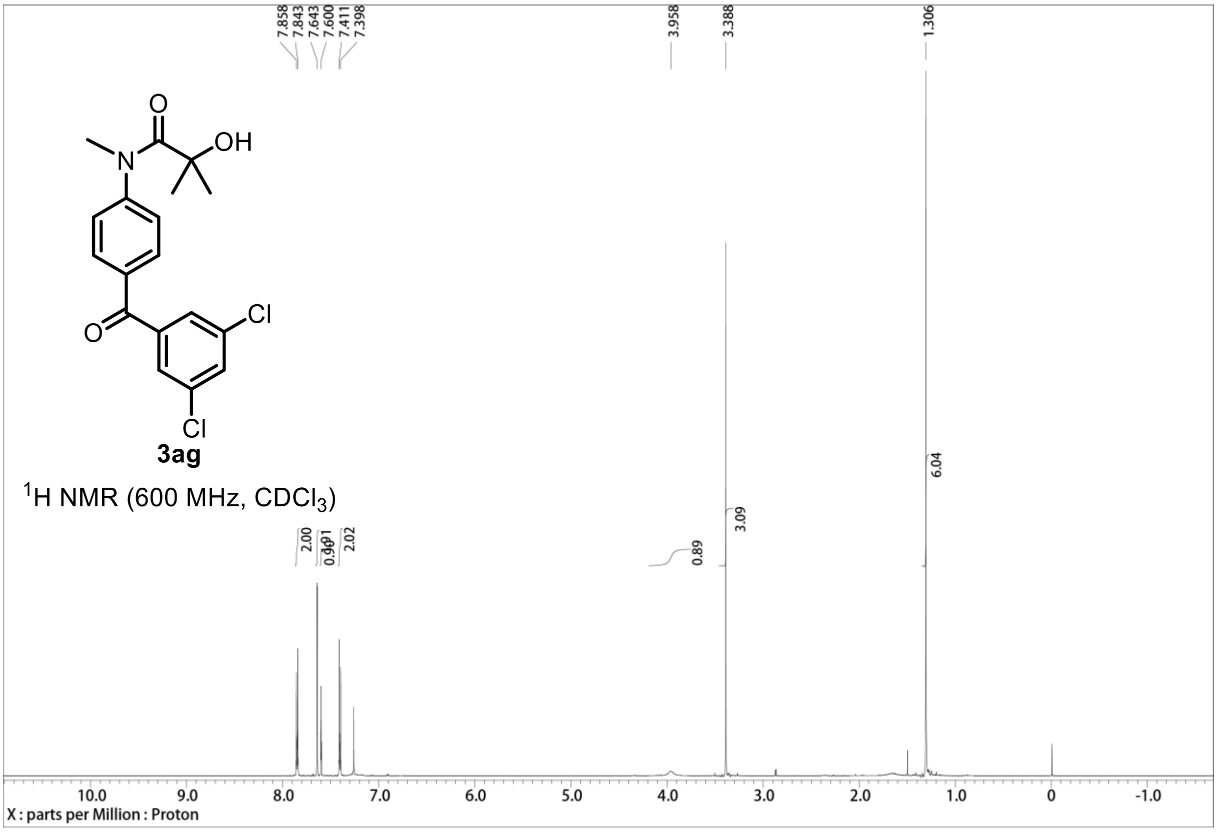
7.858
7.843
7.643
7.600
7.411
7.398
3.958
3.388
1.306
O
N
OH
O
Cl
Cl
3ag
^{1}H NMR (600 MHz, $CDCl_3$)
2.00
1.01
0.96
2.02
0.89
3.09
6.04
10.0 9.0 8.0 7.0 6.0 5.0 4.0 3.0 2.0 1.0 0 -1.0
X : parts per Million : Proton

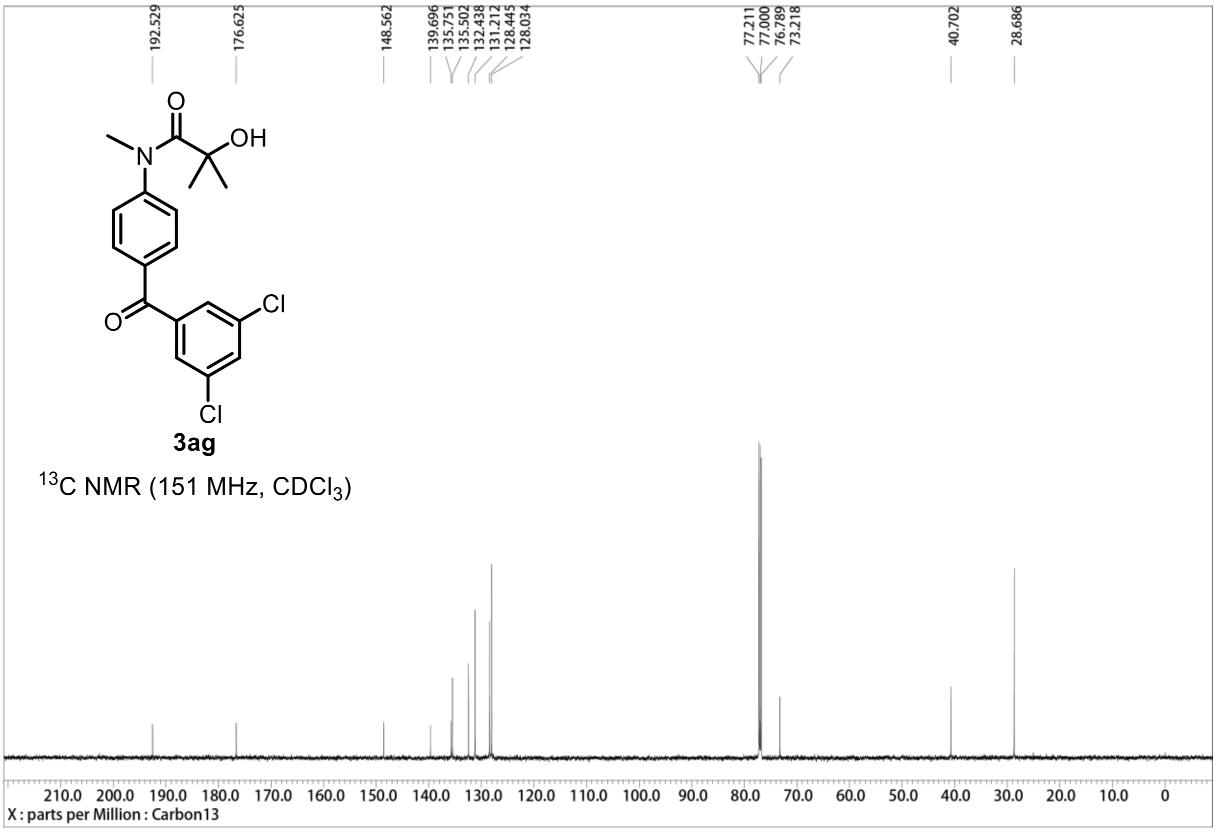
192.529
176.625
148.562
139.696
135.751
135.502
132.438
131.212
128.445
128.034
77.211
77.000
76.789
73.218
40.702
28.686
O
N
OH
O
Cl
Cl
3ag
^{13}C NMR (151 MHz, $CDCl_3$)
210.0 200.0 190.0 180.0 170.0 160.0 150.0 140.0 130.0 120.0 110.0 100.0 90.0 80.0 70.0 60.0 50.0 40.0 30.0 20.0 10.0 0
X : parts per Million : Carbon13

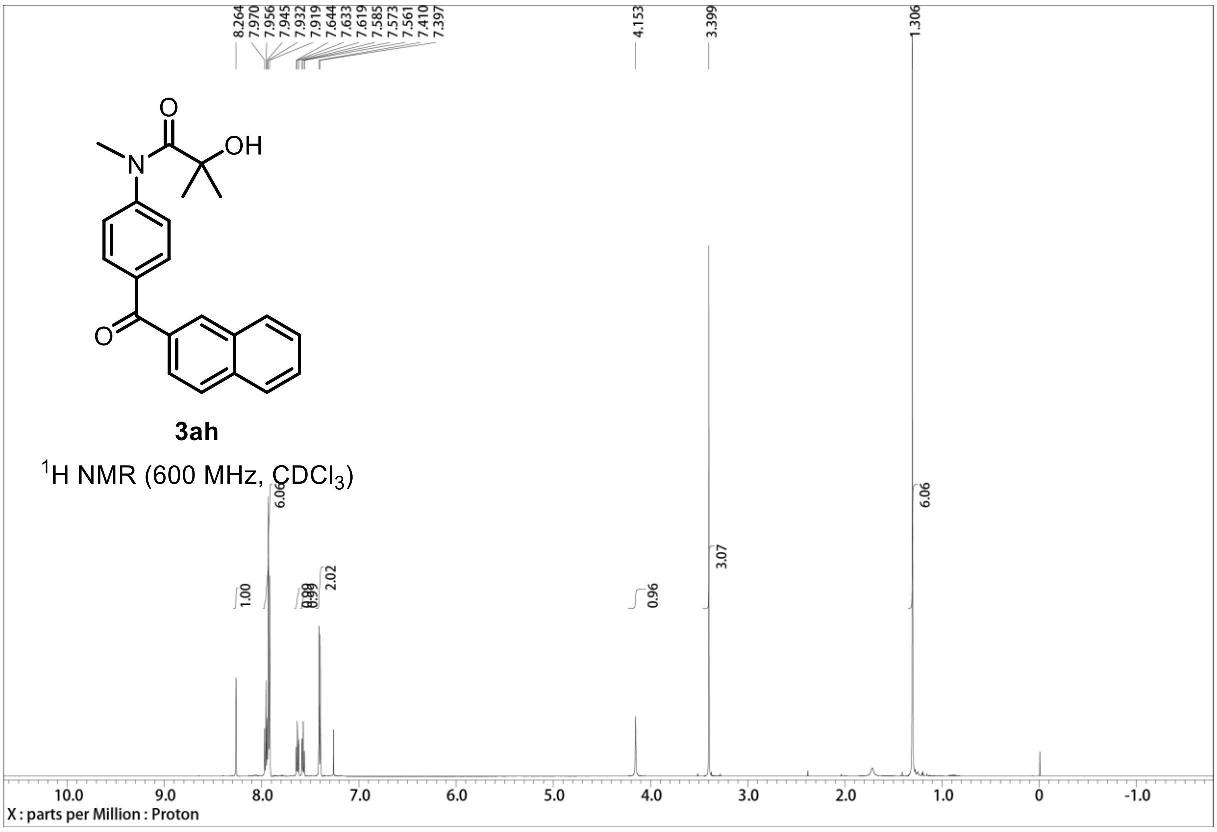
8.264
7.970
7.956
7.945
7.932
7.919
7.644
7.633
7.619
7.585
7.573
7.561
7.410
7.397
4.153
3.399
1.306
OH
N
O
O
3ah
1H NMR (600 MHz, CDCl3)
1.00
6.06
2.02
0.96
3.07
6.06
10.0
9.0
8.0
7.0
6.0
5.0
4.0
3.0
2.0
1.0
0
-1.0
X : parts per Million : Proton

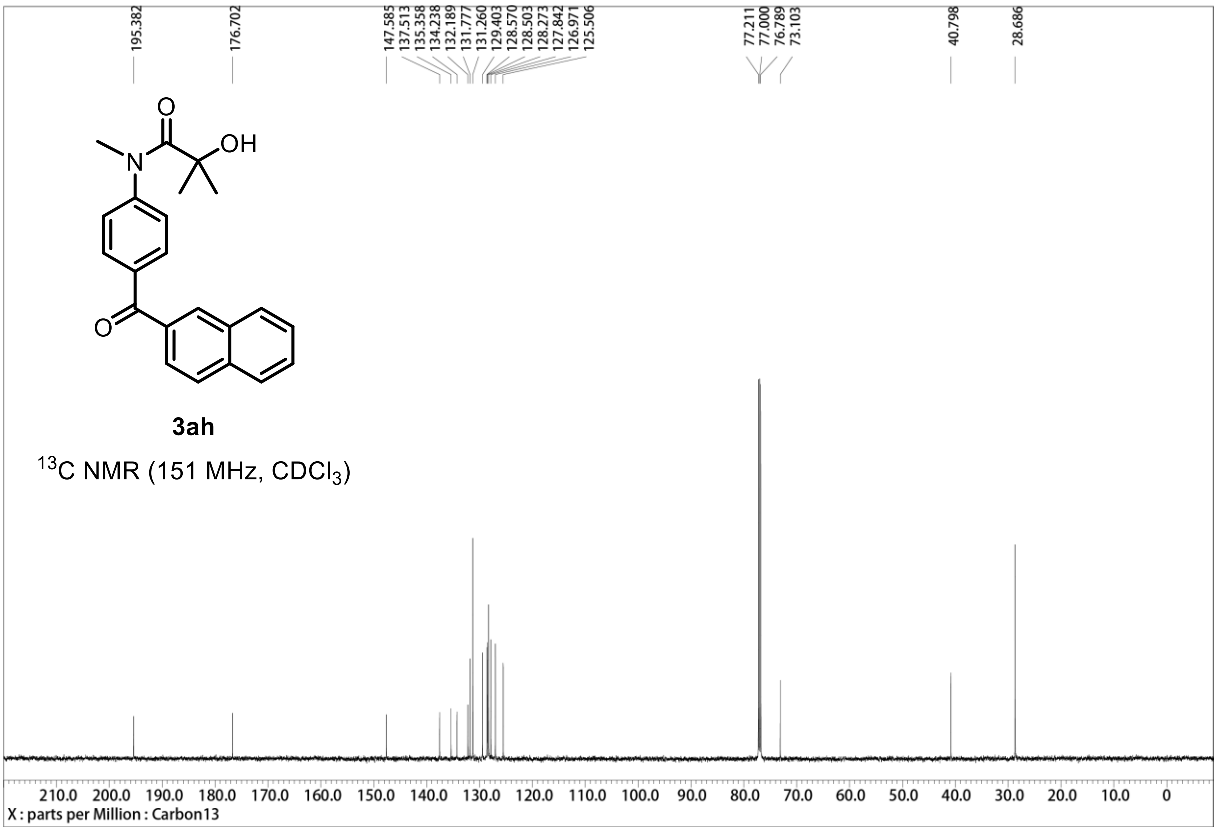
195.382
176.702
147.585
137.513
135.358
134.238
132.189
131.777
131.260
129.403
128.570
128.503
128.273
127.842
126.971
125.506
77.211
77.000
76.789
73.103
40.798
28.686
OH
N
O
O
3ah
13C NMR (151 MHz, CDCl3)
210.0
200.0
190.0
180.0
170.0
160.0
150.0
140.0
130.0
120.0
110.0
100.0
90.0
80.0
70.0
60.0
50.0
40.0
30.0
20.0
10.0
0
X : parts per Million : Carbon13

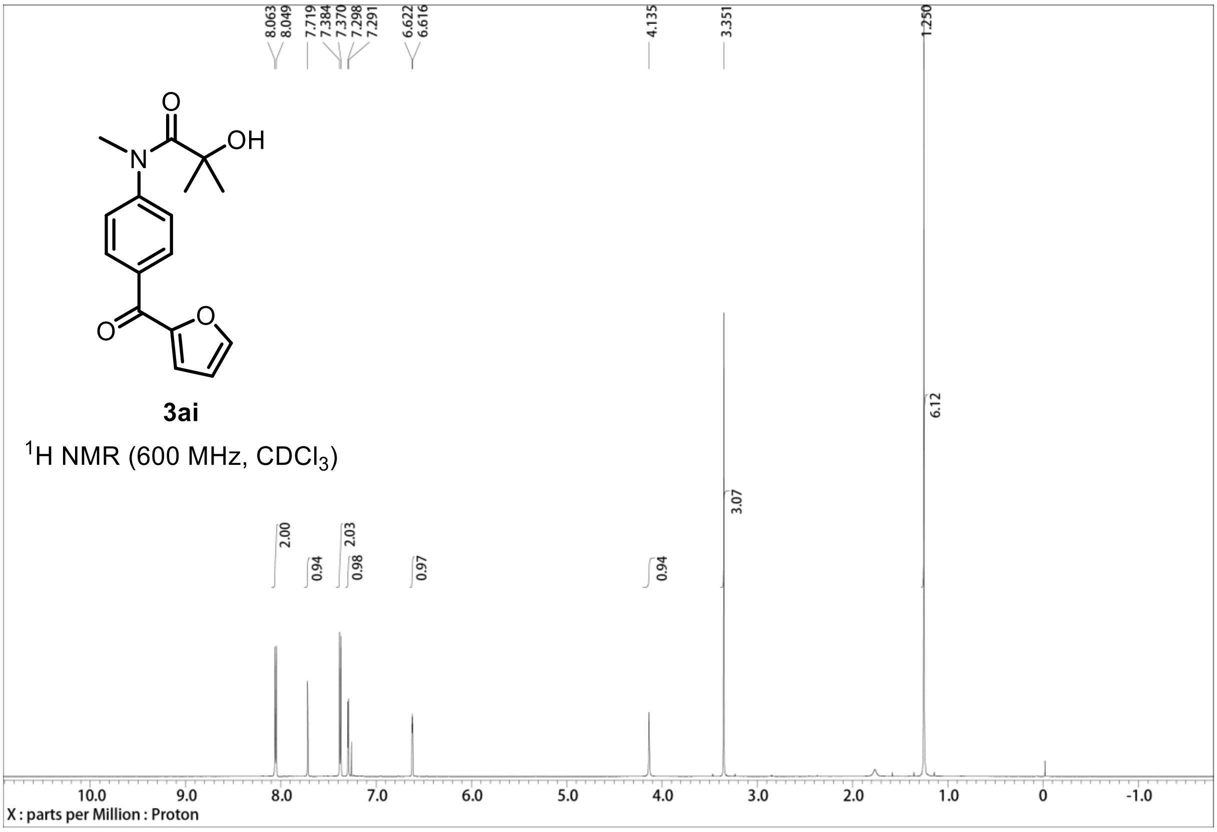

3ai

^{1}H NMR (600 MHz, $CDCl_3$)

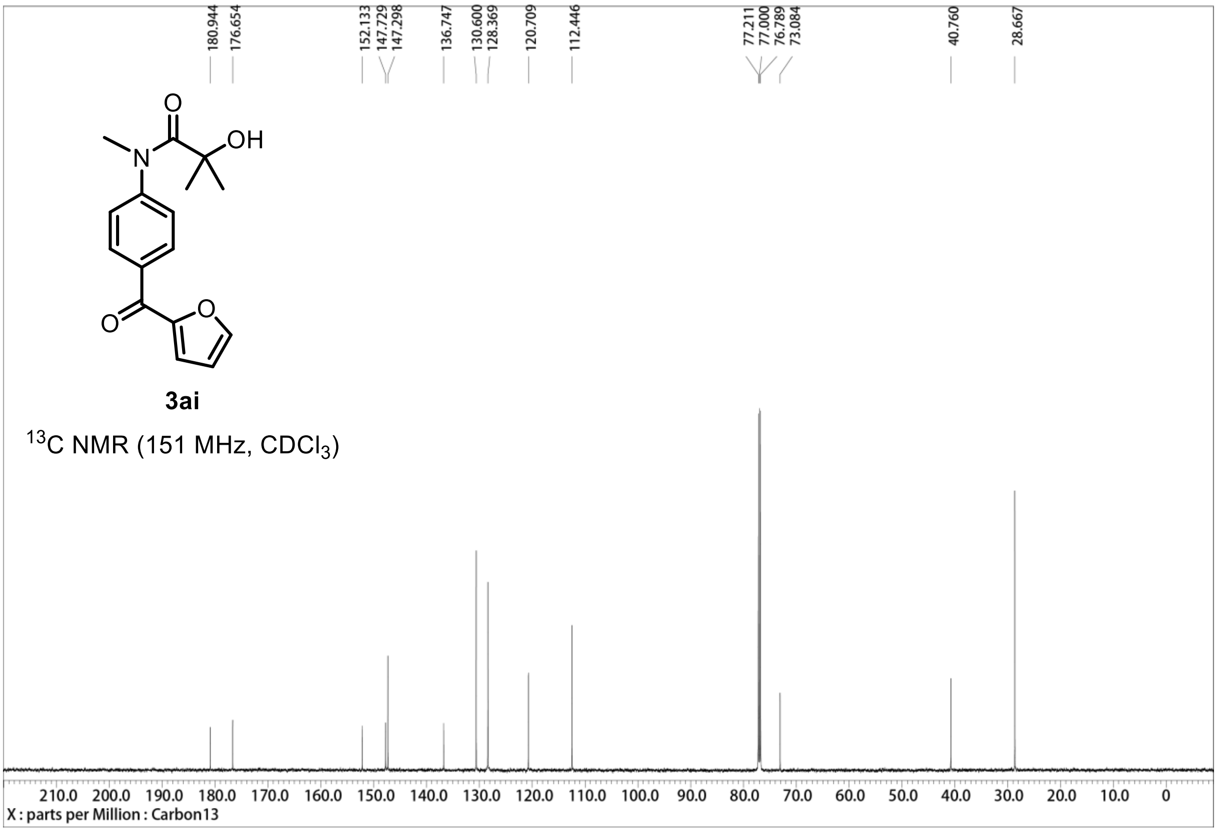

3ai

^{13}C NMR (151 MHz, $CDCl_3$)

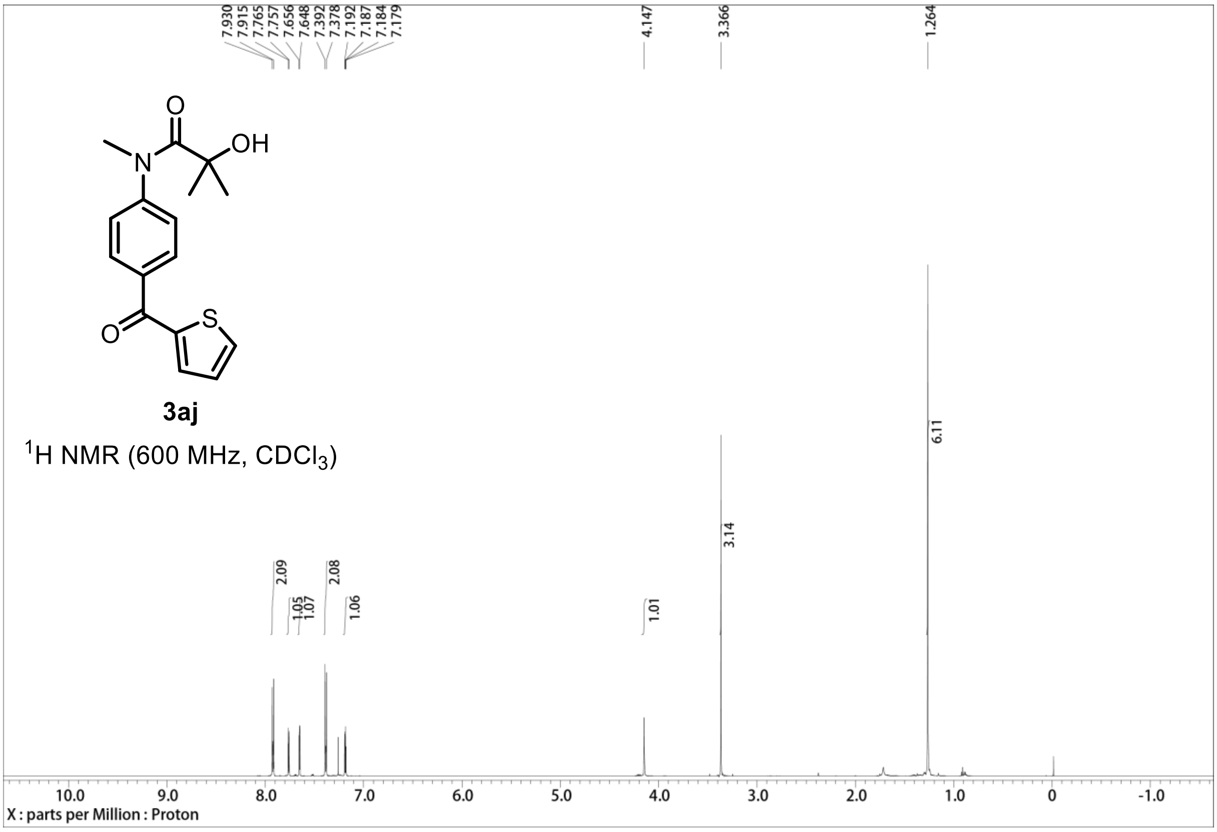
7.930 7.915 7.765 7.757 7.656 7.648 7.392 7.378 7.192 7.187 7.184 7.179
4.147
3.366
1.264
O
N
OH
O
S
3aj
^{1}H NMR (600 MHz, CDCl$_3$)
2.09 1.05 1.07 2.08 1.06
1.01
3.14
6.11
10.0 9.0 8.0 7.0 6.0 5.0 4.0 3.0 2.0 1.0 0 -1.0
X : parts per Million : Proton

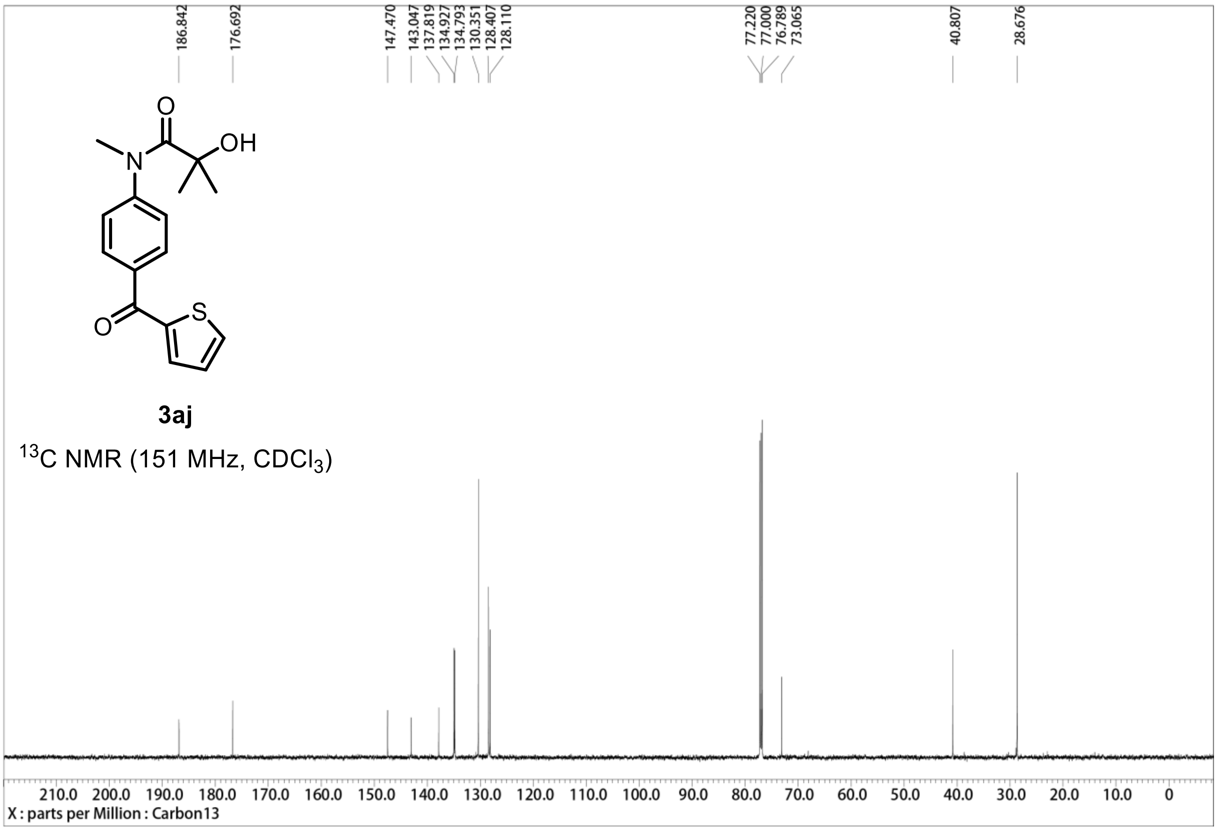
186.842
176.692
147.470 143.047 137.819 134.927 134.793 130.351 128.407 128.110
77.220 77.000 76.789 73.065
40.807
28.676
O
N
OH
O
S
3aj
^{13}C NMR (151 MHz, CDCl$_3$)
210.0 200.0 190.0 180.0 170.0 160.0 150.0 140.0 130.0 120.0 110.0 100.0 90.0 80.0 70.0 60.0 50.0 40.0 30.0 20.0 10.0 0
X : parts per Million : Carbon13

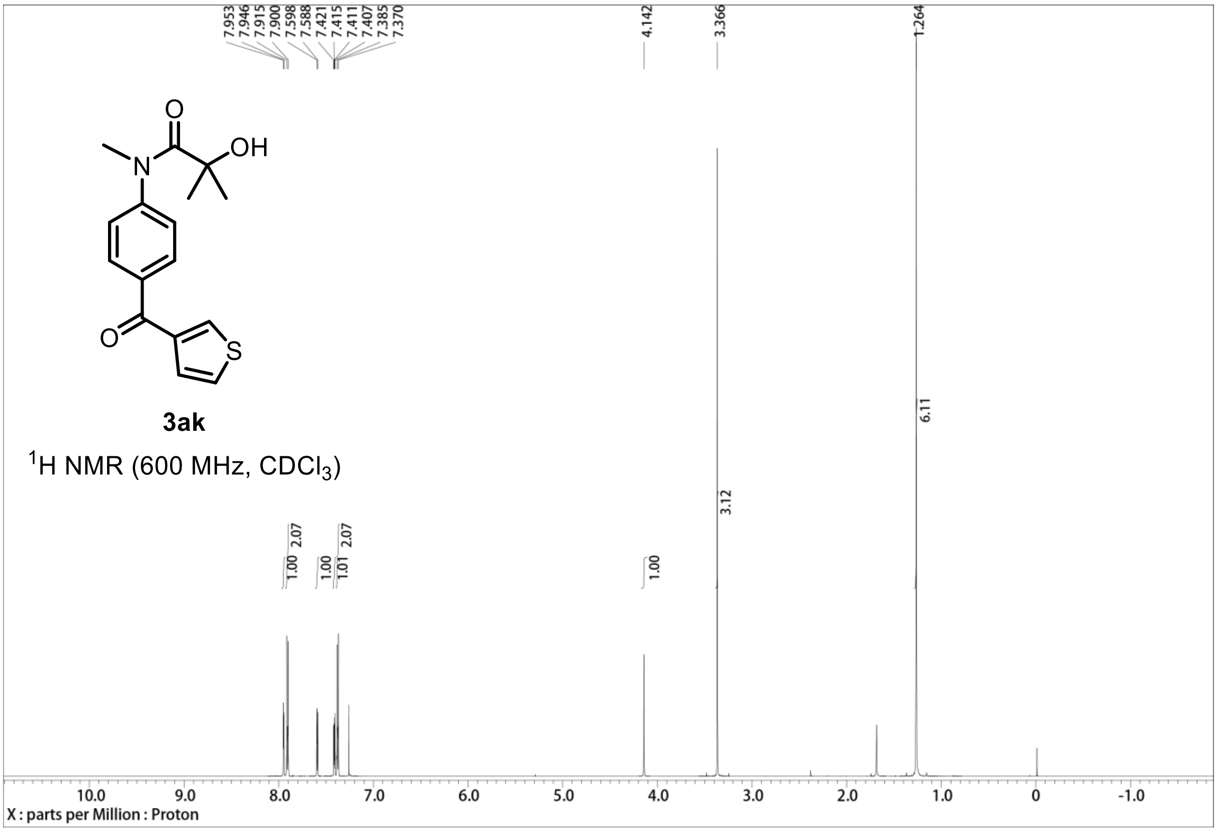
7.953
7.946
7.915
7.900
7.598
7.588
7.421
7.415
7.411
7.407
7.385
7.370
4.142
3.366
1.264
O
N
OH
O
S
3ak
1H NMR (600 MHz, CDCl3)
1.00
2.07
1.00
1.01
2.07
1.00
3.12
6.11
10.0 9.0 8.0 7.0 6.0 5.0 4.0 3.0 2.0 1.0 0 -1.0
X : parts per Million : Proton

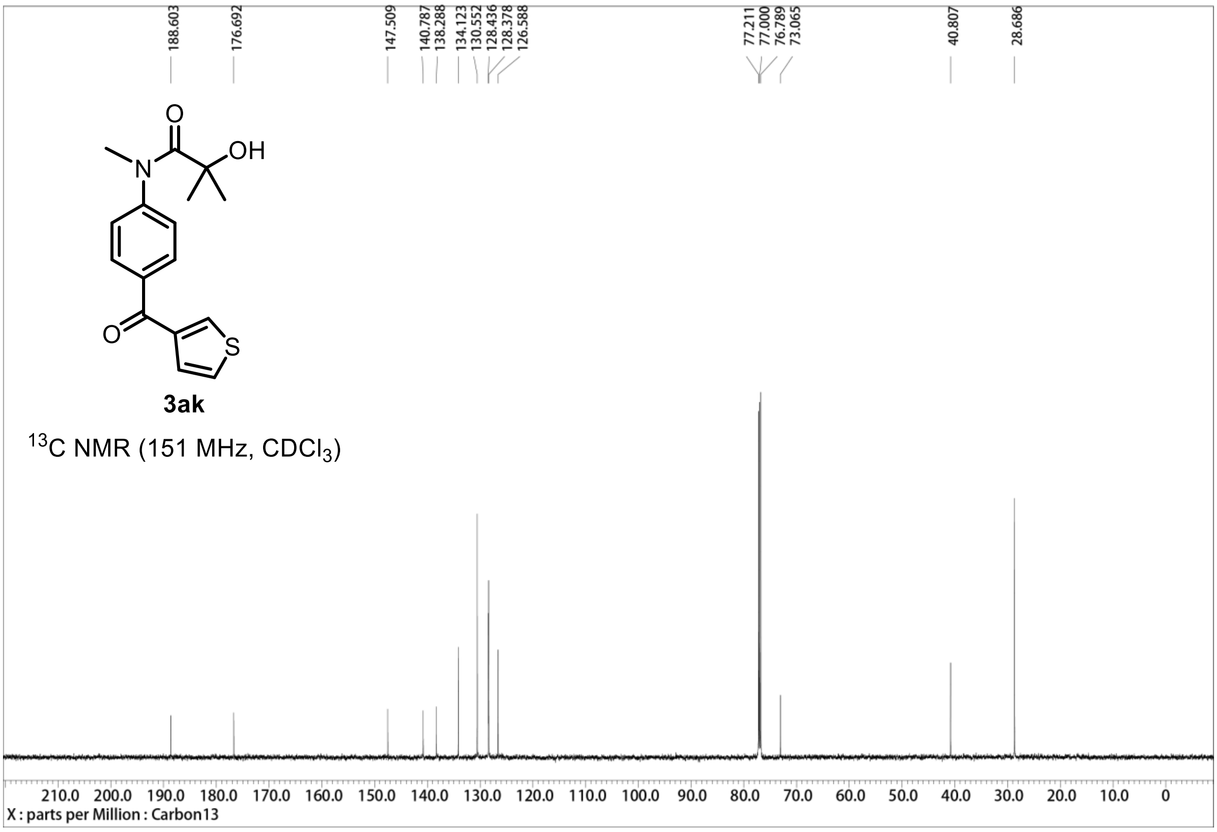
188.603
176.692
147.509
140.787
138.288
134.123
130.552
128.436
128.378
126.588
77.211
77.000
76.789
73.065
40.807
28.686
O
N
OH
O
S
3ak
13C NMR (151 MHz, CDCl3)
210.0 200.0 190.0 180.0 170.0 160.0 150.0 140.0 130.0 120.0 110.0 100.0 90.0 80.0 70.0 60.0 50.0 40.0 30.0 20.0 10.0 0
X : parts per Million : Carbon13

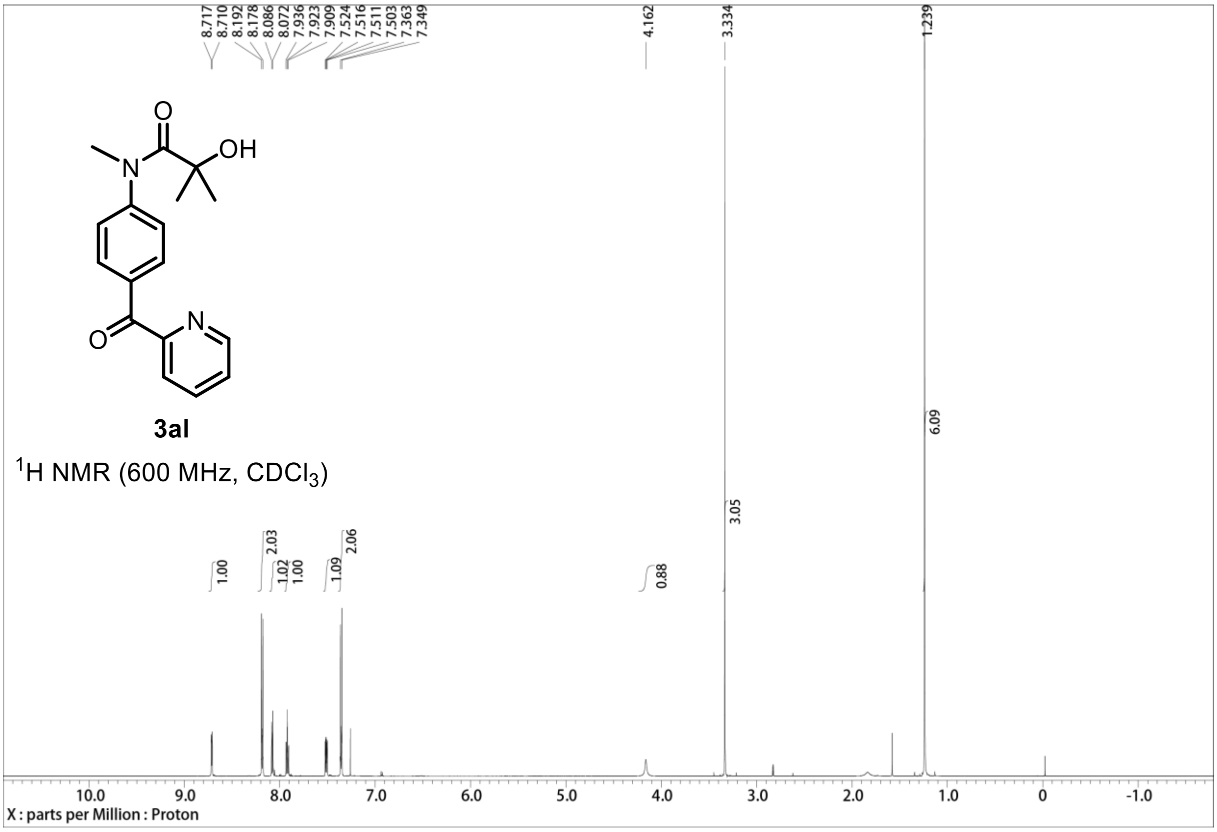
8.717
8.710
8.192
8.178
8.086
8.072
7.936
7.923
7.909
7.524
7.516
7.511
7.503
7.363
7.349
4.162
3.334
1.239
O
N
OH
O
N
3al
^{1}H NMR (600 MHz, $CDCl_3$)
1.00
2.03
1.02
1.00
1.09
2.06
0.88
3.05
6.09
10.0 9.0 8.0 7.0 6.0 5.0 4.0 3.0 2.0 1.0 0 -1.0
X : parts per Million : Proton

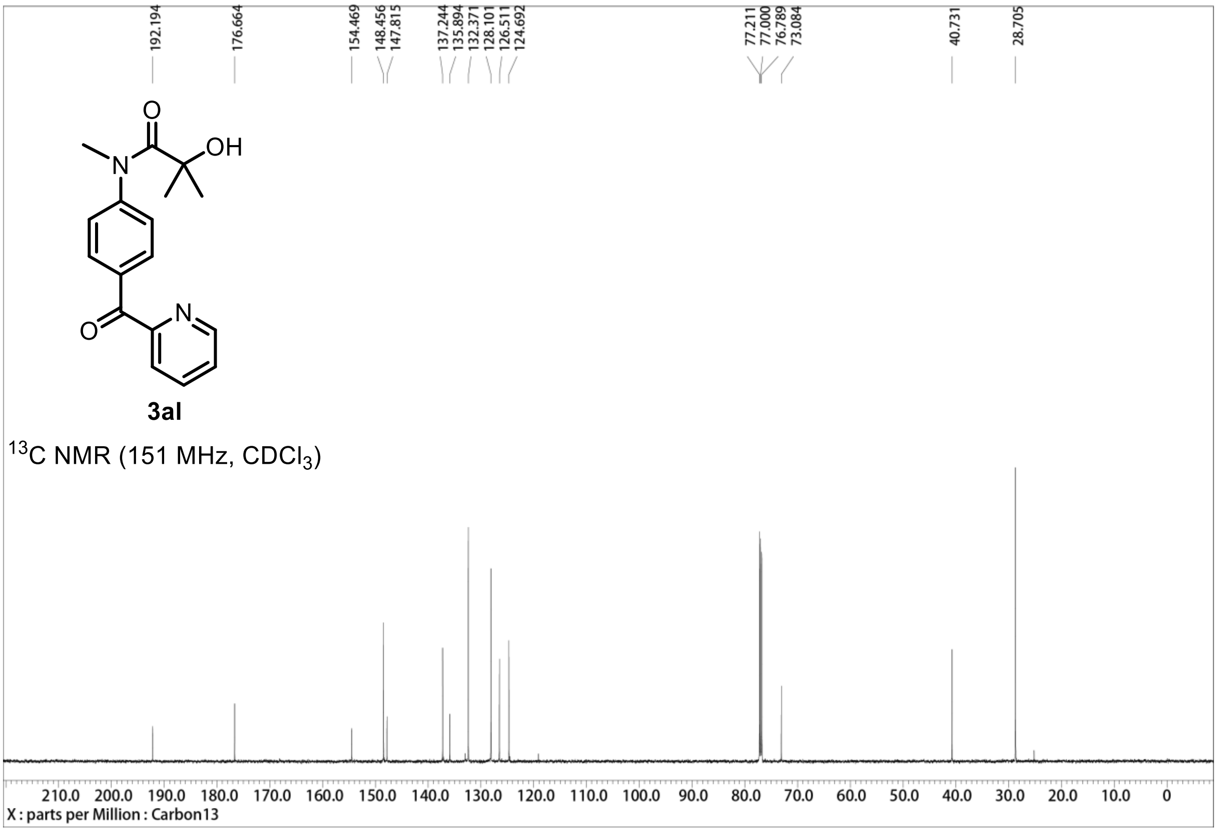
192.194
176.664
154.469
148.456
147.815
137.244
135.894
132.371
128.101
126.511
124.692
77.211
77.000
76.789
73.084
40.731
28.705
O
N
OH
O
N
3al
^{13}C NMR (151 MHz, $CDCl_3$)
210.0 200.0 190.0 180.0 170.0 160.0 150.0 140.0 130.0 120.0 110.0 100.0 90.0 80.0 70.0 60.0 50.0 40.0 30.0 20.0 10.0 0
X : parts per Million : Carbon13

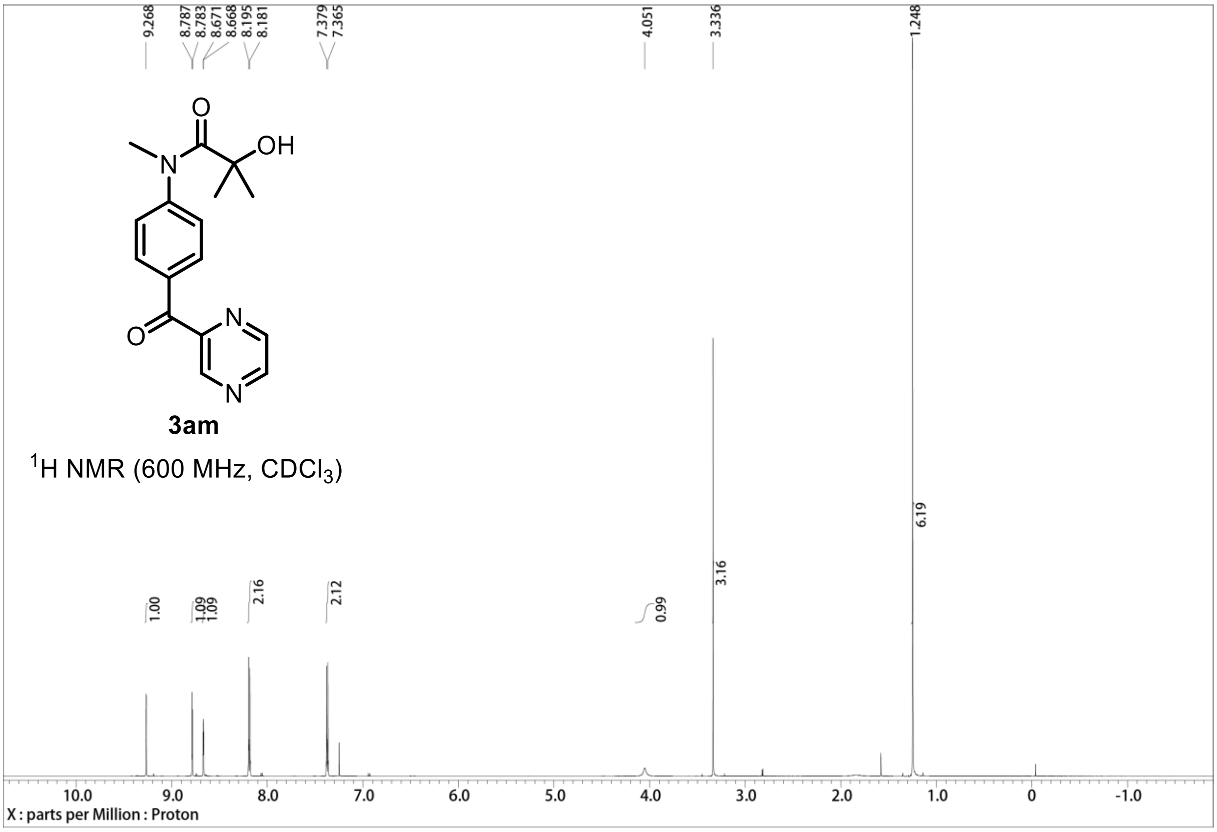

3am

^{1}H NMR (600 MHz, $CDCl_3$)

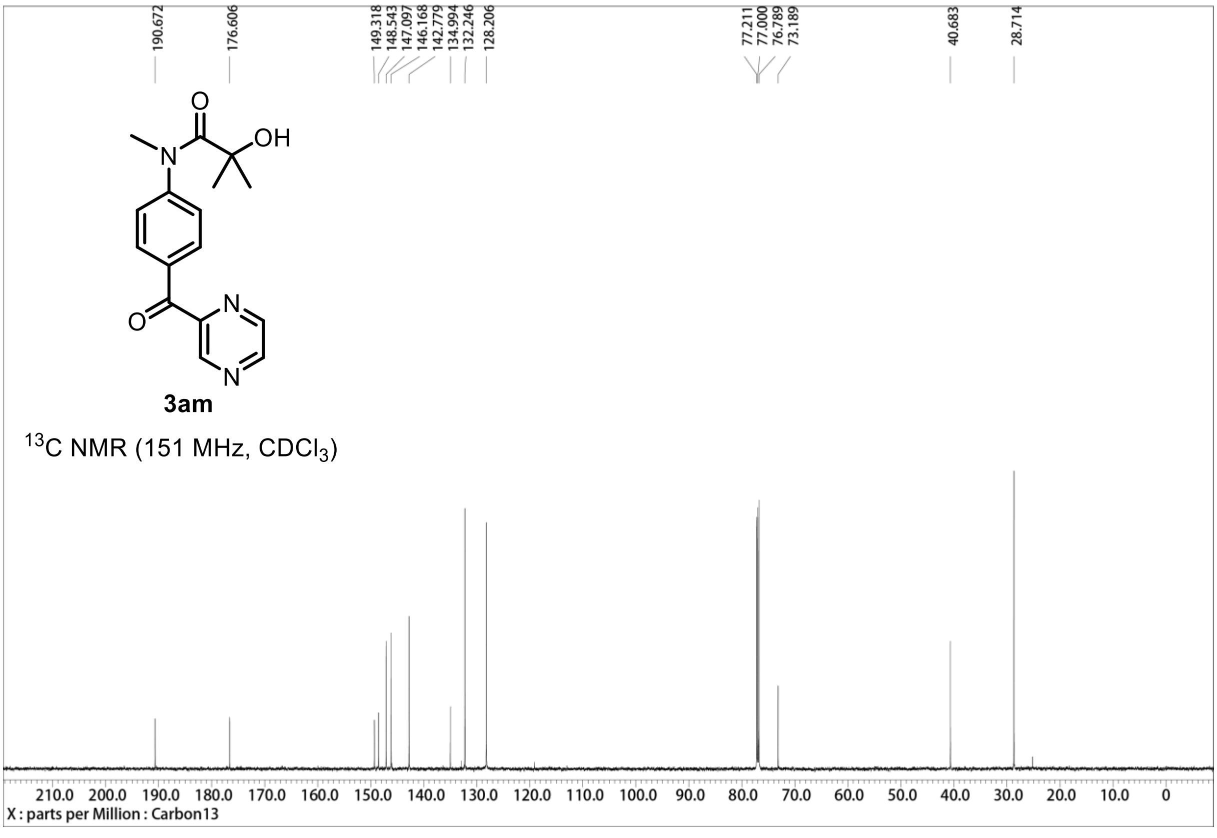

3am

^{13}C NMR (151 MHz, $CDCl_3$)

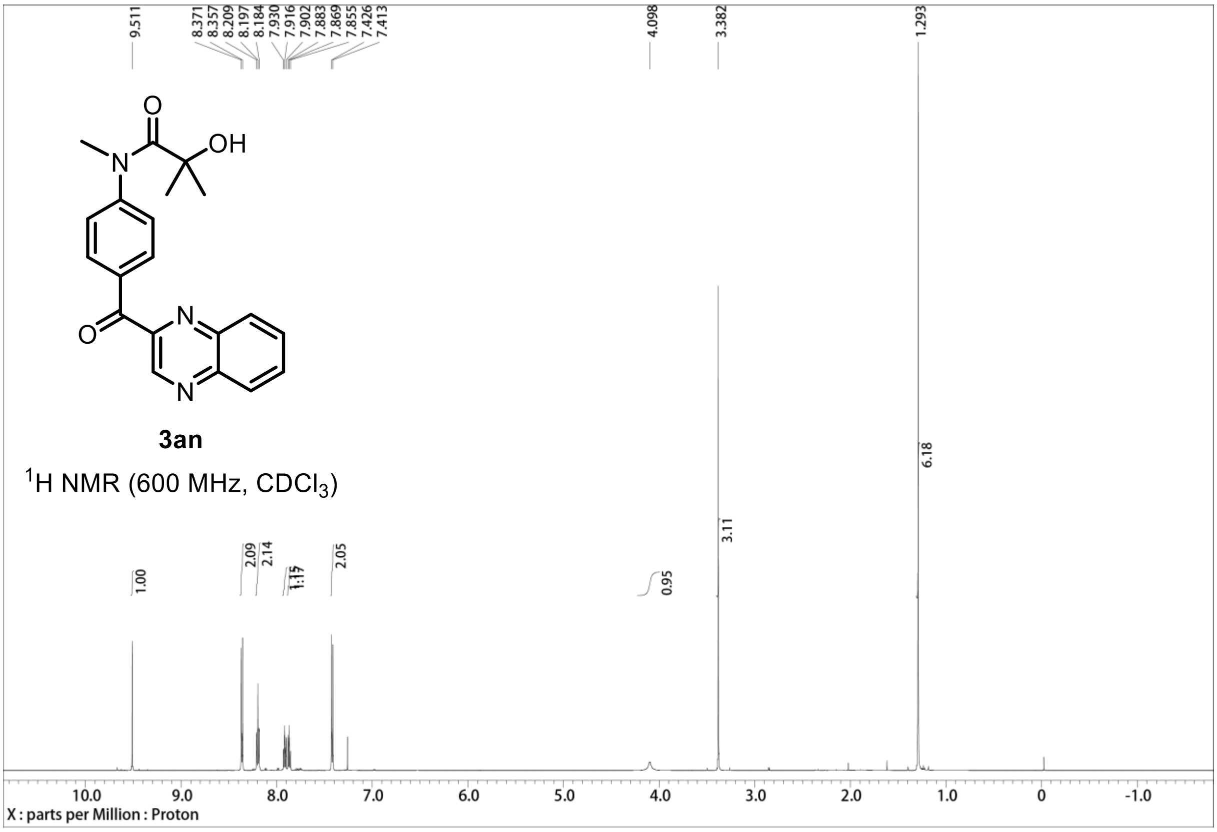
9.511
8.371
8.357
8.209
8.197
8.184
7.930
7.916
7.902
7.883
7.869
7.855
7.426
7.413
4.098
3.382
1.293
O
N
OH
O
N
N
3an
¹H NMR (600 MHz, CDCl3)
1.00
2.09
2.14
2.05
0.95
3.11
6.18
10.0
9.0
8.0
7.0
6.0
5.0
4.0
3.0
2.0
1.0
0
-1.0
X : parts per Million : Proton

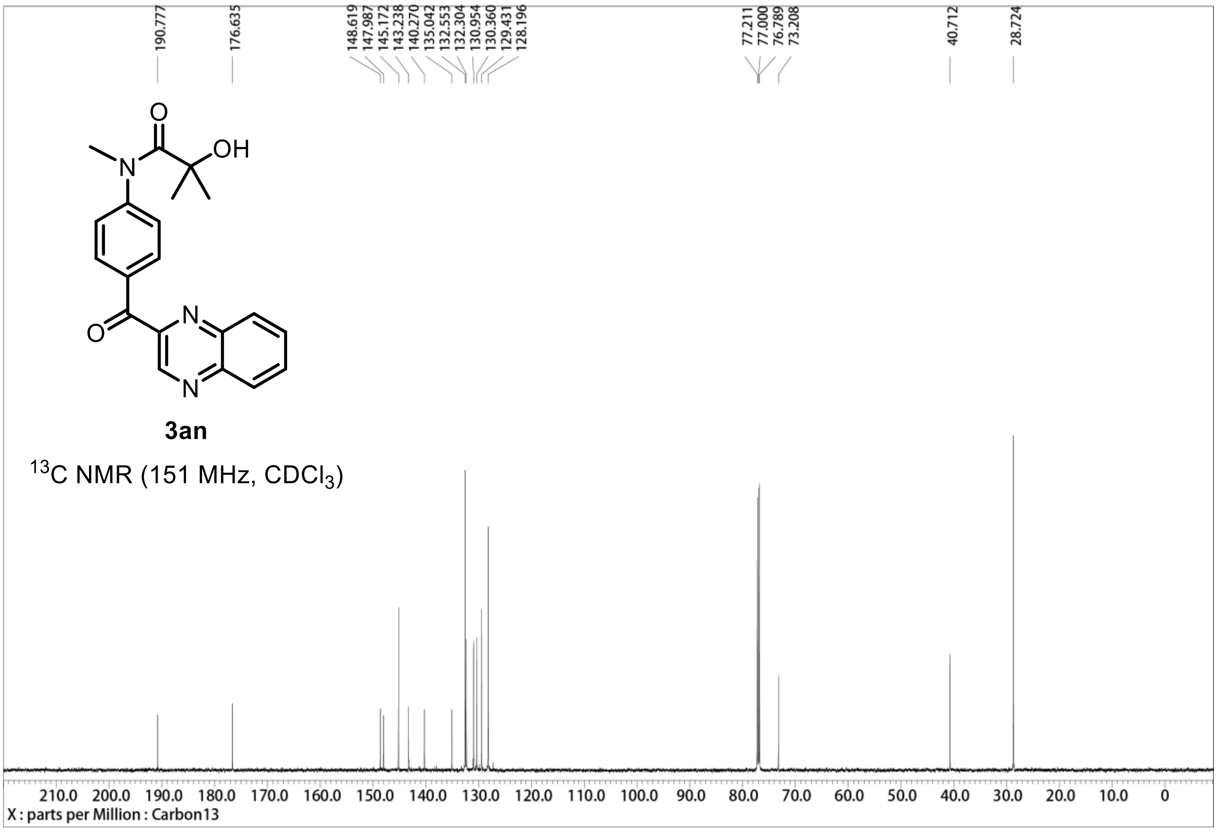
190.777
176.635
148.619
147.987
145.172
143.238
140.270
135.042
132.553
132.304
130.954
130.360
129.431
128.196
77.211
77.000
76.789
73.208
40.712
28.724
O
N
OH
O
N
N
3an
¹³C NMR (151 MHz, CDCl3)
210.0
200.0
190.0
180.0
170.0
160.0
150.0
140.0
130.0
120.0
110.0
100.0
90.0
80.0
70.0
60.0
50.0
40.0
30.0
20.0
10.0
0
X : parts per Million : Carbon13

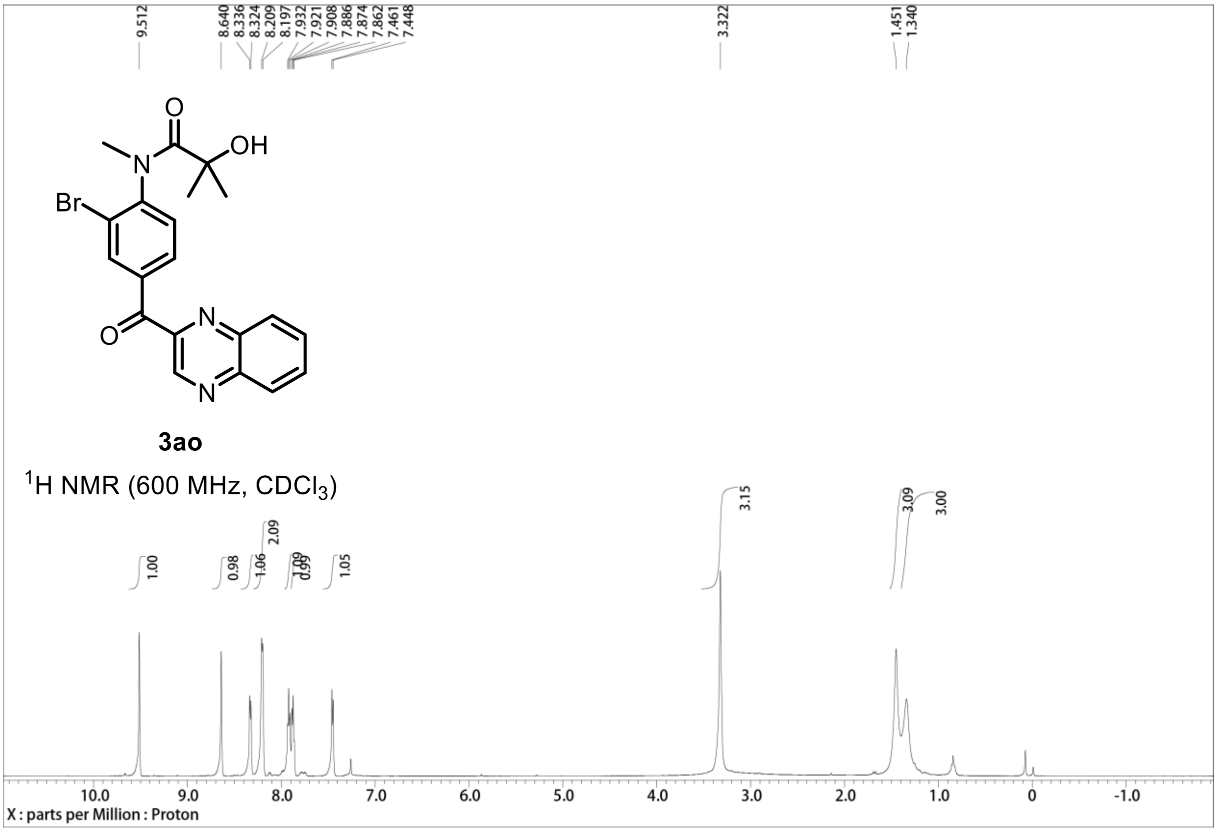
9.512
8.640
8.336
8.324
8.209
8.197
7.932
7.921
7.908
7.886
7.874
7.862
7.461
7.448
3.322
1.451
1.340
3ao
^{1}H NMR (600 MHz, $CDCl_3$)
X : parts per Million : Proton

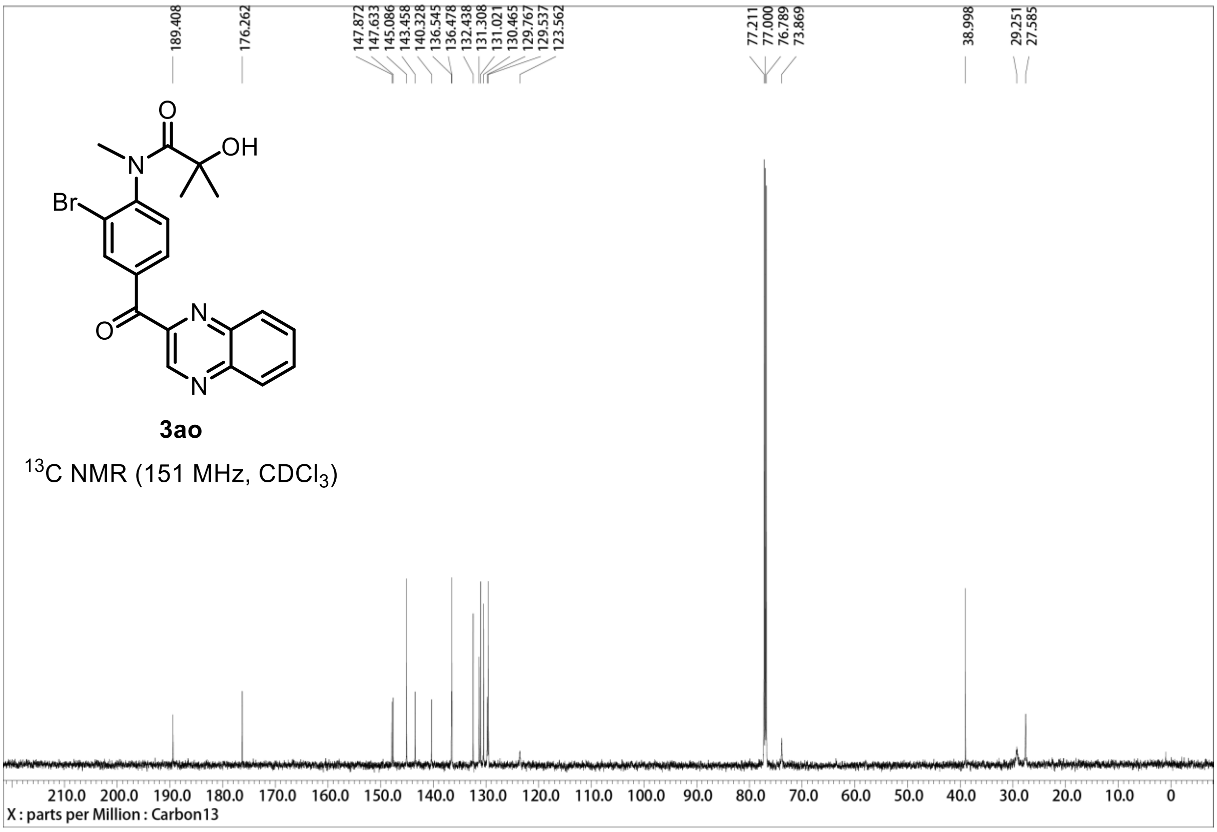
189.408
176.262
147.872
147.633
145.086
143.458
140.328
136.545
136.478
132.438
131.308
131.021
130.465
129.767
129.537
123.562
77.211
77.000
76.789
73.869
38.998
29.251
27.585
3ao
^{13}C NMR (151 MHz, $CDCl_3$)
X : parts per Million : Carbon13

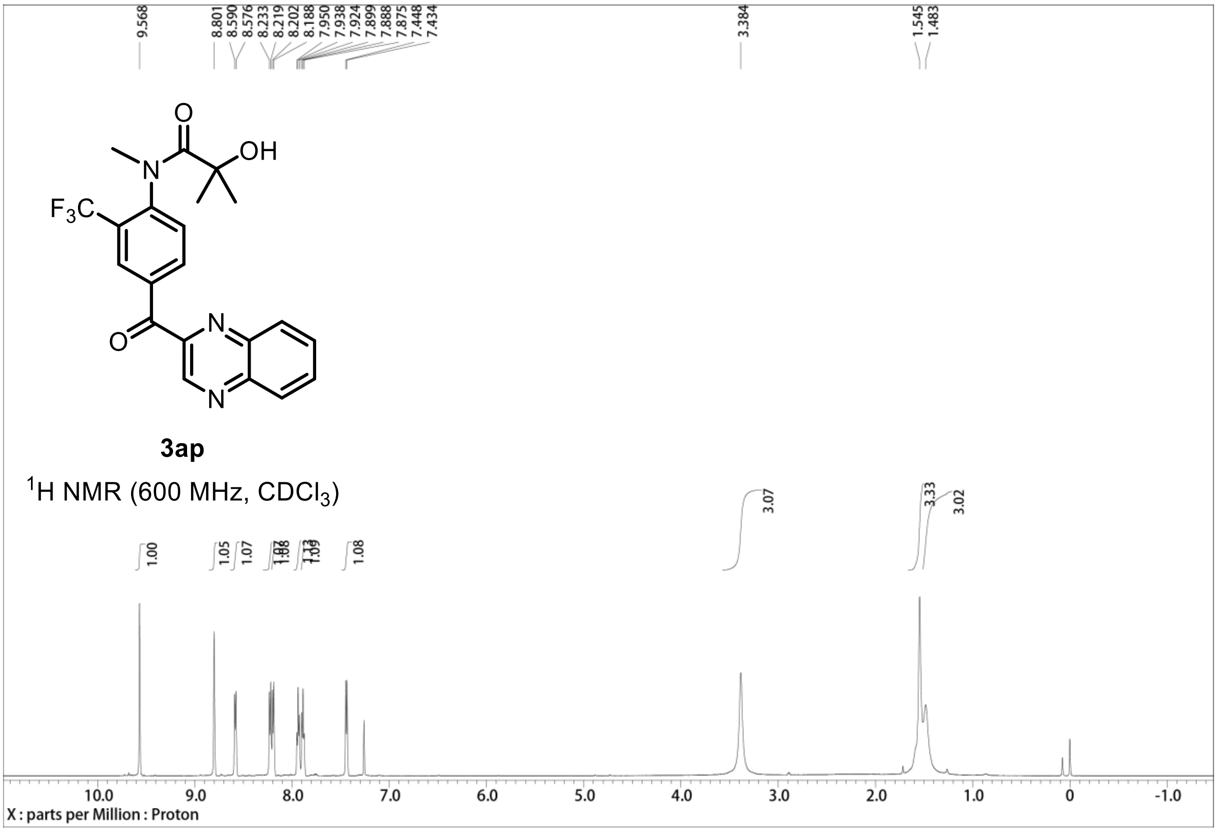
9.568
8.801
8.590
8.576
8.233
8.219
8.202
8.188
7.950
7.938
7.924
7.899
7.888
7.875
7.448
7.434
3.384
1.545
1.483
3ap
^{1}H NMR (600 MHz, $CDCl_3$)
1.00
1.05
1.07
1.08
3.07
3.33
3.02
X : parts per Million : Proton

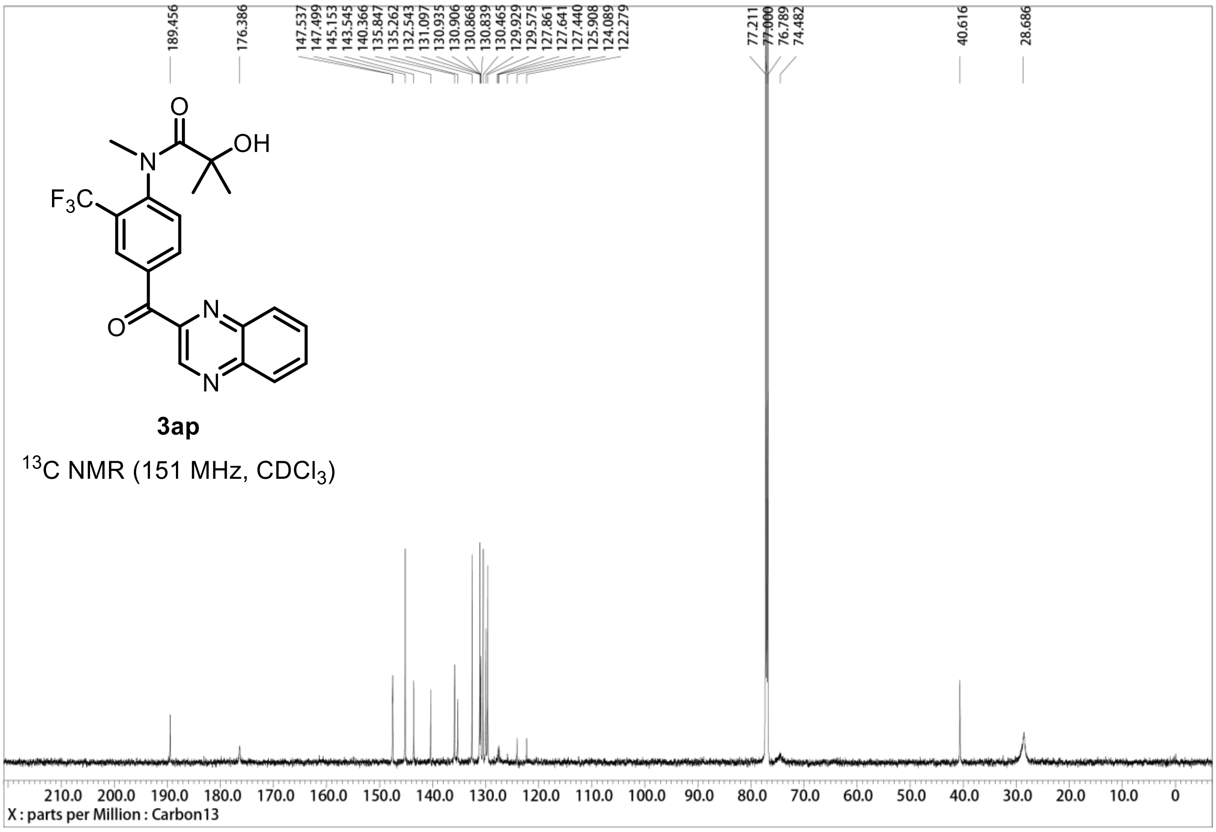
189.456
176.386
147.537
147.499
145.153
143.545
140.366
135.847
135.262
132.543
131.097
130.935
130.906
130.868
130.839
130.465
129.929
129.575
127.861
127.641
127.440
125.908
124.089
122.279
77.211
77.000
76.789
74.482
40.616
28.686
3ap
^{13}C NMR (151 MHz, $CDCl_3$)
X : parts per Million : Carbon13

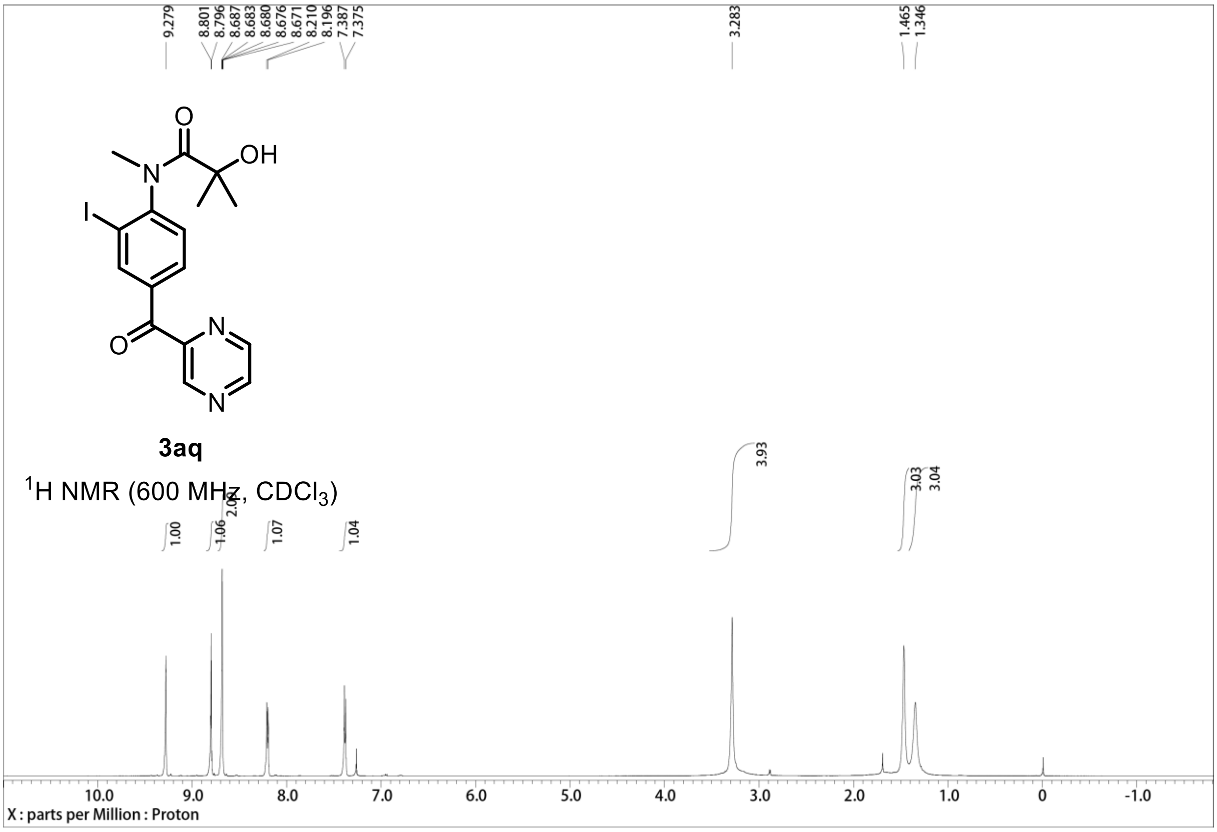
9.279
8.801
8.796
8.687
8.683
8.680
8.676
8.671
8.210
8.196
7.387
7.375
3.283
1.465
1.346
O
N
OH
I
O
N
N
3aq
^{1}H NMR (600 MHz, $CDCl_3$)
1.00
1.06
2.09
1.07
1.04
3.93
3.03
3.04
10.0
9.0
8.0
7.0
6.0
5.0
4.0
3.0
2.0
1.0
0
-1.0
X : parts per Million : Proton

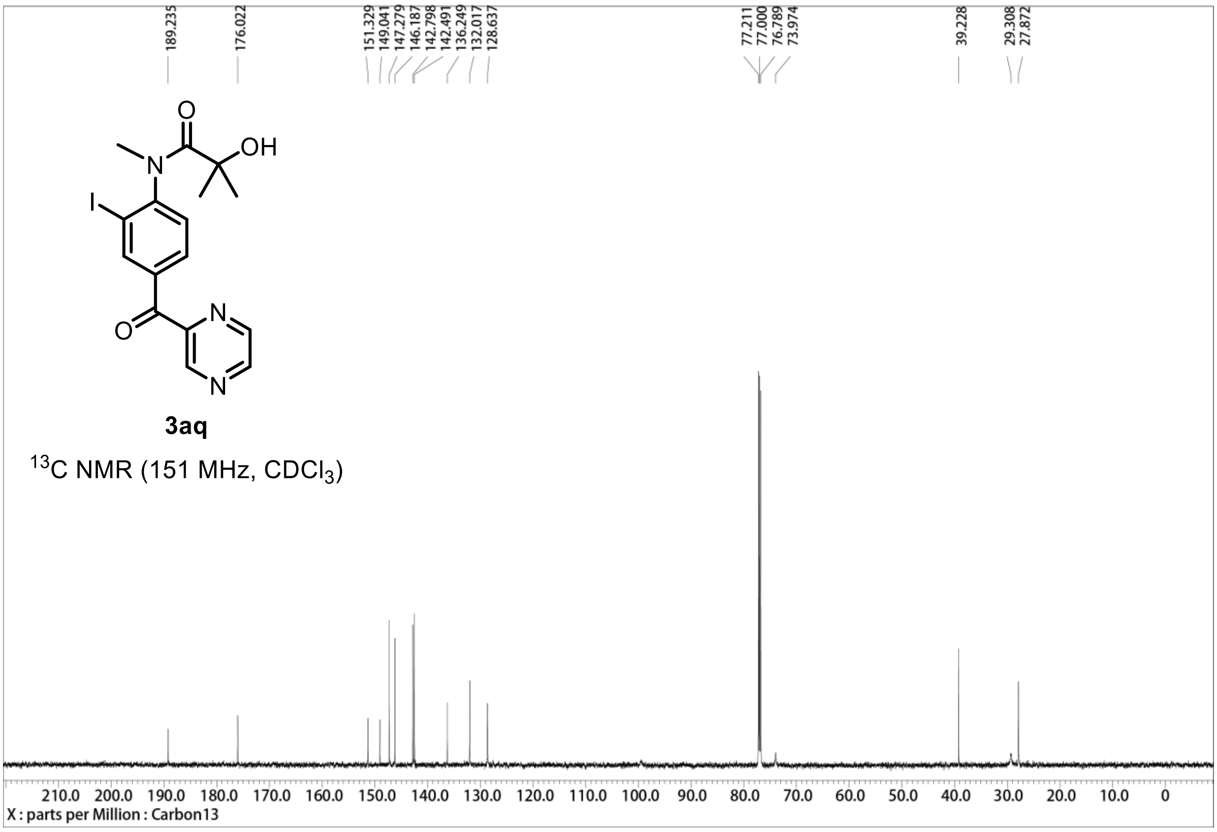
189.235
176.022
151.329
149.041
147.279
146.187
142.798
142.491
136.249
132.017
128.637
77.211
77.000
76.789
73.974
39.228
29.308
27.872
O
N
OH
I
O
N
N
3aq
^{13}C NMR (151 MHz, $CDCl_3$)
210.0
200.0
190.0
180.0
170.0
160.0
150.0
140.0
130.0
120.0
110.0
100.0
90.0
80.0
70.0
60.0
50.0
40.0
30.0
20.0
10.0
0
X : parts per Million : Carbon13

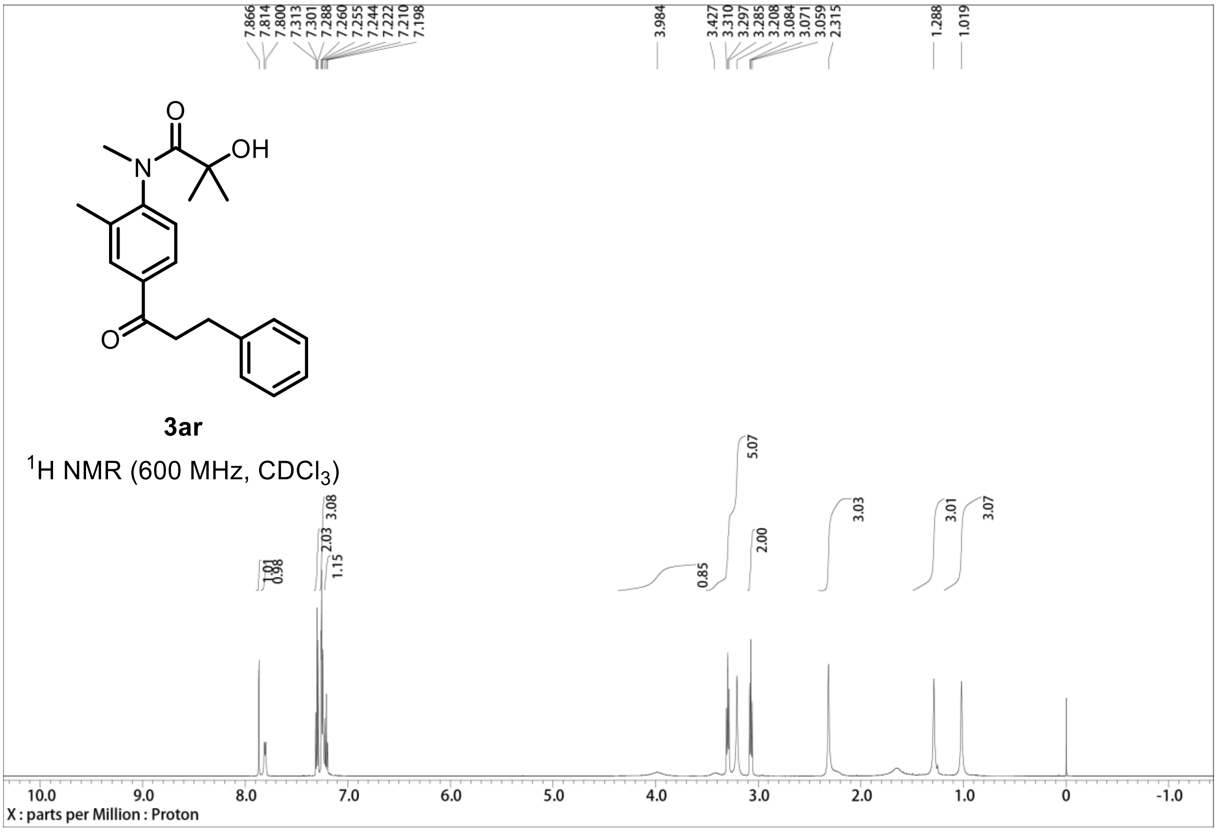
7.866
7.814
7.800
7.313
7.301
7.288
7.260
7.255
7.244
7.222
7.210
7.198
3.984
3.427
3.310
3.297
3.285
3.208
3.084
3.071
3.059
2.315
1.288
1.019
3ar
^{1}H NMR (600 MHz, $CDCl_3$)
1.01
0.98
2.03
3.08
1.15
0.85
5.07
2.00
3.03
3.01
3.07
10.0 9.0 8.0 7.0 6.0 5.0 4.0 3.0 2.0 1.0 0 -1.0
X : parts per Million : Proton

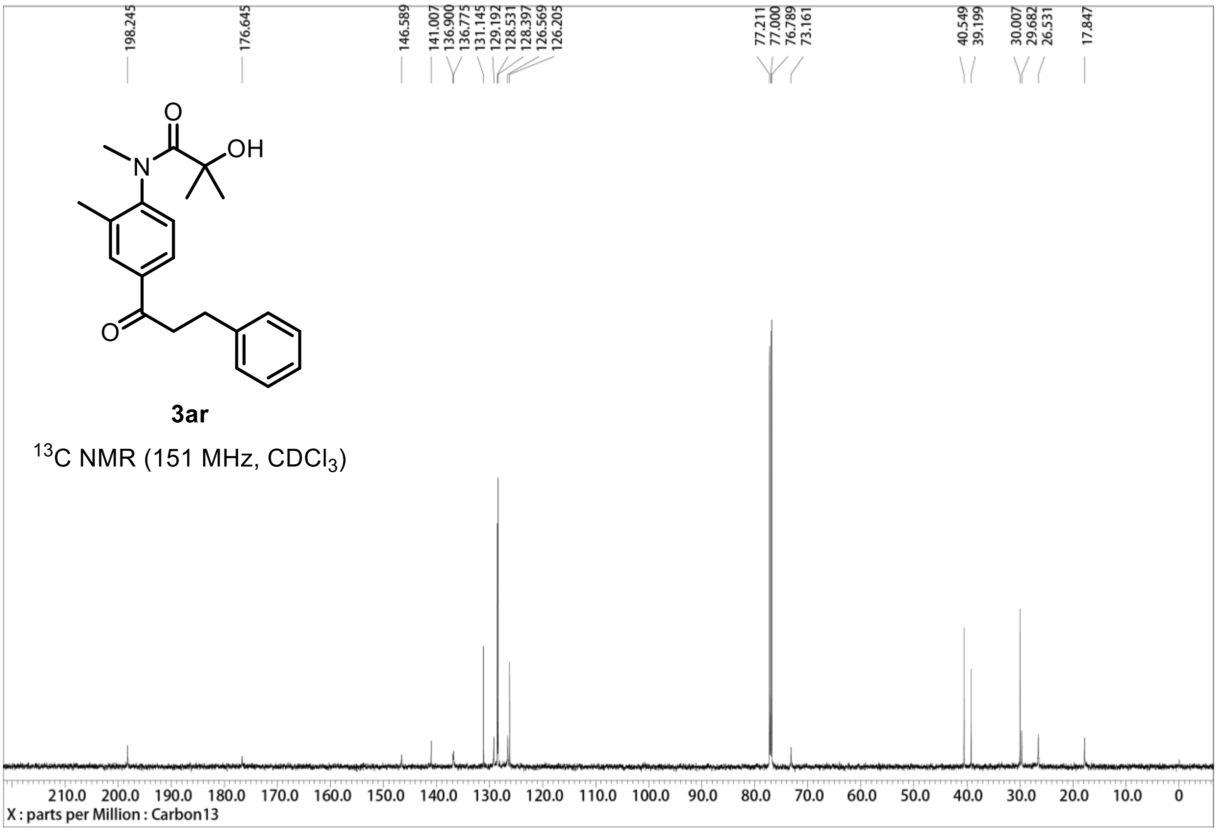
198.245
176.645
146.589
141.007
136.900
136.775
131.145
129.192
128.531
128.397
126.569
126.205
77.211
77.000
76.789
73.161
40.549
39.199
30.007
29.682
26.531
17.847
3ar
^{13}C NMR (151 MHz, $CDCl_3$)
210.0 200.0 190.0 180.0 170.0 160.0 150.0 140.0 130.0 120.0 110.0 100.0 90.0 80.0 70.0 60.0 50.0 40.0 30.0 20.0 10.0 0
X : parts per Million : Carbon13

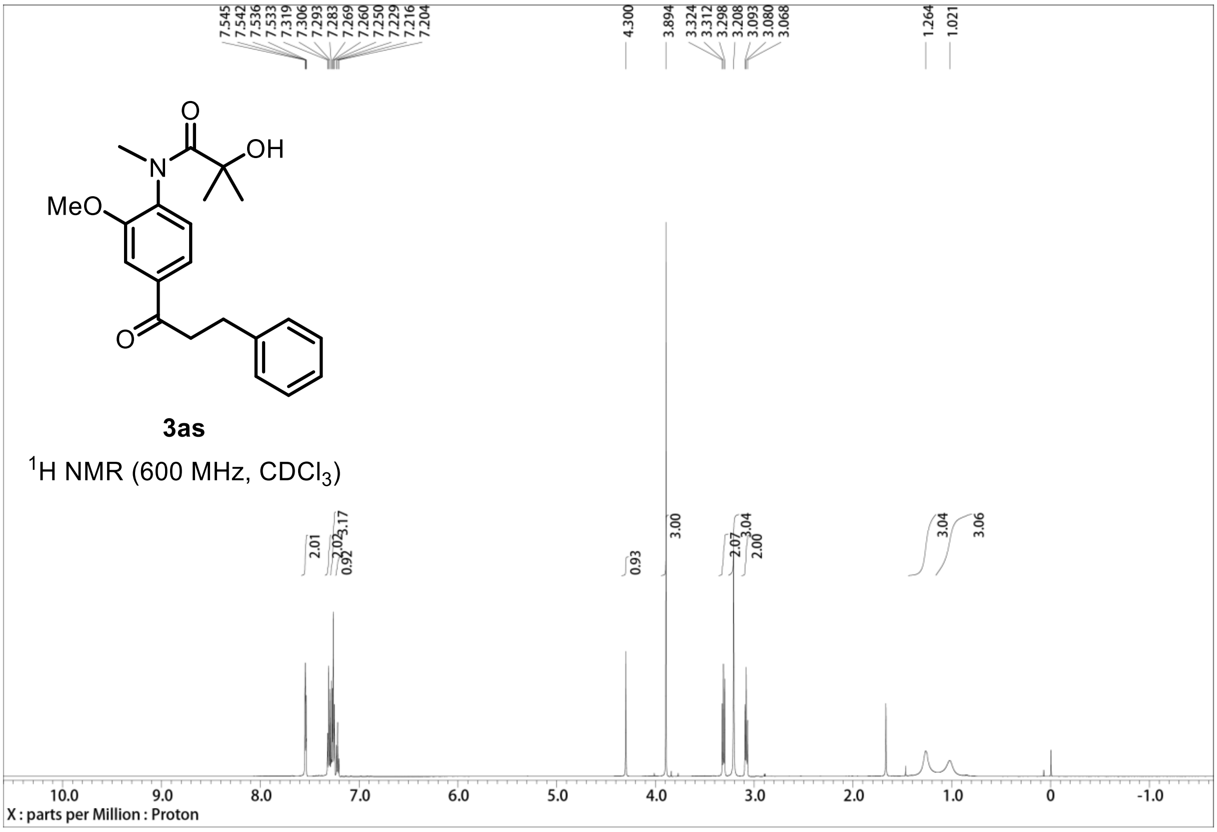
7.545 7.542 7.536 7.533 7.319 7.306 7.293 7.283 7.269 7.260 7.250 7.229 7.216 7.204
4.300
3.894 3.324 3.312 3.298 3.208 3.093 3.080 3.068
1.264 1.021
O
N
OH
MeO
O
3as
¹H NMR (600 MHz, CDCl₃)
2.01 2.02 0.92 3.17 0.93 3.00 2.07 3.04 2.00 3.04 3.06
10.0 9.0 8.0 7.0 6.0 5.0 4.0 3.0 2.0 1.0 0 -1.0
X : parts per Million : Proton

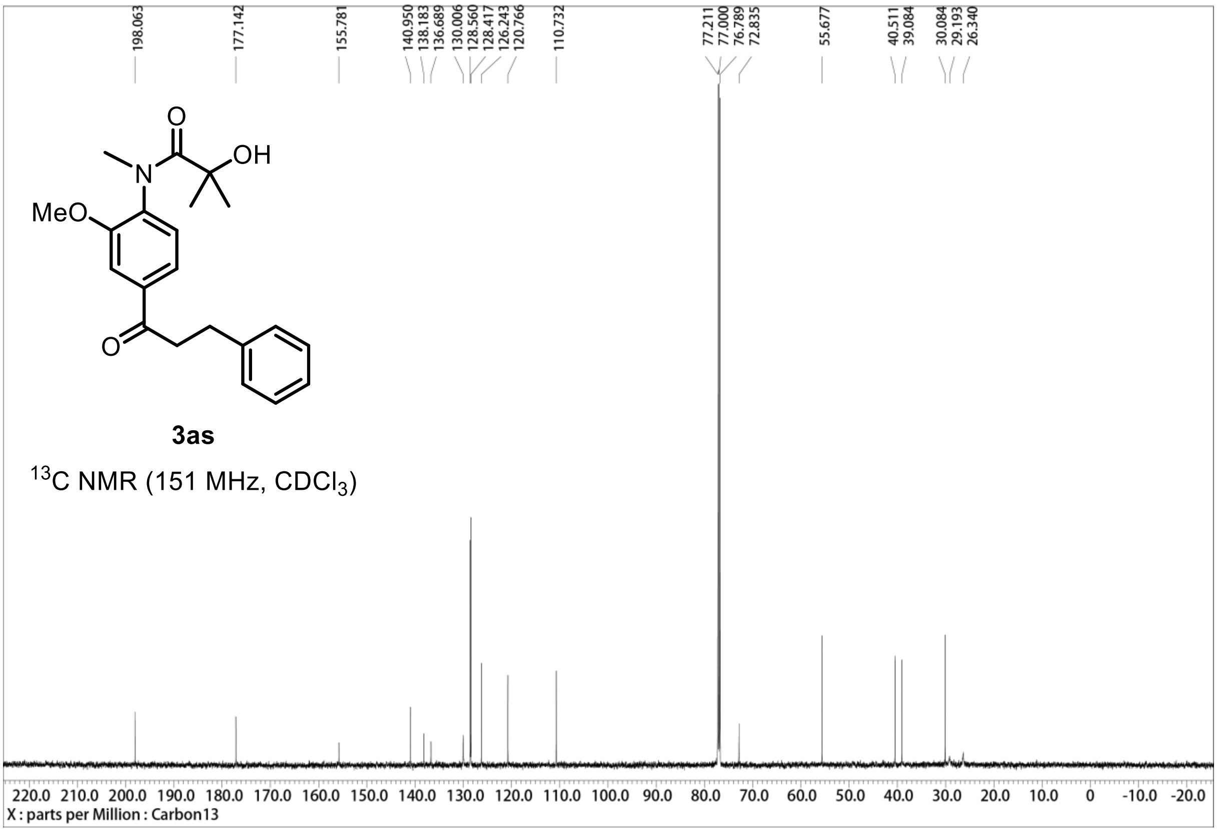
198.063
177.142
155.781
140.950 138.183 136.689 130.006 128.560 128.417 126.243 120.766
110.732
77.211 77.000 76.789 72.835
55.677
40.511 39.084
30.084 29.193 26.340
O
N
OH
MeO
O
3as
¹³C NMR (151 MHz, CDCl₃)
220.0 210.0 200.0 190.0 180.0 170.0 160.0 150.0 140.0 130.0 120.0 110.0 100.0 90.0 80.0 70.0 60.0 50.0 40.0 30.0 20.0 10.0 0 -10.0 -20.0
X : parts per Million : Carbon13

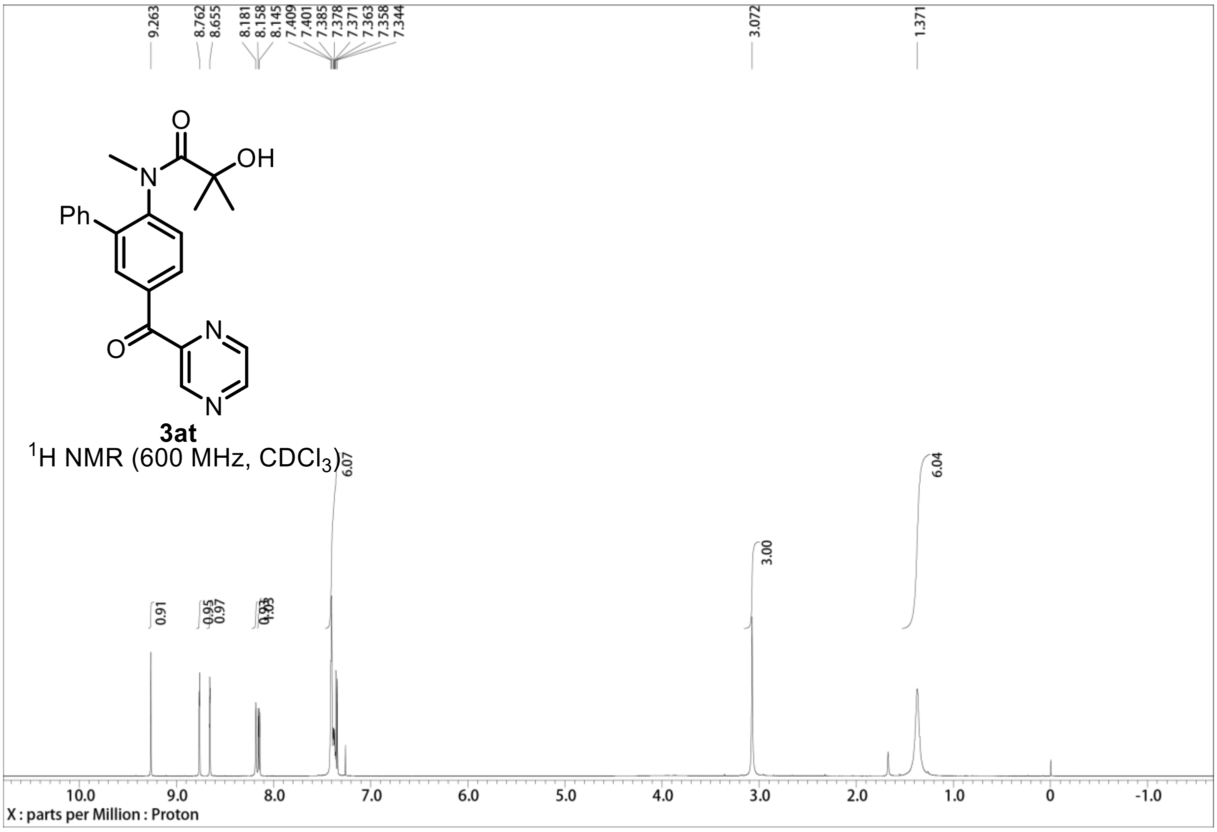
9.263
8.762
8.655
8.181
8.158
8.145
7.409
7.401
7.385
7.378
7.371
7.363
7.358
7.344
3.072
1.371
Ph
N
O
OH
N
N
O
3at
1H NMR (600 MHz, CDCl3)
0.91
0.95
0.97
0.93
1.03
6.07
3.00
6.04
10.0
9.0
8.0
7.0
6.0
5.0
4.0
3.0
2.0
1.0
0
-1.0
X : parts per Million : Proton

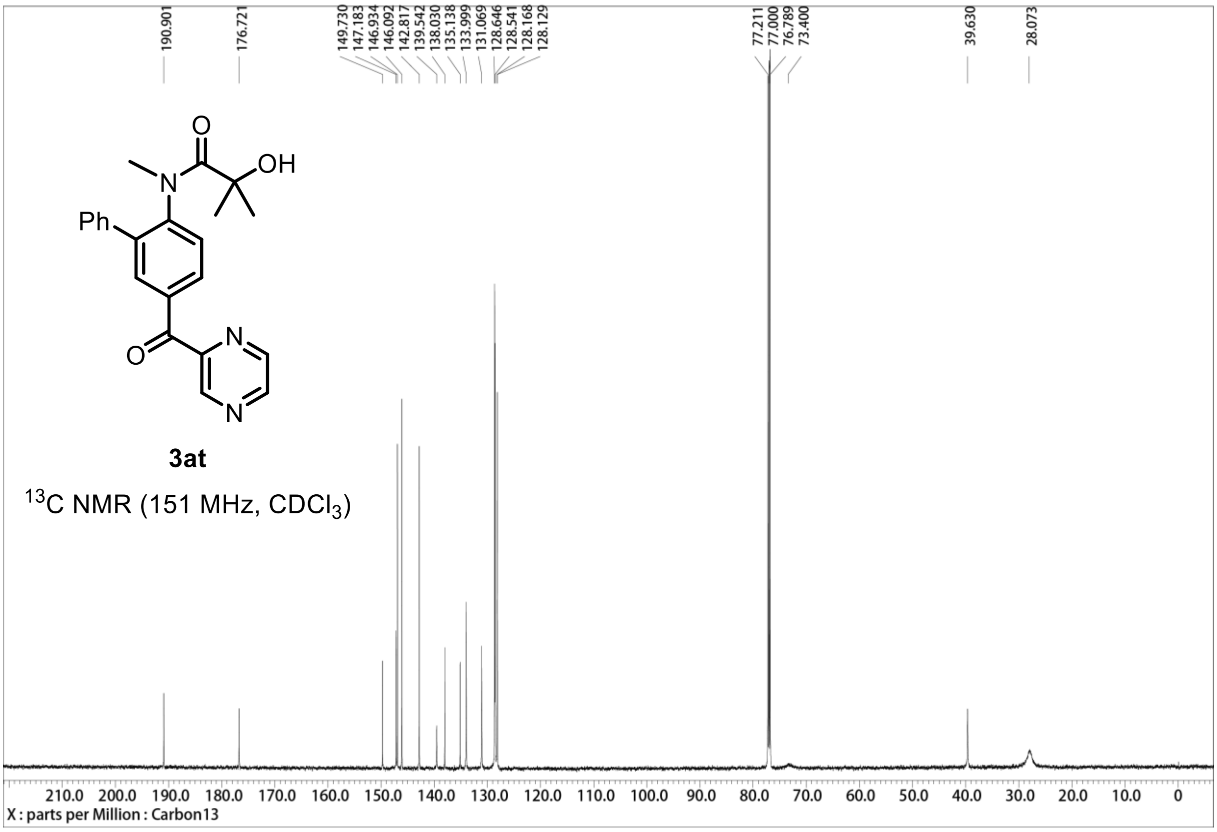
190.901
176.721
149.730
147.183
146.934
146.092
142.817
139.542
138.030
135.138
133.999
131.069
128.646
128.541
128.168
128.129
77.211
77.000
76.789
73.400
39.630
28.073
Ph
N
O
OH
N
N
O
3at
13C NMR (151 MHz, CDCl3)
210.0
200.0
190.0
180.0
170.0
160.0
150.0
140.0
130.0
120.0
110.0
100.0
90.0
80.0
70.0
60.0
50.0
40.0
30.0
20.0
10.0
0
X : parts per Million : Carbon13

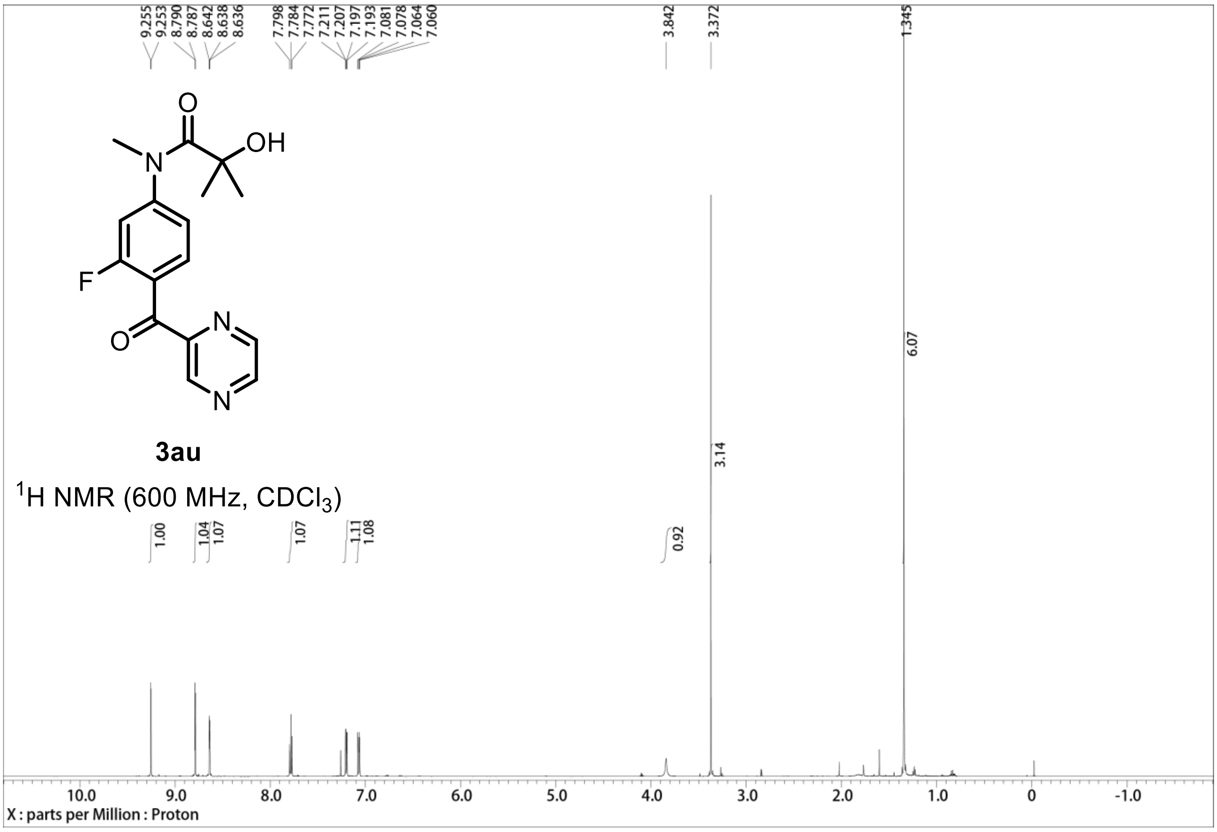
9.255 9.253 8.790 8.787 8.642 8.638 8.636 7.798 7.784 7.772 7.211 7.207 7.197 7.193 7.081 7.078 7.064 7.060 3.842 3.372 1.345
OH
N
O
F
N
N
3au
1H NMR (600 MHz, CDCl3)
1.00 1.04 1.07 1.07 1.11 1.08 0.92 3.14 6.07
10.0 9.0 8.0 7.0 6.0 5.0 4.0 3.0 2.0 1.0 0 -1.0
X : parts per Million : Proton

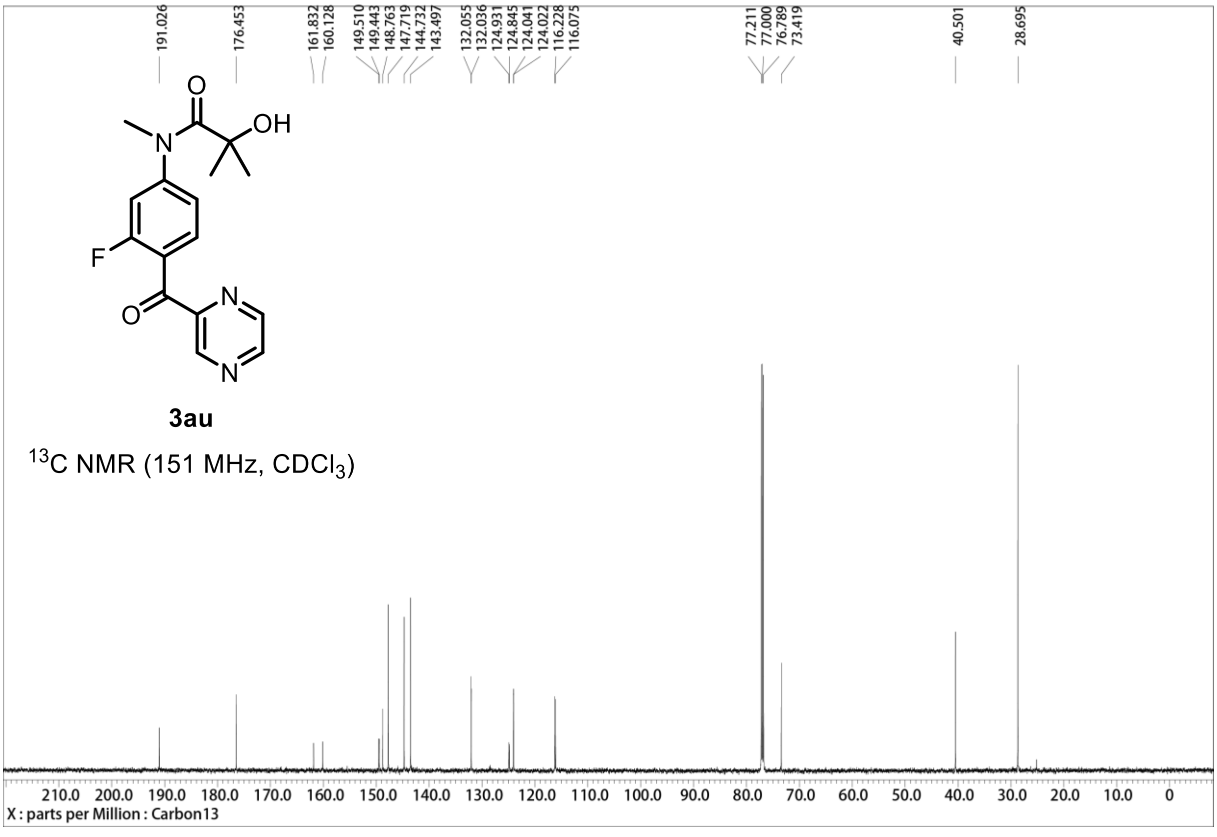
191.026 176.453 161.832 160.128 149.510 149.443 148.763 147.719 144.732 143.497 132.055 132.036 124.931 124.845 124.041 124.022 116.228 116.075 77.211 77.000 76.789 73.419 40.501 28.695
OH
N
O
F
N
N
3au
13C NMR (151 MHz, CDCl3)
210.0 200.0 190.0 180.0 170.0 160.0 150.0 140.0 130.0 120.0 110.0 100.0 90.0 80.0 70.0 60.0 50.0 40.0 30.0 20.0 10.0 0
X : parts per Million : Carbon13

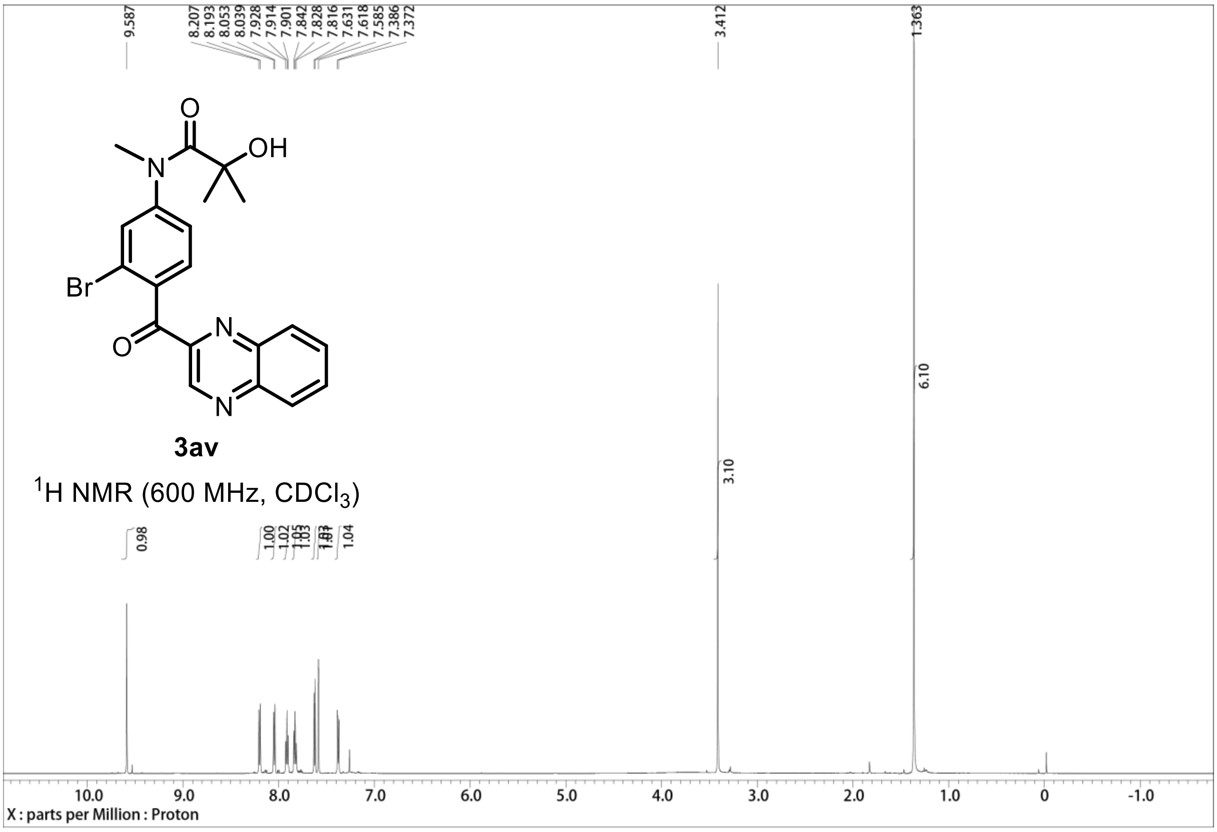

9.587
8.207
8.193
8.053
8.039
7.928
7.914
7.901
7.842
7.828
7.816
7.631
7.618
7.585
7.386
7.372
3.412
1.363
O
N
OH
Br
O
N
N
3av
^{1}H NMR (600 MHz, $CDCl_3$)
0.98
1.00
1.02
1.05
1.03
1.04
3.10
6.10
10.0
9.0
8.0
7.0
6.0
5.0
4.0
3.0
2.0
1.0
0
-1.0
X : parts per Million : Proton

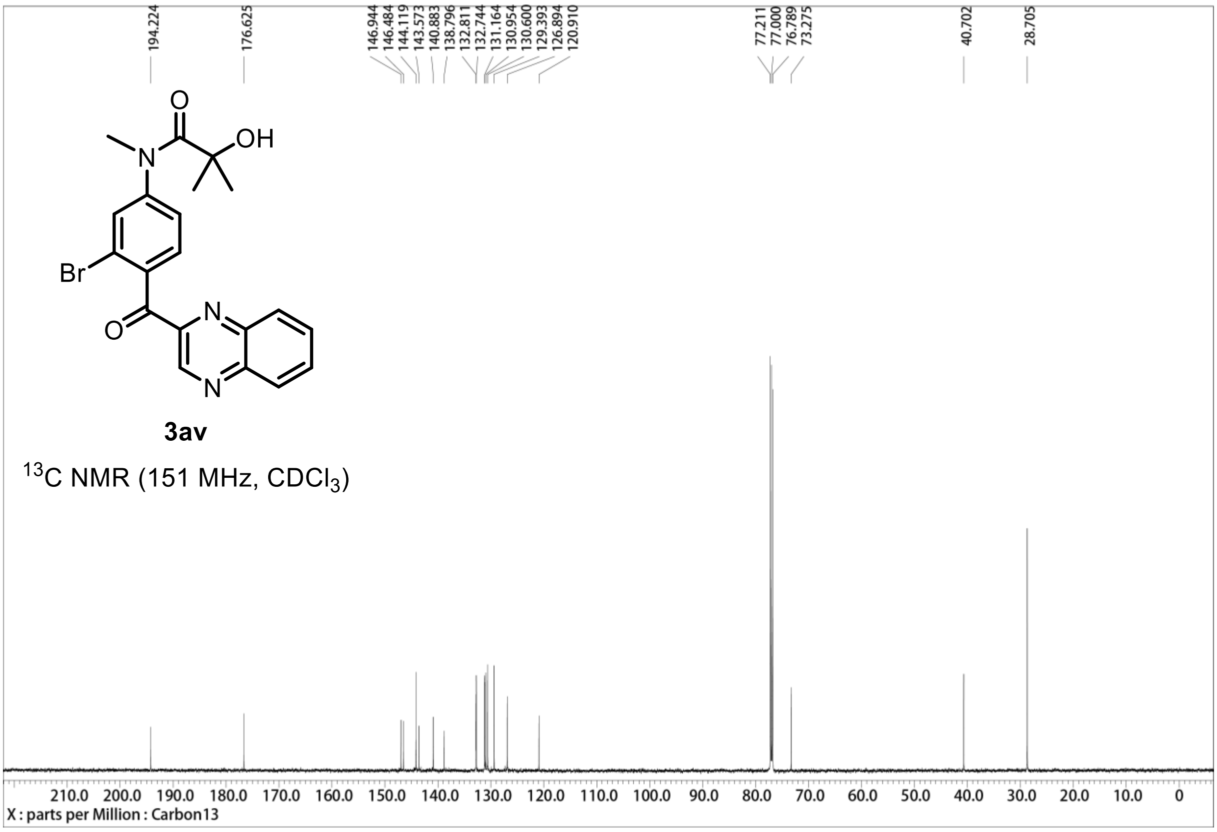

194.224
176.625
146.944
146.484
144.119
143.573
140.883
138.796
132.811
132.744
131.164
130.954
130.600
129.393
126.894
120.910
77.211
77.000
76.789
73.275
40.702
28.705
O
N
OH
Br
O
N
N
3av
^{13}C NMR (151 MHz, $CDCl_3$)
210.0
200.0
190.0
180.0
170.0
160.0
150.0
140.0
130.0
120.0
110.0
100.0
90.0
80.0
70.0
60.0
50.0
40.0
30.0
20.0
10.0
0
X : parts per Million : Carbon13

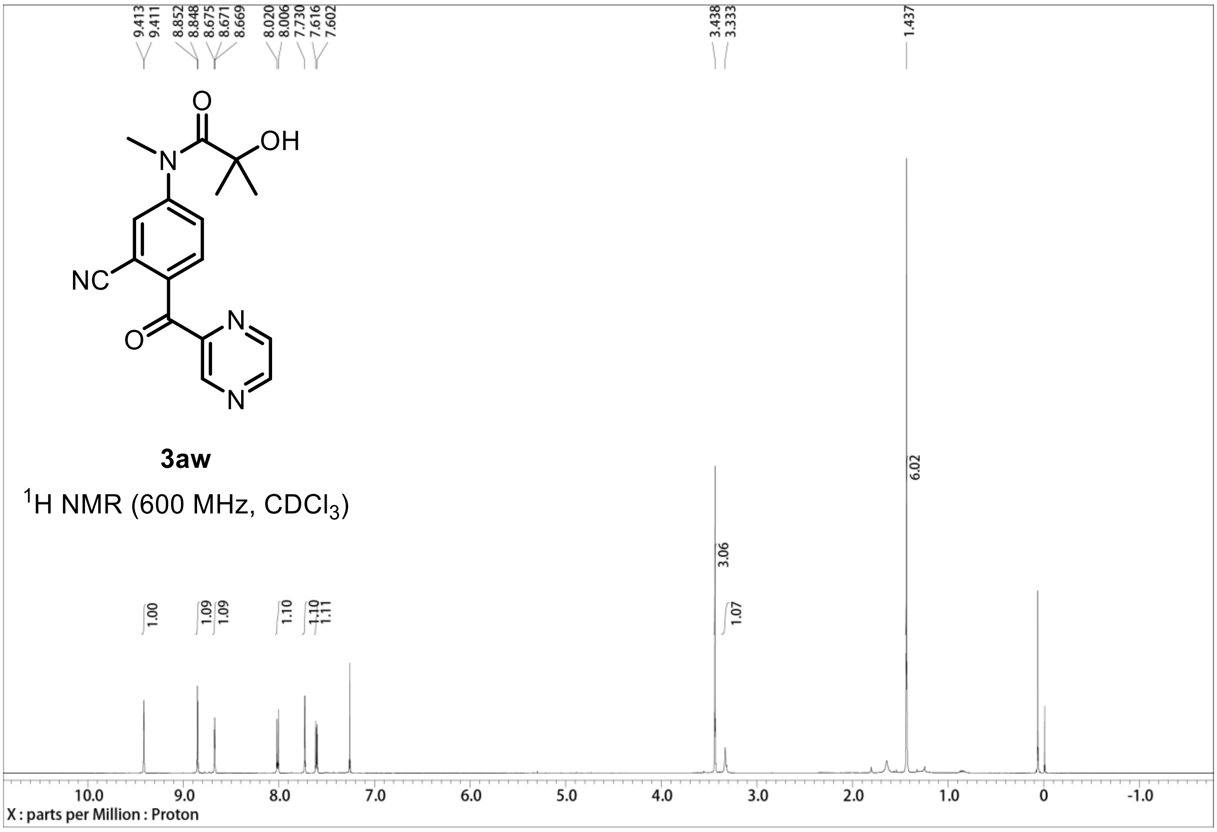
9.413
9.411
8.852
8.848
8.675
8.671
8.669
8.020
8.006
7.730
7.616
7.602
3.438
3.333
1.437
O
OH
N
NC
O
N
N
3aw
^{1}H NMR (600 MHz, CDCl$_3$)
1.00
1.09
1.09
1.10
1.10
1.11
3.06
1.07
6.02
10.0
9.0
8.0
7.0
6.0
5.0
4.0
3.0
2.0
1.0
0
-1.0
X : parts per Million : Proton

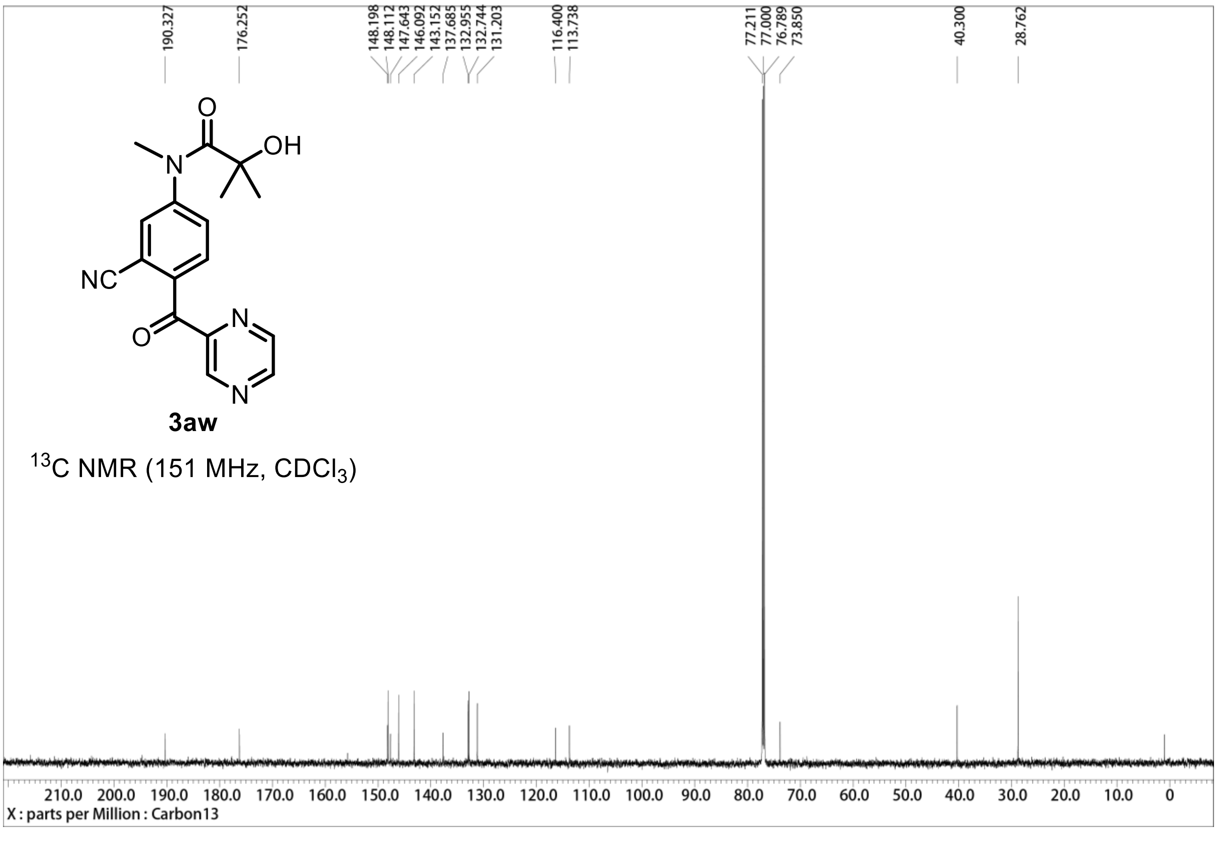
190.327
176.252
148.198
148.112
147.643
146.092
143.152
137.685
132.955
132.744
131.203
116.400
113.738
77.211
77.000
76.789
73.850
40.300
28.762
O
OH
N
NC
O
N
N
3aw
^{13}C NMR (151 MHz, CDCl$_3$)
210.0
200.0
190.0
180.0
170.0
160.0
150.0
140.0
130.0
120.0
110.0
100.0
90.0
80.0
70.0
60.0
50.0
40.0
30.0
20.0
10.0
0
X : parts per Million : Carbon13

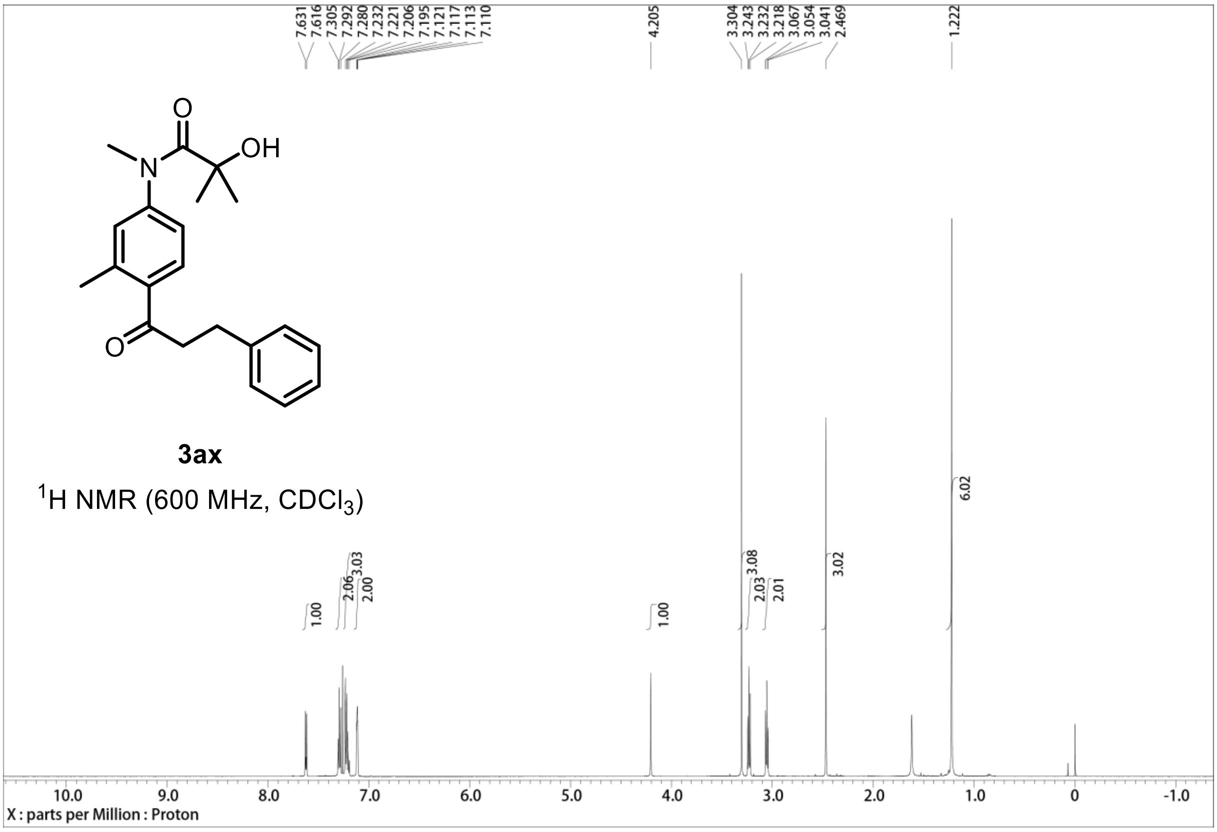
7.631
7.616
7.305
7.292
7.280
7.232
7.221
7.206
7.195
7.121
7.117
7.113
7.110
4.205
3.304
3.243
3.232
3.218
3.067
3.054
3.041
2.469
1.222
OH
N
O
O
3ax
^{1}H NMR (600 MHz, CDCl$_3$)
1.00
2.06
3.03
2.00
1.00
3.08
2.03
2.01
3.02
6.02
10.0
9.0
8.0
7.0
6.0
5.0
4.0
3.0
2.0
1.0
0
-1.0
X : parts per Million : Proton

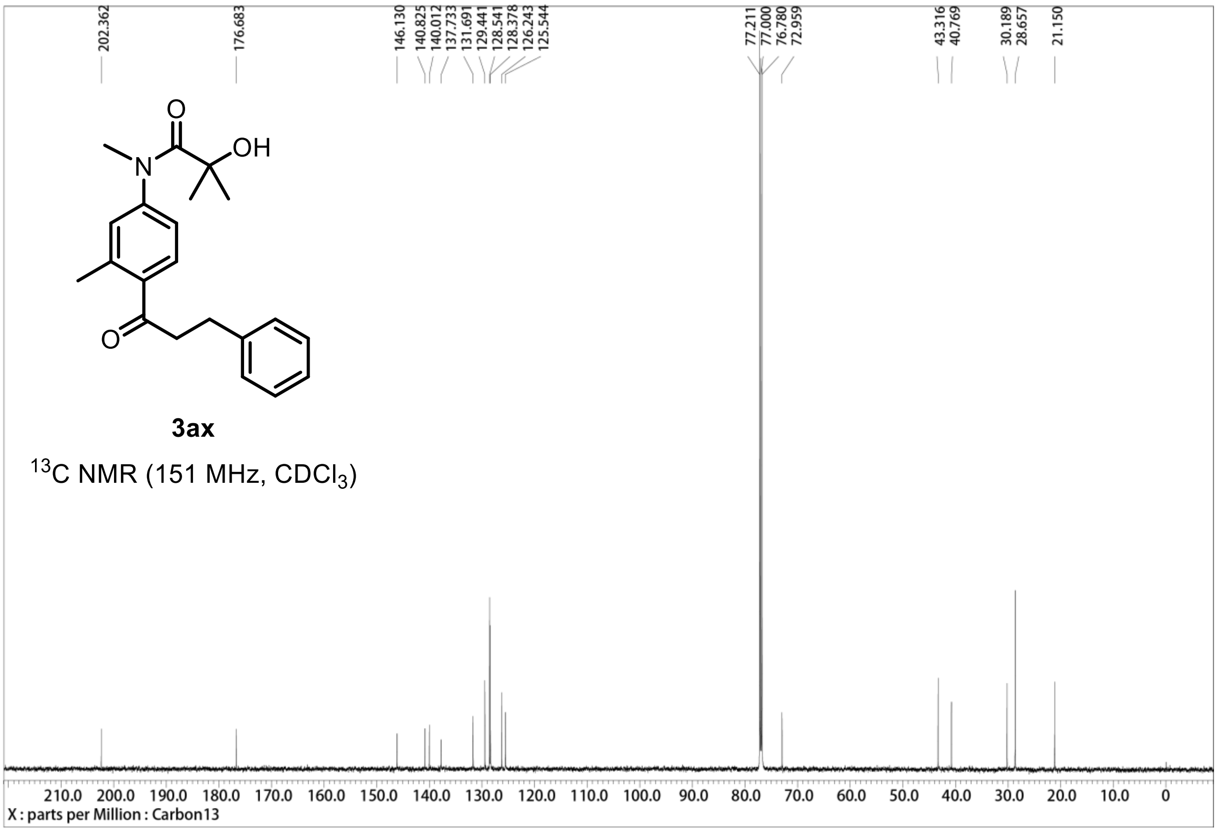
202.362
176.683
146.130
140.825
140.012
137.733
131.691
129.441
128.541
128.378
126.243
125.544
77.211
77.000
76.780
72.959
43.316
40.769
30.189
28.657
21.150
OH
N
O
O
3ax
^{13}C NMR (151 MHz, CDCl$_3$)
210.0
200.0
190.0
180.0
170.0
160.0
150.0
140.0
130.0
120.0
110.0
100.0
90.0
80.0
70.0
60.0
50.0
40.0
30.0
20.0
10.0
0
X : parts per Million : Carbon13

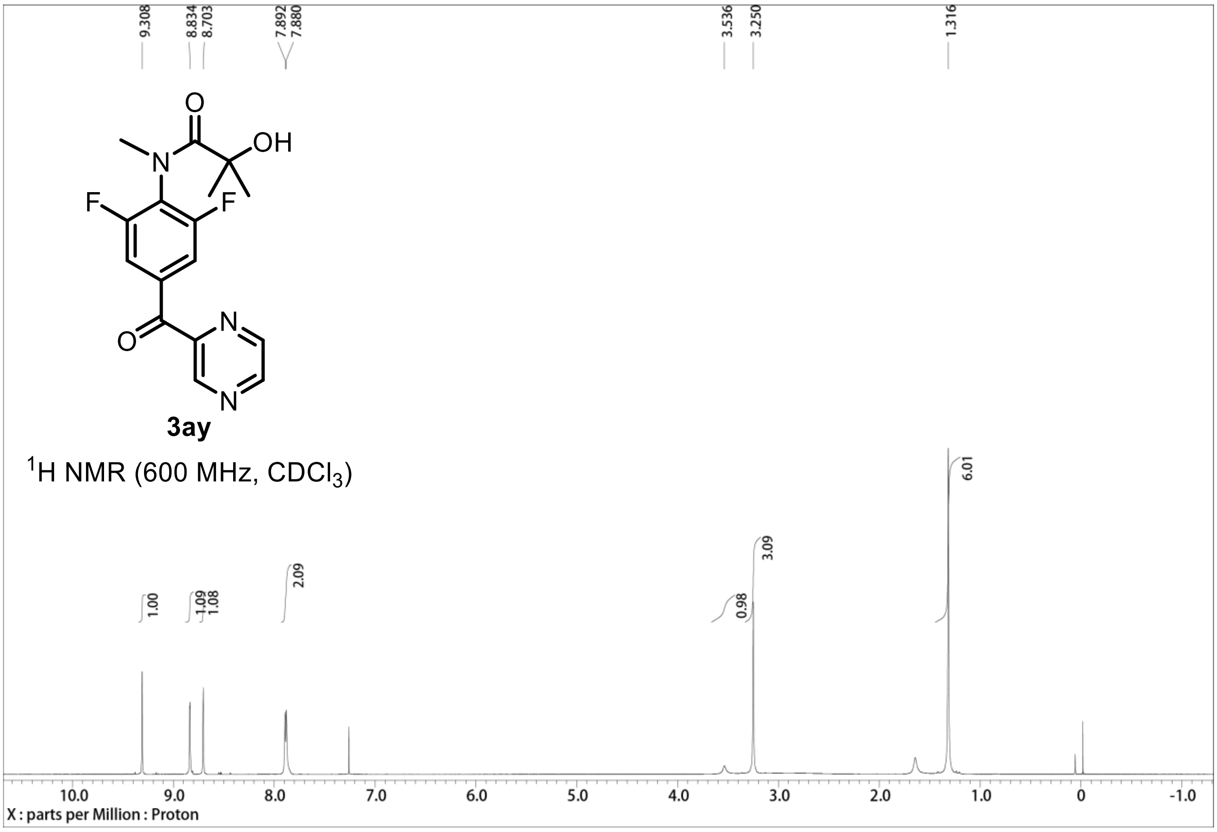
9.308
8.834
8.703
7.892
7.880
3.536
3.250
1.316
O
N
OH
F
F
O
N
N
3ay
^{1}H NMR (600 MHz, $CDCl_3$)
1.00
1.09
1.08
2.09
0.98
3.09
6.01
10.0 9.0 8.0 7.0 6.0 5.0 4.0 3.0 2.0 1.0 0 -1.0
X : parts per Million : Proton

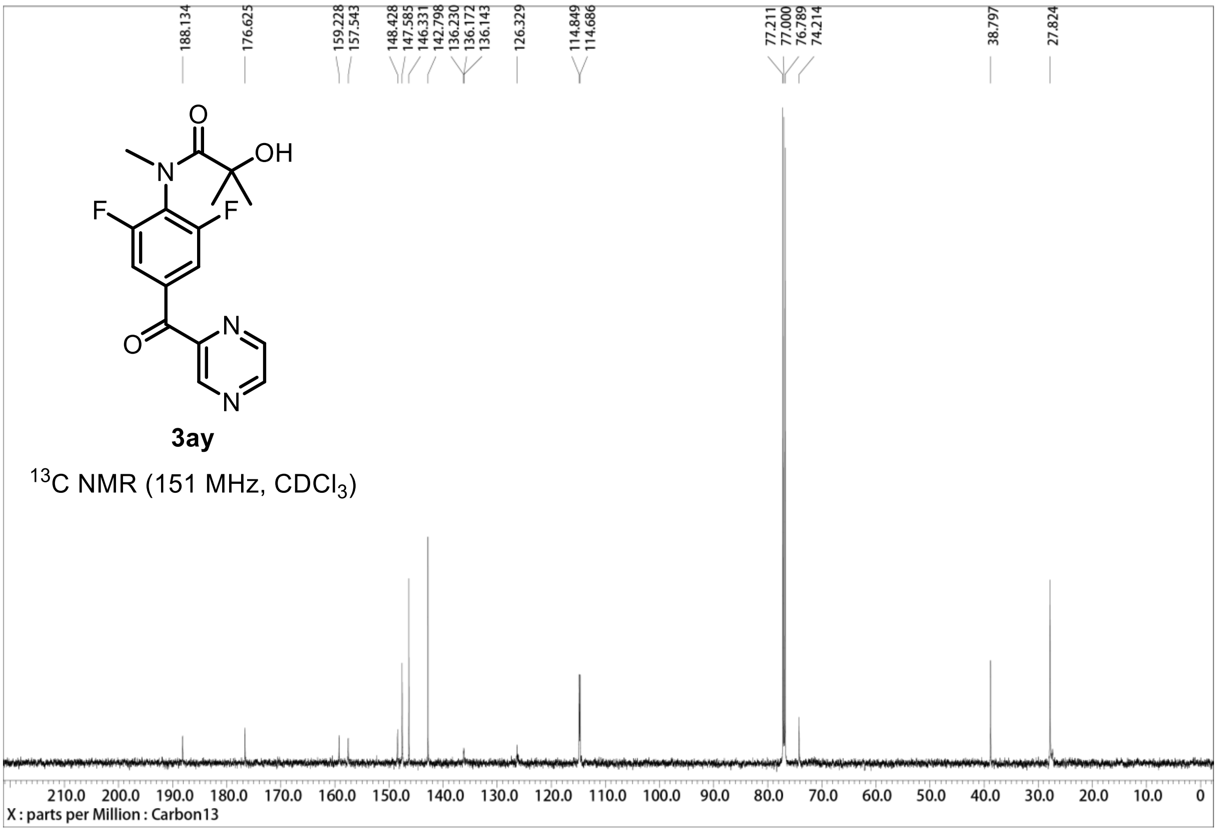
188.134
176.625
159.228
157.543
148.428
147.585
146.331
142.798
136.230
136.172
136.143
126.329
114.849
114.686
77.211
77.000
76.789
74.214
38.797
27.824
O
N
OH
F
F
O
N
N
3ay
^{13}C NMR (151 MHz, $CDCl_3$)
210.0 200.0 190.0 180.0 170.0 160.0 150.0 140.0 130.0 120.0 110.0 100.0 90.0 80.0 70.0 60.0 50.0 40.0 30.0 20.0 10.0 0
X : parts per Million : Carbon13

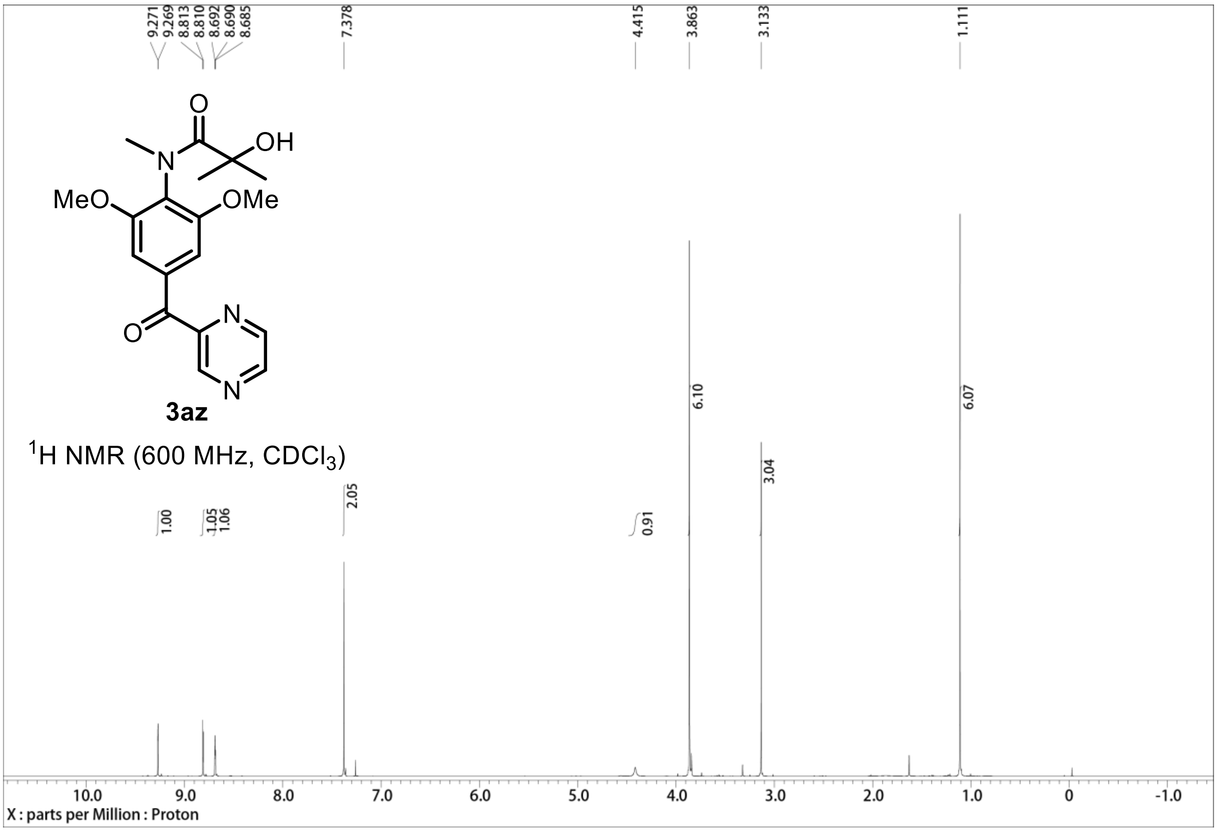

9.271
9.269
8.813
8.810
8.692
8.690
8.685
7.378
4.415
3.863
3.133
1.111
O
OH
N
MeO
OMe
O
N
N
3az
^{1}H NMR (600 MHz, $CDCl_3$)
1.00
1.05
1.06
2.05
0.91
6.10
3.04
6.07
10.0 9.0 8.0 7.0 6.0 5.0 4.0 3.0 2.0 1.0 0 -1.0
X : parts per Million : Proton

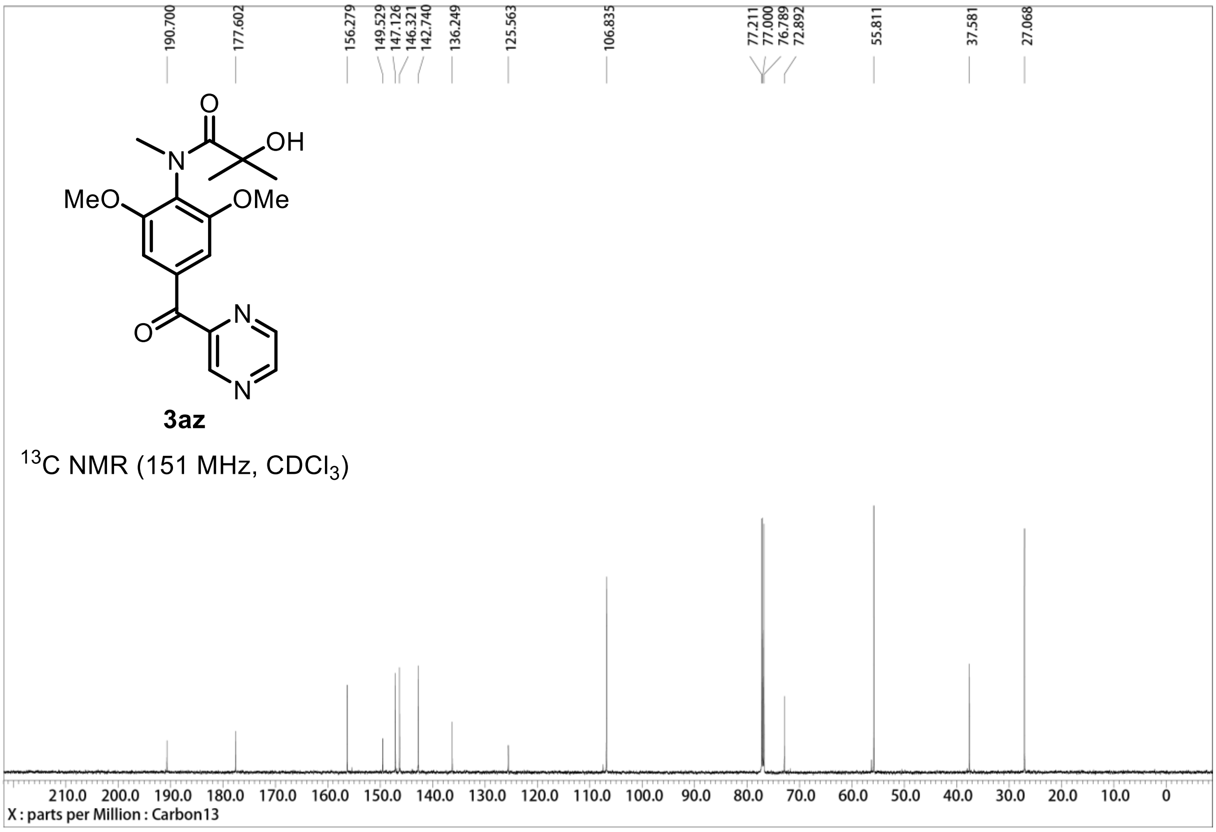

190.700
177.602
156.279
149.529
147.126
146.321
142.740
136.249
125.563
106.835
77.211
77.000
76.789
72.892
55.811
37.581
27.068
O
OH
N
MeO
OMe
O
N
N
3az
^{13}C NMR (151 MHz, $CDCl_3$)
210.0 200.0 190.0 180.0 170.0 160.0 150.0 140.0 130.0 120.0 110.0 100.0 90.0 80.0 70.0 60.0 50.0 40.0 30.0 20.0 10.0 0
X : parts per Million : Carbon13

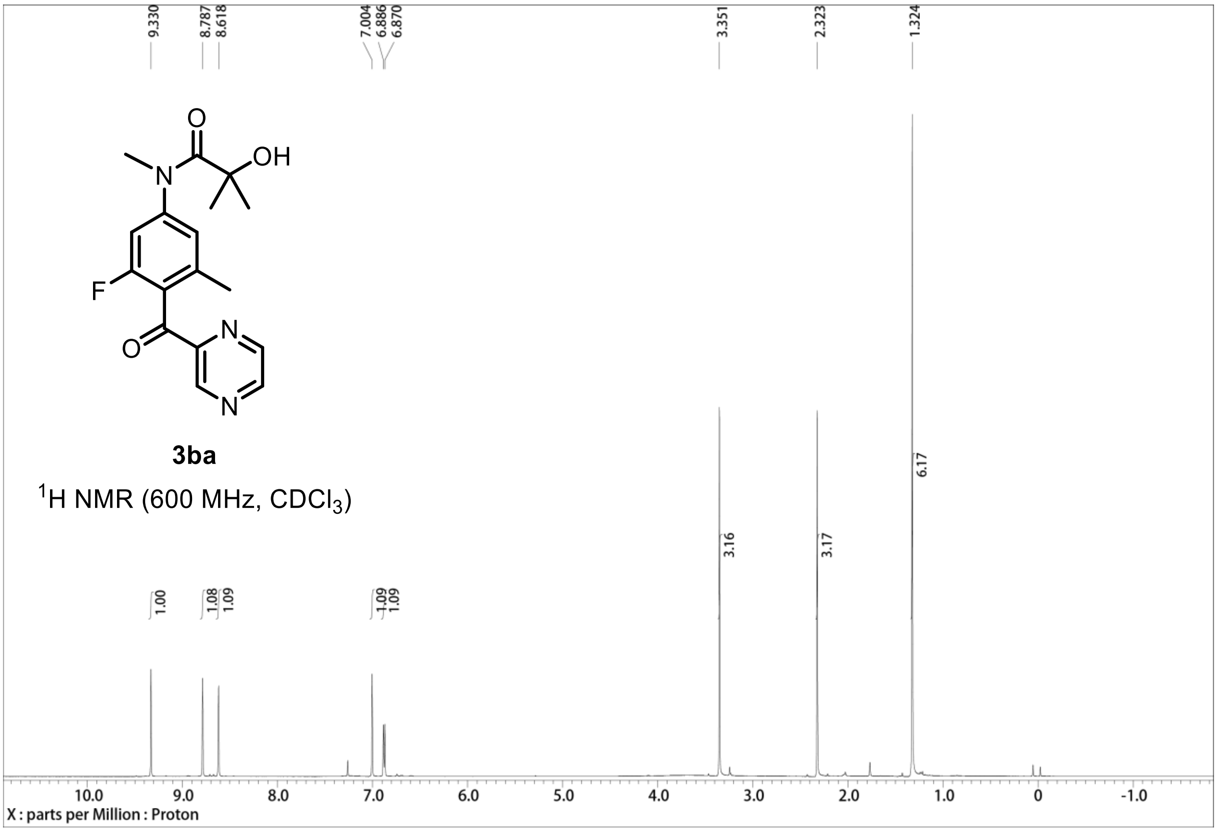
9.330
8.787
8.618
7.004
6.886
6.870
3.351
2.323
1.324
3ba
1H NMR (600 MHz, CDCl3)
1.00
1.08
1.09
1.09
1.09
3.16
3.17
6.17
X : parts per Million : Proton

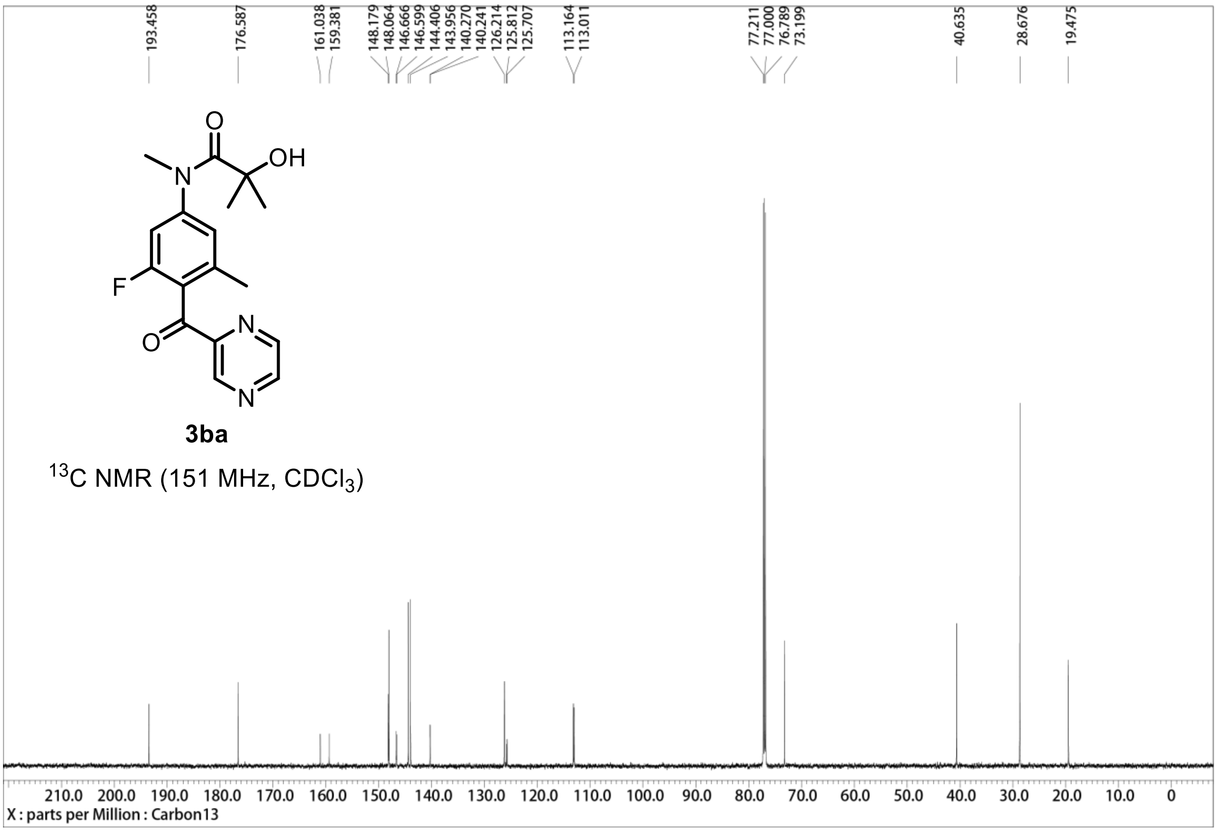
193.458
176.587
161.038
159.381
148.179
148.064
146.666
146.599
144.406
143.956
140.270
140.241
126.214
125.812
125.707
113.164
113.011
77.211
77.000
76.789
73.199
40.635
28.676
19.475
3ba
13C NMR (151 MHz, CDCl3)
X : parts per Million : Carbon13

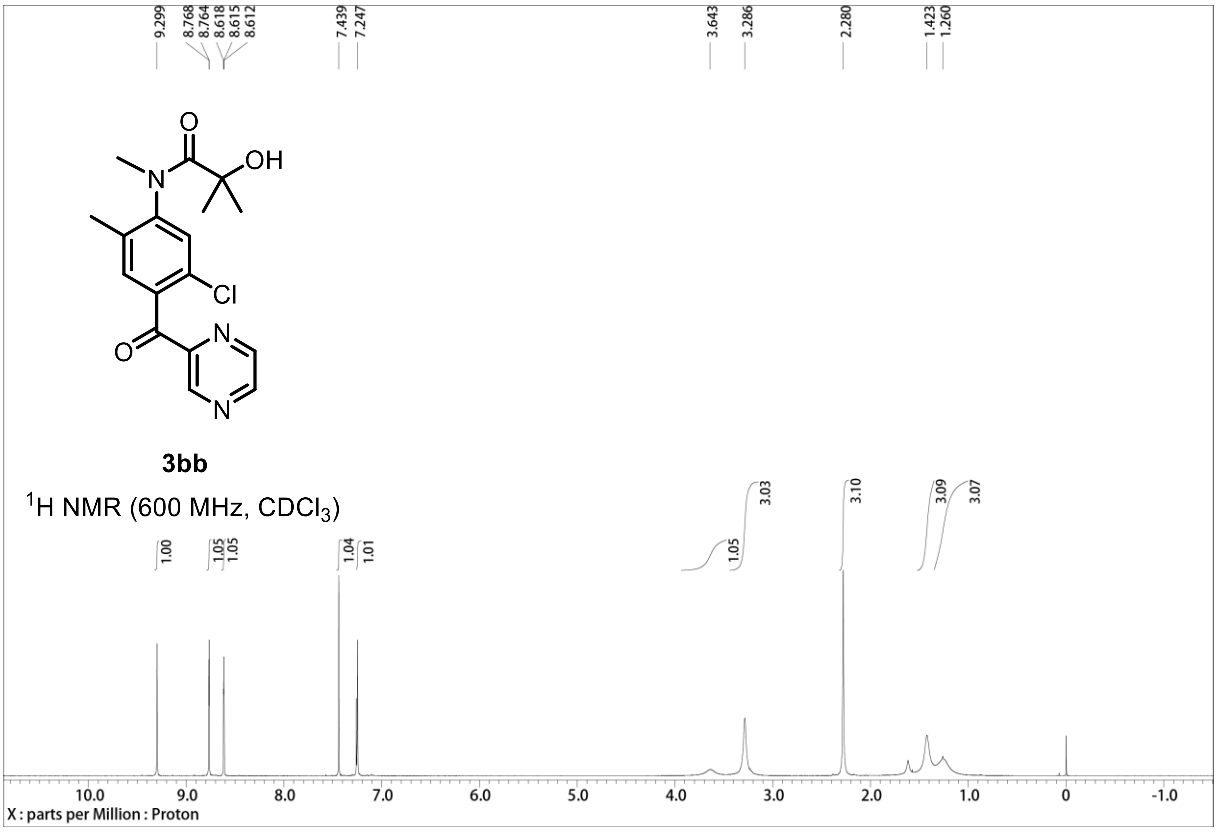

9.299
8.768
8.764
8.618
8.615
8.612
7.439
7.247
3.643
3.286
2.280
1.423
1.260
O
N
OH
Cl
O
N
N
3bb
1H NMR (600 MHz, CDCl3)
1.00
1.05
1.05
1.04
1.01
1.05
3.03
3.10
3.09
3.07
10.0
9.0
8.0
7.0
6.0
5.0
4.0
3.0
2.0
1.0
0
-1.0
X : parts per Million : Proton

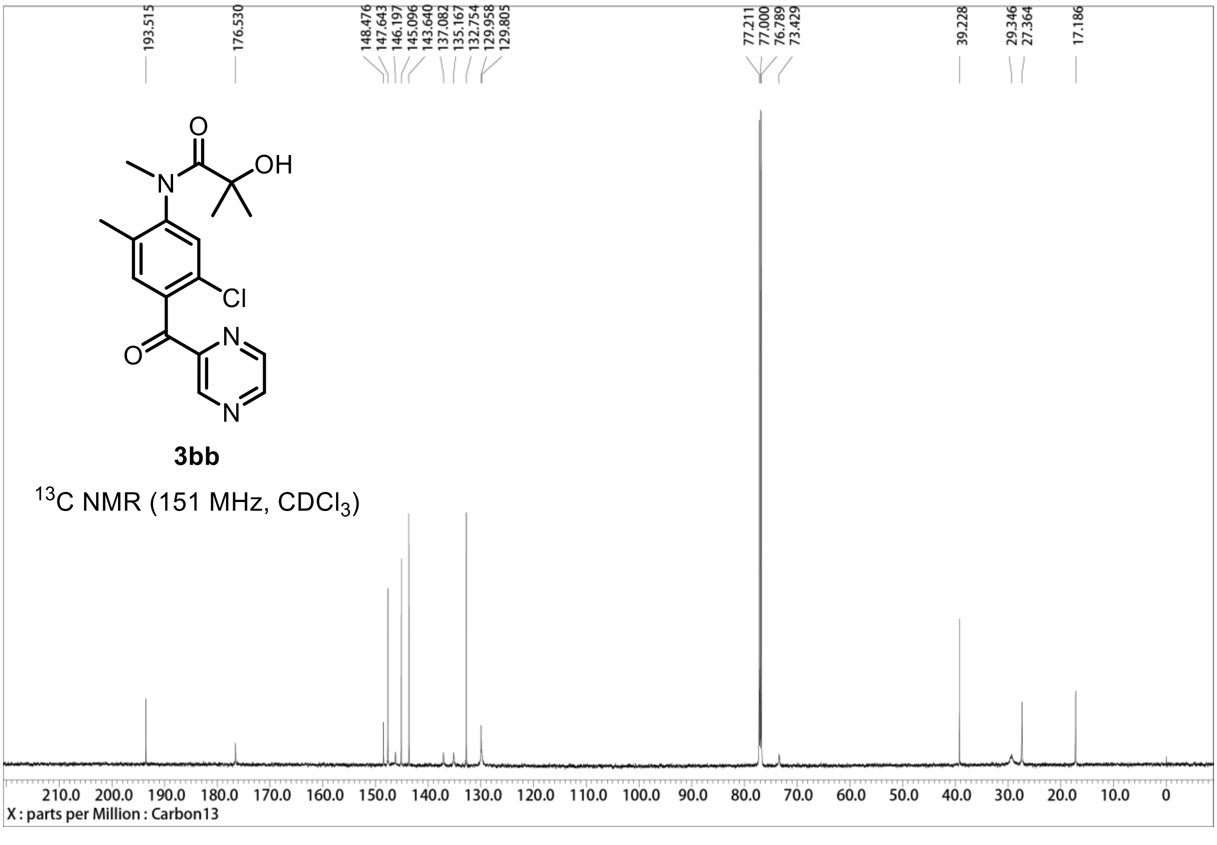

193.515
176.530
148.476
147.643
146.197
145.096
143.640
137.082
135.167
132.754
129.958
129.805
77.211
77.000
76.789
73.429
39.228
29.346
27.364
17.186
O
N
OH
Cl
O
N
N
3bb
13C NMR (151 MHz, CDCl3)
210.0
200.0
190.0
180.0
170.0
160.0
150.0
140.0
130.0
120.0
110.0
100.0
90.0
80.0
70.0
60.0
50.0
40.0
30.0
20.0
10.0
0
X : parts per Million : Carbon13

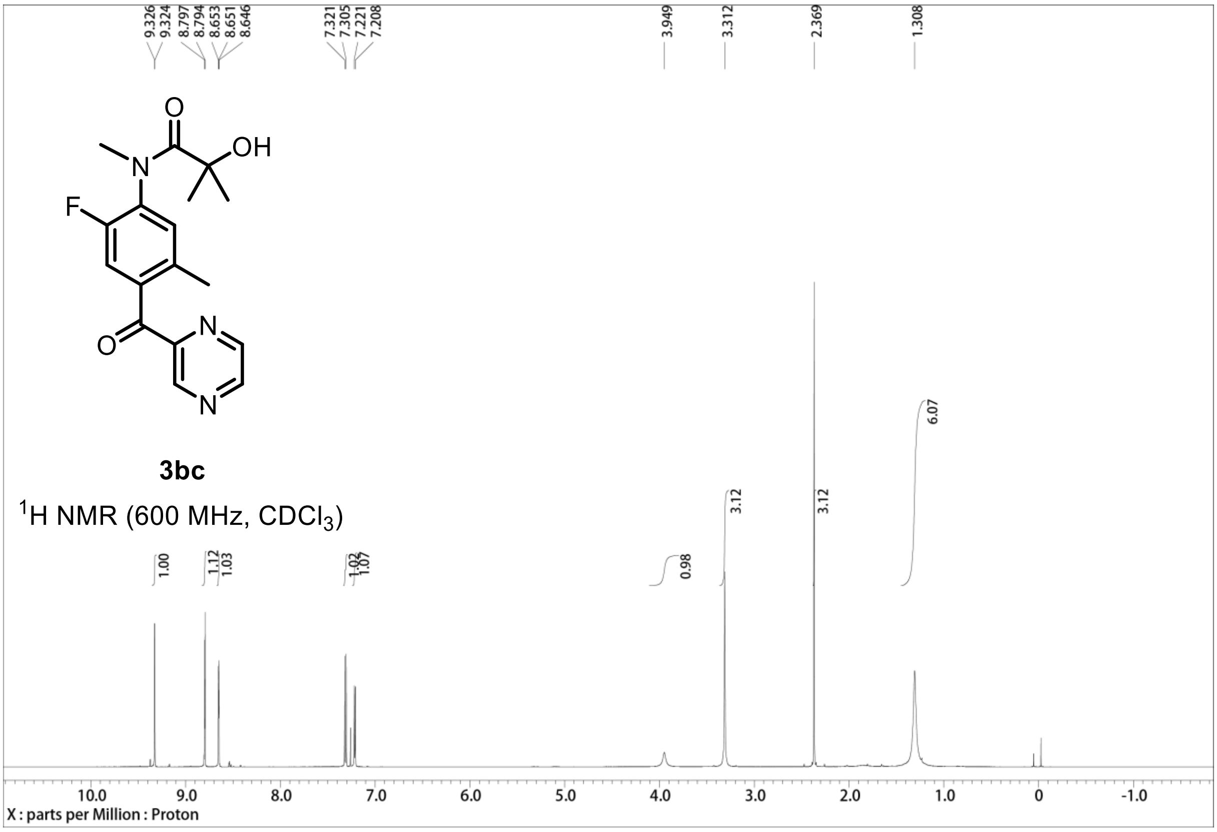
9.326
9.324
8.797
8.794
8.653
8.651
8.646
7.321
7.305
7.221
7.208
3.949
3.312
2.369
1.308
O
N
OH
F
O
N
N
3bc
1H NMR (600 MHz, CDCl3)
1.00
1.12
1.03
1.02
1.07
0.98
3.12
3.12
6.07
10.0
9.0
8.0
7.0
6.0
5.0
4.0
3.0
2.0
1.0
0
-1.0
X : parts per Million : Proton

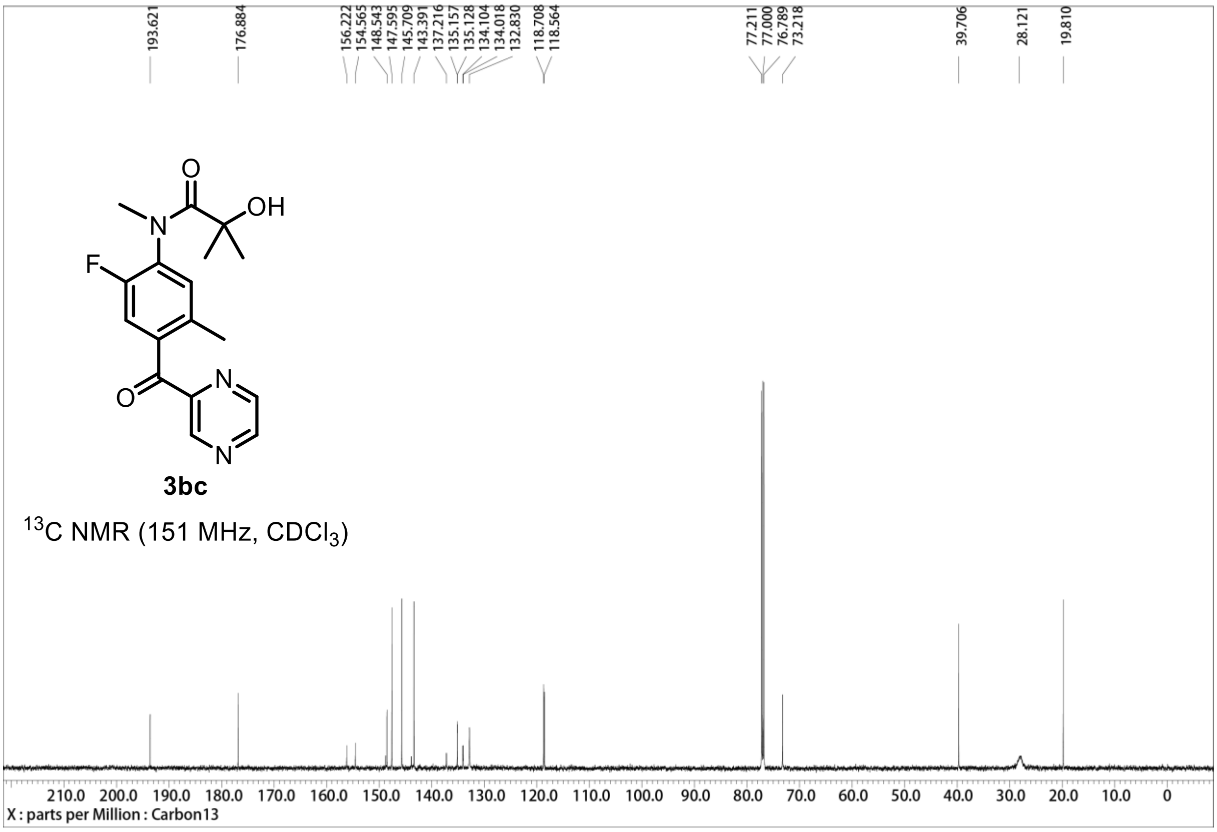
193.621
176.884
156.222
154.565
148.543
147.595
145.709
143.391
137.216
135.157
135.128
134.104
134.018
132.830
118.708
118.564
77.211
77.000
76.789
73.218
39.706
28.121
19.810
O
N
OH
F
O
N
N
3bc
13C NMR (151 MHz, CDCl3)
210.0
200.0
190.0
180.0
170.0
160.0
150.0
140.0
130.0
120.0
110.0
100.0
90.0
80.0
70.0
60.0
50.0
40.0
30.0
20.0
10.0
0
X : parts per Million : Carbon13

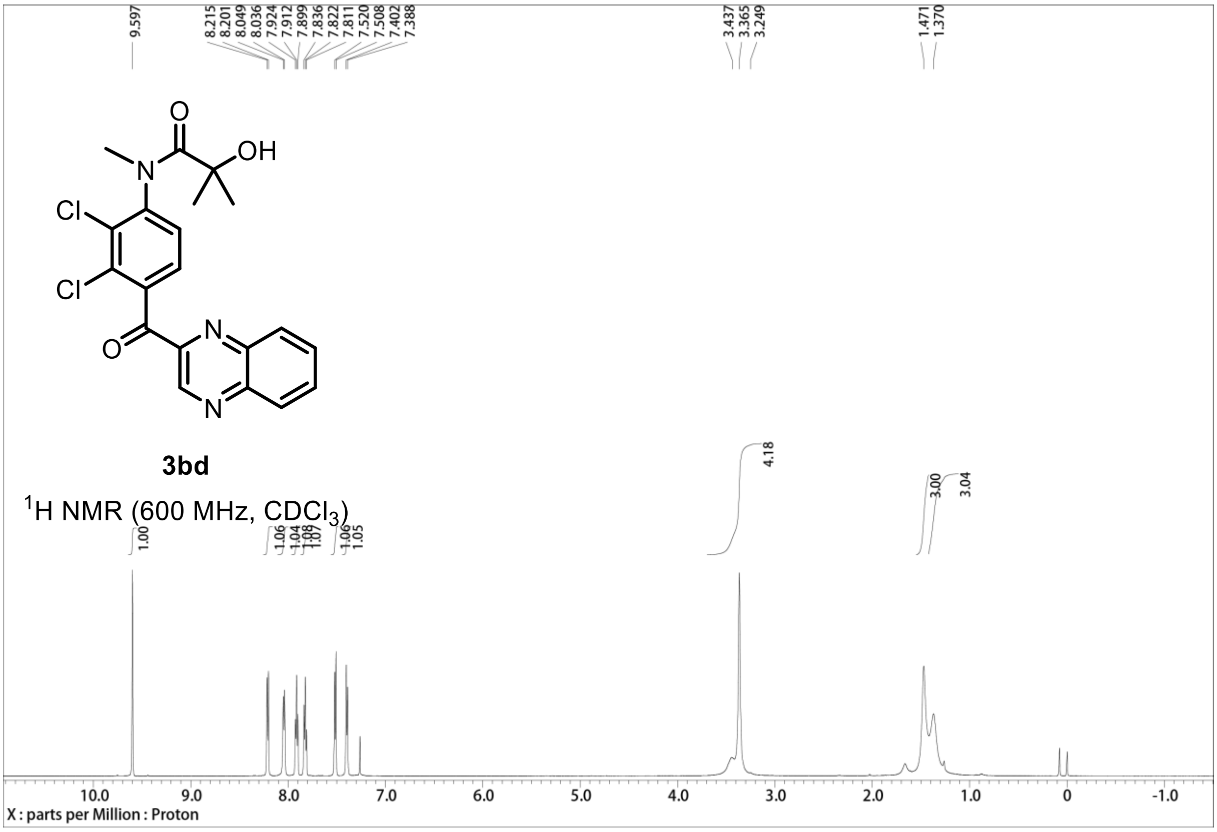
9.597
8.215
8.201
8.049
8.036
7.924
7.912
7.899
7.836
7.822
7.811
7.520
7.508
7.402
7.388
3.437
3.365
3.249
1.471
1.370
O
OH
N
Cl
Cl
O
N
N
3bd
^{1}H NMR (600 MHz, $CDCl_3$)
1.00
1.06
1.04
1.09
1.08
1.07
1.06
1.05
4.18
3.00
3.04
10.0
9.0
8.0
7.0
6.0
5.0
4.0
3.0
2.0
1.0
0
-1.0
X : parts per Million : Proton

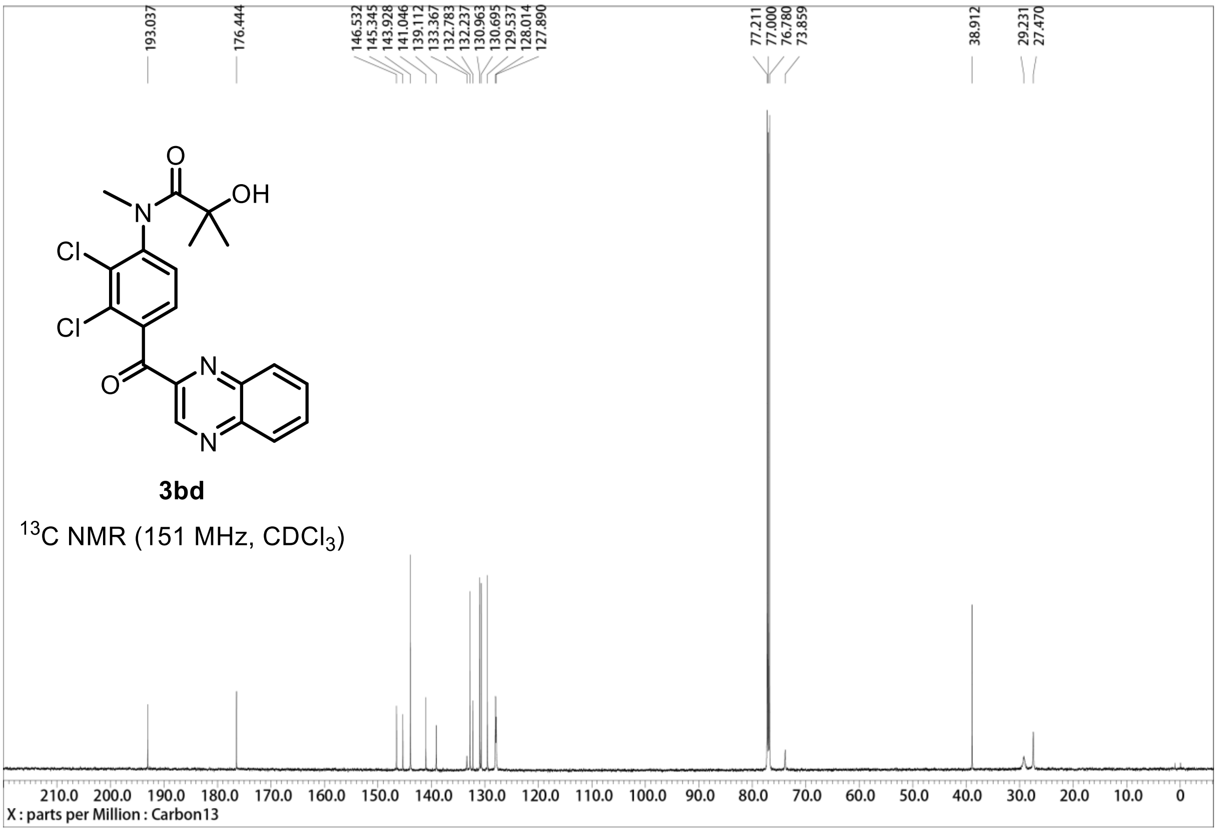
193.037
176.444
146.532
145.345
143.928
141.046
139.112
133.367
132.783
132.237
130.963
130.695
129.537
128.014
127.890
77.211
77.000
76.780
73.859
38.912
29.231
27.470
O
OH
N
Cl
Cl
O
N
N
3bd
^{13}C NMR (151 MHz, $CDCl_3$)
210.0
200.0
190.0
180.0
170.0
160.0
150.0
140.0
130.0
120.0
110.0
100.0
90.0
80.0
70.0
60.0
50.0
40.0
30.0
20.0
10.0
0
X : parts per Million : Carbon13

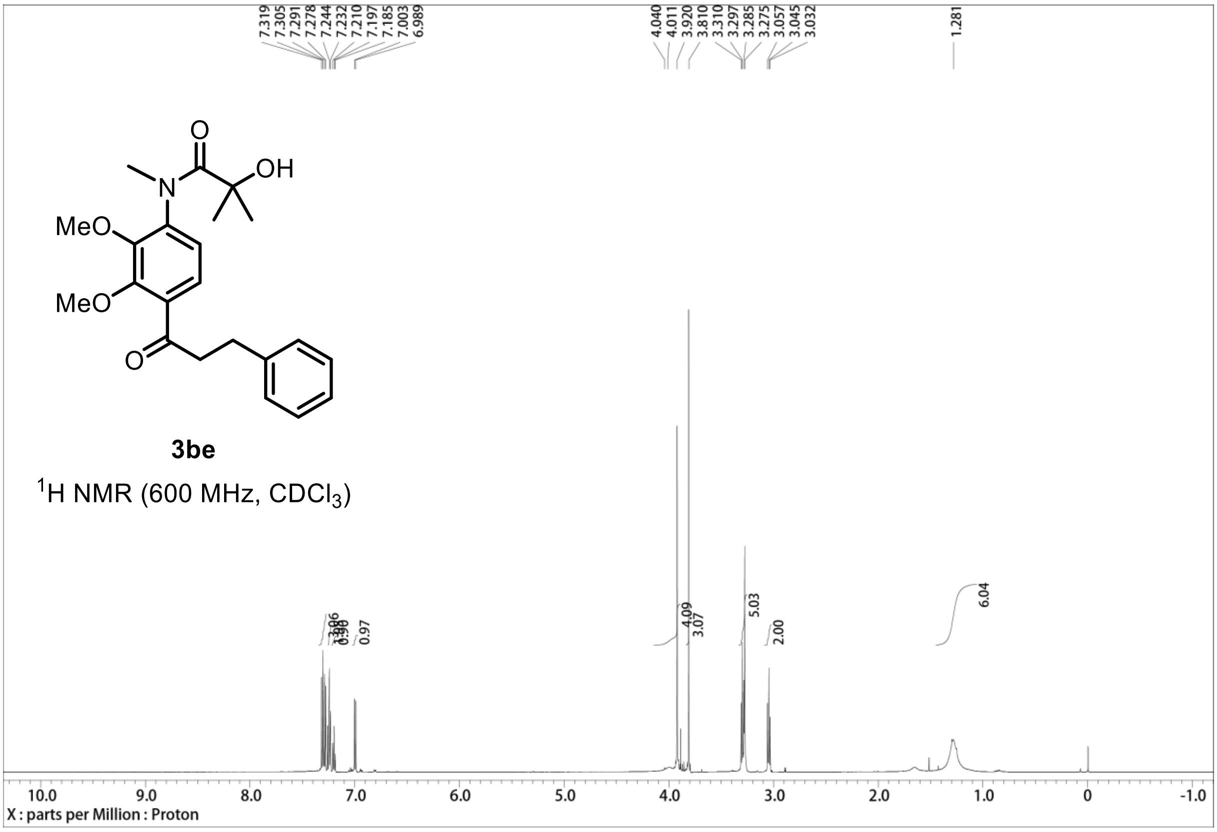
7.319
7.305
7.291
7.278
7.244
7.232
7.210
7.197
7.185
7.003
6.989
4.040
4.011
3.920
3.810
3.310
3.297
3.285
3.275
3.057
3.045
3.032
1.281
O
N
OH
MeO
MeO
O
3be
^{1}H NMR (600 MHz, CDCl$_3$)
3.06
0.97
4.09
3.07
5.03
2.00
6.04
10.0
9.0
8.0
7.0
6.0
5.0
4.0
3.0
2.0
1.0
0
-1.0
X : parts per Million : Proton

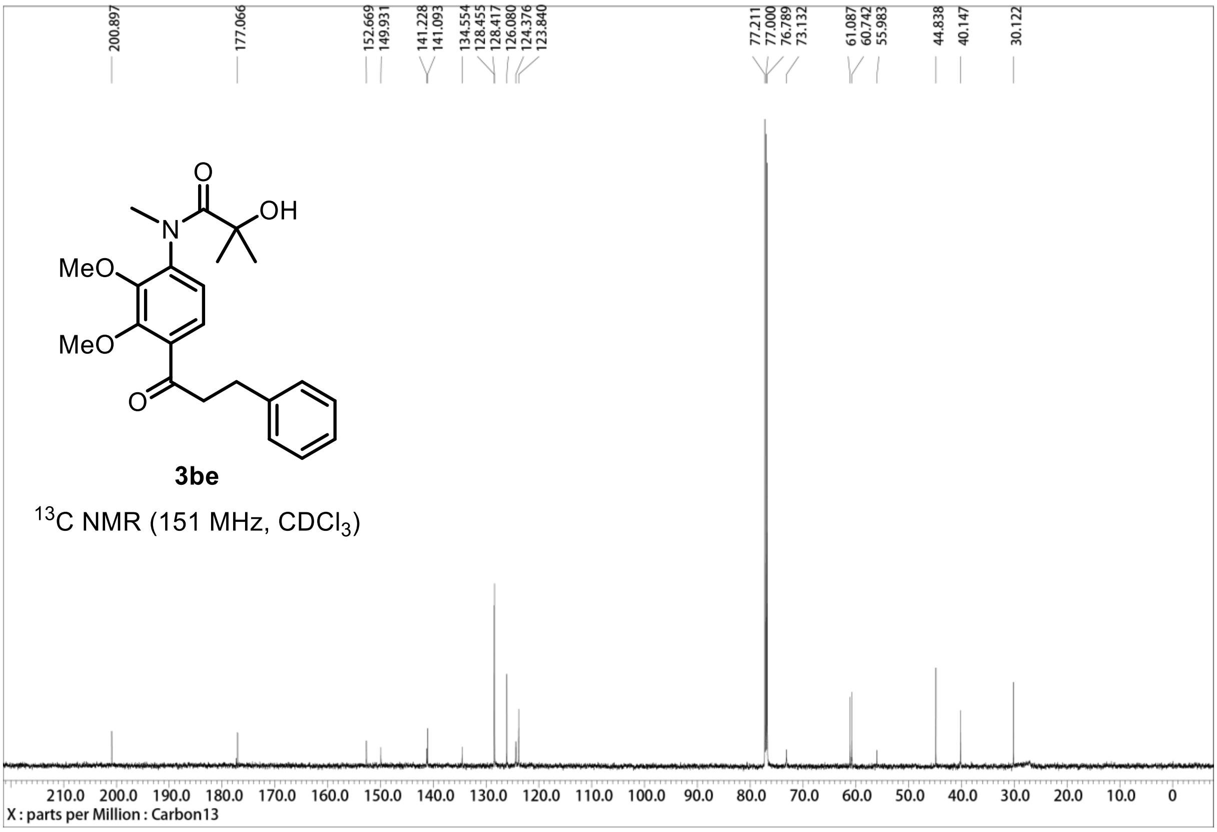
200.897
177.066
152.669
149.931
141.228
141.093
134.554
128.455
128.417
126.080
124.376
123.840
77.211
77.000
76.789
73.132
61.087
60.742
55.983
44.838
40.147
30.122
O
N
OH
MeO
MeO
O
3be
^{13}C NMR (151 MHz, CDCl$_3$)
210.0
200.0
190.0
180.0
170.0
160.0
150.0
140.0
130.0
120.0
110.0
100.0
90.0
80.0
70.0
60.0
50.0
40.0
30.0
20.0
10.0
0
X : parts per Million : Carbon13

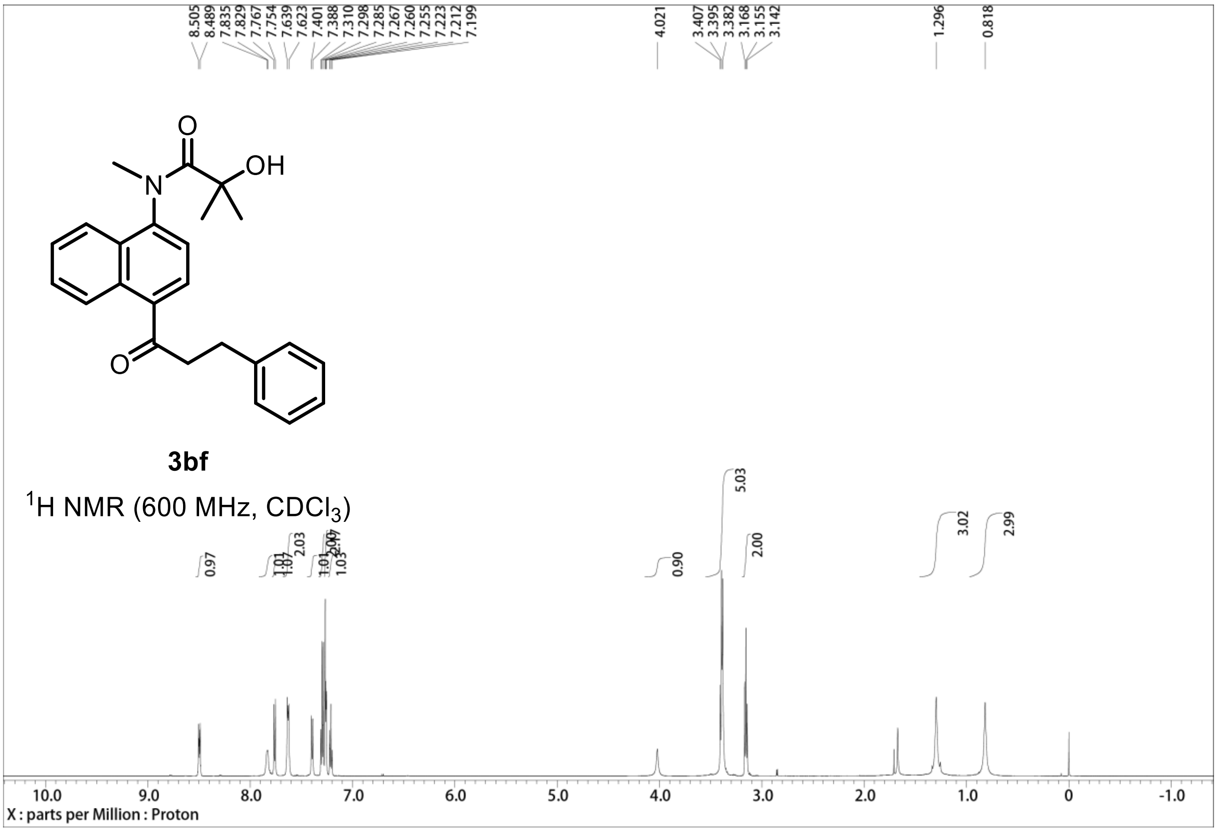
8.505
8.489
7.835
7.829
7.767
7.754
7.639
7.623
7.401
7.388
7.310
7.298
7.285
7.267
7.260
7.255
7.223
7.212
7.199
4.021
3.407
3.395
3.382
3.168
3.155
3.142
1.296
0.818
O
N
OH
O
3bf
1H NMR (600 MHz, CDCl3)
X : parts per Million : Proton

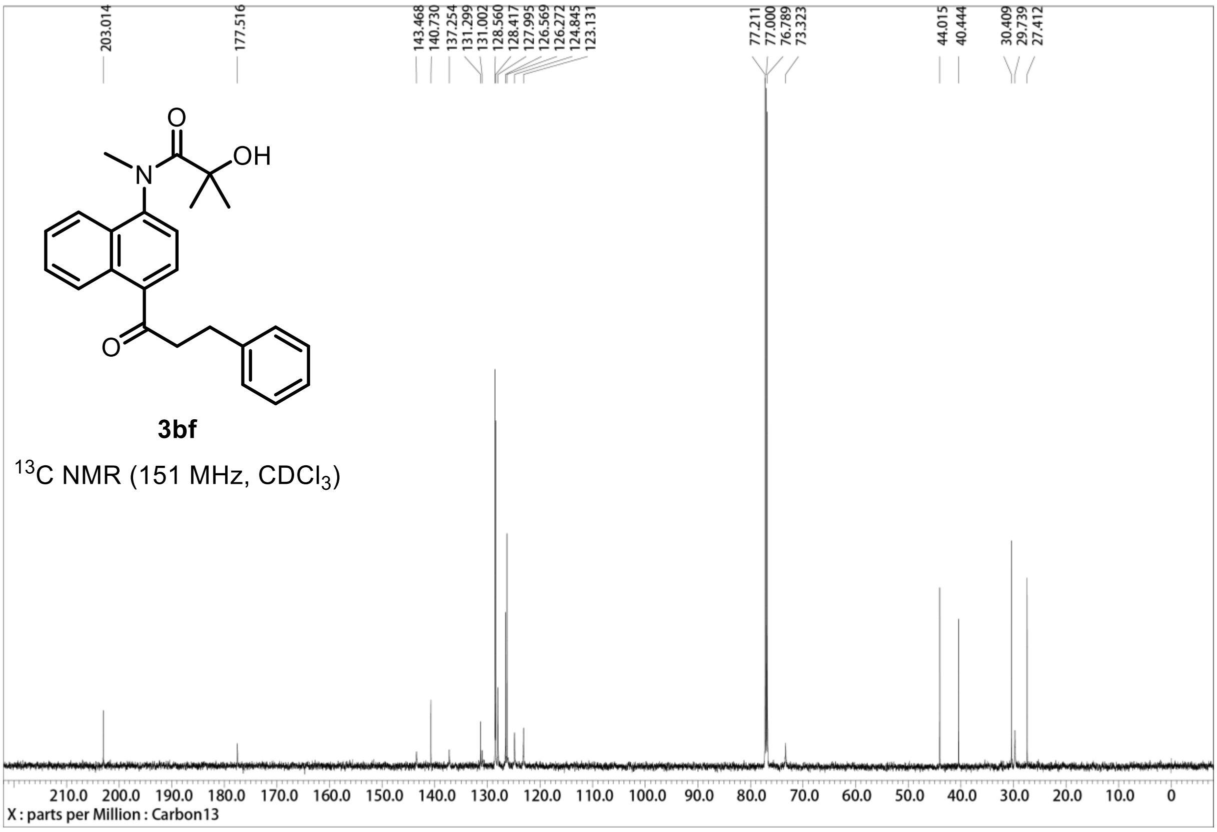
203.014
177.516
143.468
140.730
137.254
131.299
131.002
128.560
128.417
127.995
126.569
126.272
124.845
123.131
77.211
77.000
76.789
73.323
44.015
40.444
30.409
29.739
27.412
O
N
OH
O
3bf
13C NMR (151 MHz, CDCl3)
X : parts per Million : Carbon13

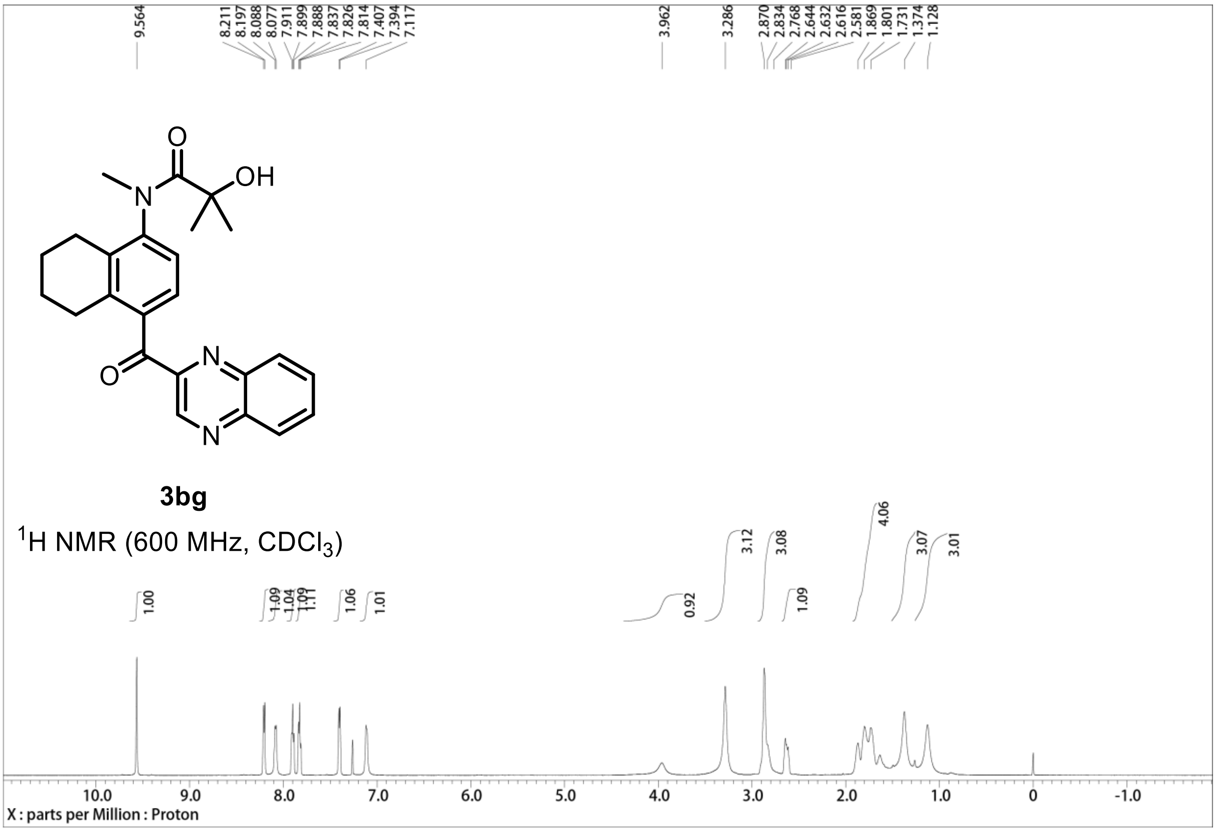
9.564
8.211 8.197 8.088 8.077 7.911 7.899 7.888 7.837 7.826 7.814 7.407 7.394 7.117
3.962
3.286
2.870 2.834 2.768 2.644 2.632 2.616 2.581 1.869 1.801 1.731 1.374 1.128
O
OH
N
N
N
O
3bg
^{1}H NMR (600 MHz, $CDCl_3$)
1.00
1.09 1.04 1.09 1.11
1.06 1.01
0.92
3.12
3.08
1.09
4.06
3.07
3.01
10.0 9.0 8.0 7.0 6.0 5.0 4.0 3.0 2.0 1.0 0 -1.0
X : parts per Million : Proton

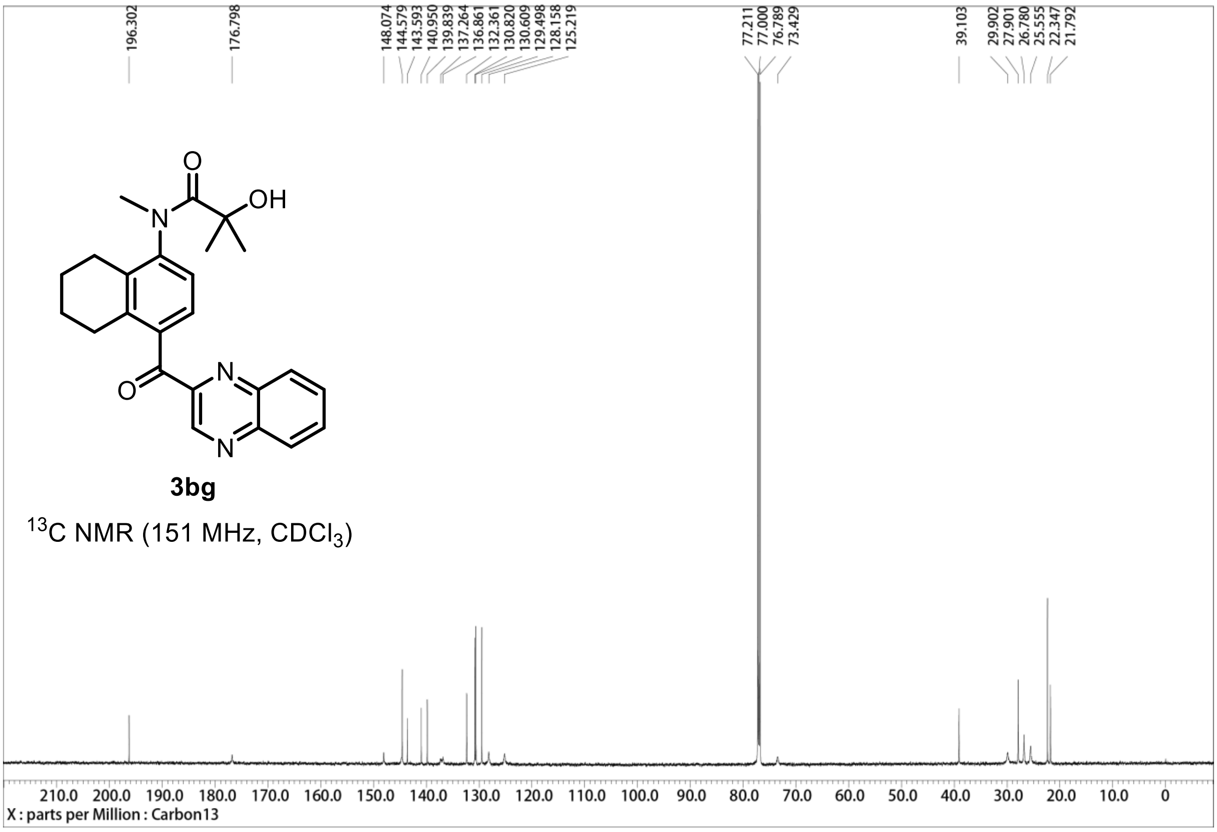
196.302
176.798
148.074 144.579 143.593 140.950 139.839 137.264 136.861 132.361 130.820 130.609 129.498 128.158 125.219
77.211 77.000 76.789 73.429
39.103
29.902 27.901 26.780 25.555 22.347 21.792
O
OH
N
N
N
O
3bg
^{13}C NMR (151 MHz, $CDCl_3$)
210.0 200.0 190.0 180.0 170.0 160.0 150.0 140.0 130.0 120.0 110.0 100.0 90.0 80.0 70.0 60.0 50.0 40.0 30.0 20.0 10.0 0
X : parts per Million : Carbon13

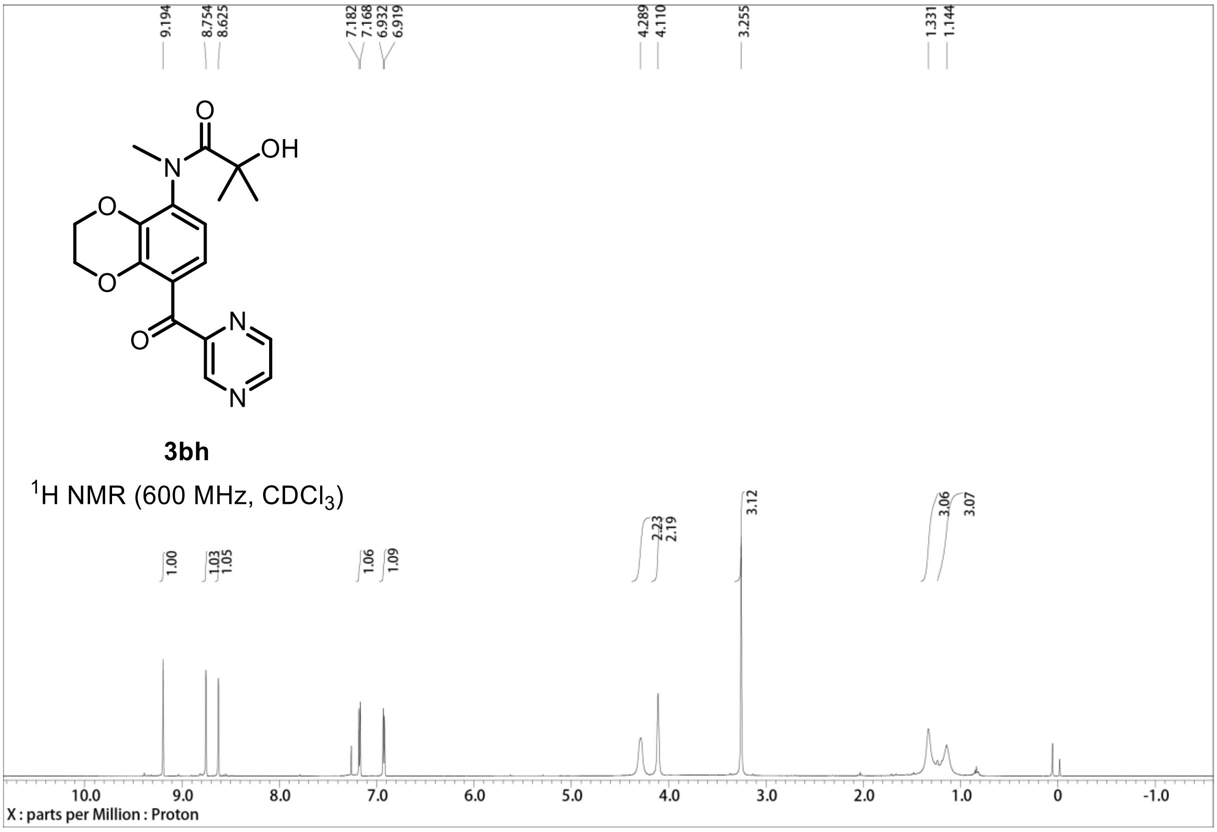
9.194
8.754
8.625
7.182
7.168
6.932
6.919
4.289
4.110
3.255
1.331
1.144
O
N
OH
O
O
O
N
N
3bh
1H NMR (600 MHz, CDCl3)
1.00
1.03
1.05
1.06
1.09
2.23
2.19
3.12
3.06
3.07
10.0
9.0
8.0
7.0
6.0
5.0
4.0
3.0
2.0
1.0
0
-1.0
X : parts per Million : Proton

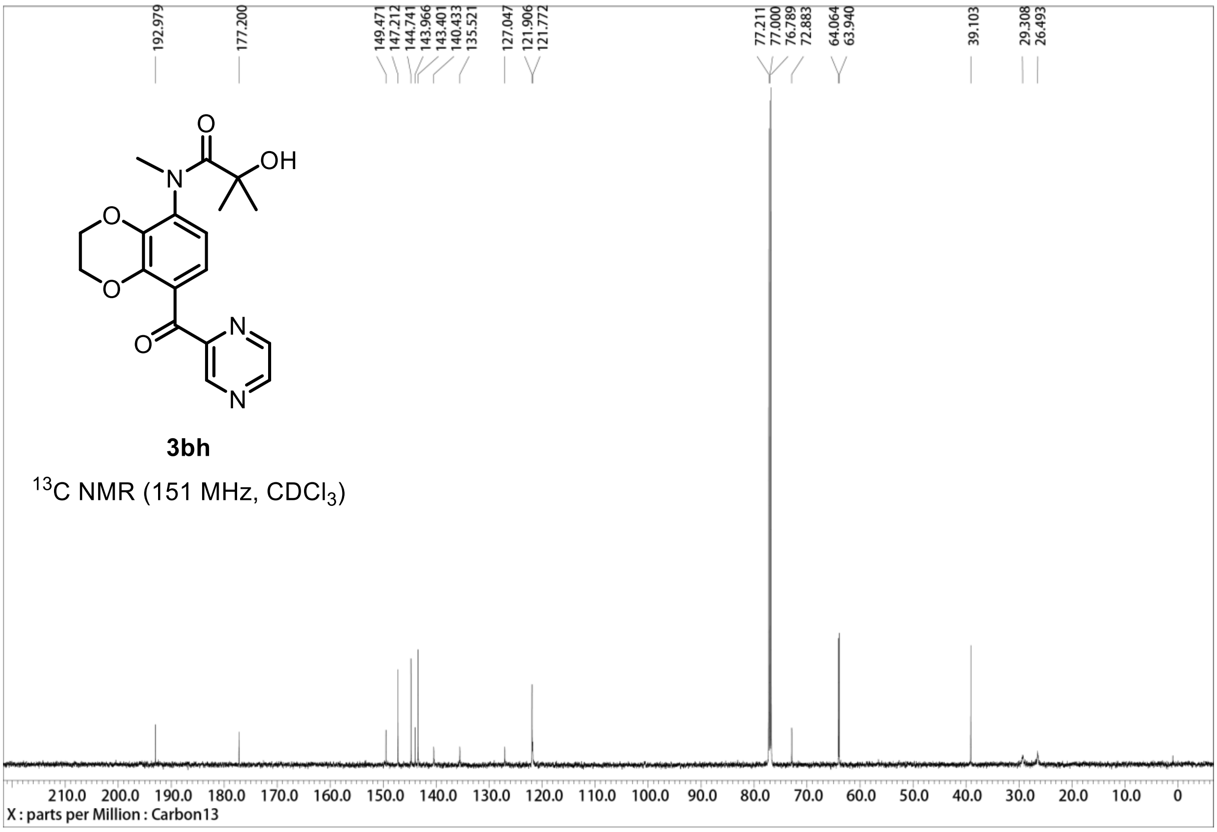
192.979
177.200
149.471
147.212
144.741
143.966
143.401
140.433
135.521
127.047
121.906
121.772
77.211
77.000
76.789
72.883
64.064
63.940
39.103
29.308
26.493
O
N
OH
O
O
O
N
N
3bh
13C NMR (151 MHz, CDCl3)
210.0
200.0
190.0
180.0
170.0
160.0
150.0
140.0
130.0
120.0
110.0
100.0
90.0
80.0
70.0
60.0
50.0
40.0
30.0
20.0
10.0
0
X : parts per Million : Carbon13

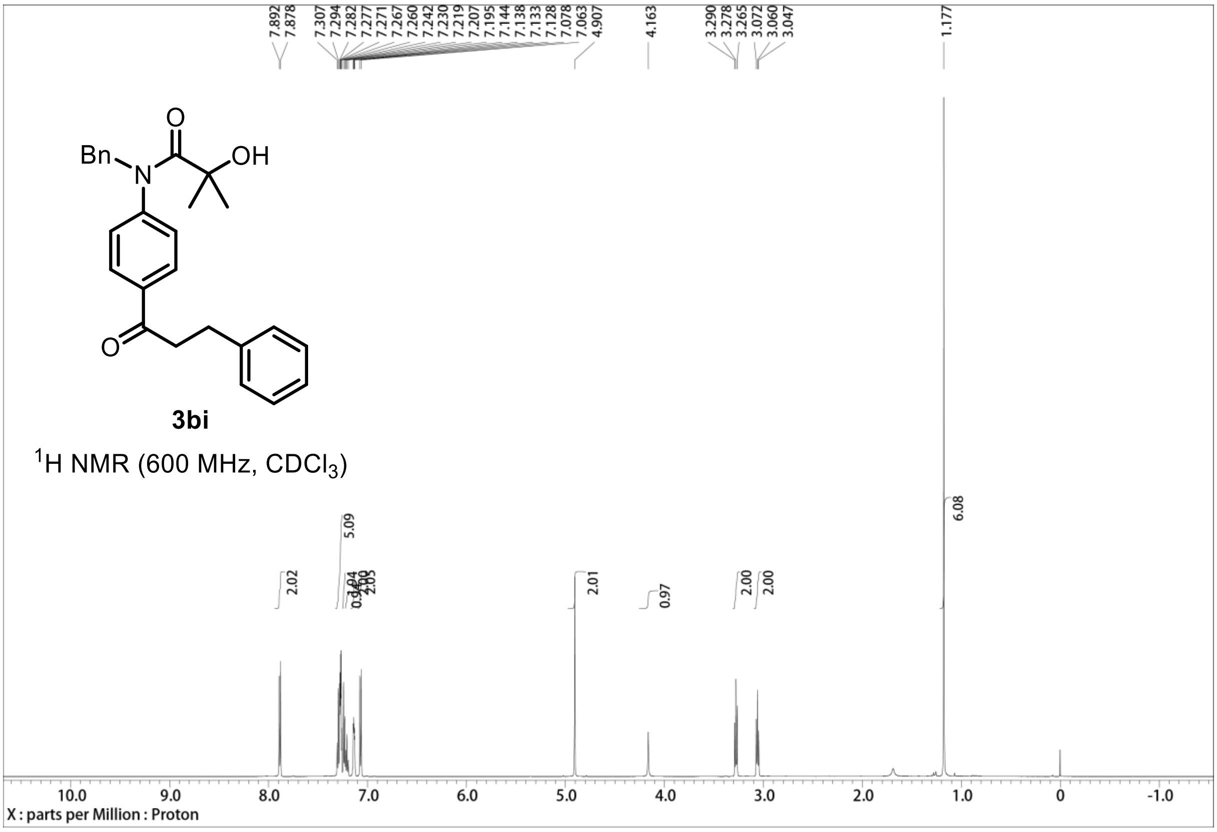

Bn
N
O
OH
O
3bi
^{1}H NMR (600 MHz, CDCl$_3$)
X : parts per Million : Proton

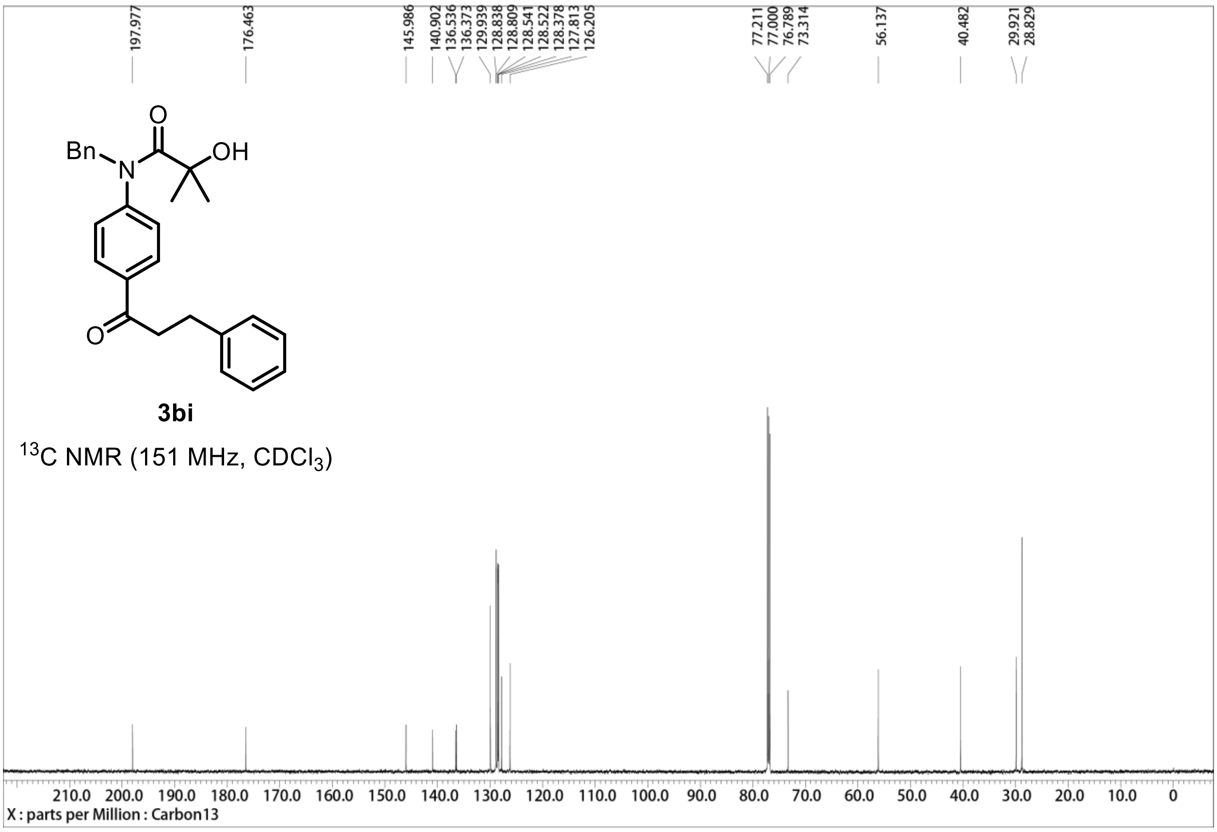

Bn
N
O
OH
O
3bi
^{13}C NMR (151 MHz, CDCl$_3$)
X : parts per Million : Carbon13

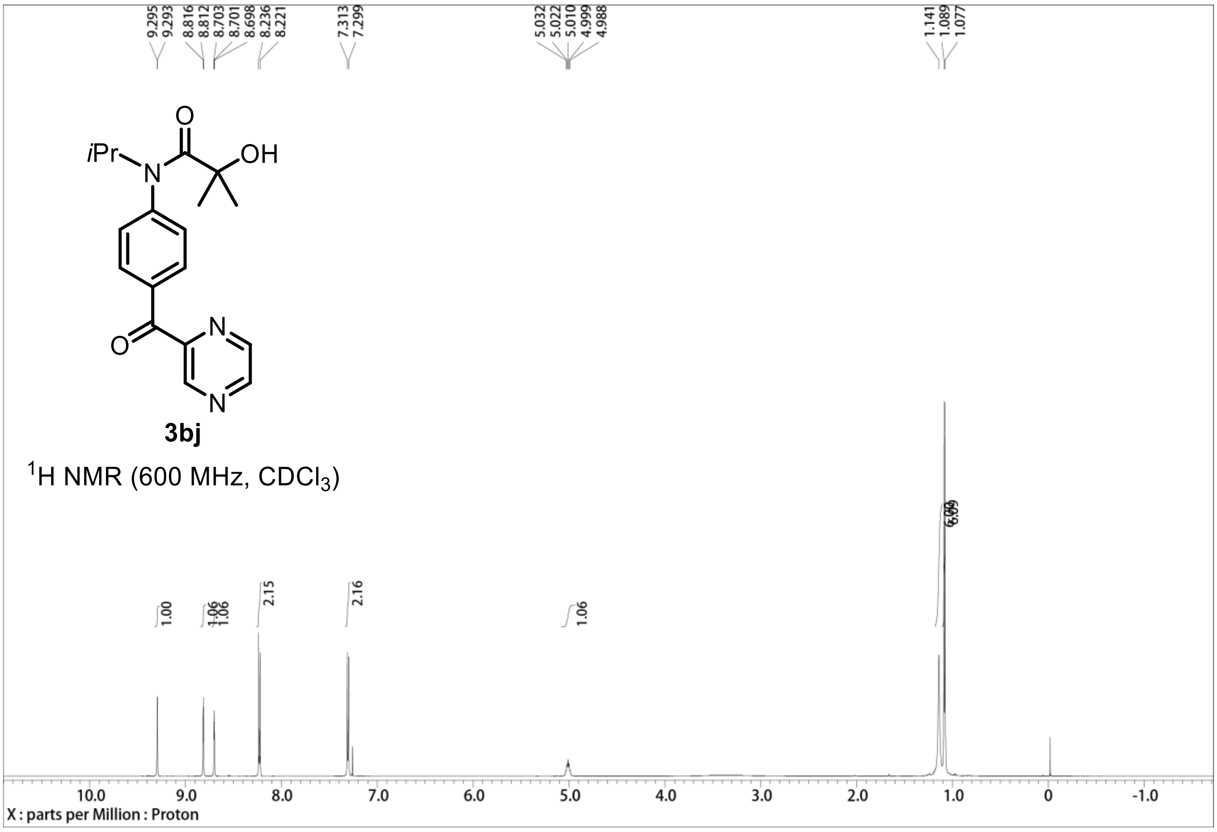
9.295
9.293
8.816
8.812
8.703
8.701
8.698
8.236
8.221
7.313
7.299
5.032
5.022
5.010
4.999
4.988
1.141
1.089
1.077
iPr
N
O
OH
O
N
N
3bj
1H NMR (600 MHz, CDCl3)
1.00
1.06
1.06
2.15
2.16
1.06
10.0
9.0
8.0
7.0
6.0
5.0
4.0
3.0
2.0
1.0
0
-1.0
X : parts per Million : Proton

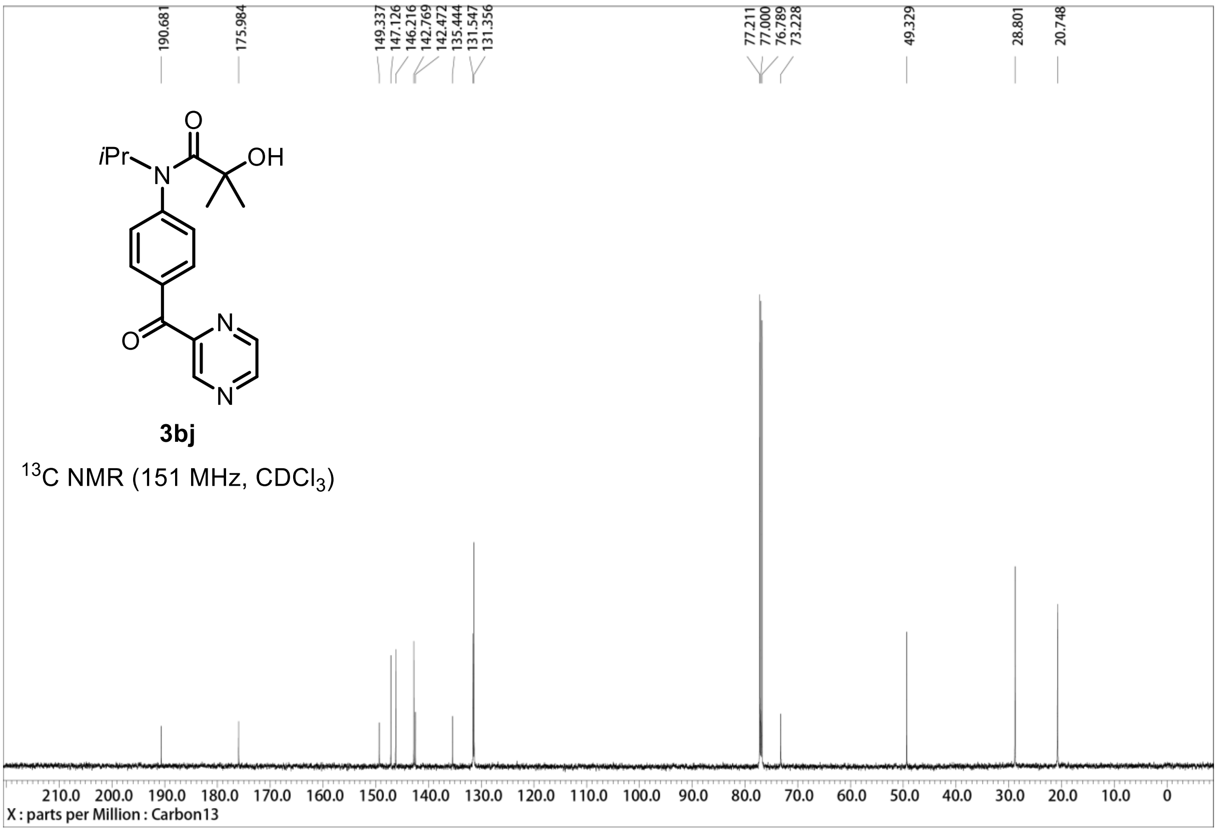
190.681
175.984
149.337
147.126
146.216
142.769
142.472
135.444
131.547
131.356
77.211
77.000
76.789
73.228
49.329
28.801
20.748
iPr
N
O
OH
O
N
N
3bj
13C NMR (151 MHz, CDCl3)
210.0
200.0
190.0
180.0
170.0
160.0
150.0
140.0
130.0
120.0
110.0
100.0
90.0
80.0
70.0
60.0
50.0
40.0
30.0
20.0
10.0
0
X : parts per Million : Carbon13

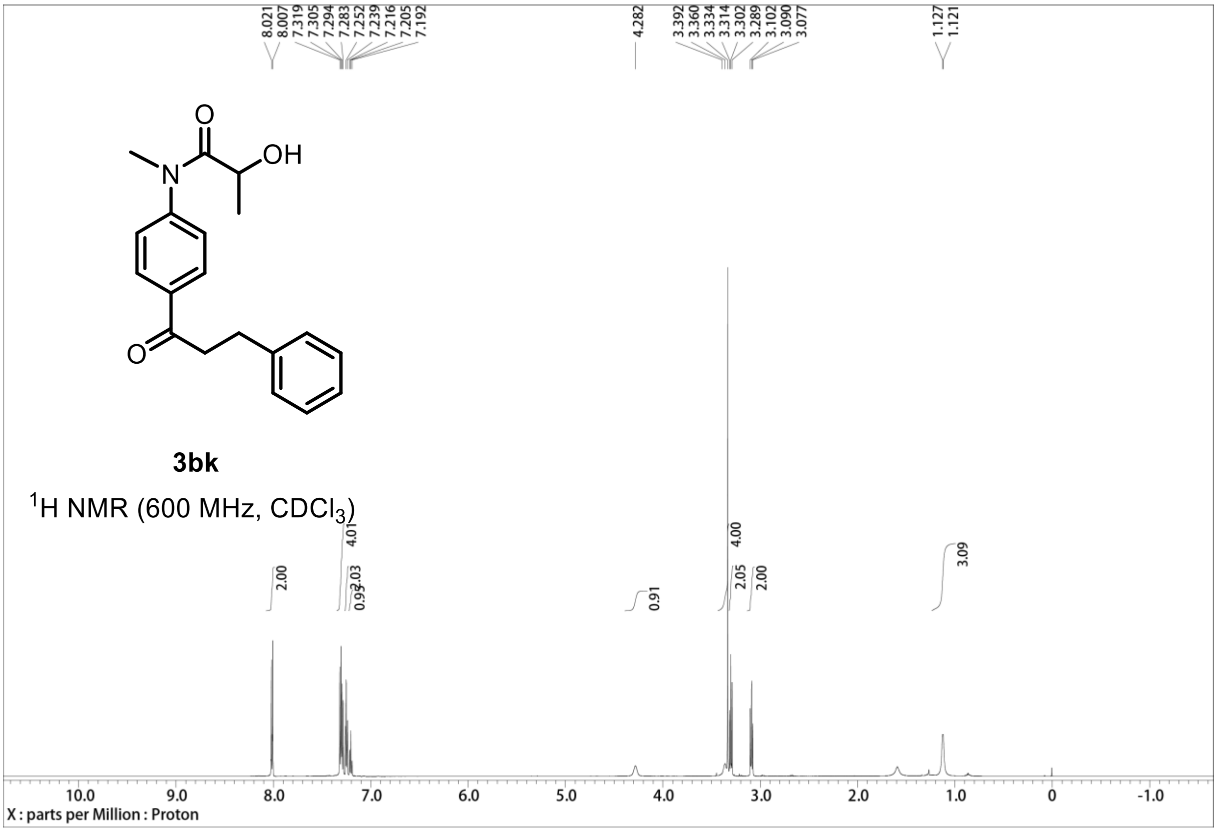

8.021
8.007
7.319
7.305
7.294
7.283
7.252
7.239
7.216
7.205
7.192
4.282
3.392
3.360
3.334
3.314
3.302
3.289
3.102
3.090
3.077
1.127
1.121
O
N
OH
O
3bk
1H NMR (600 MHz, CDCl3)
2.00
4.01
2.03
0.97
0.91
4.00
2.05
2.00
3.09
10.0 9.0 8.0 7.0 6.0 5.0 4.0 3.0 2.0 1.0 0 -1.0
X : parts per Million : Proton

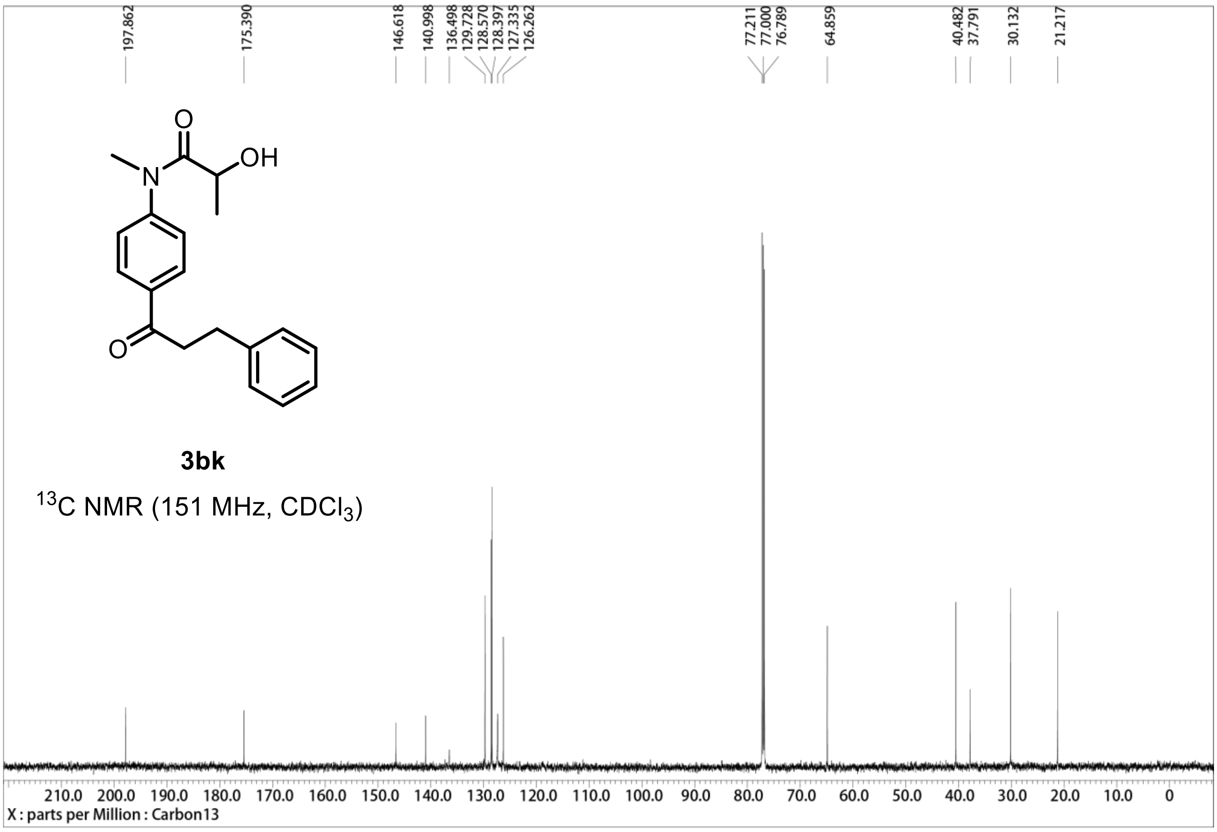

197.862
175.390
146.618
140.998
136.498
129.728
128.570
128.397
127.335
126.262
77.211
77.000
76.789
64.859
40.482
37.791
30.132
21.217
O
N
OH
O
3bk
13C NMR (151 MHz, CDCl3)
210.0 200.0 190.0 180.0 170.0 160.0 150.0 140.0 130.0 120.0 110.0 100.0 90.0 80.0 70.0 60.0 50.0 40.0 30.0 20.0 10.0 0
X : parts per Million : Carbon13

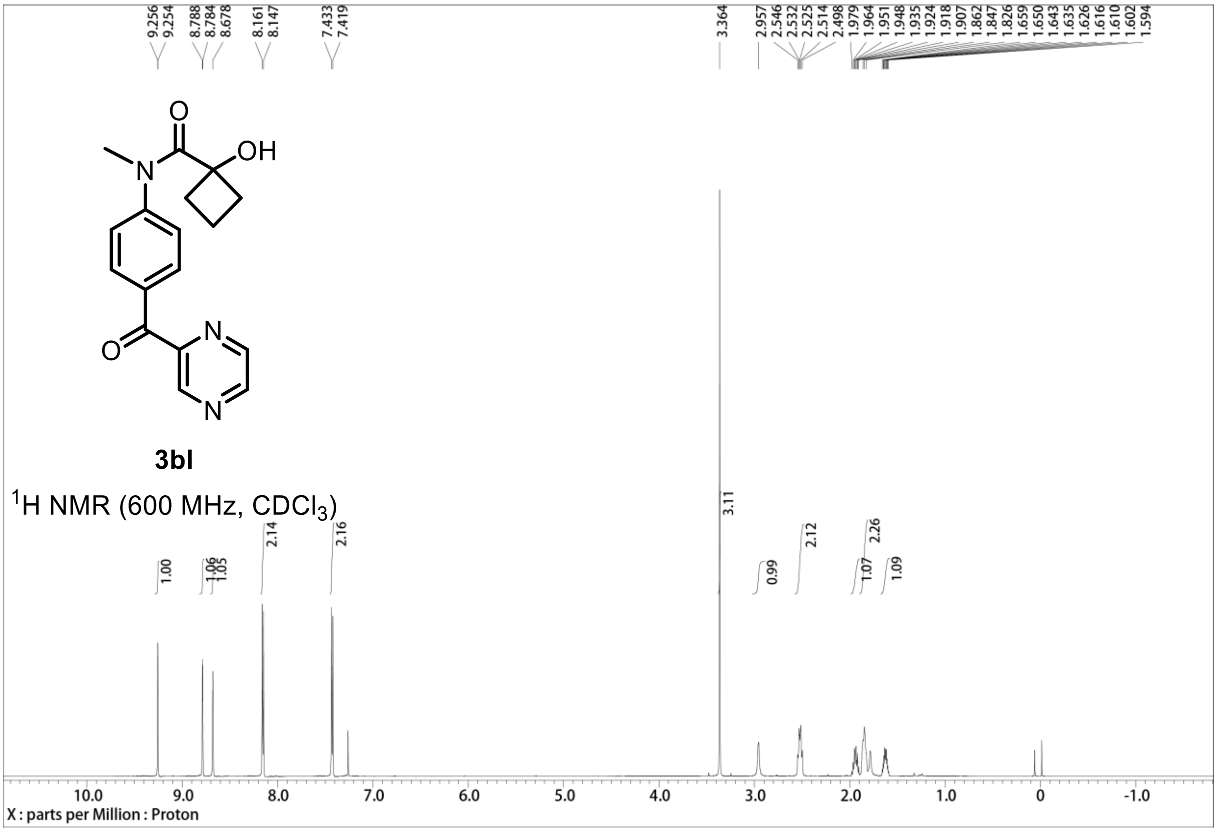
9.256 9.254 8.788 8.784 8.678 8.161 8.147 7.433 7.419
3.364 2.957 2.546 2.532 2.525 2.514 2.498 1.979 1.964 1.951 1.948 1.935 1.924 1.918 1.907 1.862 1.847 1.826 1.659 1.650 1.643 1.635 1.626 1.616 1.610 1.602 1.594
O
N
OH
O
N
N
3bl
1H NMR (600 MHz, CDCl3)
1.00 1.06 1.05 2.14 2.16 3.11 0.99 2.12 1.07 2.26 1.09
10.0 9.0 8.0 7.0 6.0 5.0 4.0 3.0 2.0 1.0 0 -1.0
X : parts per Million : Proton

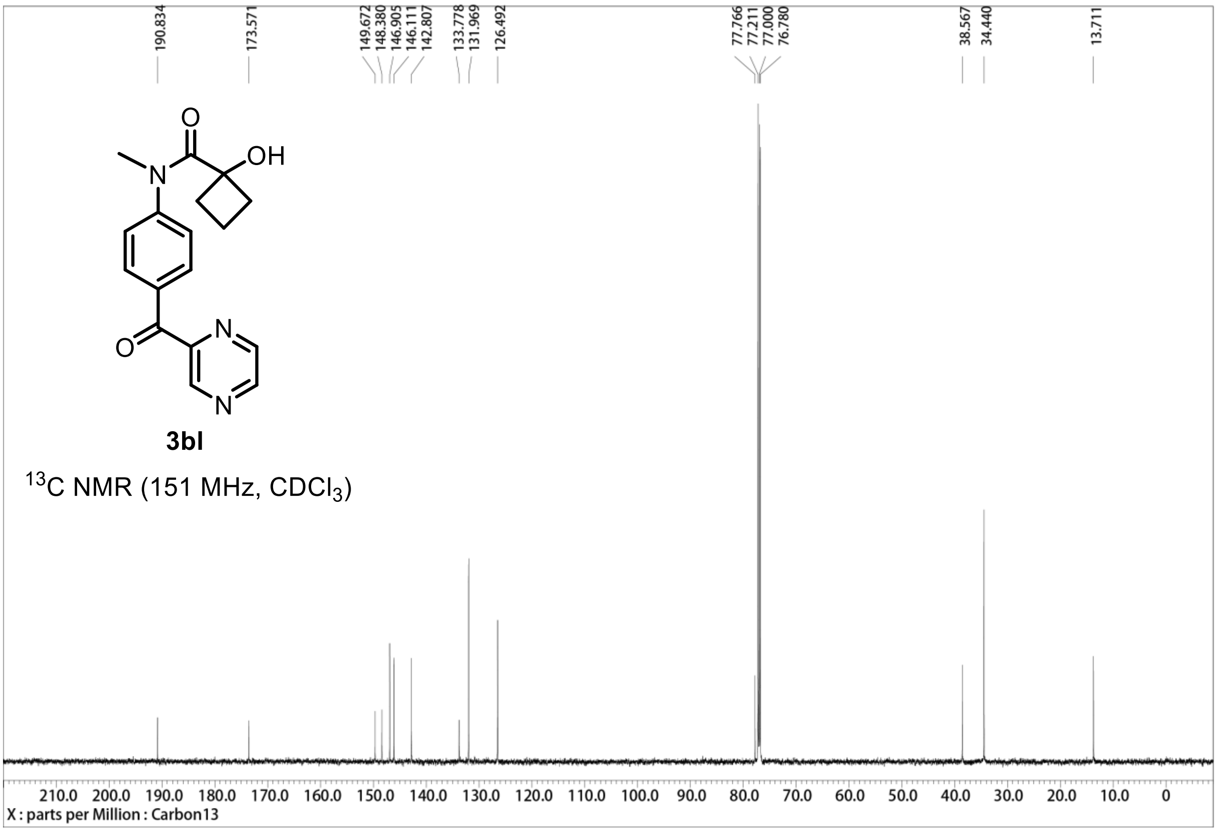
190.834 173.571 149.672 148.380 146.905 146.111 142.807 133.778 131.969 126.492 77.766 77.211 77.000 76.780 38.567 34.440 13.711
O
N
OH
O
N
N
3bl
13C NMR (151 MHz, CDCl3)
210.0 200.0 190.0 180.0 170.0 160.0 150.0 140.0 130.0 120.0 110.0 100.0 90.0 80.0 70.0 60.0 50.0 40.0 30.0 20.0 10.0 0
X : parts per Million : Carbon13

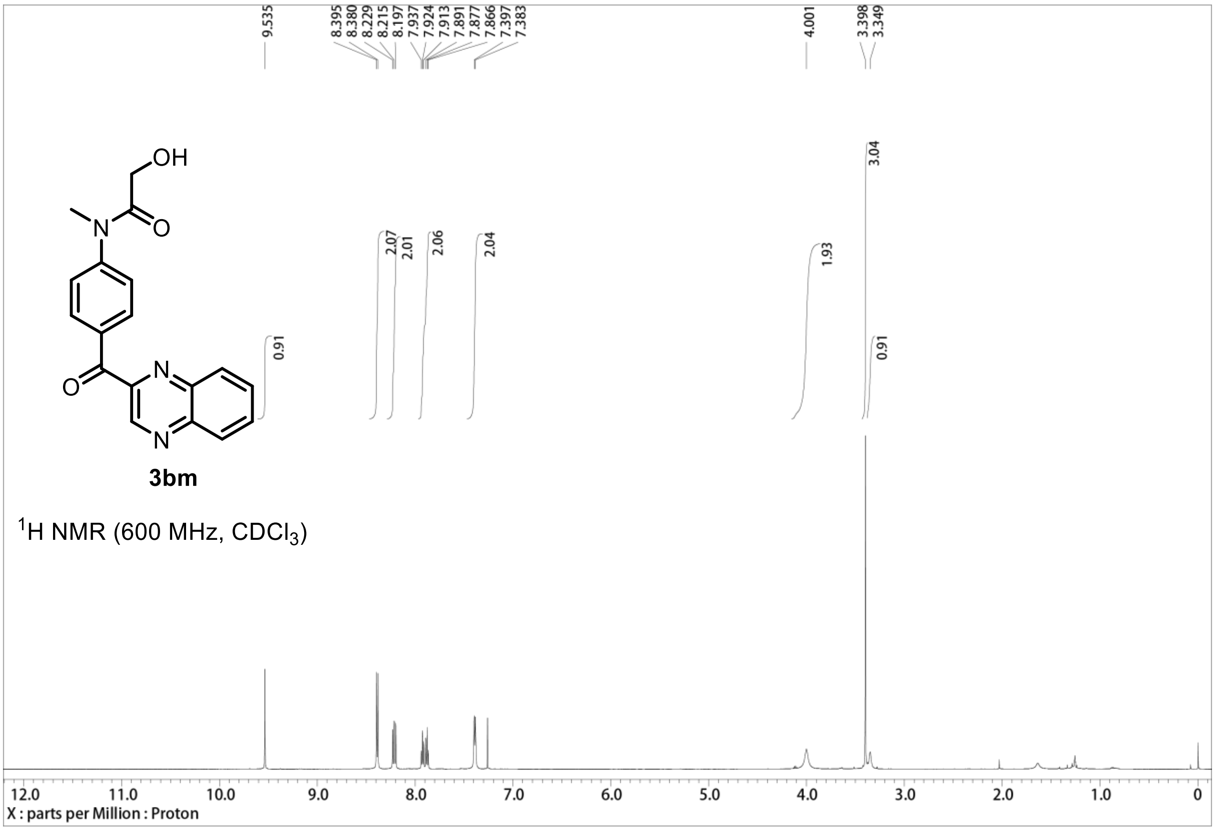
9.535
8.395
8.380
8.229
8.215
8.197
7.937
7.924
7.913
7.891
7.877
7.866
7.397
7.383
4.001
3.398
3.349
3.04
2.07
2.01
2.06
2.04
1.93
0.91
0.91
OH
N
O
O
N
N
3bm
^{1}H NMR (600 MHz, CDCl$_3$)
12.0
11.0
10.0
9.0
8.0
7.0
6.0
5.0
4.0
3.0
2.0
1.0
0
X : parts per Million : Proton

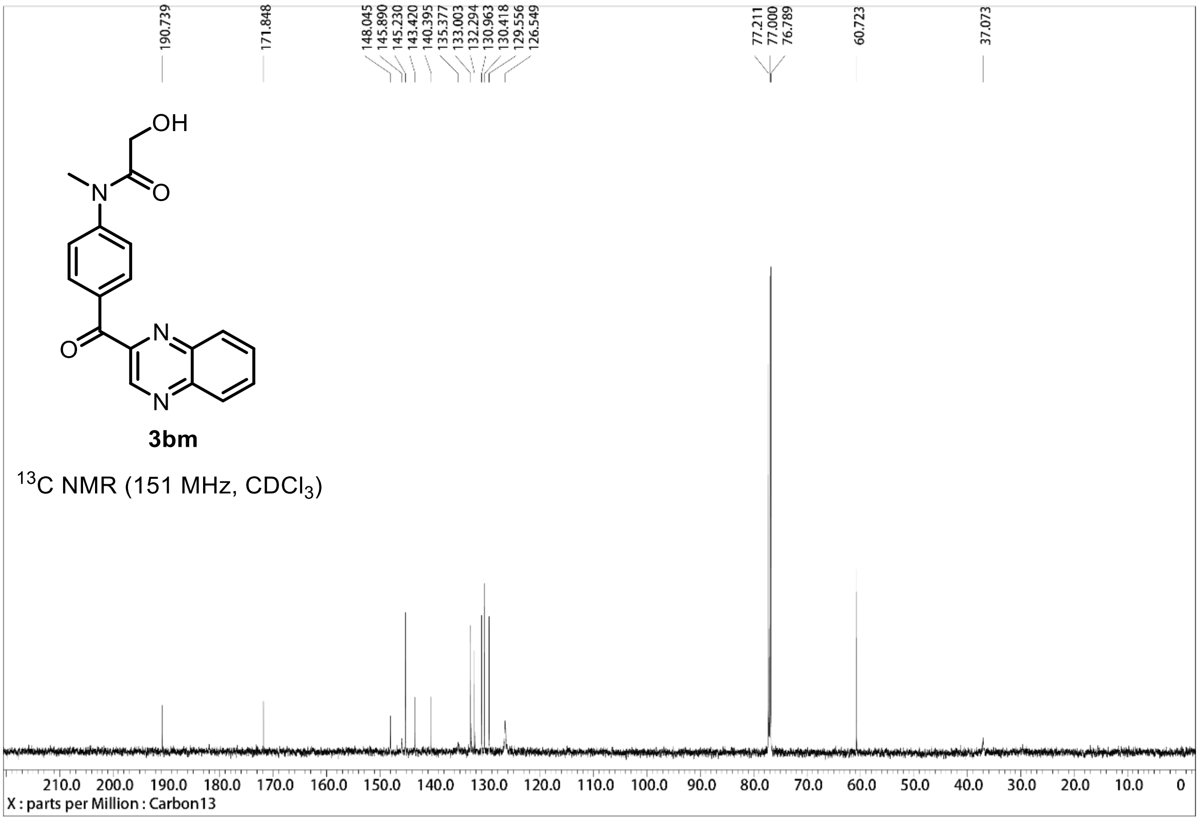
190.739
171.848
148.045
145.890
145.230
143.420
140.395
135.377
133.003
132.294
130.963
130.418
129.556
126.549
77.211
77.000
76.789
60.723
37.073
OH
N
O
O
N
N
3bm
^{13}C NMR (151 MHz, CDCl$_3$)
210.0
200.0
190.0
180.0
170.0
160.0
150.0
140.0
130.0
120.0
110.0
100.0
90.0
80.0
70.0
60.0
50.0
40.0
30.0
20.0
10.0
0
X : parts per Million : Carbon13

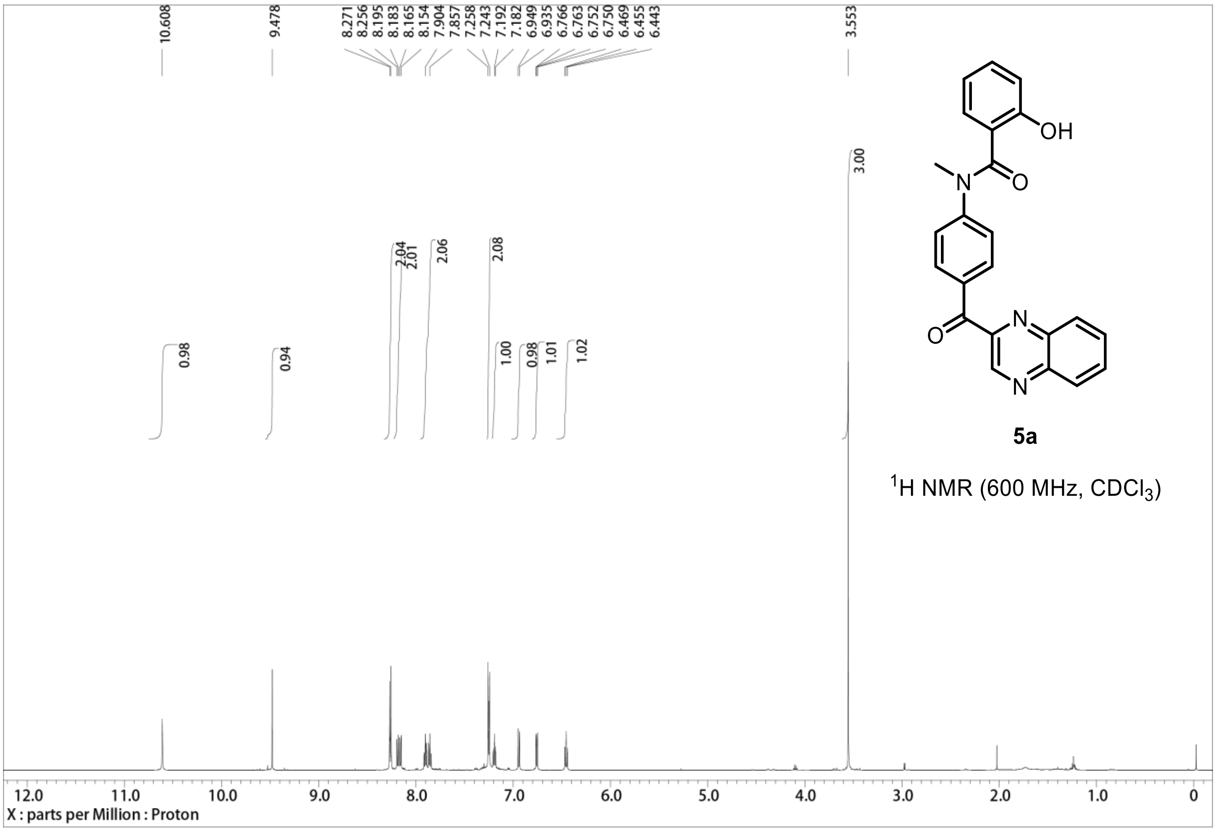
10.608
9.478
8.271 8.256 8.195 8.183 8.165 8.154 7.904 7.857 7.258 7.243 7.192 7.182 6.949 6.935 6.766 6.763 6.752 6.750 6.469 6.455 6.443
3.553
0.98 0.94 2.04 2.01 2.06 2.08 1.00 0.98 1.01 1.02 3.00
OH
N
O
O
N
N
5a
1H NMR (600 MHz, CDCl3)
12.0 11.0 10.0 9.0 8.0 7.0 6.0 5.0 4.0 3.0 2.0 1.0 0
X : parts per Million : Proton

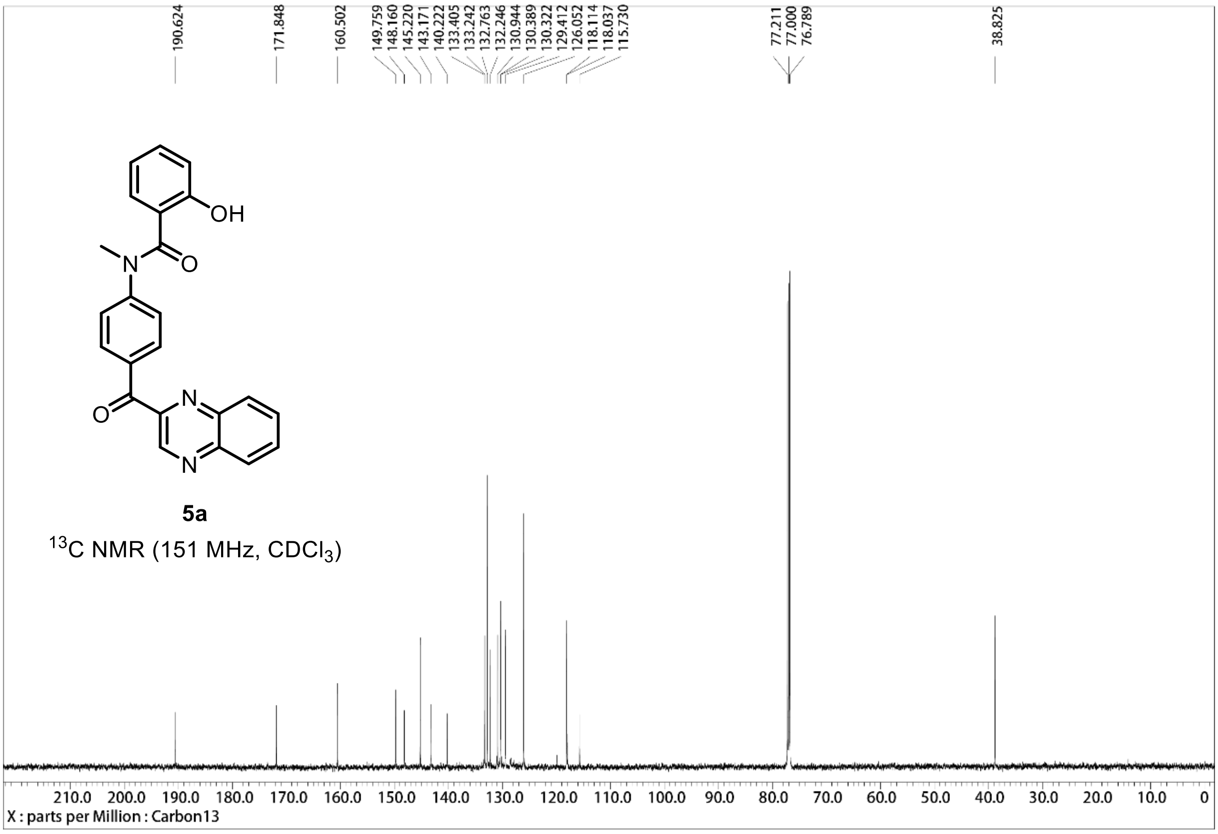
190.624
171.848
160.502
149.759 148.160 145.220 143.171 140.222 133.405 133.242 132.763 132.246 130.944 130.389 130.322 129.412 126.052 118.114 118.037 115.730
77.211 77.000 76.789
38.825
OH
N
O
O
N
N
5a
13C NMR (151 MHz, CDCl3)
210.0 200.0 190.0 180.0 170.0 160.0 150.0 140.0 130.0 120.0 110.0 100.0 90.0 80.0 70.0 60.0 50.0 40.0 30.0 20.0 10.0 0
X : parts per Million : Carbon13

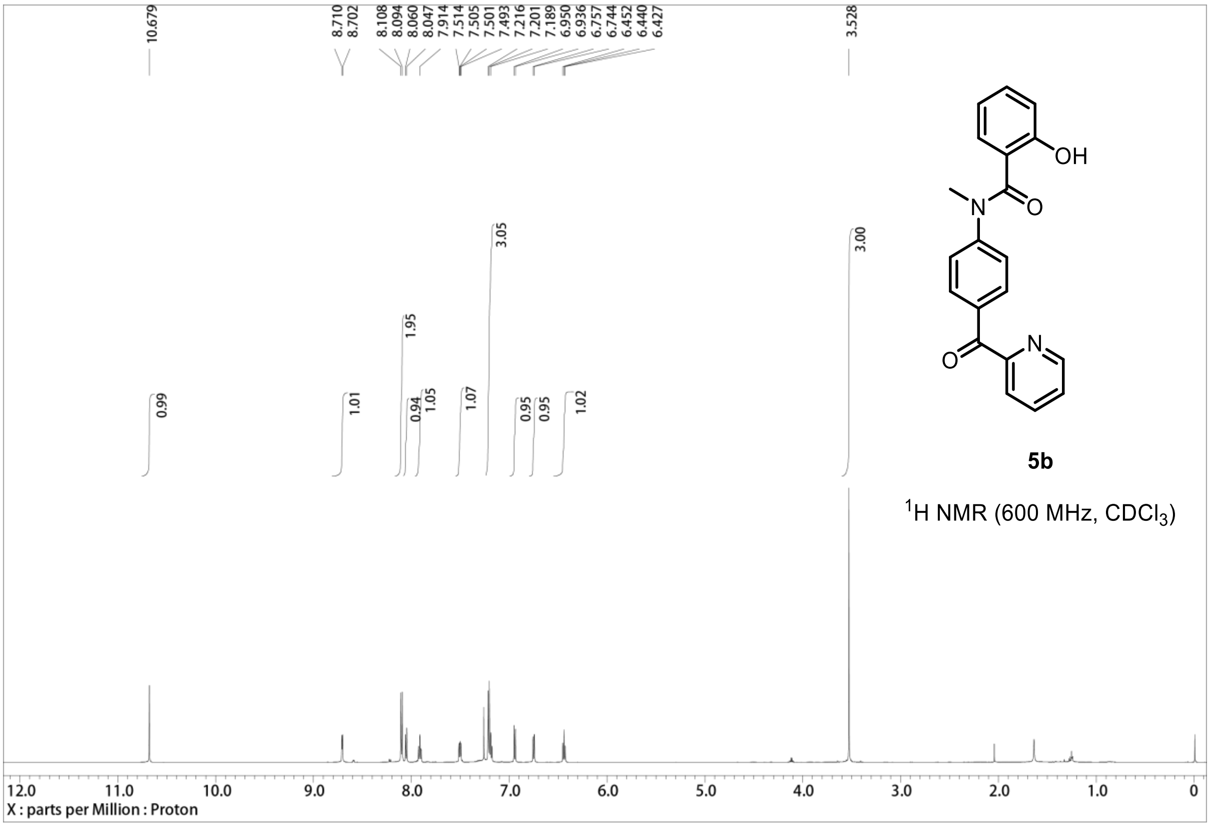
10.679
8.710
8.702
8.108
8.094
8.060
8.047
7.914
7.514
7.505
7.501
7.493
7.216
7.201
7.189
6.950
6.936
6.757
6.744
6.452
6.440
6.427
3.528
0.99
1.01
1.95
0.94
1.05
1.07
3.05
0.95
0.95
1.02
3.00
OH
N
O
N
O
5b
1H NMR (600 MHz, CDCl3)
12.0 11.0 10.0 9.0 8.0 7.0 6.0 5.0 4.0 3.0 2.0 1.0 0
X : parts per Million : Proton

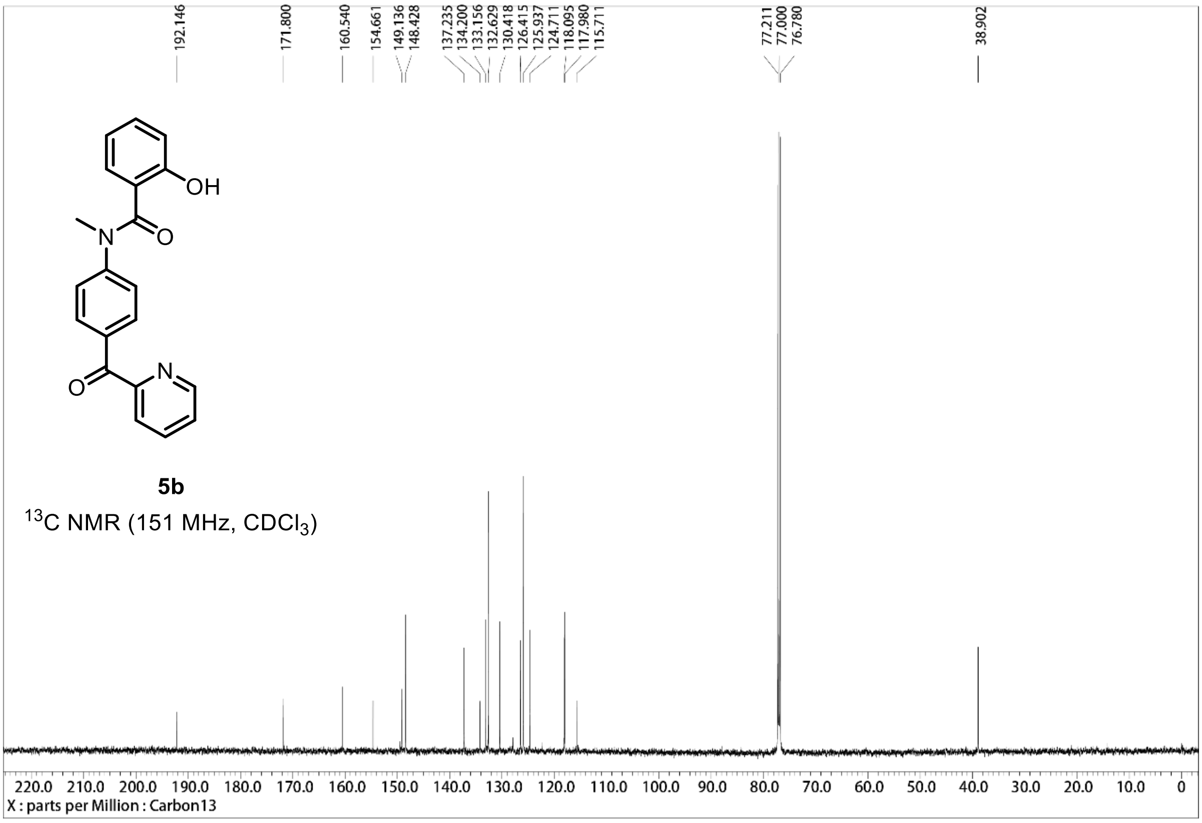
192.146
171.800
160.540
154.661
149.136
148.428
137.235
134.200
133.156
132.629
130.418
126.415
125.937
124.711
118.095
117.980
115.711
77.211
77.000
76.780
38.902
OH
N
O
N
O
5b
13C NMR (151 MHz, CDCl3)
220.0 210.0 200.0 190.0 180.0 170.0 160.0 150.0 140.0 130.0 120.0 110.0 100.0 90.0 80.0 70.0 60.0 50.0 40.0 30.0 20.0 10.0 0
X : parts per Million : Carbon13

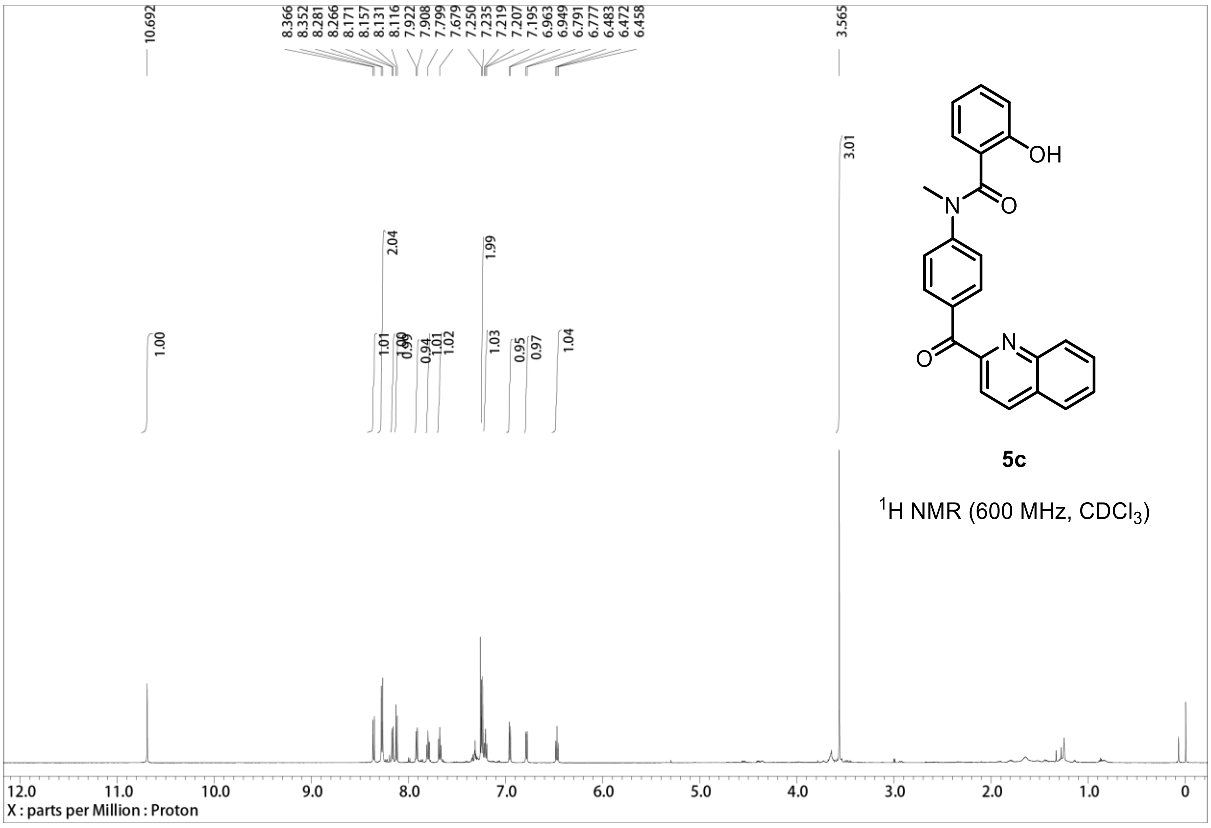
10.692
8.366 8.352 8.281 8.266 8.171 8.157 8.131 8.116 7.922 7.908 7.799 7.679 7.250 7.235 7.219 7.207 7.195 6.963 6.949 6.791 6.777 6.483 6.472 6.458
3.565
3.01
2.04
1.99
1.00
1.01 1.00 0.99 0.94 1.01 1.02
1.03 0.95 0.97 1.04
OH
N
O
N
O
5c
^{1}H NMR (600 MHz, CDCl$_3$)
12.0 11.0 10.0 9.0 8.0 7.0 6.0 5.0 4.0 3.0 2.0 1.0 0
X : parts per Million : Proton

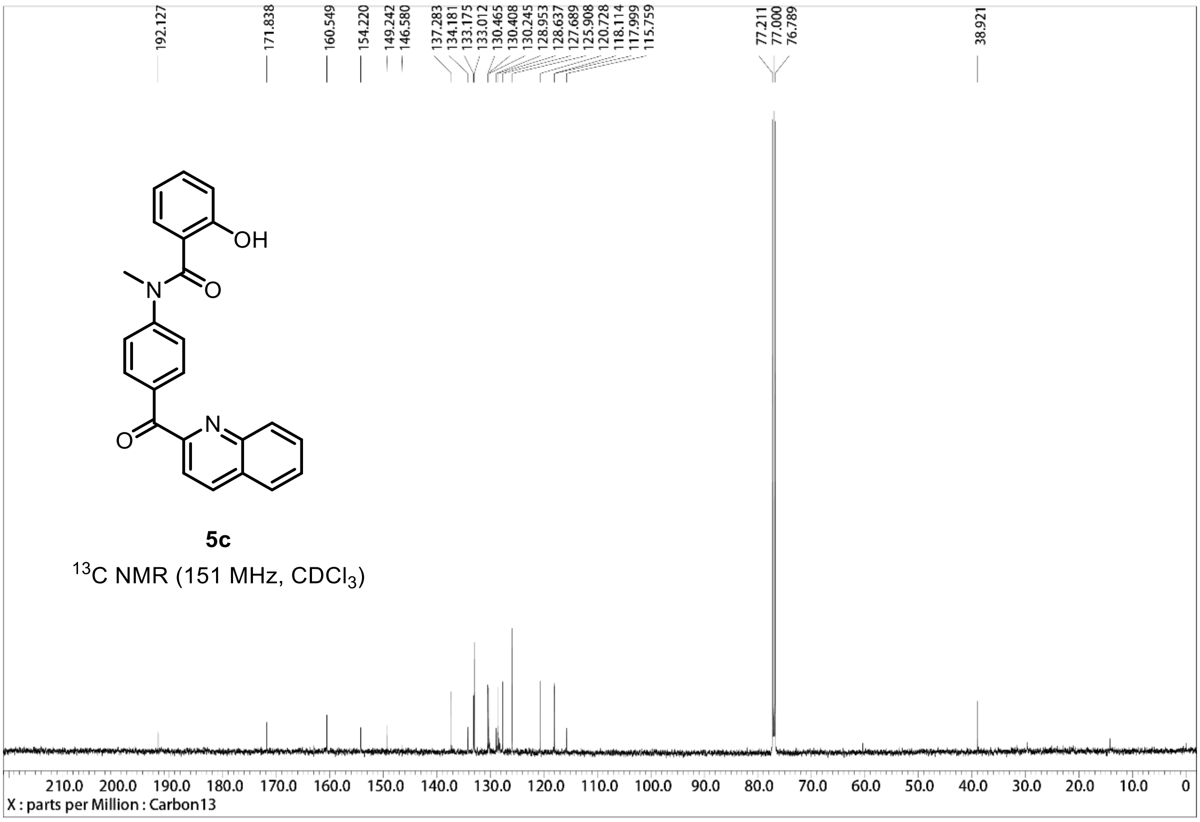
192.127
171.838
160.549
154.220
149.242 146.580
137.283 134.181 133.175 133.012 130.465 130.408 130.245 128.953 128.637 127.689 125.908 120.728 118.114 117.999 115.759
77.211 77.000 76.789
38.921
OH
N
O
N
O
5c
^{13}C NMR (151 MHz, CDCl$_3$)
210.0 200.0 190.0 180.0 170.0 160.0 150.0 140.0 130.0 120.0 110.0 100.0 90.0 80.0 70.0 60.0 50.0 40.0 30.0 20.0 10.0 0
X : parts per Million : Carbon13

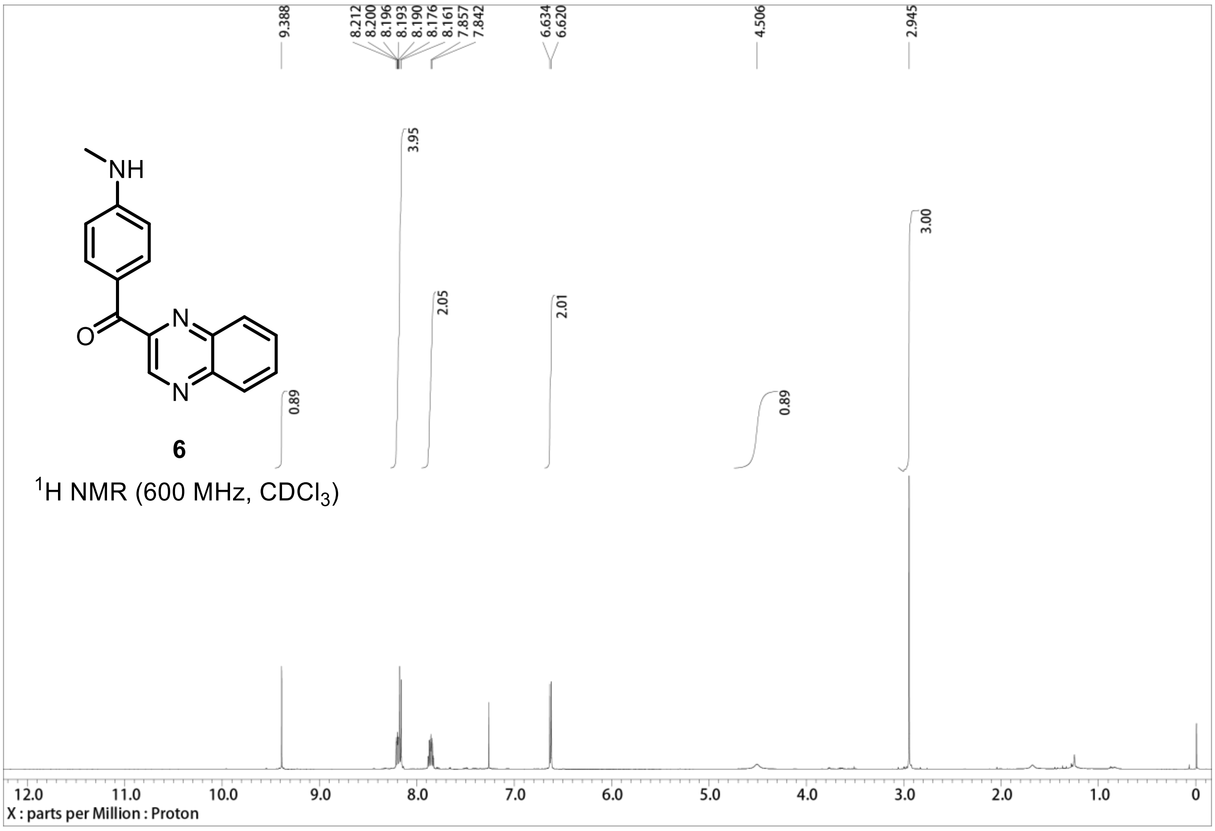

6

^{1}H NMR (600 MHz, $CDCl_3$)

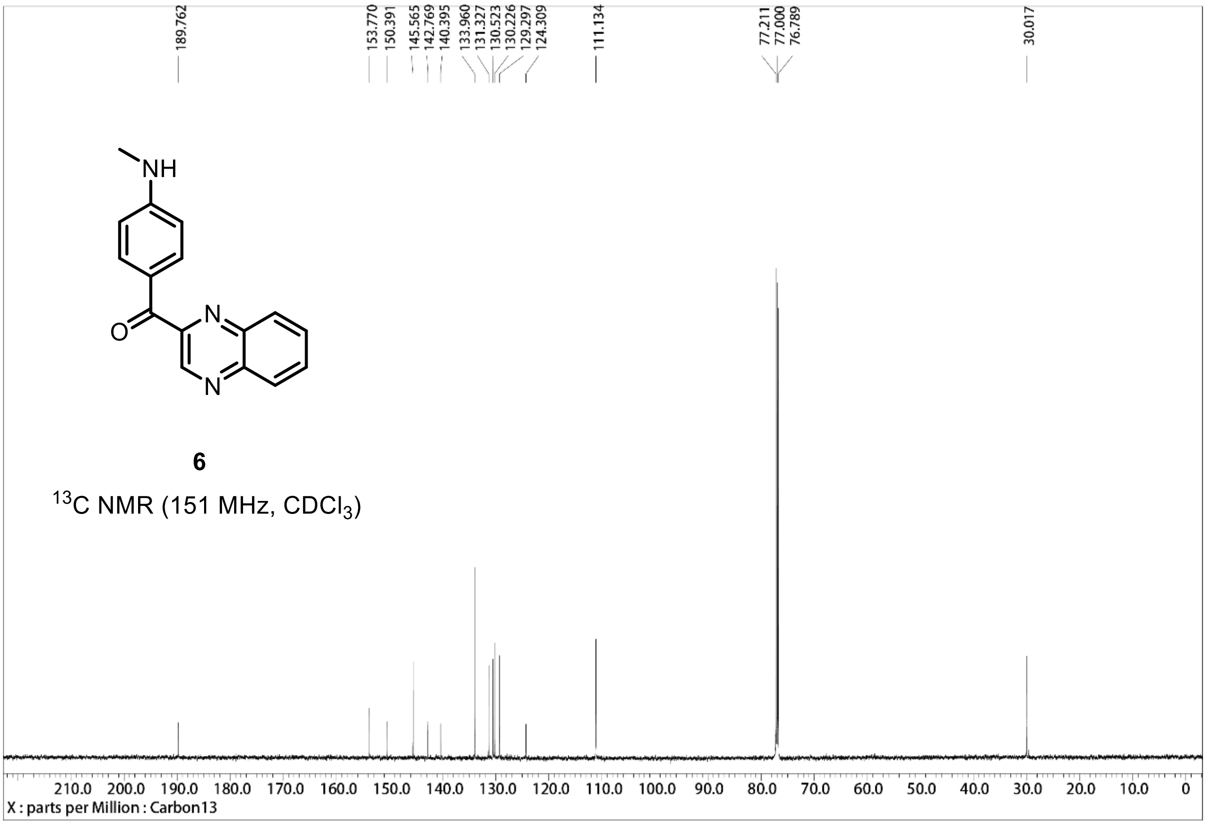

6

^{13}C NMR (151 MHz, $CDCl_3$)

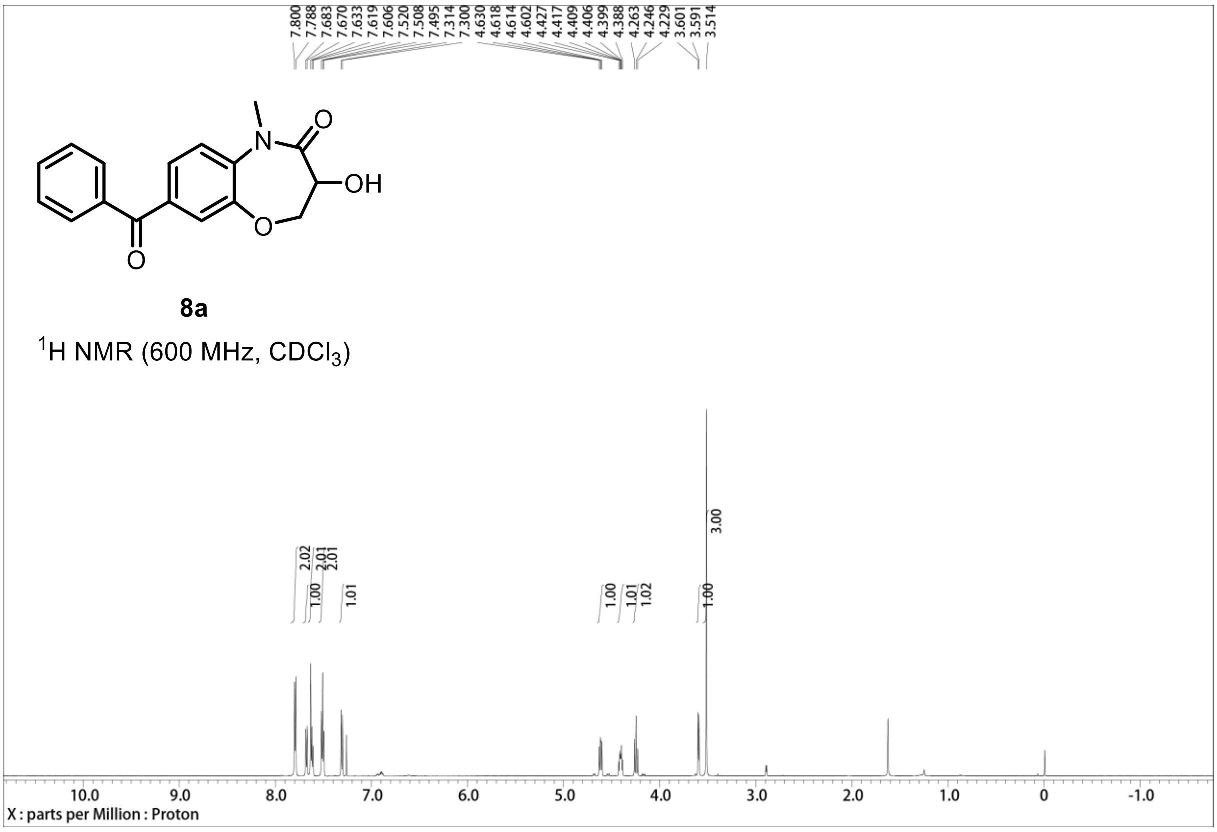
7.800
7.788
7.683
7.670
7.633
7.619
7.606
7.520
7.508
7.495
7.314
7.300
4.630
4.618
4.614
4.602
4.427
4.417
4.409
4.406
4.399
4.388
4.263
4.246
4.229
3.601
3.591
3.514
N
O
OH
O
O
8a
1H NMR (600 MHz, CDCl3)
2.02
1.00
2.01
2.01
1.01
1.00
1.01
1.02
1.00
3.00
10.0
9.0
8.0
7.0
6.0
5.0
4.0
3.0
2.0
1.0
0
-1.0
X : parts per Million : Proton

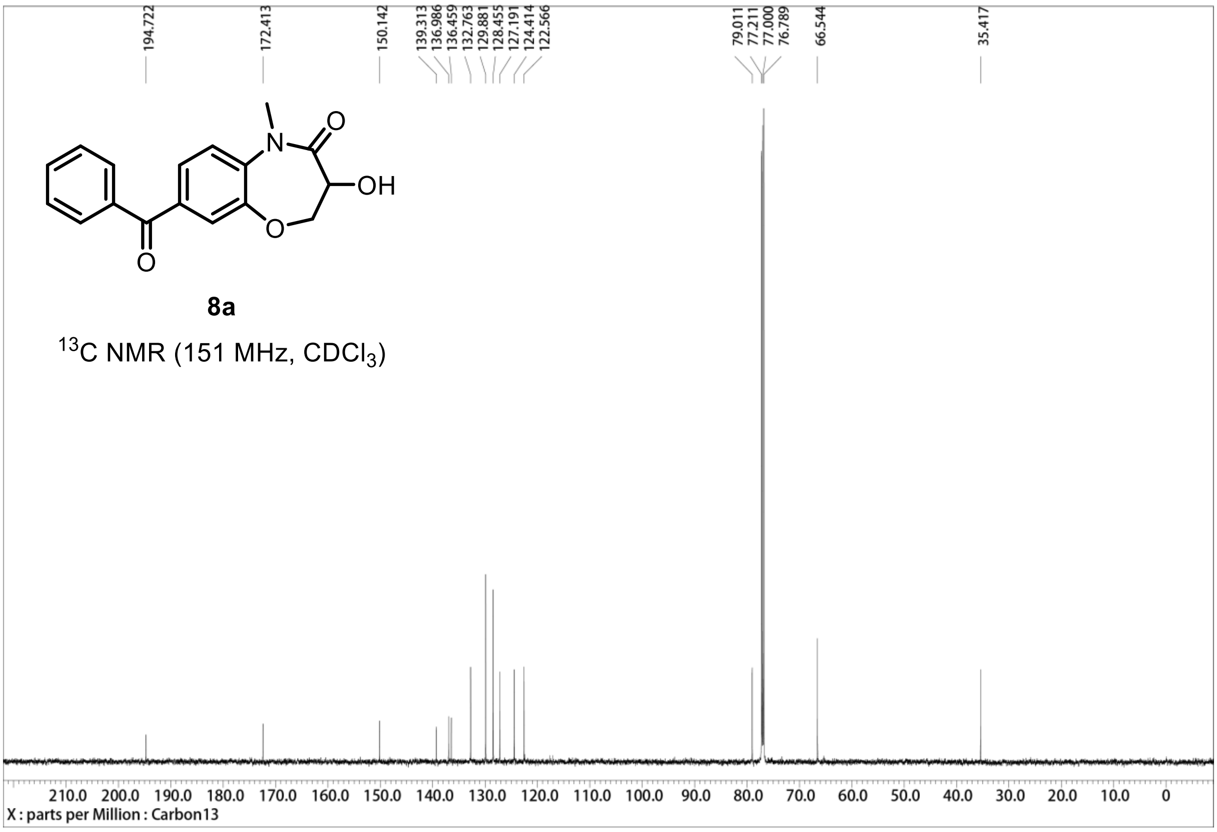
194.722
172.413
150.142
139.313
136.986
136.459
132.763
129.881
128.455
127.191
124.414
122.566
79.011
77.211
77.000
76.789
66.544
35.417
N
O
OH
O
O
8a
13C NMR (151 MHz, CDCl3)
210.0
200.0
190.0
180.0
170.0
160.0
150.0
140.0
130.0
120.0
110.0
100.0
90.0
80.0
70.0
60.0
50.0
40.0
30.0
20.0
10.0
0
X : parts per Million : Carbon13

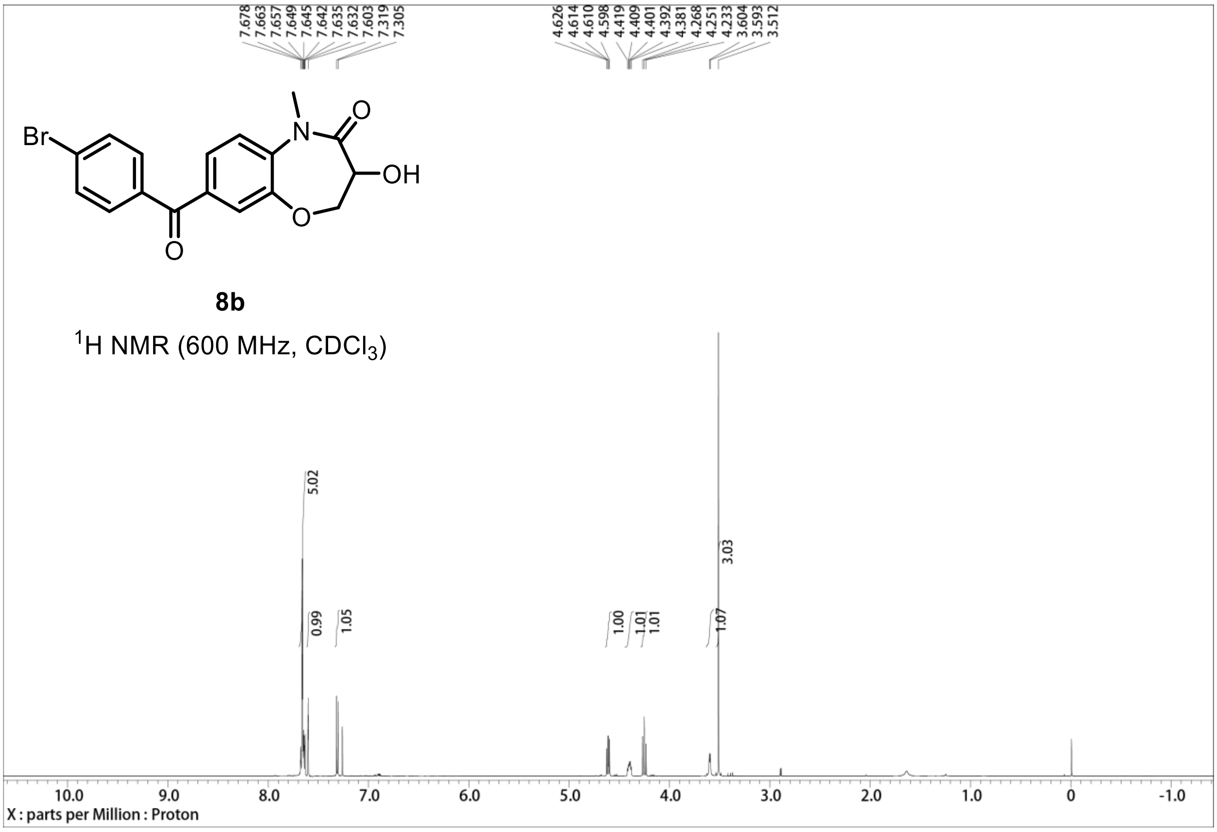

7.678
7.663
7.657
7.649
7.645
7.642
7.635
7.632
7.603
7.319
7.305
4.626
4.614
4.610
4.598
4.419
4.409
4.401
4.392
4.381
4.268
4.251
4.233
3.604
3.593
3.512
Br
N
O
OH
O
O
8b
¹H NMR (600 MHz, CDCl₃)
5.02
0.99
1.05
1.00
1.01
1.01
1.07
3.03
10.0 9.0 8.0 7.0 6.0 5.0 4.0 3.0 2.0 1.0 0 -1.0
X : parts per Million : Proton

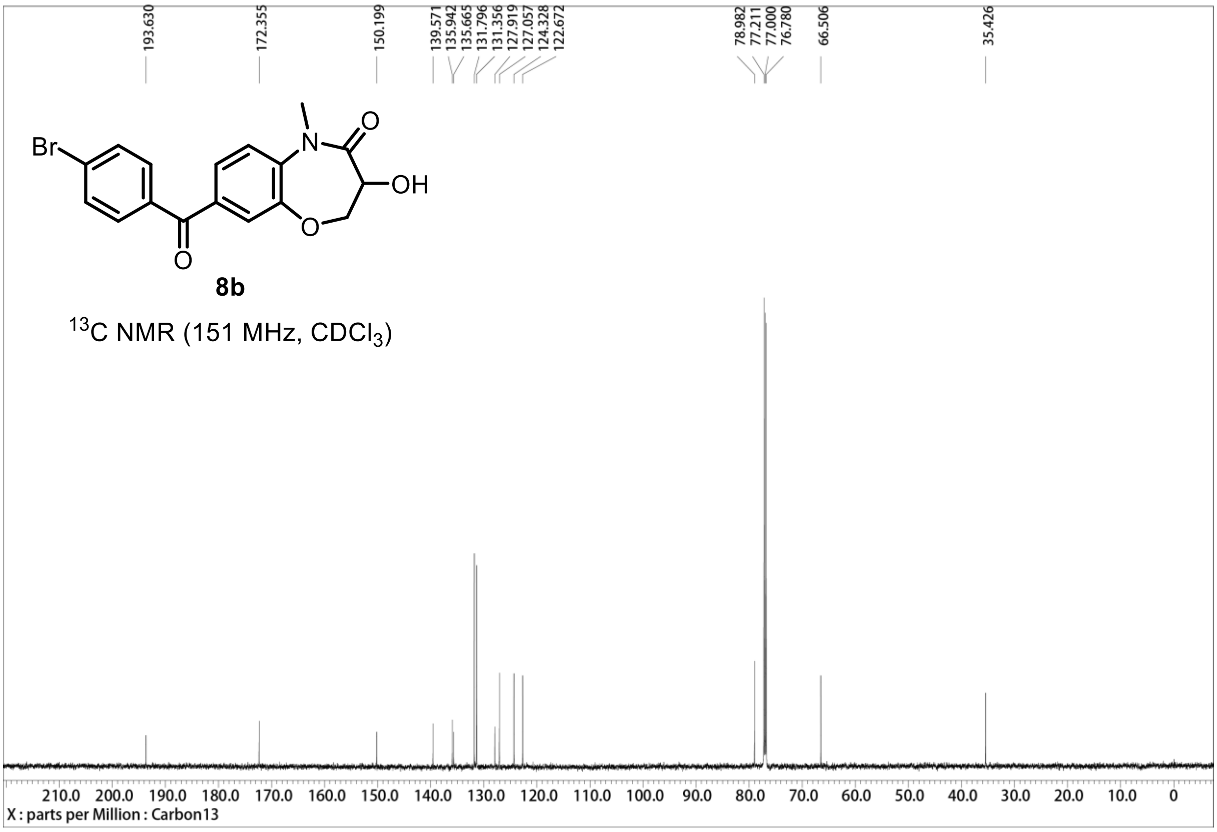

193.630
172.355
150.199
139.571
135.942
135.665
131.796
131.356
127.919
127.057
124.328
122.672
78.982
77.211
77.000
76.780
66.506
35.426
Br
N
O
OH
O
O
8b
¹³C NMR (151 MHz, CDCl₃)
210.0 200.0 190.0 180.0 170.0 160.0 150.0 140.0 130.0 120.0 110.0 100.0 90.0 80.0 70.0 60.0 50.0 40.0 30.0 20.0 10.0 0
X : parts per Million : Carbon13

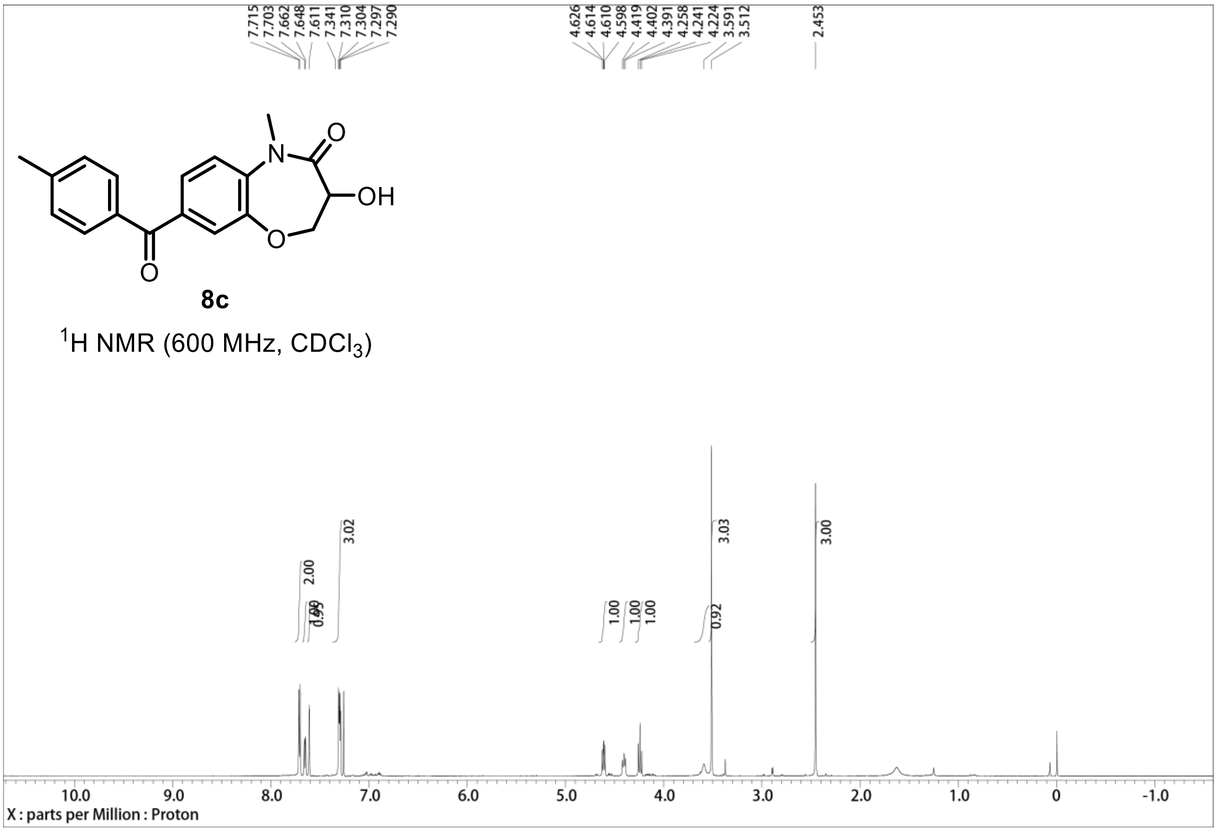
7.715 7.703 7.662 7.648 7.611 7.341 7.310 7.304 7.297 7.290
4.626 4.614 4.610 4.598 4.419 4.402 4.391 4.258 4.241 4.224 3.591 3.512
2.453
N
O
OH
O
O
8c
^{1}H NMR (600 MHz, $CDCl_3$)
X : parts per Million : Proton

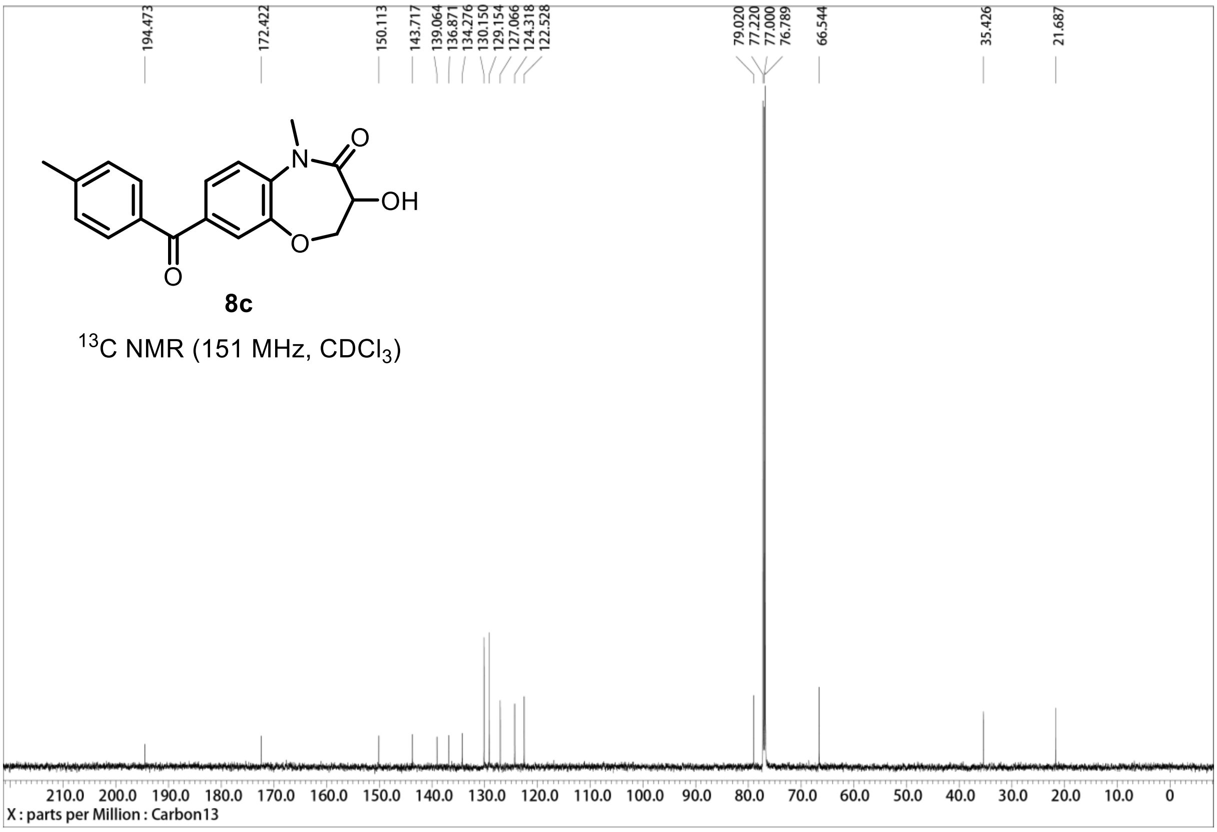
194.473
172.422
150.113 143.717 139.064 136.871 134.276 130.150 129.154 127.066 124.318 122.528
79.020 77.220 77.000 76.789
66.544
35.426
21.687
N
O
OH
O
O
8c
^{13}C NMR (151 MHz, $CDCl_3$)
X : parts per Million : Carbon13

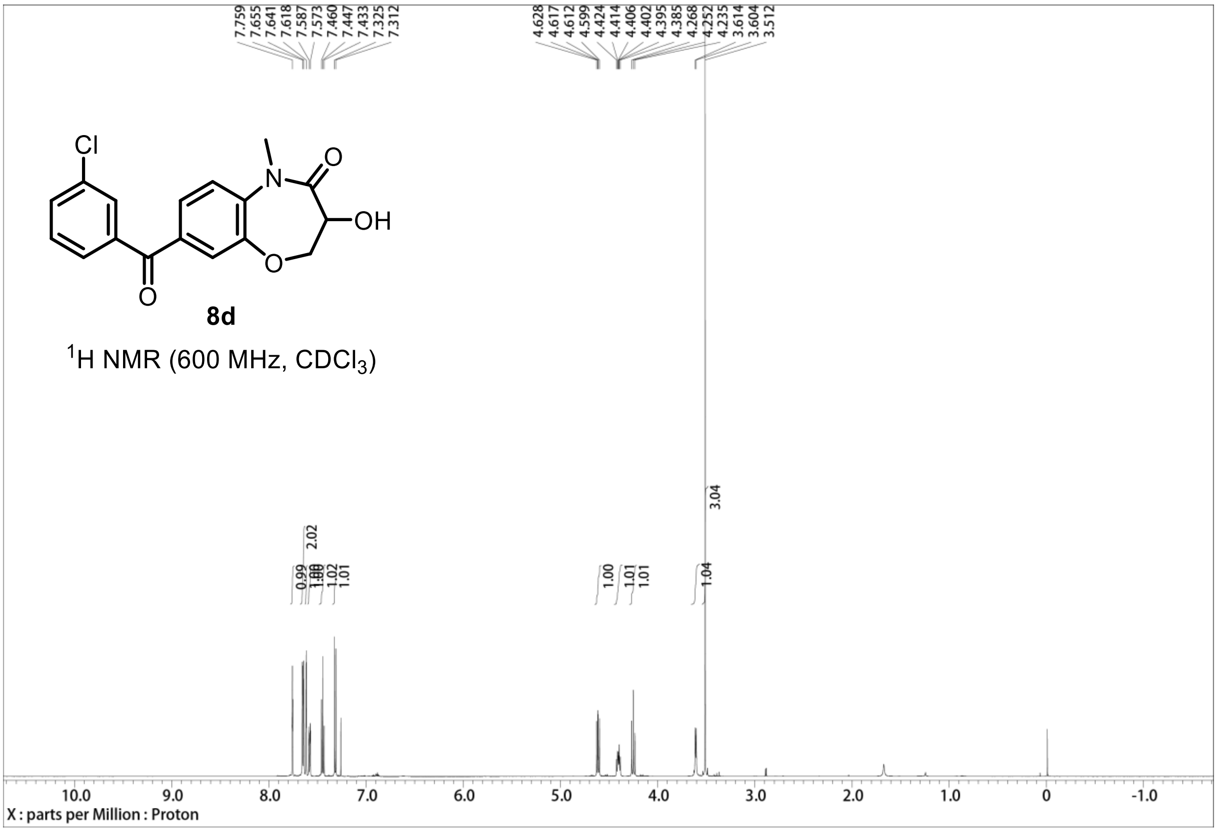
7.759
7.655
7.641
7.618
7.587
7.573
7.460
7.447
7.433
7.325
7.312
4.628
4.617
4.612
4.599
4.424
4.414
4.406
4.402
4.395
4.385
4.268
4.252
4.235
3.614
3.604
3.512
Cl
N
O
OH
O
O
8d
¹H NMR (600 MHz, CDCl3)
3.04
2.02
0.99
1.00
1.02
1.01
1.00
1.01
1.01
1.04
10.0
9.0
8.0
7.0
6.0
5.0
4.0
3.0
2.0
1.0
0
-1.0
X : parts per Million : Proton

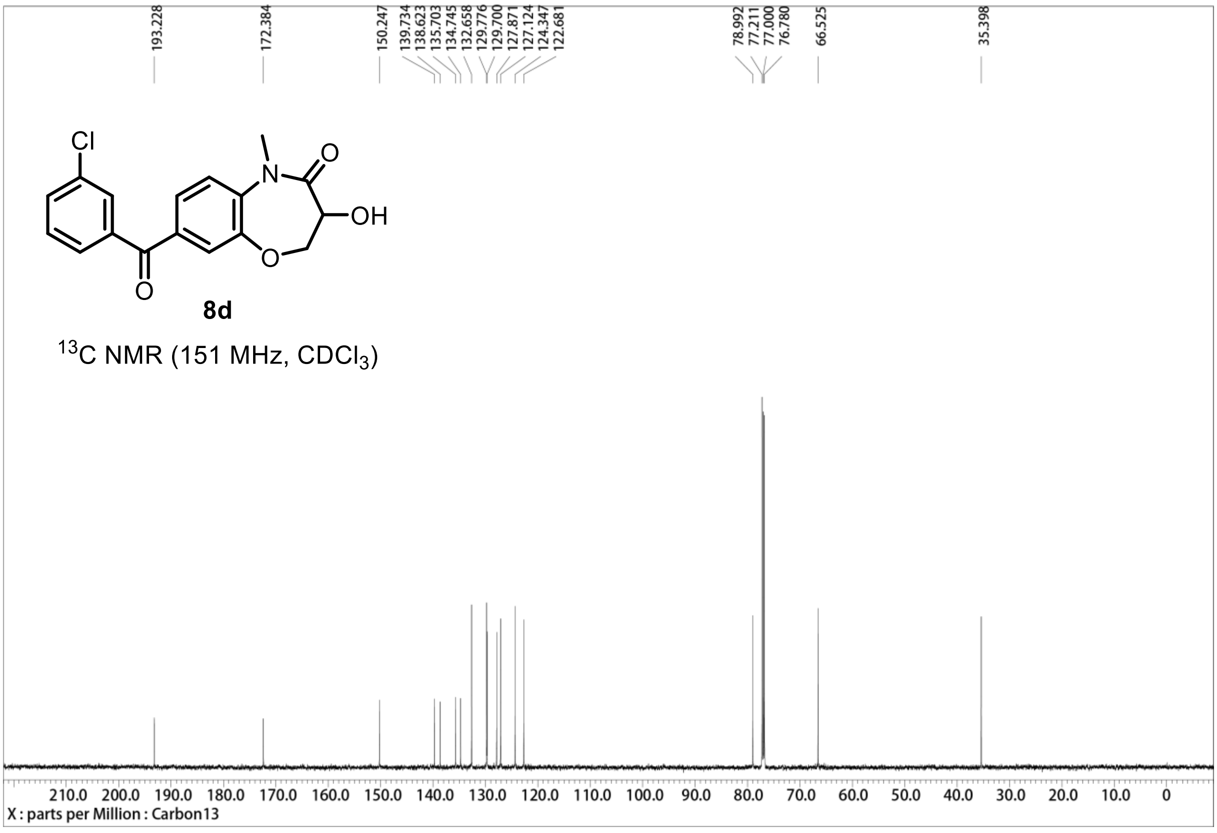
193.228
172.384
150.247
139.734
138.623
135.703
134.745
132.658
129.776
129.700
127.871
127.124
124.347
122.681
78.992
77.211
77.000
76.780
66.525
35.398
Cl
N
O
OH
O
O
8d
¹³C NMR (151 MHz, CDCl3)
210.0 200.0 190.0 180.0 170.0 160.0 150.0 140.0 130.0 120.0 110.0 100.0 90.0 80.0 70.0 60.0 50.0 40.0 30.0 20.0 10.0 0
X : parts per Million : Carbon13

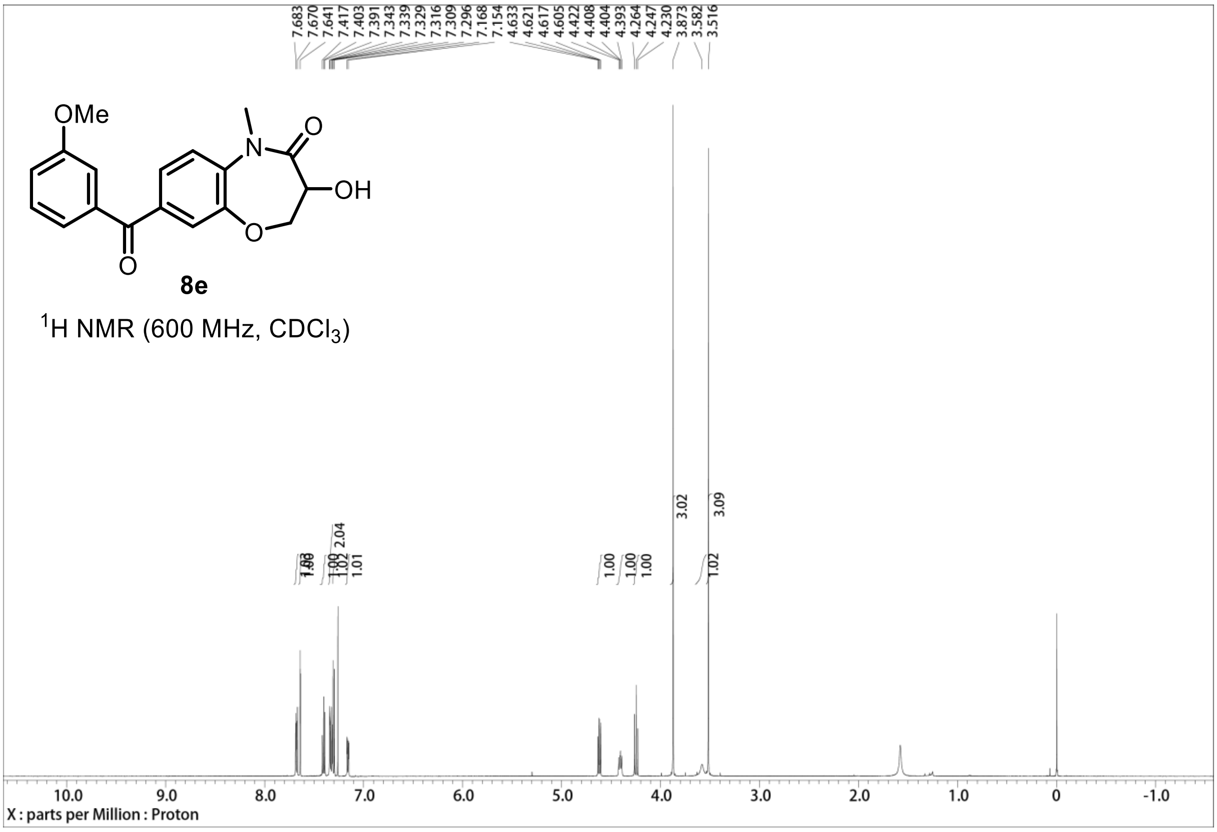
7.683 7.670 7.641 7.417 7.403 7.391 7.343 7.339 7.329 7.316 7.309 7.296 7.168 7.154 4.633 4.621 4.617 4.605 4.422 4.408 4.404 4.393 4.264 4.247 4.230 3.873 3.582 3.516
OMe
N
O
OH
O
O
8e
^{1}H NMR (600 MHz, CDCl$_3$)
3.02
3.09
2.04
1.00
1.00
1.00
1.00
1.02
1.01
10.0 9.0 8.0 7.0 6.0 5.0 4.0 3.0 2.0 1.0 0 -1.0
X : parts per Million : Proton

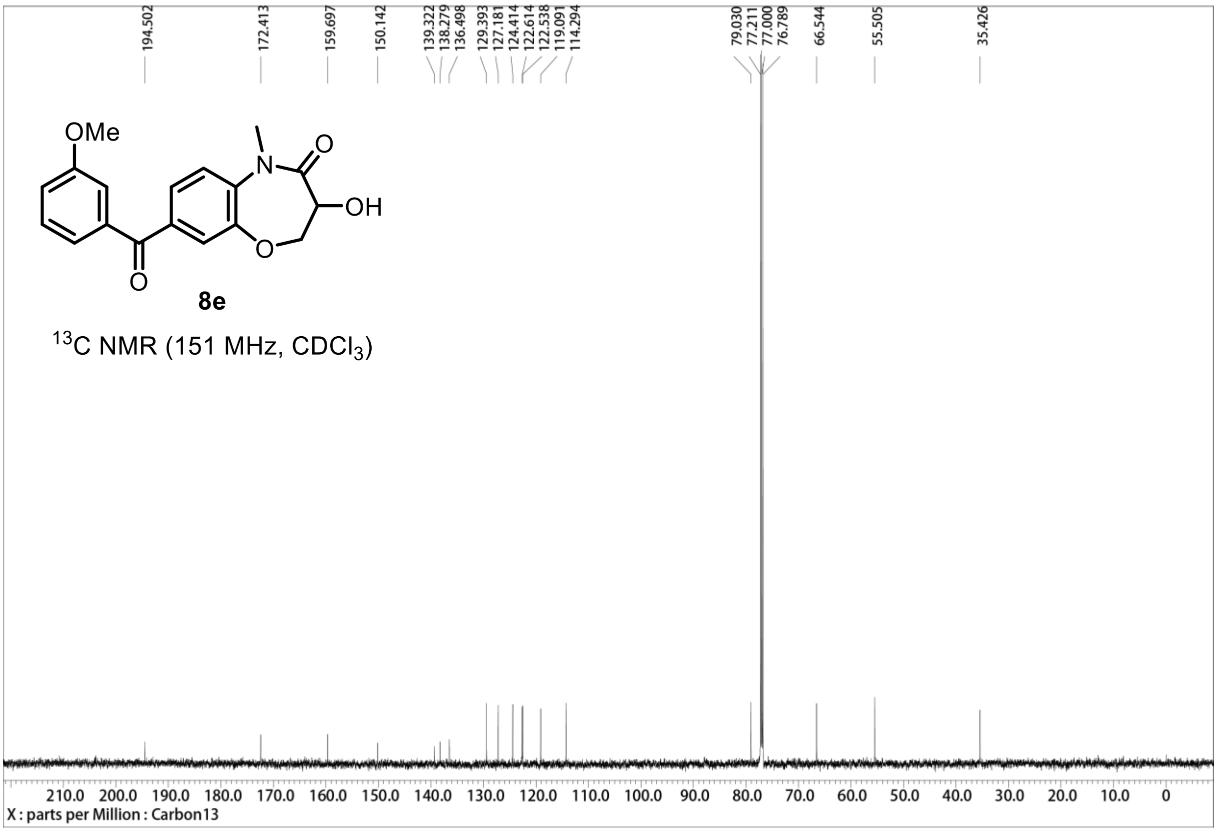
194.502
172.413
159.697
150.142
139.322 138.279 136.498
129.393 127.181 124.414 122.614 122.538 119.091 114.294
79.030 77.211 77.000 76.789
66.544
55.505
35.426
OMe
N
O
OH
O
O
8e
^{13}C NMR (151 MHz, CDCl$_3$)
210.0 200.0 190.0 180.0 170.0 160.0 150.0 140.0 130.0 120.0 110.0 100.0 90.0 80.0 70.0 60.0 50.0 40.0 30.0 20.0 10.0 0
X : parts per Million : Carbon13

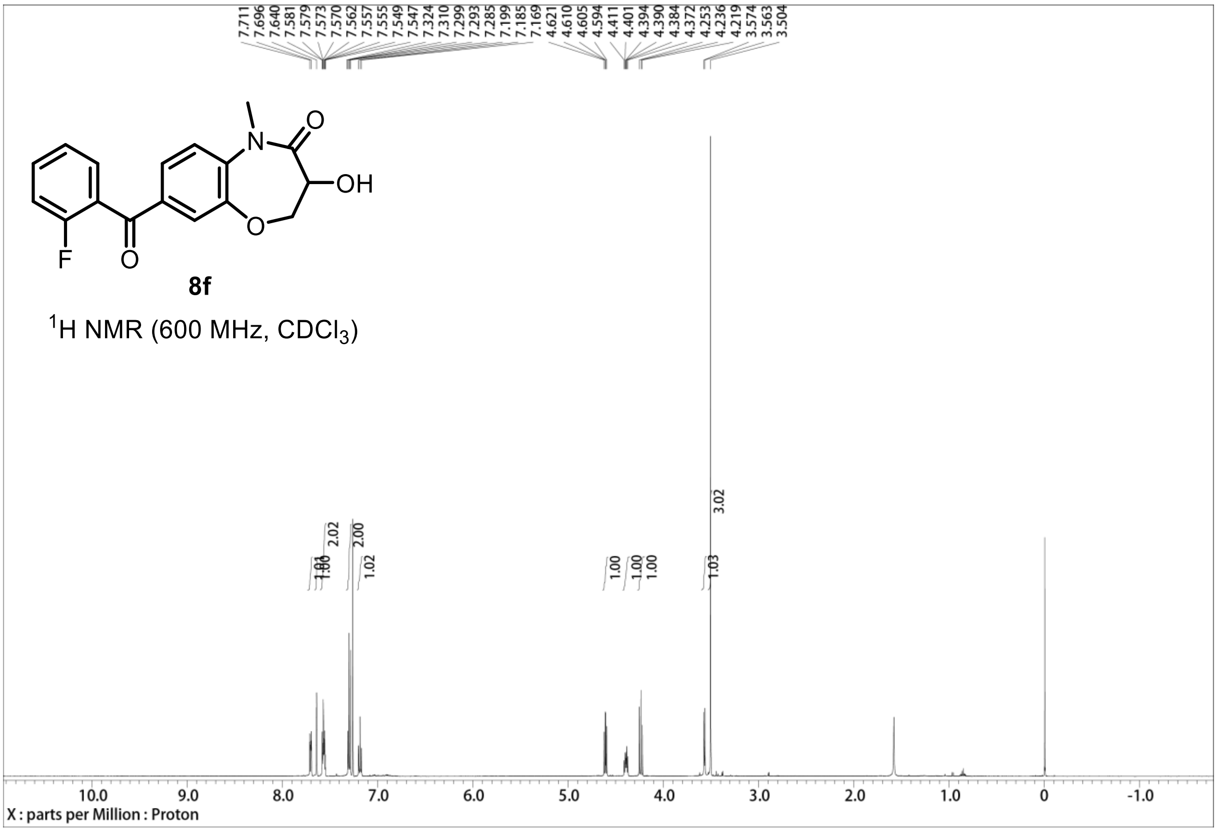
7.711 7.696 7.640 7.581 7.579 7.573 7.570 7.562 7.557 7.555 7.549 7.547 7.324 7.310 7.299 7.293 7.285 7.199 7.185 7.169 4.621 4.610 4.605 4.594 4.411 4.401 4.394 4.390 4.384 4.372 4.253 4.236 4.219 3.574 3.563 3.504
OH
N
O
O
F
O
8f
^{1}H NMR (600 MHz, $CDCl_3$)
X : parts per Million : Proton

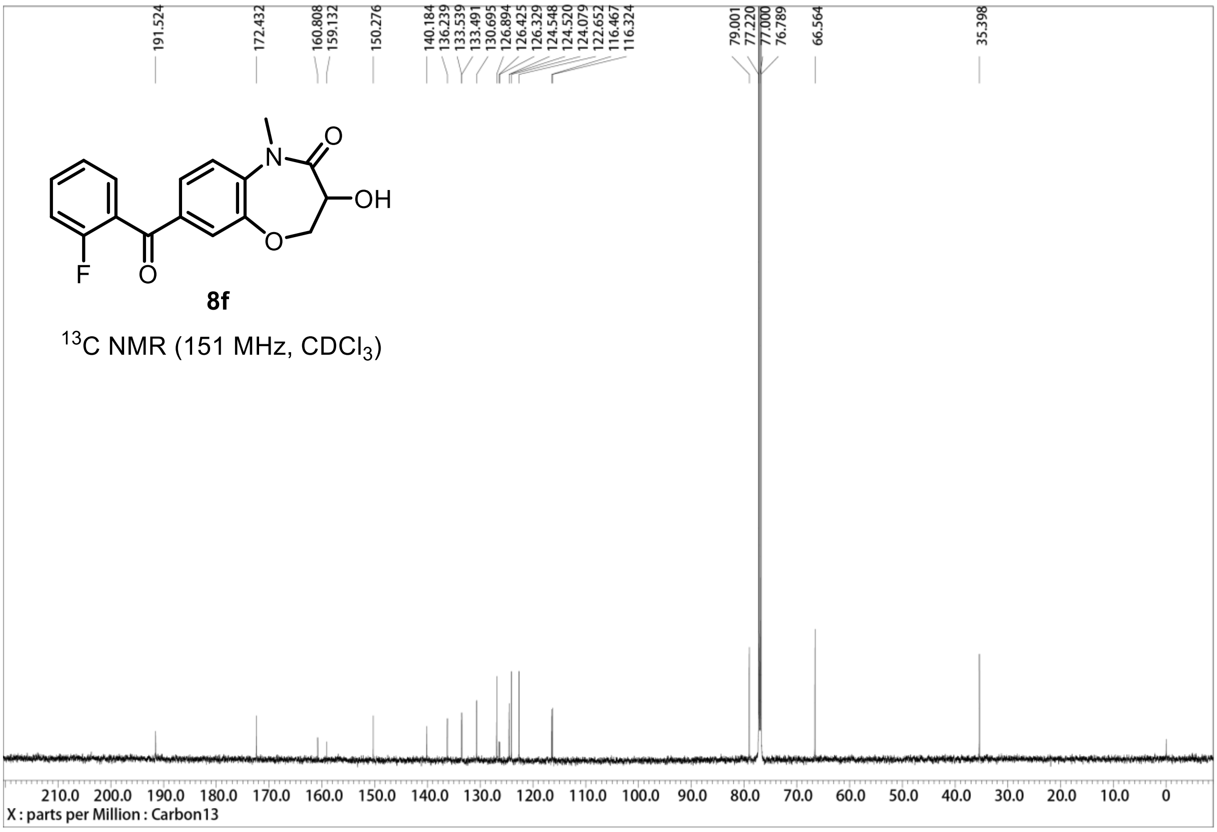
191.524 172.432 160.808 159.132 150.276 140.184 136.239 133.539 133.491 130.695 126.894 126.425 126.329 124.548 124.520 124.079 122.652 116.467 116.324 79.001 77.220 77.000 76.789 66.564 35.398
OH
N
O
O
F
O
8f
^{13}C NMR (151 MHz, $CDCl_3$)
X : parts per Million : Carbon13

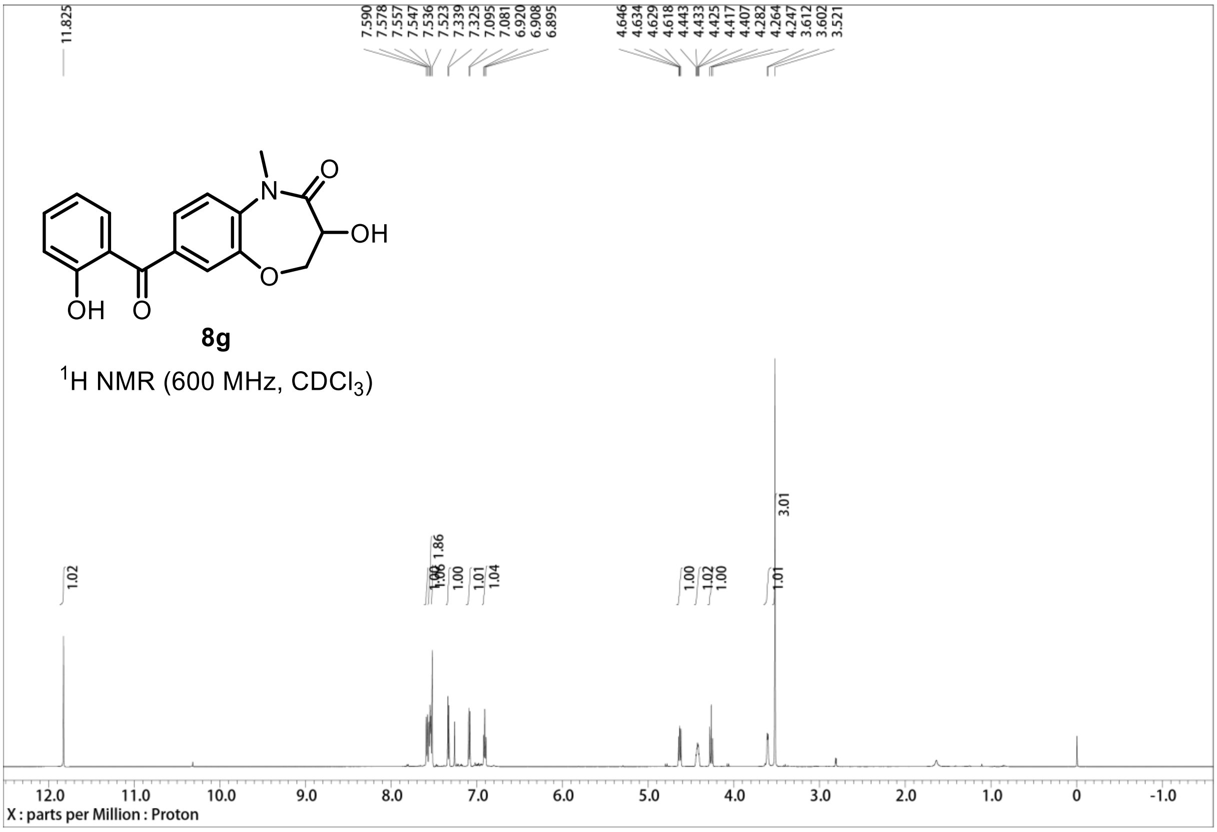
11.825
7.590
7.578
7.557
7.547
7.536
7.523
7.339
7.325
7.095
7.081
6.920
6.908
6.895
4.646
4.634
4.629
4.618
4.443
4.443
4.433
4.425
4.417
4.407
4.282
4.264
4.247
3.612
3.602
3.521
8g
1H NMR (600 MHz, CDCl3)
1.02
1.86
1.00
1.00
1.01
1.04
1.00
1.02
1.00
1.01
3.01
12.0 11.0 10.0 9.0 8.0 7.0 6.0 5.0 4.0 3.0 2.0 1.0 0 -1.0
X : parts per Million : Proton

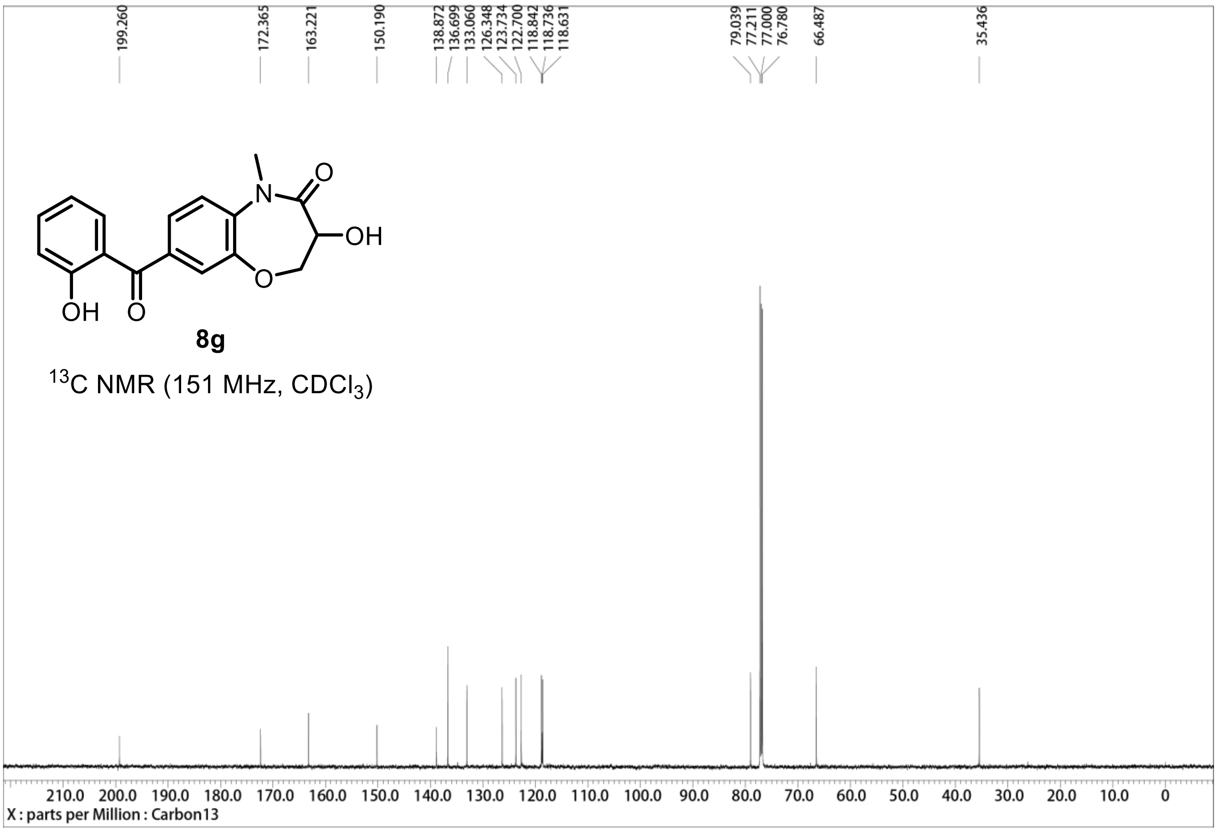
199.260
172.365
163.221
150.190
138.872
136.699
133.060
126.348
123.734
122.700
118.842
118.736
118.631
79.039
77.211
77.000
76.780
66.487
35.436
8g
13C NMR (151 MHz, CDCl3)
210.0 200.0 190.0 180.0 170.0 160.0 150.0 140.0 130.0 120.0 110.0 100.0 90.0 80.0 70.0 60.0 50.0 40.0 30.0 20.0 10.0 0
X : parts per Million : Carbon13

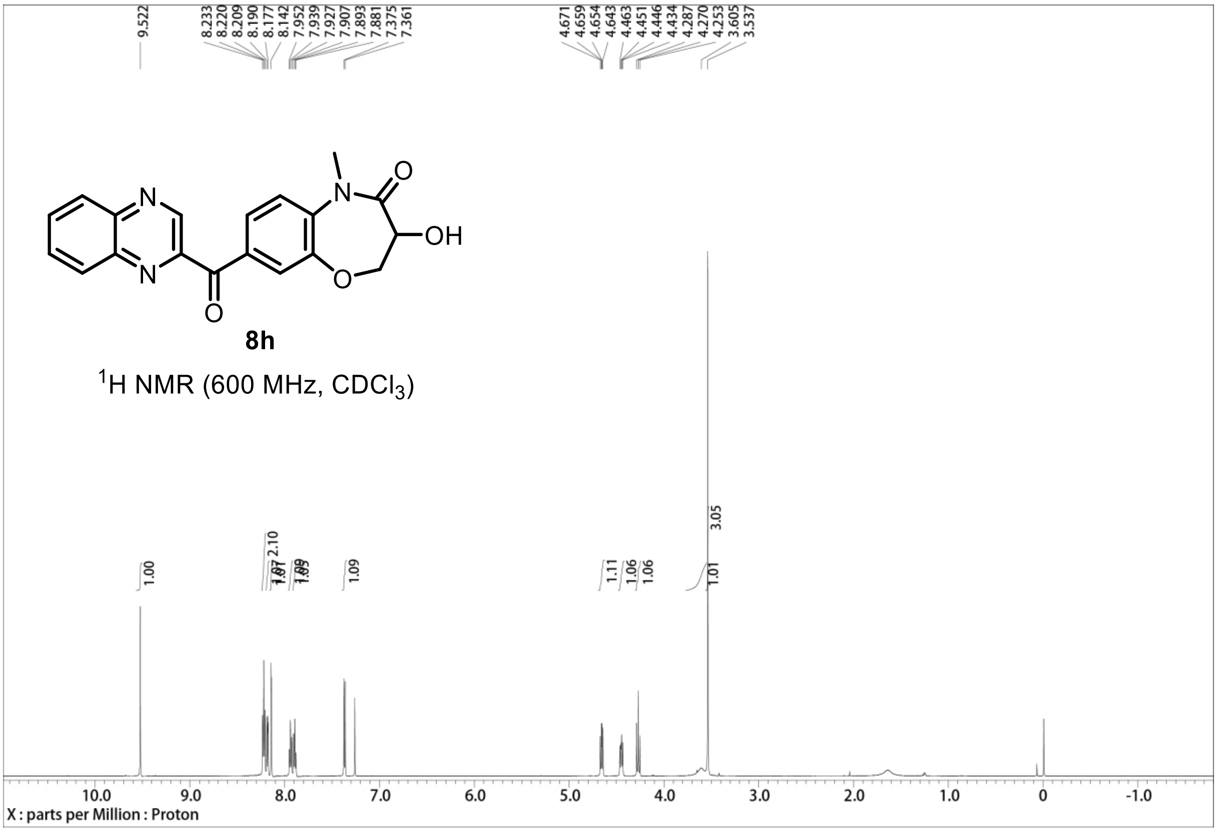
9.522
8.233
8.220
8.209
8.190
8.177
8.142
7.952
7.939
7.927
7.907
7.893
7.881
7.375
7.361
4.671
4.659
4.654
4.643
4.463
4.451
4.446
4.434
4.287
4.270
4.253
3.605
3.537
8h
^{1}H NMR (600 MHz, CDCl$_3$)
X : parts per Million : Proton

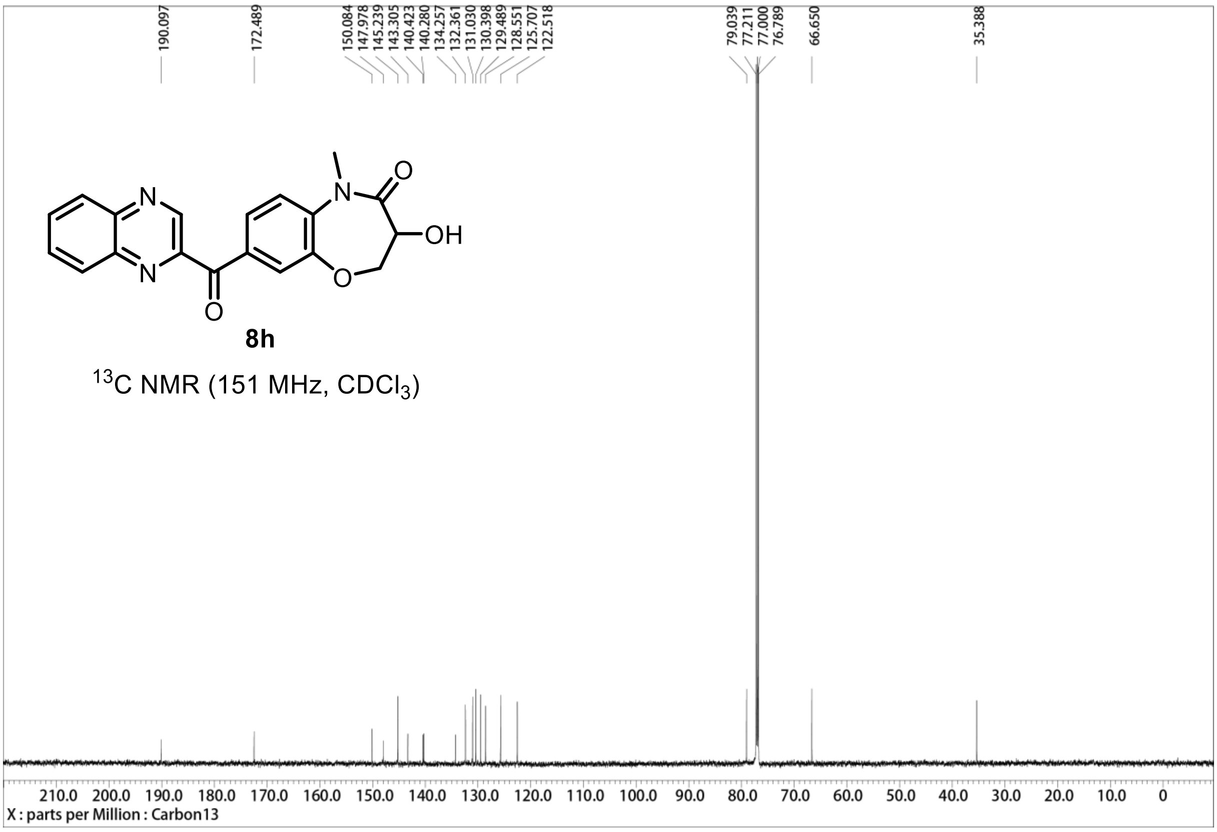
190.097
172.489
150.084
147.978
145.239
143.305
140.423
140.280
134.257
132.361
131.030
130.398
129.489
128.551
125.707
122.518
79.039
77.211
77.000
76.789
66.650
35.388
8h
^{13}C NMR (151 MHz, CDCl$_3$)
X : parts per Million : Carbon13

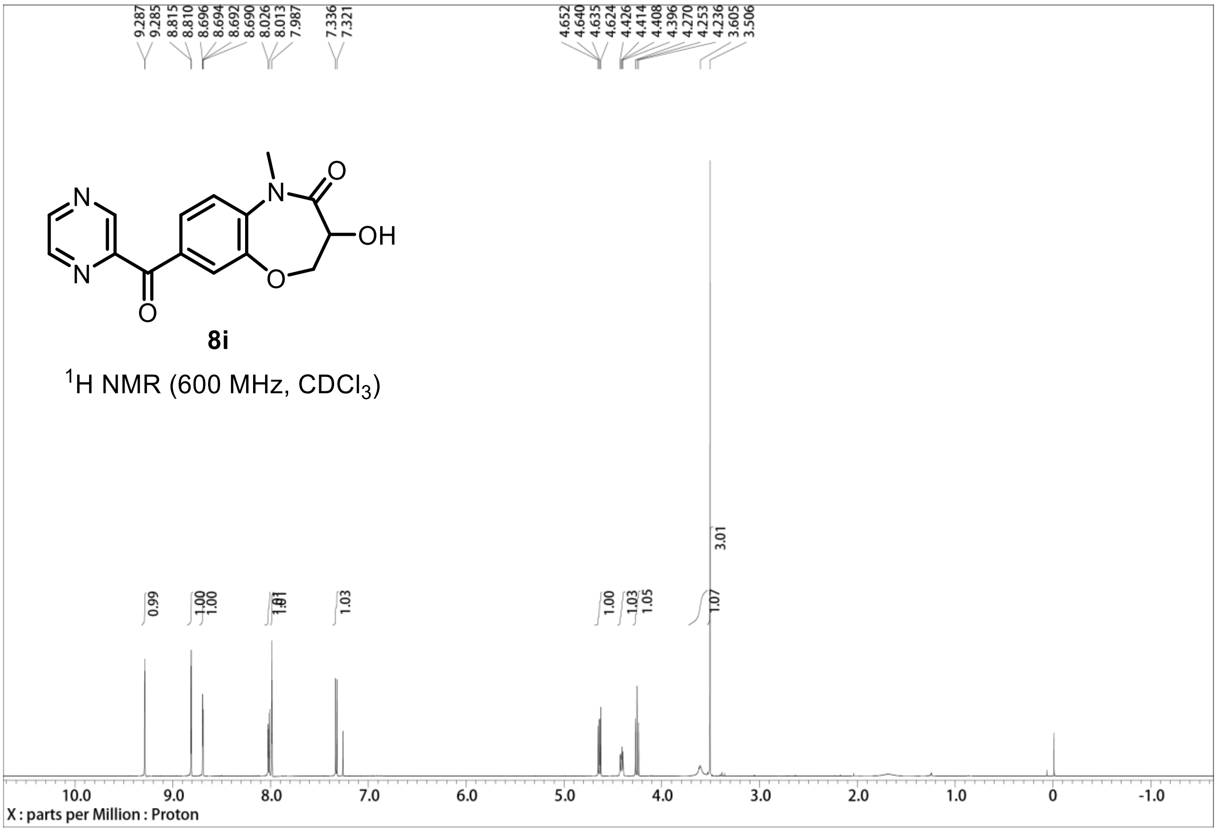
9.287
9.285
8.815
8.810
8.696
8.694
8.692
8.690
8.026
8.013
7.987
7.336
7.321
4.652
4.640
4.635
4.624
4.426
4.414
4.408
4.396
4.270
4.253
4.236
3.605
3.506
8i
^{1}H NMR (600 MHz, $CDCl_3$)
X : parts per Million : Proton

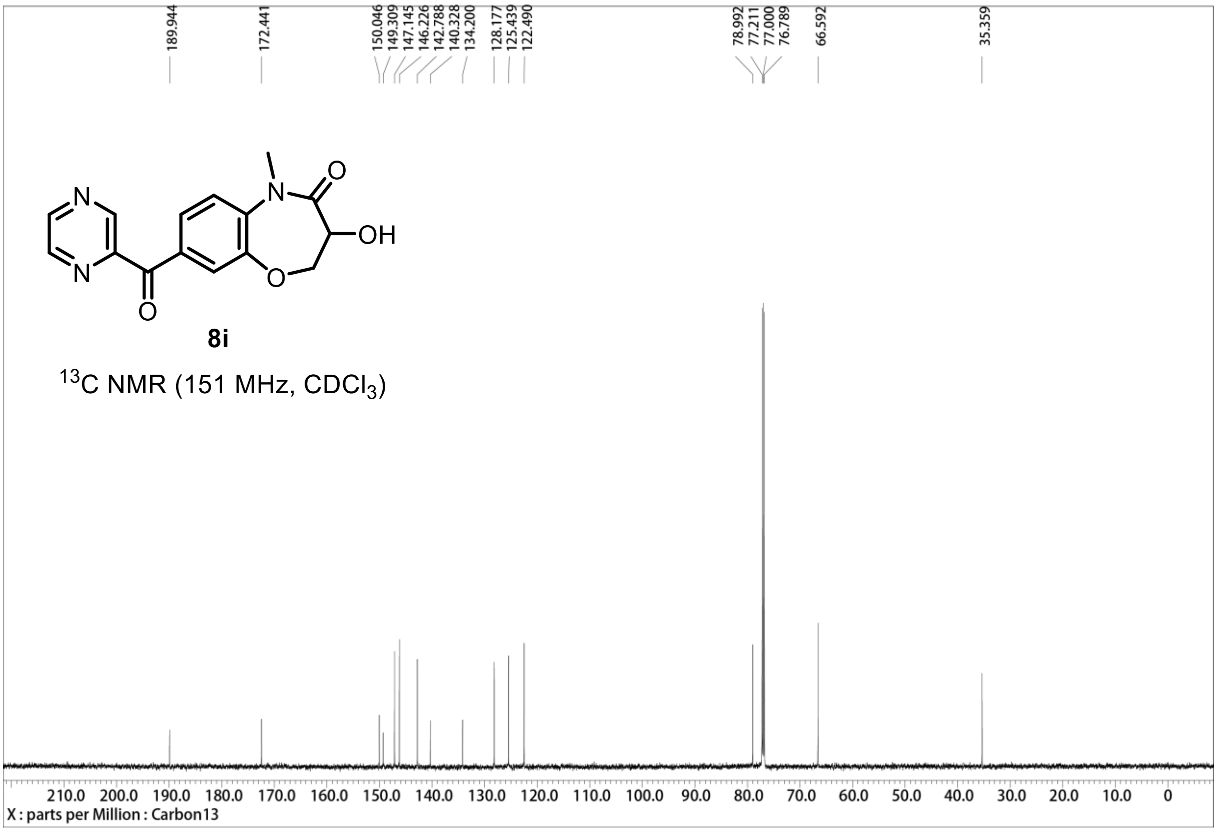
189.944
172.441
150.046
149.309
147.145
146.226
142.788
140.328
134.200
128.177
125.439
122.490
78.992
77.211
77.000
76.789
66.592
35.359
8i
^{13}C NMR (151 MHz, $CDCl_3$)
X : parts per Million : Carbon13

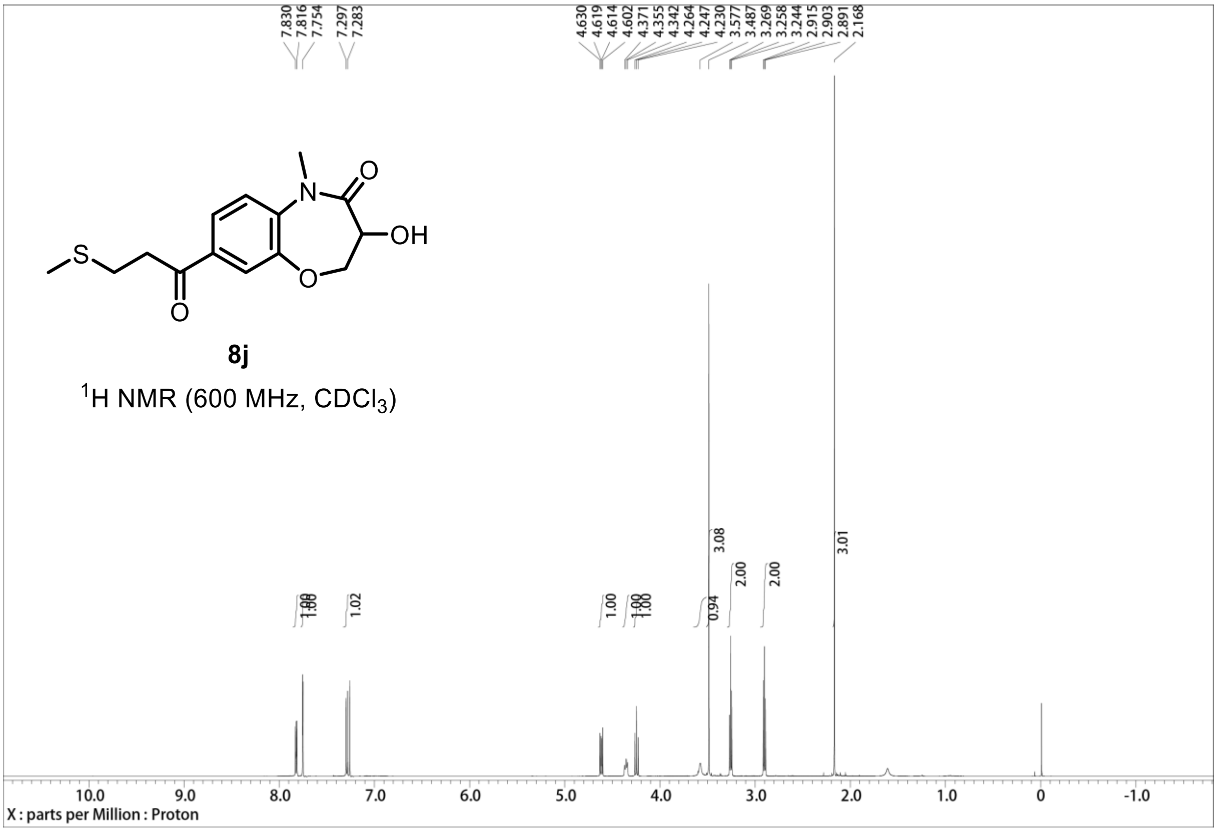
7.830
7.816
7.754
7.297
7.283
4.630
4.619
4.614
4.602
4.371
4.355
4.342
4.264
4.247
4.230
3.577
3.487
3.269
3.258
3.244
2.915
2.903
2.891
2.168
O
N
OH
S
O
O
8j
^{1}H NMR (600 MHz, $CDCl_3$)
1.00
1.00
1.02
1.00
1.00
1.00
0.94
3.08
2.00
2.00
3.01
10.0
9.0
8.0
7.0
6.0
5.0
4.0
3.0
2.0
1.0
0
-1.0
X : parts per Million : Proton

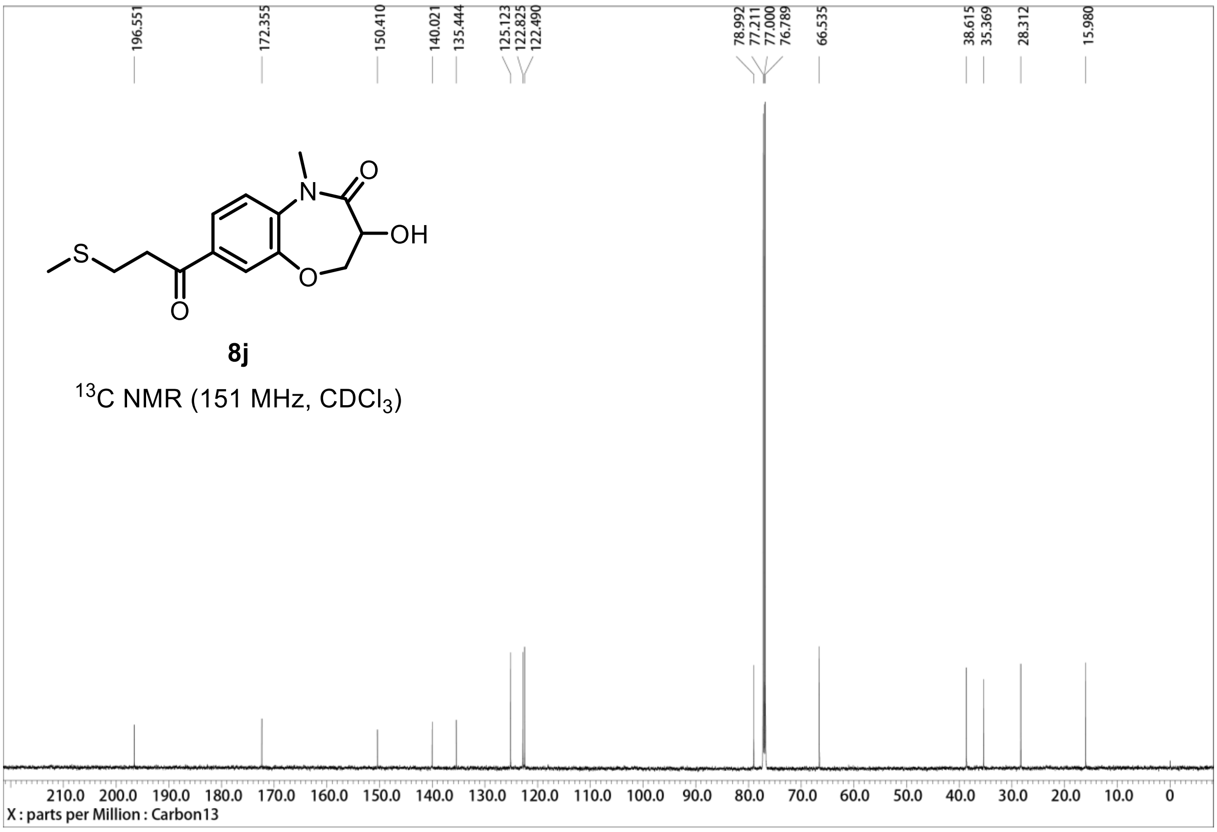
196.551
172.355
150.410
140.021
135.444
125.123
122.825
122.490
78.992
77.211
77.000
76.789
66.535
38.615
35.369
28.312
15.980
O
N
OH
S
O
O
8j
^{13}C NMR (151 MHz, $CDCl_3$)
210.0
200.0
190.0
180.0
170.0
160.0
150.0
140.0
130.0
120.0
110.0
100.0
90.0
80.0
70.0
60.0
50.0
40.0
30.0
20.0
10.0
0
X : parts per Million : Carbon13

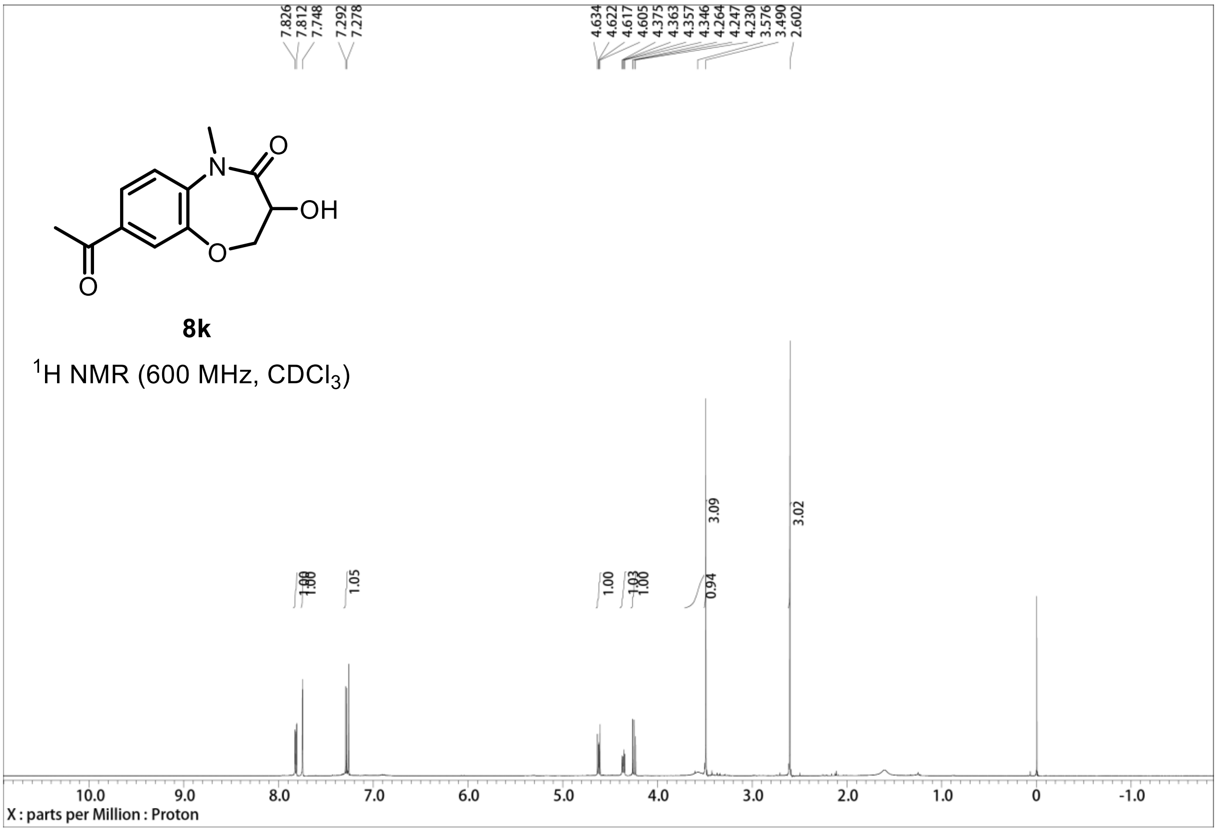
7.826
7.812
7.748
7.292
7.278
4.634
4.622
4.617
4.605
4.375
4.363
4.357
4.346
4.264
4.247
4.230
3.576
3.490
2.602
8k
^{1}H NMR (600 MHz, CDCl$_3$)
3.09
3.02
1.05
1.00
1.03
1.00
0.94
X : parts per Million : Proton

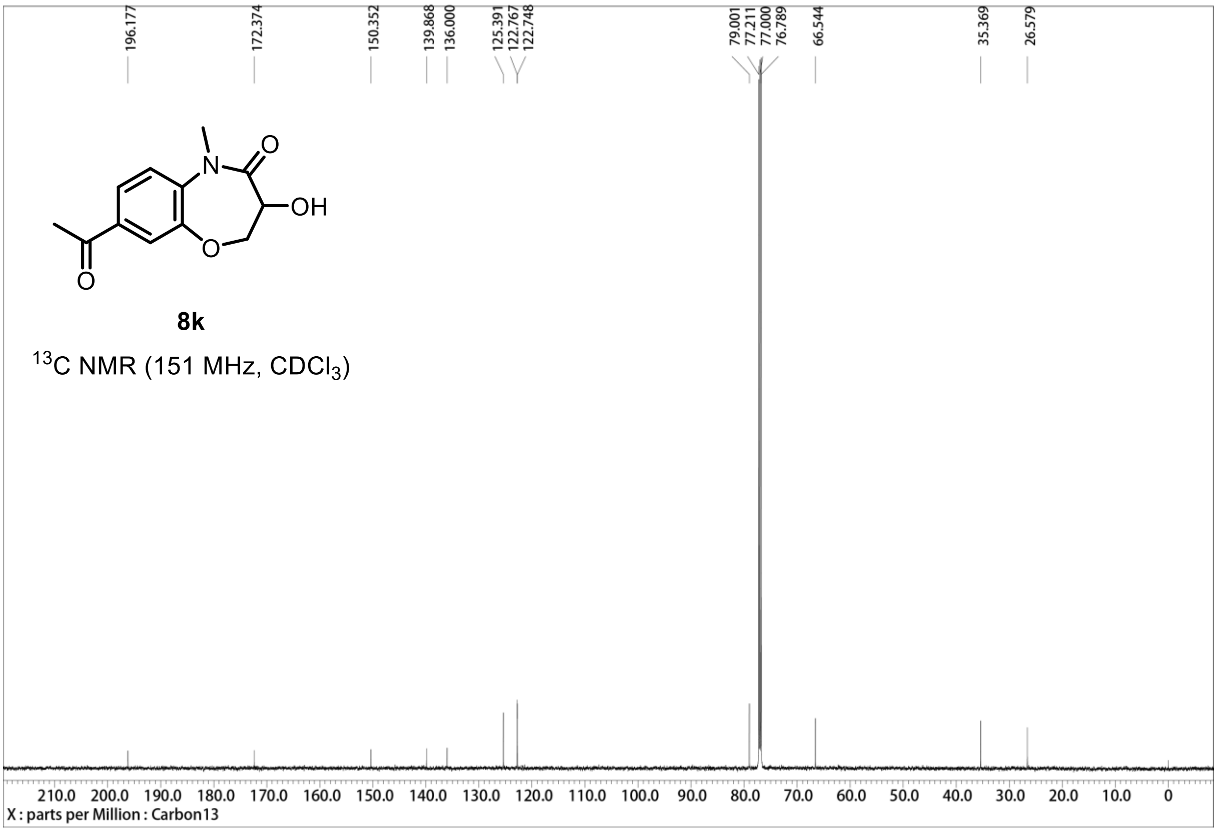
196.177
172.374
150.352
139.868
136.000
125.391
122.767
122.748
79.001
77.211
77.000
76.789
66.544
35.369
26.579
8k
^{13}C NMR (151 MHz, CDCl$_3$)
X : parts per Million : Carbon13

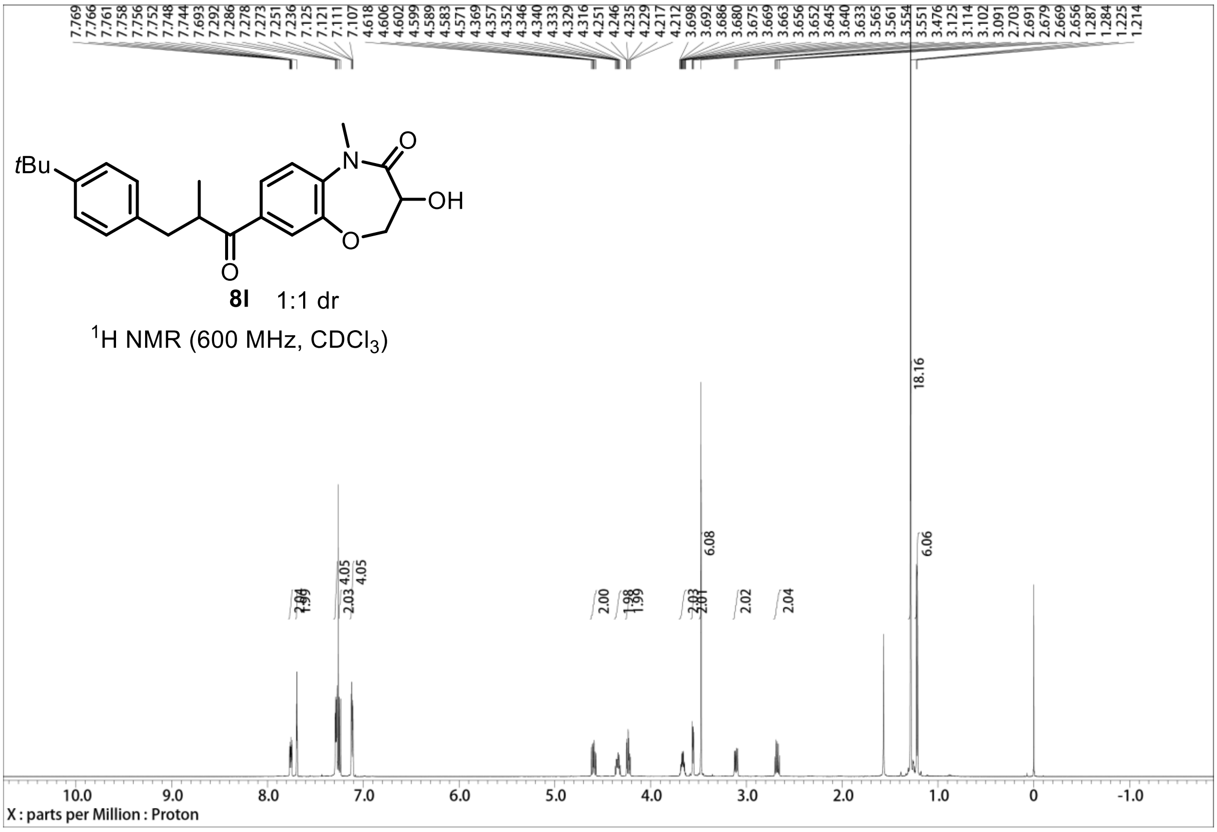
tBu
OH
8l 1:1 dr
1H NMR (600 MHz, CDCl3)
X : parts per Million : Proton

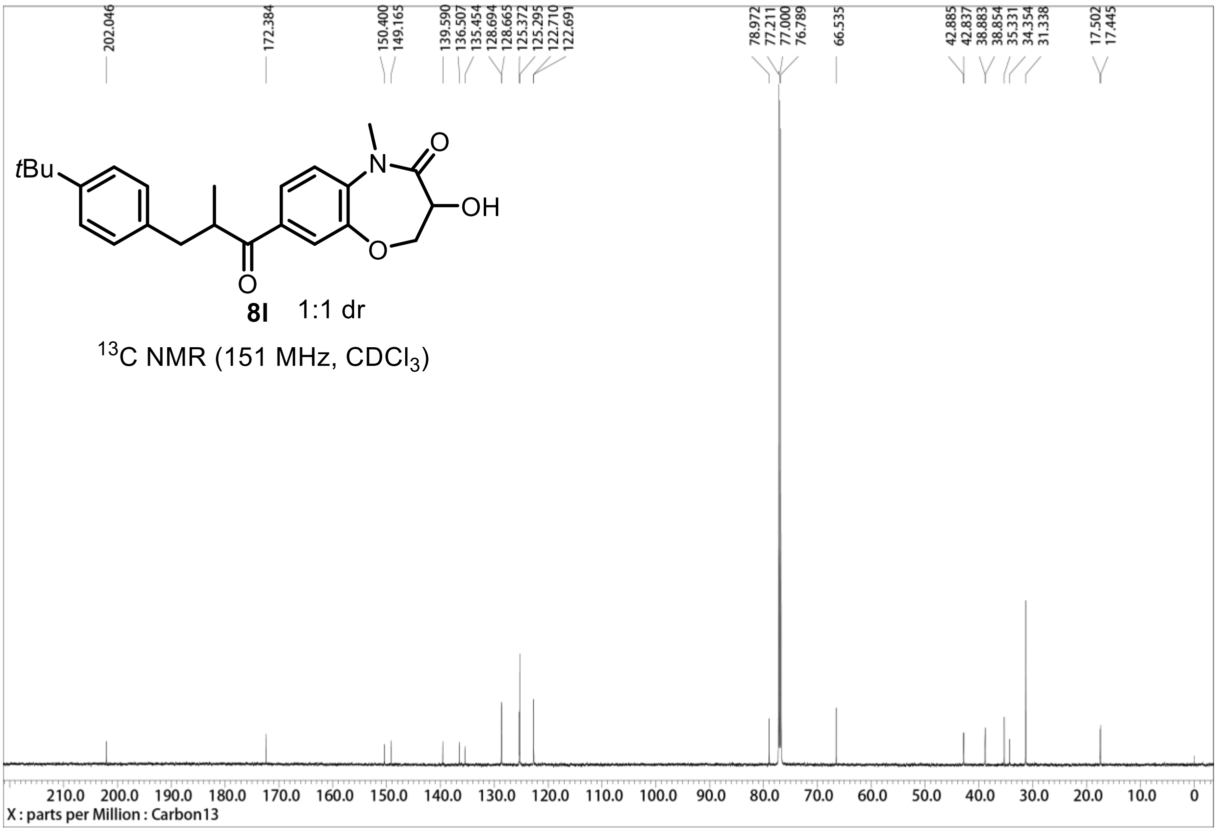
tBu
OH
8l 1:1 dr
13C NMR (151 MHz, CDCl3)
X : parts per Million : Carbon13

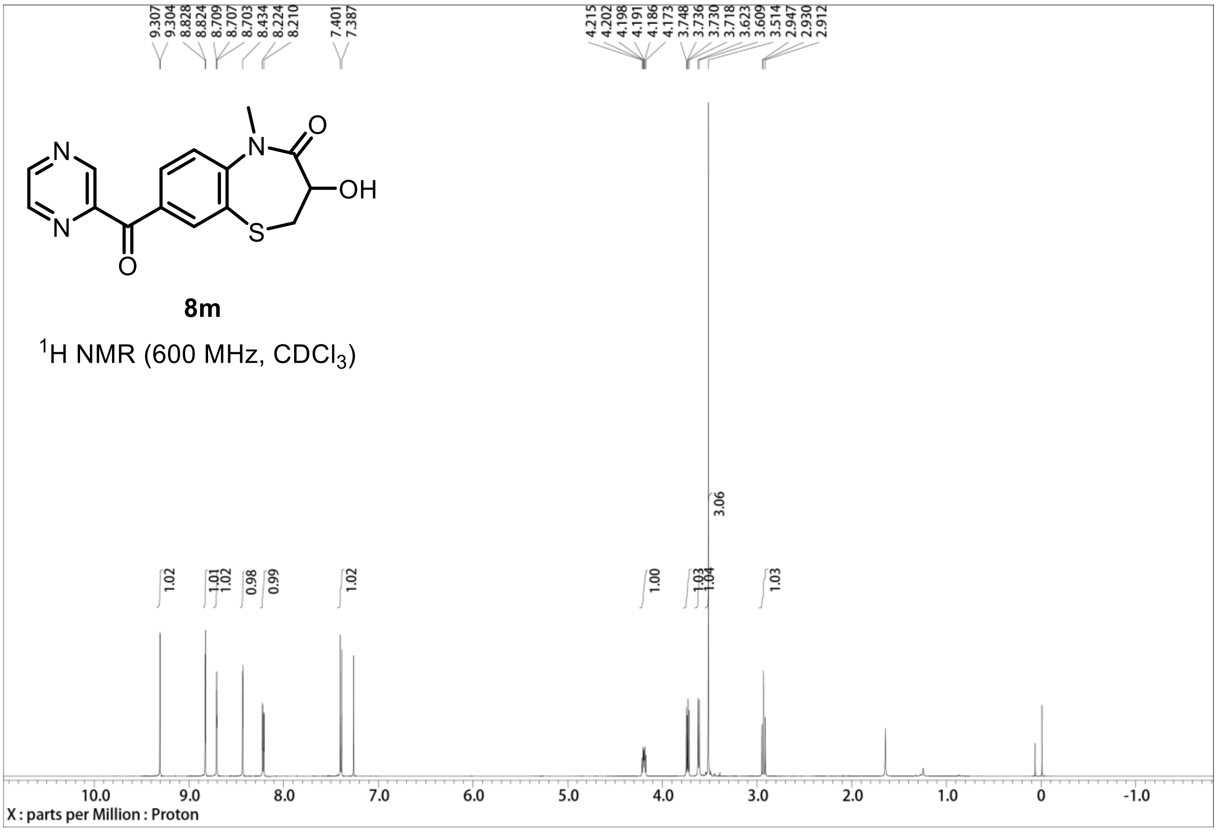
9.307
9.304
8.828
8.824
8.709
8.707
8.703
8.434
8.224
8.210
7.401
7.387
4.215
4.202
4.198
4.191
4.186
4.173
3.748
3.736
3.730
3.718
3.623
3.609
3.514
2.947
2.930
2.912
N
N
N
O
OH
S
O
8m
¹H NMR (600 MHz, CDCl₃)
3.06
1.02
1.01
1.02
0.98
0.99
1.02
1.00
1.03
1.04
1.03
10.0
9.0
8.0
7.0
6.0
5.0
4.0
3.0
2.0
1.0
0
-1.0
X : parts per Million : Proton

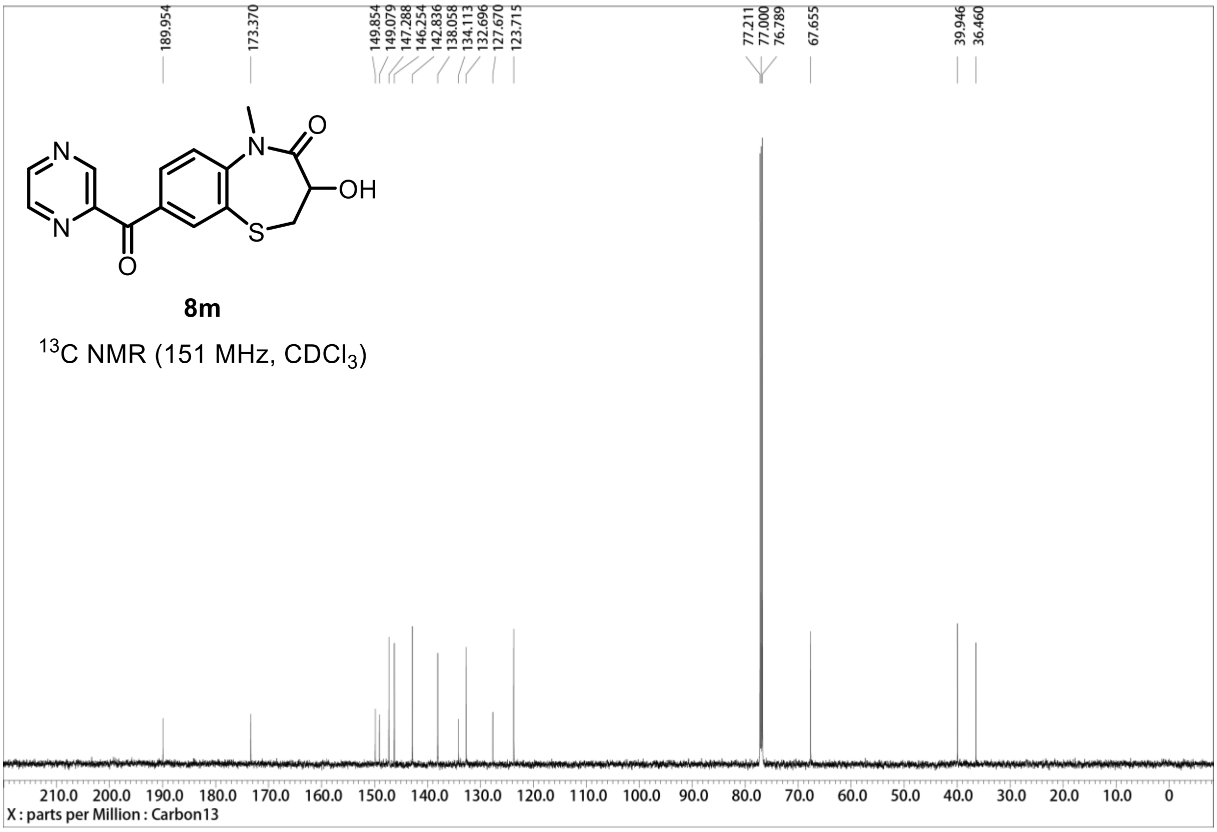
189.954
173.370
149.854
149.079
147.288
146.254
142.836
138.058
134.113
132.696
127.670
123.715
77.211
77.000
76.789
67.655
39.946
36.460
N
N
N
O
OH
S
O
8m
¹³C NMR (151 MHz, CDCl₃)
210.0
200.0
190.0
180.0
170.0
160.0
150.0
140.0
130.0
120.0
110.0
100.0
90.0
80.0
70.0
60.0
50.0
40.0
30.0
20.0
10.0
0
X : parts per Million : Carbon13

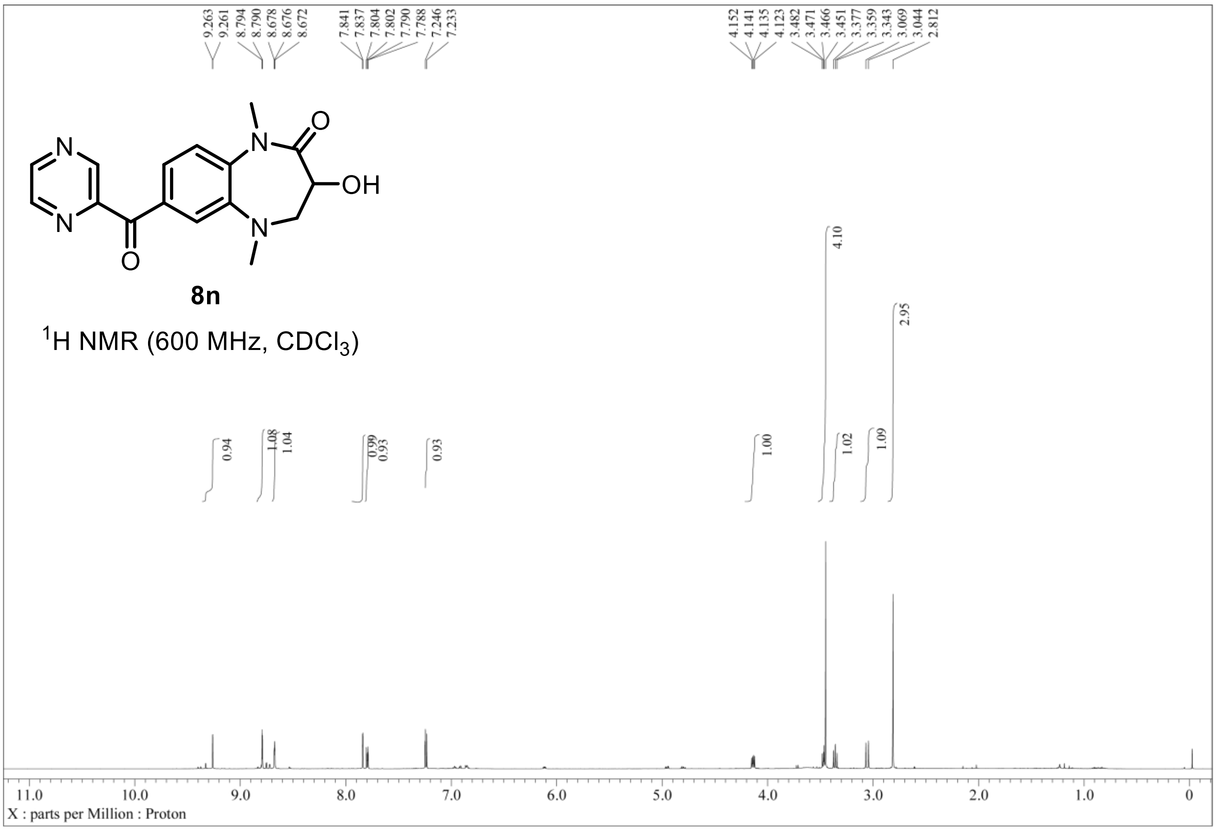
9.263
9.261
8.794
8.790
8.678
8.676
8.672
7.841
7.837
7.804
7.802
7.790
7.788
7.246
7.233
4.152
4.141
4.135
4.123
3.482
3.471
3.466
3.451
3.377
3.359
3.343
3.069
3.044
2.812
N
N
N
N
O
OH
O
8n
1H NMR (600 MHz, CDCl3)
4.10
2.95
0.94
1.08
1.04
0.99
0.93
0.93
1.00
1.02
1.09
11.0 10.0 9.0 8.0 7.0 6.0 5.0 4.0 3.0 2.0 1.0 0
X : parts per Million : Proton

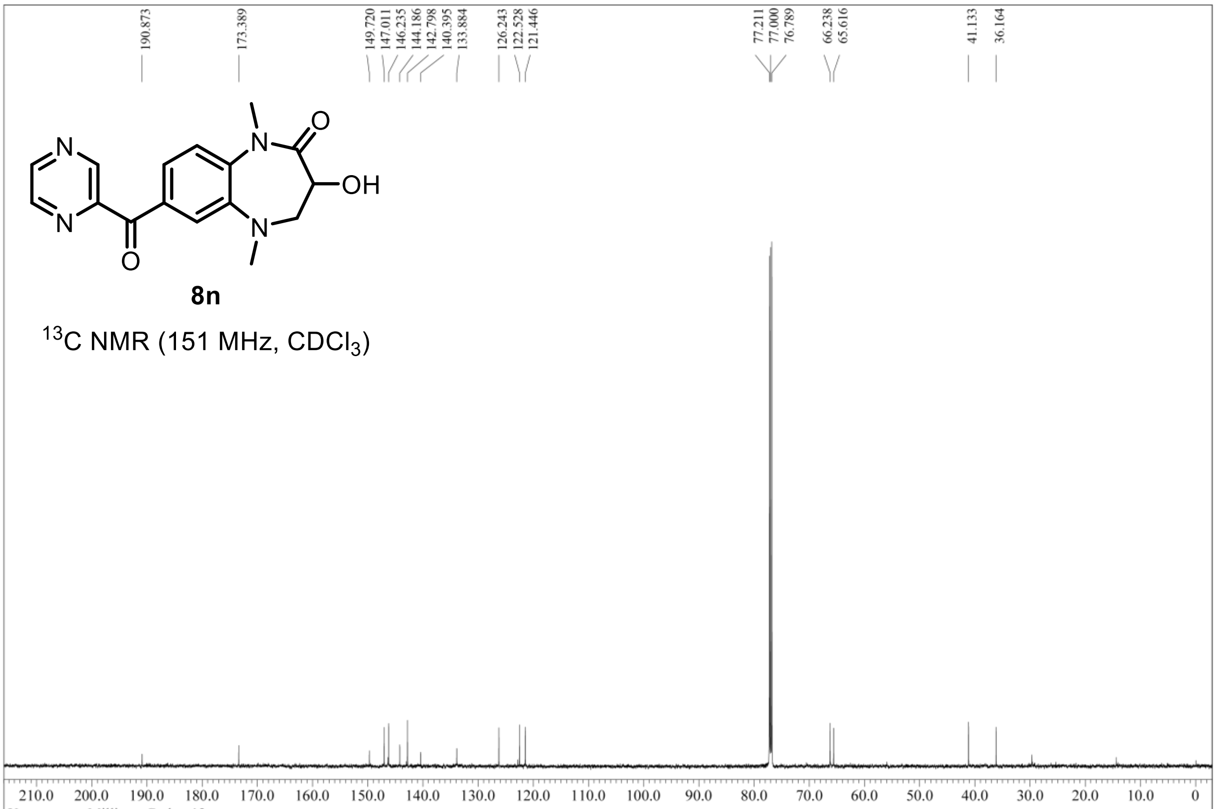
190.873
173.389
149.720
147.011
146.235
144.186
142.798
140.395
133.884
126.243
122.528
121.446
77.211
77.000
76.789
66.238
65.616
41.133
36.164
N
N
N
N
O
OH
O
8n
13C NMR (151 MHz, CDCl3)
210.0 200.0 190.0 180.0 170.0 160.0 150.0 140.0 130.0 120.0 110.0 100.0 90.0 80.0 70.0 60.0 50.0 40.0 30.0 20.0 10.0 0
X : parts per Million : Carbon13

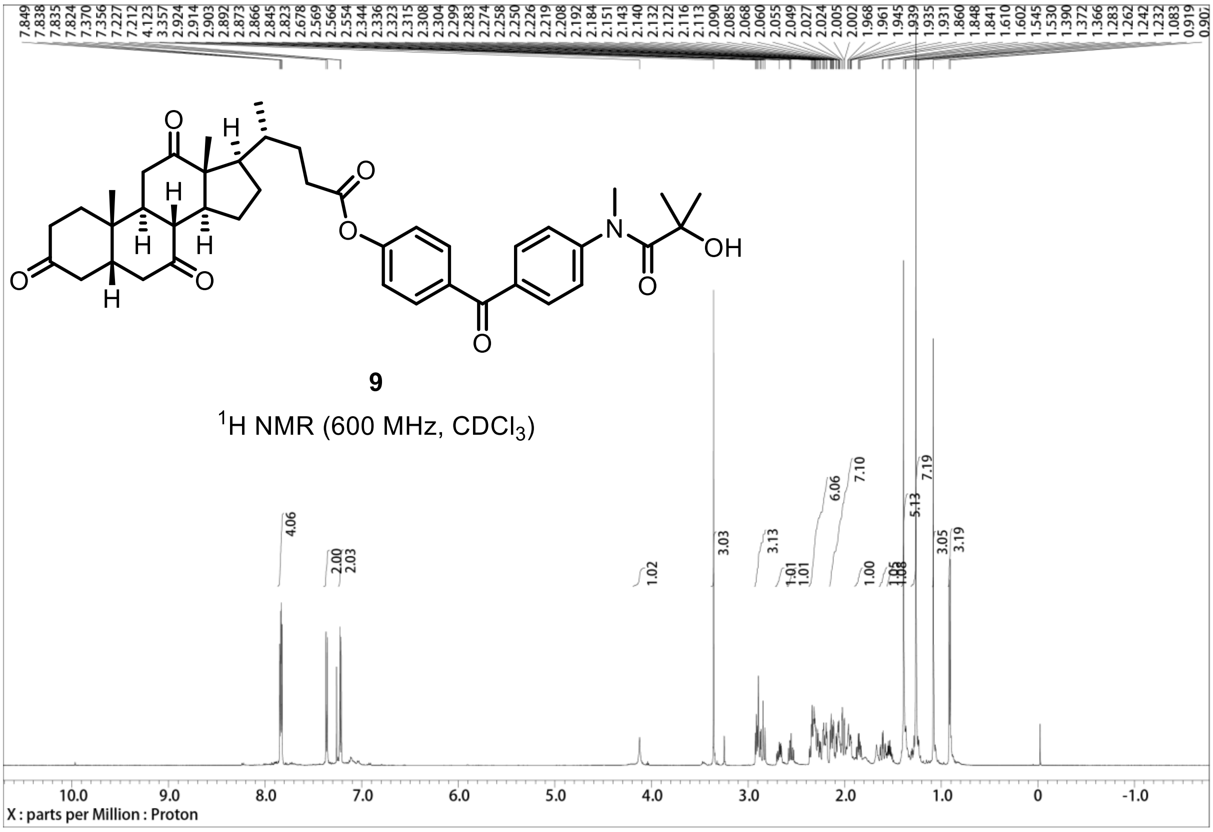
7.849 7.838 7.835 7.824 7.370 7.356 7.227 7.212 4.123 3.357 2.924 2.914 2.903 2.892 2.873 2.866 2.845 2.823 2.678 2.569 2.566 2.554 2.344 2.336 2.323 2.315 2.308 2.304 2.299 2.283 2.274 2.258 2.250 2.226 2.219 2.208 2.192 2.184 2.151 2.143 2.140 2.132 2.122 2.116 2.113 2.090 2.085 2.068 2.060 2.055 2.049 2.027 2.024 2.005 2.002 1.968 1.961 1.945 1.939 1.935 1.931 1.860 1.848 1.841 1.610 1.602 1.545 1.530 1.390 1.372 1.366 1.283 1.262 1.242 1.232 1.083 0.919 0.902
O
H
O
O
H
OH
N
H
H
O
O
H
O
O
9
^{1}H NMR (600 MHz, $CDCl_3$)
X : parts per Million : Proton

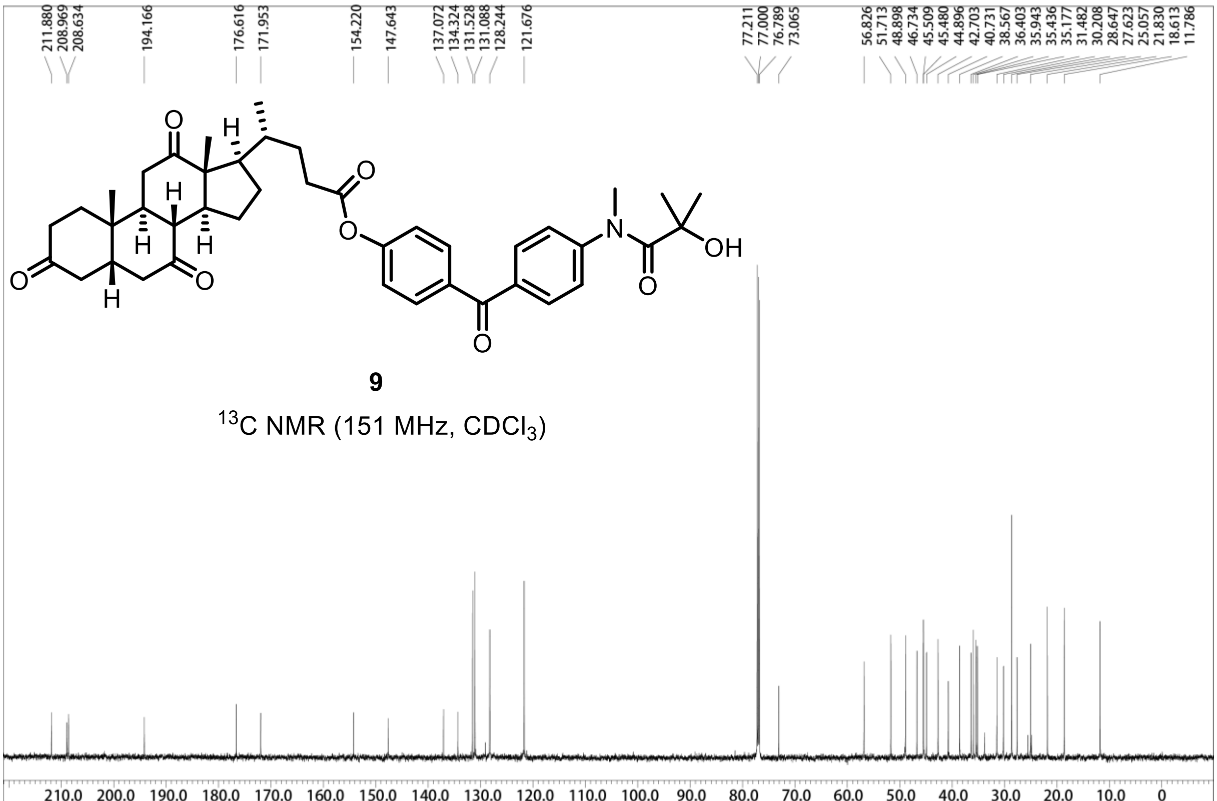
211.880 208.969 208.634 194.166 176.616 171.953 154.220 147.643 137.072 134.324 131.528 131.088 128.244 121.676 77.211 77.000 76.789 73.065 56.826 51.713 48.898 46.734 45.509 45.480 44.896 42.703 40.731 38.567 36.403 35.943 35.436 35.177 31.482 30.208 28.647 27.623 25.057 21.830 18.613 11.786
9
^{13}C NMR (151 MHz, $CDCl_3$)
X : parts per Million : Carbon13

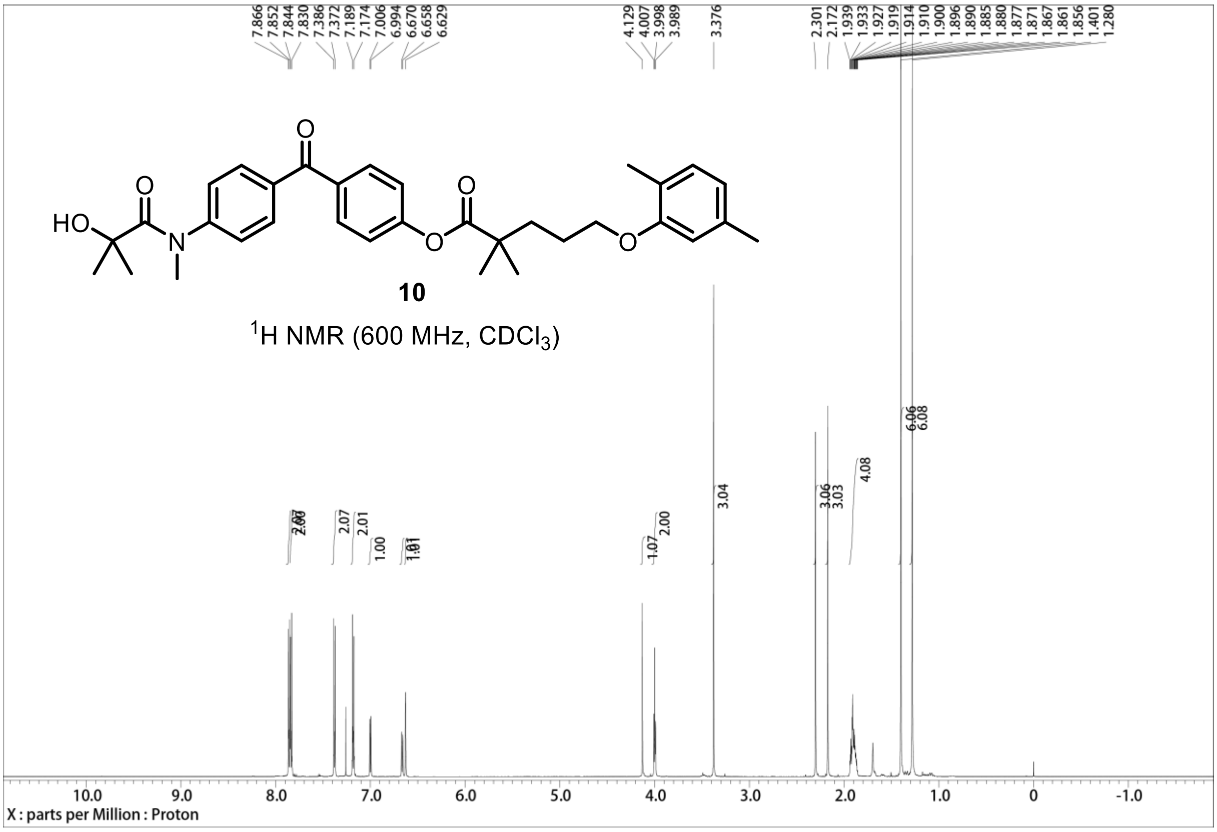
HO
O
N
O
O
O
O
10
1H NMR (600 MHz, CDCl3)
7.866
7.852
7.844
7.830
7.386
7.372
7.189
7.174
7.006
6.994
6.670
6.658
6.629
4.129
4.007
3.998
3.989
3.376
2.301
2.172
1.939
1.933
1.927
1.919
1.914
1.910
1.900
1.896
1.890
1.885
1.880
1.877
1.871
1.867
1.861
1.856
1.401
1.280
2.07
2.01
1.00
1.07
2.00
3.04
3.06
3.03
4.08
6.06
6.08
10.0 9.0 8.0 7.0 6.0 5.0 4.0 3.0 2.0 1.0 0 -1.0
X : parts per Million : Proton

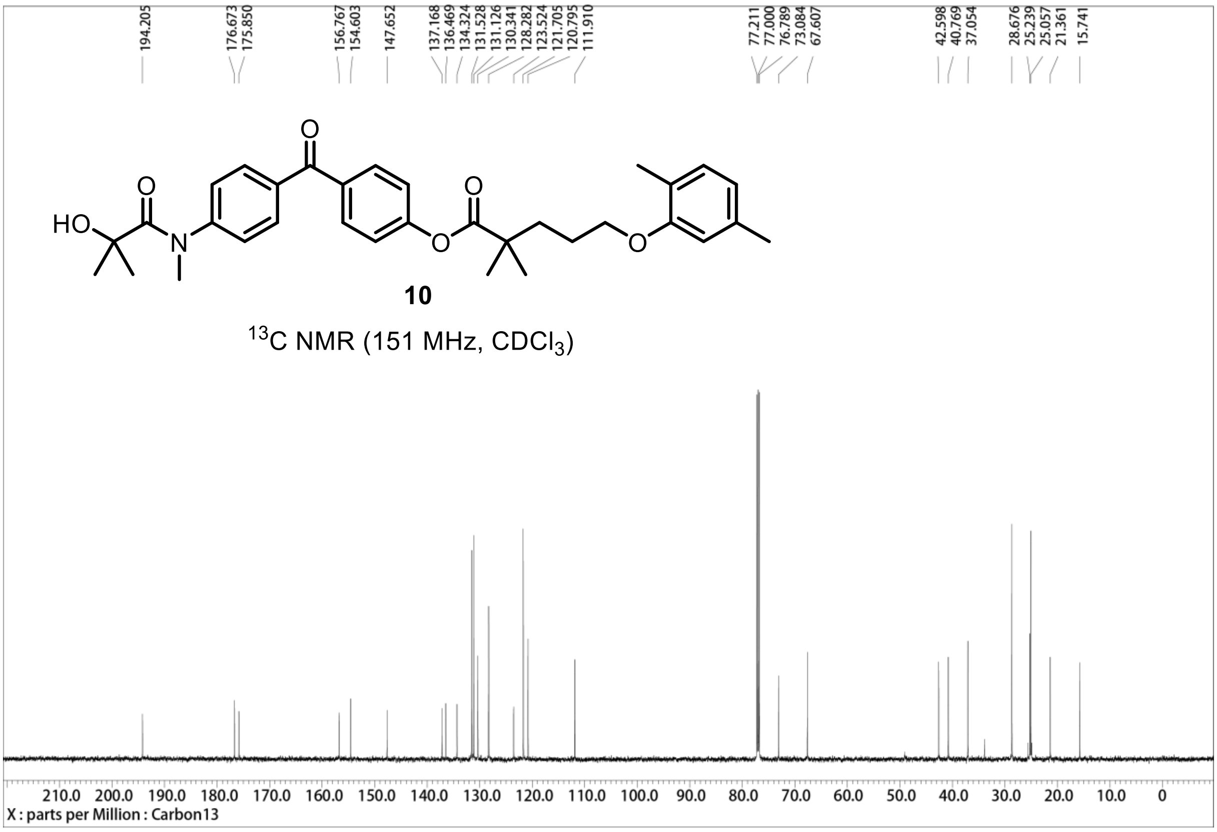
HO
O
N
O
O
O
O
10
13C NMR (151 MHz, CDCl3)
194.205
176.673
175.850
156.767
154.603
147.652
137.168
136.469
134.324
131.528
131.126
130.341
128.282
123.524
121.705
120.795
111.910
77.211
77.000
76.789
73.084
67.607
42.598
40.769
37.054
28.676
25.239
25.057
21.361
15.741
210.0 200.0 190.0 180.0 170.0 160.0 150.0 140.0 130.0 120.0 110.0 100.0 90.0 80.0 70.0 60.0 50.0 40.0 30.0 20.0 10.0 0
X : parts per Million : Carbon13

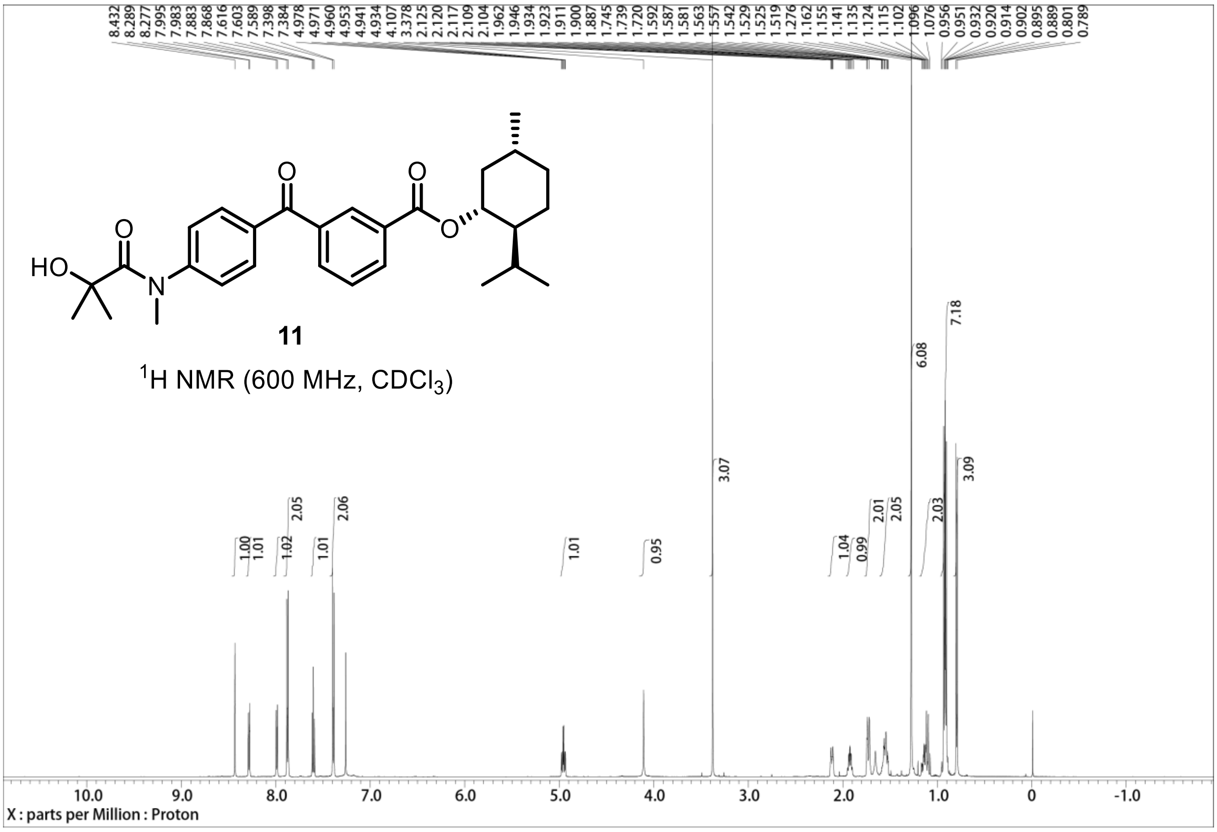

8.432 8.289 8.277 7.995 7.983 7.883 7.868 7.616 7.603 7.589 7.398 7.384 4.978 4.971 4.960 4.953 4.941 4.934 4.107 3.378 2.125 2.120 2.117 2.109 2.104 1.962 1.946 1.934 1.923 1.911 1.900 1.887 1.745 1.739 1.720 1.592 1.587 1.581 1.563 1.557 1.542 1.529 1.525 1.519 1.276 1.162 1.155 1.141 1.135 1.124 1.115 1.102 1.096 1.076 0.956 0.951 0.932 0.920 0.914 0.902 0.895 0.889 0.801 0.789
11
^{1}H NMR (600 MHz, CDCl$_3$)
X : parts per Million : Proton

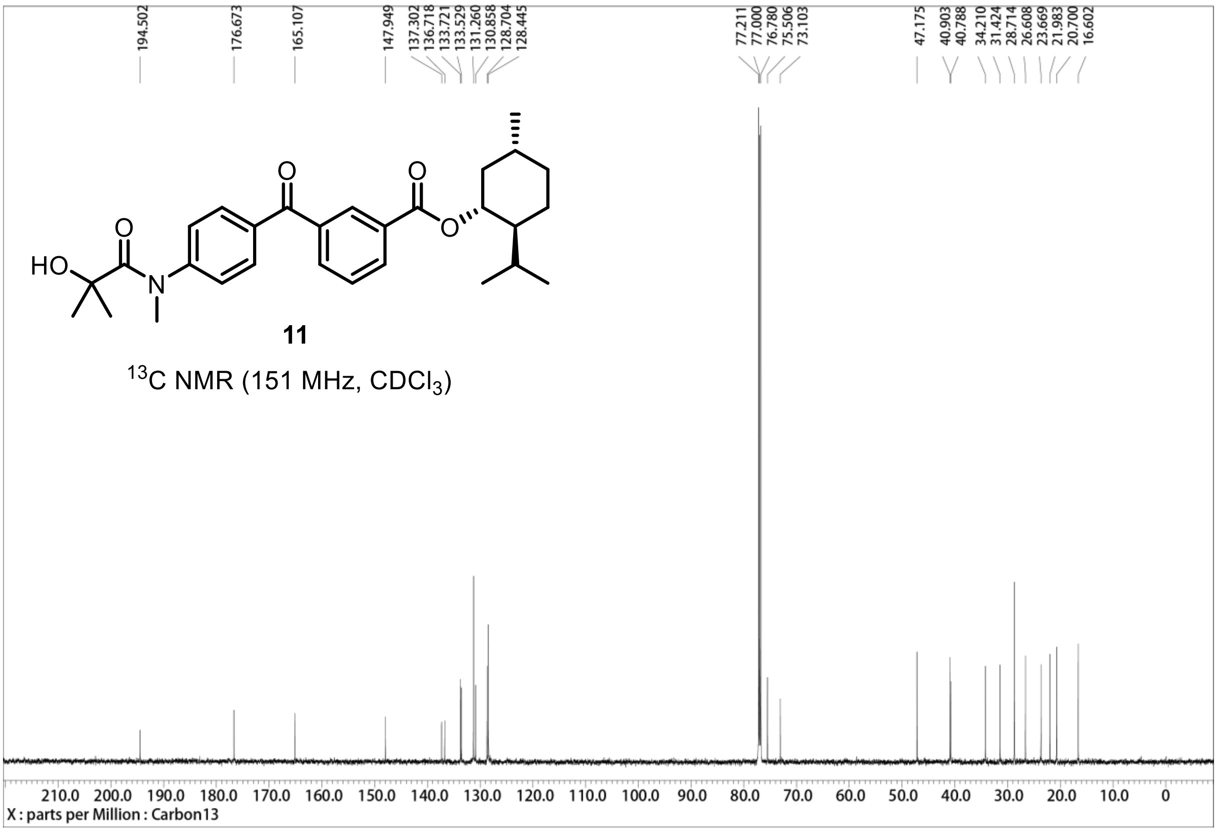

194.502 176.673 165.107 147.949 137.302 136.718 133.721 133.529 131.260 130.858 128.704 128.445 77.211 77.000 76.780 75.506 73.103 47.175 40.903 40.788 34.210 31.424 28.714 26.608 23.669 21.983 20.700 16.602
11
^{13}C NMR (151 MHz, CDCl$_3$)
X : parts per Million : Carbon13

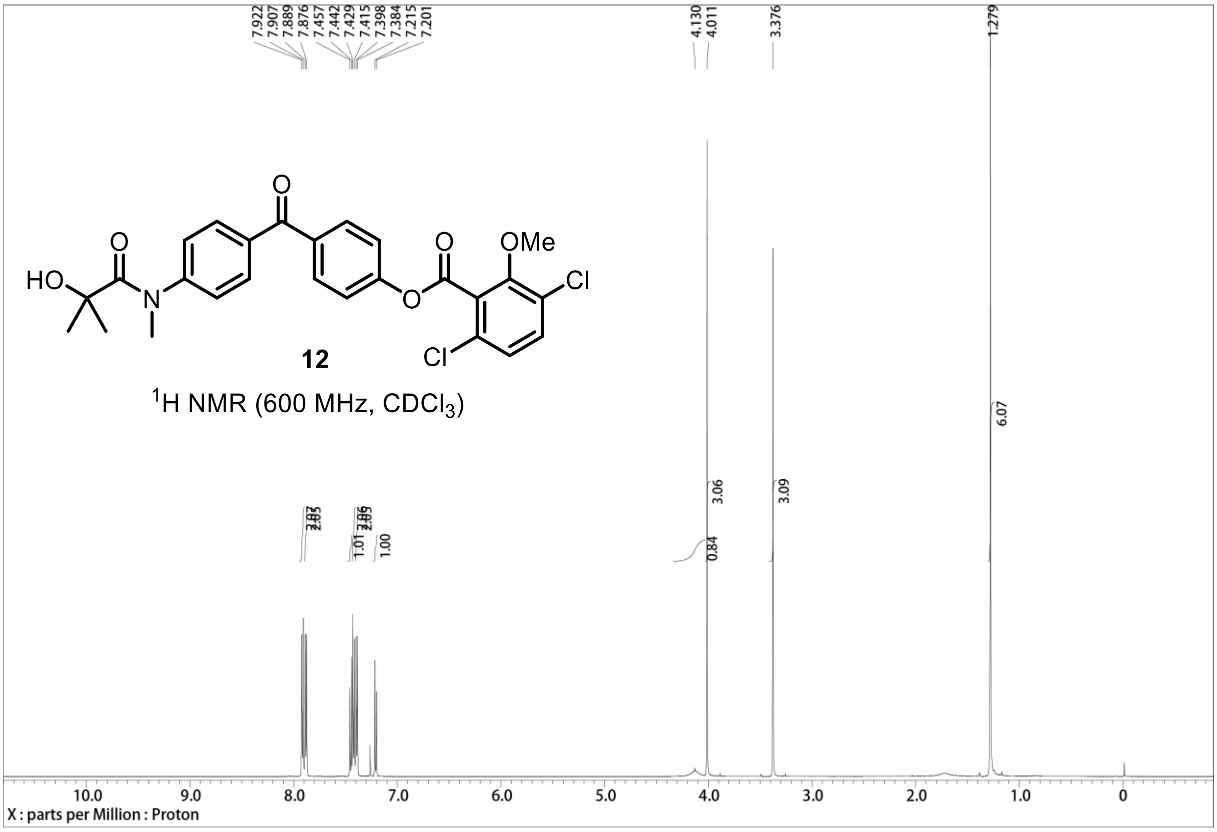
7.922
7.907
7.889
7.876
7.457
7.442
7.429
7.415
7.398
7.384
7.215
7.201
4.130
4.011
3.376
1.279
O
O
OMe
HO
N
O
Cl
O
Cl
12
^{1}H NMR (600 MHz, $CDCl_3$)
1.01
1.00
0.84
3.06
3.09
6.07
10.0
9.0
8.0
7.0
6.0
5.0
4.0
3.0
2.0
1.0
0
X : parts per Million : Proton

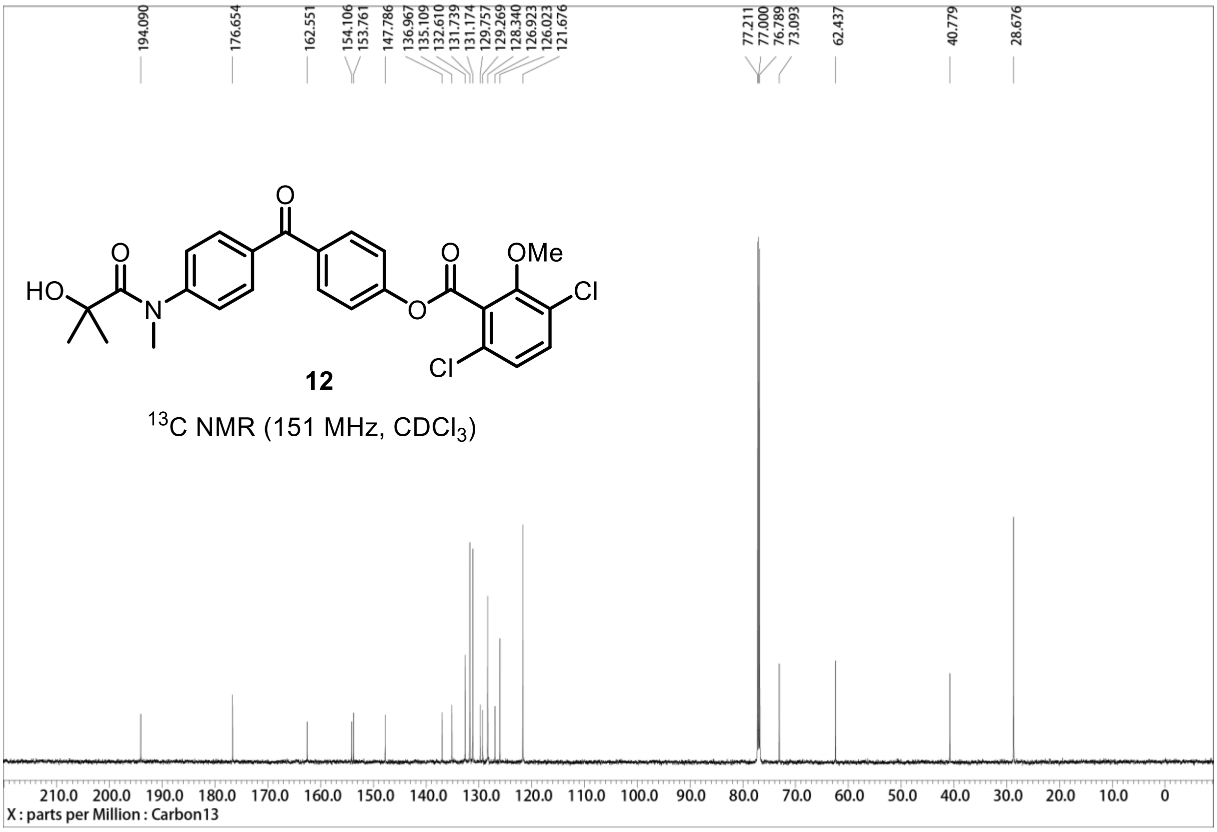
194.090
176.654
162.551
154.106
153.761
147.786
136.967
135.109
132.610
131.739
131.174
129.757
129.269
128.340
126.923
126.023
121.676
77.211
77.000
76.789
73.093
62.437
40.779
28.676
O
O
OMe
HO
N
O
Cl
O
Cl
12
^{13}C NMR (151 MHz, $CDCl_3$)
210.0
200.0
190.0
180.0
170.0
160.0
150.0
140.0
130.0
120.0
110.0
100.0
90.0
80.0
70.0
60.0
50.0
40.0
30.0
20.0
10.0
0
X : parts per Million : Carbon13

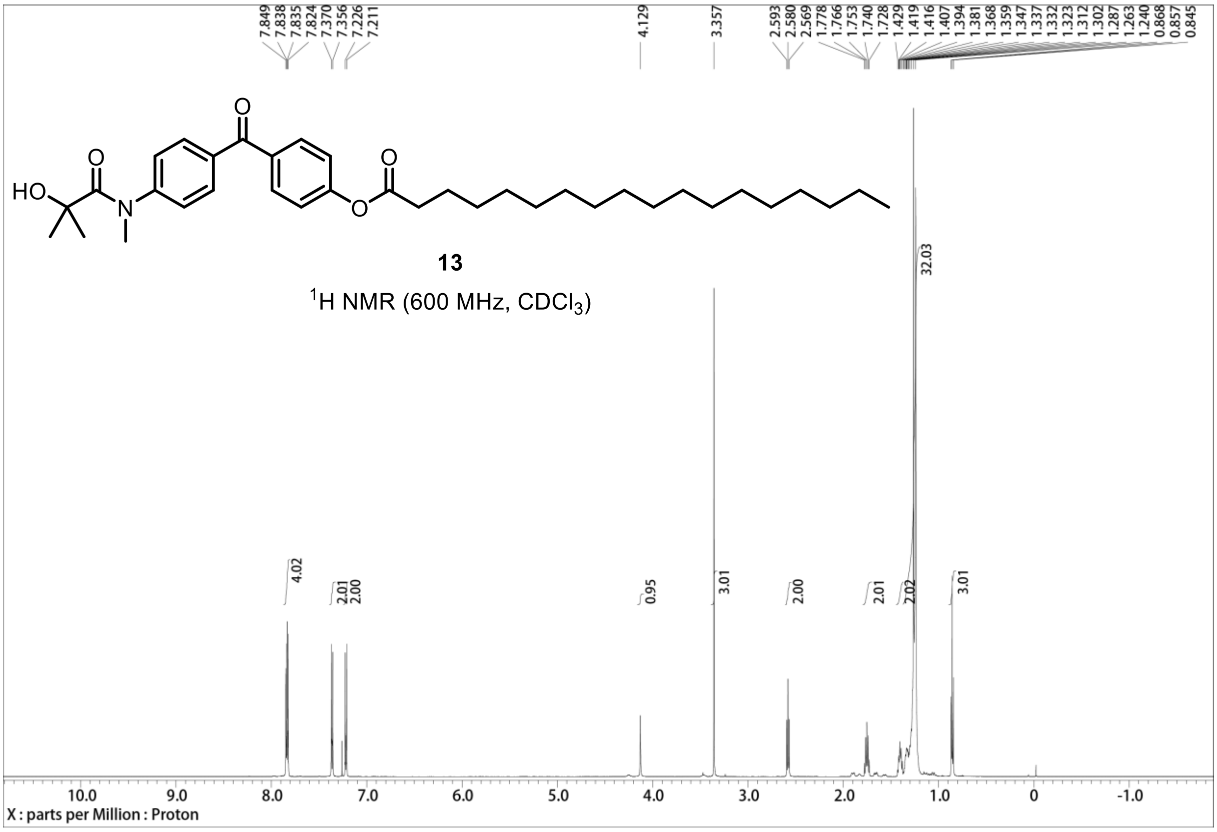

7.849
7.838
7.835
7.824
7.370
7.356
7.226
7.211
4.129
3.357
2.593
2.580
2.569
1.778
1.766
1.753
1.740
1.728
1.429
1.419
1.416
1.407
1.394
1.381
1.368
1.359
1.347
1.337
1.332
1.323
1.312
1.302
1.287
1.263
1.240
0.868
0.857
0.845
HO
O
N
O
O
O
13
1H NMR (600 MHz, CDCl3)
32.03
4.02
2.01
2.00
0.95
3.01
2.00
2.01
2.02
3.01
10.0 9.0 8.0 7.0 6.0 5.0 4.0 3.0 2.0 1.0 0 -1.0
X : parts per Million : Proton

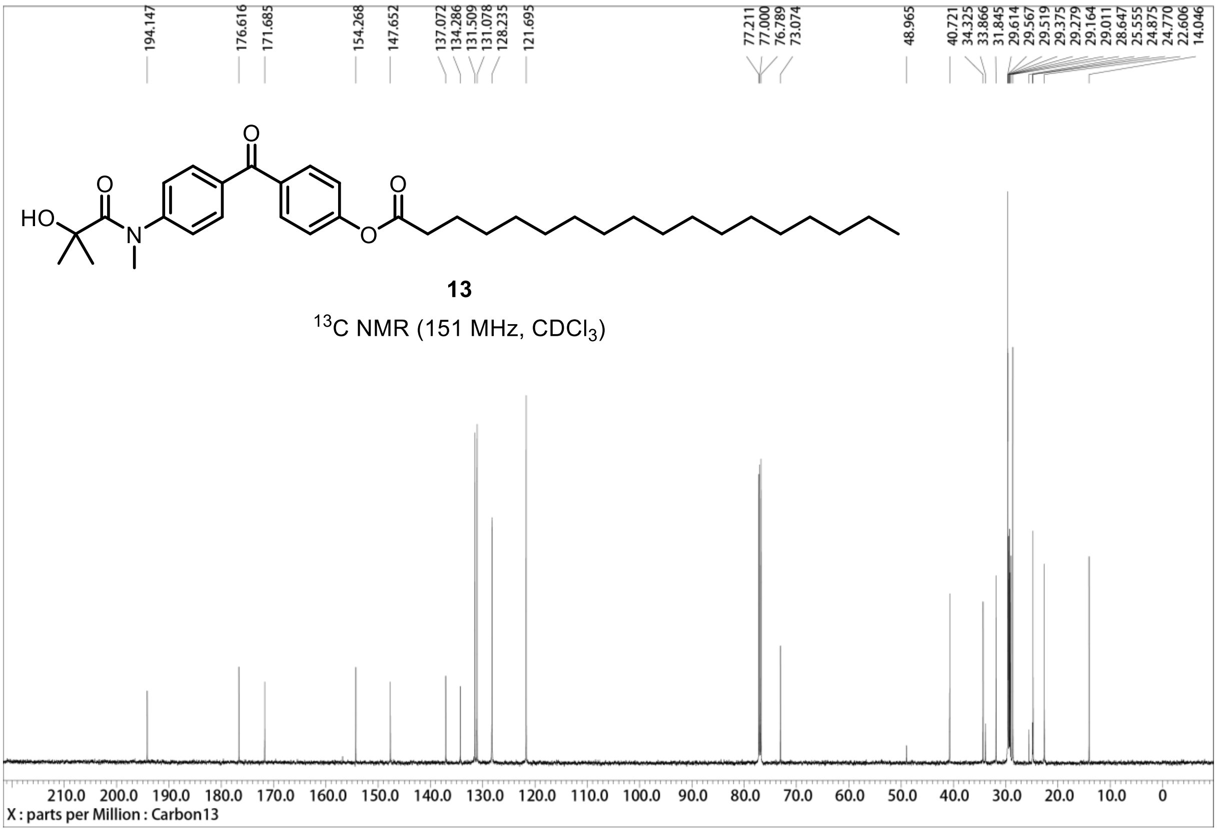

194.147
176.616
171.685
154.268
147.652
137.072
134.286
131.509
131.078
128.235
121.695
77.211
77.000
76.789
73.074
48.965
40.721
34.325
33.866
31.845
29.614
29.567
29.519
29.375
29.279
29.164
29.011
28.647
25.555
24.875
24.770
22.606
14.046
HO
O
N
O
O
O
13
13C NMR (151 MHz, CDCl3)
210.0 200.0 190.0 180.0 170.0 160.0 150.0 140.0 130.0 120.0 110.0 100.0 90.0 80.0 70.0 60.0 50.0 40.0 30.0 20.0 10.0 0
X : parts per Million : Carbon13

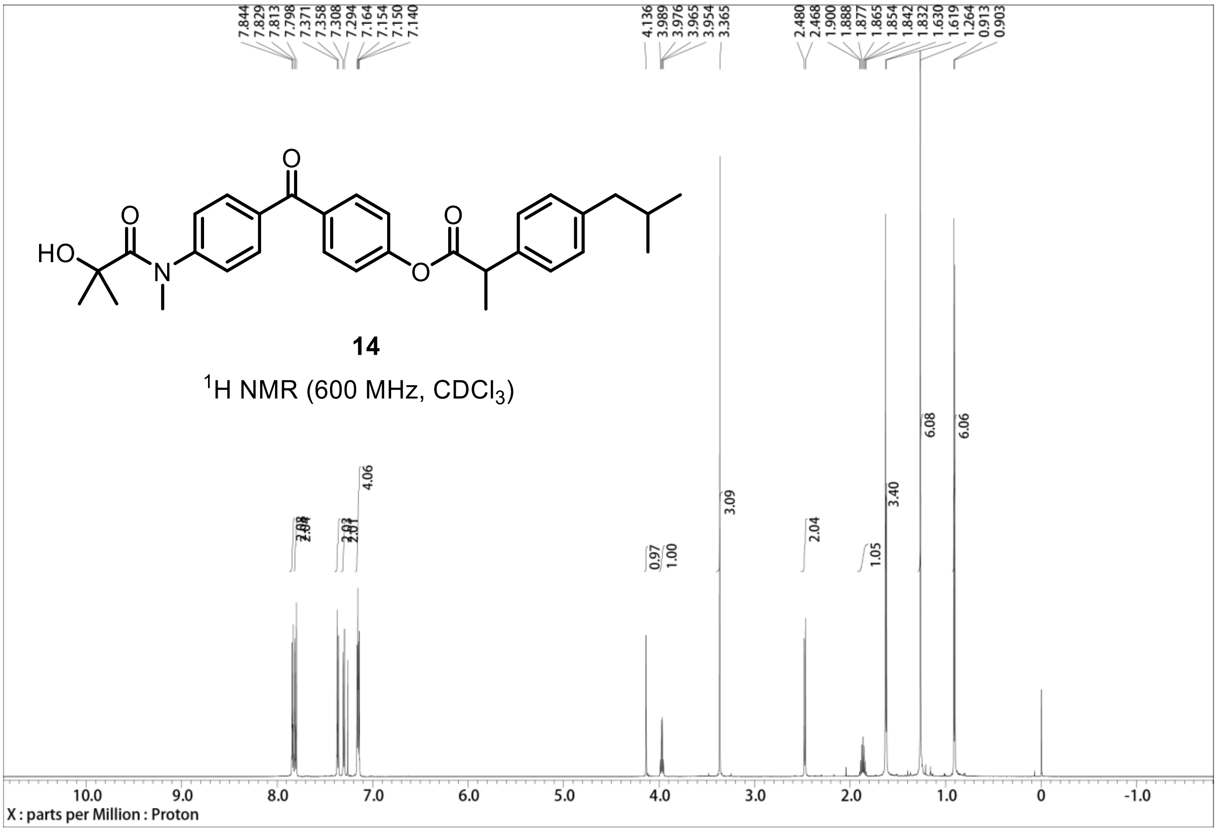
7.844 7.829 7.813 7.798 7.371 7.358 7.308 7.294 7.164 7.154 7.150 7.140
4.136 3.989 3.976 3.965 3.954 3.365
2.480 2.468 1.900 1.888 1.877 1.865 1.854 1.842 1.832 1.630 1.619 1.264 0.913 0.903
HO
O
N
O
O
O
14
1H NMR (600 MHz, CDCl3)
4.06
3.09
2.04
1.05
3.40
6.08
6.06
0.97
1.00
10.0 9.0 8.0 7.0 6.0 5.0 4.0 3.0 2.0 1.0 0 -1.0
X : parts per Million : Proton

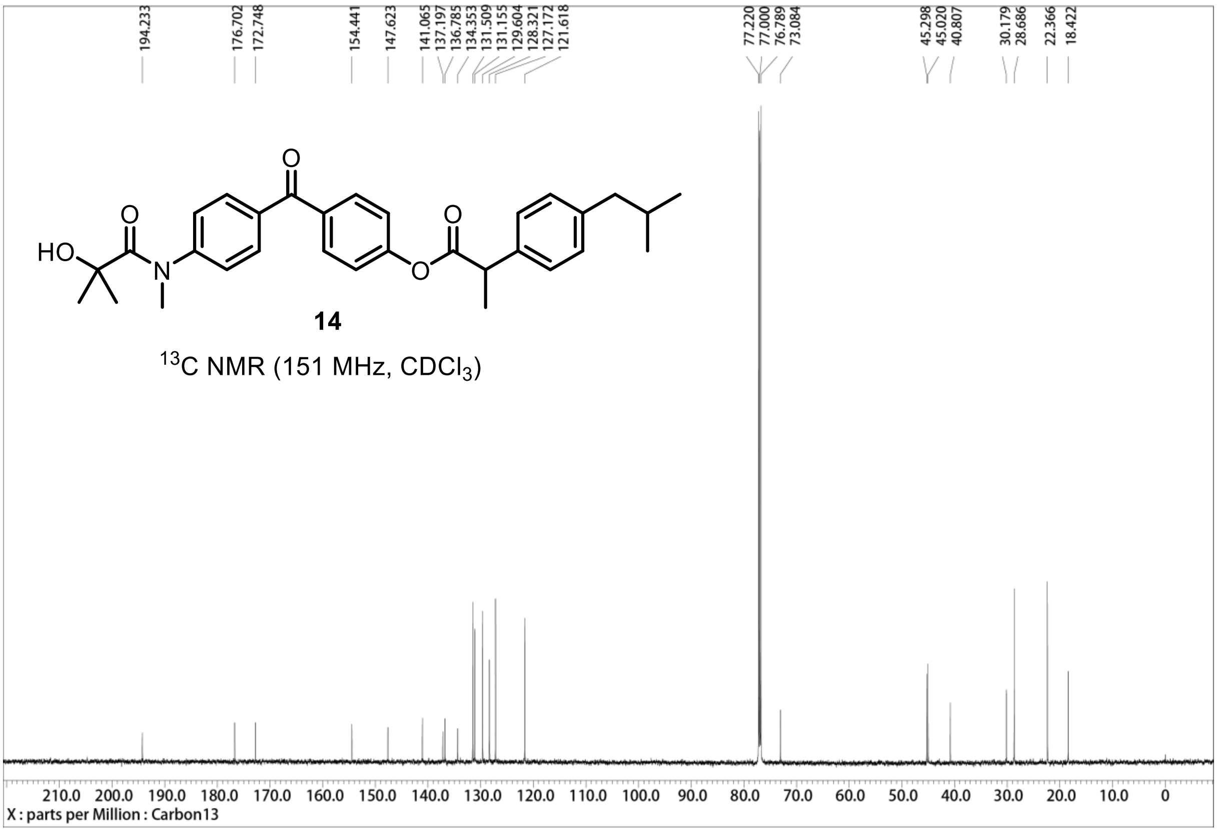
194.233
176.702 172.748
154.441 147.623 141.065 137.197 136.785 134.353 131.509 131.155 129.604 128.321 127.172 121.618
77.220 77.000 76.789 73.084
45.298 45.020 40.807
30.179 28.686 22.366 18.422
HO
O
N
O
O
O
14
13C NMR (151 MHz, CDCl3)
210.0 200.0 190.0 180.0 170.0 160.0 150.0 140.0 130.0 120.0 110.0 100.0 90.0 80.0 70.0 60.0 50.0 40.0 30.0 20.0 10.0 0
X : parts per Million : Carbon13

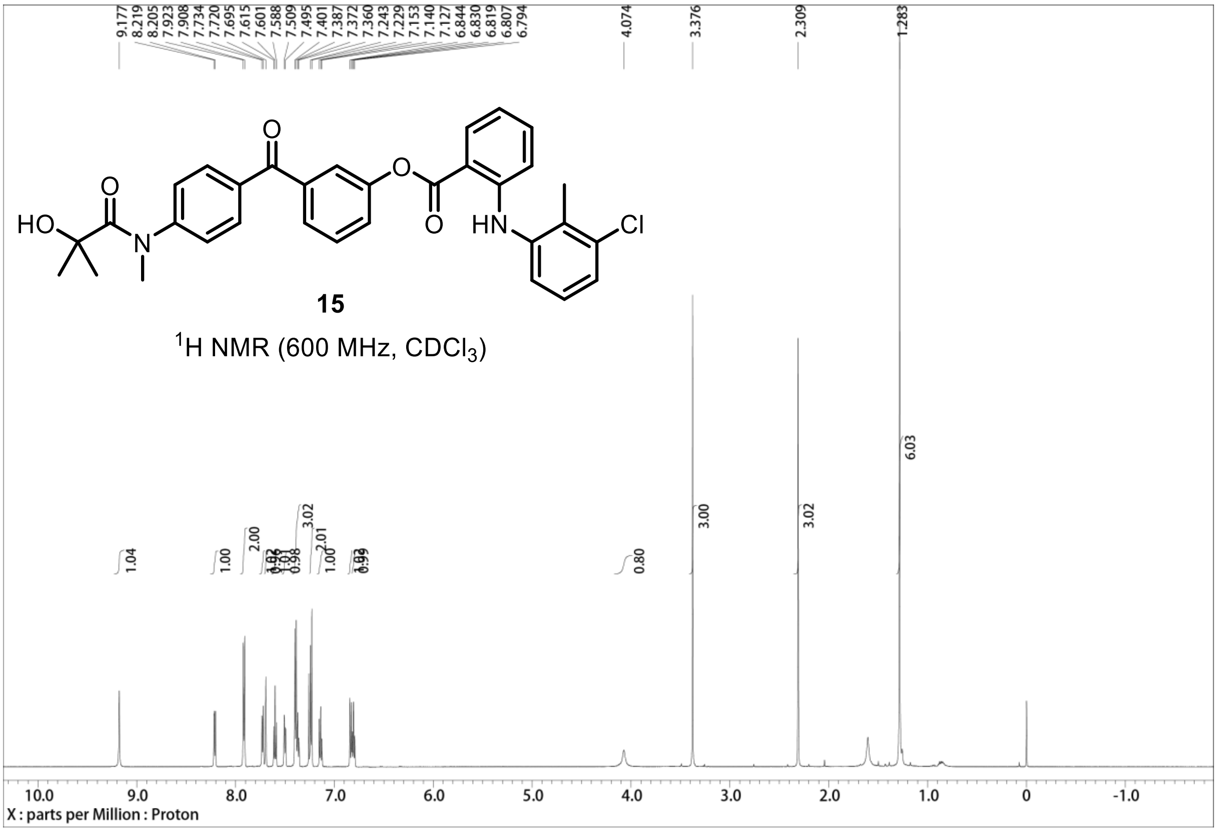
9.177 8.219 8.205 7.923 7.908 7.734 7.720 7.695 7.615 7.601 7.588 7.509 7.495 7.401 7.387 7.372 7.360 7.243 7.229 7.153 7.140 7.127 6.844 6.830 6.819 6.807 6.794
4.074
3.376
2.309
1.283
HO
O
N
O
O
O
HN
Cl
15
1H NMR (600 MHz, CDCl3)
1.04
1.00
2.00
3.02
2.01
1.00
0.80
3.00
3.02
6.03
10.0 9.0 8.0 7.0 6.0 5.0 4.0 3.0 2.0 1.0 0 -1.0
X : parts per Million : Proton

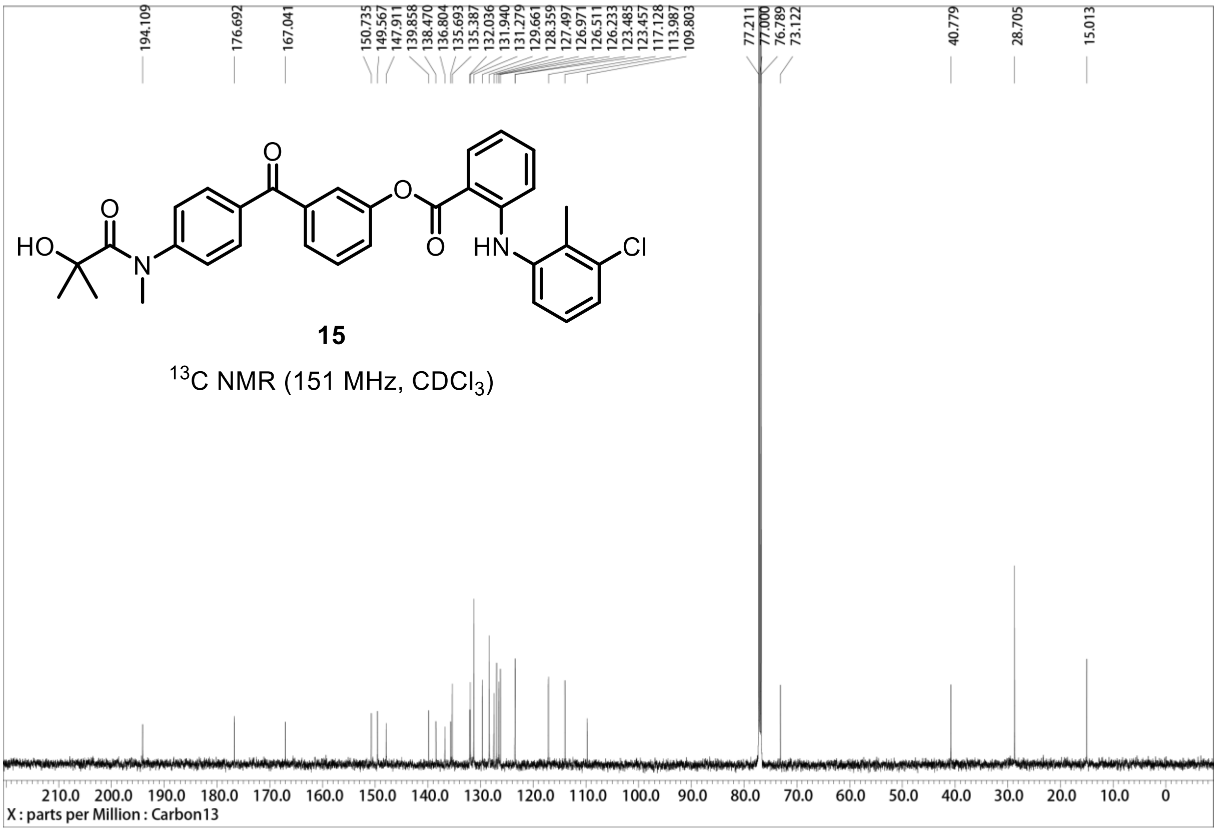
194.109
176.692
167.041
150.735 149.567 147.911 139.858 138.470 136.804 135.693 135.387 132.036 131.940 131.279 129.661 128.359 127.497 126.971 126.511 126.233 123.485 123.457 117.128 113.987 109.803
77.211 77.000 76.789 73.122
40.779
28.705
15.013
HO
O
N
O
O
O
HN
Cl
15
13C NMR (151 MHz, CDCl3)
210.0 200.0 190.0 180.0 170.0 160.0 150.0 140.0 130.0 120.0 110.0 100.0 90.0 80.0 70.0 60.0 50.0 40.0 30.0 20.0 10.0 0
X : parts per Million : Carbon13

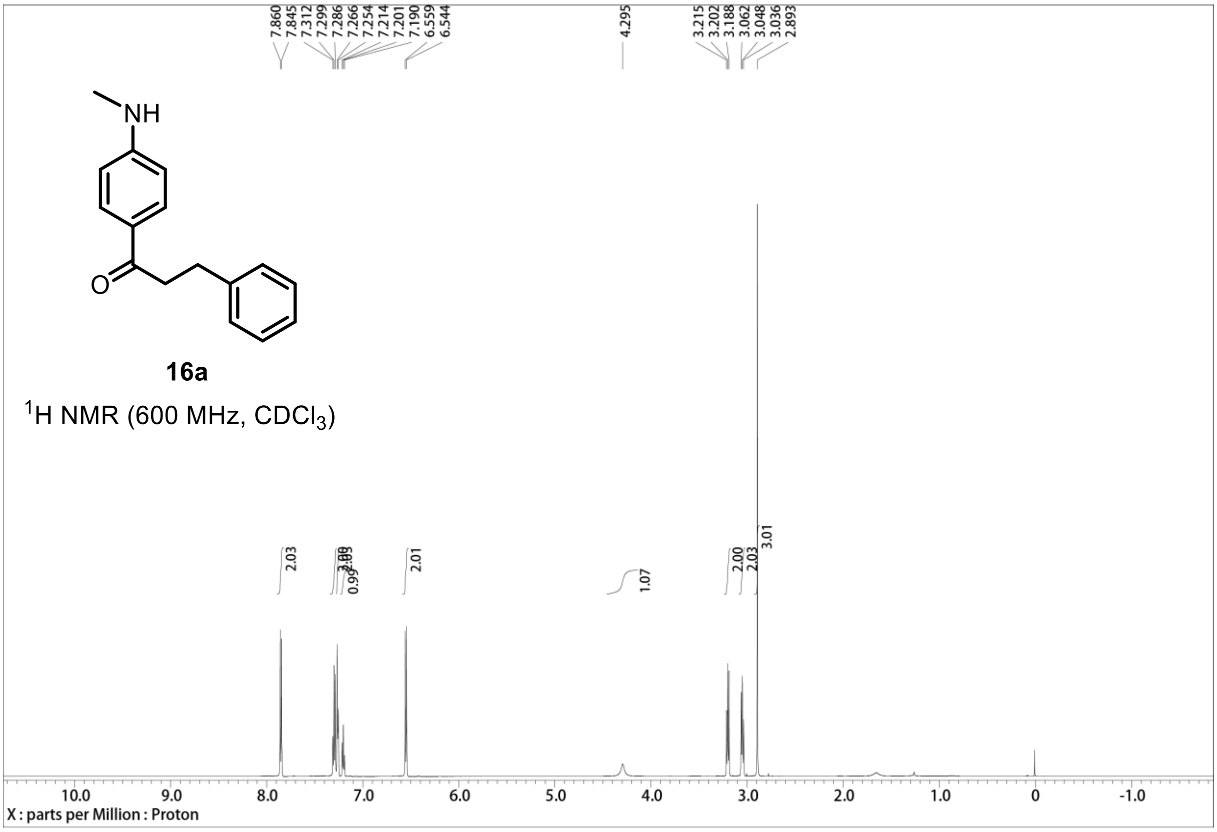
7.860
7.845
7.312
7.299
7.286
7.266
7.254
7.214
7.201
7.190
6.559
6.544
4.295
3.215
3.202
3.188
3.062
3.048
3.036
2.893
NH
O
16a
1H NMR (600 MHz, CDCl3)
2.03
2.01
1.07
2.00
2.03
3.01
X : parts per Million : Proton

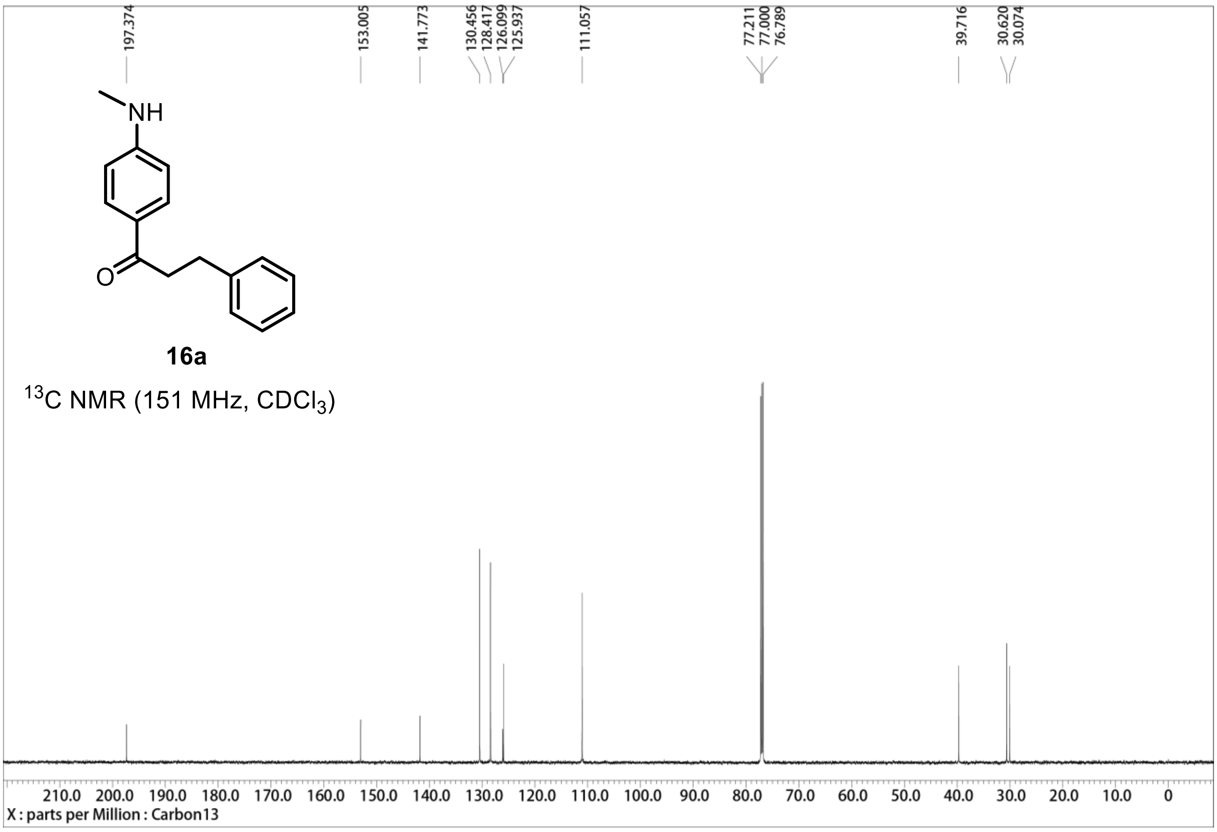
197.374
153.005
141.773
130.456
128.417
126.099
125.937
111.057
77.211
77.000
76.789
39.716
30.620
30.074
NH
O
16a
13C NMR (151 MHz, CDCl3)
X : parts per Million : Carbon13

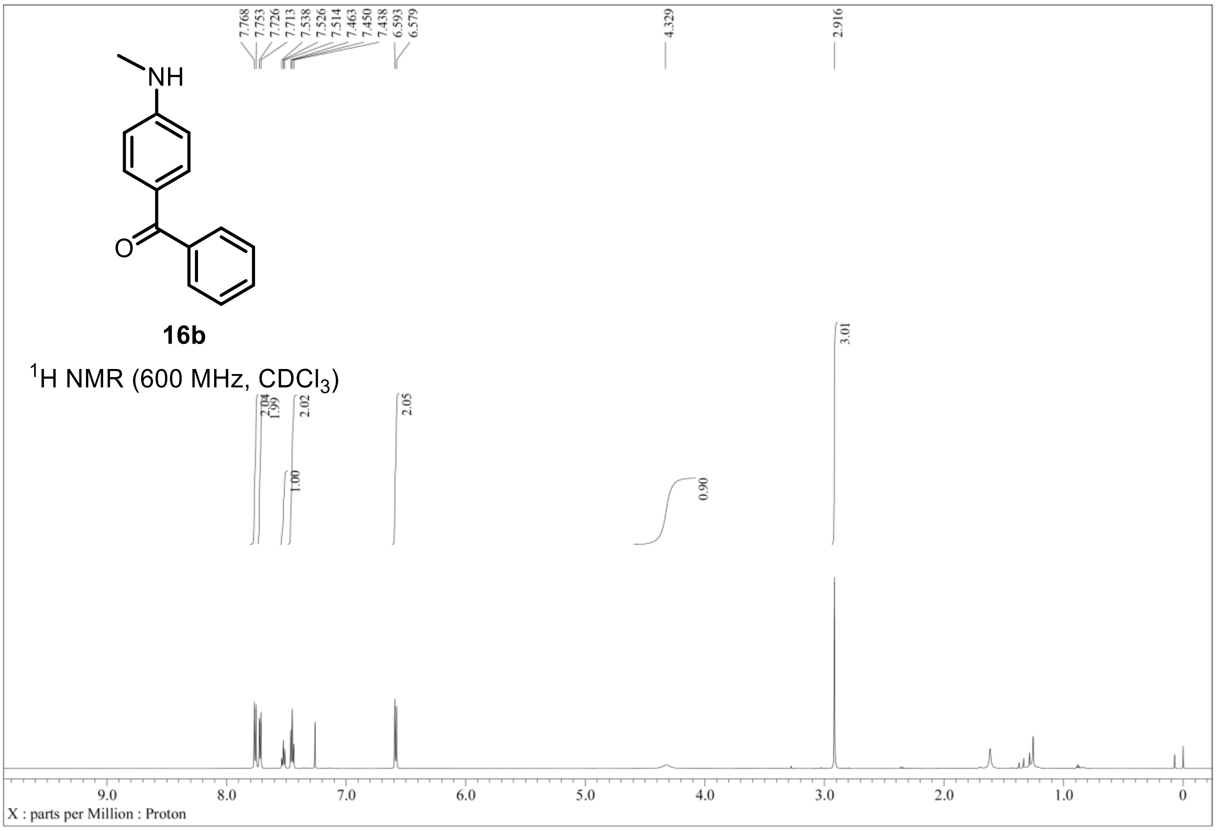

7.768
7.753
7.726
7.713
7.538
7.526
7.514
7.463
7.450
7.438
6.593
6.579
4.329
2.916
NH
O
16b
^{1}H NMR (600 MHz, CDCl$_3$)
2.04
1.99
2.02
1.00
2.05
0.90
3.01
9.0
8.0
7.0
6.0
5.0
4.0
3.0
2.0
1.0
0
X : parts per Million : Proton

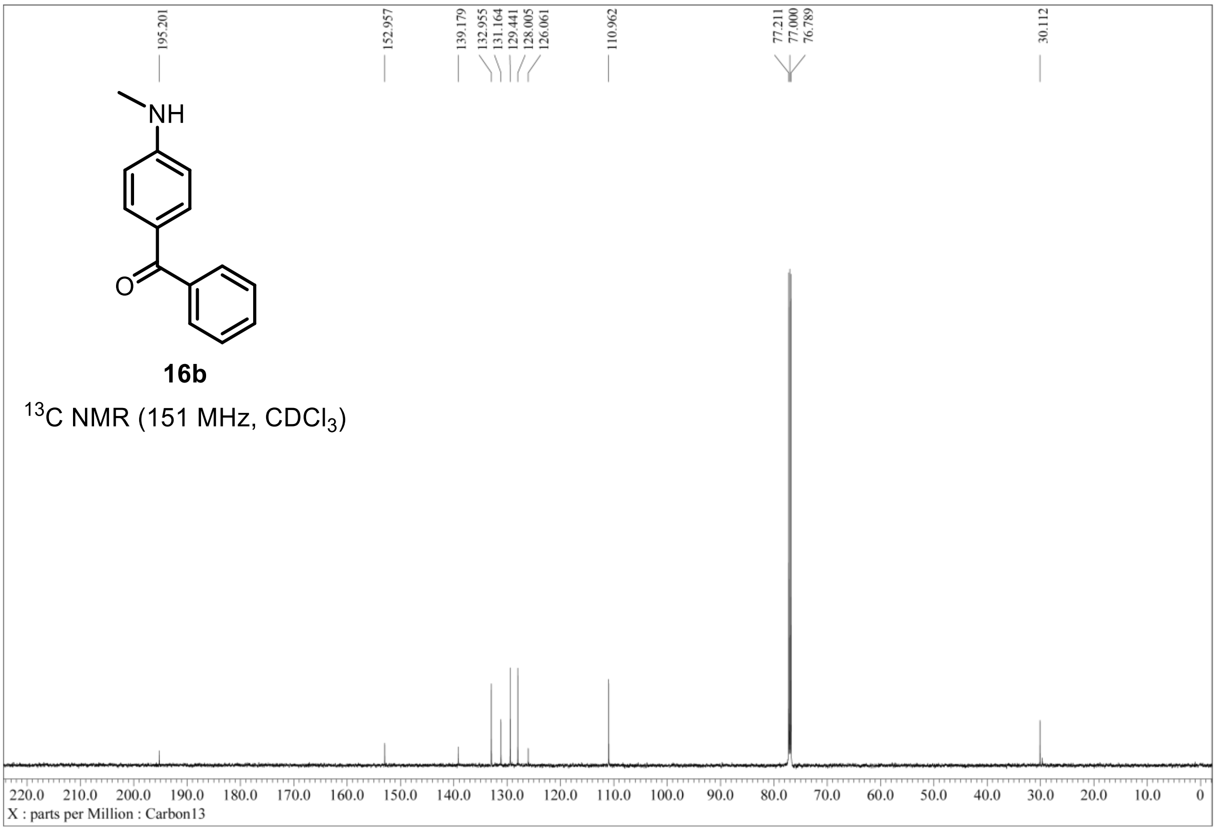

195.201
152.957
139.179
132.955
131.164
129.441
128.005
126.061
110.962
77.211
77.000
76.789
30.112
NH
O
16b
^{13}C NMR (151 MHz, CDCl$_3$)
220.0
210.0
200.0
190.0
180.0
170.0
160.0
150.0
140.0
130.0
120.0
110.0
100.0
90.0
80.0
70.0
60.0
50.0
40.0
30.0
20.0
10.0
0
X : parts per Million : Carbon13

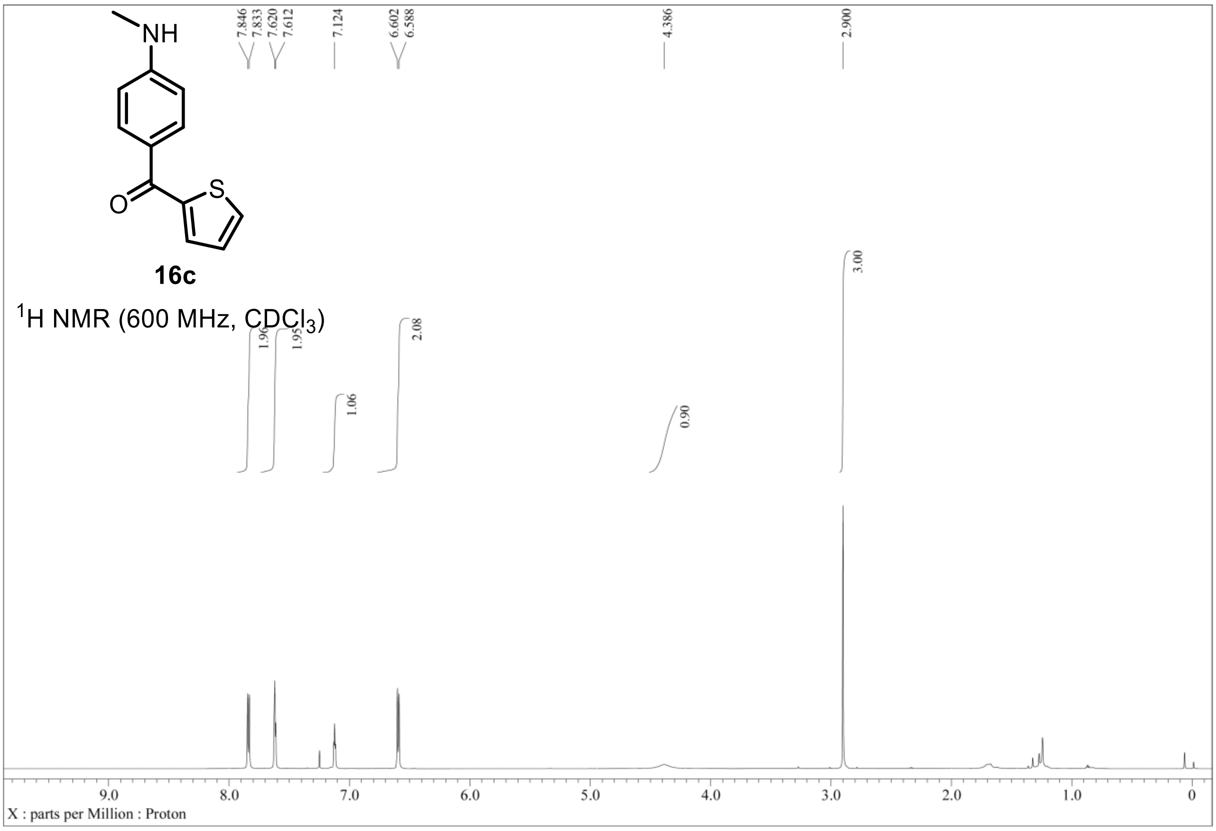

16c

^{1}H NMR (600 MHz, $CDCl_3$)

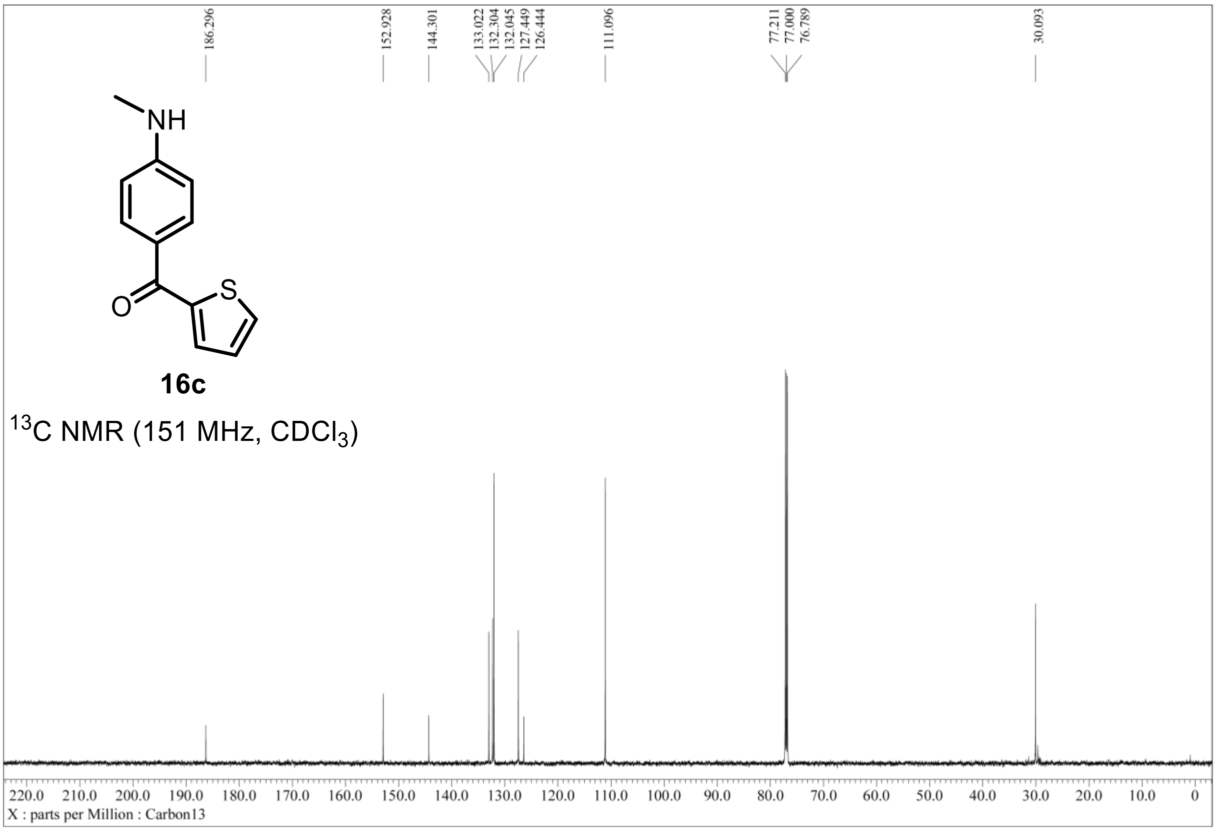

16c

^{13}C NMR (151 MHz, $CDCl_3$)

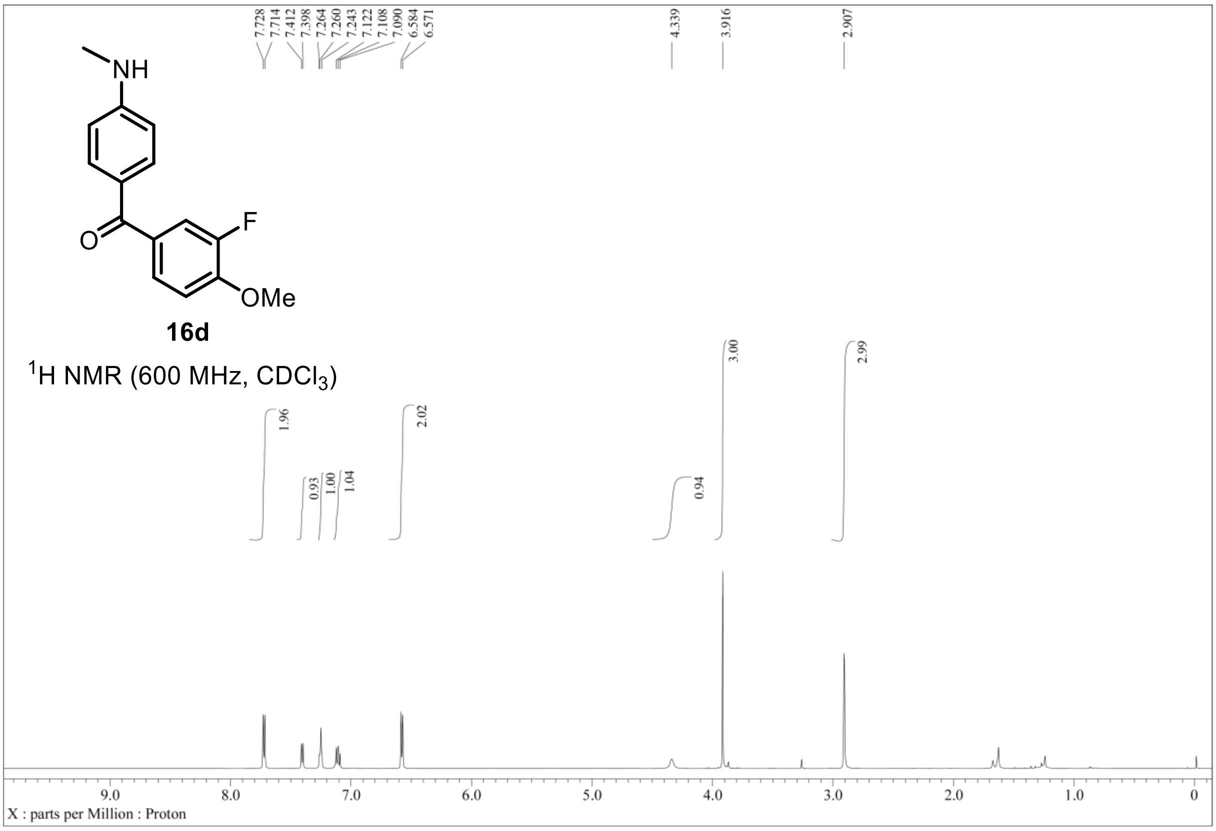
NH
F
O
OMe
16d
^{1}H NMR (600 MHz, CDCl$_3$)
7.728
7.714
7.412
7.398
7.264
7.260
7.243
7.122
7.108
7.090
6.584
6.571
4.339
3.916
2.907
1.96
0.93
1.00
1.04
2.02
0.94
3.00
2.99
9.0
8.0
7.0
6.0
5.0
4.0
3.0
2.0
1.0
0
X : parts per Million : Proton

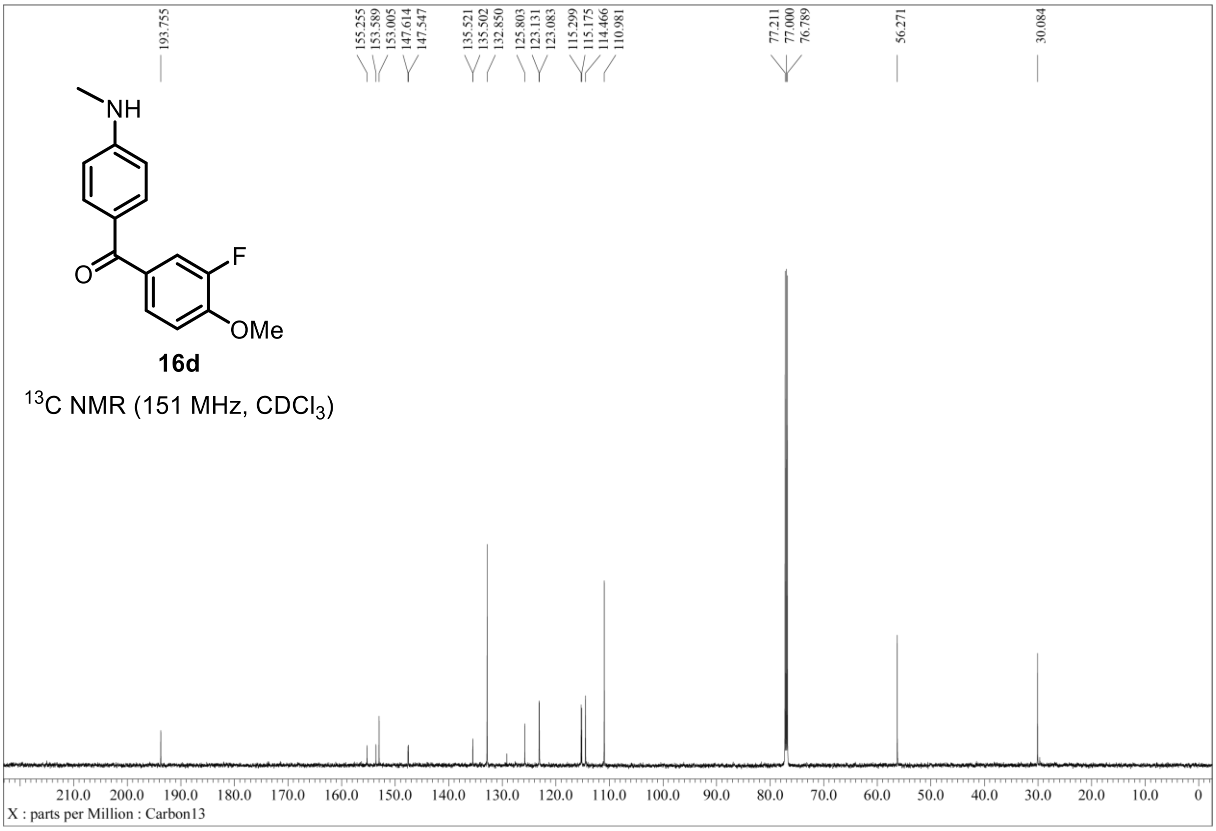
NH
F
O
OMe
16d
^{13}C NMR (151 MHz, CDCl$_3$)
193.755
155.255
153.589
153.005
147.614
147.547
135.521
135.502
132.850
125.803
123.131
123.083
115.299
115.175
114.466
110.981
77.211
77.000
76.789
56.271
30.084
210.0
200.0
190.0
180.0
170.0
160.0
150.0
140.0
130.0
120.0
110.0
100.0
90.0
80.0
70.0
60.0
50.0
40.0
30.0
20.0
10.0
0
X : parts per Million : Carbon13

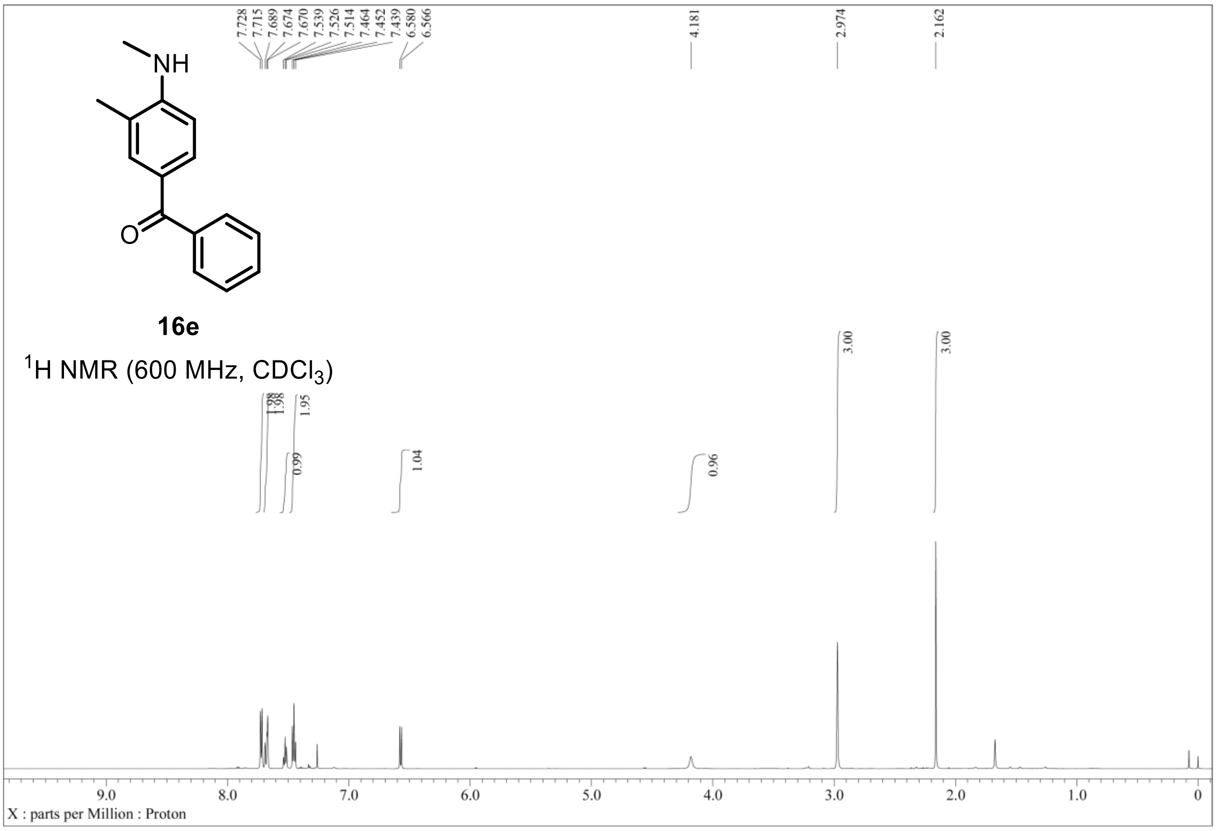

16e

^{1}H NMR (600 MHz, $CDCl_3$)

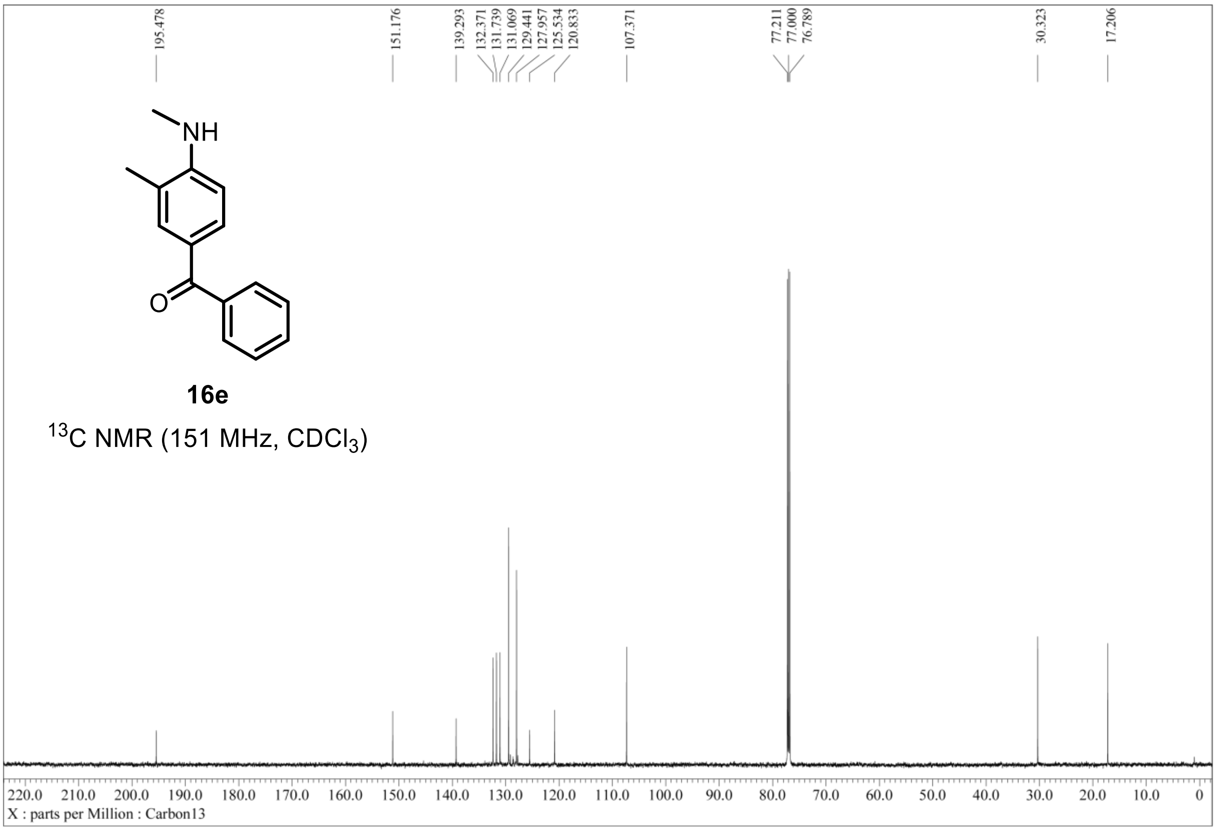

16e

^{13}C NMR (151 MHz, $CDCl_3$)

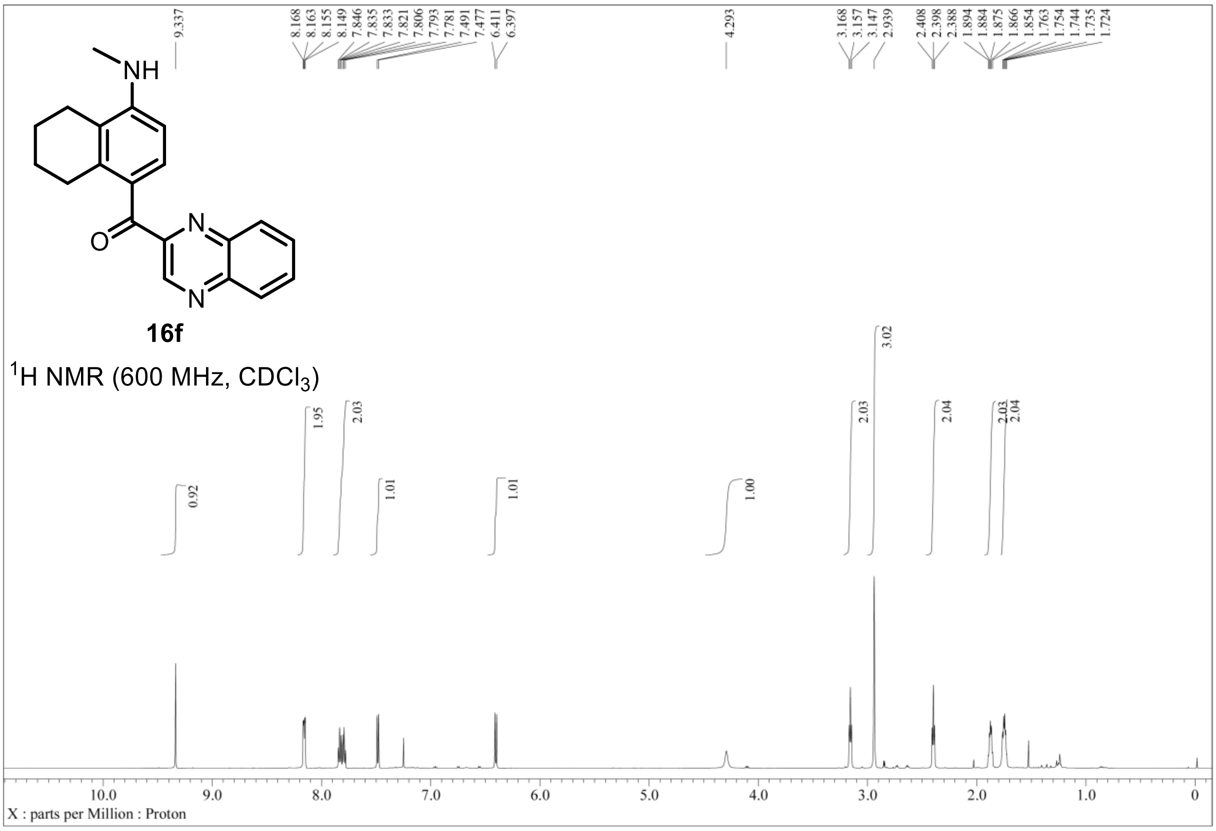
16f
^{1}H NMR (600 MHz, $CDCl_3$)
X : parts per Million : Proton

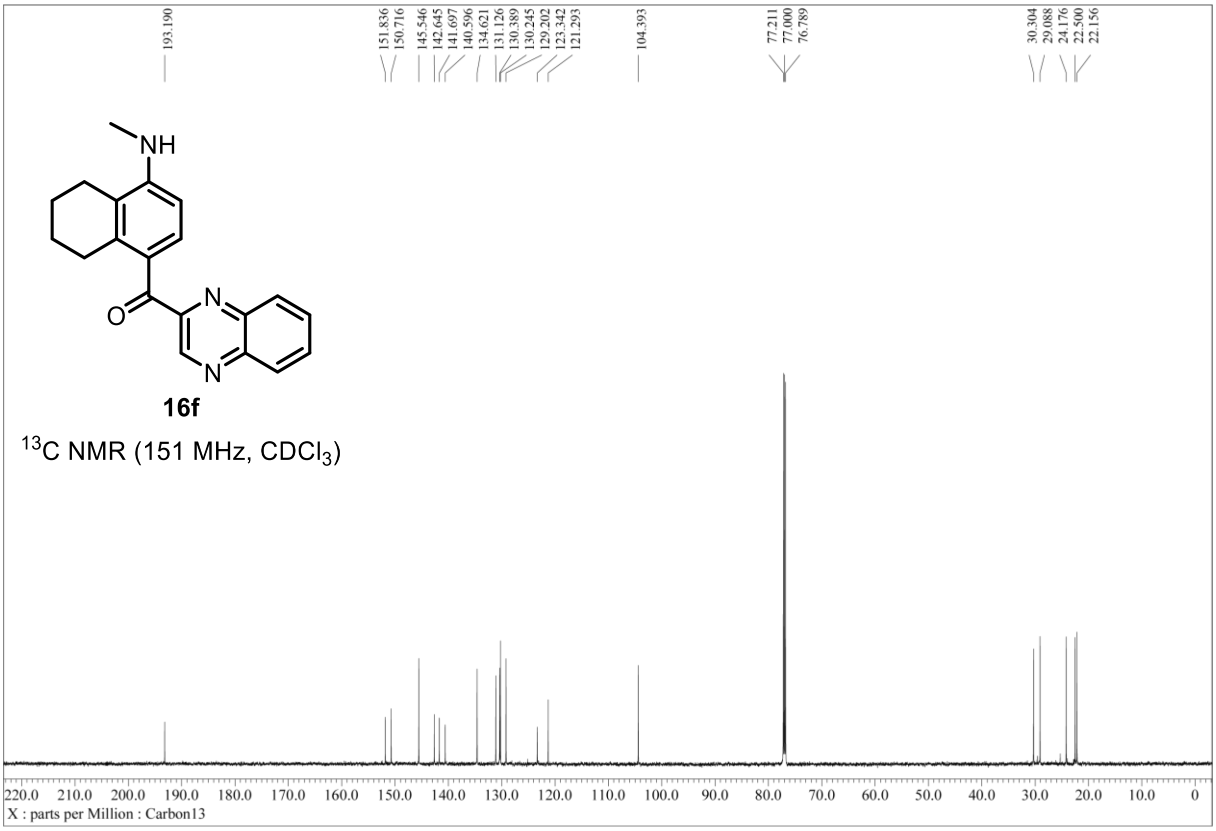
16f
^{13}C NMR (151 MHz, $CDCl_3$)
X : parts per Million : Carbon13

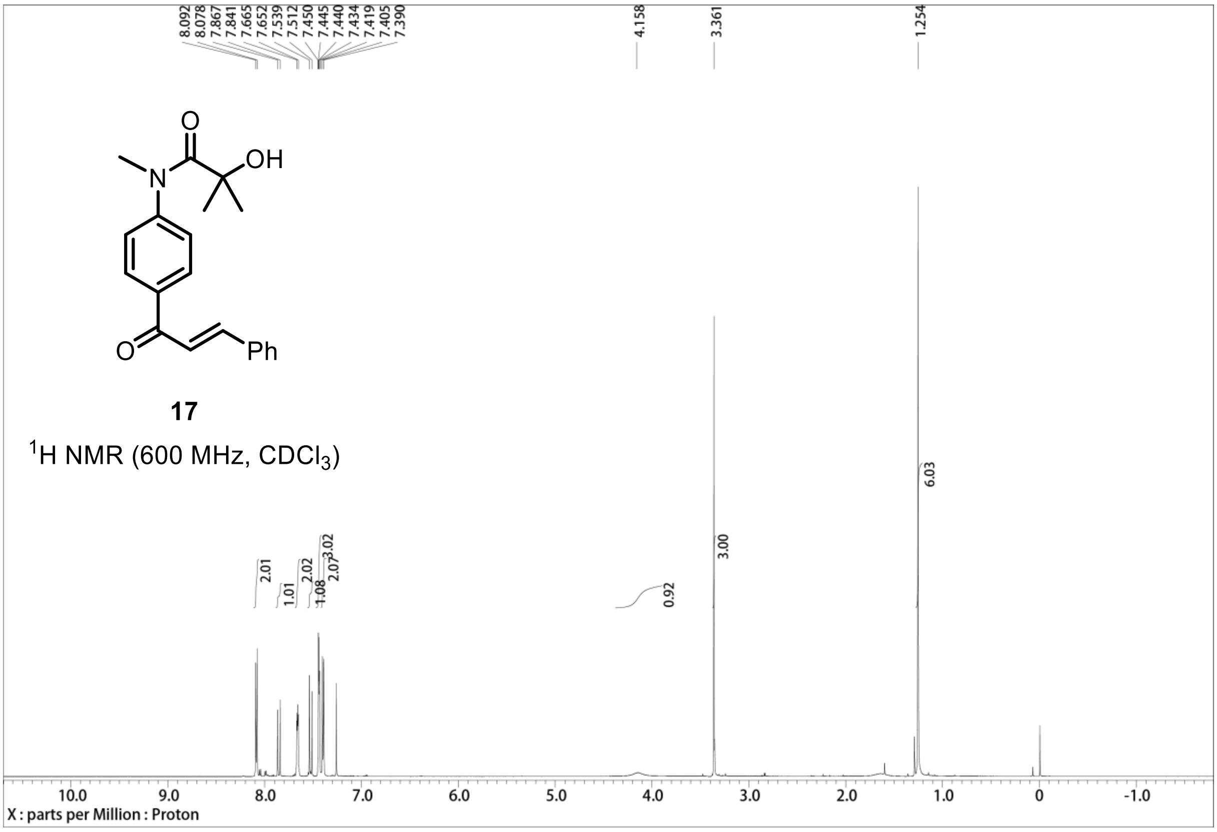
8.092
8.078
7.867
7.841
7.665
7.652
7.539
7.512
7.450
7.445
7.440
7.434
7.419
7.405
7.390
4.158
3.361
1.254
OH
O
N
O
Ph
17
^{1}H NMR (600 MHz, CDCl$_3$)
2.01
1.01
2.02
1.08
2.07
3.02
0.92
3.00
6.03
10.0 9.0 8.0 7.0 6.0 5.0 4.0 3.0 2.0 1.0 0 -1.0
X : parts per Million : Proton

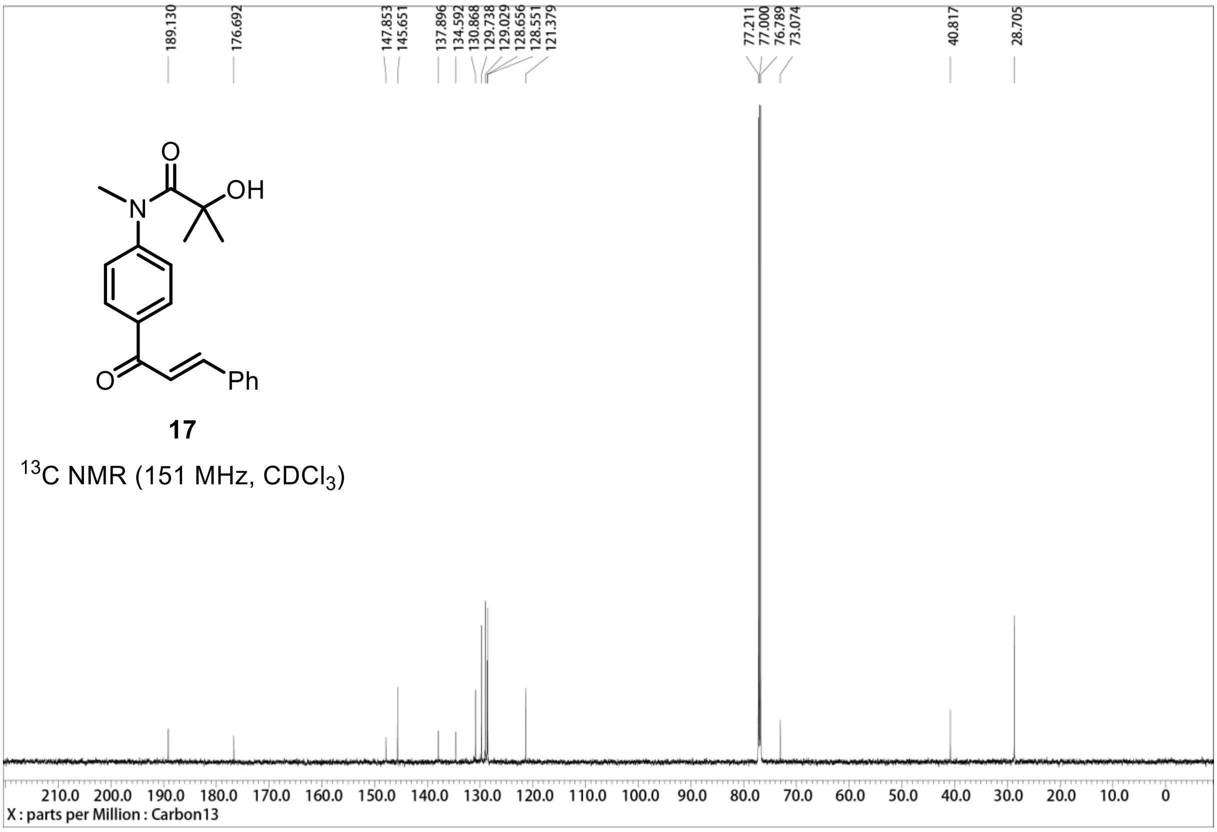
189.130
176.692
147.853
145.651
137.896
134.592
130.868
129.738
129.029
128.656
128.551
121.379
77.211
77.000
76.789
73.074
40.817
28.705
OH
O
N
O
Ph
17
^{13}C NMR (151 MHz, CDCl$_3$)
210.0 200.0 190.0 180.0 170.0 160.0 150.0 140.0 130.0 120.0 110.0 100.0 90.0 80.0 70.0 60.0 50.0 40.0 30.0 20.0 10.0 0
X : parts per Million : Carbon13

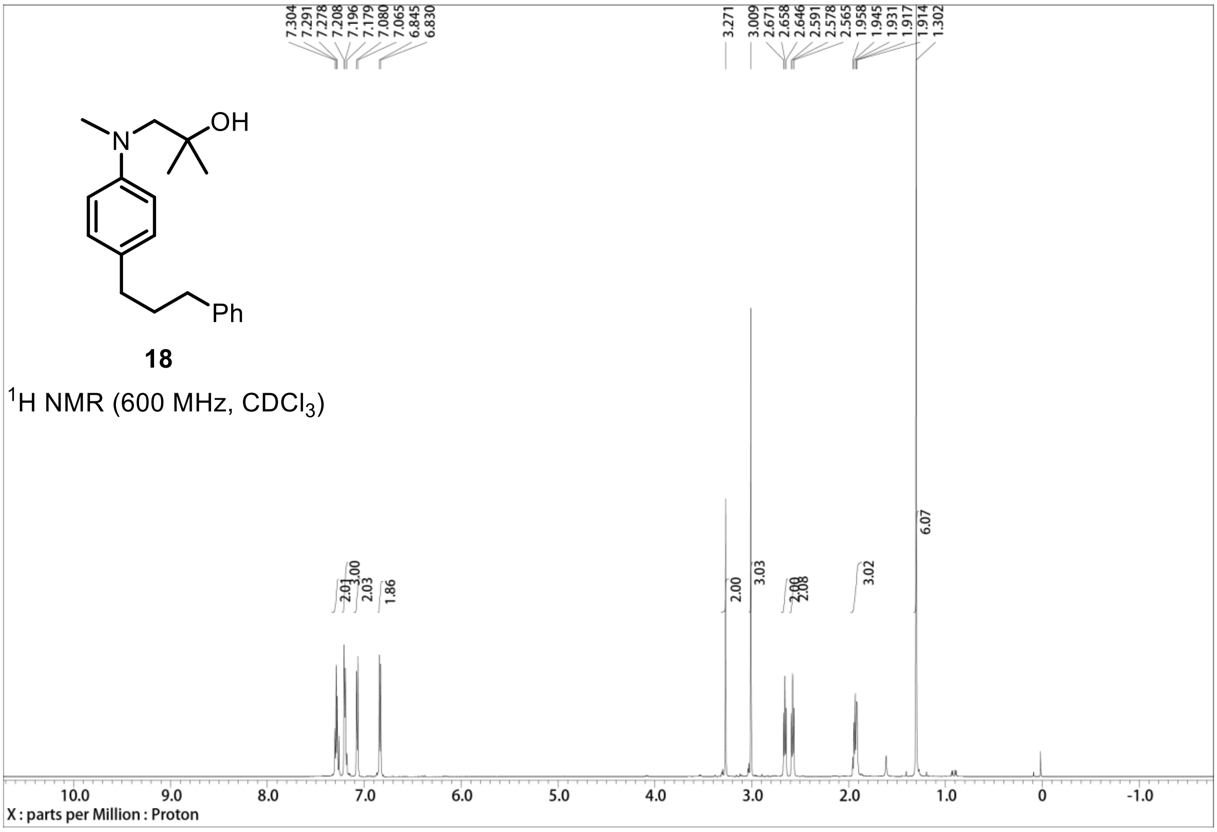

7.304
7.291
7.278
7.208
7.196
7.179
7.080
7.065
6.845
6.830
3.271
3.009
2.671
2.658
2.646
2.591
2.578
2.565
1.958
1.945
1.931
1.917
1.914
1.302
OH
N
Ph
18
^{1}H NMR (600 MHz, CDCl$_3$)
2.01
3.00
2.03
1.86
2.00
3.03
2.00
2.08
3.02
6.07
X : parts per Million : Proton

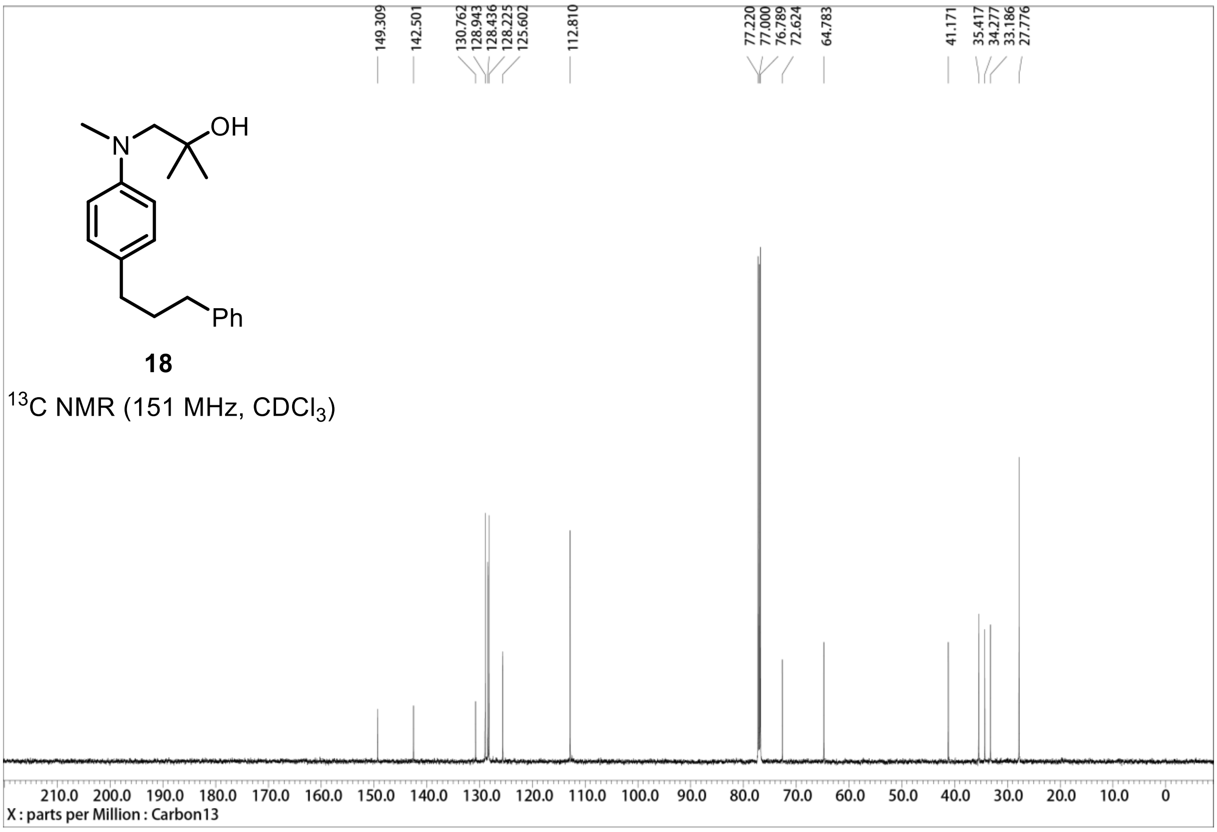

149.309
142.501
130.762
128.943
128.436
128.225
125.602
112.810
77.220
77.000
76.789
72.624
64.783
41.171
35.417
34.277
33.186
27.776
OH
N
Ph
18
^{13}C NMR (151 MHz, CDCl$_3$)
X : parts per Million : Carbon13

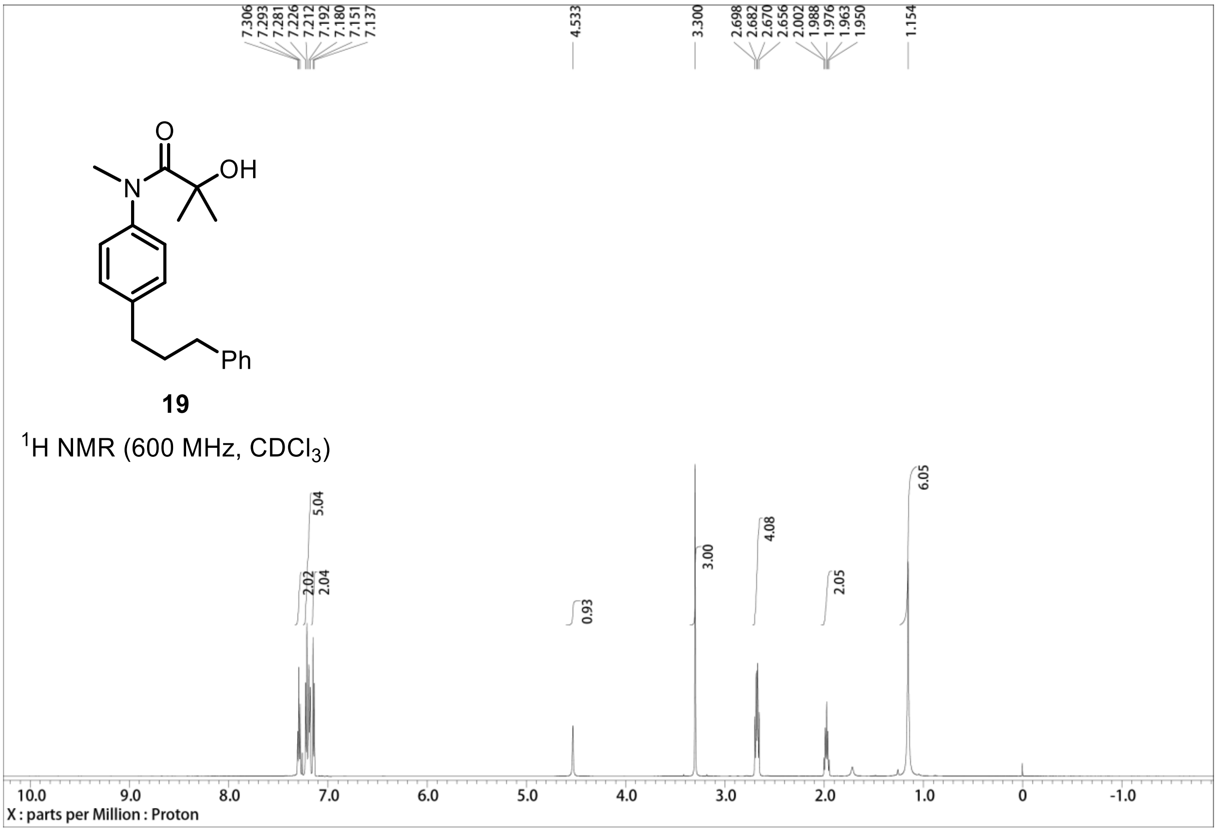
7.306
7.293
7.281
7.226
7.212
7.192
7.180
7.151
7.137
4.533
3.300
2.698
2.682
2.670
2.656
2.002
1.988
1.976
1.963
1.950
1.154
O
N
OH
Ph
19
1H NMR (600 MHz, CDCl3)
5.04
2.02
2.04
0.93
3.00
4.08
2.05
6.05
10.0
9.0
8.0
7.0
6.0
5.0
4.0
3.0
2.0
1.0
0
-1.0
X : parts per Million : Proton

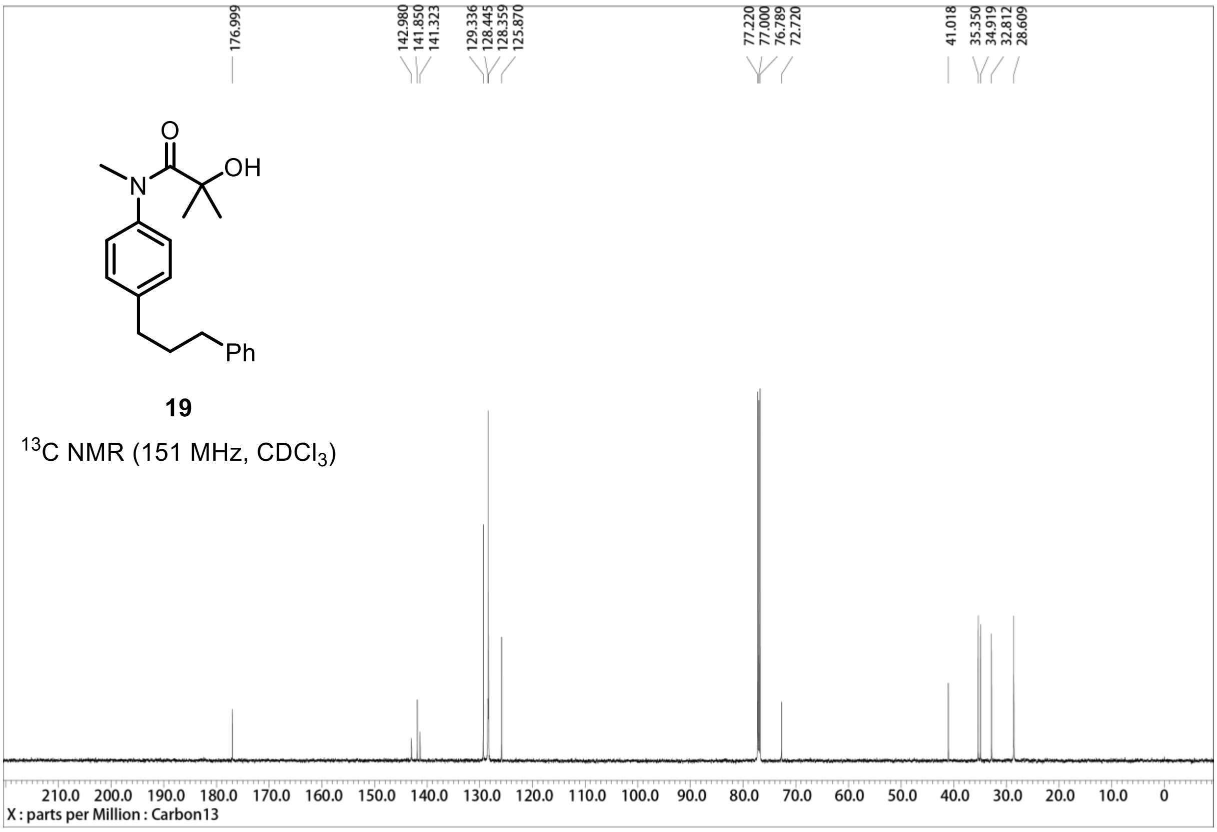
176.999
142.980
141.850
141.323
129.336
128.445
128.359
125.870
77.220
77.000
76.789
72.720
41.018
35.350
34.919
32.812
28.609
O
N
OH
Ph
19
13C NMR (151 MHz, CDCl3)
210.0
200.0
190.0
180.0
170.0
160.0
150.0
140.0
130.0
120.0
110.0
100.0
90.0
80.0
70.0
60.0
50.0
40.0
30.0
20.0
10.0
0
X : parts per Million : Carbon13

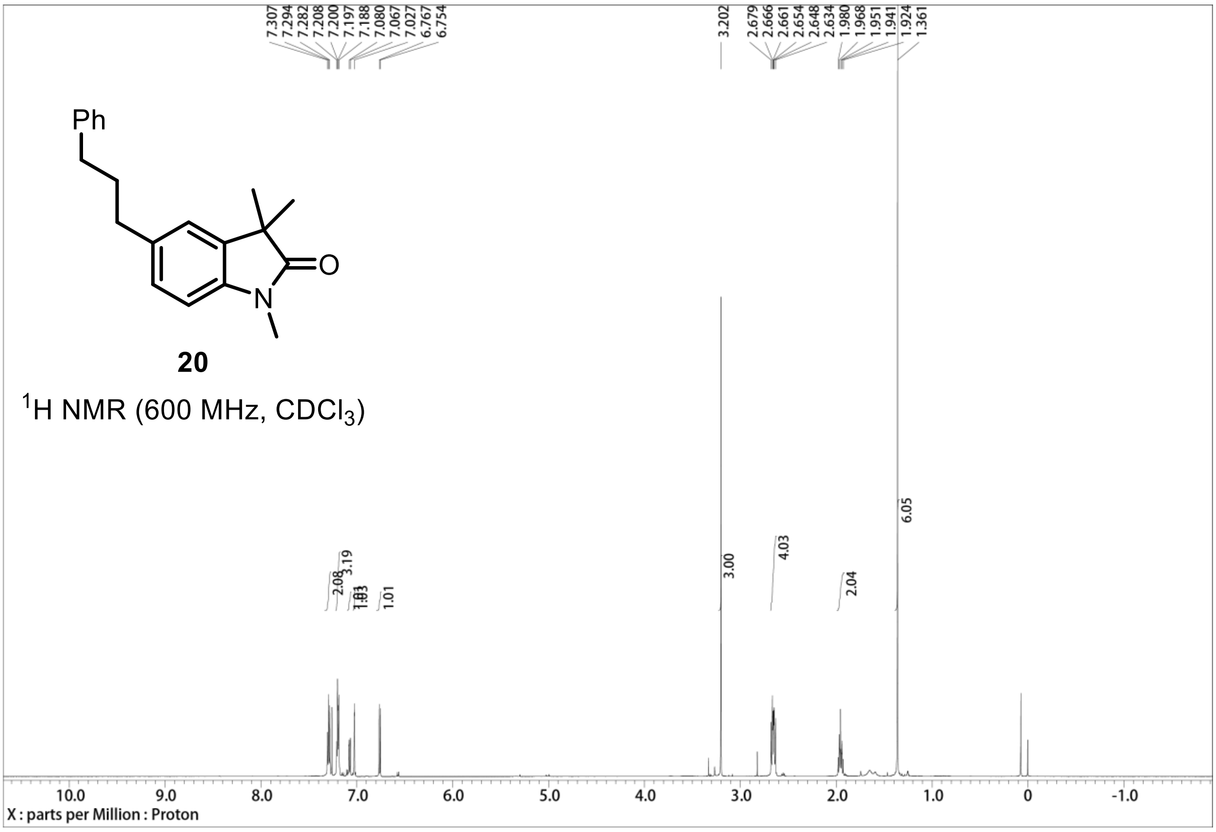

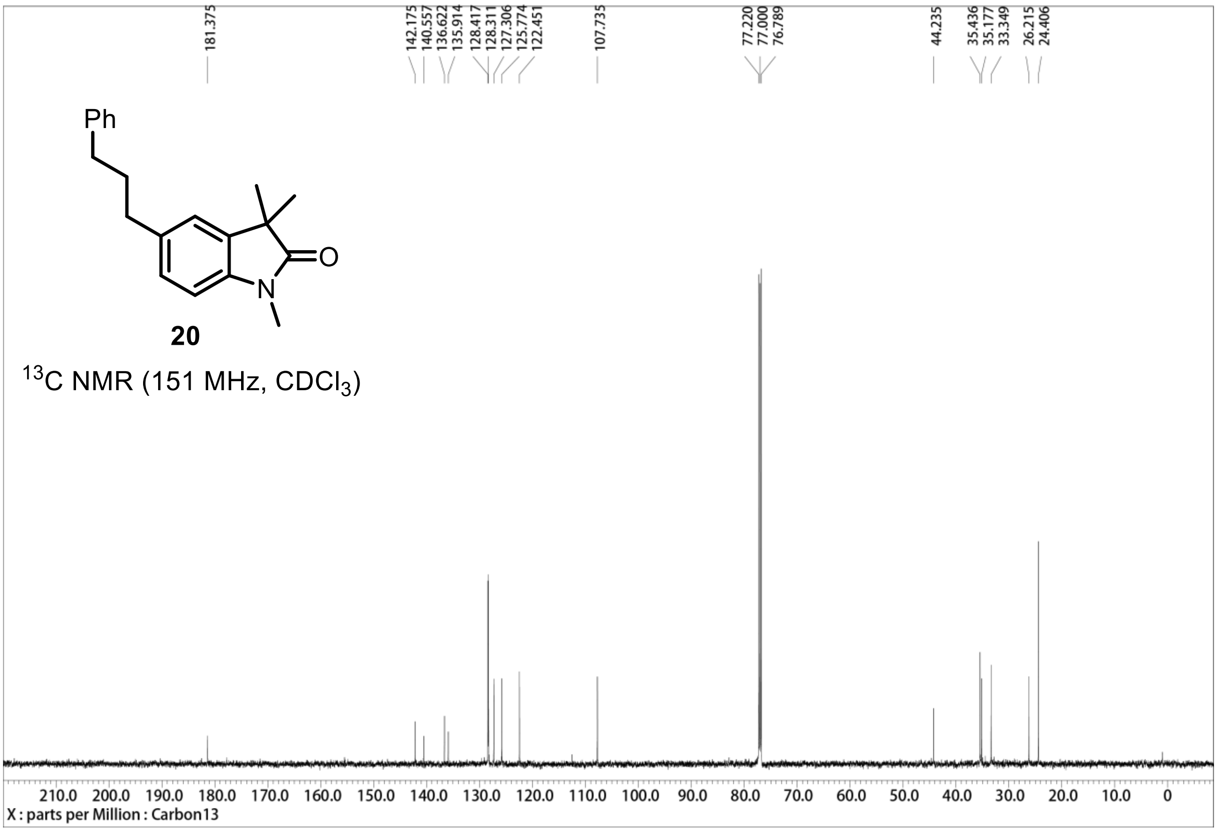

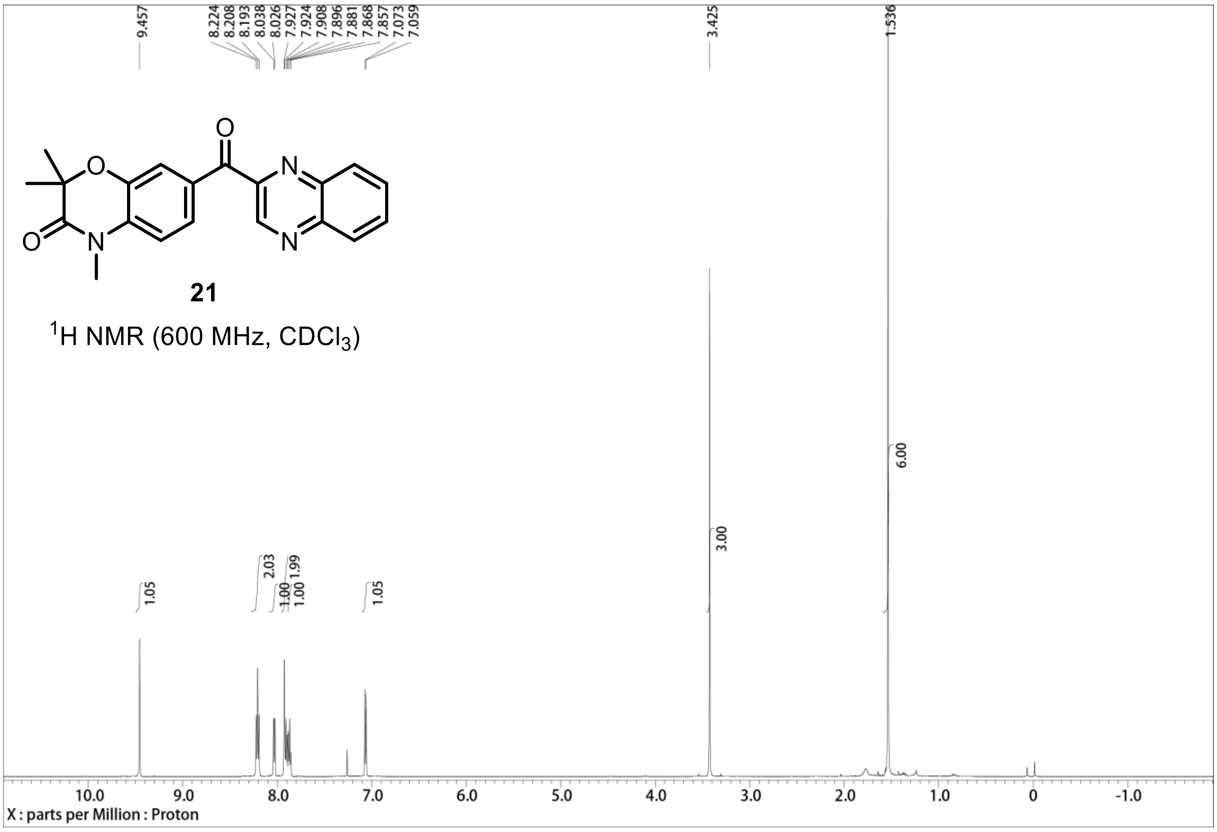
9.457
8.224 8.208 8.193 8.038 8.026 7.927 7.924 7.908 7.896 7.881 7.868 7.857 7.073 7.059
3.425
1.536
O
O
N
O
N
N
21
1H NMR (600 MHz, CDCl3)
1.05
2.03
1.00
1.00 1.99
1.05
3.00
6.00
10.0 9.0 8.0 7.0 6.0 5.0 4.0 3.0 2.0 1.0 0 -1.0
X : parts per Million : Proton

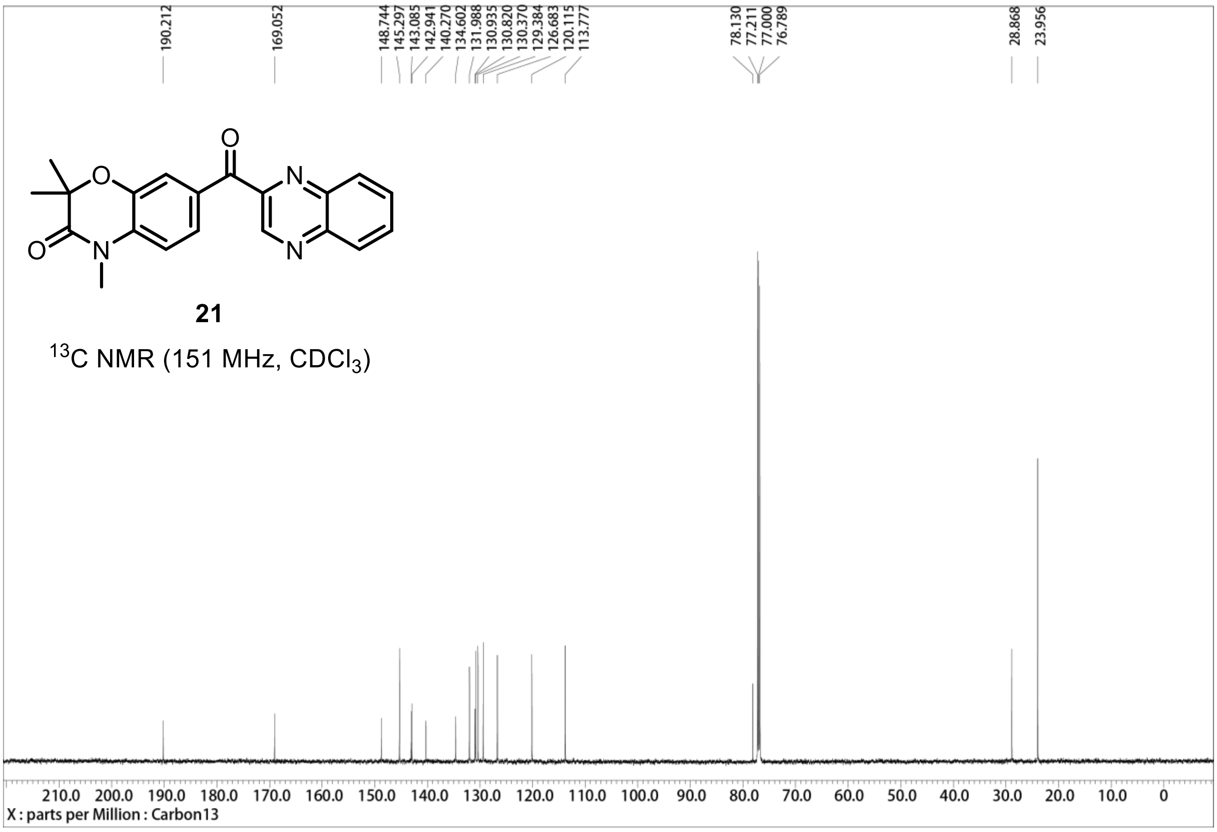
190.212
169.052
148.744 145.297 143.085 142.941 140.270 134.602 131.988 130.935 130.820 130.370 129.384 126.683 120.115 113.777
78.130 77.211 77.000 76.789
28.868 23.956
O
O
N
O
N
N
21
13C NMR (151 MHz, CDCl3)
210.0 200.0 190.0 180.0 170.0 160.0 150.0 140.0 130.0 120.0 110.0 100.0 90.0 80.0 70.0 60.0 50.0 40.0 30.0 20.0 10.0 0
X : parts per Million : Carbon13

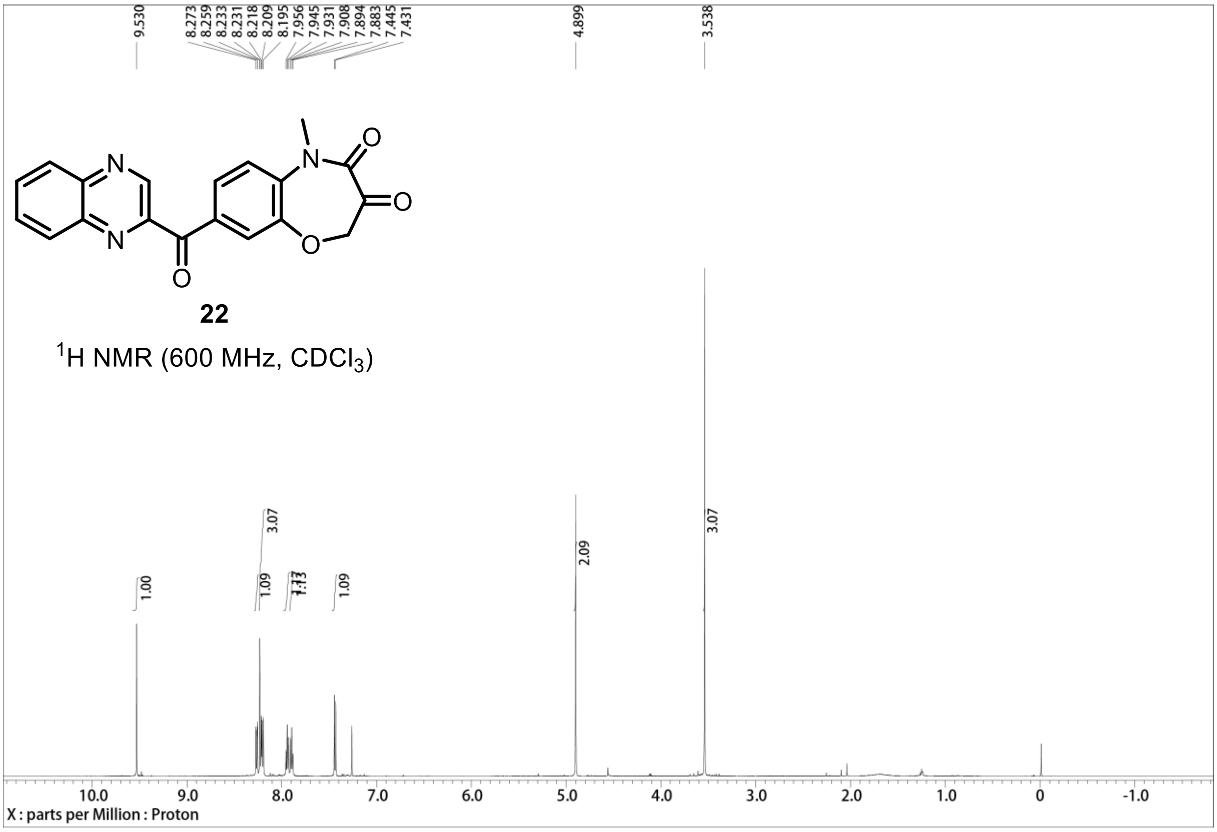
9.530
8.273 8.259 8.233 8.231 8.218 8.209 8.195 7.956 7.945 7.931 7.908 7.894 7.883 7.445 7.431
4.899
3.538
22
1H NMR (600 MHz, CDCl3)
1.00
1.09
3.07
1.13
1.09
2.09
3.07
10.0 9.0 8.0 7.0 6.0 5.0 4.0 3.0 2.0 1.0 0 -1.0
X : parts per Million : Proton

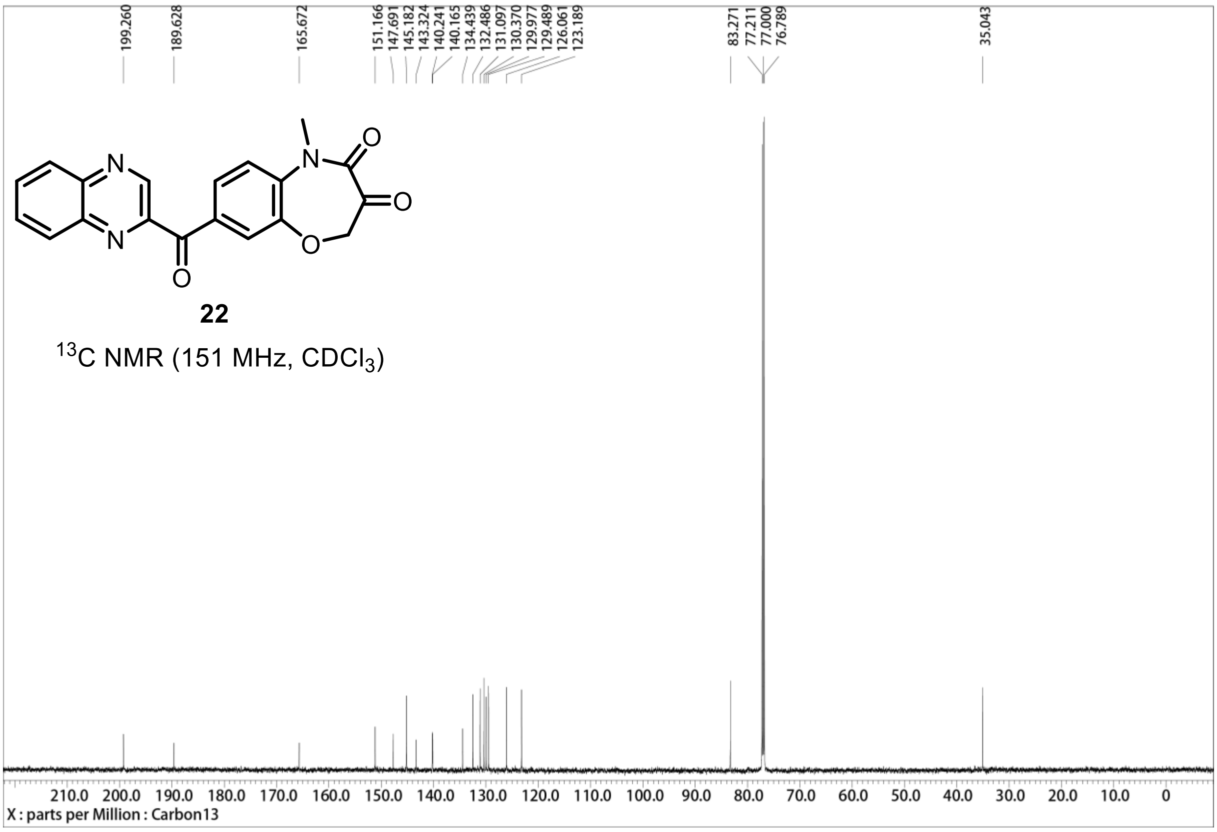
199.260
189.628
165.672
151.166 147.691 145.182 143.324 140.241 140.165 134.439 132.486 131.097 130.370 129.977 129.489 126.061 123.189
83.271 77.211 77.000 76.789
35.043
22
13C NMR (151 MHz, CDCl3)
210.0 200.0 190.0 180.0 170.0 160.0 150.0 140.0 130.0 120.0 110.0 100.0 90.0 80.0 70.0 60.0 50.0 40.0 30.0 20.0 10.0 0
X : parts per Million : Carbon13

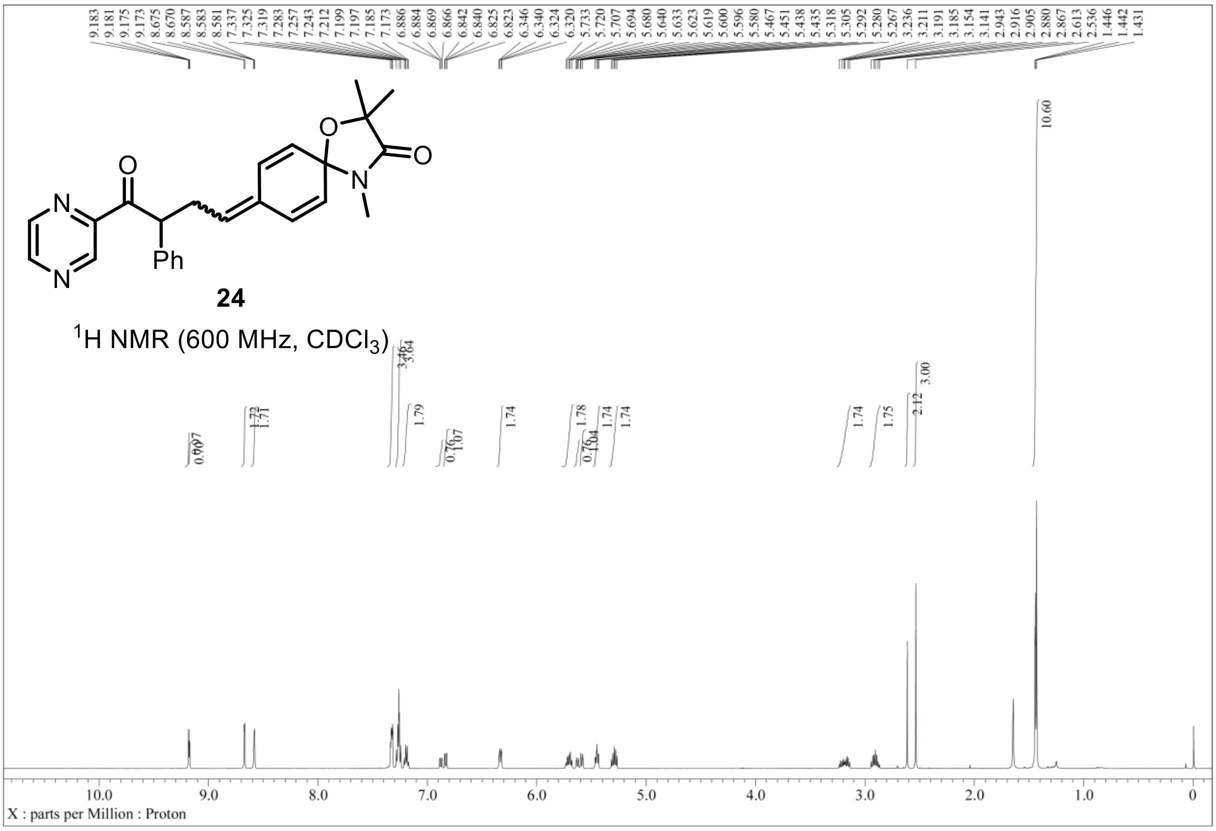

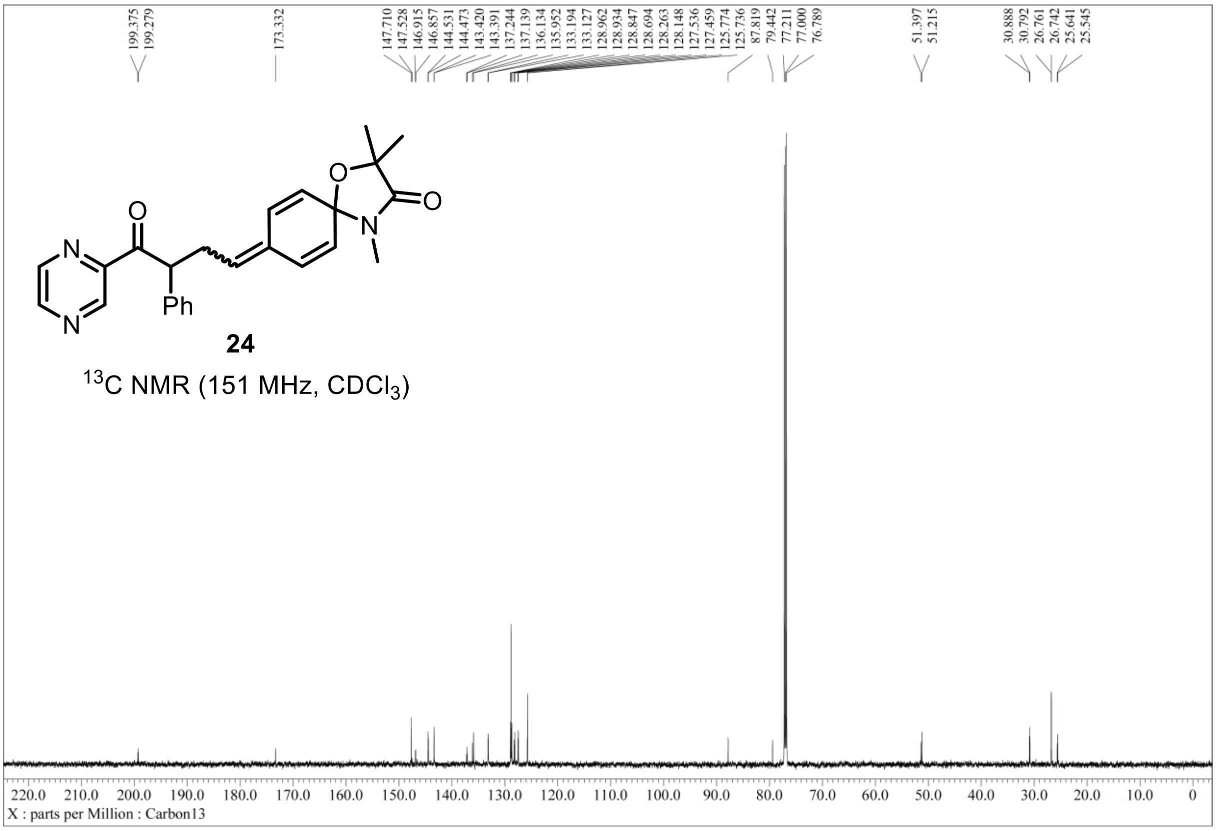

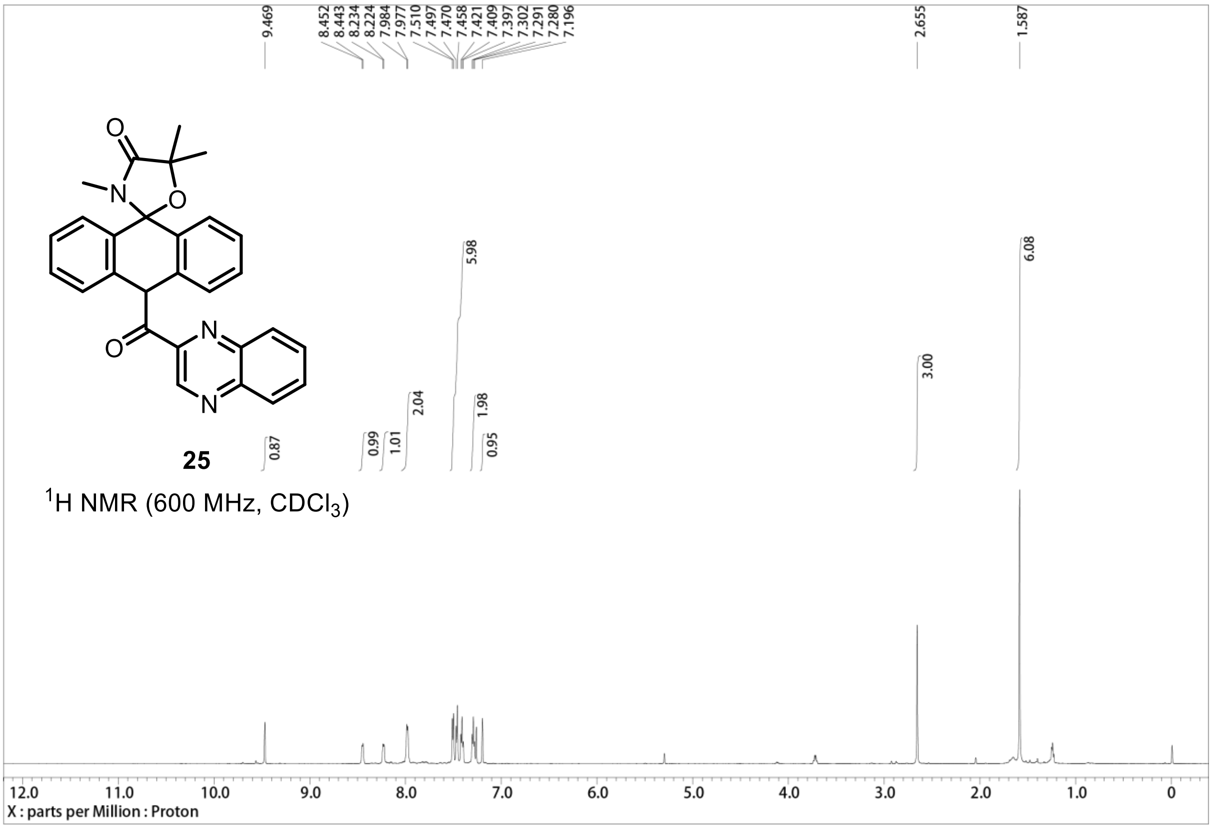

25

^{1}H NMR (600 MHz, $CDCl_3$)

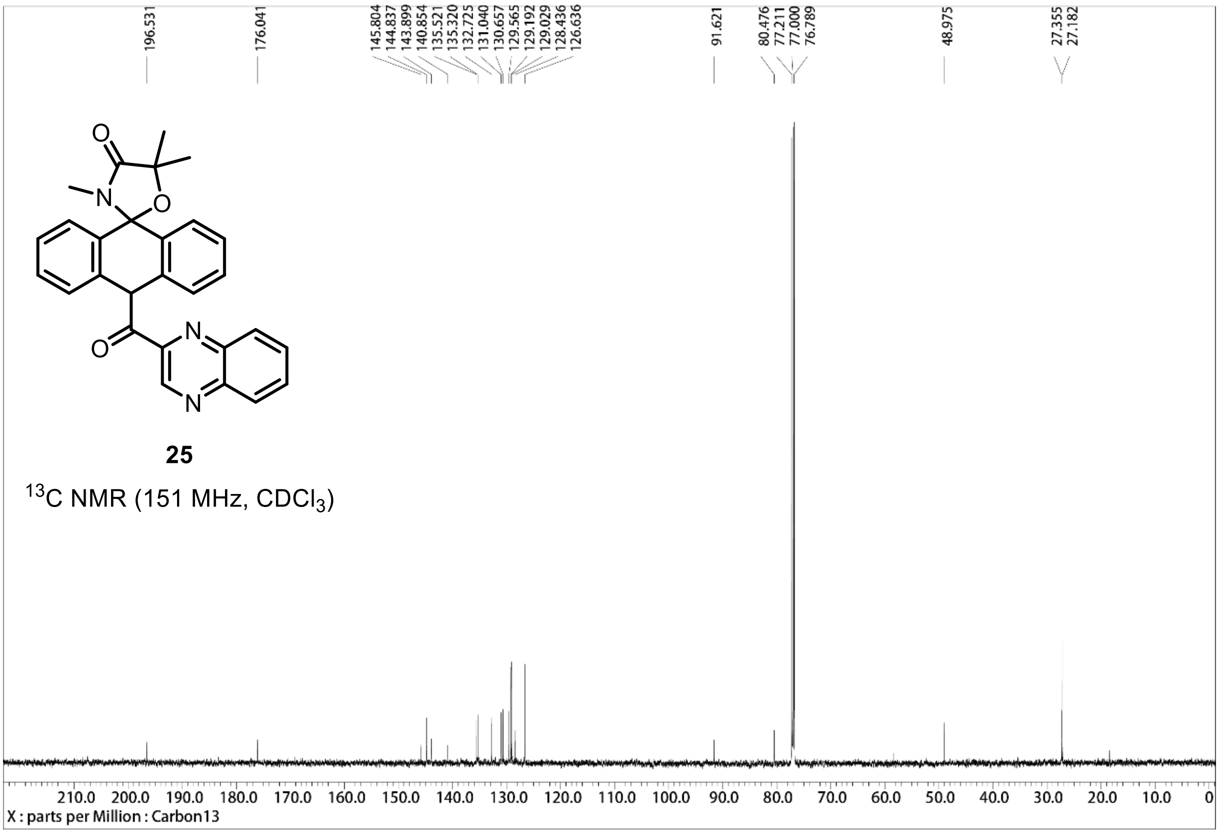

25

^{13}C NMR (151 MHz, $CDCl_3$)

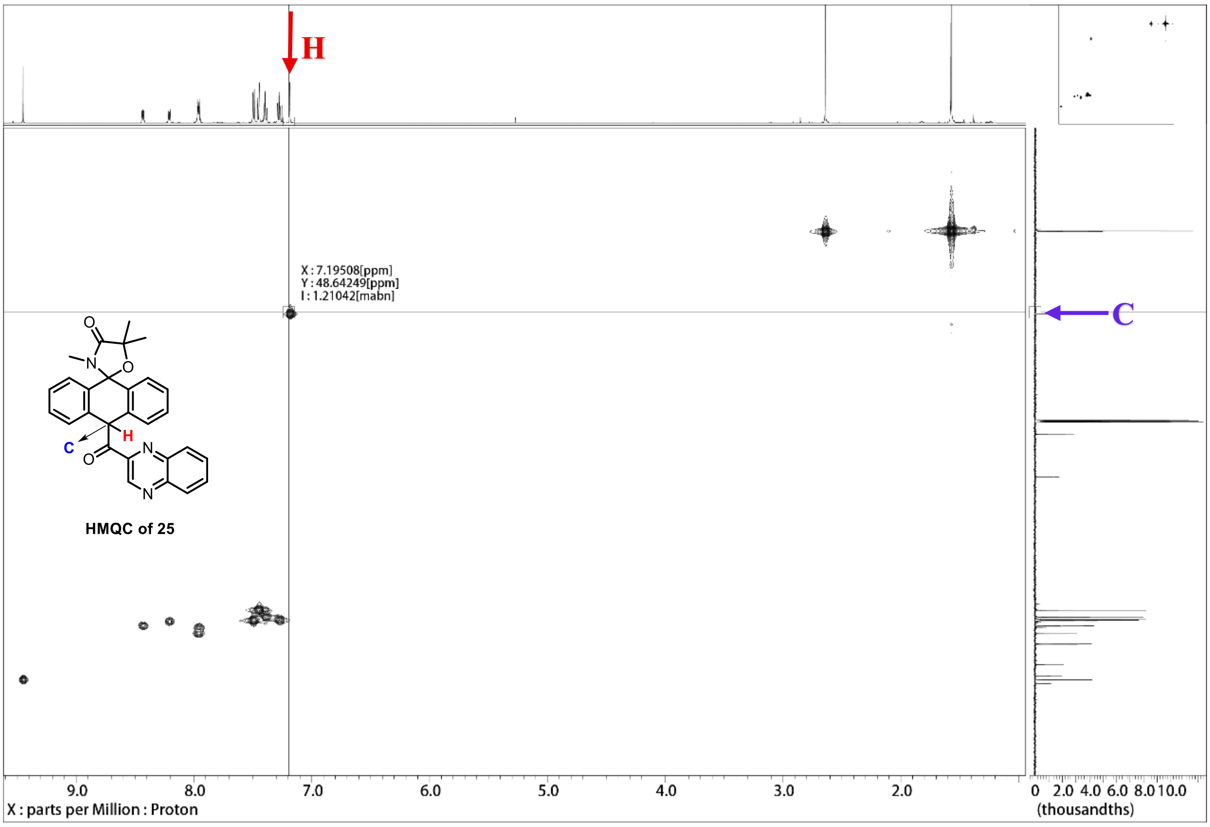
H
X : 7.19508[ppm]
Y : 48.64249[ppm]
I : 1.21042[mabn]
C
HMQC of 25
9.0
8.0
7.0
6.0
5.0
4.0
3.0
2.0
X : parts per Million : Proton
0 2.0 4.0 6.0 8.0 10.0
(thousandths)

12. References and Notes

1. Krell, E. Handbook of laboratory distillation. Elseriver Publishing Company, Amsterdam-London-New York (1963).
2. Rosengart, M. J. The technique of distillation and rectification in the laboratory. VEB Verlag Technik, Berlin (1954).
3. Stage, F. Die Kolonnen zur Laboratoriumsdestillation. Eine Übersicht über den Entwicklungsstand der Kolonnen zur Destillation im Laboratorium. *Angew. Chem.* **19**, 175−183 (1947).
4. Demchuk, D. V., Elinson, M. N. & Nikishin G. I. 'On water' Knoevenagel condensation of isatins with malononitrile. *Mendeleev Commun.* **21**, 224−225 (2011).
5. Delfau, L., Pecaut, J., Tomás-Mendivil, E. & Martin, D. Improved Protocols for the Synthesis of Precursors of Thiazol-2-ylidene N-Heterocyclic Carbenes. *Synlett* **35***,* 2097-2100 (2024).
6. Li, Q.-Z. *et al.* Oxidative Radical NHC Catalysis: Divergent Difunctionalization of Olefins through Intermolecular Hydrogen Atom Transfer. *Angew. Chem. Int. Ed.* **61**, e202207824 (2022).
7. Li, Q.-Z. *et al.* Remote site-selective arene C–H functionalization enabled by N-heterocyclic carbene organocatalysis. *Nat. Catal.* **7**, 900–911 (2024).
8. Li, Q.-Z. *et al.* Remote C(sp^3)−H Acylation of Amides and Cascade Cyclization via N-Heterocyclic Carbene Organocatalysis. *Angew. Chem. Int. Ed.* **61**, e202116629 (2022).
9. Pitzer, L., Schäfers, F. & Glorius, F. Rapid Assessment of the Reaction-Condition-Based Sensitivity of Chemical Transformations. *Angew. Chem. Int. Ed.* **58**, 8572−8576 (2019).
10. Wang, H., Bellotti, P., Zhang, X., Paulisch, T. O. & Glorius, F. A base-controlled switch of SO_2 reincorporation in photocatalyzed radical difunctionalization of alkenes. *Chem* **7**, 3412−3424 (2021).
11. Frisch, M. J. *et al.* Gaussian 16, Revision C.01, Gaussian, Inc., Wallingford CT. (2019).
12. Zhao,Y. & Truhlar, D. G. The M06 suite of density functionals for main group thermochemistry, thermochemical kinetics, noncovalent interactions, excited states, and transition elements: two new functionals and systematic testing of four M06-class functionals and 12 other functionals. *Chem. Acc.* **120**, 215−241 (2008).
13. Zhao,Y. & Truhlar, D. G. Density Functionals with Broad Applicability in Chemistry. *Acc. Chem. Res.* **41**, 157−167 (2008).
14. Krishnan, R., Binkley, J. S., Seeger, R. & Pople, J. A. Self-consistent molecular orbital methods. XX. A basis set for correlated wave functions. *J. Chem. Phys.* **72**, 650–654 (1980).
15. McLean, A. D. & Chandler, G. S. Contracted Gaussian basis sets for molecular calculations. I. Second row atoms, Z=11–18. *J. Chem. Phys.* **72**, 5639–564 (1980).

16. Cancès, E., Mennucci, B. & Tomasi, J. A new integral equation formalism for the polarizable continuum model: Theoretical background and applications to isotropic and anisotropic dielectrics. *J. Chem. Phys.* **107**, 3032−3041 (1997).
17. Mennucci, B., Cancès, E. & Tomasi, J. Evaluation of Solvent Effects in Isotropic and Anisotropic Dielectrics and in Ionic Solutions with a Unified Integral Equation Method: Theoretical Bases, Computational Implementation, and Numerical Applications. *J. Phys. Chem. B* **101**, 10506−10517 (1997).
18. Cancès, E. & Mennucci, B. New applications of integral equations methods for solvation continuum models: ionic solutions and liquid crystals. *J. Math. Chem.* **23**, 309−326 (1998).
19. Scalmani, G. & Frisch, M. J. Continuous surface charge polarizable continuum models of solvation. I. General formalism. *J. Chem. Phys.* **132**, 114110−114124 (2010).
20. Bauernschmitt, R. & Ahlrichs, R. Stability analysis for solutions of the closed shell Kohn–Sham equation. *J. Chem. Phys.* **104**, 9047−9052 (1996).
21. Fukui, K. The path of chemical reactions - the IRC approach. *Acc. Chem. Res.* **14**, 363−368 (1981).
22. Reed, A. E., Curtiss, L. A. & Weinhold, F. Intermolecular interactions from a natural bond orbital, donor-acceptor viewpoint. *Chem. Rev.* **88**, 899–926 (1988).
23. Reed, A. E., Weinstock, R. B. & Weinhold, F. Natural population analysis. *J. Phys. Chem.* **83**, 735–746 (1985).
24. Lu ,T. & Chen, F. Bond Order Analysis Based on the Laplacian of Electron Density in Fuzzy Overlap Space. *J. Phys. Chem. A* **117**, 3100−3108 (2013).
25. Lu ,T. & Chen, F. Multiwfn: A multifunctional wavefunction analyzer. *J. Comput. Chem.* **33**, 580−592 (2012).
26. Legault, C. Y. *CYLview*, 1.0b, Universite de Sherbrooke: Quebec, Canada, http://www.cylview.org. (2009).
