# Supplementary material for: Arene difunctionalization through an acyl-inserting Smiles rearrangement enabled by N-heterocyclic carbene catalysis
Source: Nat Commun. 2026 Jun 15;17:7539. doi: 10.1038/s41467-026-74546-3 (PMC13408099; doi:10.1038/s41467-026-74546-3)
Supplement: Supplementary file 2 — Transparent Peer Review file [file 41467_2026_74546_MOESM2_ESM.pdf]

nature portfolio

Peer Review File

Arene difunctionalization through an acyl-inserting Smiles rearrangement enabled by N-heterocyclic carbene catalysis

Corresponding Author: Professor Jun-Long Li

This file contains all reviewer reports in order by version, followed by all author rebuttals in order by version.

Version 0:

Reviewer comments:

Reviewer #1

(Remarks to the Author)
Li and co-workers report an N-heterocyclic carbene (NHC)–catalyzed radical protocol for the ipso/para-selective difunctionalization of arenes, which they describe as a new generation of “acyl-inserting Smiles rearrangement.” The reaction proceeds through the interception of radical Meisenheimer intermediates by NHC-bound ketyl radicals, followed by rearomatization to give 1,4-difunctionalized arenes. The authors highlight broad substrate scope, ring-expansion to benzo[b]azepines, and mechanistic insights from DFT calculations.

However, the conceptual novelty of this study appears limited. In their previous work, the authors had already demonstrated that intramolecular radical addition at the ipso-position generates a radical Meisenheimer intermediate, which then undergoes a similar coupling with a ketyl radical under NHC catalysis (ref 60, Nat. Catal. 7, 900–911 (2024)). In the present manuscript, the use of a Smiles-type rearrangement merely replaces the intramolecular aryl-migration step as the mode of Meisenheimer intermediate formation, rather than introducing a genuinely new reactivity concept. Furthermore, the mechanistic analysis by DFT calculations shows somewhat arbitrary or selectively interpreted tendencies, raising questions about the robustness of the proposed reaction pathway.

Taking these points together, I find that the manuscript, while technically sound and clearly written, does not meet the novelty and conceptual impact expected for publication in Nature Communications.

Major Comments
1. In Figure 1A, the authors frame their work in the context of “mono- to difunctionalization of arenes.” However, the key achievement in the present study is the realization of para-selective acylation using an appropriately tethered directing group. Therefore, the conceptual framing should instead emphasize the development of a para-acylation strategy rather than a generic arene difunctionalization. A quantitative or schematic comparison with existing para-acylation methodologies (e.g., directed C–H acylations, photochemical or electrochemical approaches) would greatly clarify the real advancement over previous art.
2. In the authors’ earlier report, the mechanistic model involved the formation of a σ-bond between a carbon radical at originally carbene position and the radical Meisenheimer intermediate, followed by a 1,2-migration leading to product formation—rather than a direct radical–radical coupling with a ketyl radical. The current DFT study, however, invokes a different pathway involving a direct coupling between a radical Meisenheimer intermediate and an NHC-bound ketyl radical. The authors should explicitly rationalize why the mechanistic paradigm has changed, and provide computational or experimental evidence that rules out the previously proposed 1,2-migration route.
3. In the computational model, the authors introduce a free carbonate anion to promote deprotonation and to assist the formation of TS4. However, the actual experimental conditions involve K_2CO_3 or Cs_2CO_3 as solid bases under optimized conditions. It remains unclear whether a free carbonate anion is realistically available in the reaction medium to act in this manner. The authors should justify this assumption, perhaps by evaluating an alternative model involving an explicit ion pair or solvent-assisted pathway.

Minor Comments
1. Typographical issue in Figure 1C. “reture to MI” should be corrected to “return to MI.”
2. Layout of Figure 2. The radar chart depicting the sensitivity analysis overlaps with its surrounding text box. Please adjust

the layout to avoid overlap.
3. Overall, most figures appear too small; the text labels are hardly legible at print scale. Please enlarge the graphics or increase font sizes.
4. References 56–59 are cited as the authors' previous work; however, none of these references are authored by the present group. Please correct this misattribution.
5. The term "Low O_2" is ambiguous. In the Supporting Information, it is described as "degassed for three times," but it is unclear whether this refers to a fully inert atmosphere (e.g., under N_2 or Ar) or simply partial degassing. Please clarify the experimental meaning of "Low O_2" and its reproducibility.

Reviewer #2

(Remarks to the Author)
Li et al. have developed an arene difunctionalization method that simultaneously installs two functional groups via an NHC-supported acyl-inserting Smiles rearrangement. They performed systematic optimization of reaction conditions including choice of NHC pre-catalyst and solvent. The substrate scope, including arene and aldehyde, is broad, and late-stage functionalization studies validate the synthetic applicability of the methodology. Their mechanistic investigations combining experiment and DFT calculations are in general thorough. Overall, the work is in good shape for publication after the following suggestions and comments are addressed.

1. Figure 2, the percentage values in radar graph are not sufficiently legible. Please edit the figure so the numbers are clearly visible. Also, define explicitly the ranges for "high T", "low T", and the other performance criteria used in the plot.

2. The authors state that DFT calculations were carried out using (U)M06-2X with GD3 dispersion correction. However, M06-2X is a meta-hybrid functional that was parameterized to partially account for medium-range correlation and dispersion-like interactions. In many previous benchmark studies, M06-2X yields good results even without an explicit dispersion correction. Thus, adding D3 may risk double counting or overcorrection in certain regimes. The authors should justify why they chose (U)M06-2X with GD3 dispersion correction. It's recommended to cite earlier DFT studies on similar systems, and include a benchmark comparing results with different functionals to show that the choice is sensible.

3. Since many radical and zwitterionic species are involved along the reaction coordinate, the authors should clearly specify the spin densities on each atom. A Natural Population Analysis or other charge decomposition should also be included to confirm that the computed geometries/transition states are consistent with the structures depicted in the manuscript.

4. In the mechanistic discussion, the authors propose:
"This delocalization stabilizes TS4 via hyperconjugation, thereby favoring the C–O cleavage pathway. In contrast, TS4′ exhibits pronounced ring distortion, which likely perturbs hyperconjugative interactions and thereby renders this pathway less favorable."
To support this claim, an orbital interaction diagram should be included to show the hyperconjugation.

Reviewer #3

(Remarks to the Author)
The manuscript by Li et al. reports an elegant N-heterocyclic carbene (NHC)-catalyzed radical acyl-inserting Smiles rearrangement that enables ipso/para-selective arene difunctionalization. The study is of high quality, featuring a broad substrate scope, well-designed mechanistic experiments, and convincing DFT analysis. The reaction concept is innovative and should attract significant attention in the field of NHC organocatalysis.
Overall, the manuscript is well-written and the conclusions are supported by experimental data. However, several minor points should be addressed before publication:
Comments:
1. There are several minor typographical errors (e.g., "unprecededted" on page 13 should read "unprecedented"; "difunctionaliztion" → "difunctionalization"). Please proofread carefully.

2. The proposed spiro dearomatized intermediate shown in the TOC is quite intriguing. Would it be possible to isolate (or intentionally design) such a spiro dearomatization intermediate experimentally? Moreover, in the case of a pyridine substrate, would the reaction be trapped in the spiro dearomatized state? Although this might represent another research direction, it would be very interesting if such conditions could lead to piperidine-type derivatives.

3. Did the authors systematically examine other possible 4-, 6-, or 7-exo radical cyclization pathways under similar conditions? A brief comment on this aspect would help clarify the reaction's selectivity and scope.

4. Are the NHC pre-catalysts used in this study commercially available? If they are not, please provide appropriate references for their preparation.

5. The methodology is indeed powerful, especially the synthetic applications demonstrated in Figures 4A and 5B. If possible, it would be helpful to include representative failed substrates or reaction limitations in the Supporting Information to give readers a more comprehensive understanding of the reaction scope.

Reviewer #4

(Remarks to the Author)
In this manuscript, Li and co-workers describes an NHC-catalyzed ipso/para-difunctionalization of arenes via a radical pathway. The transformation begins with a phenol ether bearing a redox-active N–O bond, which undergoes SET with the electron-rich Breslow intermediate (formed from the NHC catalyst and an aldehyde) to generate a nitrogen-centered radical, followed by a Smiles rearrangement. The resulting radical variant of the Meisenheimer intermediate is then intercepted by a persistent ketyl radical, ultimately undergoing elimination and rearomatization to deliver the desired difunctionalized product. The reaction design builds on the authors' previous work on radical para-acylation (ref. 60), where only para-functionalization was achieved. In this study, by replacing the alkyl substituent with an oxygen atom, they cleverly convert the original substituent into a leaving group, enabling an O-to-N swap. The substrate scope is broad, and notably, cyclic ether substrates afford ring-expansion products, giving benzoazepine analogues, which I find quite impressive. Overall, the work is carefully done and well suited for publication in Nat. Commun.

Minor issues

1. In Figure 1A, the two panels on the left convey essentially the same information. I recommend combining them.

2. In the discussion of Figure 3, the authors note that "An aldehyde featuring a sulfur-containing alkyl chain exhibited exceptionally high reactivity." Could the authors provide a possible explanation for this exceptional reactivity?

3. Have the authors explored radical relay coupling? Introducing styrene could potentially enable a three-component condensation.

4. In Figure 4, product 5l, dr should be provided.

5. For substrate 1, is substitution essential? The authors only presented gem-dimethyl and monomethyl analogues. Are unsubstituted derivatives also tolerated?

6. Page 6, line 122, "bis-arene substrate 3at" should be "biaryl product 3at".

7. Can the authors comment more on the N-leaving group?

Version 1:

Reviewer comments:

Reviewer #1

(Remarks to the Author)
The authors have clearly invested significant effort into the revision, and the new computational data provides a more detailed view of the reaction profile. Despite these improvements, the manuscript still suffers from a fundamental lack of internal consistency. The transition in the mechanistic paradigm from the authors' own Nature Catalysis 2024 report is particularly problematic because it lacks a rigorous comparative foundation. This ambiguity prevents a definitive endorsement of the proposed mechanism. Major revision is necessary to resolve these critical issues.

Major comment
While the focus on para-selective acylation is now better emphasized, the conceptual leap from the 2024 report remains tenuous. The previous study established a platform based on intramolecular radical addition at the ipso-position. The current work replaces this with a Smiles-type rearrangement to generate the Meisenheimer intermediate. Although this change is synthetically useful, it represents an incremental modification of the initiation step rather than a fundamental shift in reactivity. The authors must clarify whether this work introduces a new class of NHC catalysis or if it is simply a strategic extension of their established radical Meisenheimer framework. A comparative scheme illustrating the divergent pathways of the two systems would clarify this distinction.

The most significant weakness in the revised manuscript is the unexplained departure from the previous mechanistic model. In their earlier work, the authors proposed a 1,2-migration from a σ-bonded intermediate as the key product-forming step. The current manuscript, however, proposes direct radical-radical coupling between the Meisenheimer intermediate and an NHC-bound ketyl radical. The provided DFT data does not justify this shift in interpretation. It remains unclear why the 1,2-migration pathway, previously considered dominant, is now discounted. A direct computational comparison between the 1,2-migration and radical-radical coupling pathways must be performed under identical methodological settings. Without a clear explanation for this divergence, such as substrate-specific electronic effects or reaction conditions, the mechanistic narrative appears internally inconsistent with the authors' own precedents.

Minor comment
The authors have made a helpful effort to incorporate the role of the carbonate base. However, the current model assumes a freely solvated carbonate anion in an organic medium, which is a significant idealization given the heterogeneous nature of

K2CO3 or Cs2CO3. While a full re-evaluation using contact ion pair models may be beyond the scope of this study, the manuscript would benefit from a more transparent discussion regarding this simplification. Considering that the computed energy differences between competing pathways are relatively modest, it is important to acknowledge how the modeling of the base might influence the calculated barriers, particularly for the deprotonation step at TS4.

Reviewer #2

(Remarks to the Author)
The authors have addressed my comments satisfactorily. The radar plot presentation has been improved and the criteria for the sensitivity analysis are now clearly defined.
The computational section has been strengthened substantially. The authors now provide results without D3, incorporate counterions to better reflect experimental conditions, and demonstrate that the energetic trends are consistent across these methodological choices. In addition, the inclusion of charge/spin-density analyses improves confidence in the assignments of radical and zwitterionic intermediates and transition states.
The mechanistic discussion around TS4 vs TS4' has been appropriately revised to reflect what is supported by the updated model, replacing the prior hyperconjugation-centered argument with a clearer, more defensible rationale based on bond metrics and ring distortion.
Overall, I believe the manuscript is now in good shape and I recommend to be accepted.

Reviewer #3

(Remarks to the Author)
I am satisfied with current revised version. The manuscript can be accepted for publication.

Reviewer #4

(Remarks to the Author)
The authors have made meticulous efforts to address essentially all reviewers' comments. I have no additional revision requests. The revision is good to go.

Version 2:

Reviewer comments:

Reviewer #1

(Remarks to the Author)
The authors have invested immense effort and demonstrated remarkable sincerity in thoroughly addressing the concerns raised in the previous round of review. The additional computational data and comprehensive explanations provided in the rebuttal have successfully resolved my initial queries regarding the internal consistency of the mechanistic interpretations. I have no further requests for revision and recommend that the manuscript be accepted for publication in its current form.

Open Access This Peer Review File is licensed under a Creative Commons Attribution 4.0 International License, which permits use, sharing, adaptation, distribution and reproduction in any medium or format, as long as you give appropriate credit to the original author(s) and the source, provide a link to the Creative Commons license, and indicate if changes were made.
In cases where reviewers are anonymous, credit should be given to 'Anonymous Referee' and the source.
The images or other third party material in this Peer Review File are included in the article's Creative Commons license, unless indicated otherwise in a credit line to the material. If material is not included in the article's Creative Commons license and your intended use is not permitted by statutory regulation or exceeds the permitted use, you will need to obtain permission directly from the copyright holder.
To view a copy of this license, visit https://creativecommons.org/licenses/by/4.0/

Reviewer #1:

1. Comment: Li and co-workers report an N-heterocyclic carbene (NHC)–catalyzed radical protocol for the ipso/para-selective difunctionalization of arenes, which they describe as a new generation of "acyl-inserting Smiles rearrangement." The reaction proceeds through the interception of radical Meisenheimer intermediates by NHC-bound ketyl radicals, followed by rearomatization to give 1,4-difunctionalized arenes. The authors highlight broad substrate scope, ring-expansion to benzo[*b*]azepines, and mechanistic insights from DFT calculations.

Response: We greatly appreciate the valuable and objective comments provided by the reviewer. We have thoroughly examined each point and made the necessary revisions. We hope that the updated manuscript will be deemed appropriate for *Nat. Commun*.

2. Comment: However, the conceptual novelty of this study appears limited. In their previous work, the authors had already demonstrated that intramolecular radical addition at the ipso-position generates a radical Meisenheimer intermediate, which then undergoes a similar coupling with a ketyl radical under NHC catalysis (ref 60, Nat. Catal. 7, 900–911 (2024)). In the present manuscript, the use of a Smiles-type rearrangement merely replaces the intramolecular aryl-migration step as the mode of Meisenheimer intermediate formation, rather than introducing a genuinely new reactivity concept.

Response: We sincerely thank the reviewer for the thoughtful and critical assessment of our manuscript. We would like to take this opportunity to clarify the conceptual advancements involved in this work, particularly in relation to our previous study (*Nat. Catal.*, 7, 900–911 (2024)). While we acknowledge the reviewer's insight that the current study shares certain mechanistic similarities with our previous report—the unique cross-coupling of a radical Meisenheimer intermediate (MI) with the NHC-bound ketyl radical—we respectfully maintain that this work introduces a clear and meaningful departure from previous paradigms, and represents a substantial advancement both in mechanistic terms and synthetic applications. The key advancements in this work are outlined below:

1. Upgrading mechanistic paradigm of Smiles rearrangement chemistry. We know that the classical Smiles rearrangement proceeds through an intramolecular dearomative addition to generate a radical or anionic MI, followed by a simple aryl-migrative rearomatization. While advances have demonstrated the interception of MI to furnish the dearomatized cyclohexadiene products (for selected recent examples, see manuscript ref. 57–59), these strategies inherently terminate the Smiles sequence, precluding rearomatization and access to functionalized aromatic systems. To be different from the above-mentioned established chemistry, ***this work has successfully achieved both arene functionalization and migration, thereby upgrading the traditional Smiles rearrangement reactions.***

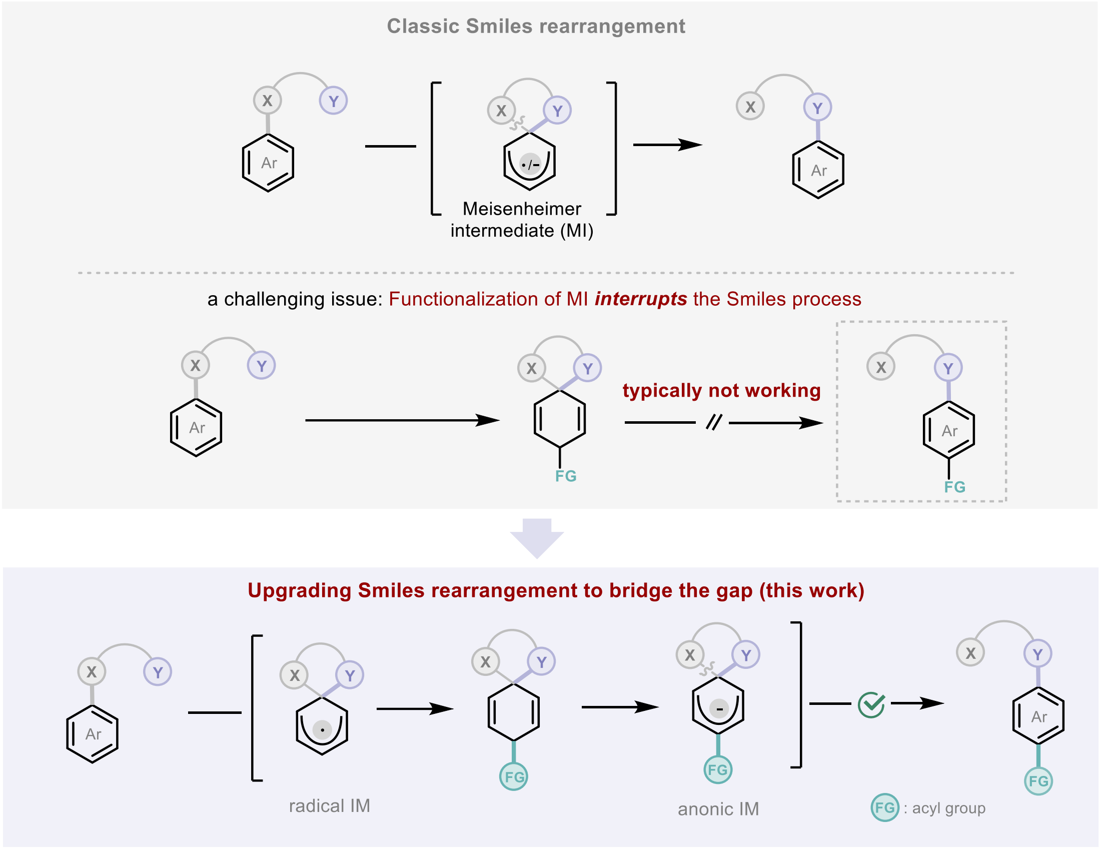

As shown in the figure above (see *the scheme with the lilac background*), our NHC catalytic system offers a simple and effective solution to bridge the gap: the initial radical MI is intercepted by an NHC-bound ketyl radical, and the resulting acyl group promotes deprotonation to form an anionic MI, which then undergoes rearomatization. This novel "radical MI to anionic MI" transformation not only facilitates rearomatization, completing the full Smiles rearrangement, but also installs two new functional groups at the aromatic *ipso* and *para* positions in a single step. In comparison to our previous work, this method represents more than just a replacement of steps, but a substantial mechanistic advancement that unlocks reactivity previously inaccessible in classical Smiles rearrangement chemistry.

2. **A brand new synthetic application scenarios: organocatalytic *ipso*/*para*-difunctionalization of diverse arenes & ring expansion to facilitate skeletal diversification.** Compared with our earlier work (ref. 60), the present method unlocks reactivity that was previously inaccessible, including concurrent functionalization of the arene *ipso* C–O and *para* C–H bonds, as well as an unprecedented six- to seven-membered ring expansion.

While our prior remote acylation strategy constituted a valuable method for arene functionalization, it exhibited several notable limitations: (i) it was limited to five-membered MIs; (ii) the side chain employed as a directing group was not removable; and (iii) functionalization was confined to the *para* position. In contrast, the current strategy successfully addresses these constraints, thereby substantially broadening the synthetic

potential and structural diversity accessible through an alternative Smiles-type chemistry. The synthetic significances and advancements in this work were shown as follows:

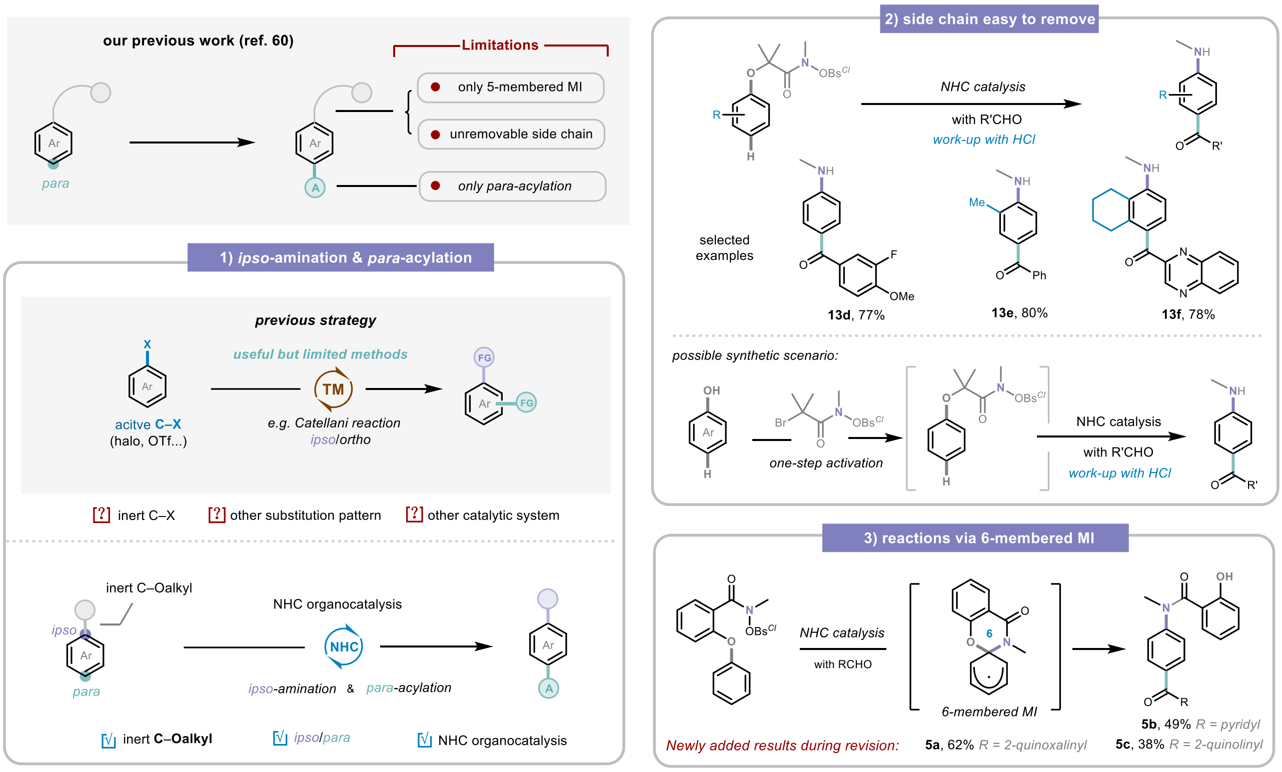

1) Direct arene difunctionalization offers a promising strategy for installing two functional groups in one step; however, existing methods remain limited, with most approaches restricted to *ipso*/*ortho*-selective pathways that rely on a reactive Csp^2–X bond, particularly in transition-metal catalyzed Catellani-type reactions. In contrast, our manuscript presents a fundamentally distinct approach—an NHC organocatalytic strategy that enables unprecedented *ipso*-amination of inert aromatic C–O and *para*-acylation of C–H bonds, thereby expanding the scope and diversity of accessible substitution patterns in arene functionalization.

2) The current method features a removable side chain, achieved by incorporating an oxygen atom into the side chain. It allows for traceless cleavage via a simple acidic workup. In principle, the side chain can also be installed efficiently in a single step, allowing for the rapid conversion of a simple phenol into 4-acyl-substituted aniline (for detailed discussion, see revised SI, Section 3).

3) Expanding beyond the previously reported pathway that predominantly relied on five-membered Meisenheimer intermediates, our system now also accommodates six-membered MIs. Acting on Reviewer 3's insightful suggestion, we successfully implemented this extension using 2-phenoxybenzamide derivatives, which undergo the target transformation via a six-membered intermediate. These new findings notably improve the synthetic generality of the method over previous approaches.

Importantly, the *ipso* heteroatom translocation enables challenging ring-expansion reactions on bicyclic

substrates—transformations that were previously out of reach with our prior methodology. For instance, as illustrated in the following scheme, the benzopyran-derived substrates (six-membered rings) could be efficiently converted into benzo[*b*]azepine analogues (seven-membered rings), further showcasing the distinctive power of our organocatalytic platform.

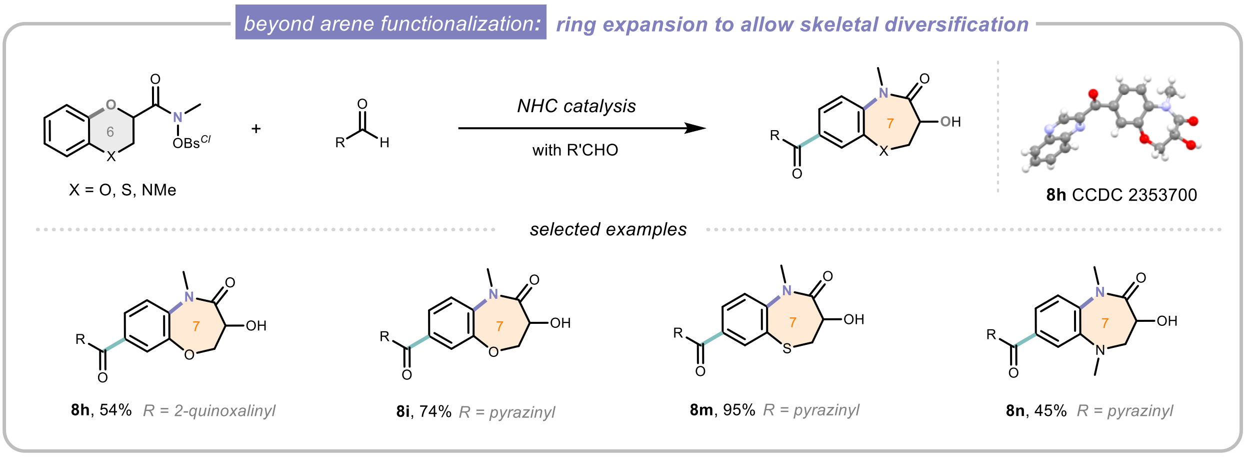

Once again, we would like to express our gratitude for the opportunity to clarify these points. We sincerely hope that these explanations help convey the novelty and significance of our revised work.

3. Comment: Furthermore, the mechanistic analysis by DFT calculations shows somewhat arbitrary or selectively interpreted tendencies, raising questions about the robustness of the proposed reaction pathway.

Response: Thank you very much for the helpful and constructive comments. We sincerely apologize for the shortcomings in our previous DFT studies. In response to your detailed "Major Comments 2 & 3" regarding the DFT calculations, as well as the related concerns raised by Reviewer 2, we conducted a thorough re-investigation and have substantially revised our computational studies. The key revisions include: 1) providing a clear explanation for why the 1,2-migration pathway was not initially discussed in the main text and revising the manuscript to acknowledge the possibility of this route; 2) incorporating K^+ counterions in our calculations to maintain electronic neutrality for all intermediates and transition states; 3) reoptimizing all reaction pathways without the D3 dispersion correction; 4) systematically re-examining and correcting the assignment of charges and radicals with the assistance of Mulliken and Natural Population Analysis (NPA), *etc*. (for detail, see the following responses to "Major Comments 2 & 3" and other related responses)

We believe these substantial revisions have significantly strengthened the DFT calculations and improved the robustness of the proposed reaction pathway. Accordingly, the updated computational details and corresponding discussions are now included in the revised version of both manuscript and supplementary information. Once again, we deeply appreciate your careful reading and valuable suggestions, which have significantly strengthened our work.

4. Comment: Taking these points together, I find that the manuscript, while technically sound and clearly written, does not meet the novelty and conceptual impact expected for publication in Nature Communications.

Response: We are grateful for the reviewer's valuable and critical feedback, particularly the positive remark that our manuscript is both technically sound and clearly written. After carefully reflecting on all of your comments, we realize that our previous submission may not have sufficiently emphasized the novelty and conceptual significance of our research. Therefore, we would like to present an updated statement that more accurately and comprehensively articulates these important aspects of our work (please also see the response to Comment 2), which are listed as follows:

1. **A novel organocatalysis strategy has been developed for *ipso/para*-selective functionalization of arenes**. Arene difunctionalization provides powerful strategy to install two functional groups in a single step. However, existing methods for direct arene difunctionalizations remain scarce and are confined to *ipso*/*ortho* selectivity (e.g. transition metal-catalyzed Catellani reaction). In this work, we present an unique NHC organocatalytic strategy that realize difunctionalization of the *ipso* C–O and *para* C–H bonds of arenes.

2. **An "*Acyl Inerting Smiles Rearrangement*" is disclosed to supplement the classical Smiles rearrangement chemistry.** The classic Smiles rearrangement proceeds *via* an intramolecular dearomative addition to form radical or anionic Meisenheimer intermediates, followed by a simple aryl-migrative rearomatization. In contrast, our organocatalytic transformation unveils an unprecedented "*Acyl-Inserting Smiles Rearrangement*". Here, the radical MI is intercepted by an NHC-bound radical, and the incorporated acyl group subsequently promotes deprotonation to generate an anionic MI, which undergoes rearomatization. This mechanistic innovation expands the canonical paradigm of rearrangement chemistry and enables previously inaccessible modes of arene functionalization.

3. **Novel synthetic application has been unlocked, and detailed mechanistic investigations elucidate the reaction pathway.** This method exhibits excellent functional-group tolerance and provides access to a wide range of aniline products (91 examples, up to 98% yield). Beyond arene functionalization, novel synthetic application scenario is showcased by ring-expansion reaction to benzo[*b*]azepine derivatives and its synthetic generality is further highlighted by the late-stage diversification of pharmaceutically relevant molecules. Experimental mechanistic studies and DFT calculations of the energy profile corroborate both the NHC radical catalytic cycle and the acyl-inserting Smiles rearrangement pathway.

We have made a point-by-point responds in the above and following response to address your concerns and also made the necessary revisions. We hope our responses and revisions will meet with your approval.

5. Comment: Major Comments

1. In Figure 1A, the authors frame their work in the context of “mono- to difunctionalization of arenes.” However, the key achievement in the present study is the realization of para-selective acylation using an appropriately tethered directing group. Therefore, the conceptual framing should instead emphasize the development of a para-acylation strategy rather than a generic arene difunctionalization. A quantitative or schematic comparison with existing para-acylation methodologies (e.g., directed C–H acylations, photochemical or electrochemical approaches) would greatly clarify the real advancement over previous art.

Response: Thanks a lot for the reviewer’s valuable and constructive comments. We greatly appreciate the suggestion to highlight the *para*-acylation aspect, which is indeed an important and noteworthy feature of our methodology. As suggested, we have revised the corresponding text in the Introduction section to state more explicitly: “In contrast to conventional arene acylation strategies, this method achieves precise *para*-selective acylation of readily available aryl ether substrates...” We have also added representative references on conventional acylation methodologies, and these revisions have been highlighted in the revised version of manuscript.

In addition, the reviewer rightly points out that a key outcome of this work is the development of a *para*-acylation strategy and requests a comparison with existing acylation methods. We fully agree that achieving high site-selectivity is a significant contribution. In response, we have added a schematic comparison (see revised supplementary information, Section 4) that outlines several representative aromatic C–H acylation strategies, their typical site-selectivity, and key characteristics. As depicted in the figure below, classical electrophilic substitutions, such as Friedel–Crafts reactions, typically require harsh conditions (strong acids, elevated temperatures). Radical acylative additions, often initiated photochemically or electrochemically, are largely governed by the arene’s intrinsic electronic properties. Both approaches rely on the intrinsic electronic nature of the aromatic ring to determine site-selectivity. Directing-group-assisted transition-metal catalysis typically leads to *ortho*-selective acylation. In contrast, our approach, involving radical coupling between a dearomatized carbon-centered radical and an NHC-bound ketyl radical, achieves exclusive *para*-acylation while simultaneously swapping the heteroatom at the *ipso* position. This comparison, as suggested by the reviewer, more clearly emphasizes the significance of our work. We sincerely thank the reviewer for this helpful suggestion.

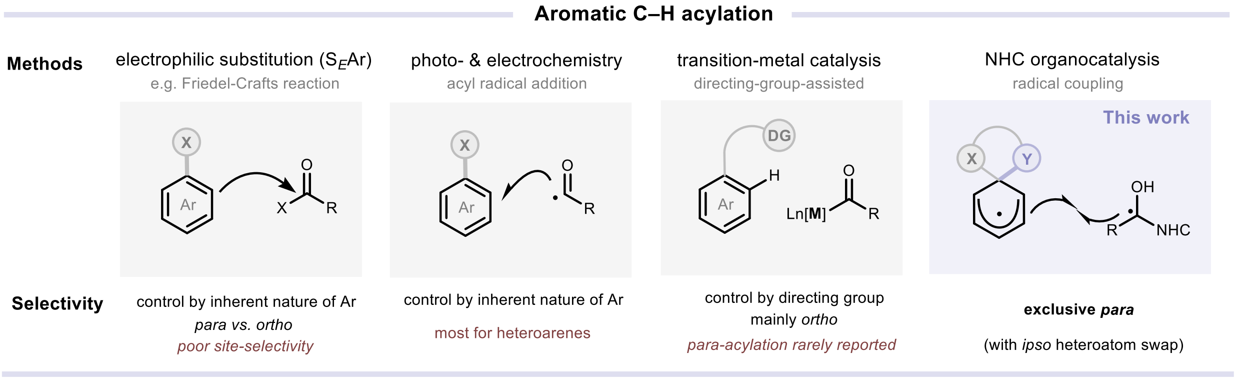

At the same time, we would also like to respectfully suggest that another key innovation of this work lies in its ability to accomplish two distinct and valuable transformations simultaneously within a single catalytic process. While the *para*-acylation is certainly crucial, the reaction also concurrently facilitates an *ipso* C–O to C–N conversion—effectively performing an O-to-N swap on the aromatic ring. As we know that converting aryl ethers, especially those with inert C(sp^2)–O alkyl bonds, into valuable aniline derivatives under mild conditions remains a significant challenge in synthetic chemistry. Notable precedents, such as heteroatom translocation via Smiles rearrangements (*e.g.*, *J. Am. Chem. Soc.* **146**, 6476 (2024); *Angew. Chem. Int. Ed.* **56**, 12533 (2017)), have demonstrated the potential for such transformations. This aspect was recognized positively by Reviewer 4, who commented: “In this study, by replacing the alkyl substituent with an oxygen atom, they cleverly convert the original substituent into a leaving group, enabling an O-to-N swap.” As such, the *ipso* O-to-N substitution should be considered another key advance of this work.

Consequently, we believe that the primary conceptual advance lies not just in a new acylation method, but in the creation of a novel catalytic platform that combines 1) challenging *ipso* heteroatom substitution with 2) *para*-selective C–H functionalization. This synergy results in 1,4-difunctionalization of arenes, directly yielding valuable 4-acylated aniline scaffolds. Moreover, our transformation is enabled under mild, transition-metal-free conditions using NHC organocatalysis. The ability of organocatalytic system to orchestrate the activation of inert C–O bonds, mediate radical generation, control regioselectivity over long distances, and facilitate rearomatization represents a notable advance in the field of organocatalysis. Therefore, in addition to the *para*-acylation, we also wish to highlight these related advances in our manuscript.

Finally, in response to the reviewer’s suggestions, we have removed the previous conceptual framing that centered on generic arene difunctionalization. Instead, in the revised Figure 1A in the manuscript, we present an overview of prior art on *ipso* and *para*-functionalization of arenes, respectively. Then, we underscore the challenge of achieving site-selective *ipso*/*para* difunctionalization under mild conditions. This updated schematic introduction allows us to more precisely and effectively demonstrate the advancements of

our work over previous methods, as advised by the reviewer.

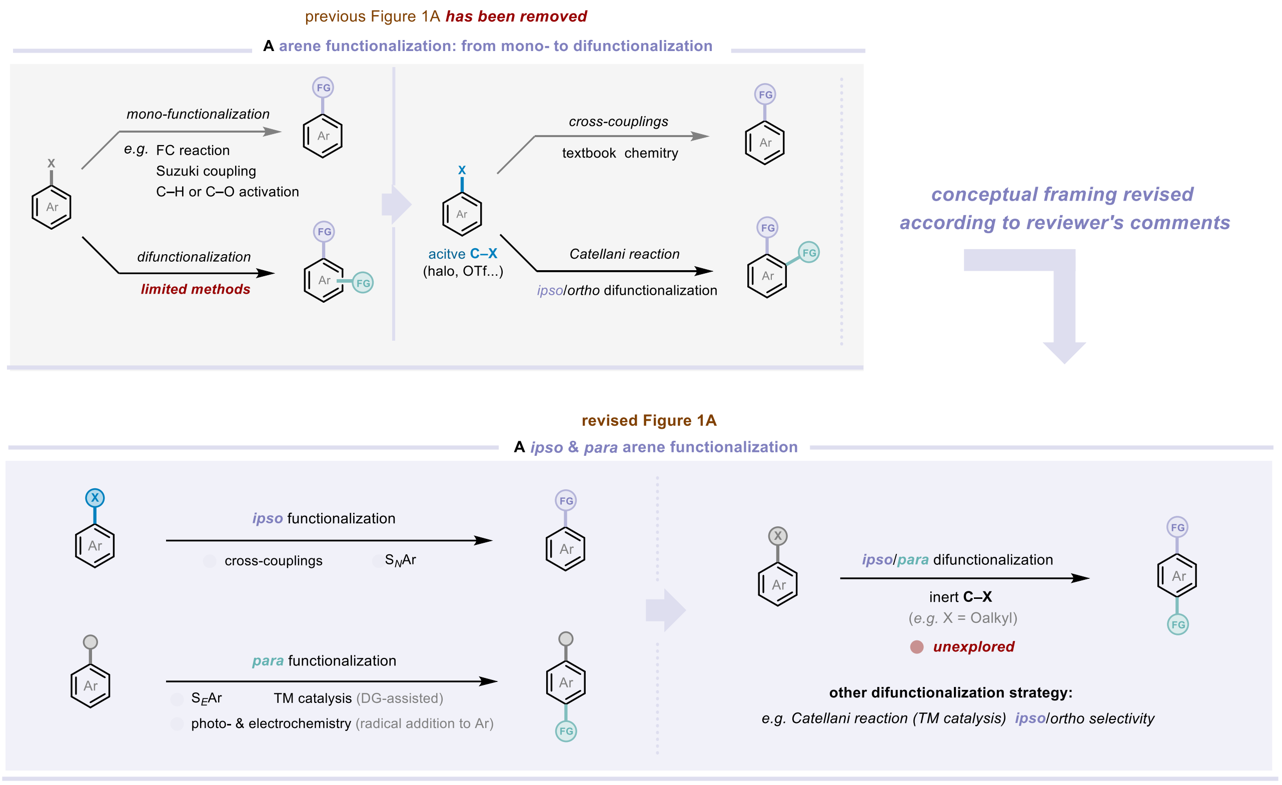

In summary, we have gratefully followed the reviewer's suggestion to more effectively emphasize the *para*-acylation achievement. We also hope that our detailed explanation highlights the conceptual novelty of this work comprehensively, which lies in the successful integration of two challenging transformations—heteroatom translocation and *para*-selective radical acylation—into a single organocatalytic system. We are truly thankful for the reviewer's insightful comments and for the opportunity to elaborate on these points.

6. Comment: 2. In the authors' earlier report, the mechanistic model involved the formation of a σ-bond between a carbon radical at originally carbene position and the radical Meisenheimer intermediate, followed by a 1,2-migration leading to product formation—rather than a direct radical–radical coupling with a ketyl radical. The current DFT study, however, invokes a different pathway involving a direct coupling between a radical Meisenheimer intermediate and an NHC-bound ketyl radical. The authors should explicitly rationalize why the mechanistic paradigm has changed, and provide computational or experimental evidence that rules out the previously proposed 1,2-migration route.

Response: Many thanks for the reviewer's valuable and insightful comments. In fact, both mechanistic pathways—the direct radical–radical coupling and the alternative route involving 1,2-carbon migration—were evaluated in our initial DFT studies, with the comparative results provided in Figure S1 of the previous version of the supplementary information. As shown in the scheme below, radical coupling at

the carbene carbon is calculated to be 1.3 kcal/mol higher in energy than coupling at the original acyl carbon. Notably, the 1,2-carbon migration must overcome a higher energy barrier of 15.6 kcal/mol to reach the transition state **s-TS2-p**, which exhibits a Gibbs free energy of –50.3 kcal/mol. Based on these results, we initially considered the 1,2-migration pathway less favorable and therefore did not include it in the previous main manuscript.

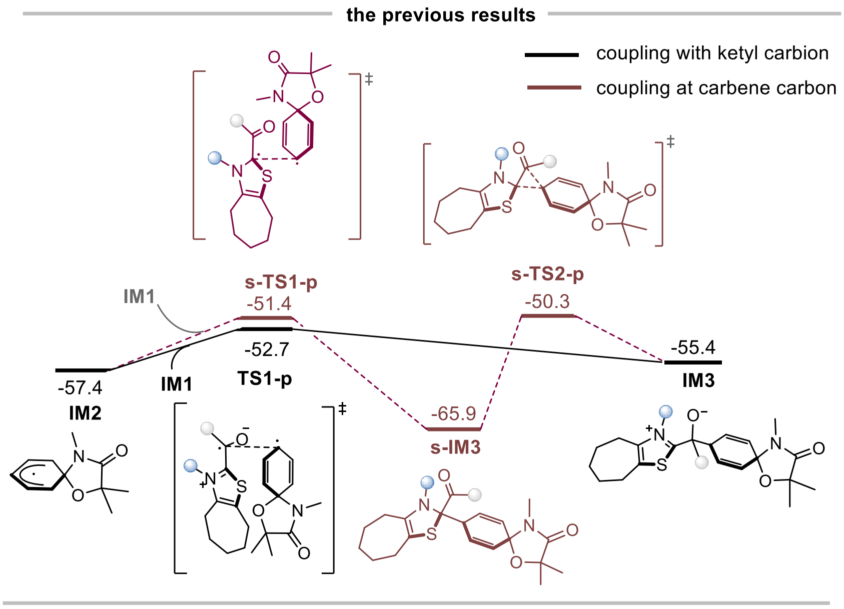

In addition, following the suggestion from this reviewer and Reviewer 2, we have re-evaluated the energies using the M06-2X functional without D3 dispersion correction. The updated calculations (summarized in the following figure) re-affirm our initial conclusion regarding the relative preference for direct coupling over the 1,2-migration pathway. Nevertheless, we acknowledge that the energy difference between the two pathways is modest and insufficient to conclusively exclude the 1,2-migration route. Accordingly, we have added the following statement to the revised manuscript: “In addition, another radical coupling pathway involving a 1,2-carbon migration process is also possible (see Figure S1 in Supplementary Information)”. These revisions have been updated and highlighted in the revised manuscript.

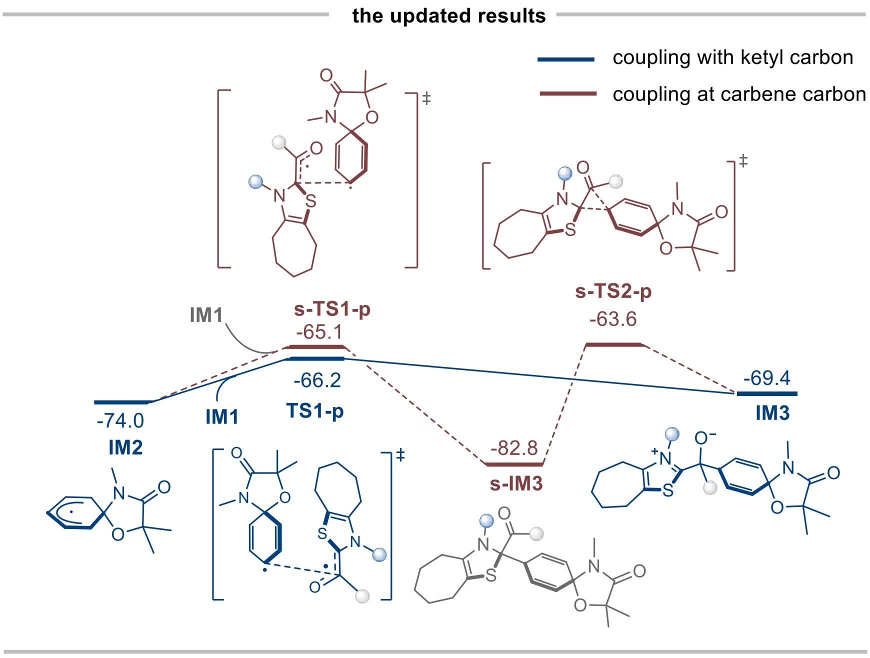

7. Comment: 3. In the computational model, the authors introduce a free carbonate anion to promote deprotonation and to assist the formation of TS4. However, the actual experimental conditions involve K_2CO_3 or Cs_2CO_3 as solid bases under optimized conditions. It remains unclear whether a free carbonate anion is realistically available in the reaction medium to act in this manner. The authors should justify this assumption, perhaps by evaluating an alternative model involving an explicit ion pair or solvent-assisted pathway.

Response: We greatly appreciate the reviewer's thoughtful comments. In our earlier DFT studies, we employed the carbonate anion to simplify the computational process. However, we did not fully consider that, under actual experimental conditions, the carbonate anion cannot exist independently. Following the reviewer's suggestion, we have used an ion pair involving the actual experimental base K_2CO_3 to update our DFT calculations. Accordingly, we have re-optimized all the relevant intermediates and transition states by adding K^+ counterions. Selected key structural changes are shown in the following figure.

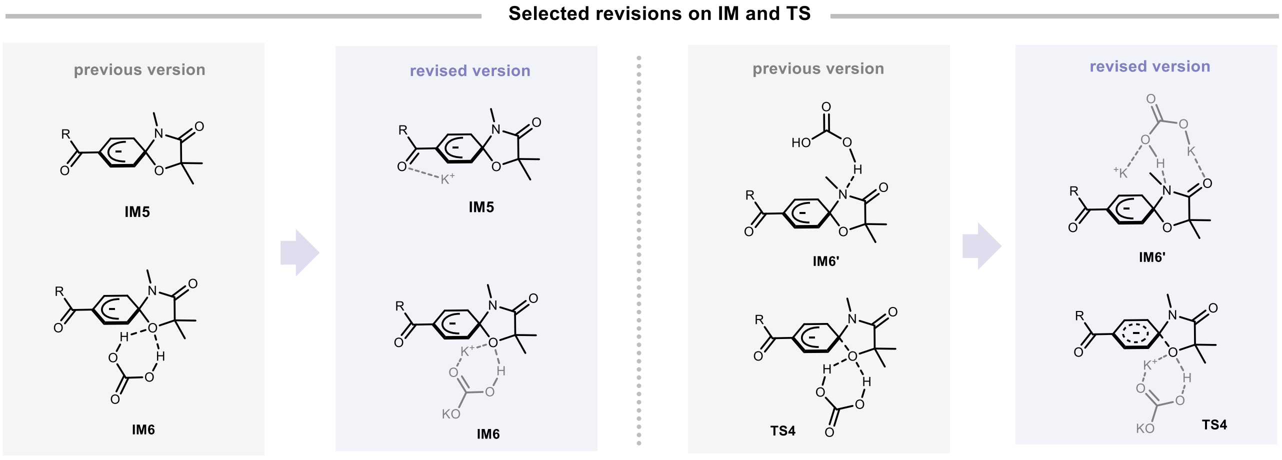

Notably, the updated energies of both the intermediates and transition states across the entire energy profile have been significantly lowered compared to the previous version. Nevertheless, the regio- and chemoselectivities in the updated computational results remain fully consistent with our prior conclusions.

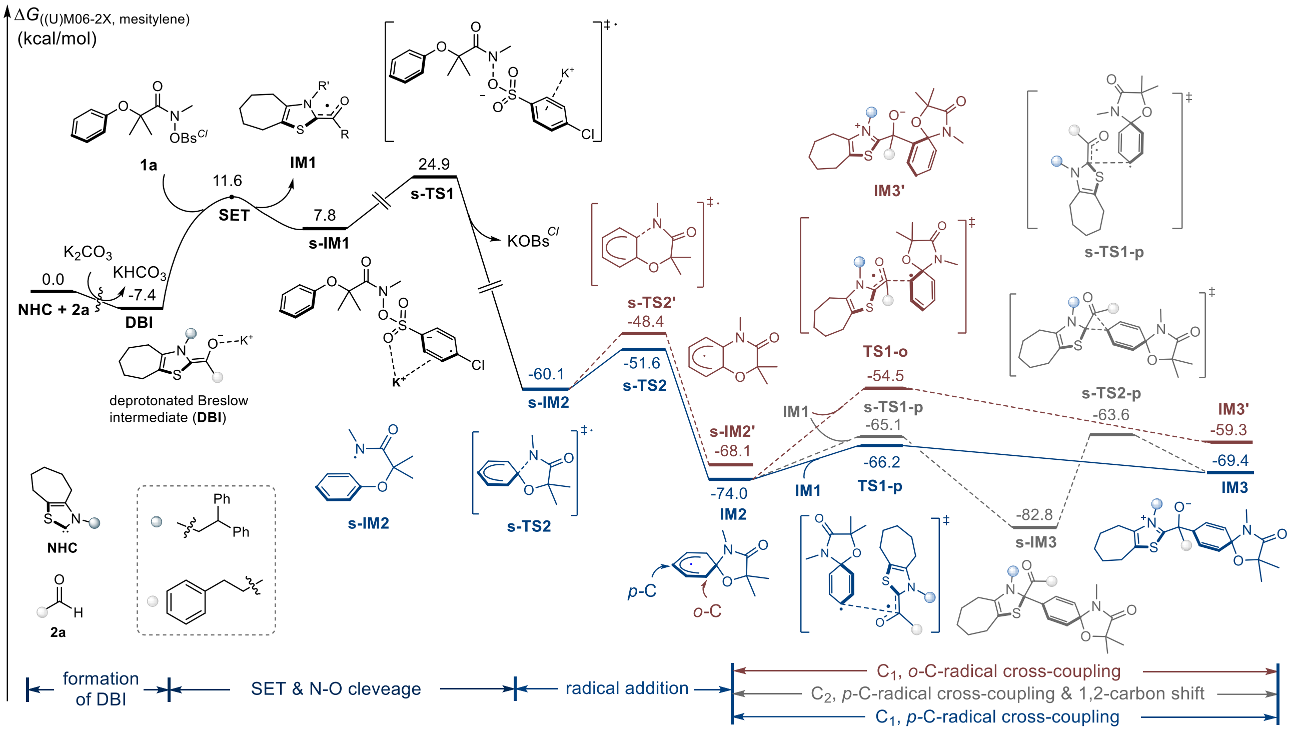

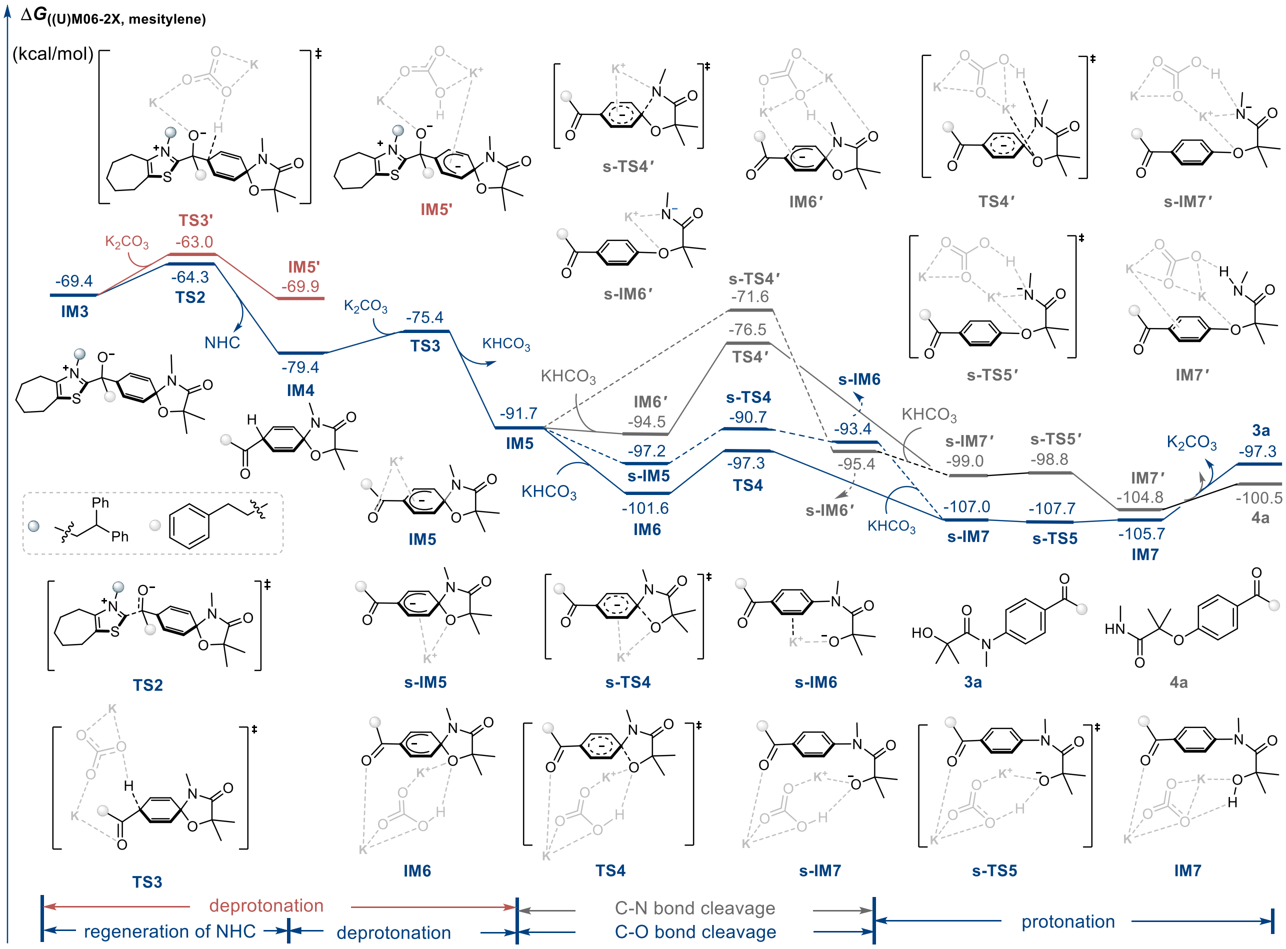

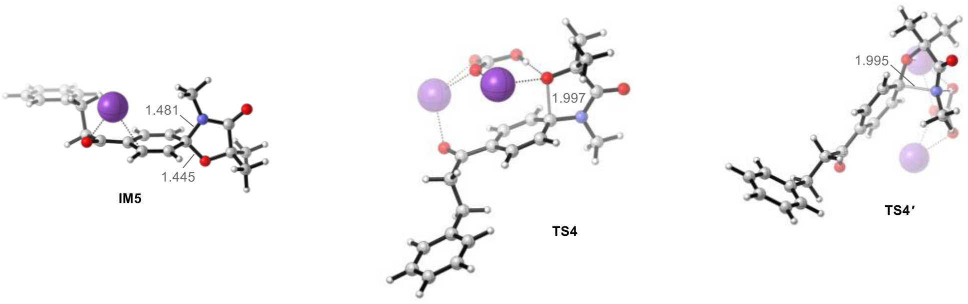

Based on these results, we have updated the entire computational study, including the main manuscript and the supplementary information. The whole pathway is outlined in the above figures, which has been updated in the supplementary information.

Accordingly, the revised energy profile in Figure 6B (energy profile for reaction pathway) has been updated in the main manuscript, as illustrated below.

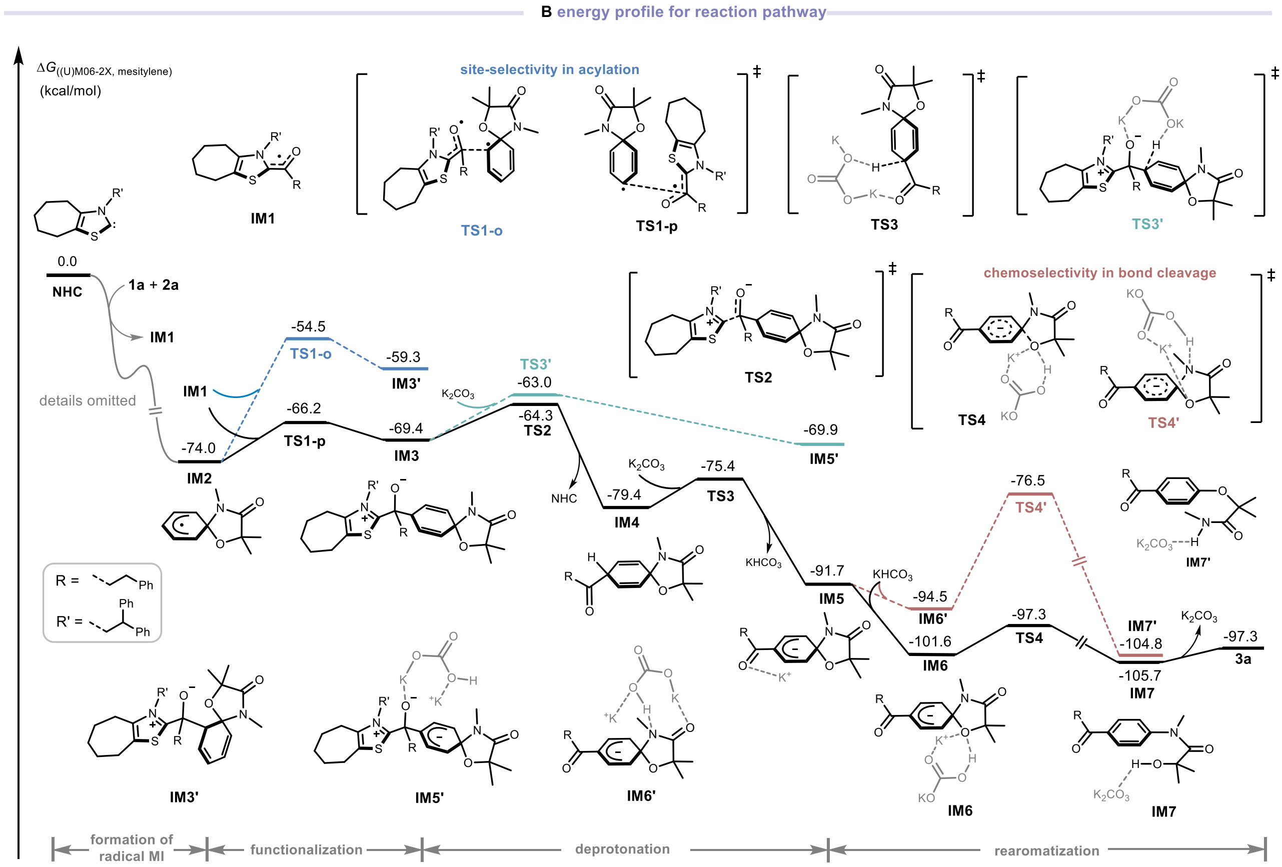

Again, we are grateful for the reviewer’s constructive feedback, which has significantly improved the DFT calculations and reinforced the robustness of the proposed reaction pathway.

8. Comment: Minor Comments

1. Typographical issue in Figure 1C. “reture to MI” should be corrected to “return to MI.”

Response: Many thanks for the reviewer’s valuable comments. The typographical issue “reture to MI” has been corrected to “return to MI”.

9. Comment: 2. Layout of Figure 2. The radar chart depicting the sensitivity analysis overlaps with its surrounding text box. Please adjust the layout to avoid overlap.

Response: Thanks a lot for the reviewer's helpful comments. According to the reviewer's suggestion, we have adjusted the radar chart to avoid overlap with the surrounding text box.

10. Comment: 3. Overall, most figures appear too small; the text labels are hardly legible at print scale. Please enlarge the graphics or increase font sizes.

Response: Thank you very much for the reviewer's helpful suggestions. According to the reviewer's suggestion, we have enlarged the font sizes of all text labels throughout the figures in the revised manuscript to ensure clear legibility.

11. Comment: 4. References 56–59 are cited as the authors' previous work; however, none of these references are authored by the present group. Please correct this misattribution.

Response: Many thanks for the reviewer's valuable comments. Actually, ref. 56–59 have been previously cited as selected reviews on radical NHC catalysis, while our previous work on remote acylation was cited as ref. 60. As noted by the reviewer, we fully agree with the reviewer's comment that the previous text "we disclosed a remote *para*-selective C–H functionalization of arenes by trapping the transient radical MI through *N*-heterocyclic carbene (NHC) catalysis[56–59]", incorrectly attributed the concept of radical NHC catalysis to our prior study. According to the reviewer's suggestion, we have reversed the order of citation, and the review articles is now cited as ref. 61–64 (the previous ref. 56–69). Accordingly, we have revised the citation to "we disclosed a remote *para*-selective C–H functionalization[60] of arenes by trapping the transient radical MI through *N*-heterocyclic carbene (NHC) catalysis[61–64]". These revisions have been highlighted in the revised manuscript. Again, thank you so much for this helpful suggestion.

12. Comment: 5. The term "Low O_2" is ambiguous. In the Supporting Information, it is described as "degassed for three times," but it is unclear whether this refers to a fully inert atmosphere (e.g., under N_2 or Ar) or simply partial degassing. Please clarify the experimental meaning of "Low O_2" and its reproducibility.

Response: Thank you very much for the reviewer's helpful comments. In response to the reviewer's suggestion, we have added a footnote to entry 4 of Table S7 (corresponding to the sample degassed three times), specifying "Under a fully argon atmosphere" to clarify that the experimental operation was conducted under a completely argon atmosphere.

Again, thank you very much for the constructive comments from this reviewer, and we hope our detailed responses and careful revisions will meet with your approval.

Reviewer #2:

1. Comment: Li et al. have developed an arene difunctionalization method that simultaneously installs two functional groups via an NHC-supported acyl-inserting Smiles rearrangement. They performed systematic optimization of reaction conditions including choice of NHC pre-catalyst and solvent. The substrate scope, including arene and aldehyde, is broad, and late-stage functionalization studies validate the synthetic applicability of the methodology. Their mechanistic investigations combining experiment and DFT calculations are in general thorough. Overall, the work is in good shape for publication after the following suggestions and comments are addressed.

Response: Thank you so much for the reviewer's positive comments and kind recommendations. We sincerely hope that our carefully revised work will be finally suitable for *Nat. Commun*.

2. Comment: 1. Figure 2, the percentage values in radar graph are not sufficiently legible. Please edit the figure so the numbers are clearly visible. Also, define explicitly the ranges for "high T", "low T", and the other performance criteria used in the plot.

Response: We deeply appreciate the reviewer's valuable comments. The radar graph for the sensitivity screening has been elegantly invented by Glorius and co-coworkers. In our initial studies, we have performed the assessment of reaction-condition-based sensitivity and presented the data according to the Glorius' method: *Angew. Chem. Int. Ed.* **58**, 8572–8576 (2019).

Following the reviewer's suggestion, we have revised the radar chart by enlarging the font size of the labels and adjusting the color of the values to ensure clear visibility. In addition, we have provided a detailed definition of the condition derivation on the right side of the radar chart. The details of revisions have been shown in the following figure.

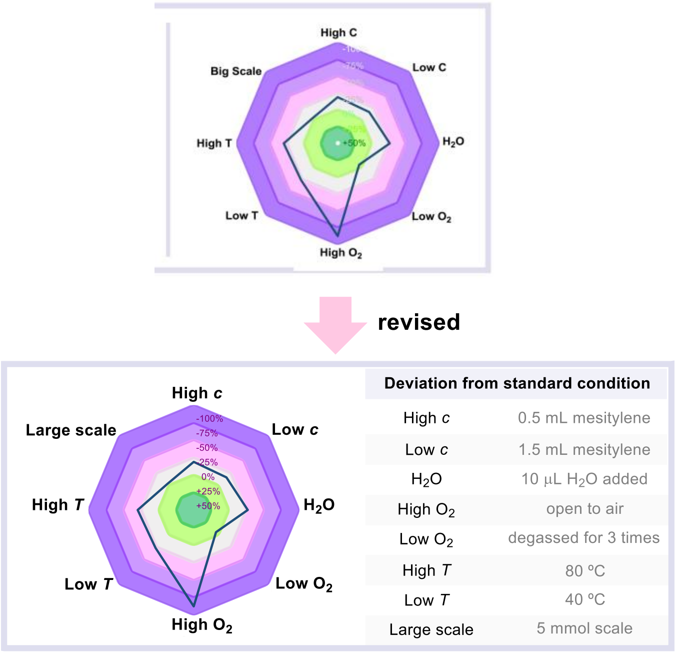

Deviation from standard condition	
High *c*	0.5 mL mesitylene
Low *c*	1.5 mL mesitylene
H_2O	10 μL H_2O added
High O_2	open to air
Low O_2	degassed for 3 times
High *T*	80 °C
Low *T*	40 °C
Large scale	5 mmol scale

3. Comment: 2. The authors state that DFT calculations were carried out using (U)M06-2X with GD3 dispersion correction. However, M06-2X is a meta-hybrid functional that was parameterized to partially account for medium-range correlation and dispersion-like interactions. In many previous benchmark studies, M06-2X yields good results even without an explicit dispersion correction. Thus, adding D3 may risk double counting or overcorrection in certain regimes. The authors should justify why they chose (U)M06-2X with GD3 dispersion correction. It's recommended to cite earlier DFT studies on similar systems, and include a benchmark comparing results with different functionals to show that the choice is sensible.

Response: Thanks a lot for your constructive comments on our calculations. In our previous DFT studies, we employed the (U)M06-2X functional with Grimme's D3 dispersion correction primarily because this method has been successfully applied to several organic reaction systems (e.g. *Sci. Adv.* **11**, eaeb0206 (2025); *Nat. Chem.* (2025), doi: 10.1038/s41557-025-01885-x; *Nat Commun.* **11**, 946 (2020)).

Inspired by the reviewer's insightful suggestions, and to further confirm the reliability of the M06-2X density functional theory method level, we also applied (U)M06-2X without GD3 dispersion correction to examine the energy profiles of our organic reaction pathway. The corresponding energy profile with this approach has been achieved and is now provided below.

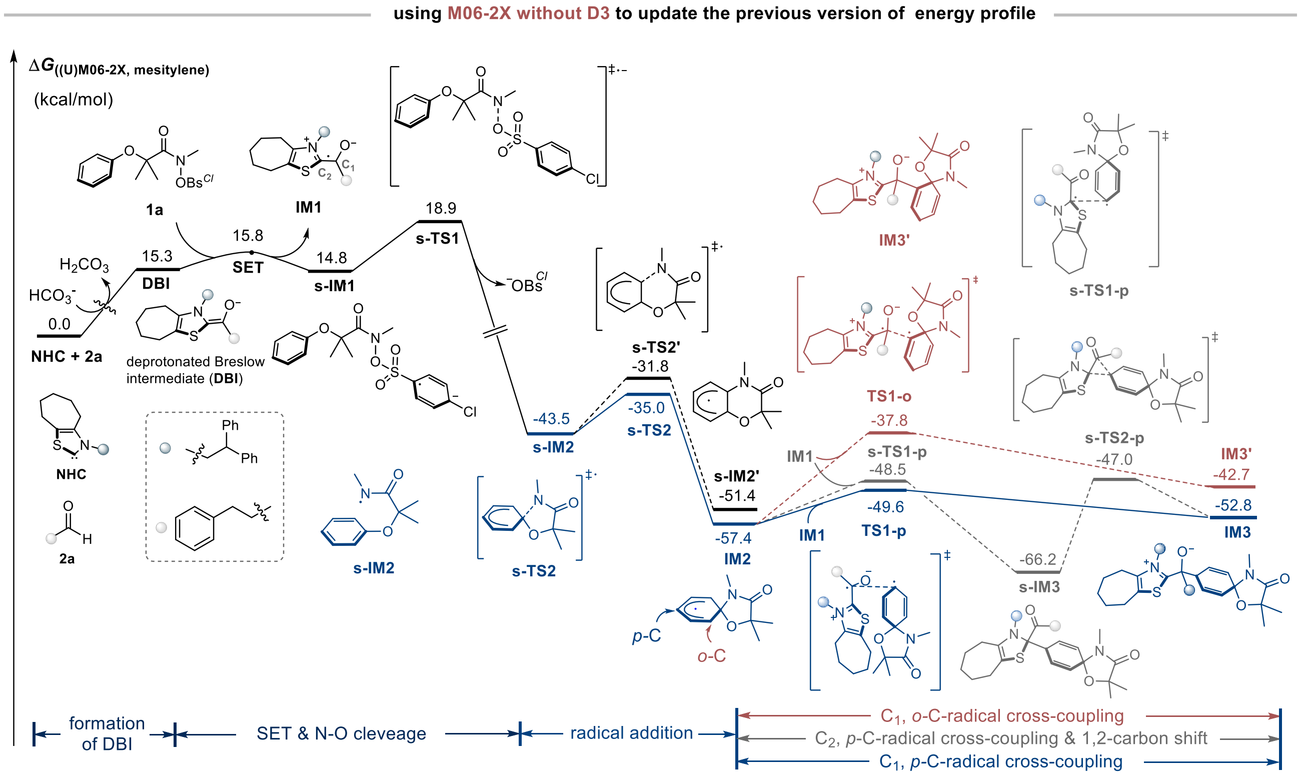

using M06-2X without D3 to update the previous version of energy profile

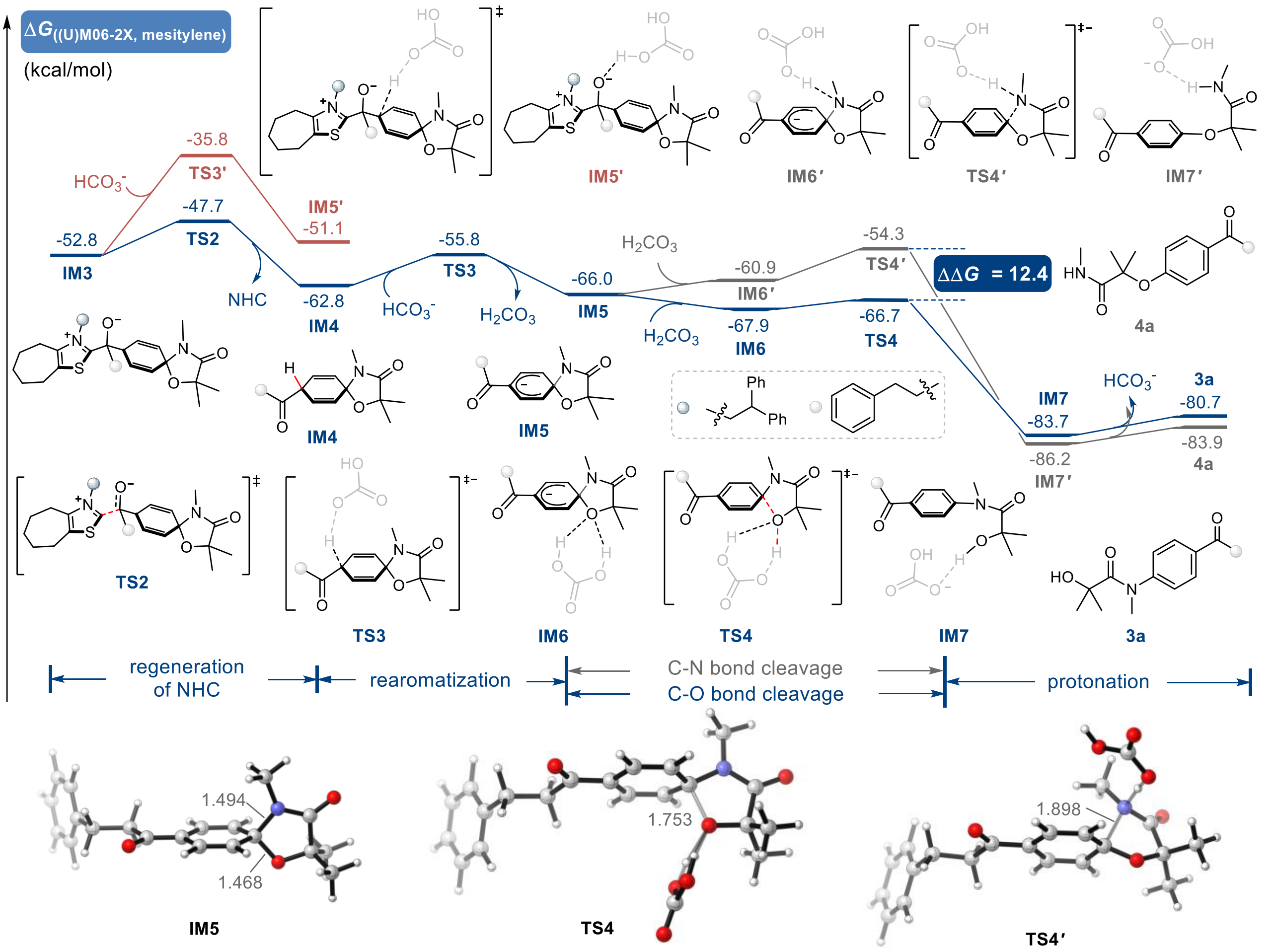

From the above results, we are pleased to observe that the energy trends obtained with (U)M06-2X (without GD3) closely resemble those from our earlier M06-2X-D3 calculations. For clearer comparison, we have combined the Gibbs free energy data from both methods into a single graph to illustrate their similarity (see the figure below), which clearly illustrates their consistent energetic tendencies.

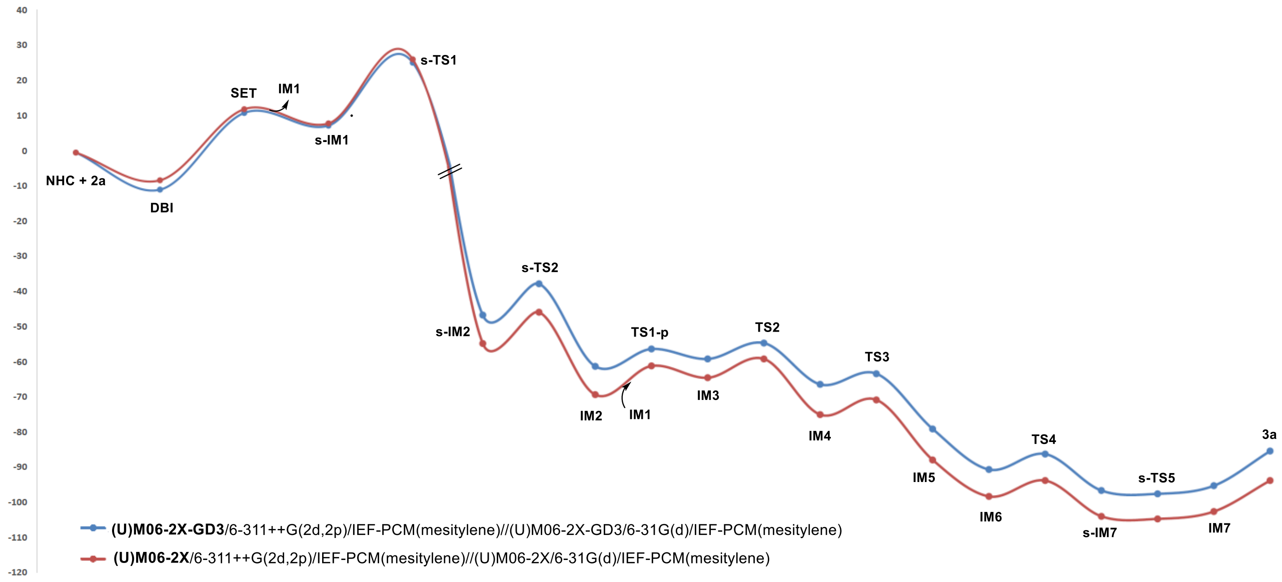

Nevertheless, we also fully agree with the reviewer that adding D3 may risk double counting or overcorrection in certain regimes. In light of this important consideration, we have revised the computational results throughout the main manuscript and the supplementary information to reflect the outcomes obtained by using *(U)M06-2X without D3 dispersion correction*. In addition, according to the reviewer 1's comments, we have now explicitly included K^+ counterions in related relevant intermediates and transition states to better model the actual reaction environment. These updated structures have been fully reoptimized by using *(U)M06-2X without D3 dispersion correction*, and the final revised version of Figure 6B, incorporating the K^+-containing species, has been revised in the manuscript, which is shown below.

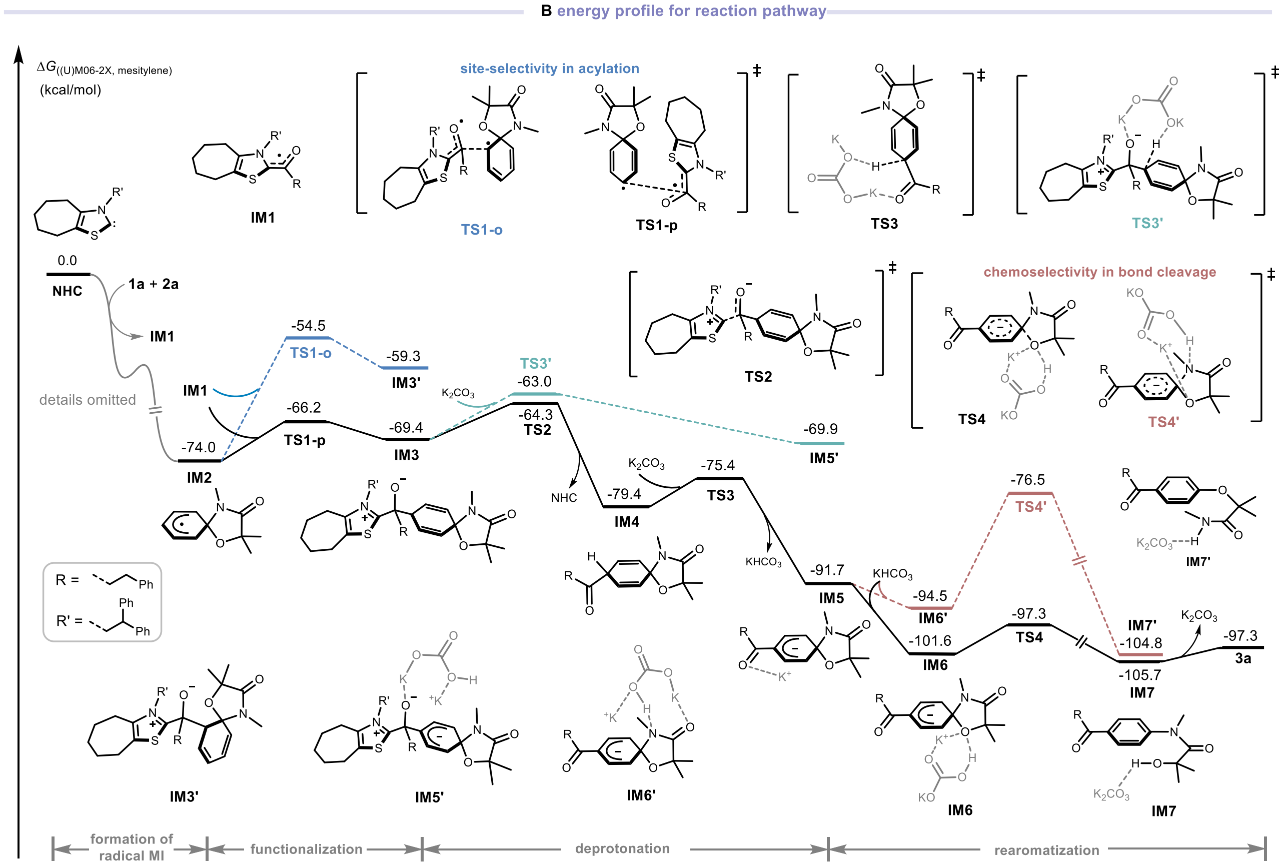

Collectively, these refinements significantly enhance the accuracy and reliability of our computational model, and we greatly appreciate the reviewer's valuable guidance in helping us strengthen the manuscript.

4. Comment: 3. Since many radical and zwitterionic species are involved along the reaction coordinate, the authors should clearly specify the spin densities on each atom. A Natural Population Analysis or other charge decomposition should also be included to confirm that the computed geometries/transition states are consistent with the structures depicted in the manuscript.

Response: Thank you so much for your constructive comments on our calculations. According to the reviewer's suggestion, we have carried out Mulliken or Natural Population Analysis to rigorously examine

the charge distribution and spin density of relative key intermediates and transition states in our proposed reaction pathway. Selected examples have been listed in the following figures. The detailed analysis provides valuable insight into the electronic structure of the key species and intermediates involved and help validate the reliability of our computed geometries. These updated computational results have been added in the revised supplementary information. Notably, representative results from these population analyses are summarized in the figures below, where selected intermediates and important transition states are annotated with their respective atomic charges and spin densities.

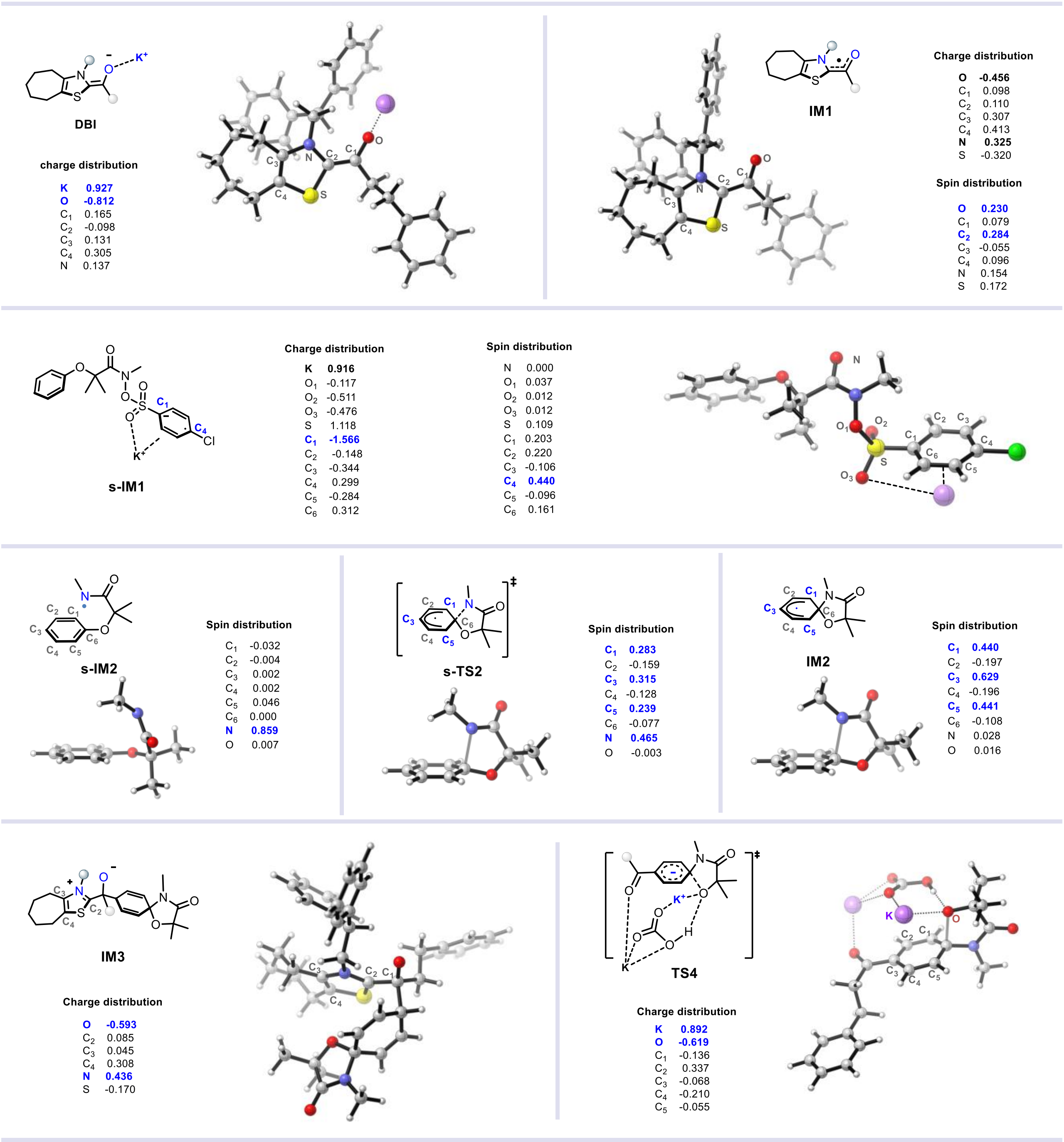

Based on this refined electronic characterization, we really identified minor inaccuracies in a few previously optimized structures—particularly in the cases that have ambiguous spin localization or charge assignment. Consequently, we have revised the corresponding intermediates and transition states in the manuscript and SI, accordingly. Some corrections are provided below for clarity.

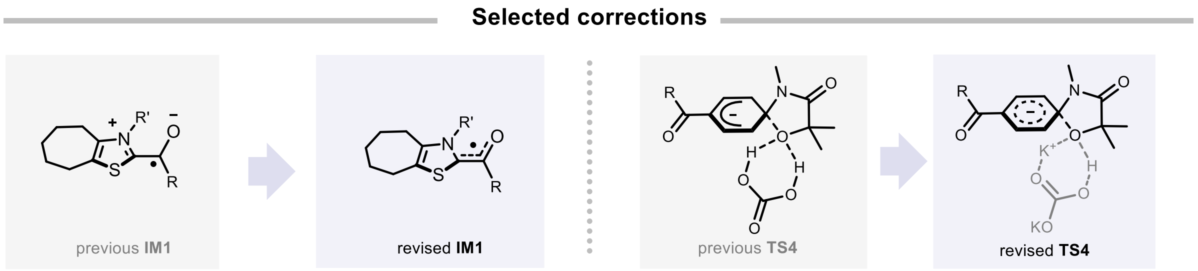

These improvements further strengthen the consistency and robustness of our mechanistic interpretation, and we appreciate the reviewer's valuable input in guiding this enhancement.

5. Comment: 4. In the mechanistic discussion, the authors propose:

"This delocalization stabilizes TS4 via hyperconjugation, thereby favoring the C–O cleavage pathway. In contrast, TS4′ exhibits pronounced ring distortion, which likely perturbs hyperconjugative interactions and thereby renders this pathway less favorable." To support this claim, an orbital interaction diagram should be included to show the hyperconjugation.

Response: Thanks a lot for the reviewer's insightful comments and helpful suggestions. Based on our previous calculation model, we performed a frontier molecular orbital (FMO) study on the **TS4** (without considering the counter cation) in the previous model, and we indeed observed apparent orbital overlap within the five-membered ring, which we interpreted as evidence of stabilizing hyperconjugative interactions favoring C–O bond cleavage. (see the following figure, *upper part*)

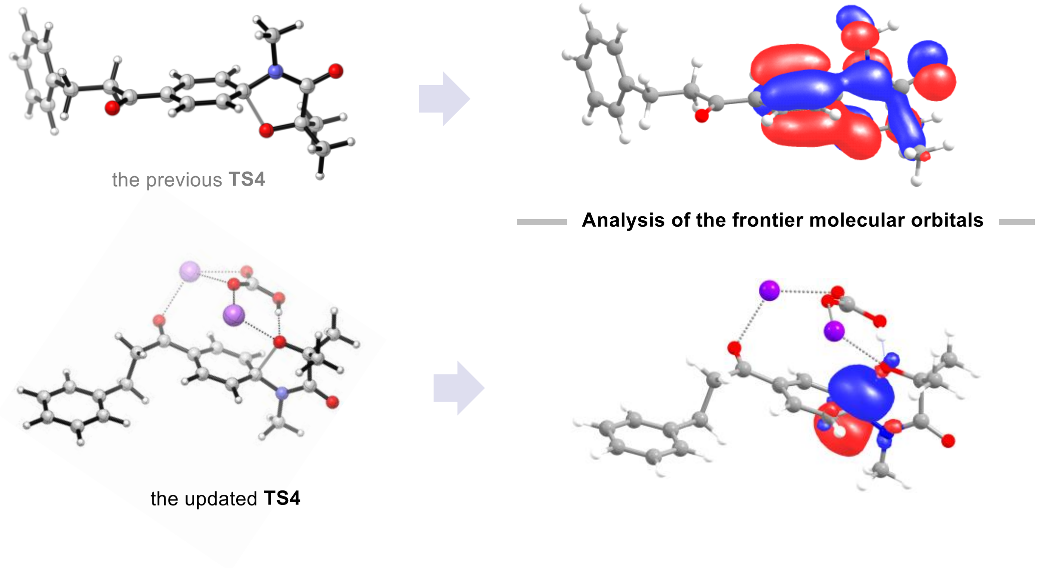

Following the suggestion from the other Reviewer 1#, we have already updated our calculation model, and used an alternative, more-accurate model involving an ion pair. Thus, we carefully reoptimized the geometry of the updated **TS4** that involve K^+ in the transition state. Upon re-examination of the FMOs for this updated structure, however, the expected hyperconjugative interactions are no longer clearly discernible. This result suggests that hyperconjugation may not be the dominant stabilizing factor in the revised transition state.

To further investigate the origin of the observed selectivity, we recalculated the Laplacian Bond Order (LBO) for the C–O and C–N bonds in the updated **TS4**. While the absolute values have slightly increased compared to our previous results (from 0.212 vs. 0.486 to 0.260 vs. 0.521), the relative trend remains consistent. These results suggest that the C–O bond is significantly weaker than the C–N bond, well supporting preferential C–O cleavage.

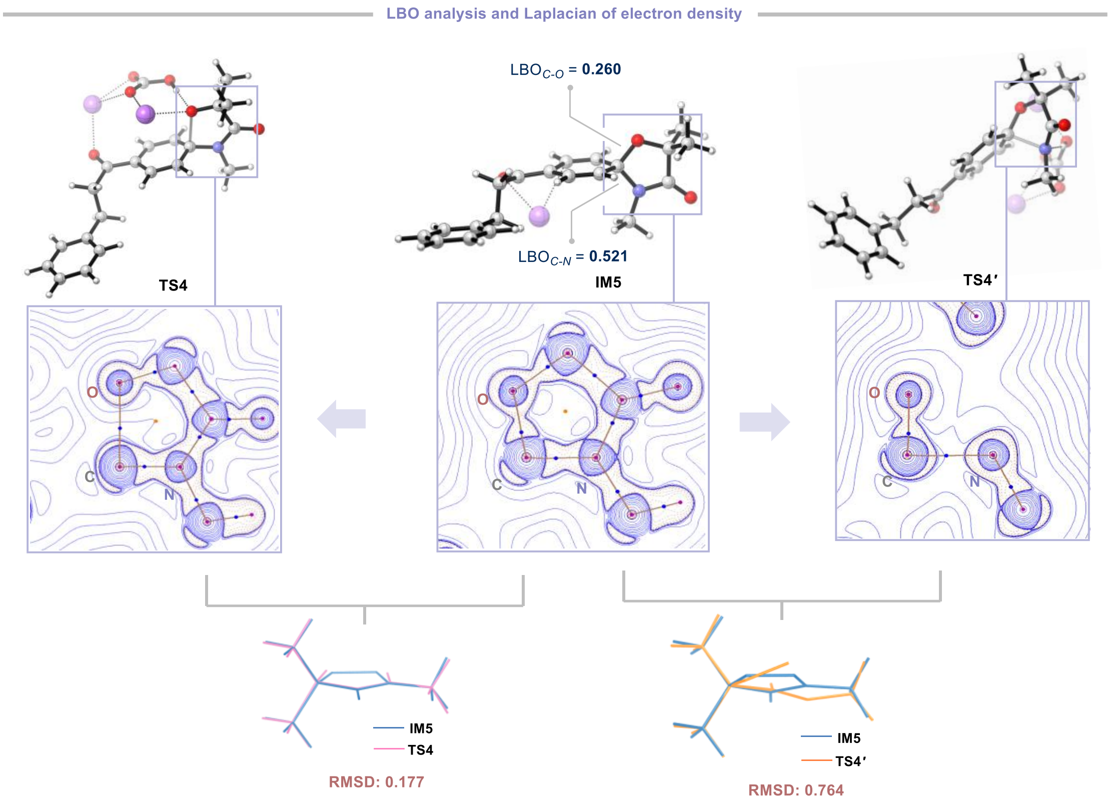

In addition, the analysis of Laplacian of electron density reveals that the spiro five-membered ring in the revised **TS4** also adopts a nearly planar geometry, similar to the precursor intermediate **IM5**. In contrast, **TS4′**, the transition state leading to the C–N cleavage, exhibits pronounced geometric distortion relative to **IM5**. This structural difference is quantified using root mean square displacement/deviation (RMSD) of atomic positions between **IM5** and each transition state. The RMSD value for **IM5**/**TS4′** is substantially higher than that for **IM5**/**TS4** (0.764 *vs.* 0.177), confirming greater geometric perturbation along the C–N cleavage pathway. Therefore, we propose that this ring distortion in **TS4′**, rather than differential hyperconjugation, is a possible factor disfavoring the C–N cleavage pathway. Based on these newly obtained

results, we have revised Figure 6 to reflect the updated geometries and revised the corresponding discussion in the manuscript to clarify the mechanistic rationale. Again, thank you very much for the insightful and constructive comments and valuable suggestions from Reviewer 2.

Reviewer #3:

1. Comment: The manuscript by Li et al. reports an elegant N-heterocyclic carbene (NHC)-catalyzed radical acyl-inserting Smiles rearrangement that enables ipso/para-selective arene difunctionalization. The study is of high quality, featuring a broad substrate scope, well-designed mechanistic experiments, and convincing DFT analysis. The reaction concept is innovative and should attract significant attention in the field of NHC organocatalysis. Overall, the manuscript is well-written and the conclusions are supported by experimental data. However, several minor points should be addressed before publication:

Response: We thank the reviewer for their positive assessment and valuable recommendations. We have revised and further updated the manuscript according to the nice suggestions from this reviewer, and we really hope the carefully revised work will meet with your approval.

2. Comment: 1. There are several minor typographical errors (e.g., “unprecededted” on page 13 should read “unprecedented”; “difunctionaliztion” → “difunctionalization”). Please proofread carefully.

Response: Thanks a lot for the reviewer’s helpful suggestions. In the section of Reaction development, the error “difunctionaliztion” has been corrected to “difunctionalization”; in Discussion section, the error “unprecededted” has been corrected to “unprecedented”. These revisions have been highlighted in the revised version of manuscript.

3. Comment: 2. The proposed spiro dearomatized intermediate shown in the TOC is quite intriguing. Would it be possible to isolate (or intentionally design) such a spiro dearomatization intermediate experimentally? Moreover, in the case of a pyridine substrate, would the reaction be trapped in the spiro dearomatized state? Although this might represent another research direction, it would be very interesting if such conditions could lead to piperidine-type derivatives.

Response: Many thanks for the reviewer’s constructive comments. We are delighted that the proposed spiro dearomatized intermediate featured in the TOC graphic captured the reviewer’s interest. It indeed represents a key conceptual point of our mechanistic hypothesis. Following the reviewer’s nice suggestion, we made substantial efforts to strengthen the experimental investigation into the isolation of the spiro dearomatized species. In addition to our previously successful isolation of the spiro dearomatized and ring-opening products in the radical-clock experiments (substrate **1b**→product **21** in the original version, now updated to substrate **1b**→ product **24**), we conducted several additional experimental studies during the revision process.

First, as shown in the following scheme, we attempt to synthesize a 4-pyridinyl-derived substrate according to the suggestion from the reviewer. However, hydrolysis of the ester group in this 4-pyridinyl scaffold proved unsuccessful, despite a series of condition screening. On the other hand, direct amidation of the ester also failed to offer the desired amide substrate. Then, we turned to the 3-pyridinyl analogue, which we successfully synthesized from 3-hydroxypyridine using a similar synthetic route. Unfortunately, when subjected to our standard catalytic system with either alkyl or aryl aldehydes, no evidence of dearomatized intermediates or difunctionalized products was observed by ^{1}H NMR or LCMS analysis.

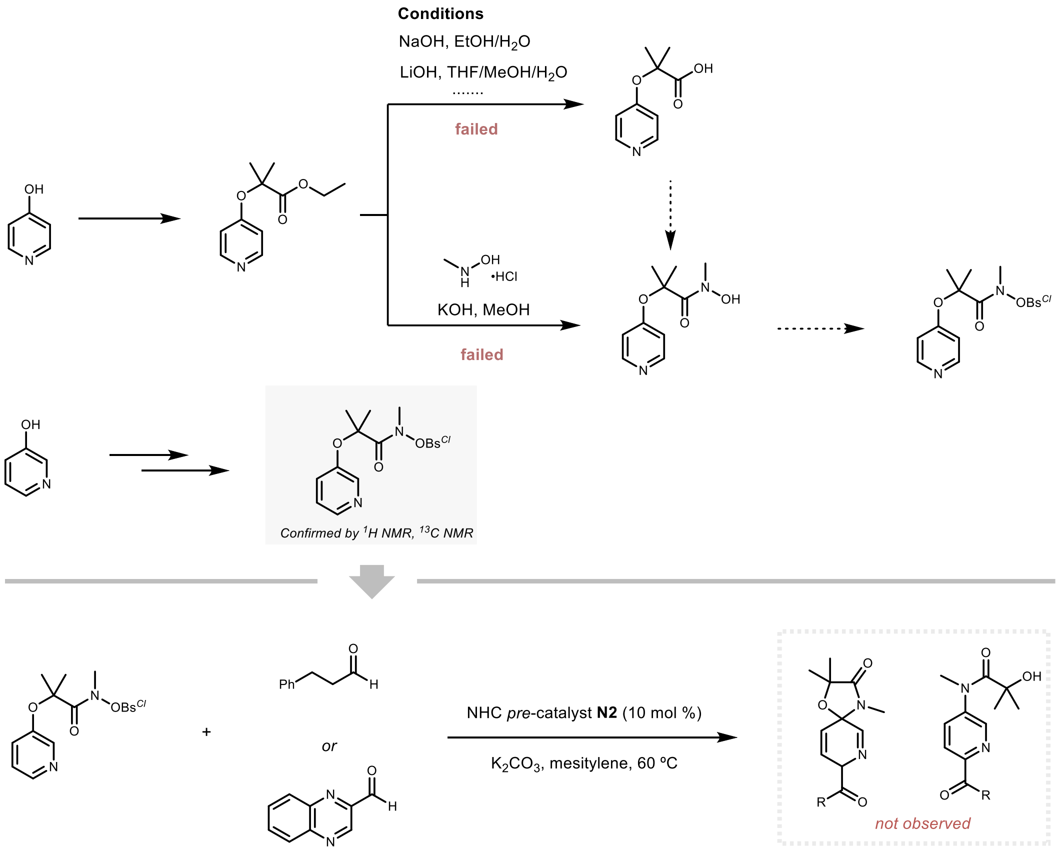

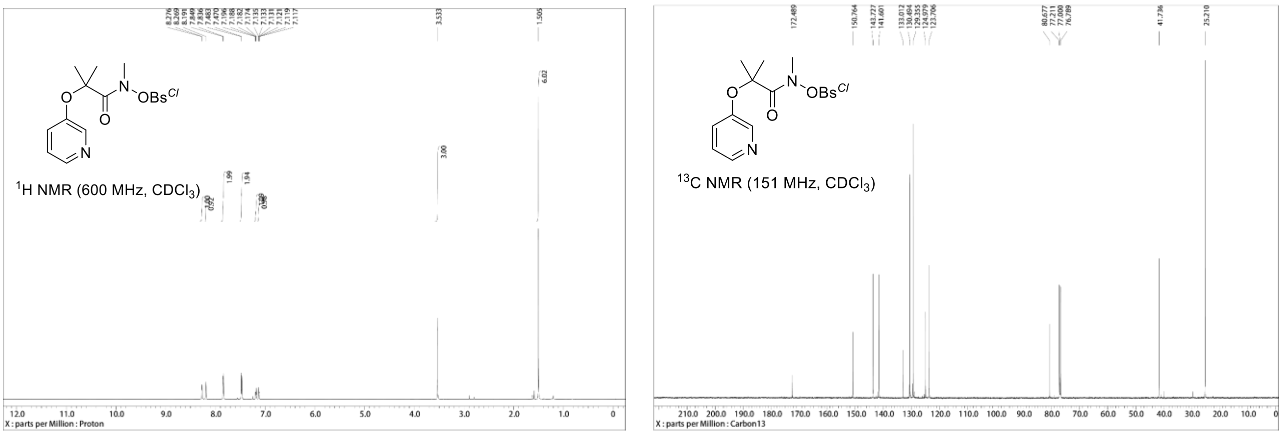

Then, we turned our attention to an alternative 9-anthranyl substrate **A**, which was selected based on the relatively lower aromatic stabilization energy of the anthracene moiety (~84 kcal/mol). We hypothesized that

its reduced tendency toward rearomatization might allow the isolation of the dearomatized intermediate. Encouragingly, by using this substrate, we have successfully isolated a dearomatized intermediate **B** from the reaction mixture. The structure of **B** has been fully characterized by ^{1}H NMR, ^{13}C NMR, HMQC, and HRMS, confirming the formation of the spiro-dearomatized species. These new findings have been added to the revised supplementary information. Again, thank you so much for the reviewer's constructive suggestion, which really helped us to further strengthen the revised work.

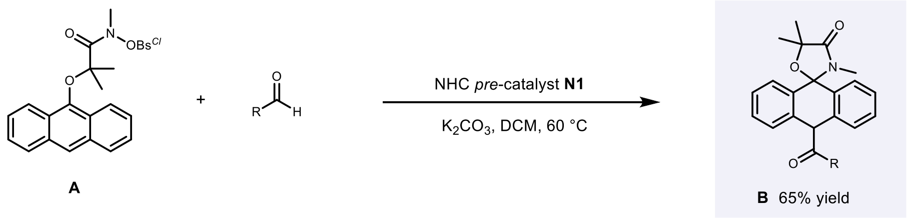

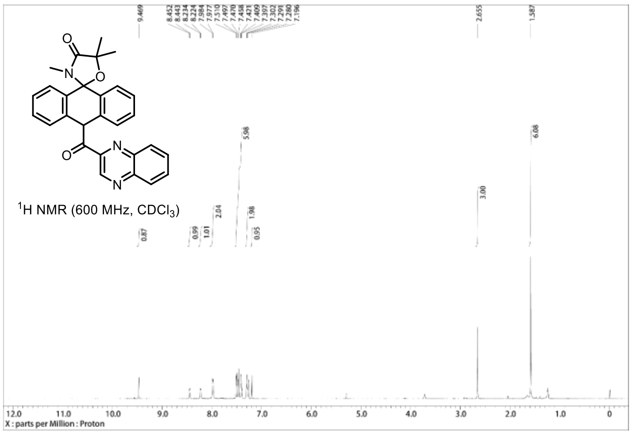

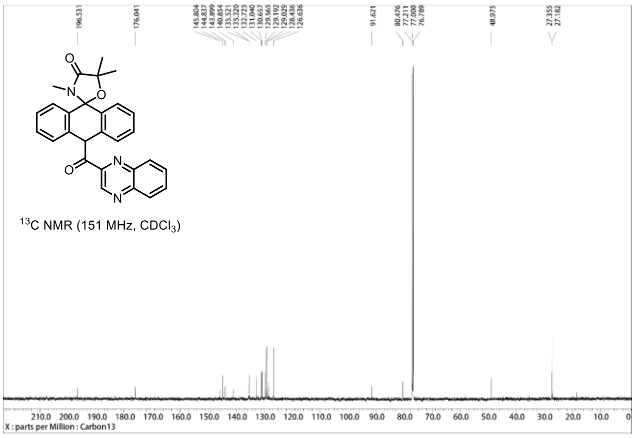

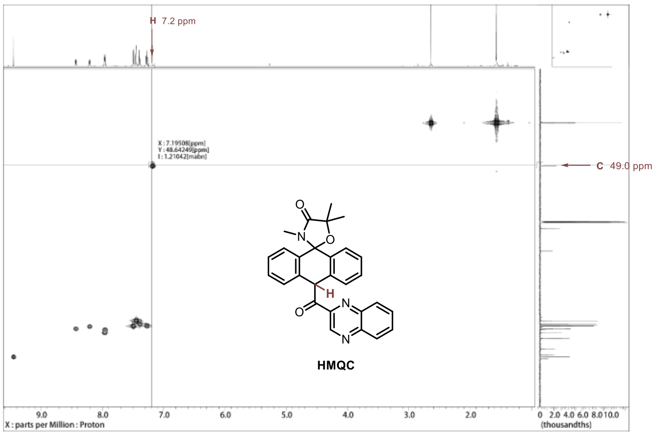

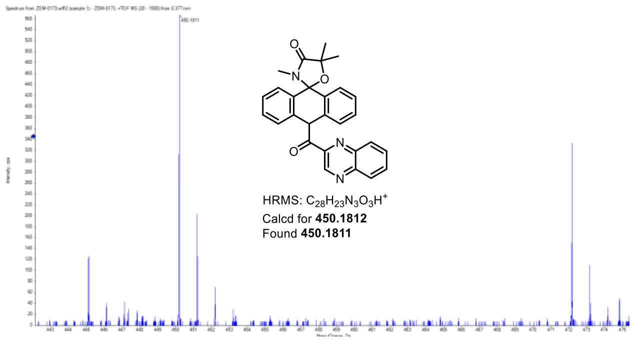

4. Comment: 3. Did the authors systematically examine other possible 4-, 6-, or 7-exo radical cyclization pathways under similar conditions? A brief comment on this aspect would help clarify the reaction's selectivity and scope.

Response: Thank you very much for the reviewer's valuable comments. According to your suggestion, we have systematically investigated the potential reactivities of the dearomatized intermediates with ring sizes

beyond the conventional five-membered transition state. First, we have devoted considerable effort to the design and synthesis of a series of substrates (**a**–**e**) containing the tethers capable of facilitating 4-, 6-, or 7-exo radical cyclization pathways (see the following figures).

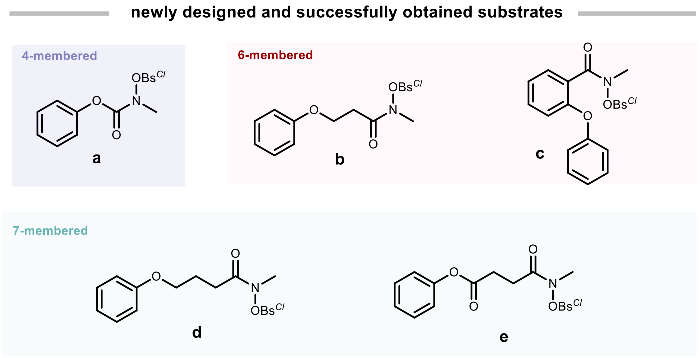

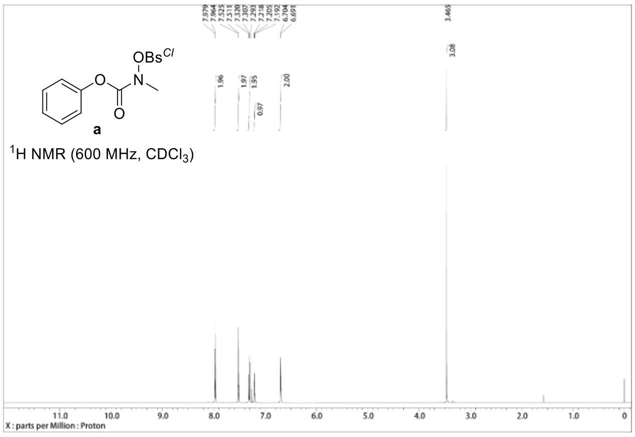

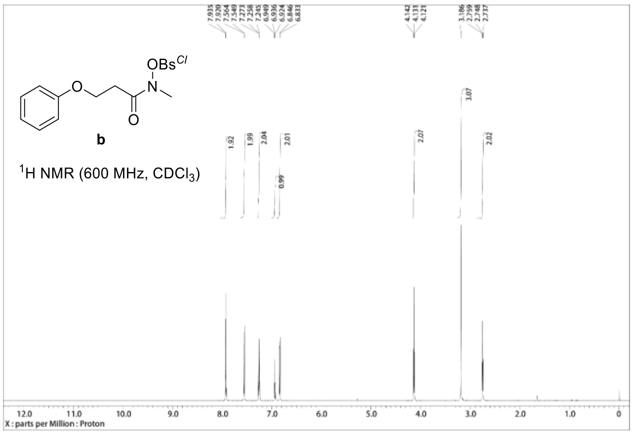

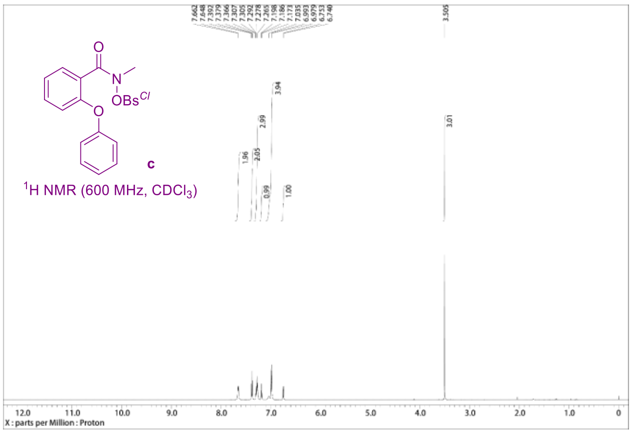

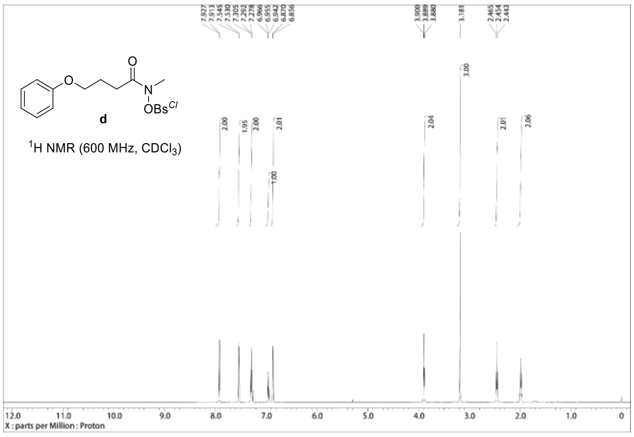

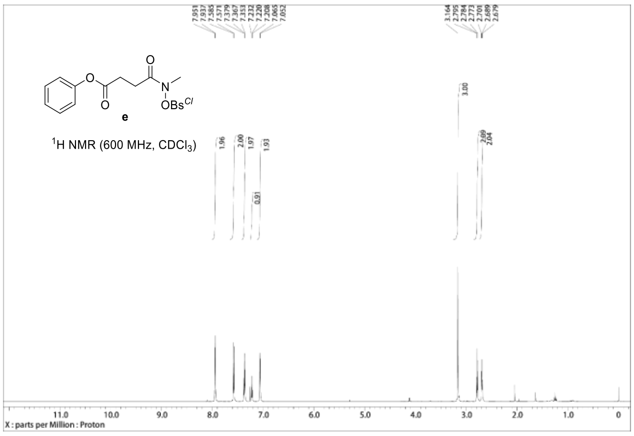

With these newly synthesized substrates in hand, we evaluated their reactivity in our organocatalytic system. Unfortunately, substrates **a**, **b**, **d**, and **e** proved to be completely unreactive under the standard conditions. To exhaustively assess their potential, we conducted an extensive screening of reaction parameters, including a range of NHC pre-catalysts (**N1**–**N7**), various inorganic and organic bases ($KHCO_3$, Cs_2CO_3, K_2CO_3, K_3PO_4, and DABCO), as well as different solvents (DCM, MTBE, MeCN, DMSO, DMF). Despite these comprehensive screening efforts, no detectable conversion to the desired products was observed for these substrates, suggesting that the geometric or electronic constraints imposed by the linkers are incompatible with the key steps of the catalytic cycle—particularly the intramolecular radical addition.

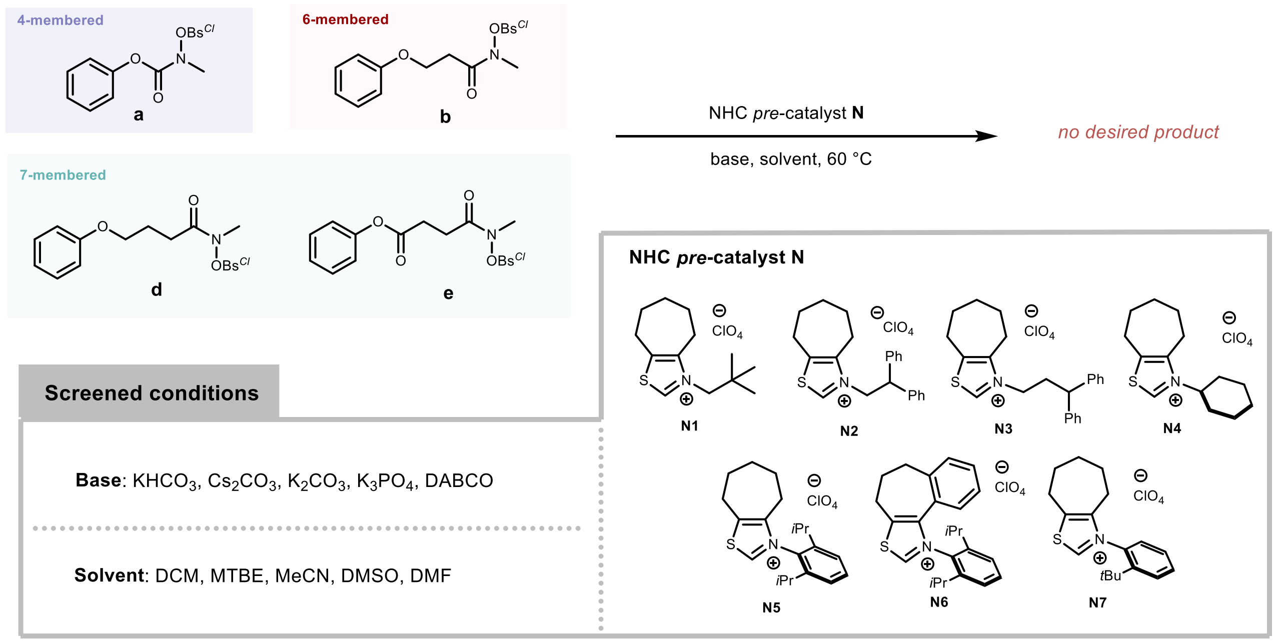

To our delight, we discover that 2-phenoxybenzamide **c**, which was designed to proceed through a six-membered Meisenheimer-type intermediate, could readily undergo the target radical transformation under the standard catalytic conditions, delivering the difunctionalized products **5a**–**5c** in good yields. This outcome confirms that the six-membered cyclization pathway is feasible within our organocatalytic protocol.

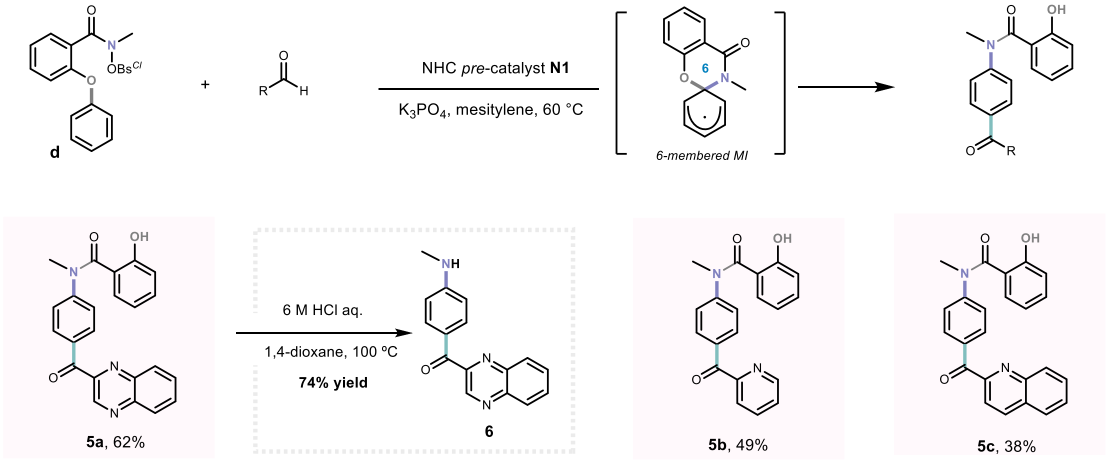

To further confirm the structural assignment of these newly obtained products, **5a** was subjected to acidic hydrolysis using 6 M HCl in 1,4-dioxane under reflux conditions. This amide bond cleavage cleanly afforded the corresponding *para*-acyl-substituted aniline **6**, which was isolated in 74% yield—providing clear evidence for the formation of C–N bond at the *ipso*-postion and the acyl C–C bond at the *para*-position, respectively. For clarity and verification, the ^{1}H NMR spectra of compounds **5a**–**5c** and **6** are provided in the figure below.

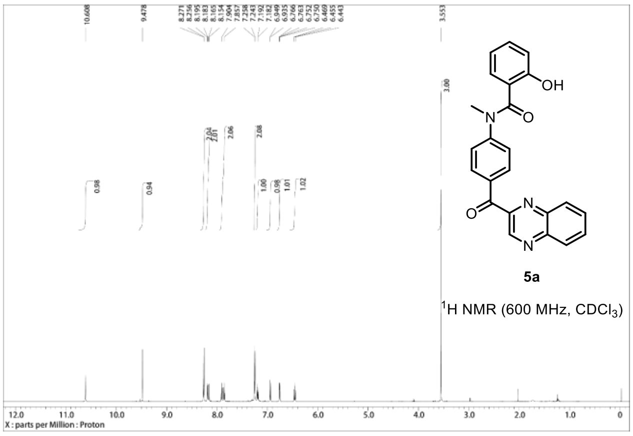

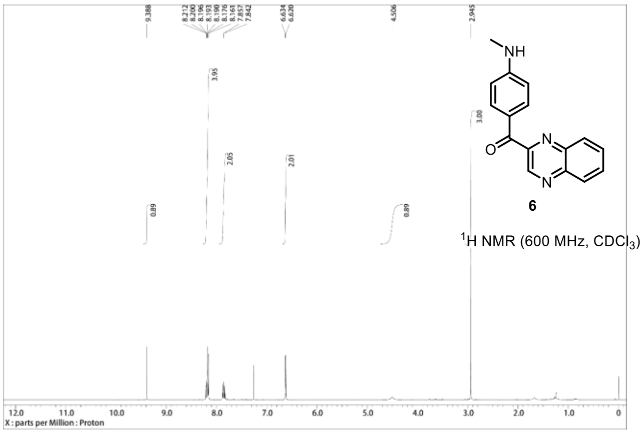

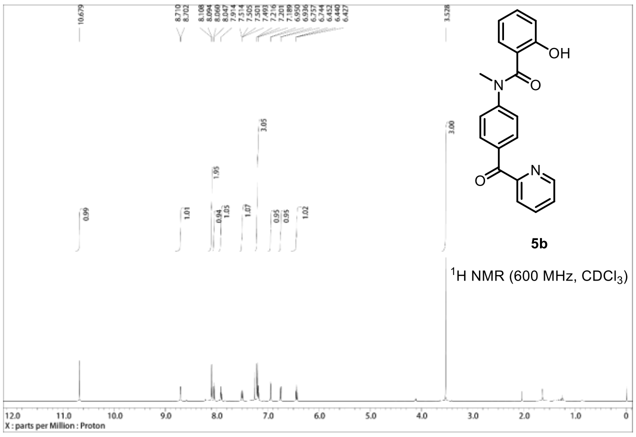

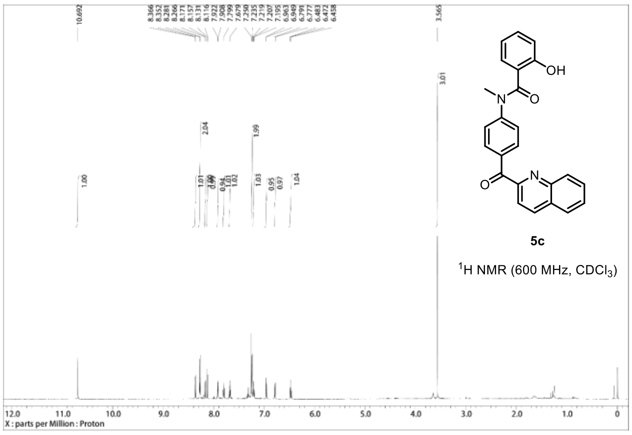

Based on these experimental findings, we have incorporated a new panel—Figure 4B—into the revised manuscript to clearly illustrate the formation of products **5a**–**5c** and the hydrolyzed product **6** (see figure below). In addition, all unsuccessful substrates and corresponding optimization trials—including those involving substrates **a**, **b**, **d**, and **e**—have been systematically documented in the supplementary information.

The revised Figure 4B in manuscript:

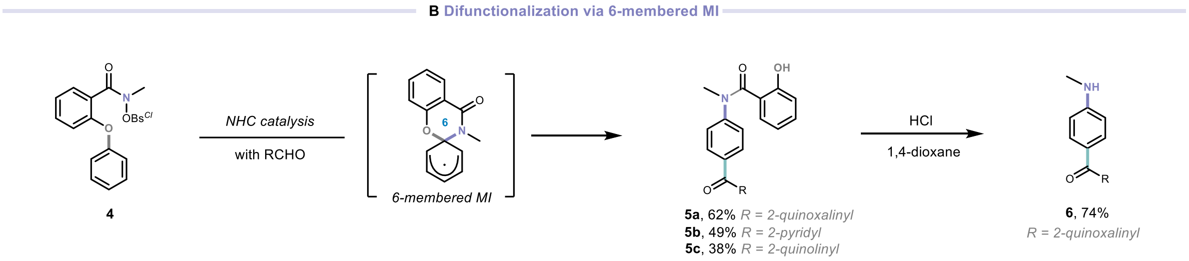

We believe these additional studies significantly strengthen the scope and mechanistic understanding of our method, and we are grateful to the reviewer for giving this valuable suggestion.

5. Comment: 4. Are the NHC pre-catalysts used in this study commercially available? If they are not, please provide appropriate references for their preparation.

Response: Thanks a lot for the reviewer's insightful comments. According to this suggestion from this reviewer, we have carefully reviewed the origin of each NHC *pre*-catalyst employed in our work. We confirm that all of them are either commercially available or have been previously reported in the literature. Therefore, we have added the following clarification to the general information section of the supplementary information: "NHC *pre*-catalysts **N1**[5], **N2**[5], **N3**[6], **N4**[6], **N5**[6], **N6**[7], **N7**[5], **N9**[6], **N11**[7], **N12**[5] were synthesized according to the reported literature procedures. NHC *pre*-catalysts **N8** and **N10** are commercially available". Corresponding references have also been properly cited. These changes have been highlighted in the revised version of supplementary information.

6. Comment: 5. The methodology is indeed powerful, especially the synthetic applications demonstrated in Figures 4A and 5B. If possible, it would be helpful to include representative failed substrates or reaction limitations in the Supporting Information to give readers a more comprehensive understanding of the reaction scope.

Response: Thanks a lot for the reviewer's nice and insightful comments. According to this suggestion from the reviewer, we have summarized the substrates that proved unsuccessful in our catalytic system. As shown in the figure below, this summary has been included in the revised Supplementary Information.

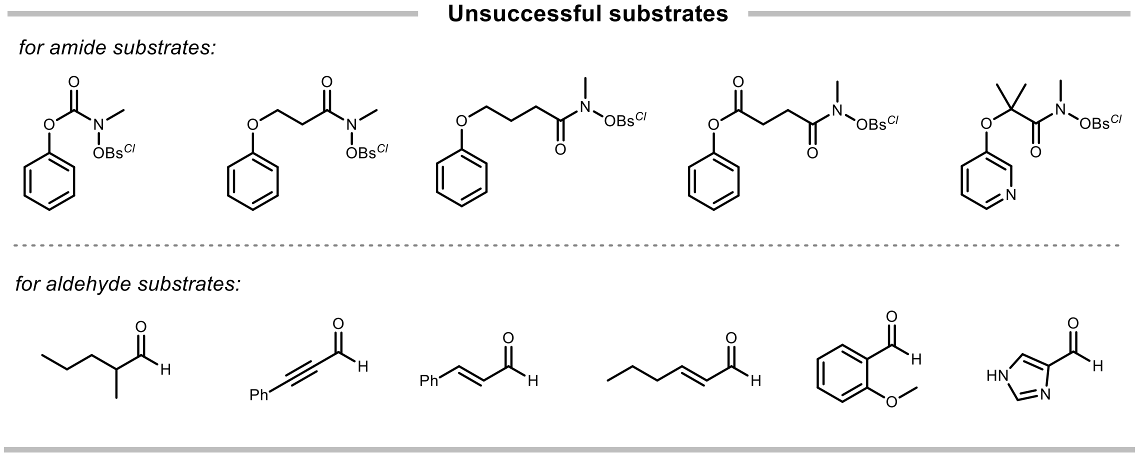

Again, thank you very much for the helpful and construnctive suggestions from the Reviewer 3. We hope our responses and revisions will meet with your approval.

Reviewer #4:

1. Comment: In this manuscript, Li and co-workers describes an NHC-catalyzed ipso/para-difunctionalization of arenes via a radical pathway. The transformation begins with a phenol ether bearing a redox-active N–O bond, which undergoes SET with the electron-rich Breslow intermediate

(formed from the NHC catalyst and an aldehyde) to generate a nitrogen-centered radical, followed by a Smiles rearrangement. The resulting radical variant of the Meisenheimer intermediate is then intercepted by a persistent ketyl radical, ultimately undergoing elimination and rearomatization to deliver the desired difunctionalized product. The reaction design builds on the authors' previous work on radical para-acylation (ref. 60), where only para-functionalization was achieved. In this study, by replacing the alkyl substituent with an oxygen atom, they cleverly convert the original substituent into a leaving group, enabling an O-to-N swap. The substrate scope is broad, and notably, cyclic ether substrates afford ring-expansion products, giving benzoazepine analogues, which I find quite impressive. Overall, the work is carefully done and well suited for publication in Nat. Commun.

Response: We sincerely thank the reviewer for the positive comments and kind recommendations. We hope that our carefully revised work will be finally suitable for *Nat. Commun.*

2. Comment: 1. In Figure 1A, the two panels on the left convey essentially the same information. I recommend combining them.

Response: Thanks a lot for the reviewer's helpful and constructive suggestions regarding Figure 1. We totally agree that the two left-hand panels in the previous version conveyed highly overlapping information. According to the suggestion from this reviewer and the comment (see: Page 6—8 in this response letter) from Reviewer 1[#], we have carefully revised and updated Figure 1A. To clearly demonstrate the revision, we have included a side-by-side comparison of the original and updated versions of Figure 1A in the following figure. Again, thank you so much for this useful and valuable suggestion.

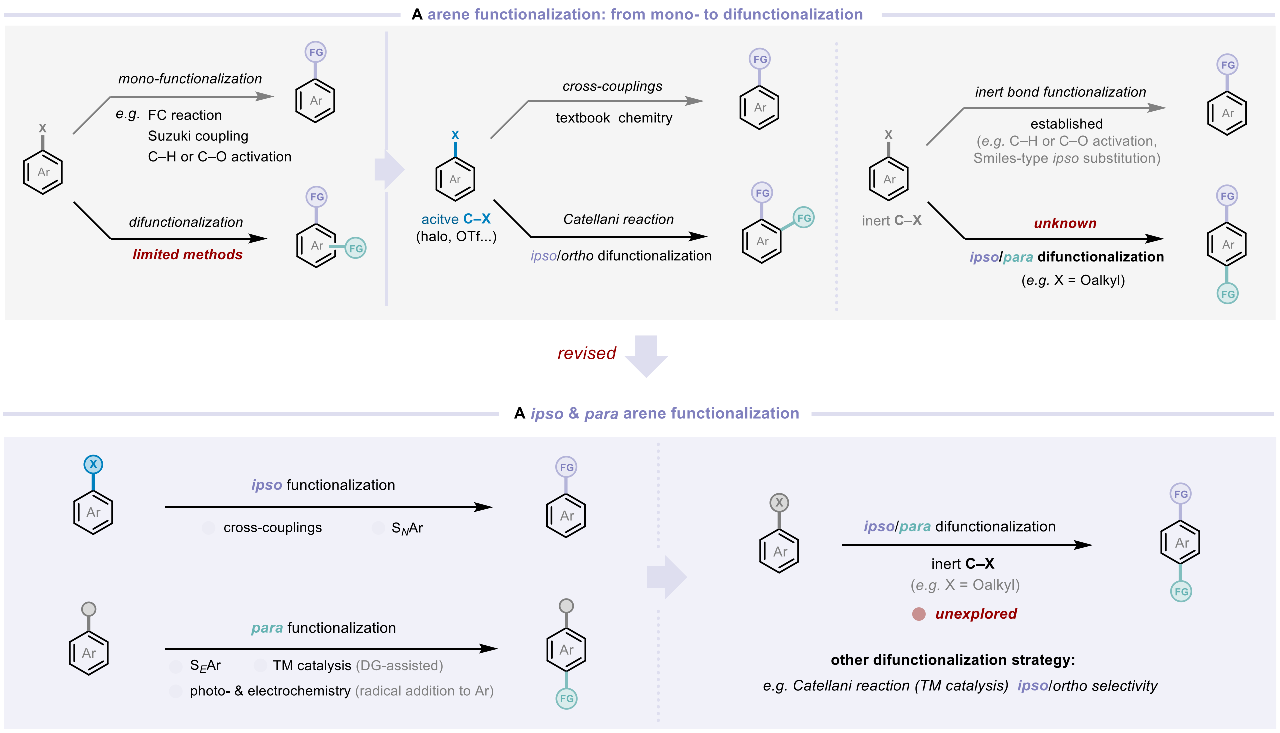

3. Comment: 2. In the discussion of Figure 3, the authors note that "An aldehyde featuring a sulfur-containing alkyl chain exhibited exceptionally high reactivity." Could the authors provide a possible explanation for this exceptional reactivity?

Response: Many thanks for the reviewer's constructive comments. In the previous version of the manuscript, the aldehyde containing a sulfur-based alkyl chain was described as showing "exceptionally high reactivity," based primarily on its high isolated yield (96%). However, in light of the reviewer's comments, we acknowledge that this wording may be misleading, as high yield does not necessarily reflect enhanced intrinsic reactivity. To further clarify this point, we investigated the reaction kinetics of the sulfur-containing aldehyde in comparison with a typical aliphatic aldehyde (**3i**) under identical standard conditions. Both substrates reached full conversion after approximately 12 hours, suggesting that the sulfur-containing aldehyde does not exhibit enhanced intrinsic reactivity relative to alkyl aldehydes. The reason for the higher isolated yield remains unclear at this stage. We speculate that weak noncovalent interactions involving the sulfur atom—such as S···*π* interactions or Lewis basic interactions with the catalyst or intermediates—could potentially reduce side reactions, although no direct experimental evidence is currently available. Therefore, we have revised the discussion associated with Figure 3 to present a more neutral statement: "An aldehyde featuring a sulfur-containing alkyl chain provided 96% yield," and the wording has been appropriately downplayed in the revised manuscript. Again, thank you so much for this helpful comment.

4. Comment: 3. Have the authors explored radical relay coupling? Introducing styrene could potentially enable a three-component condensation.

Response: Thank you very much for the reviewer's valuable comments. Inspired by your comment, we have explored the possibility of a three-component coupling by introducing styrene into the model reaction system of substrates **1a** and **2a**.

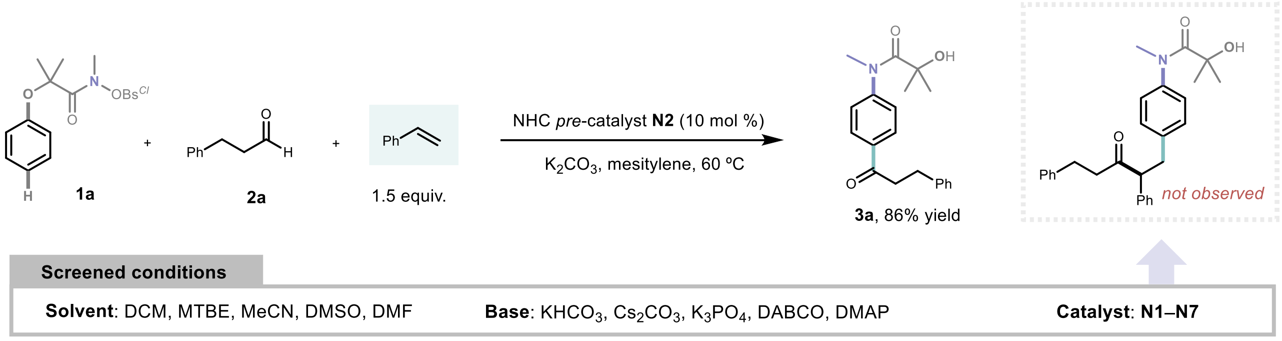

As shown in the above scheme, however, the reaction still proceeded selectively to afford the original two-component product **3a** in high yield, with no detectable formation of the anticipated three-component adduct. Then, we conducted an extensive optimization of the reaction conditions including the solvent, base

and catalyst. Unfortunately, none of these conditions resulted in formation of the desired three-component product; instead, either **3a** was obtained exclusively or decomposition pathways predominated.

5. Comment: 4. In Figure 4, product 5l, dr should be provided.

Response: Thank you very much for the reviewer's helpful comments. The dr value for product **5l** (1:1 dr) has been updated in the revised manuscript.

6. Comment: 5. For substrate 1, is substitution essential? The authors only presented gem-dimethyl and monomethyl analogues. Are unsubstituted derivatives also tolerated?

Response: Many thanks for the reviewer's valuable and insightful comments. According to the reviewer's suggestion, we have synthesized the unsubstituted analogue and subjected the substrate to our standard reaction conditions. To our delight, the transformation proceeded smoothly, delivering the desired product **3bm** in excellent yield (94%), as shown in the following scheme. This result clearly demonstrates that alkyl substitution at the α-position is not essential for achieving this catalytic transformation. As shown in the scheme below, both the starting material and product **3bm** were fully characterized, and their ^{1}H NMR spectra are provided as follows. Based on these results, we have updated Figure 4A in the revised manuscript to include this important case, and all associated experimental details, spectral data, and characterization results have been added into the revised supplementary information.

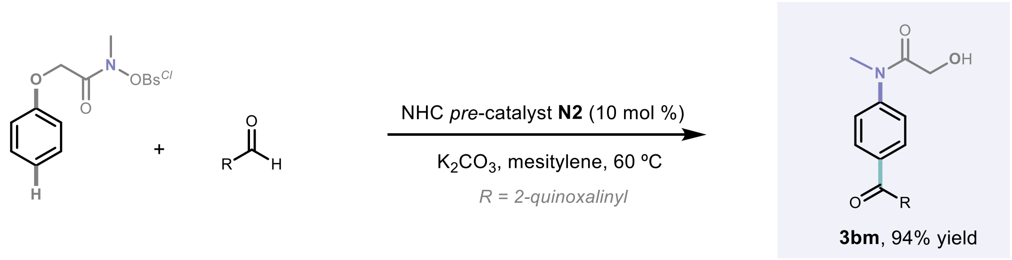

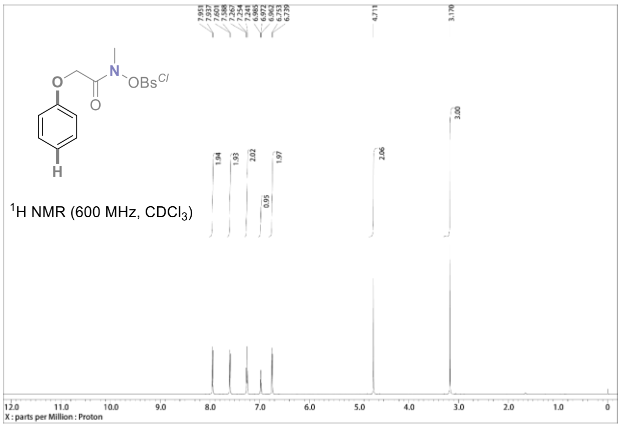

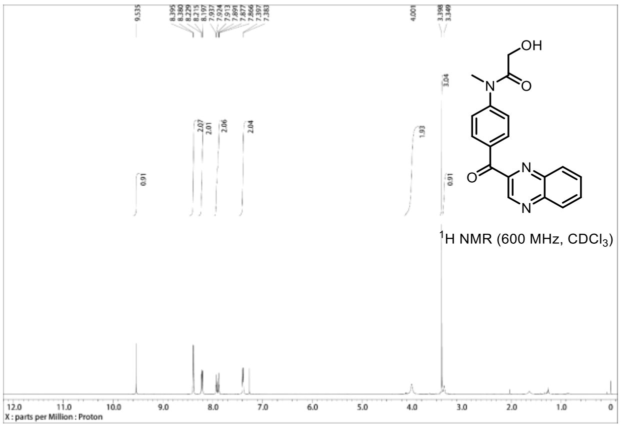

Once again, we deeply appreciate the reviewer's forward-looking perspective, which has directly inspired these valuable experimental advances.

7. Comment: 6. Page 6, line 122, "bis-arene substrate 3at" should be "biaryl product 3at".

Response: Thanks a lot for the reviewer's helpful comments. According to the reviewer's suggestion, "*bis*-arene substrate (**3at**)" has been corrected to "biaryl substrate (**3at**)".

8. Comment: 7. Can the authors comment more on the N-leaving group?

Response: Thank you very much for the reviewer's constructive comments. Actually, the nature of the *N*-leaving group plays a pivotal role in facilitating the initial single-electron transfer event, which triggers the entire catalytic radical cycle. In our earlier optimization studies (in previous Table S5, also summarized in the upper panel of the figure below), we systematically evaluated a series of sulfonyl leaving group (LG). It seems that the electron-withdrawing group on benzenesulfonyl group, such as chloro significantly enhanced reactivity. In contrast, electron-donating groups (**LG1**–**LG3**) led to markedly reduced yields. However, the strong electron-withdrawing group NO_2 (**LG4**) is not suitable for such reaction, might due to its ability to engage in competing SET pathways (*e.g.* Rovis's work: *J. Am. Chem. Soc.* **136**, 14674 (2014)). Notably, our previous work also identified 4-chlorobenzenesulfonyl as the optimal *N*-leaving group after extensive screening (*e.g. Angew. Chem. Int. Ed.* **61**, e202116629 (2022)).

In light of the reviewer's suggestion, we have now extended our investigation beyond sulfonyl-based leaving groups to explore alternative motifs. As shown in the lower part of the following figure, we successfully synthesized two new substrates featuring benzoyl (**A** and **B**) and phthalimide as potential N-leaving groups (**C**), and their spectrum of ^{1}H NMR and ^{13}C NMR are provided as follows.

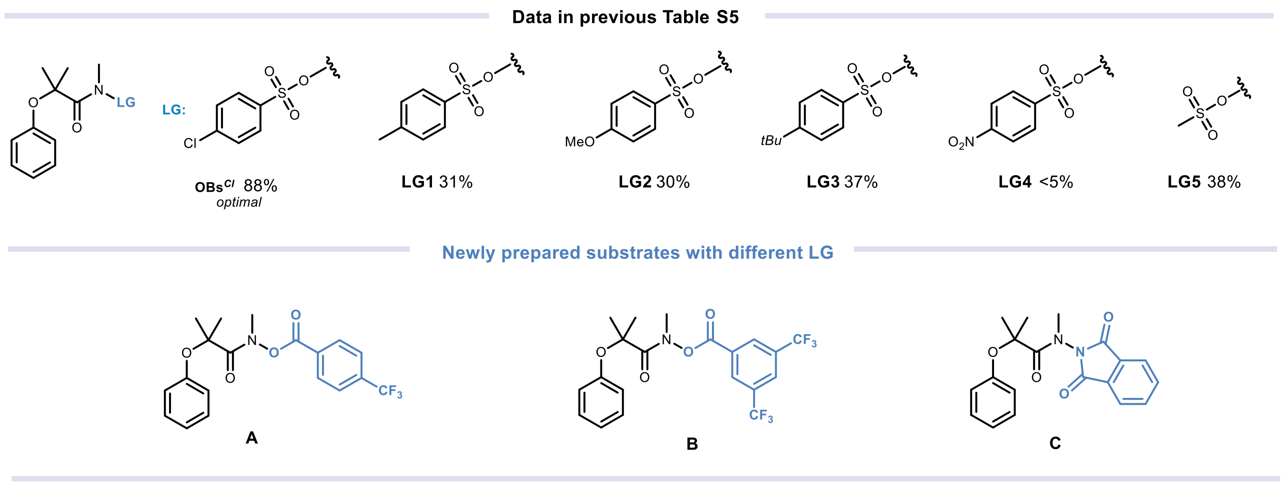

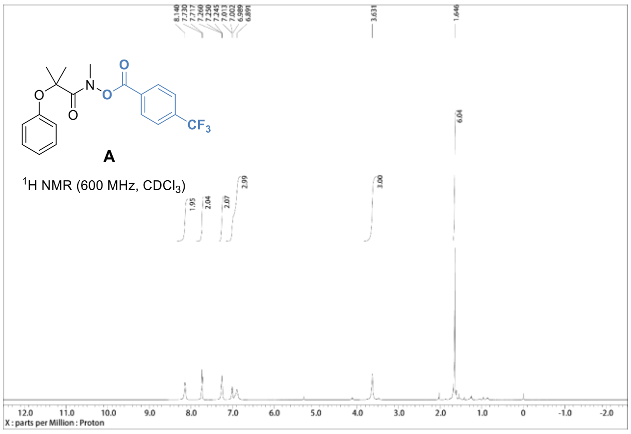

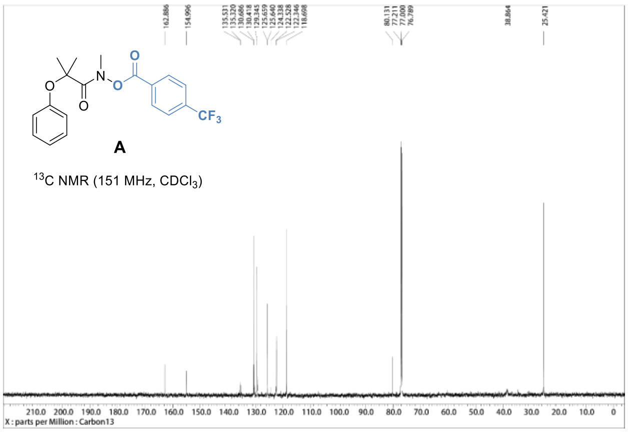

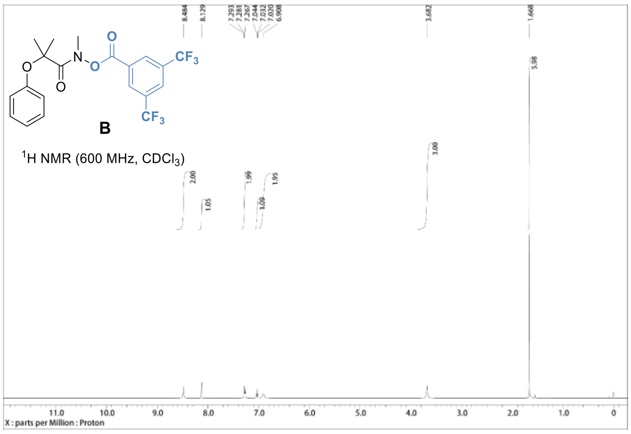

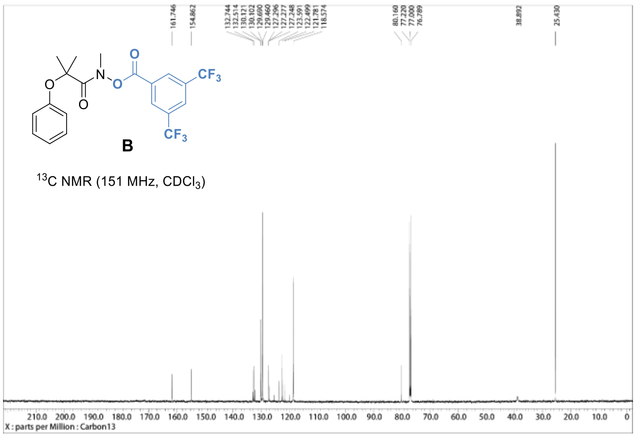

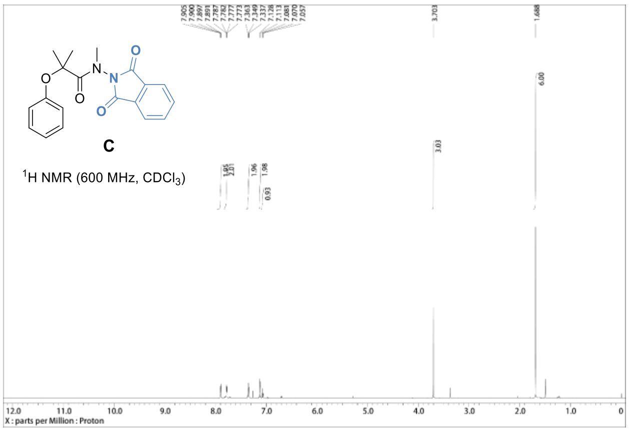

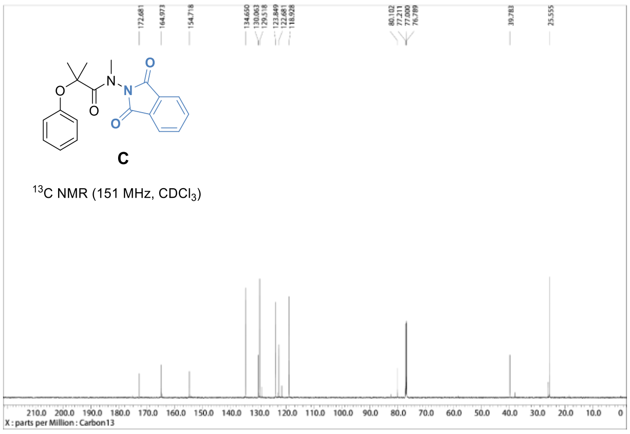

Upon subjecting these substrates to our standard reaction conditions, we observed some reactivity with the benzoyl derivatives: substrates **A** and **B** afforded the desired product in 27% and 37% yield, respectively.

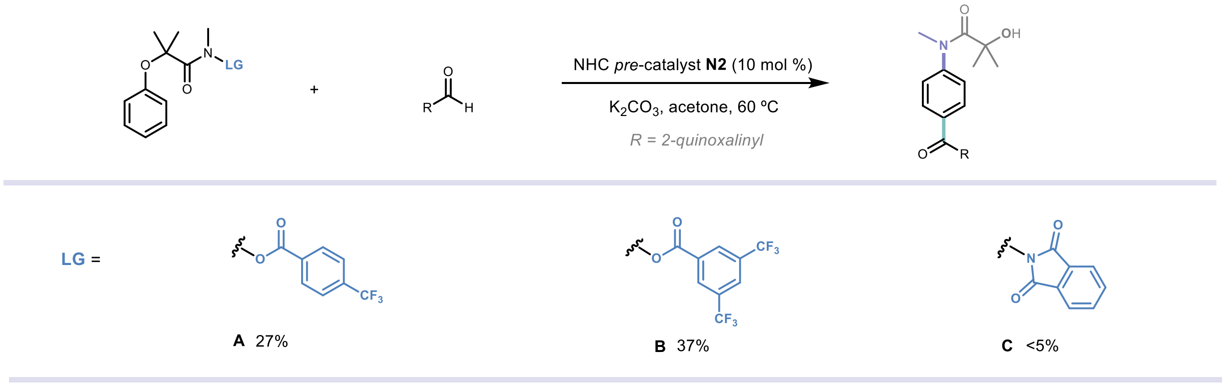

However, the phthalimide-containing substrate is completely unreactive, likely due to its poor leaving-group ability under our organocatalytic conditions All the relevant discussions, including these newly added experimental data and compound characterizations have been incorporated into the revised supplementary information.

Again, many thanks for the insightful and constructive suggestions from Reviewer 4. We hope our responses and revisions will meet with your approval.

Reviewer #1:

1. Comment: The authors have clearly invested significant effort into the revision, and the new computational data provides a more detailed view of the reaction profile. Despite these improvements, the manuscript still suffers from a fundamental lack of internal consistency. The transition in the mechanistic paradigm from the authors' own Nature Catalysis 2024 report is particularly problematic because it lacks a rigorous comparative foundation. This ambiguity prevents a definitive endorsement of the proposed mechanism. Major revision is necessary to resolve these critical issues.

Response: We greatly appreciate the valuable and objective comments provided by the reviewer and we deeply appreciate the time and expertise you have devoted to evaluating our work. We are truly grateful that the reviewer acknowledged the substantial improvements made to our manuscript in the previous revision. We have carefully examined the valuable second round of feedback, especially regarding the consistency of the mechanistic interpretation relative to our previous *Nature Catalysis* 2024 report. In response, we have carefully provided a demonstration to clarify that, although conceptual connections exist, the current work represents a meaningful advancement. Furthermore, in light of your valuable comments on the reaction mechanism, we have carefully updated the DFT calculations and provided a more in-depth mechanistic discussion. A detailed comparison between the 1,2-migration pathway and the direct radical-radical coupling mechanism was conducted, and supported by updated DFT calculations performed under identical methodological settings. In fact, our previous *Nature Catalysis* paper suggested that both reaction pathways are plausible, a finding that aligns well with the conclusions in this updated work. We hope that the carefully revised manuscript is now suitable for publication in *Nature Communications*.

2. Comment: Major comment

While the focus on para-selective acylation is now better emphasized, the conceptual leap from the 2024 report remains tenuous. The previous study established a platform based on intramolecular radical addition at the ipso-position. The current work replaces this with a Smiles-type rearrangement to generate the Meisenheimer intermediate. Although this change is synthetically useful, it represents an incremental modification of the initiation step rather than a fundamental shift in reactivity. The authors must clarify whether this work introduces a new class of NHC catalysis or if it is simply a strategic extension of their established radical Meisenheimer framework. A comparative scheme illustrating the divergent pathways of the two systems would clarify this distinction.

Response: We sincerely thank the reviewer for the insightful and constructive comments. We fully agree that both this work and our 2024 study are rooted in Meisenheimer intermediate chemistry, and we appreciate the reviewer's recognition of this connection. We also acknowledge that the current study builds upon our prior

work. Nevertheless, we respectfully clarify that the key distinction lies in the fundamentally different bond-cleavage selectivity of the Meisenheimer intermediate. In our earlier work, the reaction proceeded via cleavage of the newly formed C–Y bond, resulting in arene mono-functionalization. In contrast, the current system selectively cleaves the pre-existing C–X bond, enabling para-selective acylation accompanied by simultaneous functionalization at the C–X site. This inversion of bond-cleavage preference gives rise to an efficient arene difunctionalization manifold, which we believe constitutes a meaningful and non-trivial advance beyond our prior study. Importantly, this work represents a significant advance in the field of Smiles rearrangement, as it enables the installation of a functional group onto the migrating aryl unit during the rearrangement process. In line with the reviewer’s suggestion to better clarify the mechanistic differences between the two systems, we have added a new figure to the revised Supplementary Information, accompanied by an appropriate discussion.

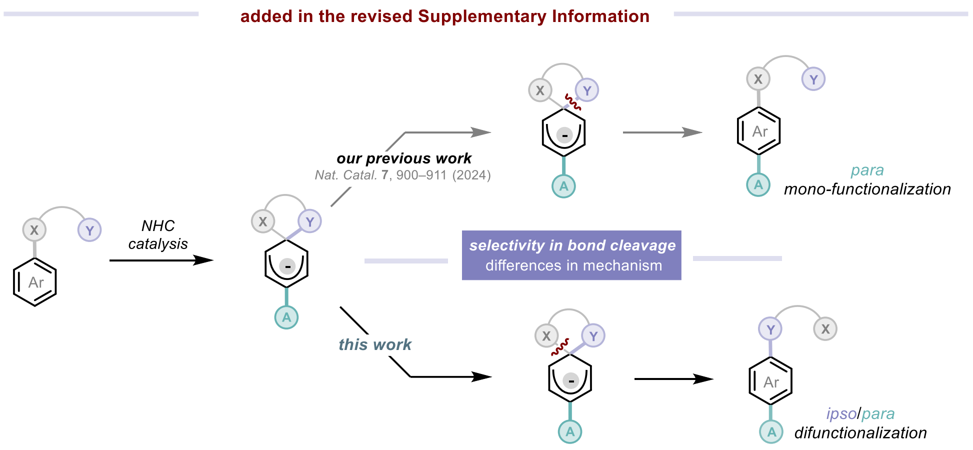

In addition, we are truly grateful that the reviewer recognized our work as “synthetically useful.” While the utility of this methodology has been documented in our previous revision, we would like to further highlight representative transformations that are NOT accessible using our previous *Nature Catalysis* method. Notably, the *ipso* heteroatom translocation unlocks challenging ring-expansion processes of bicyclic substrates, a challenging transformation that was previously inaccessible. As illustrated in the scheme below, benzopyran-derived substrates (six-membered rings) can be efficiently converted into benzo[*b*]azepine analogues (seven-membered rings). These examples further highlight the unique synthetic power of this organocatalytic strategy.

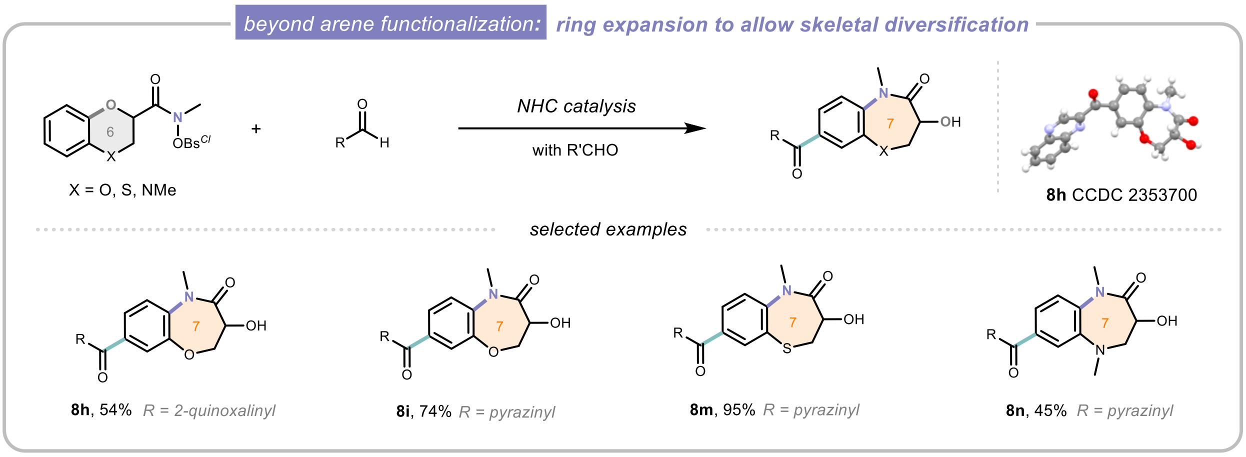

Furthermore, according to the reviewer's suggestion, we have also revised Figure 1C in the main manuscript (see the following figure) to include the pathway of our previous work (red marked). The revised figure clearly illustrates the mechanistic distinction of the two pathways. Accordingly, the description text "which in turn promotes the desired rearomatization. Inspired by this principle……" has been modified to "which in turn promotes the desired rearomatization *via* C–X bond cleavage and achieves the mono-functionalization. Inspired by this principle…"; and "Subsequent rearomatization of this intermediate accomplishes ipso/para difunctionalization" has been modified to "Subsequent rearomatization *via* alternative C–Y bond cleavage accomplishes ipso/para difunctionalization". We hope this addition and revision clarifies the distinction, and underscores the conceptual advance of the present methodology.

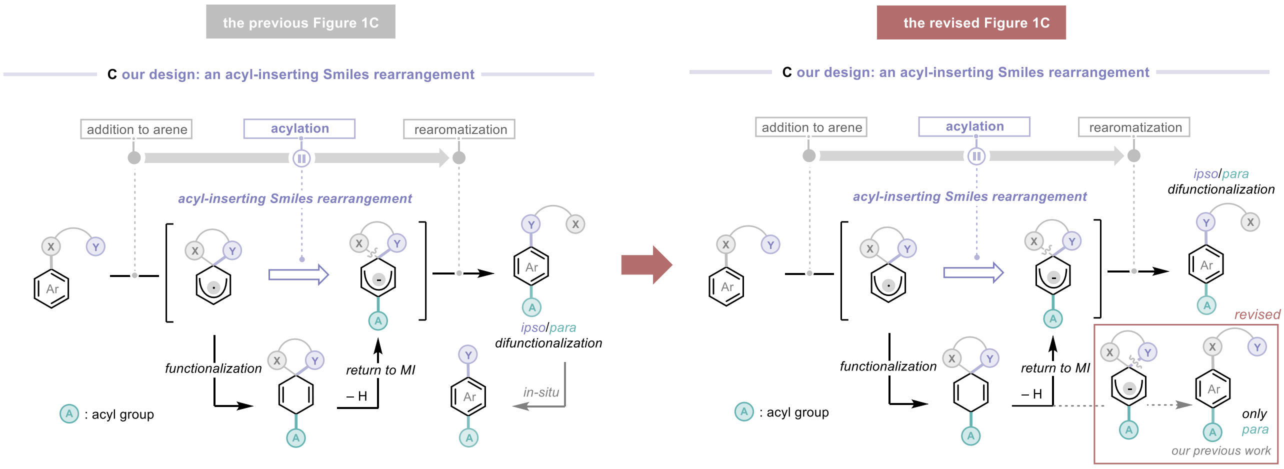

3. Comment: The most significant weakness in the revised manuscript is the unexplained departure from the previous mechanistic model. In their earlier work, the authors proposed a 1,2-migration from a σ-bonded intermediate as the key product-forming step. The current manuscript, however, proposes direct radical-radical coupling between the Meisenheimer intermediate and an NHC-bound ketyl radical. The provided DFT data does not justify this shift in interpretation. It remains unclear why the 1,2-migration

pathway, previously considered dominant, is now discounted. A direct computational comparison between the 1,2-migration and radical-radical coupling pathways must be performed under identical methodological settings. Without a clear explanation for this divergence, such as substrate-specific electronic effects or reaction conditions, the mechanistic narrative appears internally inconsistent with the authors' own precedents.

Response: We thank the reviewer for the insightful comments on the mechanistic pathway. We would like to respectfully emphasize that the mechanism proposed in the current study is still consistent with our previous work and does not represent a deviation. In our earlier study, we identified two competing pathways—1,2-migration and direct C1 radical-radical coupling—without bias toward a single dominant pathway. As illustrated in the figure below (taken from our previous report), DFT calculations showed that the transition-state Gibbs free energies for direct C1 coupling and 1,2-migration are very close (−32.7 vs. −31.4 kcal/mol). These results indicate that both pathways are kinetically feasible and likely coexist under the reaction conditions. Actually, we had described the direct C1 coupling route in the previous text as: "*An alternative possible route to **IM-5** involves the direct cross-coupling of **IM-3** with the ketyl radical **IM-2'**, a process that proceeds through a transition state **TS$_{3\text{-}5}$**, surmounting an energy barrier of 7.8 kcal/mol*". We apologize this point was not sufficiently emphasized in our previous response.

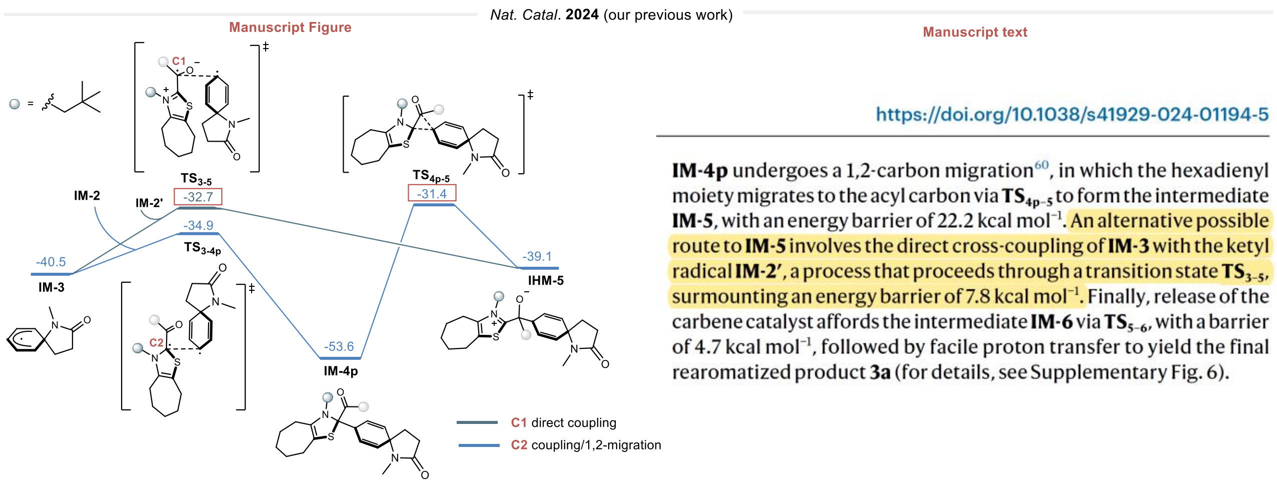

In the present work, we propose that both mechanistic pathways are, in principle, possible and accessible. As depicted in the scheme below, C1- and C2-centered radical intermediates can dynamically interconvert via a spin-center shift (SCS) process, thereby coexisting under the reaction conditions. Consequently, whether the reaction proceeds through direct C1 radical coupling or via C2 coupling followed by 1,2-migration was suggested by depending on the structure of the NHC catalyst. Previous studies by Karl A. Scheidt and co-workers (*Angew. Chem. Int. Ed.* **60**, 17925, (2021)) have shown that NHCs bearing bulky *N*-substituents favor radical coupling at the C1 position due to steric shielding of the C2 site by the *N*-aryl

group. As a result, most prior work has predominantly invoked a C1 direct coupling pathway. By contrast, less sterically encumbered NHCs reduce this bias, rendering both C1 and C2 coupling pathways accessible.

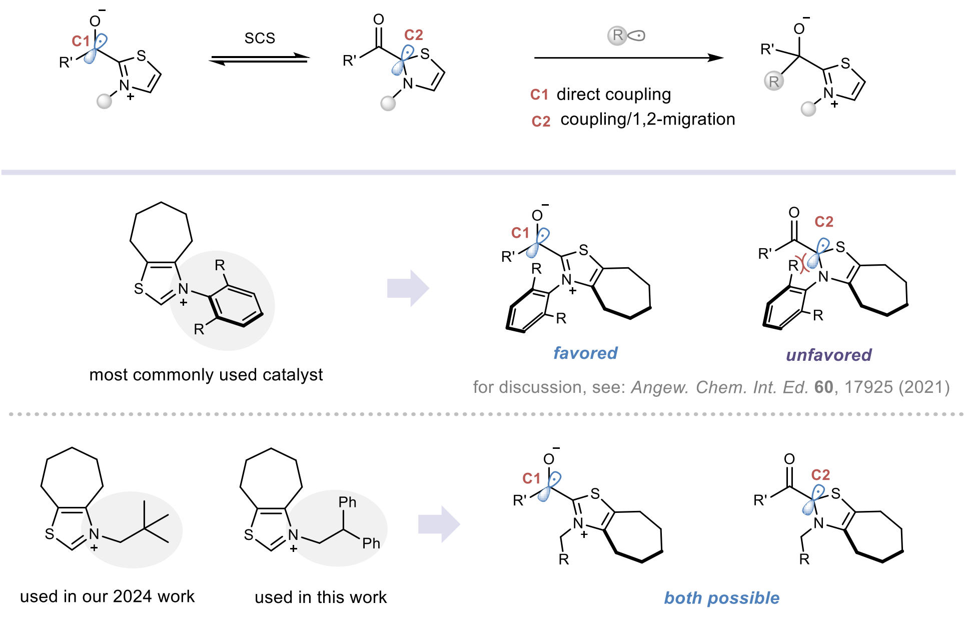

In the present manuscript, we chose to place less emphasis on the 1,2-shift pathway in the main text and instead focused primarily on the C1 coupling pathway, as the primary aim of this study is to elucidate the C–X bond cleavage event rather than to comprehensively dissect the radical acylation mechanism. We therefore considered an in-depth discussion of the acylation pathway to be beyond the primary aim of the study. Another important reason is that several sterically demanding NHC catalysts employed in this work (Figure 2, **N6** and **N7**), which are theoretically disfavored for 1,2-migration, still delivered the desired products efficiently. This observation suggests that the C1 coupling pathway may represent a more general mechanistic scenario. Nevertheless, we actually do not rule out the involvement of the C2 coupling/1,2-migration pathway. Indeed, this pathway was previously considered and discussed in our Supplementary Information, as shown in the figure below:

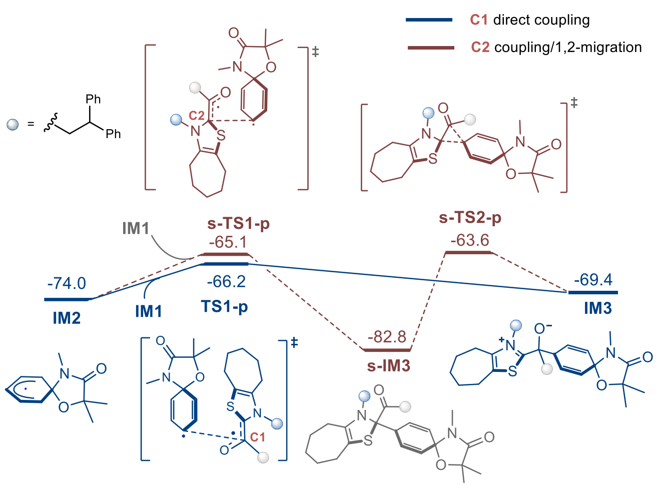

Prompted by the reviewer's insightful comment, we revisited our previous computational data to reassess the validity of the proposed pathways. Intriguingly, in this work, the previously identified coupling transition states show negligible spin density at both the C1 and C2 centers (additional remarks: *the coupling transition states in our previous Nature Catalysis paper show significant spin density*), a feature that could be interpreted as indicative of a polar, ion-pair-based mechanism rather than radical–radical coupling. However, subsequent DFT analysis reveals that the single-electron transfer necessary to generate such ion-pair species from **IM1** and **IM2** is associated with an exceedingly high activation barrier (>100 kcal/mol), rendering this pathway kinetically inaccessible. These findings strongly argue against a polar mechanism and instead still support a radical-mediated process.

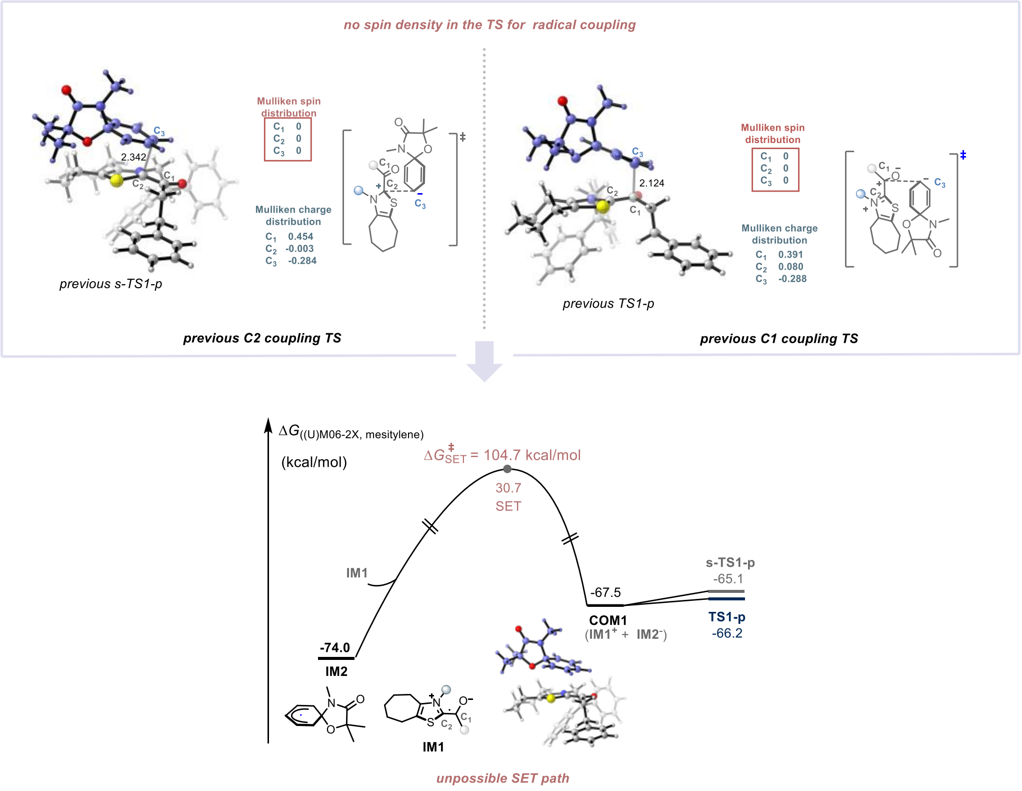

Based on these findings, we recognized that the previously proposed transition states are not reliable and should be excluded. We subsequently carried out an extensive conformational search to identify the correct radical–radical coupling transition states. This effort led to the successful identification of eight new transition-state structures (four corresponding to C1-selective and four to C2-selective pathways), each exhibiting the expected spin density distribution. The lowest-energy transition states were subsequently selected as representative structures for further analysis.

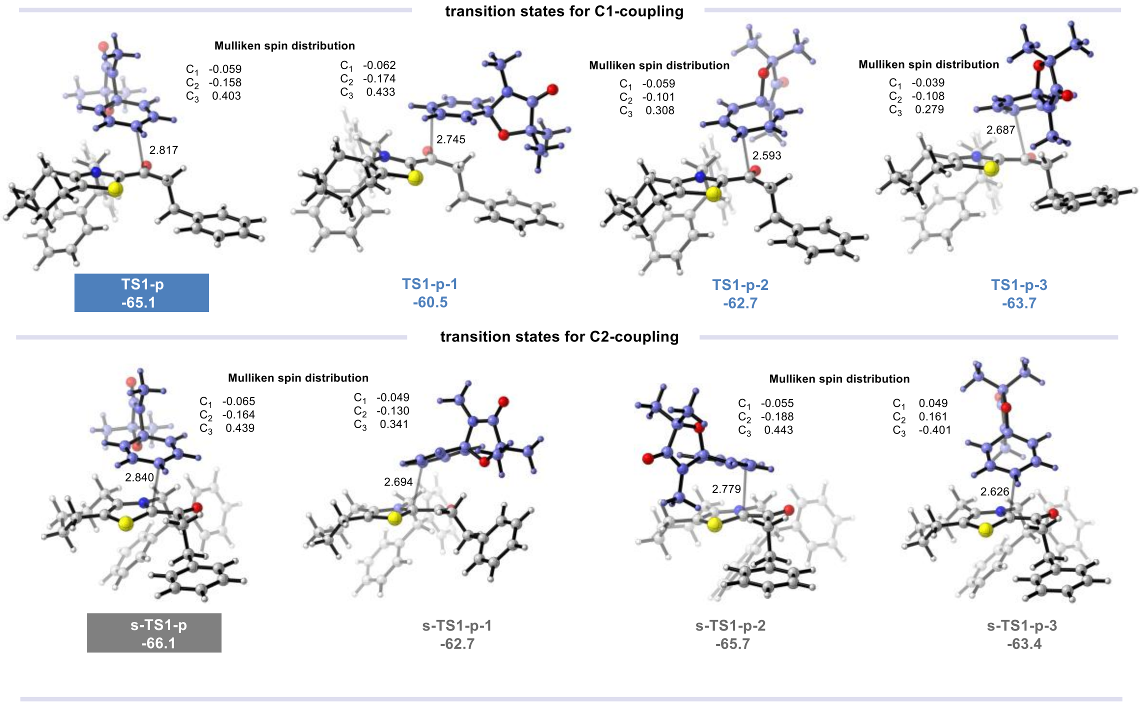

Accordingly, we have updated the energy profiles of the radical coupling pathways (from **IM2** to **IM3**), as shown below. The highest energy barriers for the two pathways remain very similar (−65.1 vs. −63.6 kcal/mol). Therefore, although the transition states have been revised and corrected, the updated results continue to support our original conclusion that both pathways can coexist under the standard reaction conditions.

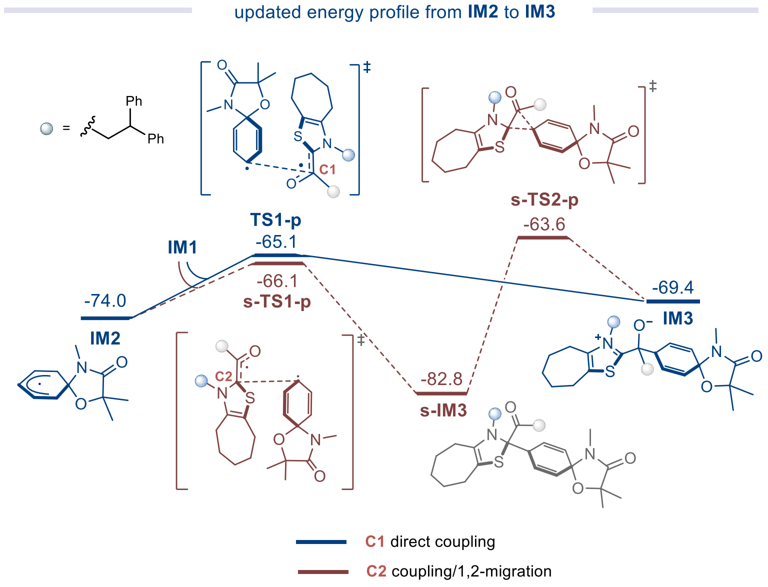

In summary, the revised computational results have now been incorporated into both the manuscript and the Supplementary Information. To provide a clearer comparison of the two pathways, we have added a new Figure S3 in Section 10 of the Supplementary Information, along with the corresponding discussion. In

addition, the main text has been updated to include the note “(for detailed discussion, see Figure S3 in the Supplementary Information)” following the sentence “In addition, another radical coupling pathway involving a 1,2-carbon migration process is also possible.” We sincerely thank the reviewer for the rigorous and insightful comments, which have enabled us to identify and correct this important issue in the present work. Again, thank you so much for this helpful comment.

4. Comment: Minor comment

The authors have made a helpful effort to incorporate the role of the carbonate base. However, the current model assumes a freely solvated carbonate anion in an organic medium, which is a significant idealization given the heterogeneous nature of K2CO3 or Cs2CO3. While a full re-evaluation using contact ion pair models may be beyond the scope of this study, the manuscript would benefit from a more transparent discussion regarding this simplification. Considering that the computed energy differences between competing pathways are relatively modest, it is important to acknowledge how the modeling of the base might influence the calculated barriers, particularly for the deprotonation step at TS4.

Response: We sincerely thank the reviewer for acknowledging our previous efforts in incorporating the role of the base, and many thanks for providing valuable suggestions for further improvement. We fully agree that K_2CO_3 and Cs_2CO_3 typically exist as insoluble solid powders in organic solvents, thus constituting a heterogeneous system. Thus, modeling them as fully solvated ions in our calculations is indeed a simplification and represents an idealization. In response to this important comment, we have conducted a series of additional experiments and DFT calculations to further clarify and validate our computational methodology.

To probe the effect of base solubility on reaction selectivity (particularly **TS4**), we carried out a series of additional experiments. Using *n*Bu_4OAc in mesitylene **provided a homogeneous system**, in which C–O bond cleavage proceeded efficiently with high chemoselectivity (condition A). We next investigated partially heterogeneous or heterogeneous systems: potassium 2-ethylhexanoate (RCO_2K), an organic base with some solubility in THF or in a mesitylene/THF (1:1) mixture, exists as a partially dissolved suspension rather than a fully homogeneous solution. Under these conditions, exclusive formation of the C–O bond cleavage product **3a** was still observed (conditions B and C). Similarly, despite the poor solubility of Cs_2CO_3 or RCO_2K in mesitylene, the reactions consistently afforded **3a** without any detectable C–N cleavage products (conditions D and E). **These experimental results collectively suggest that the solubility of the base does not play a determining role in bond cleavage selectivity.**

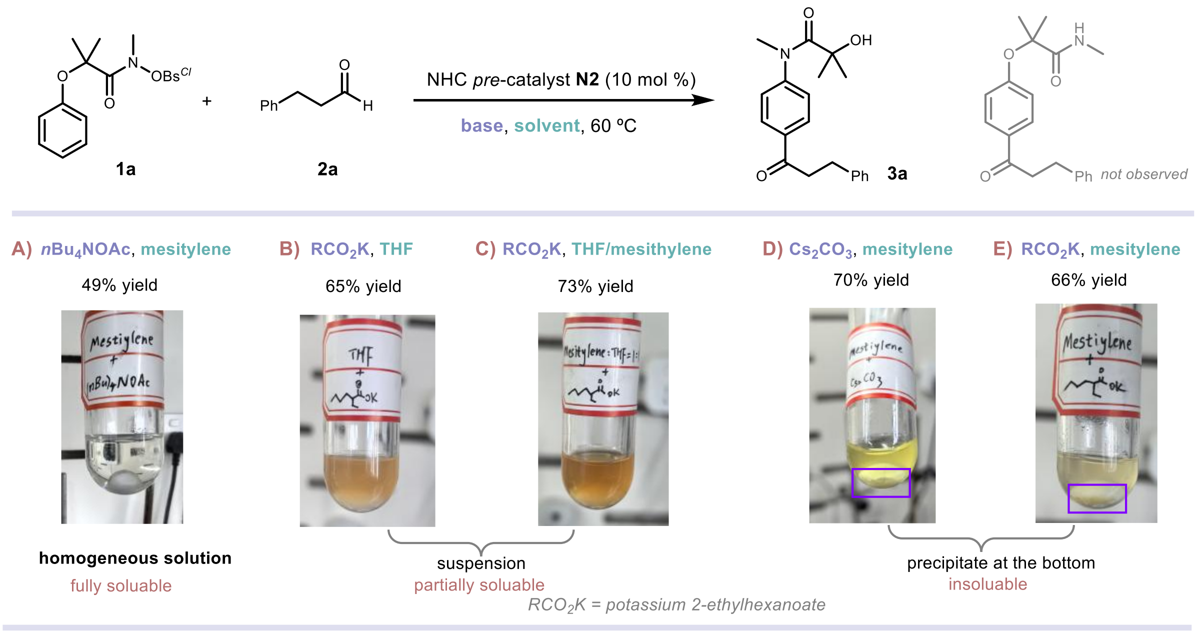

Subsequently, we evaluated a homogeneous system using *n*Bu$_4$OAc as the base in our computational model. As shown in the figure below, the transition state for C–N bond cleavage is higher in energy by 13.1 kcal/mol compared to that for C–O bond cleavage, in excellent agreement with the experimental observations. Notably, **this result is obtained without invoking the simplifications inherent to heterogeneous systems, yet it reproduces the same selectivity trend observed in our earlier calculations.** This consistency indicates that our previously employed simplified model can be considered reliable and capable of providing robust support for the experimentally observed selectivity.

Furthermore, we performed additional DFT calculations to examine different base/solvent combinations in heterogeneous systems, in order to evaluate the generality of the computational approach. Gratifyingly, the calculated energy profiles for the C–N versus C–O bond cleavage pathways show consistent trends across these different conditions, with energy barrier differences remaining greater than 10 kcal/mol in all cases. These results further support the robustness and reliability of our computational methodology.

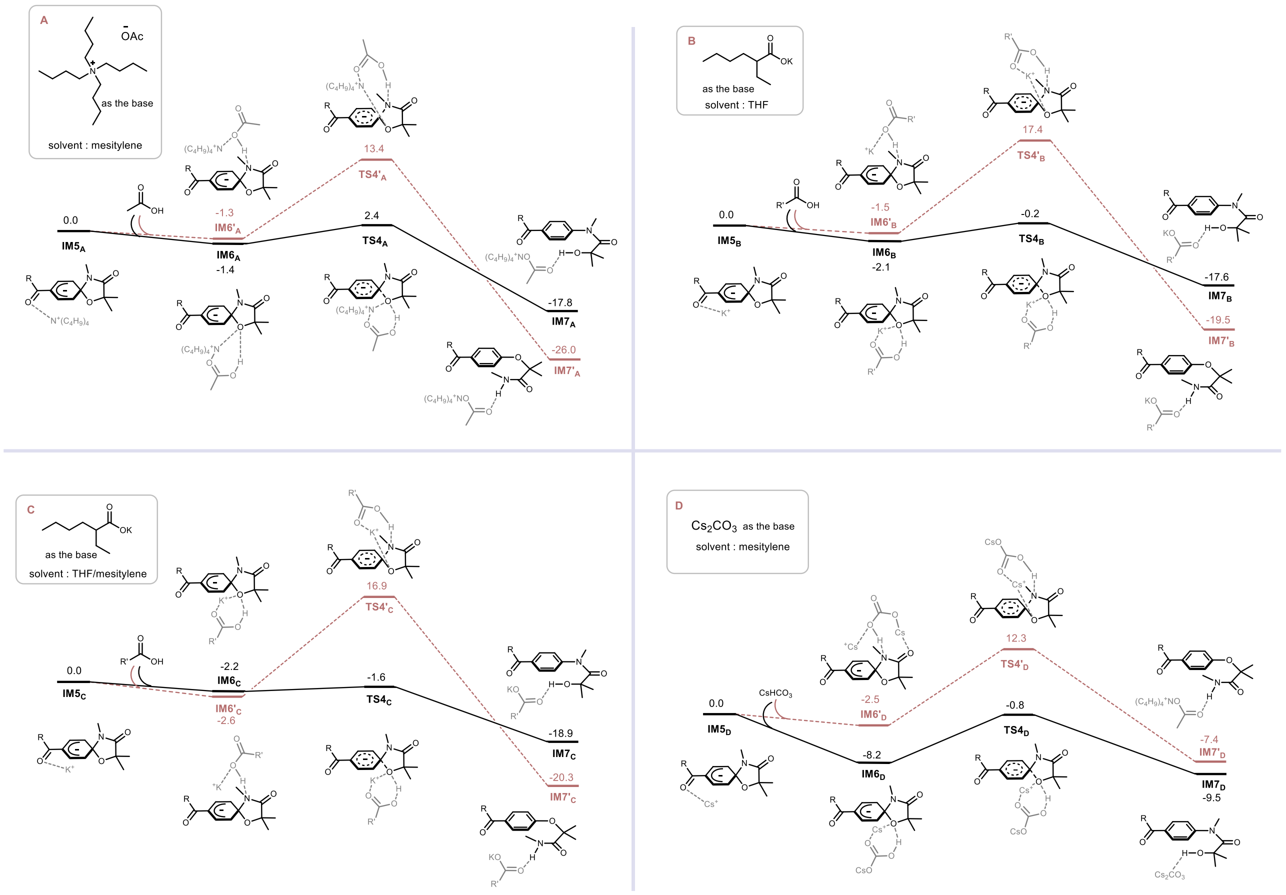

Accordingly, the above experimental results and their discussion have been included in Section 2 of the Supplementary Information. The corresponding DFT calculations and analysis of the simplified computational model have been added as a new Figure S7 in Section 10 of the Supplementary Information. In addition, the statement "Due to the heterogeneous nature of K_2CO_3 in mesitylene, a simplified calculation model that assumes a freely solvated carbonate anion was used (see Figure S7 for discussion)." has been incorporated into the revised manuscript. All modifications have been clearly marked in the revised files.

We sincerely appreciate the reviewer's highly constructive comments. We also thank Reviewer 1 for the detailed feedback in the second round of review. We have carefully addressed all concerns and sincerely hope that the revised manuscript is now suitable for publication in *Nature Communications*.

Reviewer #2:

1. Comment: The authors have addressed my comments satisfactorily. The radar plot presentation has been improved and the criteria for the sensitivity analysis are now clearly defined.

The computational section has been strengthened substantially. The authors now provide results without D3, incorporate counterions to better reflect experimental conditions, and demonstrate that the energetic trends are consistent across these methodological choices. In addition, the inclusion of charge/spin-density analyses improves confidence in the assignments of radical and zwitterionic intermediates and transition states.

The mechanistic discussion around TS4 vs TS4' has been appropriately revised to reflect what is supported by the updated model, replacing the prior hyperconjugation-centered argument with a clearer, more defensible rationale based on bond metrics and ring distortion.

Overall, I believe the manuscript is now in good shape and I recommend to be accepted.

Response: We are grateful to the reviewer for the positive assessment and for acknowledging the substantial improvements made to the manuscript. We are pleased that the revisions to the radar plot, the clarification of the sensitivity criteria, and the strengthened computational studies, have addressed the concerns of this reviewer. We also appreciate the feedback on the mechanistic discussion, which we have revised to provide a clearer rationale based on the updated model. Thank you for recommending the manuscript for acceptance.

Reviewer #3:

1. Comment: I am satisfied with current revised version. The manuscript can be accepted for publication.

Response: Thank you very much for your time and valuable feedback. We are delighted to hear that you are satisfied with the revised manuscript and appreciate your recommendation for acceptance. We hope the final version meets the journal's standards and will be of interest to the readership.

Reviewer #4:

1. Comment: The authors have made meticulous efforts to address essentially all reviewers' comments. I have no additional revision requests. The revision is good to go.

Response: Many thanks for your thorough evaluation and for acknowledging the meticulous efforts we devoted to addressing the reviewers' feedback. We deeply appreciate your endorsement of the revised manuscript and are grateful for your support.
